# Supplementary material for: The bovine alveolar macrophage DNA methylome is resilient to infection with Mycobacterium bovis
Source: Sci Rep. 2019 Feb 6;9:1510. doi: 10.1038/s41598-018-37618-z (PMC6365515; doi:10.1038/s41598-018-37618-z)
Supplement: Supplementary file 1 [file 41598_2018_37618_MOESM1_ESM.pdf]

Supporting information

The bovine alveolar macrophage DNA methylome is resilient to infection with *Mycobacterium bovis*

Running Title: Impact of mycobacterial infection on DNA methylation

Alan Mark O'Doherty [1,*,¶], Kevin Rue-Albrecht [2,¶], David Andrew Magee [1], Simone Ahting [3], Rachelle Elizabeth Irwin [4], Thomas Johnathan Hall [1], John Arthur Browne [1], Nicolas Claude Nalpas [5], Colum Patrick Walsh [4], Stephen Vincent Gordon [6,7], Marcin Wlodzimierz Wojewodzic [8] and David Evan MacHugh [1,7]

Supplementary figure 1. DNA methylation profiles at imprinted loci. Red line = infected; Blue line = control. Average methylation is represented on the y-axis (0.2 = 20%, 0.5 = 50% and 0.8 = 80%).

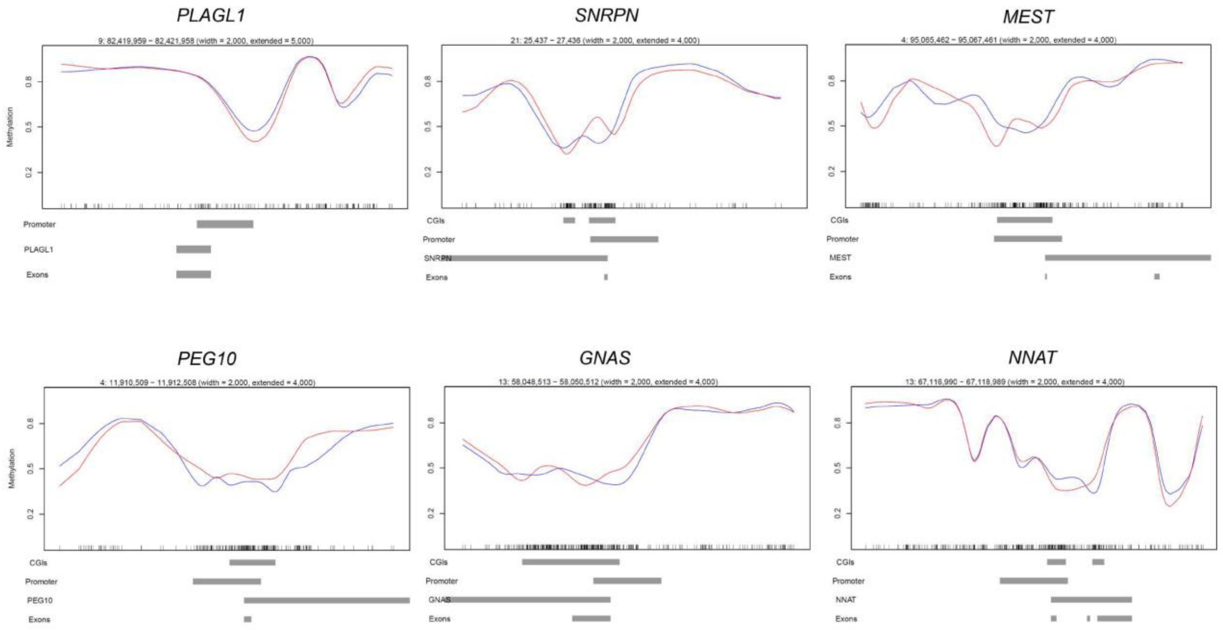

Supplementary file 1. Collated reports of the Bismark pipeline leading to the final methylation calls.

Bismark Processing Report

trimgalore/Merged/C1_ATCACG_R1_merged_val_1.fq.gz and trimgalore/Merged/C1_ATCACG_R2_merged_val_2.fq.gz

Data processed at 16:37 on 2016-05-01

Alignment

Sequence pairs analysed in total	57521034
Paired-end alignments with a unique best hit	38884464
Pairs without alignments under any condition	9677196
Pairs that did not map uniquely	8959374
Genomic sequence context not extractable (edges of chromosomes)	214

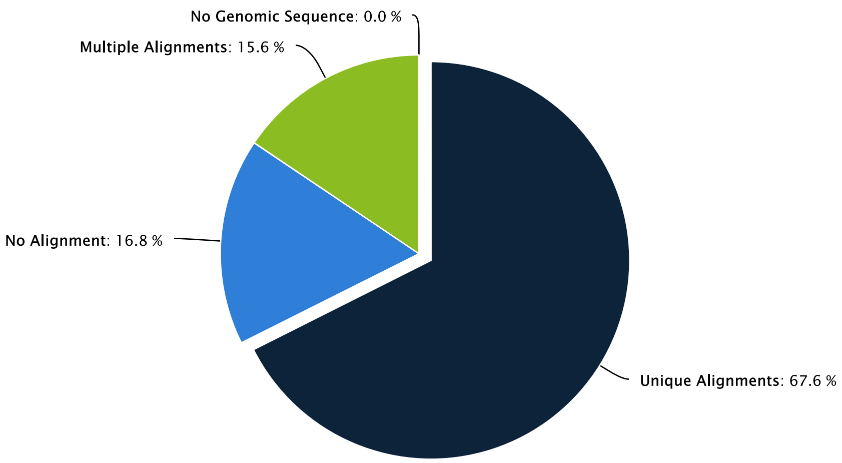

Cytosine Methylation

Total C's analysed	1998531151
Methylated C's in CpG context	95544165
Methylated C's in CHG context	7485859
Methylated C's in CHH context	10715405
Methylated C's in Unknown context	7908
Unmethylated C's in CpG context	36603820
Unmethylated C's in CHG context	485029377
Unmethylated C's in CHH context	1363152525
Unmethylated C's in Unknown context	23175
Percentage methylation (CpG context)	72.3%
Percentage methylation (CHG context)	1.5%
Percentage methylation (CHH context)	0.8%

**Methylated C's in Unknown context**	N/A%

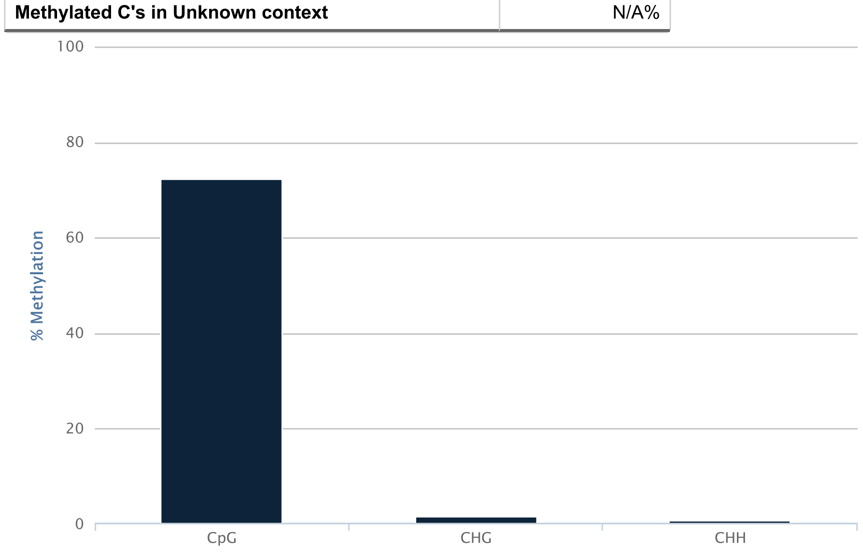

Alignment to Individual Bisulfite Strands

**OT**	19446435	original top strand
**CTOT**	0	complementary to original top strand
**CTOB**	0	complementary to original bottom strand
**OB**	19437815	original bottom strand

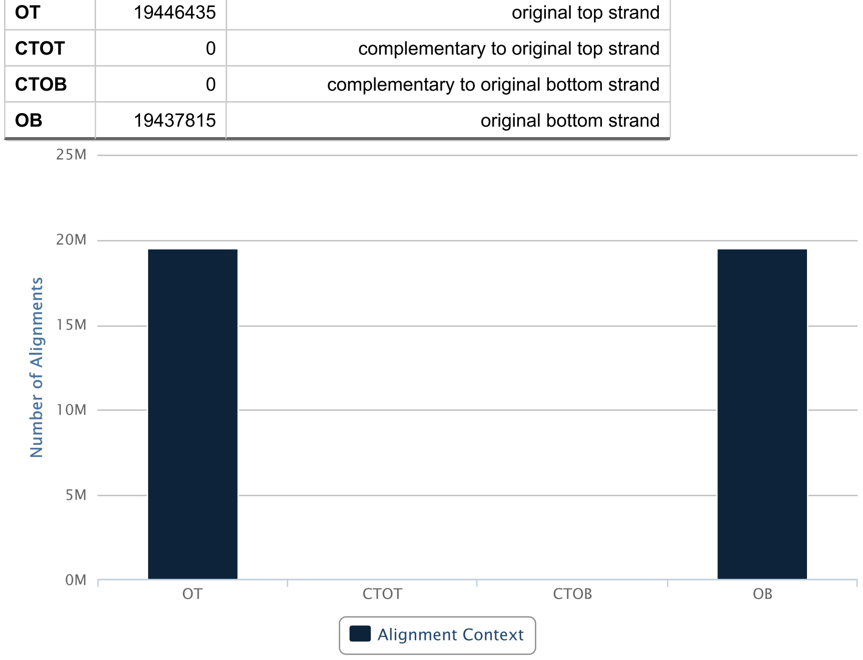

Deduplication

**Alignments analysed**	38884250
**Unique alignments**	36517624
**Duplicates removed**	2366626
Duplicated alignments were found at **2027957** different positions	

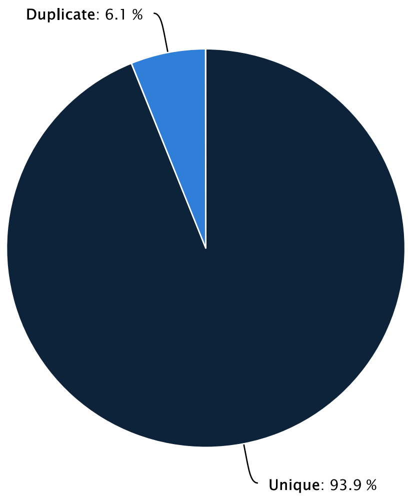

Cytosine Methylation after Extraction

**Total C's analysed**	1089177222
**Methylated C's in CpG context**	48019052
**Methylated C's in CHG context**	2172046
**Methylated C's in CHH context**	3922167
**Unmethylated C's in CpG context**	18467502
**Unmethylated C's in CHG context**	258822577
**Unmethylated C's in CHH context**	757773878
**Percentage methylation (CpG context)**	72.2%
**Percentage methylation (CHG context)**	0.8%
**Percentage methylation (CHH context)**	0.5%

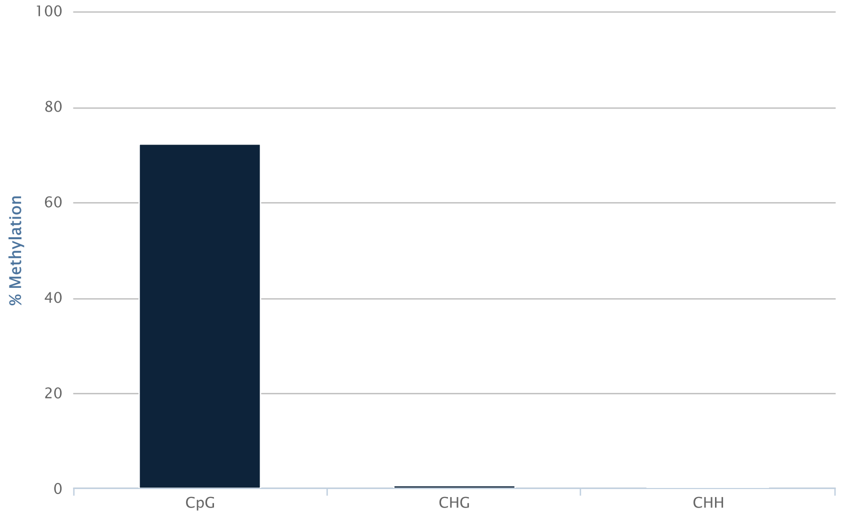

Nucleotide Coverage

Nucleotide Class	Counts Sample	Counts Genome	% in Sample	% in Genome
**A**	2440759345	769902373	28.29	29.06
**T**	2282467446	771539118	26.45	29.12
**C**	1819760772	553941589	21.09	20.91
**G**	2086172379	554298949	24.18	20.92
**AC**	433381200	134825334	5.06	5.09
**CA**	634931486	194999195	7.42	7.36
**TC**	498204323	168302551	5.82	6.35

Nucleotide Class	Counts Sample	Counts Genome	% in Sample	% in Genome
**CT**	581601044	189605870	6.80	7.16
**CC**	466381544	141780183	5.45	5.35
**CG**	120202250	27540367	1.40	1.04
**GC**	406912022	109014748	4.75	4.11
**GG**	594405910	141809778	6.95	5.35
**AG**	677622317	189448103	7.92	7.15
**GA**	602184141	168131316	7.04	6.35
**TG**	677372619	195484730	7.92	7.38
**GT**	457608178	135324159	5.35	5.11
**TT**	649192839	248110054	7.59	9.36
**TA**	441645383	159621424	5.16	6.02
**AT**	578296977	198476252	6.76	7.49
**AA**	738012337	247130183	8.62	9.33

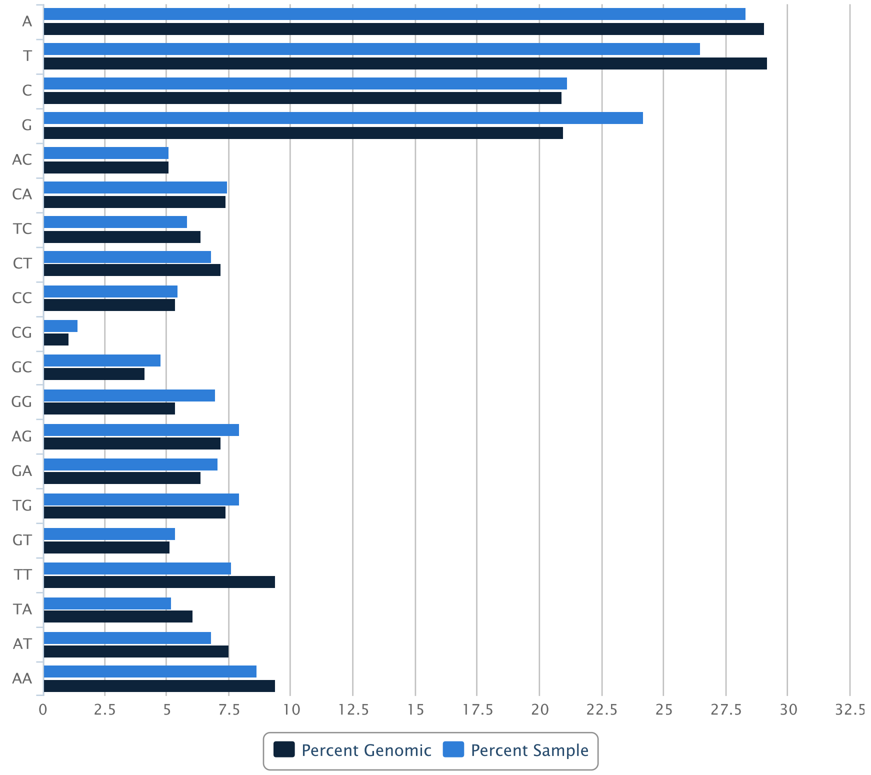

M-Bias Plot

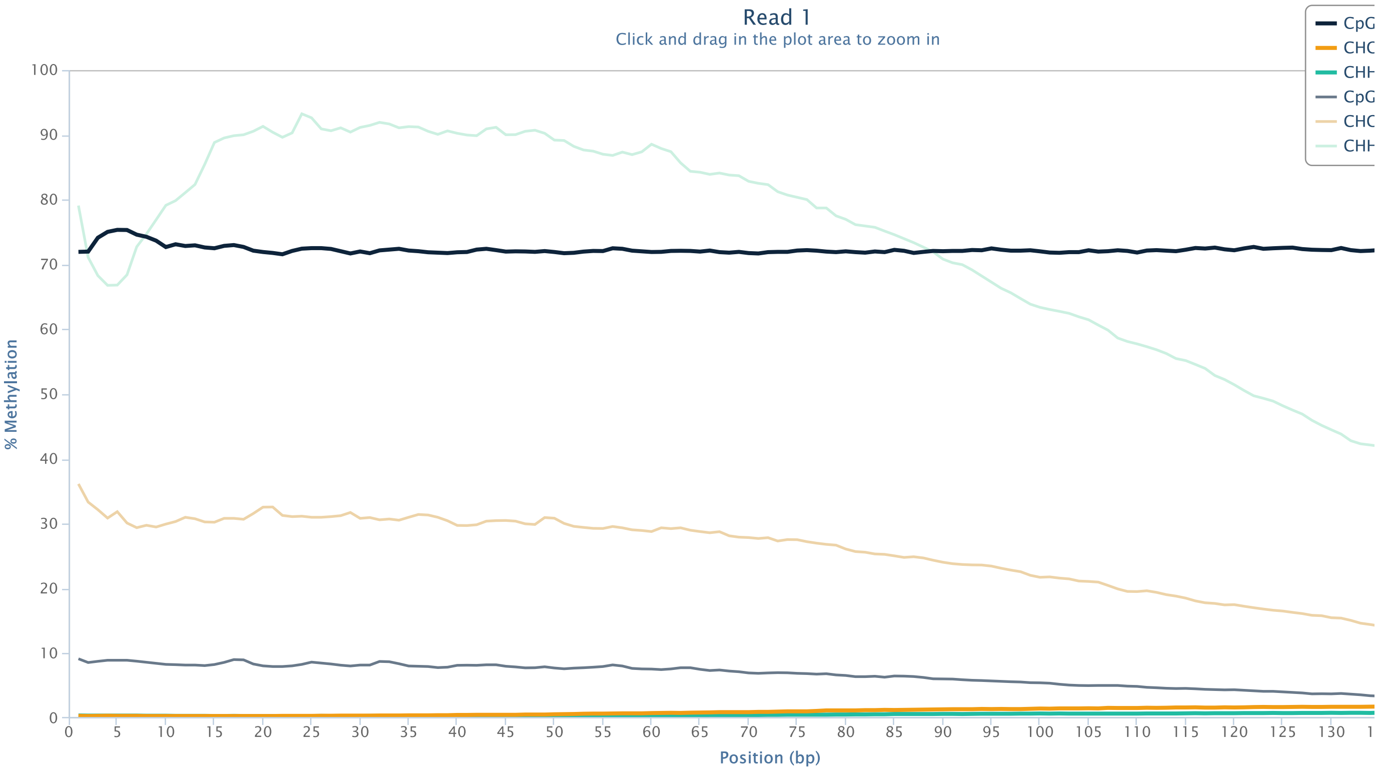

Analysis produced by **Bismark** (version v0.15.0) - a tool to map bisulfite converted sequence reads and determine cytosine methylation states

Report graphs rendered using jQuery and Highcharts. Page design by Phil Ewels.

Bismark Processing Report

trimgalore/Merged/C4_TGACCA_R1_merged_val_1.fq.gz and trimgalore/Merged/C4_TGACCA_R2_merged_val_2.fq.gz

Data processed at 16:37 on 2016-05-01

Alignment

**Sequence pairs analysed in total**	58847507
**Paired-end alignments with a unique best hit**	38051076
**Pairs without alignments under any condition**	10926328
**Pairs that did not map uniquely**	9870103
**Genomic sequence context not extractable (edges of chromosomes)**	266

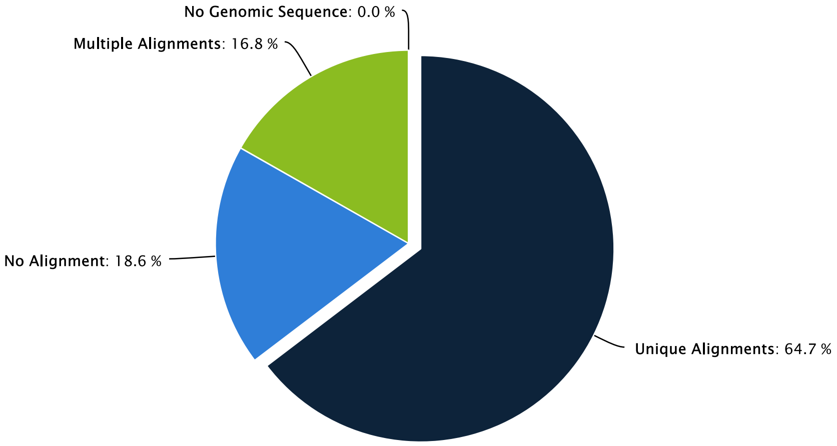

Cytosine Methylation

**Total C's analysed**	2012866865
**Methylated C's in CpG context**	104586167
**Methylated C's in CHG context**	8069645
**Methylated C's in CHH context**	12013265
**Methylated C's in Unknown context**	10267
**Unmethylated C's in CpG context**	38540768
**Unmethylated C's in CHG context**	493409202
**Unmethylated C's in CHH context**	1356247818
**Unmethylated C's in Unknown context**	25829
**Percentage methylation (CpG context)**	73.1%
**Percentage methylation (CHG context)**	1.6%
**Percentage methylation (CHH context)**	0.9%

**Methylated C's in Unknown context**	N/A%

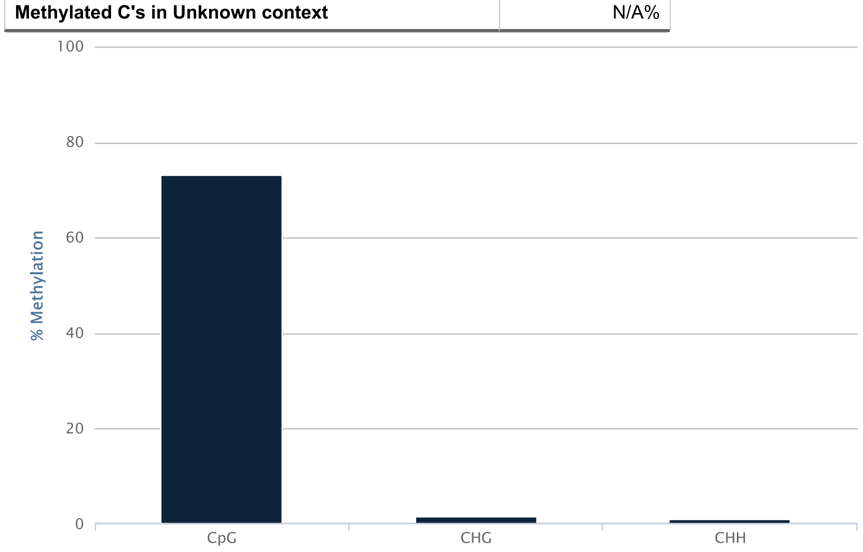

Alignment to Individual Bisulfite Strands

**OT**	19029515	original top strand
**CTOT**	0	complementary to original top strand
**CTOB**	0	complementary to original bottom strand
**OB**	19021295	original bottom strand

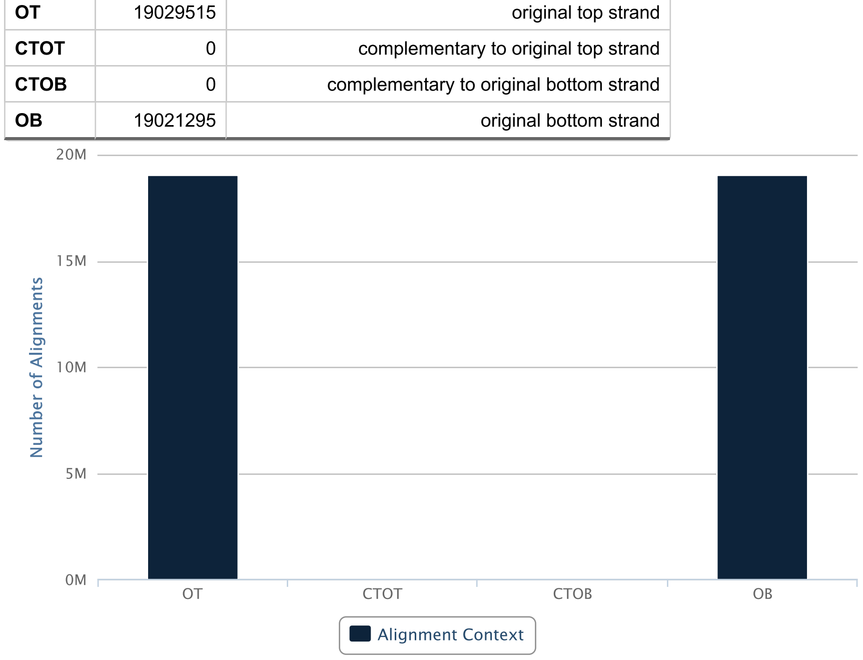

Deduplication

**Alignments analysed**	38050810
**Unique alignments**	34987583
**Duplicates removed**	3063227
Duplicated alignments were found at **2556068** different positions	

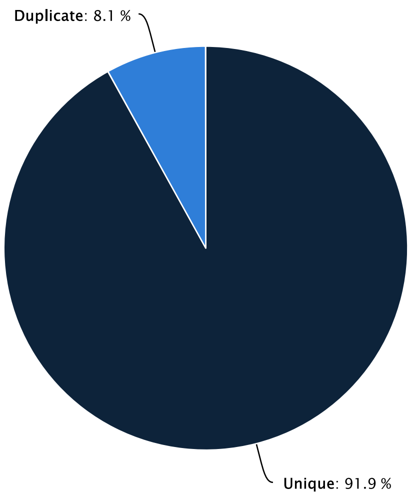

Cytosine Methylation after Extraction

**Total C's analysed**	1106697321
**Methylated C's in CpG context**	52567138
**Methylated C's in CHG context**	2309250
**Methylated C's in CHH context**	4695622
**Unmethylated C's in CpG context**	19508156
**Unmethylated C's in CHG context**	266353526
**Unmethylated C's in CHH context**	761263629
**Percentage methylation (CpG context)**	72.9%
**Percentage methylation (CHG context)**	0.9%
**Percentage methylation (CHH context)**	0.6%

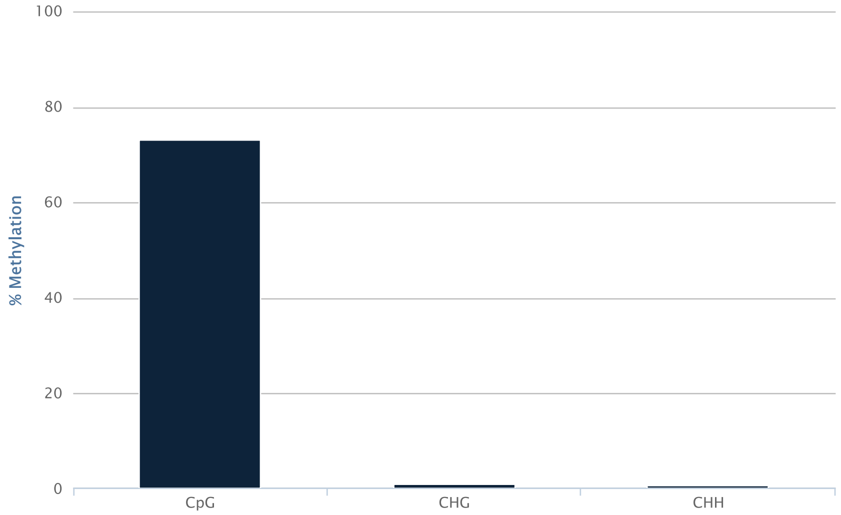

Nucleotide Coverage

Nucleotide Class	Counts Sample	Counts Genome	% in Sample	% in Genome
**A**	2447607806	769902373	28.23	29.06
**T**	2255670961	771539118	26.01	29.12
**C**	1828710427	553941589	21.09	20.91
**G**	2139032811	554298949	24.67	20.92
**AC**	435055084	134825334	5.06	5.09
**CA**	637107403	194999195	7.41	7.36
**TC**	494254726	168302551	5.75	6.35

Nucleotide Class	Counts Sample	Counts Genome	% in Sample	% in Genome
**CT**	577625927	189605870	6.72	7.16
**CC**	467676706	141780183	5.44	5.35
**CG**	129825342	27540367	1.51	1.04
**GC**	417784013	109014748	4.86	4.11
**GG**	617767330	141809778	7.18	5.35
**AG**	690121779	189448103	8.02	7.15
**GA**	616866262	168131316	7.17	6.35
**TG**	683864658	195484730	7.95	7.38
**GT**	462529297	135324159	5.38	5.11
**TT**	629040040	248110054	7.31	9.36
**TA**	432681230	159621424	5.03	6.02
**AT**	571738979	198476252	6.65	7.49
**AA**	737610854	247130183	8.58	9.33

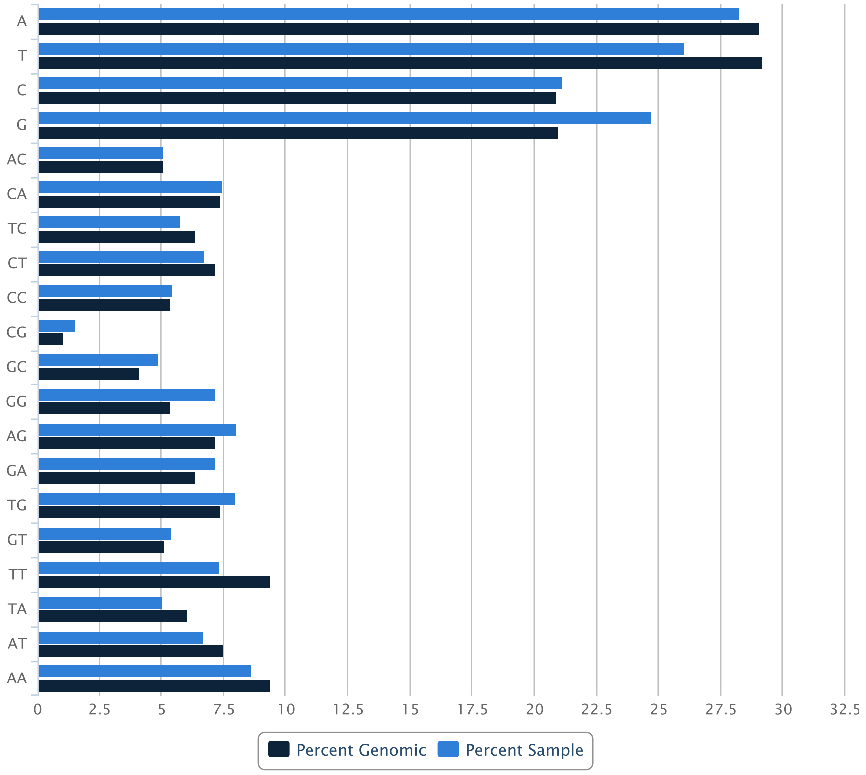

M-Bias Plot

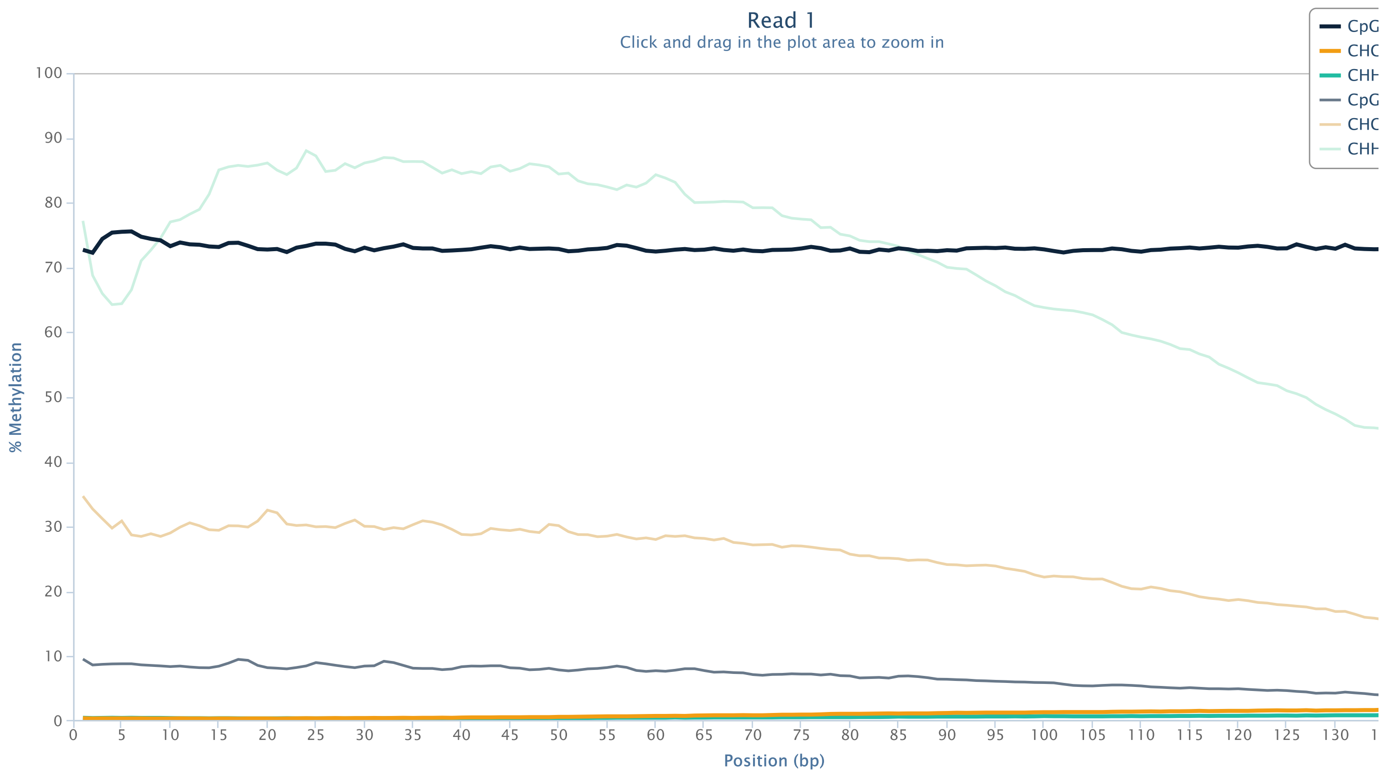

Analysis produced by **Bismark** (version v0.15.0) - a tool to map bisulfite converted sequence reads and determine cytosine methylation states

Report graphs rendered using jQuery and Highcharts. Page design by Phil Ewels.

Bismark Processing Report

trimgalore/Merged/C5_CAGATC_R1_merged_val_1.fq.gz and trimgalore/Merged/C5_CAGATC_R2_merged_val_2.fq.gz

Data processed at 16:37 on 2016-05-01

Alignment

**Sequence pairs analysed in total**	66078568
**Paired-end alignments with a unique best hit**	42653335
**Pairs without alignments under any condition**	12327453
**Pairs that did not map uniquely**	11097780
**Genomic sequence context not extractable (edges of chromosomes)**	269

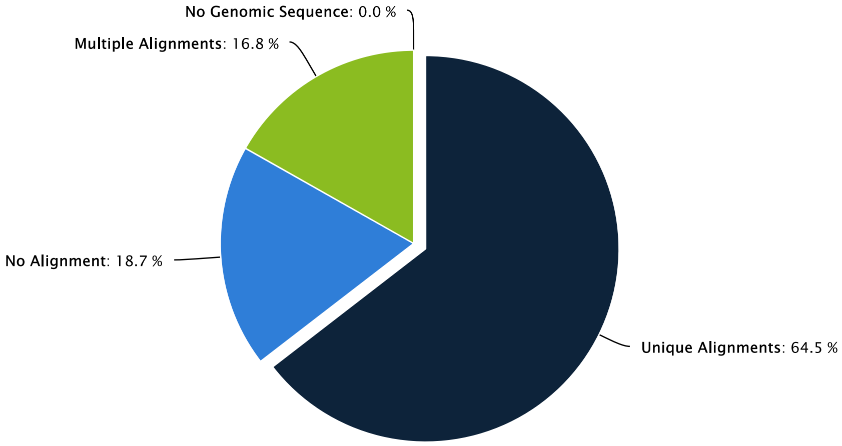

Cytosine Methylation

**Total C's analysed**	2206703733
**Methylated C's in CpG context**	113325512
**Methylated C's in CHG context**	9134408
**Methylated C's in CHH context**	13018965
**Methylated C's in Unknown context**	11337
**Unmethylated C's in CpG context**	43073304
**Unmethylated C's in CHG context**	542700997
**Unmethylated C's in CHH context**	1485450547
**Unmethylated C's in Unknown context**	28208
**Percentage methylation (CpG context)**	72.5%
**Percentage methylation (CHG context)**	1.7%
**Percentage methylation (CHH context)**	0.9%

**Methylated C's in Unknown context**	N/A%

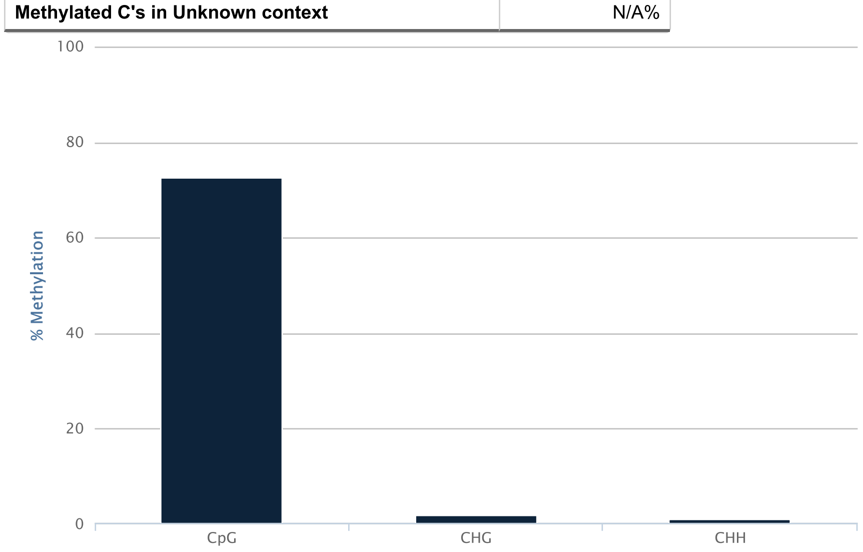

Alignment to Individual Bisulfite Strands

**OT**	21327841	original top strand
**CTOT**	0	complementary to original top strand
**CTOB**	0	complementary to original bottom strand
**OB**	21325225	original bottom strand

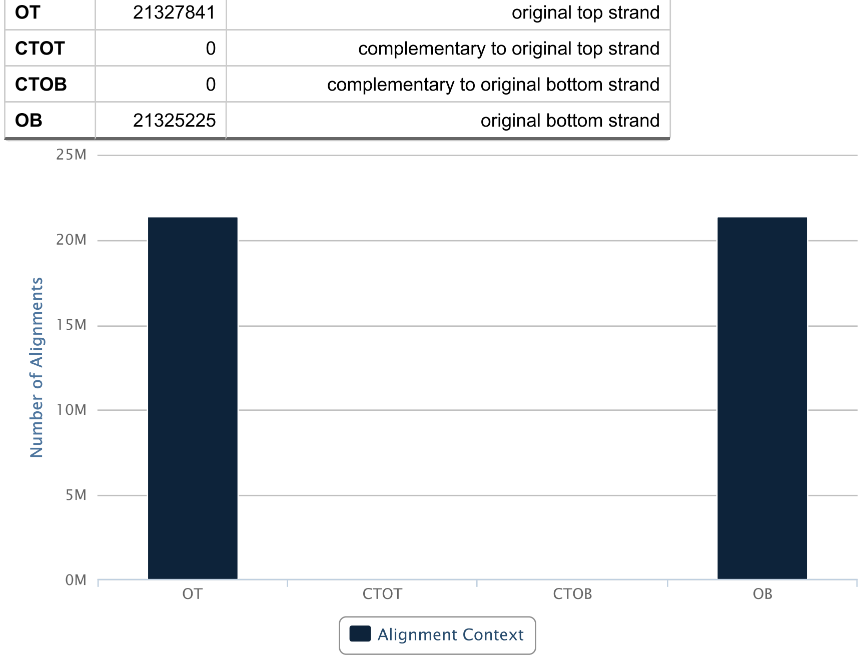

Deduplication

**Alignments analysed**	42653066
**Unique alignments**	38671531
**Duplicates removed**	3981535
Duplicated alignments were found at **3346166** different positions	

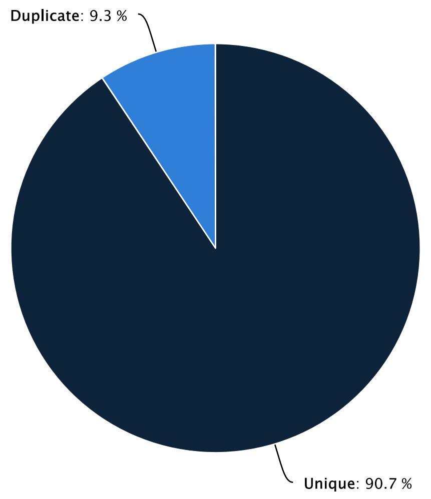

Cytosine Methylation after Extraction

**Total C's analysed**	1182168496
**Methylated C's in CpG context**	55511497
**Methylated C's in CHG context**	2519663
**Methylated C's in CHH context**	4831767
**Unmethylated C's in CpG context**	21347875
**Unmethylated C's in CHG context**	285742081
**Unmethylated C's in CHH context**	812215613
**Percentage methylation (CpG context)**	72.2%
**Percentage methylation (CHG context)**	0.9%
**Percentage methylation (CHH context)**	0.6%

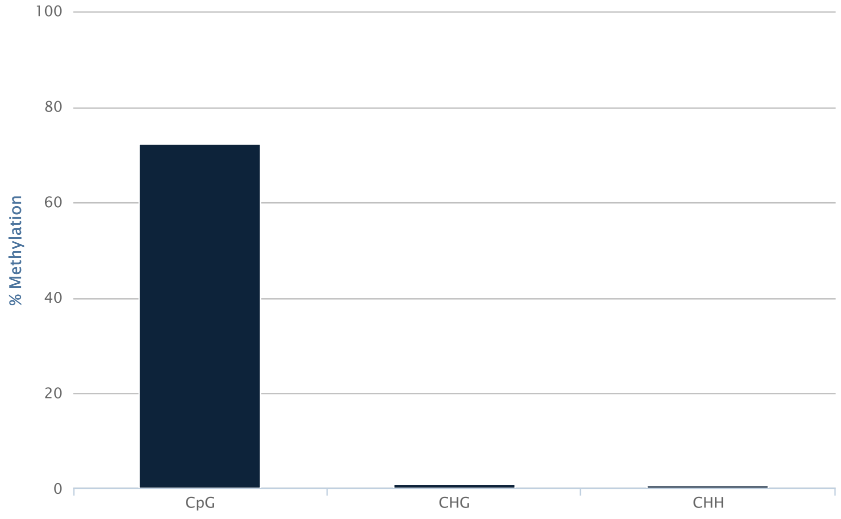

Nucleotide Coverage

Nucleotide Class	Counts Sample	Counts Genome	% in Sample	% in Genome
**A**	2674651655	769902373	28.17	29.06
**T**	2463438374	771539118	25.95	29.12
**C**	2003994519	553941589	21.11	20.91
**G**	2351902403	554298949	24.77	20.92
**AC**	475959525	134825334	5.05	5.09
**CA**	698225743	194999195	7.42	7.36
**TC**	539480403	168302551	5.73	6.35

Nucleotide Class	Counts Sample	Counts Genome	% in Sample	% in Genome
CT	631837924	189605870	6.71	7.16
CC	513481468	141780183	5.45	5.35
CG	141884327	27540367	1.51	1.04
GC	459461432	109014748	4.88	4.11
GG	682337434	141809778	7.25	5.35
AG	757639824	189448103	8.05	7.15
GA	674920547	168131316	7.17	6.35
TG	750331102	195484730	7.97	7.38
GT	508284149	135324159	5.40	5.11
TT	684505689	248110054	7.27	9.36
TA	471470373	159621424	5.01	6.02
AT	622302204	198476252	6.61	7.49
AA	803905114	247130183	8.54	9.33

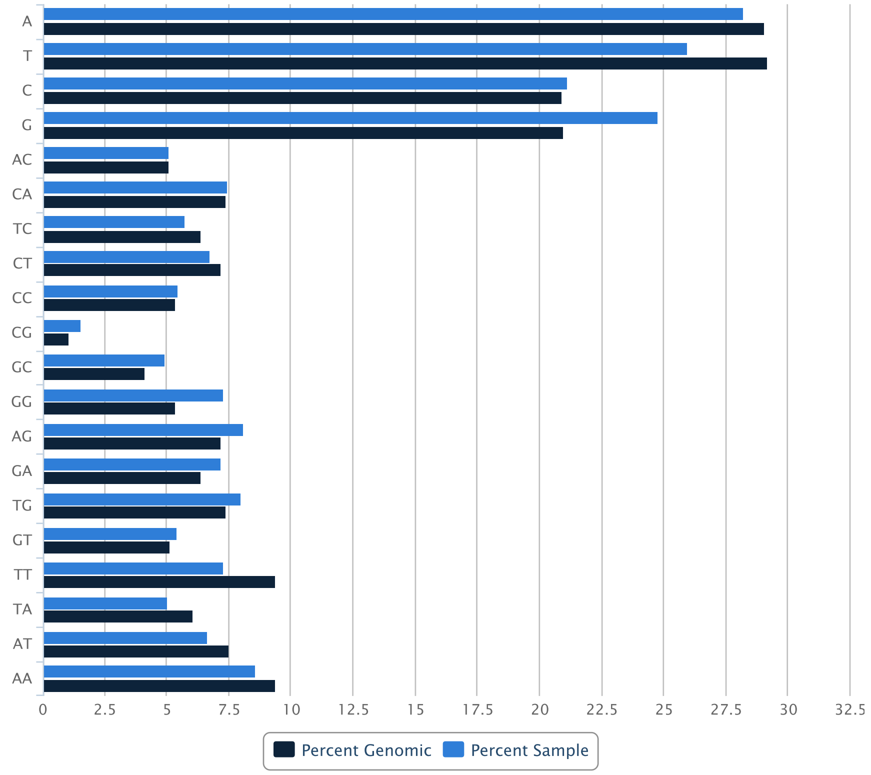

M-Bias Plot

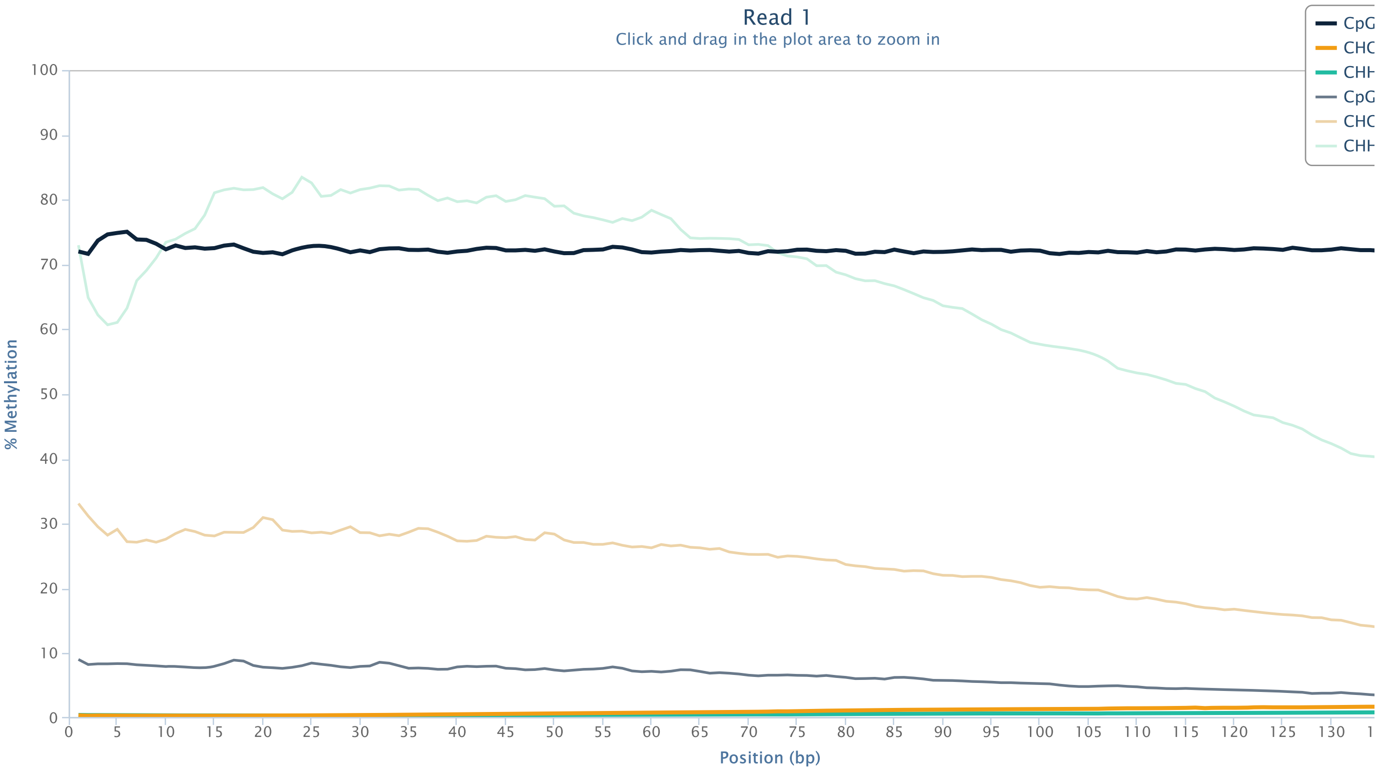
Read 1
Click and drag in the plot area to zoom in
% Methylation
Position (bp)

Bismark Processing Report

trimgalore/Merged/C6_TAGCTT_R1_merged_val_1.fq.gz and trimgalore/Merged/C6_TAGCTT_R2_merged_val_2.fq.gz

Data processed at 16:37 on 2016-05-01

Alignment

**Sequence pairs analysed in total**	48774732
**Paired-end alignments with a unique best hit**	32343579
**Pairs without alignments under any condition**	9031412
**Pairs that did not map uniquely**	7399741
**Genomic sequence context not extractable (edges of chromosomes)**	261

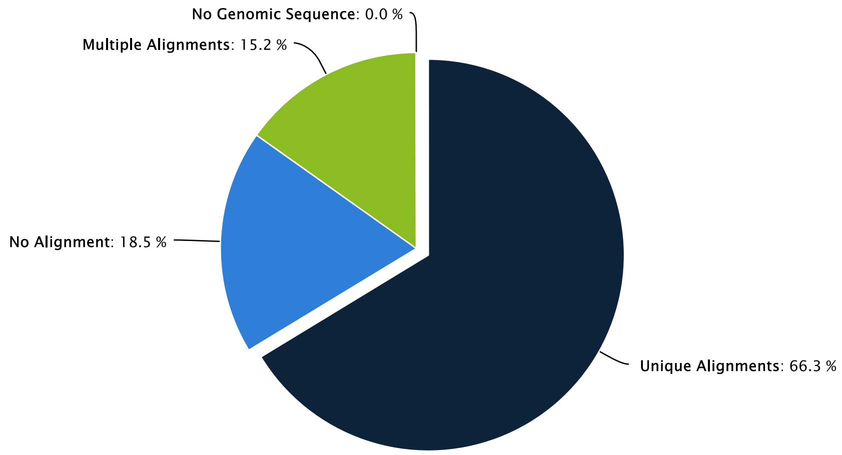

Cytosine Methylation

**Total C's analysed**	1755829252
**Methylated C's in CpG context**	83242824
**Methylated C's in CHG context**	6174578
**Methylated C's in CHH context**	9449130
**Methylated C's in Unknown context**	7143
**Unmethylated C's in CpG context**	30117478
**Unmethylated C's in CHG context**	419467949
**Unmethylated C's in CHH context**	1207377293
**Unmethylated C's in Unknown context**	20220
**Percentage methylation (CpG context)**	73.4%
**Percentage methylation (CHG context)**	1.5%
**Percentage methylation (CHH context)**	0.8%

**Methylated C's in Unknown context**	N/A%

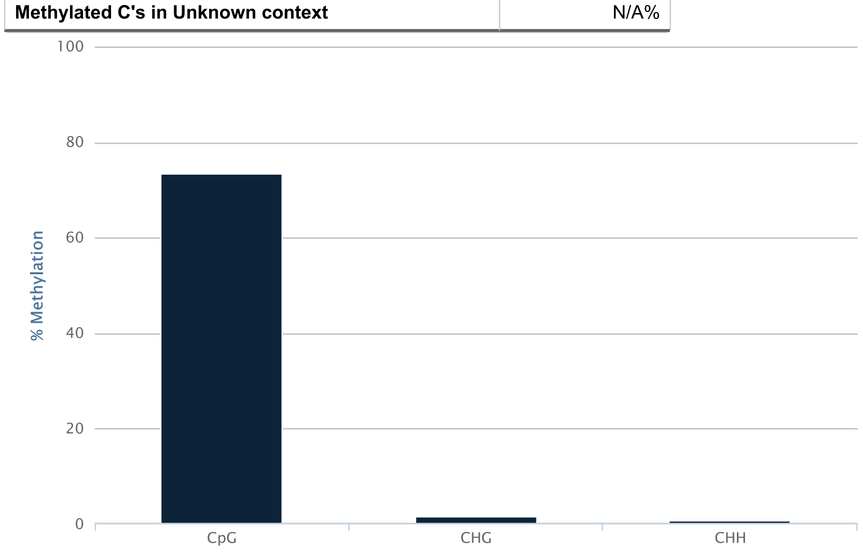

Alignment to Individual Bisulfite Strands

**OT**	16177511	original top strand
**CTOT**	0	complementary to original top strand
**CTOB**	0	complementary to original bottom strand
**OB**	16165807	original bottom strand

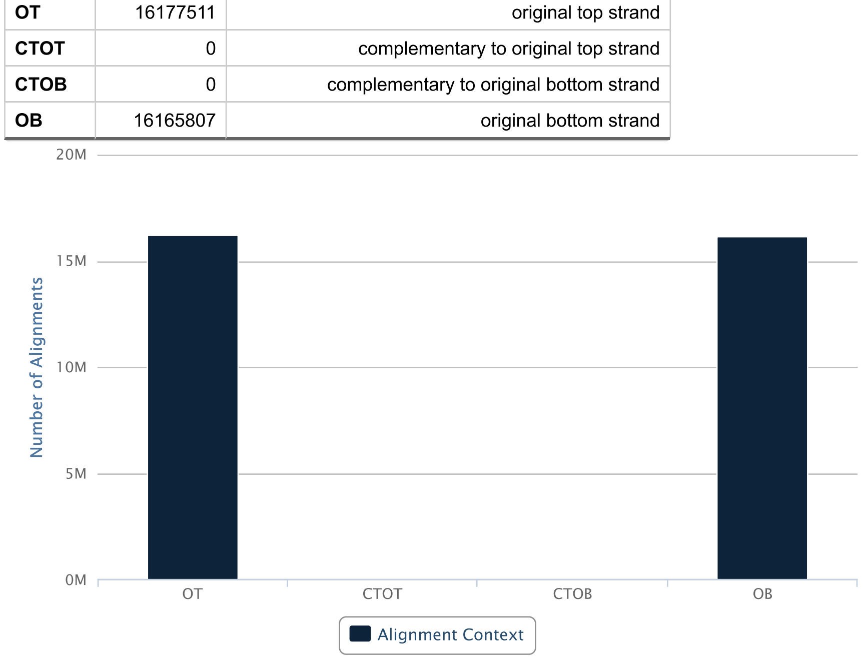

Deduplication

**Alignments analysed**	32343318
**Unique alignments**	29310188
**Duplicates removed**	3033130
Duplicated alignments were found at **2630692** different positions	

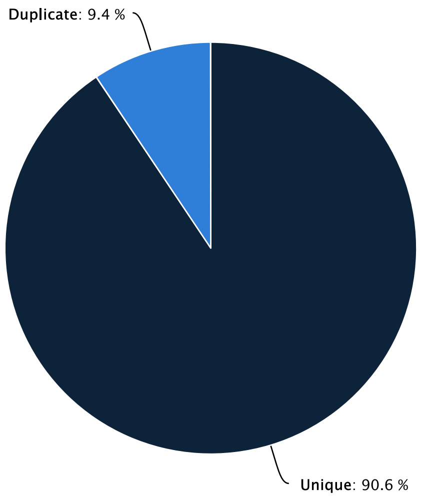

Cytosine Methylation after Extraction

**Total C's analysed**	969940383
**Methylated C's in CpG context**	42905336
**Methylated C's in CHG context**	1735765
**Methylated C's in CHH context**	3511285
**Unmethylated C's in CpG context**	15588532
**Unmethylated C's in CHG context**	227713671
**Unmethylated C's in CHH context**	678485794
**Percentage methylation (CpG context)**	73.4%
**Percentage methylation (CHG context)**	0.8%
**Percentage methylation (CHH context)**	0.5%

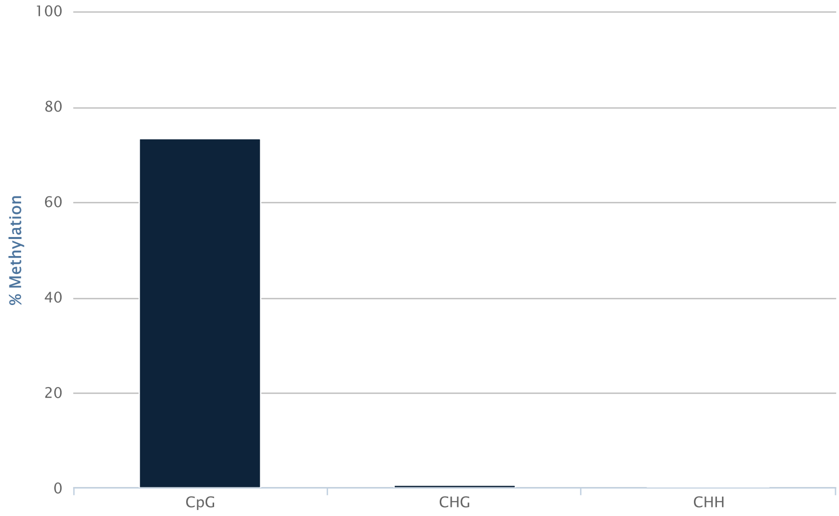

Nucleotide Coverage

Nucleotide Class	Counts Sample	Counts Genome	% in Sample	% in Genome
**A**	2129598022	769902373	28.47	29.06
**T**	1997256724	771539118	26.71	29.12
**C**	1578595678	553941589	21.11	20.91
**G**	1773455630	554298949	23.71	20.92
**AC**	378454184	134825334	5.10	5.09
**CA**	552889855	194999195	7.45	7.36
**TC**	437698968	168302551	5.90	6.35

Nucleotide Class	Counts Sample	Counts Genome	% in Sample	% in Genome
CT	506386675	189605870	6.82	7.16
CC	403485274	141780183	5.44	5.35
CG	101777160	27540367	1.37	1.04
GC	346914398	109014748	4.68	4.11
GG	495634446	141809778	6.68	5.35
AG	580335000	189448103	7.82	7.15
GA	517675829	168131316	6.98	6.35
TG	582043884	195484730	7.84	7.38
GT	393892139	135324159	5.31	5.11
TT	573729716	248110054	7.73	9.36
TA	389812658	159621424	5.25	6.02
AT	510416259	198476252	6.88	7.49
AA	649313635	247130183	8.75	9.33

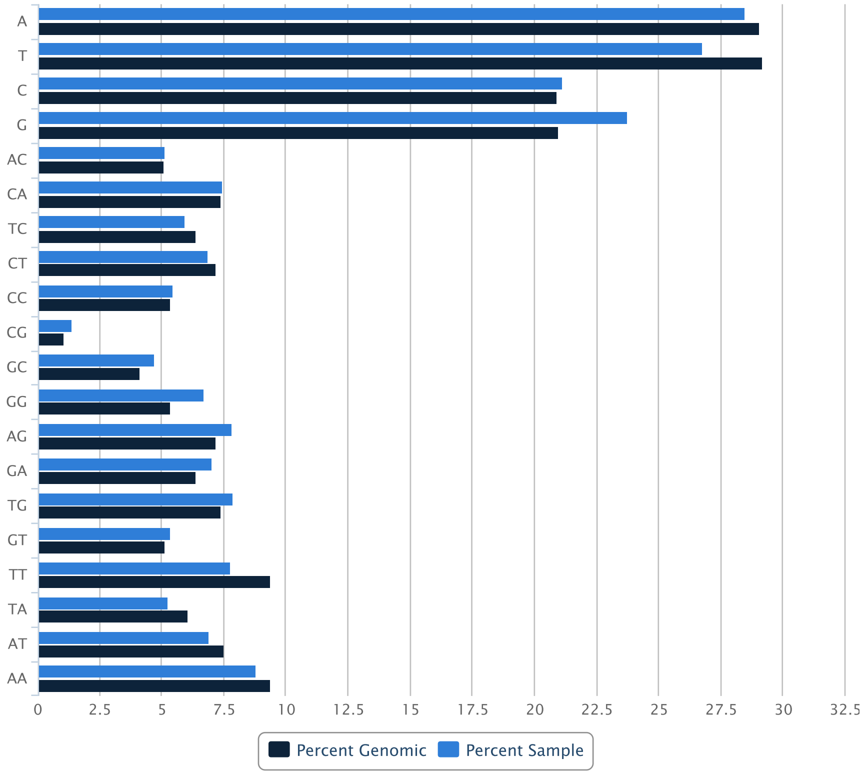

M-Bias Plot

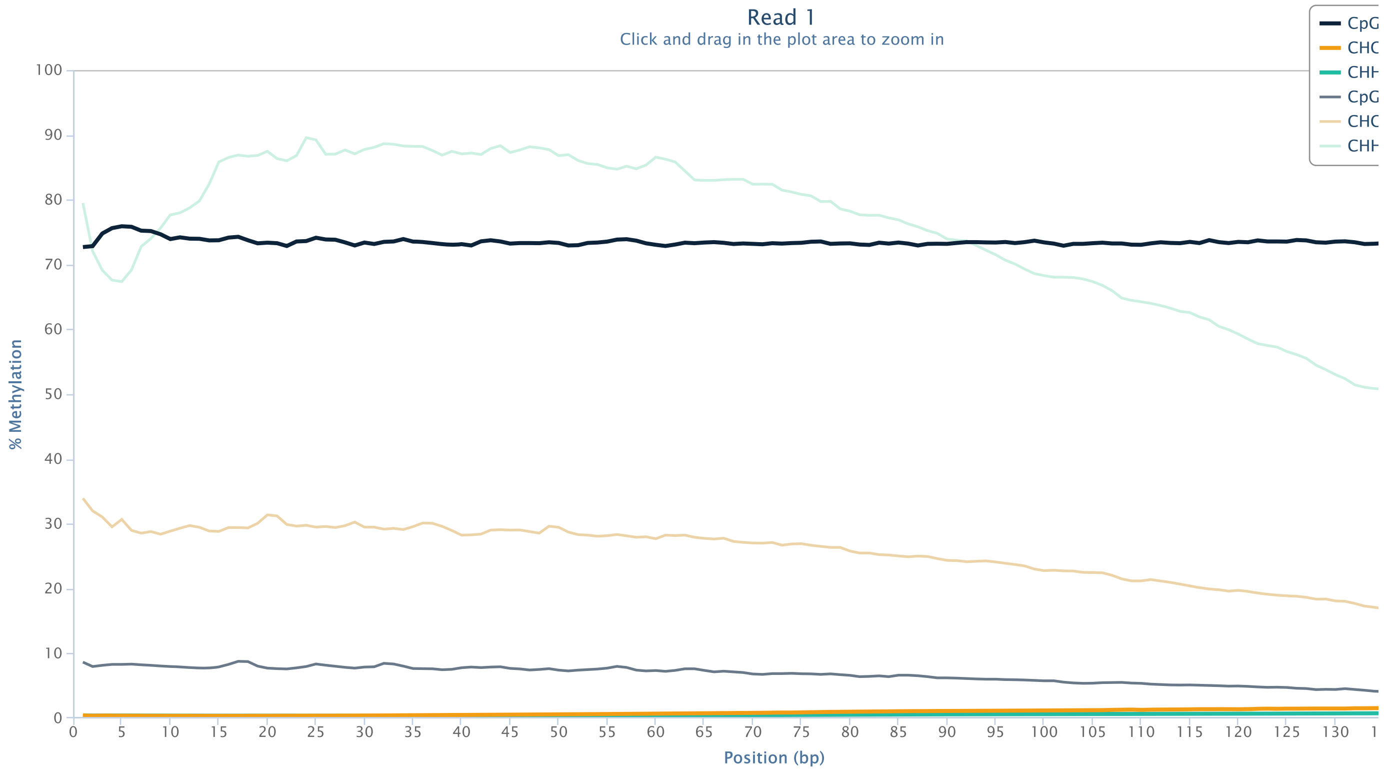

Analysis produced by **Bismark** (version v0.15.0) - a tool to map bisulfite converted sequence reads and determine cytosine methylation states

Report graphs rendered using jQuery and Highcharts. Page design by Phil Ewels.

Babraham Bioinformatics

Bismark Processing Report

trimgalore/Merged/C7_ATCACG_R1_merged_val_1.fq.gz and trimgalore/Merged/C7_ATCACG_R2_merged_val_2.fq.gz

Data processed at 16:37 on 2016-05-01

Alignment

**Sequence pairs analysed in total**	44290139
**Paired-end alignments with a unique best hit**	28847310
**Pairs without alignments under any condition**	9103927
**Pairs that did not map uniquely**	6338902
**Genomic sequence context not extractable (edges of chromosomes)**	203

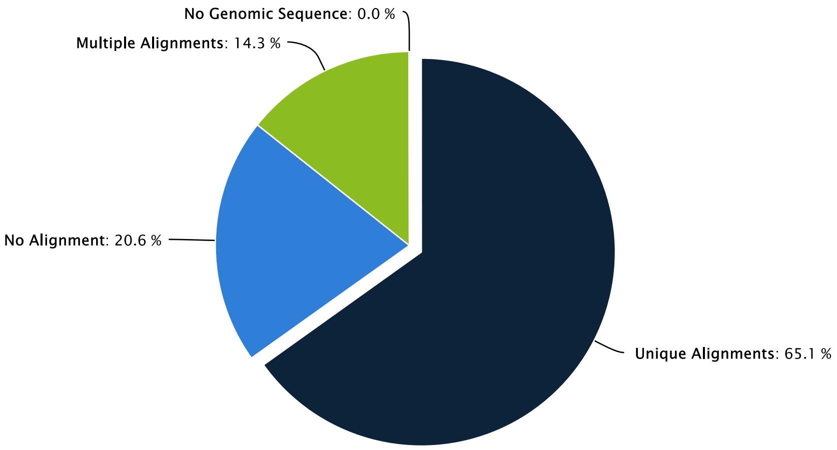

Cytosine Methylation

**Total C's analysed**	1617502049
**Methylated C's in CpG context**	81854996
**Methylated C's in CHG context**	5623945
**Methylated C's in CHH context**	8537536
**Methylated C's in Unknown context**	6155
**Unmethylated C's in CpG context**	30667320
**Unmethylated C's in CHG context**	391949309
**Unmethylated C's in CHH context**	1098868943
**Unmethylated C's in Unknown context**	18591
**Percentage methylation (CpG context)**	72.7%
**Percentage methylation (CHG context)**	1.4%
**Percentage methylation (CHH context)**	0.8%

**Methylated C's in Unknown context**	N/A%

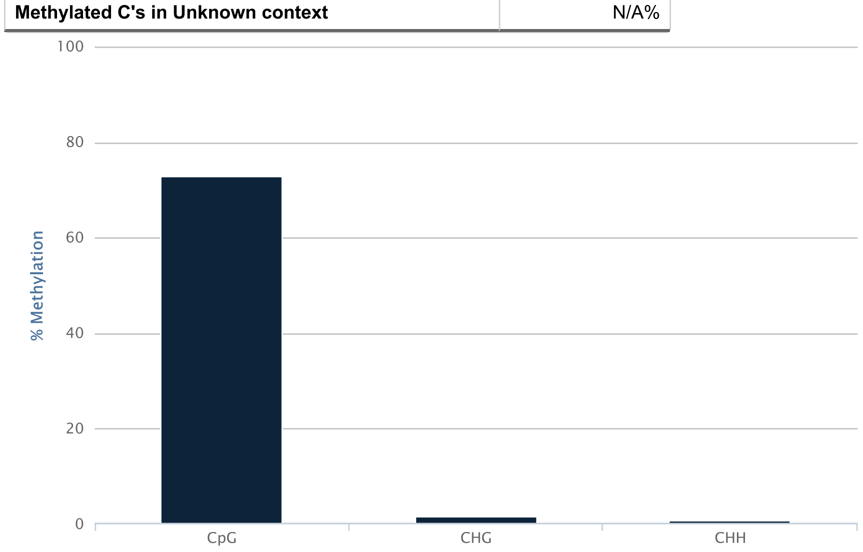

Alignment to Individual Bisulfite Strands

**OT**	14429205	original top strand
**CTOT**	0	complementary to original top strand
**CTOB**	0	complementary to original bottom strand
**OB**	14417902	original bottom strand

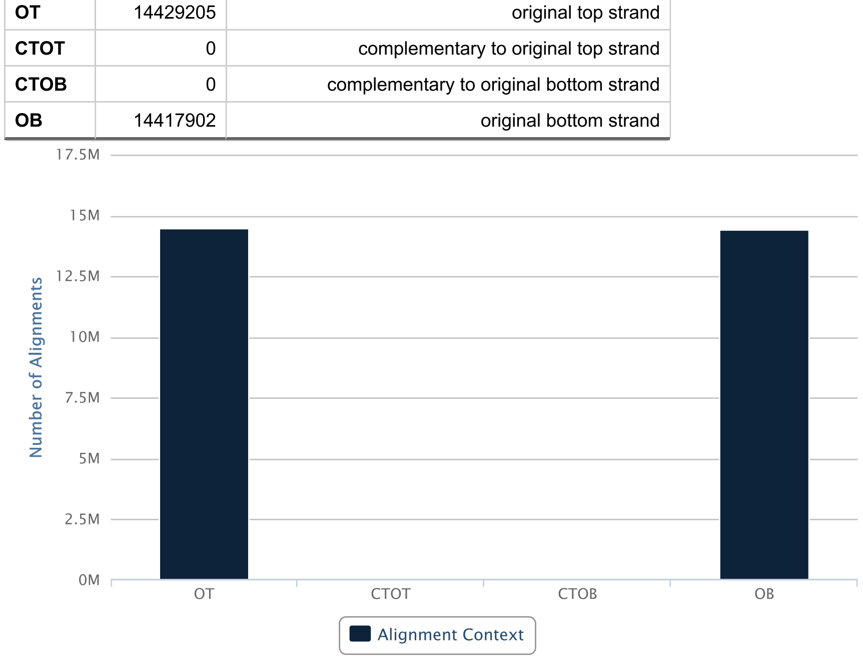

Deduplication

**Alignments analysed**	28847107
**Unique alignments**	25686906
**Duplicates removed**	3160201
Duplicated alignments were found at **2709696** different positions	

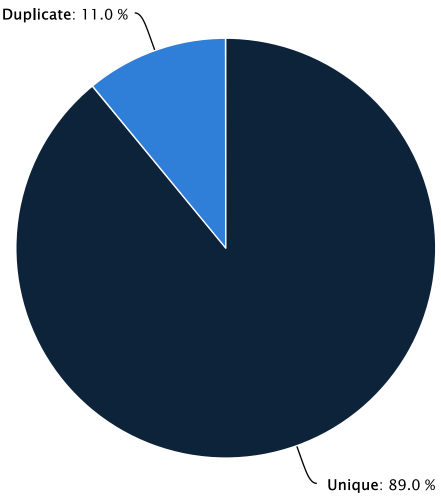

Cytosine Methylation after Extraction

**Total C's analysed**	910766854
**Methylated C's in CpG context**	42558319
**Methylated C's in CHG context**	1474917
**Methylated C's in CHH context**	3202408
**Unmethylated C's in CpG context**	16169355
**Unmethylated C's in CHG context**	217018089
**Unmethylated C's in CHH context**	630343766
**Percentage methylation (CpG context)**	72.5%
**Percentage methylation (CHG context)**	0.7%
**Percentage methylation (CHH context)**	0.5%

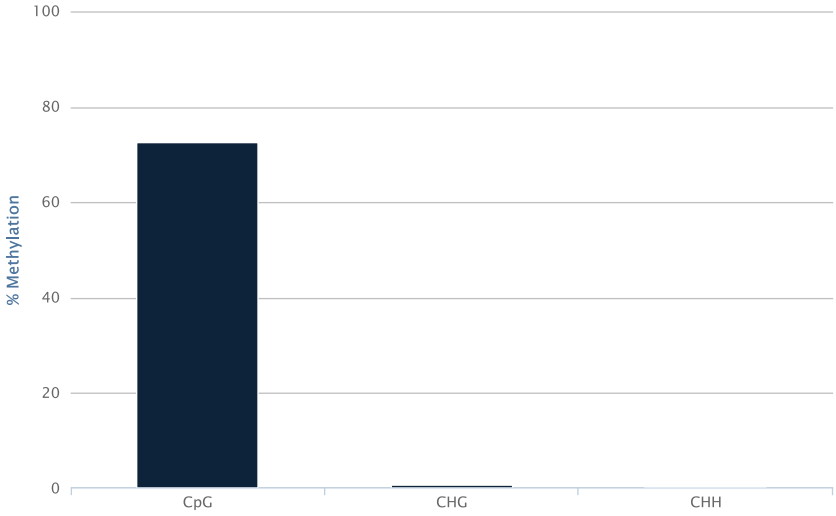

Nucleotide Coverage

Nucleotide Class	Counts Sample	Counts Genome	% in Sample	% in Genome
**A**	1977425257	769902373	28.49	29.06
**T**	1822194661	771539118	26.25	29.12
**C**	1460110526	553941589	21.03	20.91
**G**	1682115545	554298949	24.23	20.92
**AC**	351046418	134825334	5.10	5.09
**CA**	512281864	194999195	7.44	7.36
**TC**	398355573	168302551	5.78	6.35

Nucleotide Class	Counts Sample	Counts Genome	% in Sample	% in Genome
**CT**	462923295	189605870	6.72	7.16
**CC**	370854477	141780183	5.38	5.35
**CG**	101518074	27540367	1.47	1.04
**GC**	329793882	109014748	4.79	4.11
**GG**	477526092	141809778	6.93	5.35
**AG**	547191295	189448103	7.94	7.15
**GA**	489399926	168131316	7.10	6.35
**TG**	542592128	195484730	7.88	7.38
**GT**	367678522	135324159	5.34	5.11
**TT**	513541121	248110054	7.45	9.36
**TA**	355281336	159621424	5.16	6.02
**AT**	467274709	198476252	6.78	7.49
**AA**	602372322	247130183	8.74	9.33

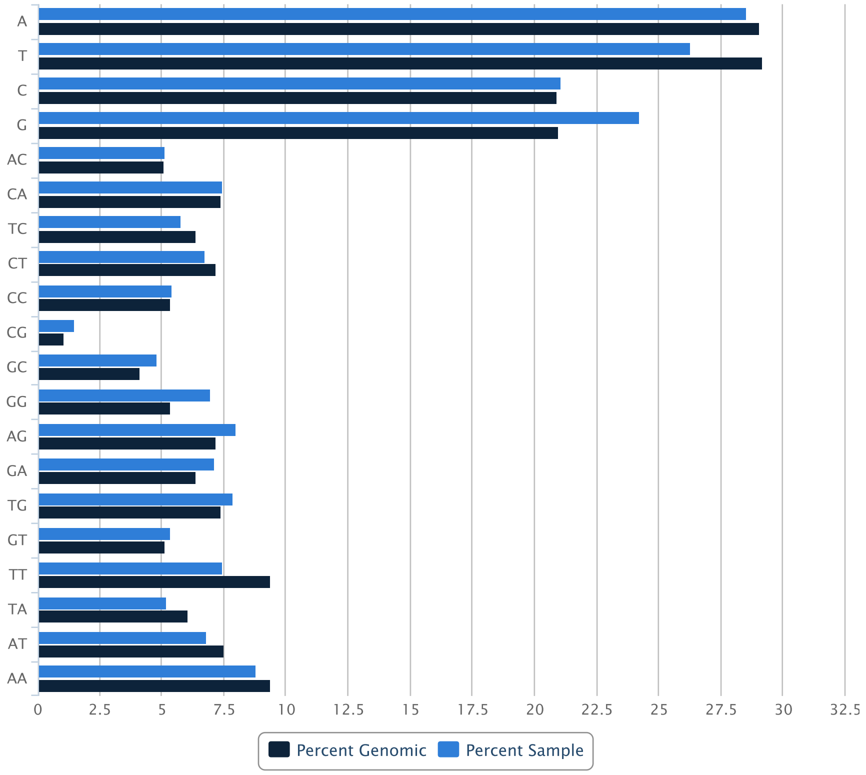

M-Bias Plot

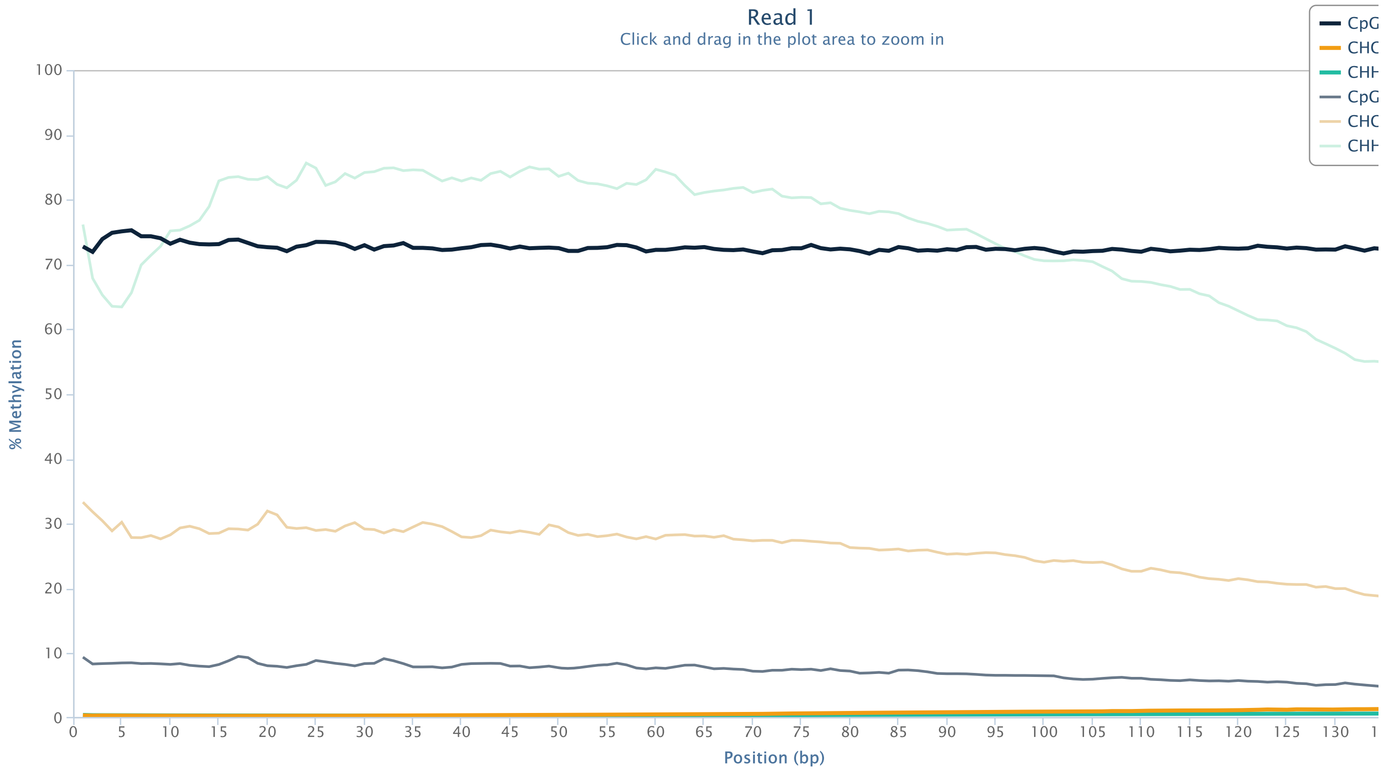

Read 1
Click and drag in the plot area to zoom in
% Methylation
Position (bp)
CpG
CHG
CHH
CpG
CHG
CHH

Bismark Processing Report

trimgalore/Merged/C8_TGACCA_R1_merged_val_1.fq.gz and trimgalore/Merged/C8_TGACCA_R2_merged_val_2.fq.gz

Data processed at 16:37 on 2016-05-01

Alignment

**Sequence pairs analysed in total**	62222813
**Paired-end alignments with a unique best hit**	40776427
**Pairs without alignments under any condition**	11458624
**Pairs that did not map uniquely**	9987762
**Genomic sequence context not extractable (edges of chromosomes)**	224

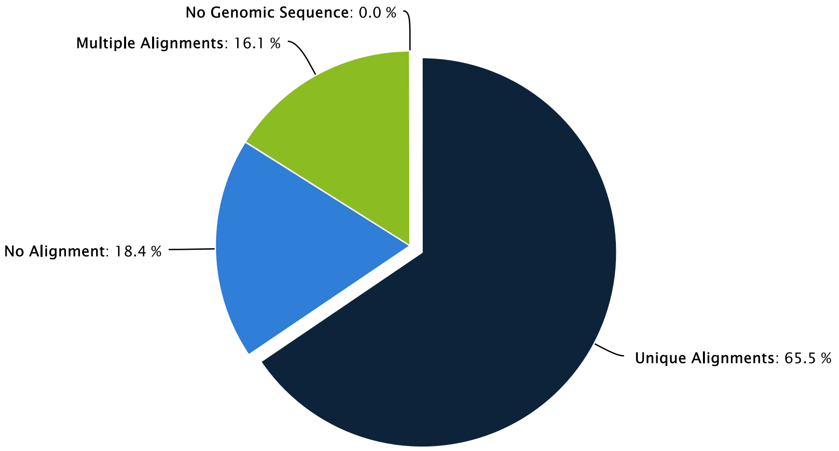

Cytosine Methylation

**Total C's analysed**	2122804760
**Methylated C's in CpG context**	99074471
**Methylated C's in CHG context**	8637897
**Methylated C's in CHH context**	13401530
**Methylated C's in Unknown context**	9457
**Unmethylated C's in CpG context**	44087053
**Unmethylated C's in CHG context**	515315890
**Unmethylated C's in CHH context**	1442287919
**Unmethylated C's in Unknown context**	26742
**Percentage methylation (CpG context)**	69.2%
**Percentage methylation (CHG context)**	1.6%
**Percentage methylation (CHH context)**	0.9%

**Methylated C's in Unknown context**	N/A%

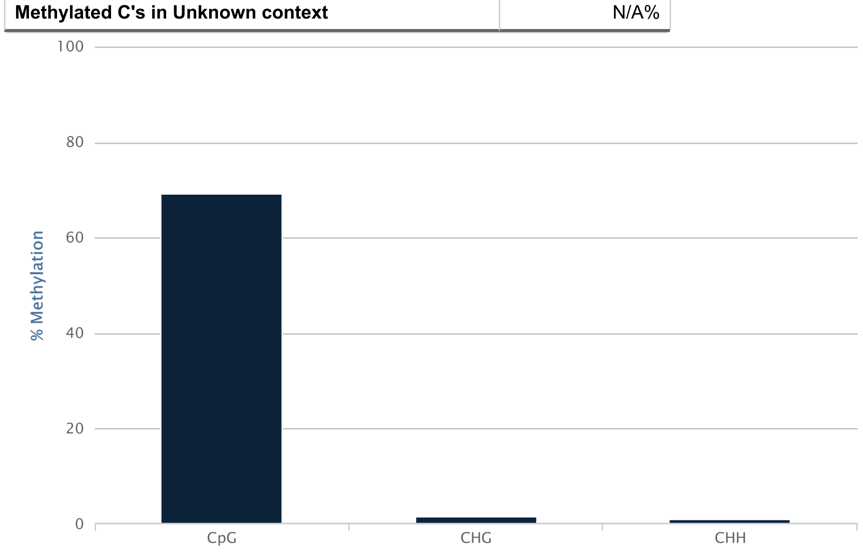

Alignment to Individual Bisulfite Strands

**OT**	20391252	original top strand
**CTOT**	0	complementary to original top strand
**CTOB**	0	complementary to original bottom strand
**OB**	20384951	original bottom strand

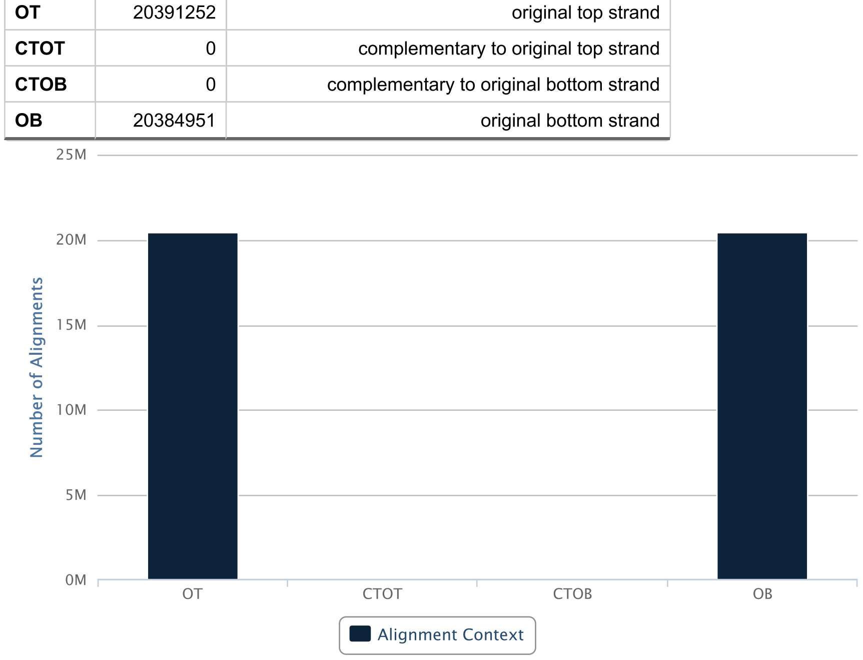

Deduplication

**Alignments analysed**	40776203
**Unique alignments**	36989206
**Duplicates removed**	3786997

Duplicated alignments were found at **3217390** different positions

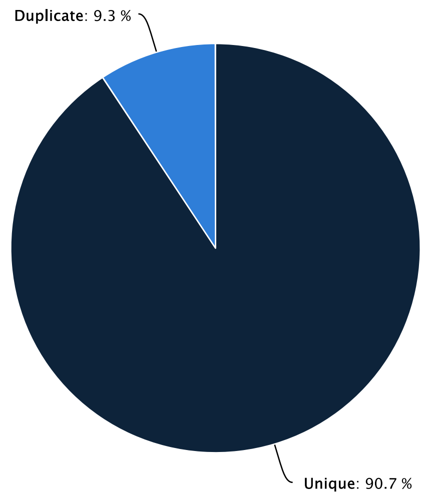

Cytosine Methylation after Extraction

**Total C's analysed**	1120993132
**Methylated C's in CpG context**	48238108
**Methylated C's in CHG context**	2621535
**Methylated C's in CHH context**	5246108
**Unmethylated C's in CpG context**	21648657
**Unmethylated C's in CHG context**	266773679
**Unmethylated C's in CHH context**	776465045
**Percentage methylation (CpG context)**	69.0%
**Percentage methylation (CHG context)**	1.0%
**Percentage methylation (CHH context)**	0.7%

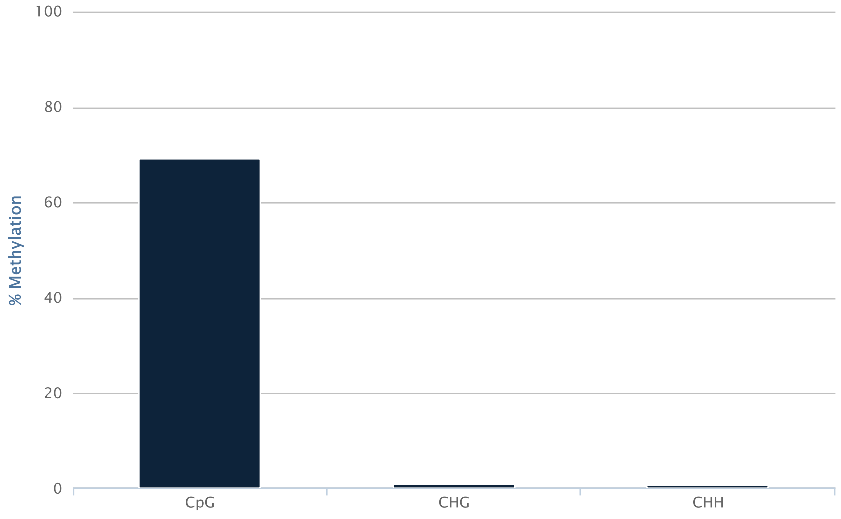

Nucleotide Coverage

Nucleotide Class	Counts Sample	Counts Genome	% in Sample	% in Genome
**A**	2581225340	769902373	28.25	29.06
**T**	2406049377	771539118	26.33	29.12
**C**	1931026458	553941589	21.13	20.91
**G**	2219722659	554298949	24.29	20.92
**AC**	459189301	134825334	5.07	5.09
**CA**	671911439	194999195	7.41	7.36
**TC**	527608062	168302551	5.82	6.35

Nucleotide Class	Counts Sample	Counts Genome	% in Sample	% in Genome
**CT**	615364033	189605870	6.79	7.16
**CC**	496183668	141780183	5.47	5.35
**CG**	130021467	27540367	1.43	1.04
**GC**	432539228	109014748	4.77	4.11
**GG**	635055965	141809778	7.01	5.35
**AG**	719892490	189448103	7.94	7.15
**GA**	641669165	168131316	7.08	6.35
**TG**	717218090	195484730	7.91	7.38
**GT**	484651706	135324159	5.35	5.11
**TT**	680738917	248110054	7.51	9.36
**TA**	463521078	159621424	5.11	6.02
**AT**	608978758	198476252	6.72	7.49
**AA**	778926009	247130183	8.59	9.33

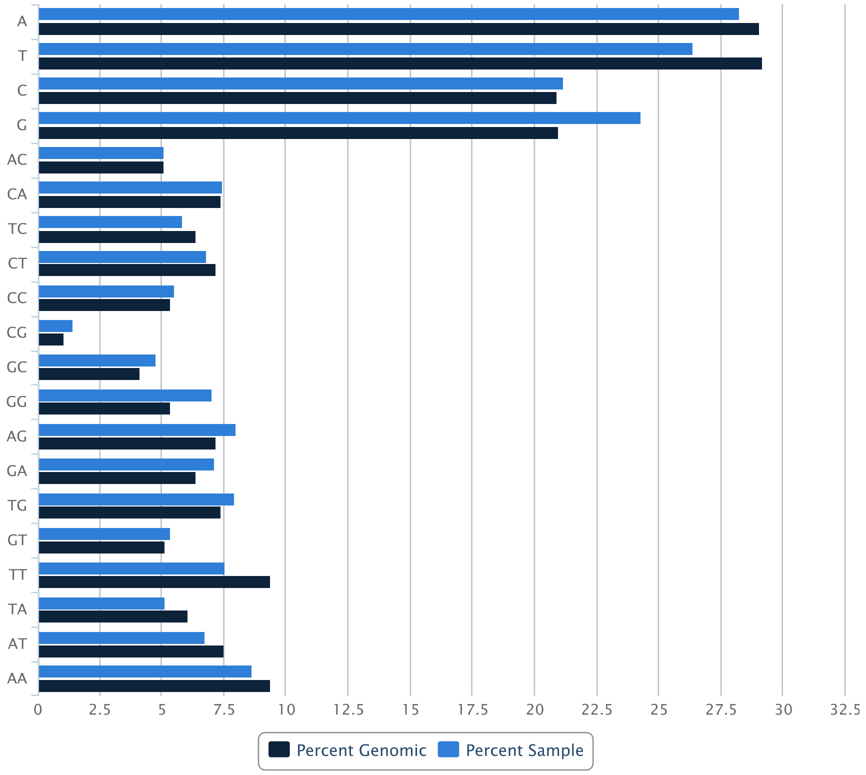

M-Bias Plot

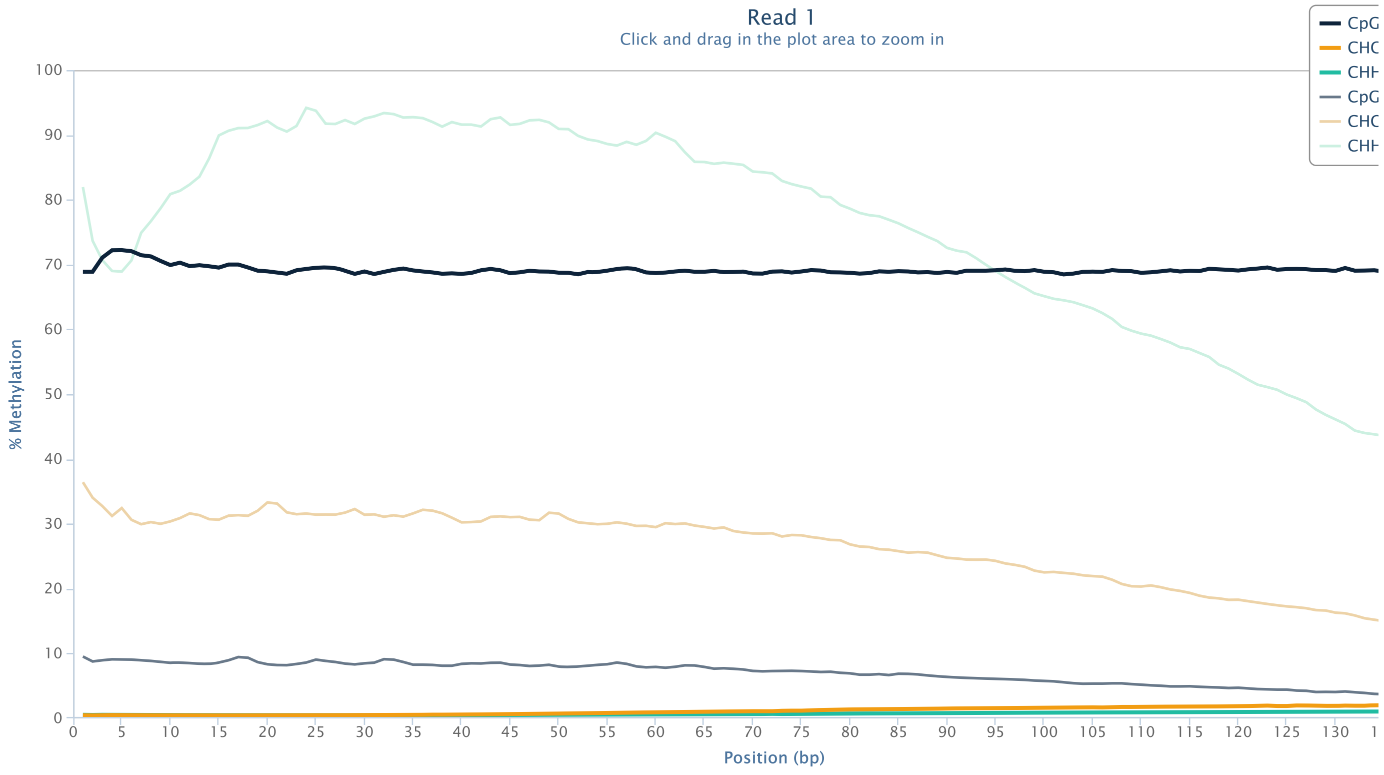

Read 1
Click and drag in the plot area to zoom in
% Methylation
Position (bp)
CpG
CHG
CHH
CpG
CHG
CHH

Bismark Processing Report

trimgalore/Merged/C11_CAGATC_R1_merged_val_1.fq.gz and trimgalore/Merged/C11_CAGATC_R2_merged_val_2.fq.gz

Data processed at 16:37 on 2016-05-01

Alignment

**Sequence pairs analysed in total**	56334011
**Paired-end alignments with a unique best hit**	36540606
**Pairs without alignments under any condition**	11523560
**Pairs that did not map uniquely**	8269845
**Genomic sequence context not extractable (edges of chromosomes)**	245

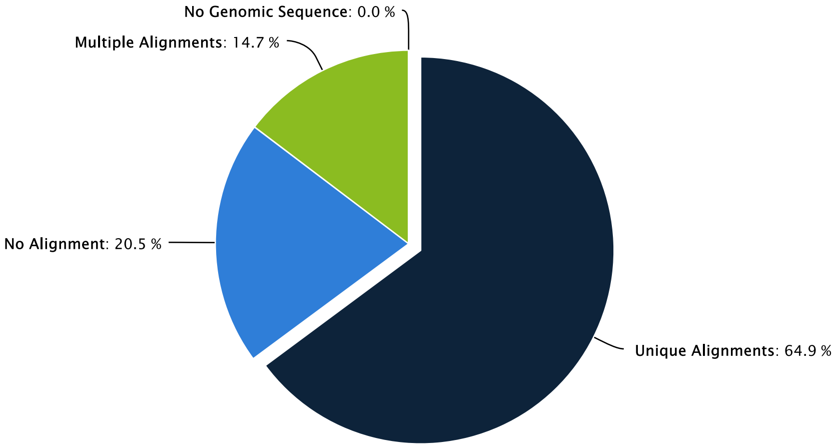

Cytosine Methylation

**Total C's analysed**	1986724101
**Methylated C's in CpG context**	98784072
**Methylated C's in CHG context**	7389841
**Methylated C's in CHH context**	11371764
**Methylated C's in Unknown context**	7632
**Unmethylated C's in CpG context**	37561804
**Unmethylated C's in CHG context**	484093331
**Unmethylated C's in CHH context**	1347523289
**Unmethylated C's in Unknown context**	22892
**Percentage methylation (CpG context)**	72.5%
**Percentage methylation (CHG context)**	1.5%
**Percentage methylation (CHH context)**	0.8%

**Methylated C's in Unknown context**	N/A%

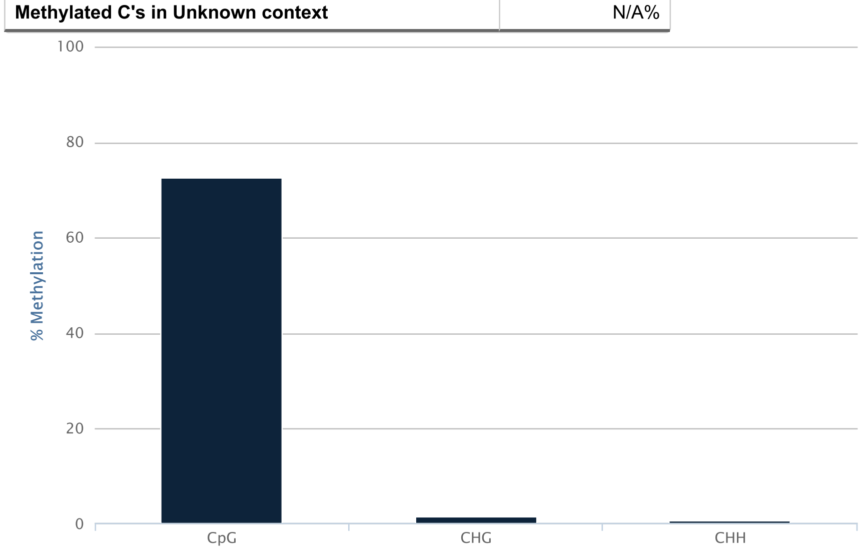

Alignment to Individual Bisulfite Strands

**OT**	18275324	original top strand
**CTOT**	0	complementary to original top strand
**CTOB**	0	complementary to original bottom strand
**OB**	18265037	original bottom strand

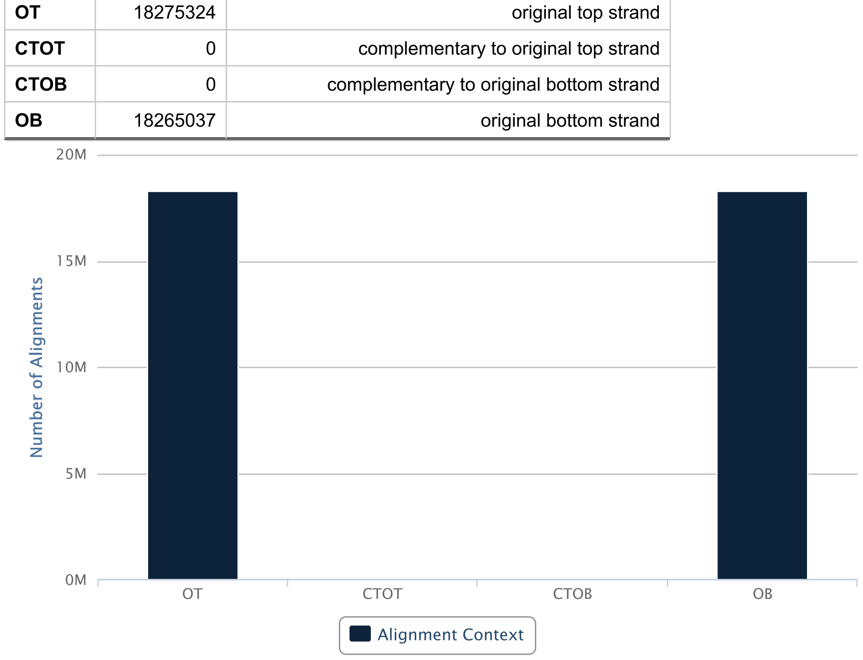

Deduplication

**Alignments analysed**	36540361
**Unique alignments**	33163089
**Duplicates removed**	3377272
Duplicated alignments were found at **2871698** different positions	

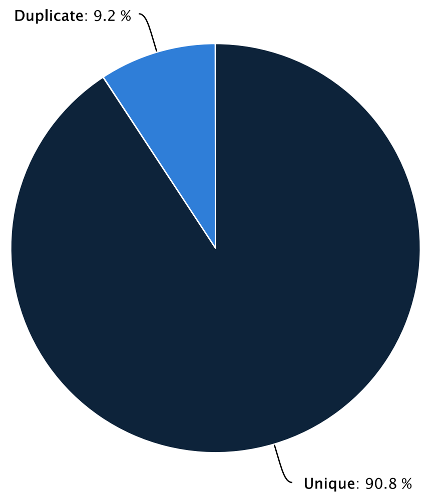

Cytosine Methylation after Extraction

**Total C's analysed**	1092948098
**Methylated C's in CpG context**	50253422
**Methylated C's in CHG context**	2097185
**Methylated C's in CHH context**	4370326
**Unmethylated C's in CpG context**	19249485
**Unmethylated C's in CHG context**	261686139
**Unmethylated C's in CHH context**	755291541
**Percentage methylation (CpG context)**	72.3%
**Percentage methylation (CHG context)**	0.8%
**Percentage methylation (CHH context)**	0.6%

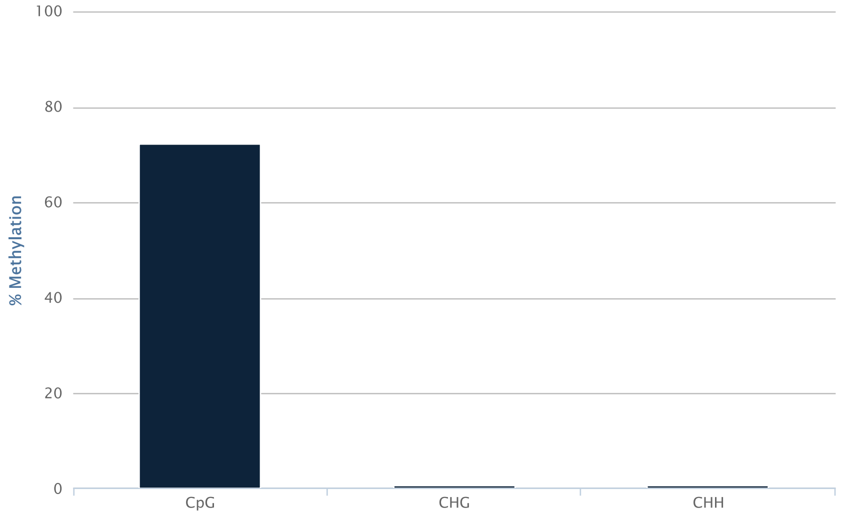

Nucleotide Coverage

Nucleotide Class	Counts Sample	Counts Genome	% in Sample	% in Genome
**A**	2433553932	769902373	28.46	29.06
**T**	2236380448	771539118	26.15	29.12
**C**	1797707723	553941589	21.02	20.91
**G**	2083943648	554298949	24.37	20.92
**AC**	431957802	134825334	5.09	5.09
**CA**	631046642	194999195	7.44	7.36
**TC**	488219723	168302551	5.75	6.35

Nucleotide Class	Counts Sample	Counts Genome	% in Sample	% in Genome
**CT**	569498163	189605870	6.71	7.16
**CC**	458031221	141780183	5.40	5.35
**CG**	123230668	27540367	1.45	1.04
**GC**	406176775	109014748	4.79	4.11
**GG**	595050384	141809778	7.01	5.35
**AG**	677867532	189448103	7.99	7.15
**GA**	606427371	168131316	7.15	6.35
**TG**	671561197	195484730	7.91	7.38
**GT**	453925290	135324159	5.35	5.11
**TT**	626950044	248110054	7.39	9.36
**TA**	434083920	159621424	5.12	6.02
**AT**	571793190	198476252	6.74	7.49
**AA**	739381055	247130183	8.71	9.33

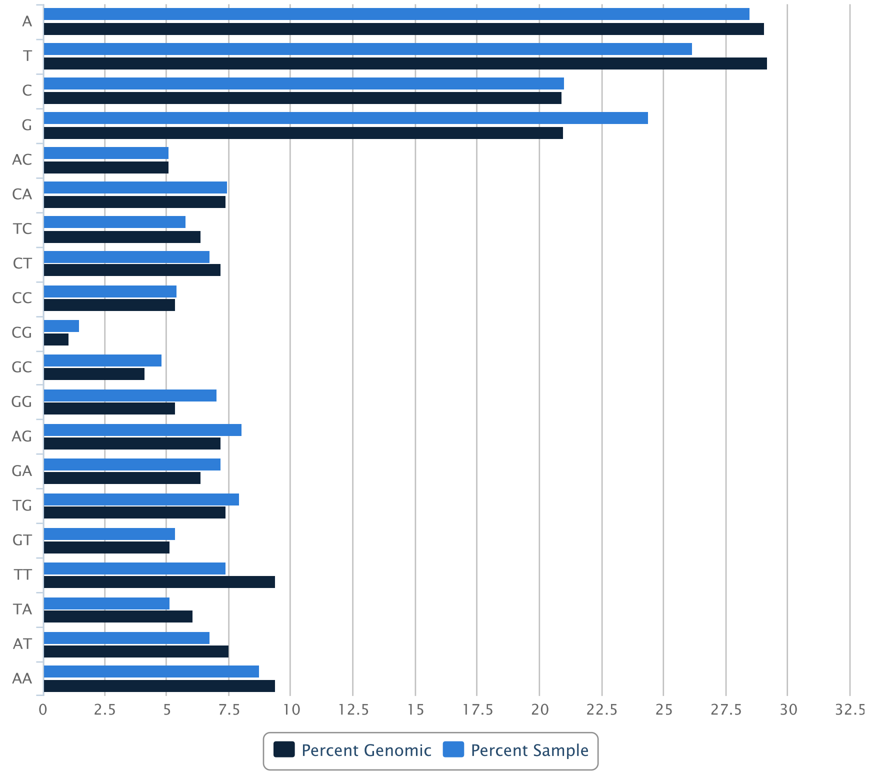

M-Bias Plot

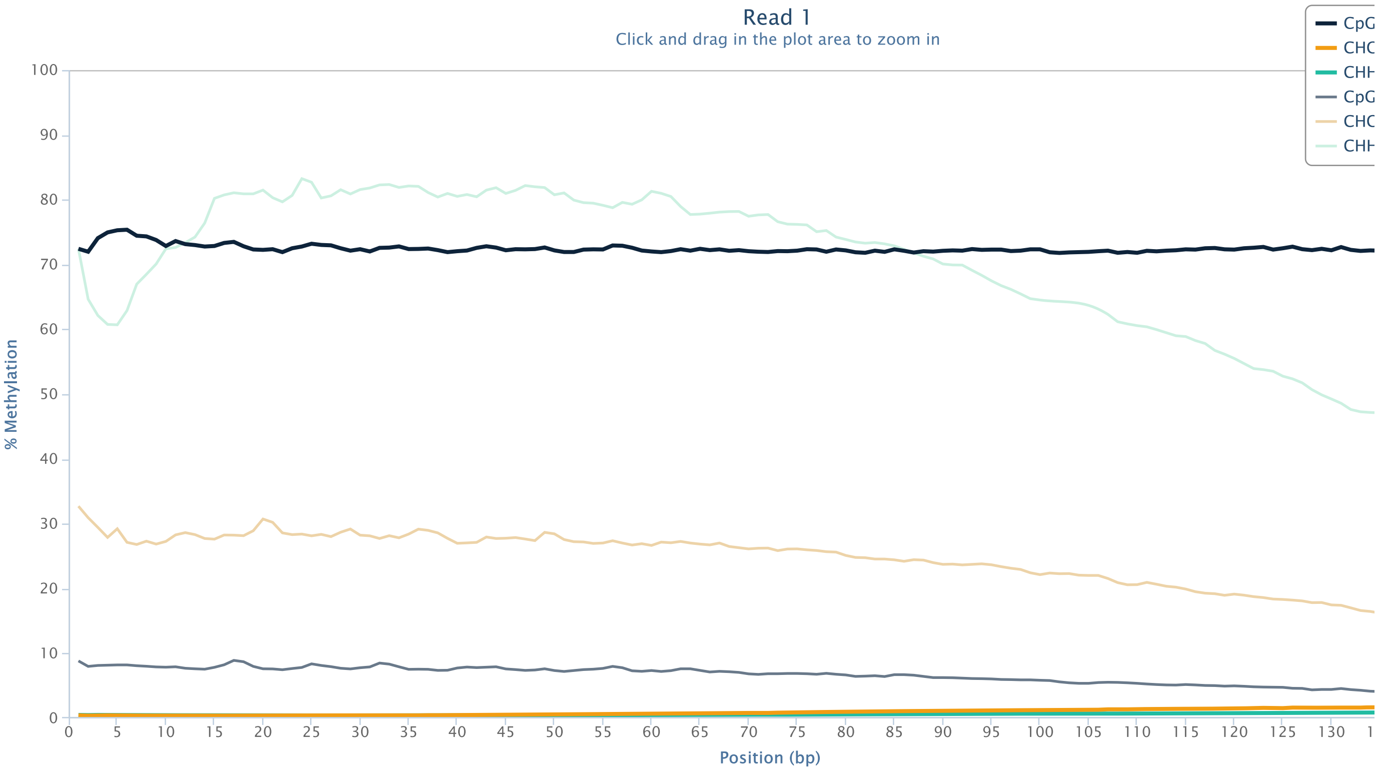

Analysis produced by **Bismark** (version v0.15.0) - a tool to map bisulfite converted sequence reads and determine cytosine methylation states

Report graphs rendered using jQuery and Highcharts. Page design by Phil Ewels.

Babraham Bioinformatics

Bismark Processing Report

trimgalore/Merged/C12_TAGCTT_R1_merged_val_1.fq.gz and trimgalore/Merged/C12_TAGCTT_R2_merged_val_2.fq.gz

Data processed at 16:37 on 2016-05-01

Alignment

**Sequence pairs analysed in total**	46411355
**Paired-end alignments with a unique best hit**	29521538
**Pairs without alignments under any condition**	10606636
**Pairs that did not map uniquely**	6283181
**Genomic sequence context not extractable (edges of chromosomes)**	244

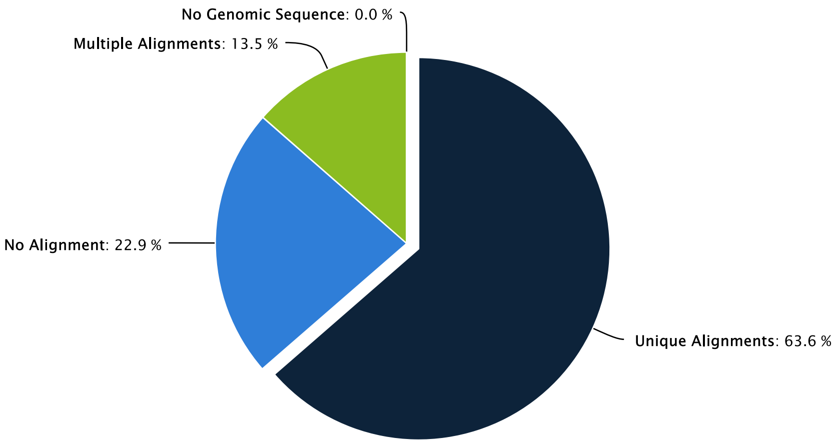

Cytosine Methylation

**Total C's analysed**	1683498024
**Methylated C's in CpG context**	82187635
**Methylated C's in CHG context**	5833231
**Methylated C's in CHH context**	9620619
**Methylated C's in Unknown context**	6305
**Unmethylated C's in CpG context**	31648470
**Unmethylated C's in CHG context**	404836787
**Unmethylated C's in CHH context**	1149371282
**Unmethylated C's in Unknown context**	19688
**Percentage methylation (CpG context)**	72.2%
**Percentage methylation (CHG context)**	1.4%
**Percentage methylation (CHH context)**	0.8%

**Methylated C's in Unknown context**	N/A%

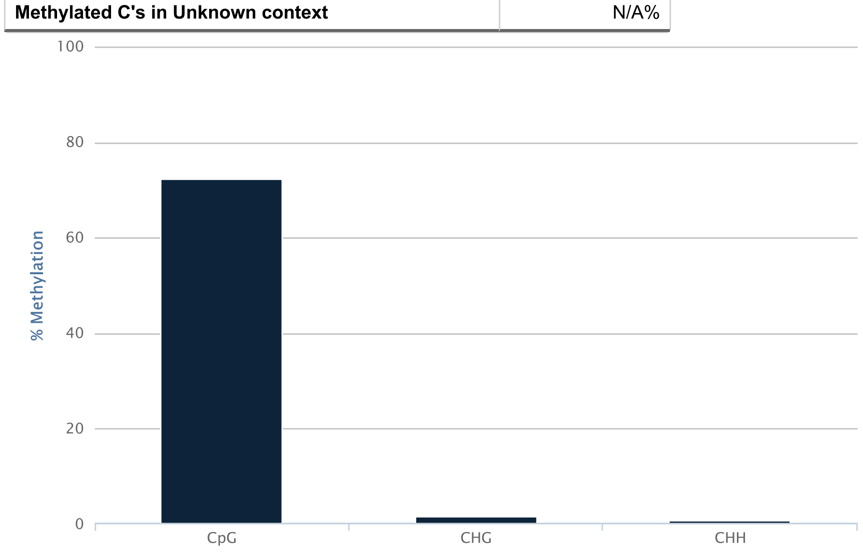

Alignment to Individual Bisulfite Strands

**OT**	14765306	original top strand
**CTOT**	0	complementary to original top strand
**CTOB**	0	complementary to original bottom strand
**OB**	14755988	original bottom strand

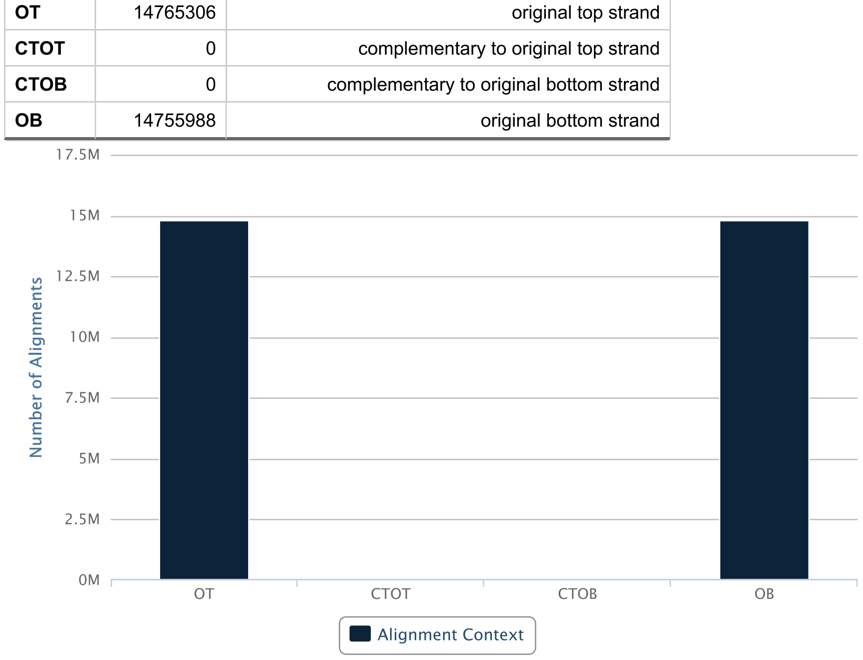

Deduplication

**Alignments analysed**	29521294
**Unique alignments**	25525904
**Duplicates removed**	3995390
Duplicated alignments were found at **3362708** different positions	

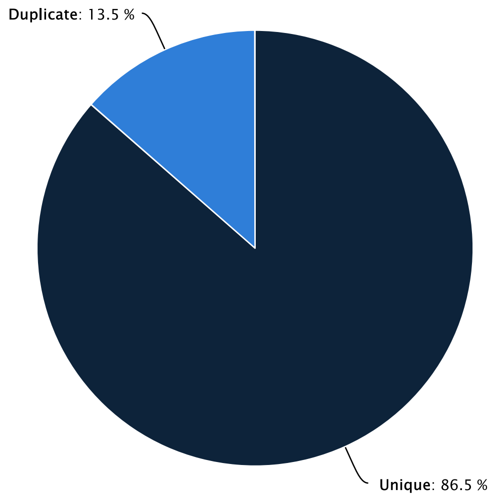

Cytosine Methylation after Extraction

**Total C's analysed**	944637258
**Methylated C's in CpG context**	43011859
**Methylated C's in CHG context**	1502647
**Methylated C's in CHH context**	3563463
**Unmethylated C's in CpG context**	16818202
**Unmethylated C's in CHG context**	224046958
**Unmethylated C's in CHH context**	655694129
**Percentage methylation (CpG context)**	71.9%
**Percentage methylation (CHG context)**	0.7%
**Percentage methylation (CHH context)**	0.5%

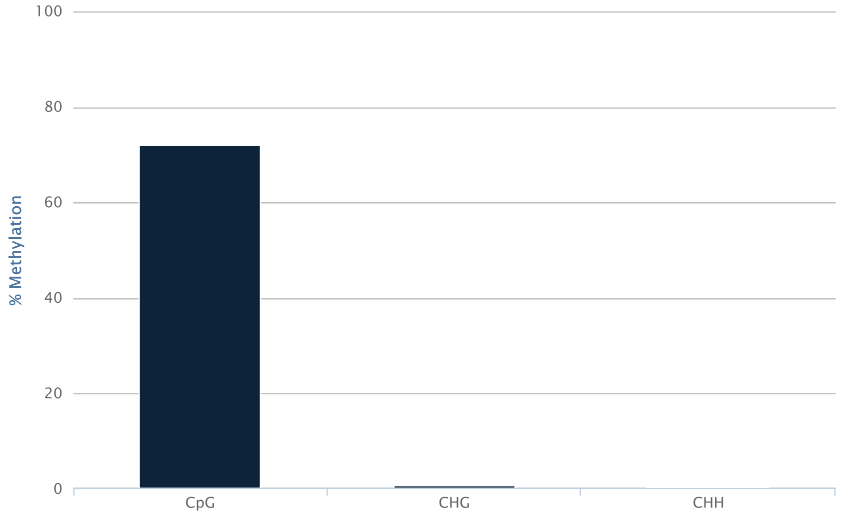

Nucleotide Coverage

Nucleotide Class	Counts Sample	Counts Genome	% in Sample	% in Genome
**A**	2064208950	769902373	28.69	29.06
**T**	1898142654	771539118	26.38	29.12
**C**	1508081468	553941589	20.96	20.91
**G**	1725648223	554298949	23.98	20.92
**AC**	365880075	134825334	5.12	5.09
**CA**	532064517	194999195	7.45	7.36
**TC**	413676332	168302551	5.79	6.35

Nucleotide Class	Counts Sample	Counts Genome	% in Sample	% in Genome
**CT**	479590936	189605870	6.71	7.16
**CC**	381588768	141780183	5.34	5.35
**CG**	101942167	27540367	1.43	1.04
**GC**	336853392	109014748	4.72	4.11
**GG**	485012377	141809778	6.79	5.35
**AG**	564798112	189448103	7.91	7.15
**GA**	506726204	168131316	7.09	6.35
**TG**	560449988	195484730	7.85	7.38
**GT**	379795121	135324159	5.32	5.11
**TT**	537544662	248110054	7.53	9.36
**TA**	373430318	159621424	5.23	6.02
**AT**	490360320	198476252	6.86	7.49
**AA**	633350662	247130183	8.87	9.33

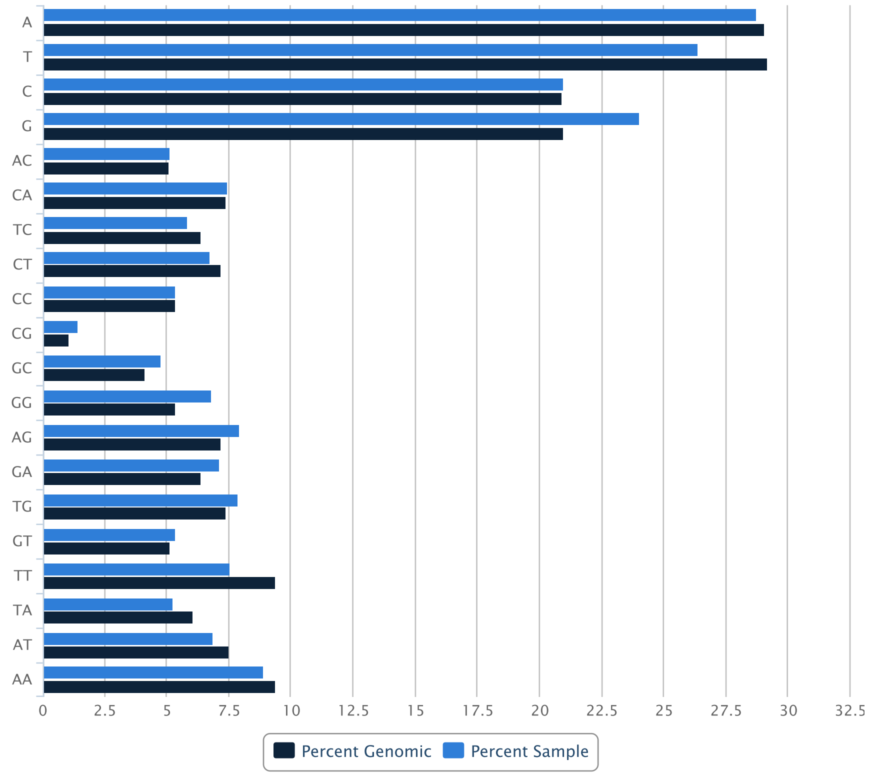

M-Bias Plot

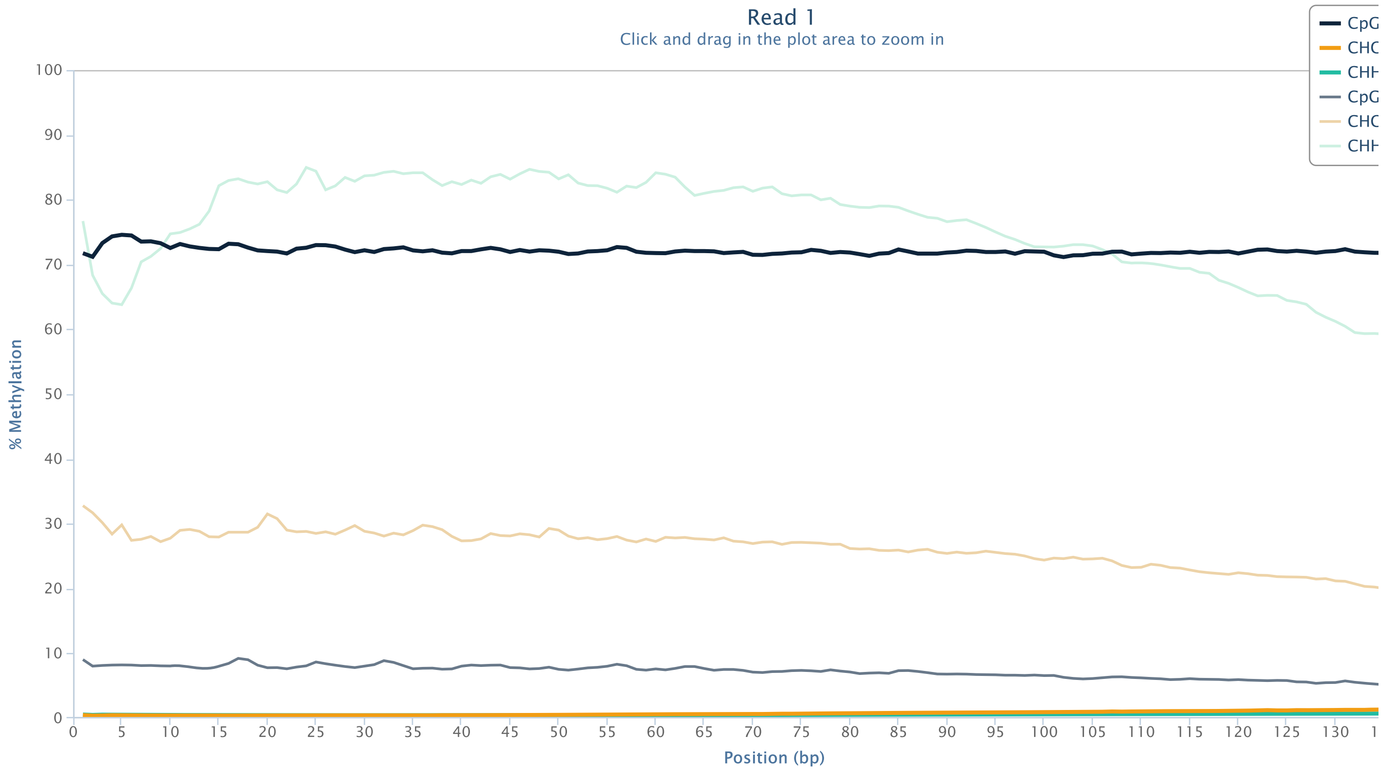

Analysis produced by **Bismark** (version v0.15.0) - a tool to map bisulfite converted sequence reads and determine cytosine methylation states

Report graphs rendered using jQuery and Highcharts. Page design by Phil Ewels.

Bismark Processing Report

trimgalore/Merged/M1_CGATGT_R1_merged_val_1.fq.gz and trimgalore/Merged/M1_CGATGT_R2_merged_val_2.fq.gz

Data processed at 16:37 on 2016-05-01

Alignment

**Sequence pairs analysed in total**	53408360
**Paired-end alignments with a unique best hit**	36461749
**Pairs without alignments under any condition**	9378541
**Pairs that did not map uniquely**	7568070
**Genomic sequence context not extractable (edges of chromosomes)**	219

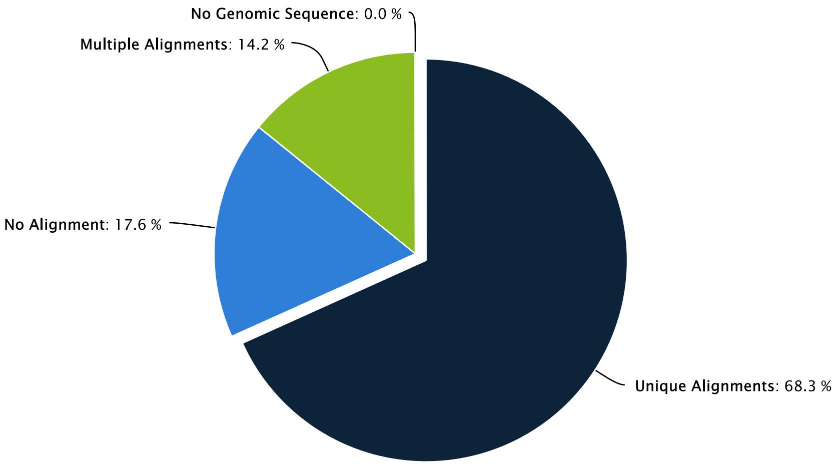

Cytosine Methylation

**Total C's analysed**	2049740866
**Methylated C's in CpG context**	96053495
**Methylated C's in CHG context**	6505783
**Methylated C's in CHH context**	9539177
**Methylated C's in Unknown context**	6279
**Unmethylated C's in CpG context**	36658062
**Unmethylated C's in CHG context**	488056413
**Unmethylated C's in CHH context**	1412927936
**Unmethylated C's in Unknown context**	21173
**Percentage methylation (CpG context)**	72.4%
**Percentage methylation (CHG context)**	1.3%
**Percentage methylation (CHH context)**	0.7%

**Methylated C's in Unknown context**	N/A%

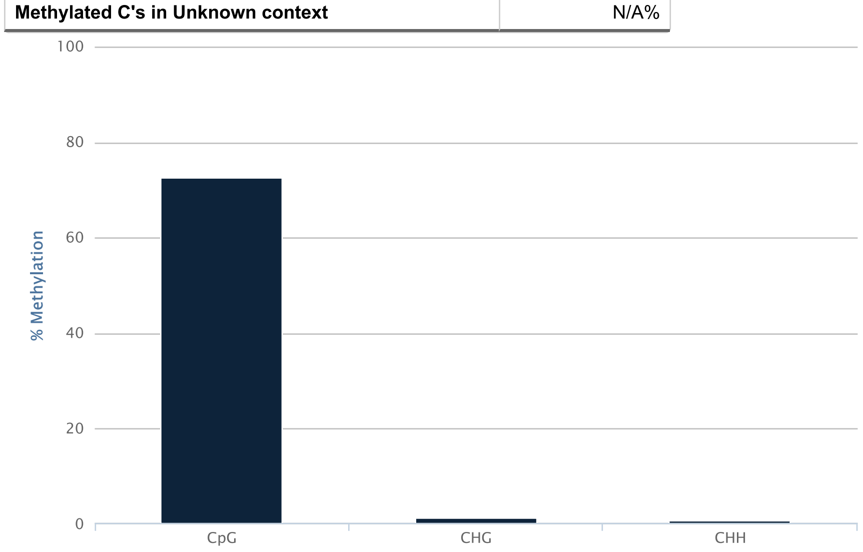

Alignment to Individual Bisulfite Strands

**OT**	18230625	original top strand
**CTOT**	0	complementary to original top strand
**CTOB**	0	complementary to original bottom strand
**OB**	18230905	original bottom strand

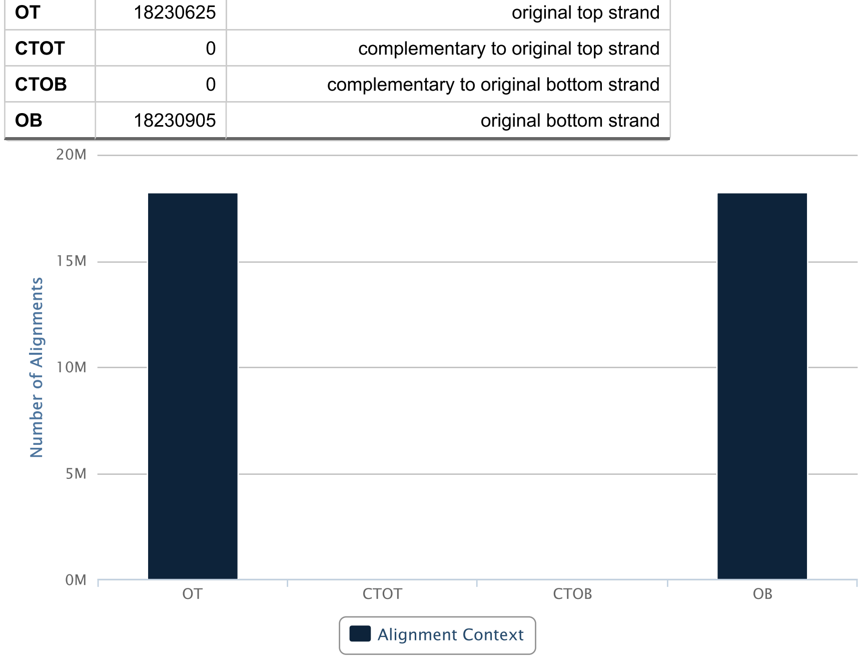

Deduplication

**Alignments analysed**	36461530
**Unique alignments**	34062917
**Duplicates removed**	2398613

Duplicated alignments were found at **2071534** different positions

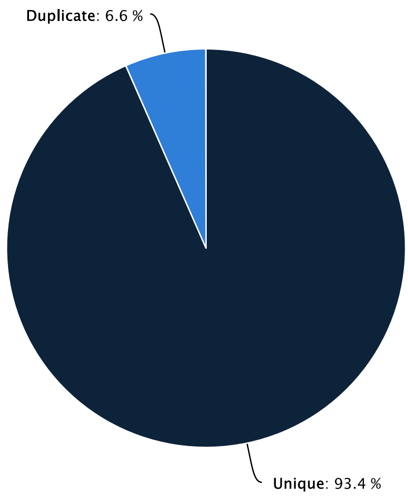

Cytosine Methylation after Extraction

**Total C's analysed**	1199910660
**Methylated C's in CpG context**	52083959
**Methylated C's in CHG context**	1725441
**Methylated C's in CHH context**	3460670
**Unmethylated C's in CpG context**	20014821
**Unmethylated C's in CHG context**	280535325
**Unmethylated C's in CHH context**	842090444
**Percentage methylation (CpG context)**	72.2%
**Percentage methylation (CHG context)**	0.6%
**Percentage methylation (CHH context)**	0.4%

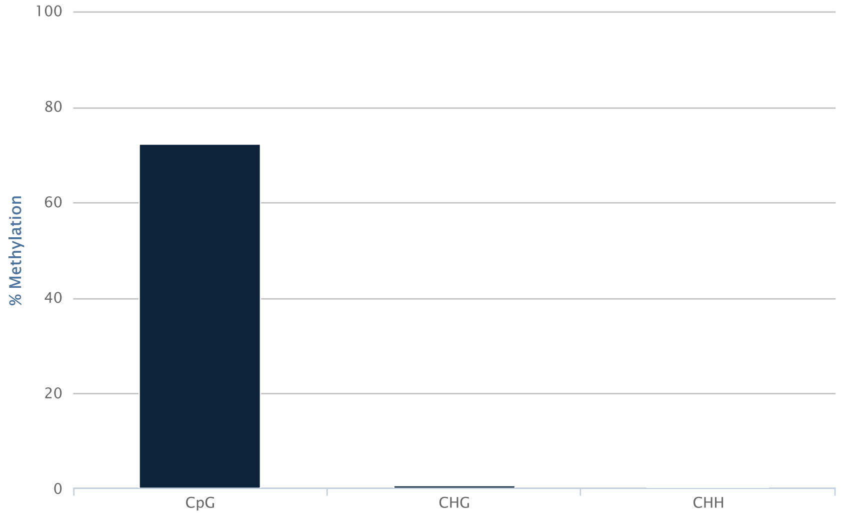

Nucleotide Coverage

Nucleotide Class	Counts Sample	Counts Genome	% in Sample	% in Genome
**A**	2460804005	769902373	28.34	29.06
**T**	2329278927	771539118	26.83	29.12
**C**	1843146139	553941589	21.23	20.91
**G**	2048906160	554298949	23.60	20.92
**AC**	439299123	134825334	5.10	5.09
**CA**	641359560	194999195	7.44	7.36
**TC**	513014973	168302551	5.95	6.35

Nucleotide Class	Counts Sample	Counts Genome	% in Sample	% in Genome
**CT**	593104397	189605870	6.88	7.16
**CC**	473391140	141780183	5.49	5.35
**CG**	119227592	27540367	1.38	1.04
**GC**	404094997	109014748	4.69	4.11
**GG**	569816466	141809778	6.61	5.35
**AG**	669190155	189448103	7.77	7.15
**GA**	595707041	168131316	6.91	6.35
**TG**	675016878	195484730	7.83	7.38
**GT**	457735466	135324159	5.31	5.11
**TT**	672841896	248110054	7.81	9.36
**TA**	452368276	159621424	5.25	6.02
**AT**	591265498	198476252	6.86	7.49
**AA**	748874453	247130183	8.69	9.33

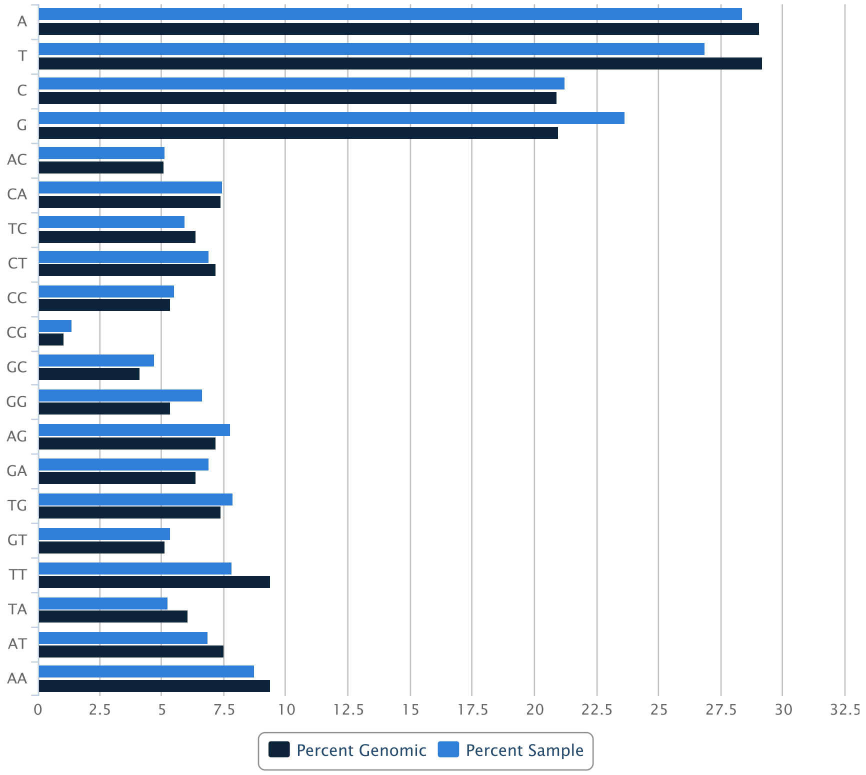

M-Bias Plot

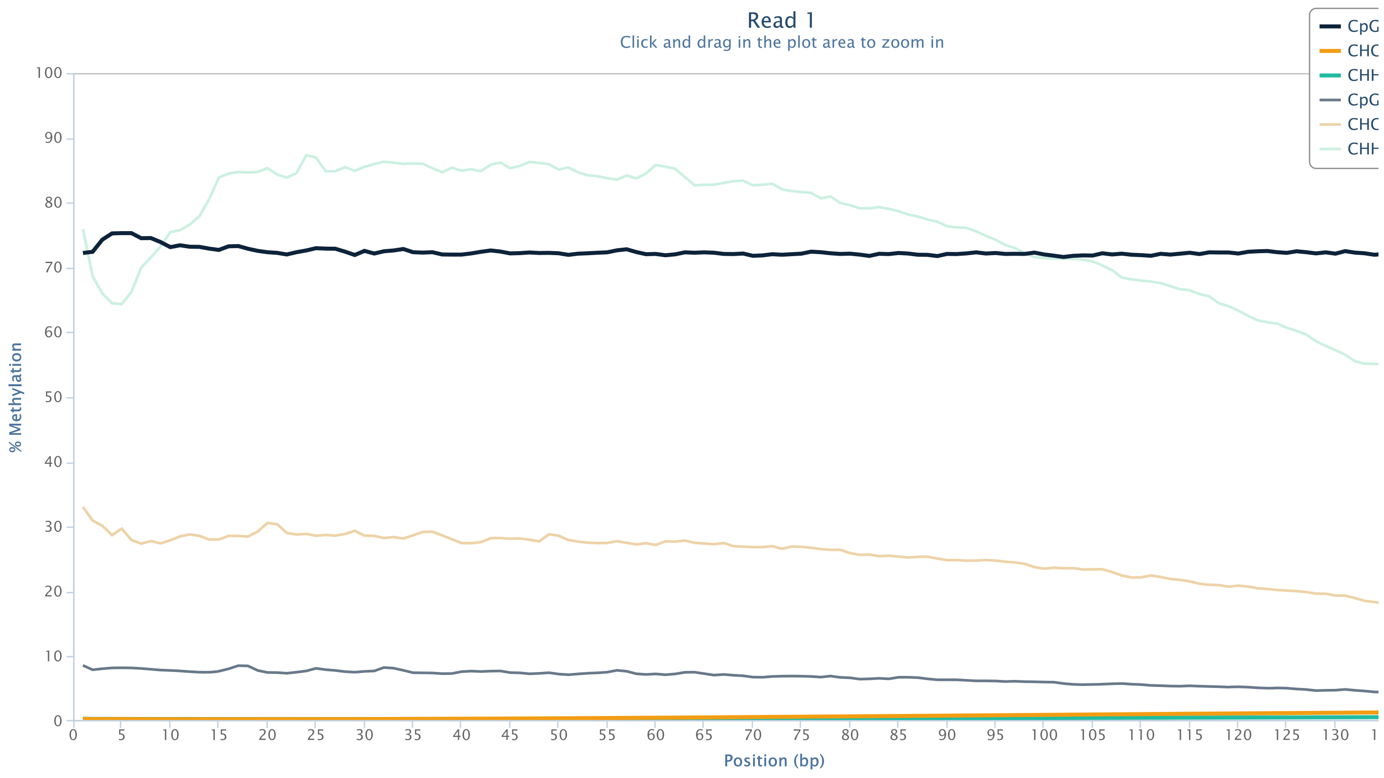

Analysis produced by **Bismark** (version v0.15.0) - a tool to map bisulfite converted sequence reads and determine cytosine methylation states

Report graphs rendered using jQuery and Highcharts. Page design by Phil Ewels.

Babraham Bioinformatics

Bismark Processing Report

trimgalore/Merged/M4_ACAGTG_R1_merged_val_1.fq.gz and
trimgalore/Merged/M4_ACAGTG_R2_merged_val_2.fq.gz

Data processed at 16:37 on 2016-05-01

Alignment

**Sequence pairs analysed in total**	56389378
**Paired-end alignments with a unique best hit**	36201082
**Pairs without alignments under any condition**	11202742
**Pairs that did not map uniquely**	8985554
**Genomic sequence context not extractable (edges of chromosomes)**	296

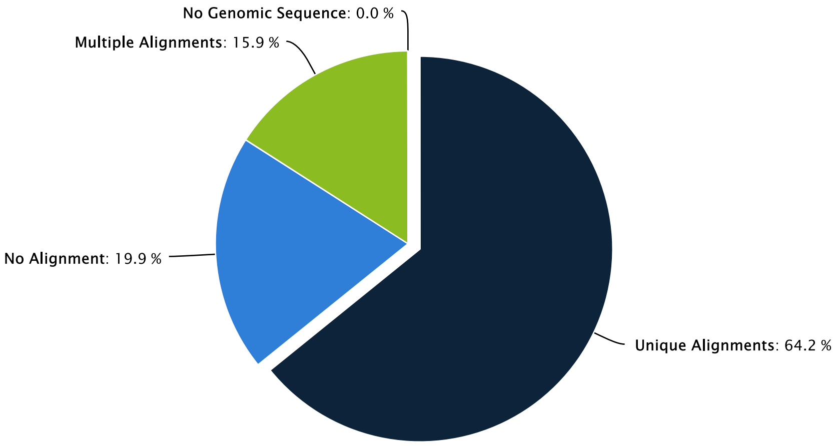

Cytosine Methylation

**Total C's analysed**	1986932553
**Methylated C's in CpG context**	103060816
**Methylated C's in CHG context**	7051010
**Methylated C's in CHH context**	9850420
**Methylated C's in Unknown context**	8788
**Unmethylated C's in CpG context**	37392834
**Unmethylated C's in CHG context**	485110323
**Unmethylated C's in CHH context**	1344467150
**Unmethylated C's in Unknown context**	24266
**Percentage methylation (CpG context)**	73.4%
**Percentage methylation (CHG context)**	1.4%
**Percentage methylation (CHH context)**	0.7%

**Methylated C's in Unknown context**	N/A%

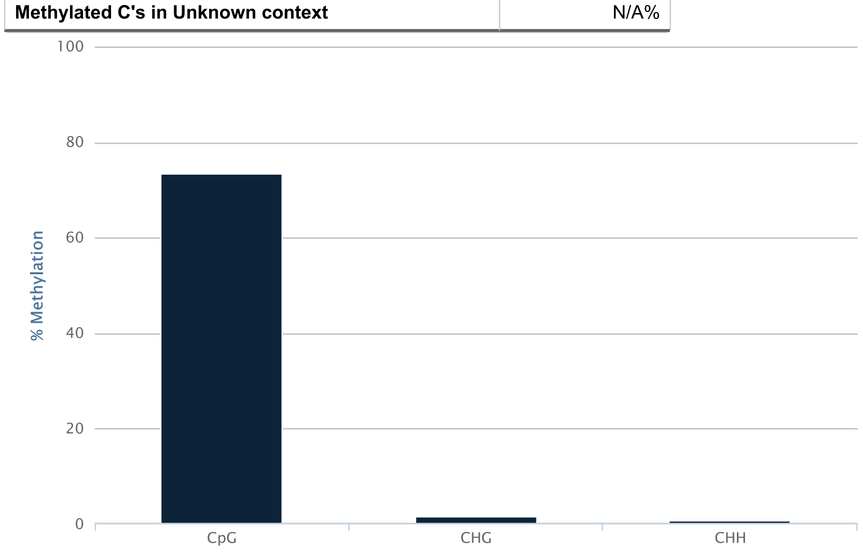

Alignment to Individual Bisulfite Strands

**OT**	18108511	original top strand
**CTOT**	0	complementary to original top strand
**CTOB**	0	complementary to original bottom strand
**OB**	18092275	original bottom strand

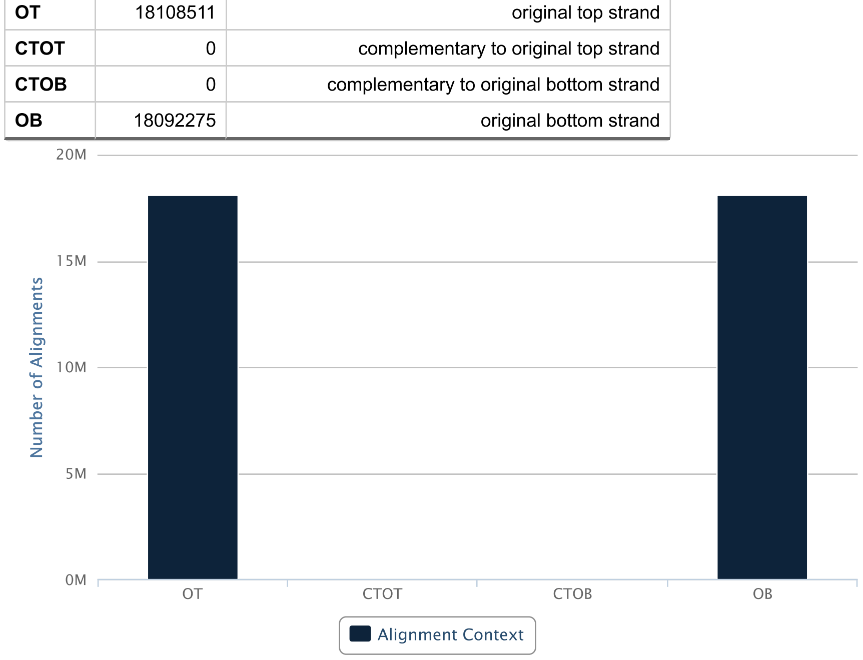

Deduplication

**Alignments analysed**	36200786
**Unique alignments**	31879866
**Duplicates removed**	4320920
Duplicated alignments were found at **3626547** different positions	

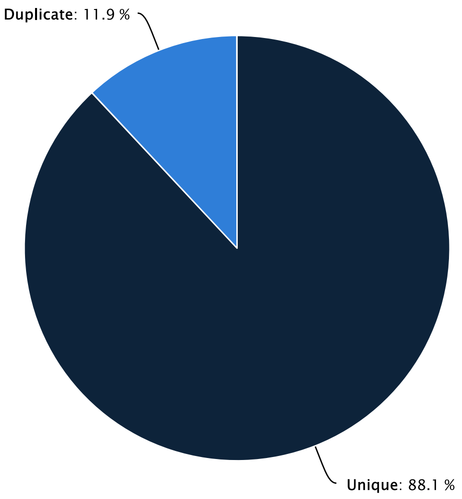

Cytosine Methylation after Extraction

**Total C's analysed**	1073822338
**Methylated C's in CpG context**	51221278
**Methylated C's in CHG context**	1760841
**Methylated C's in CHH context**	3426959
**Unmethylated C's in CpG context**	18779892
**Unmethylated C's in CHG context**	257877277
**Unmethylated C's in CHH context**	740756091
**Percentage methylation (CpG context)**	73.2%
**Percentage methylation (CHG context)**	0.7%
**Percentage methylation (CHH context)**	0.5%

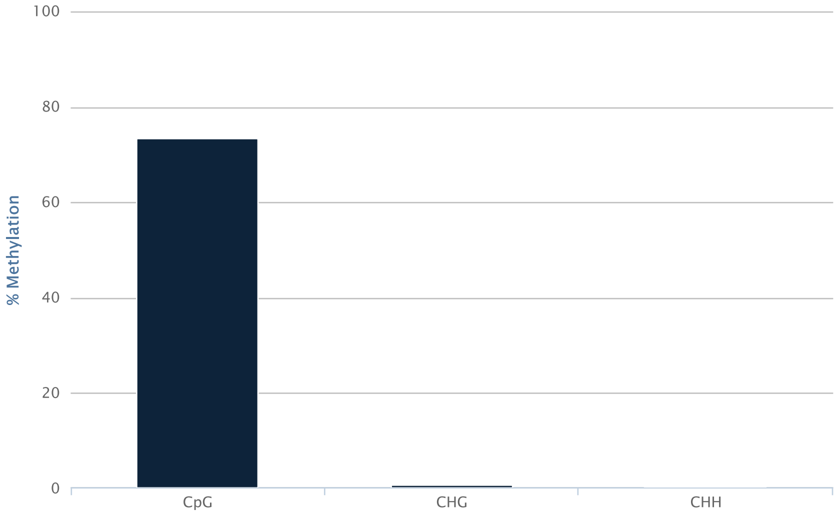

Nucleotide Coverage

Nucleotide Class	Counts Sample	Counts Genome	% in Sample	% in Genome
**A**	2405478286	769902373	28.32	29.06
**T**	2216347314	771539118	26.09	29.12
**C**	1791384352	553941589	21.09	20.91
**G**	2080386892	554298949	24.49	20.92
**AC**	427840168	134825334	5.08	5.09
**CA**	626081114	194999195	7.43	7.36
**TC**	486592035	168302551	5.77	6.35

Nucleotide Class	Counts Sample	Counts Genome	% in Sample	% in Genome
**CT**	566247369	189605870	6.72	7.16
**CC**	456735024	141780183	5.42	5.35
**CG**	126479471	27540367	1.50	1.04
**GC**	407233921	109014748	4.83	4.11
**GG**	595376444	141809778	7.06	5.35
**AG**	673448197	189448103	7.99	7.15
**GA**	603369071	168131316	7.16	6.35
**TG**	668457434	195484730	7.93	7.38
**GT**	452467054	135324159	5.37	5.11
**TT**	619607192	248110054	7.35	9.36
**TA**	426285007	159621424	5.06	6.02
**AT**	564172862	198476252	6.69	7.49
**AA**	727676809	247130183	8.63	9.33

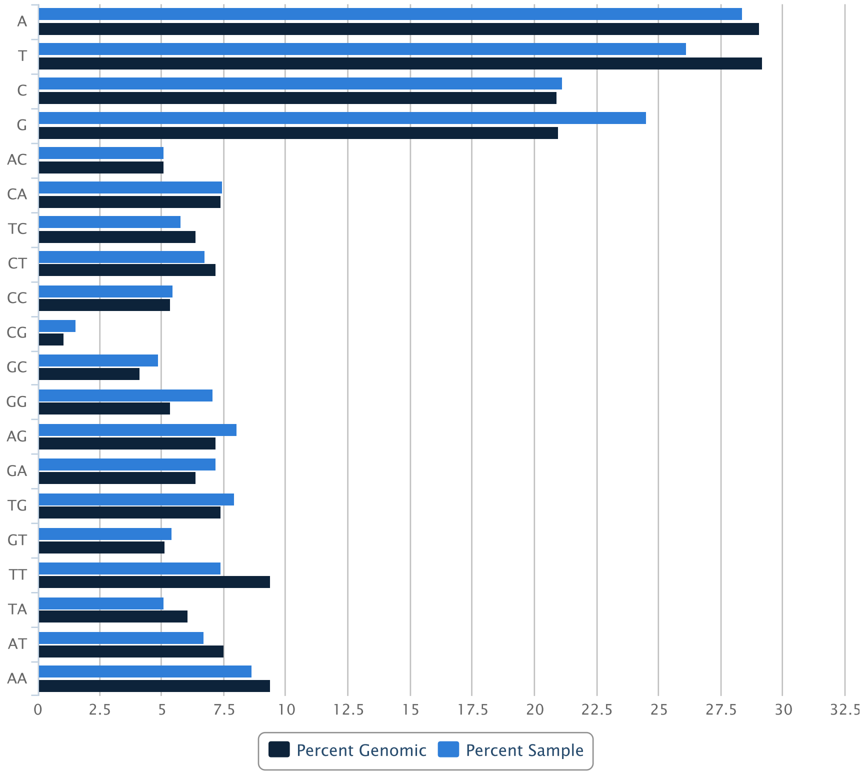

M-Bias Plot

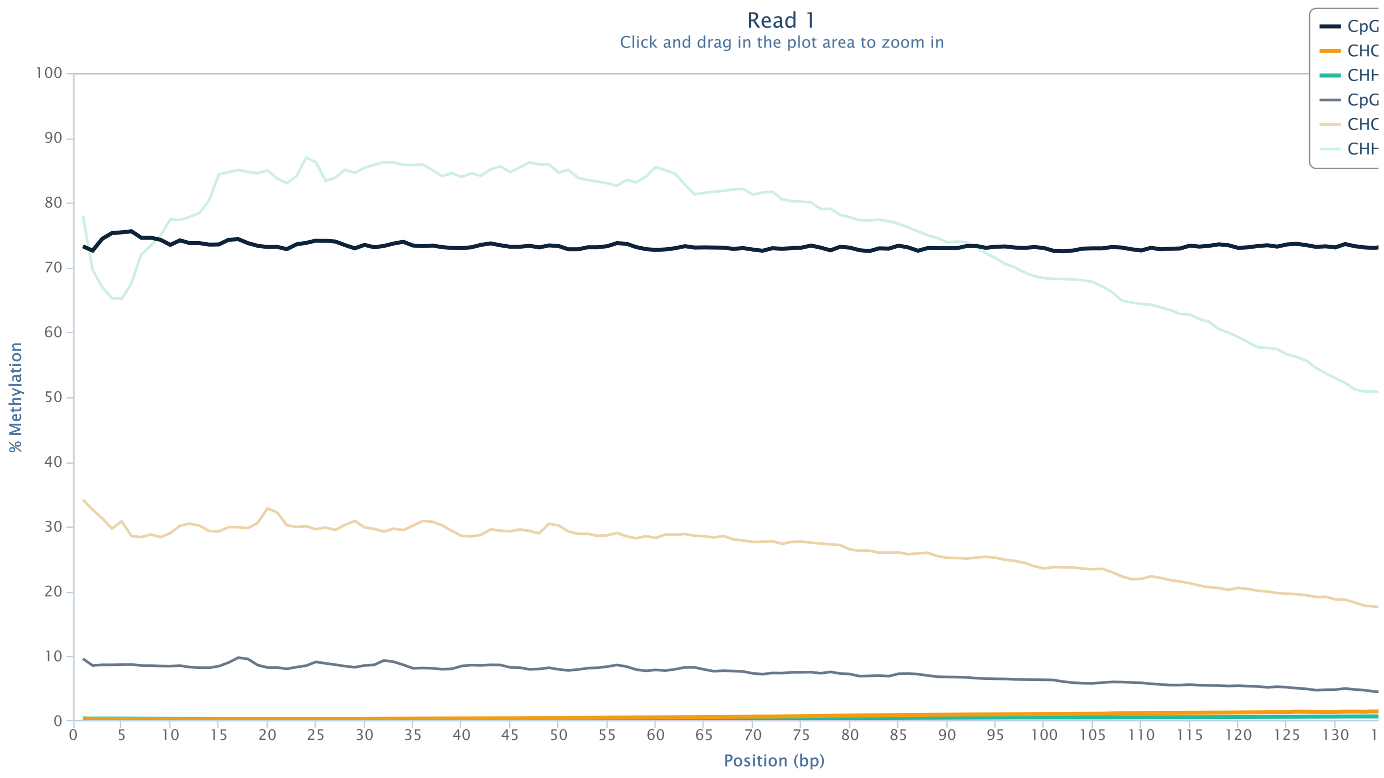

Analysis produced by **Bismark** (version v0.15.0) - a tool to map bisulfite converted sequence reads and determine cytosine methylation states

Report graphs rendered using jQuery and Highcharts. Page design by Phil Ewels.

Bismark Processing Report

trimgalore/Merged/M5_ACTTGA_R1_merged_val_1.fq.gz and trimgalore/Merged/M5_ACTTGA_R2_merged_val_2.fq.gz

Data processed at 16:37 on 2016-05-01

Alignment

**Sequence pairs analysed in total**	53477317
**Paired-end alignments with a unique best hit**	31927779
**Pairs without alignments under any condition**	12498701
**Pairs that did not map uniquely**	9050837
**Genomic sequence context not extractable (edges of chromosomes)**	244

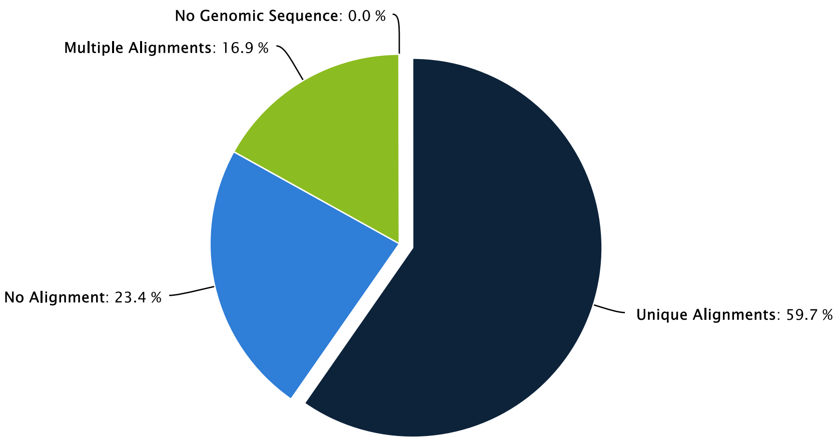

Cytosine Methylation

**Total C's analysed**	1743359957
**Methylated C's in CpG context**	96510322
**Methylated C's in CHG context**	6908765
**Methylated C's in CHH context**	9929321
**Methylated C's in Unknown context**	9693
**Unmethylated C's in CpG context**	36733019
**Unmethylated C's in CHG context**	432911120
**Unmethylated C's in CHH context**	1160367410
**Unmethylated C's in Unknown context**	24113
**Percentage methylation (CpG context)**	72.4%
**Percentage methylation (CHG context)**	1.6%
**Percentage methylation (CHH context)**	0.8%

**Methylated C's in Unknown context**	N/A%

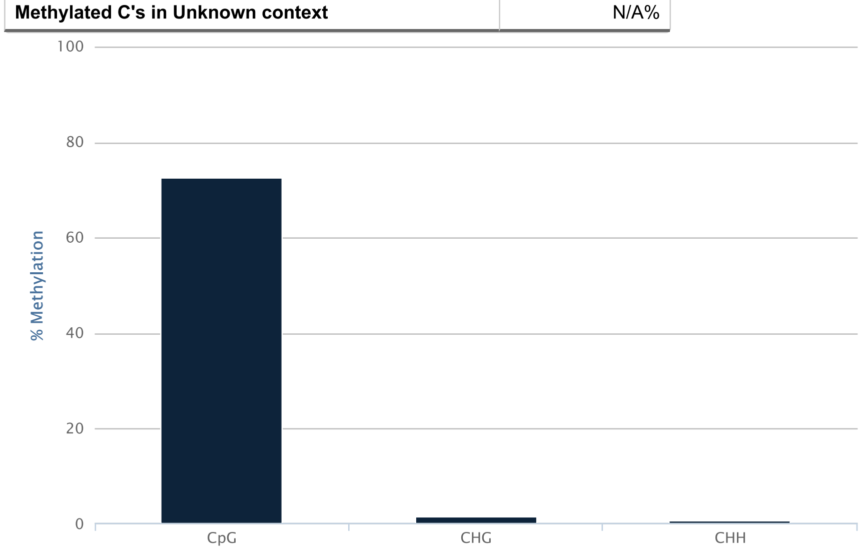

Alignment to Individual Bisulfite Strands

**OT**	15980726	original top strand
**CTOT**	0	complementary to original top strand
**CTOB**	0	complementary to original bottom strand
**OB**	15946809	original bottom strand

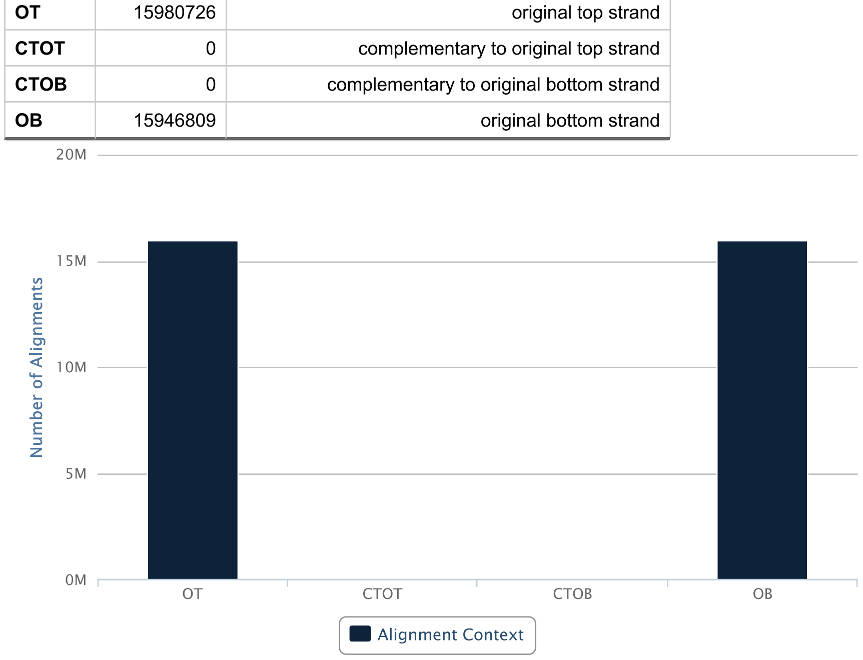

Deduplication

**Alignments analysed**	31927535
**Unique alignments**	25936243
**Duplicates removed**	5991292
Duplicated alignments were found at **4740732** different positions	

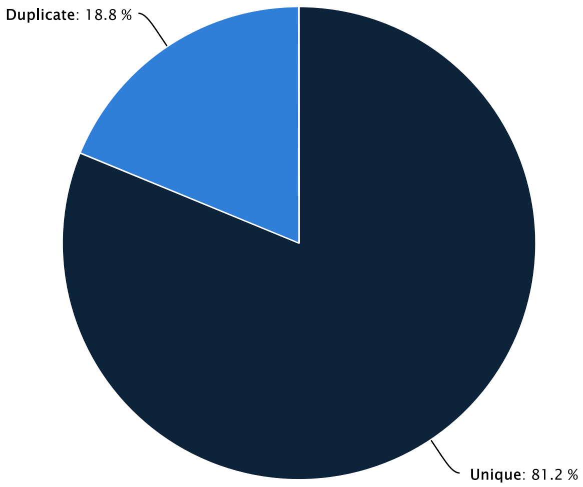

Cytosine Methylation after Extraction

**Total C's analysed**	862863612
**Methylated C's in CpG context**	43685536
**Methylated C's in CHG context**	1665427
**Methylated C's in CHH context**	3388646
**Unmethylated C's in CpG context**	16938016
**Unmethylated C's in CHG context**	210843048
**Unmethylated C's in CHH context**	586342939
**Percentage methylation (CpG context)**	72.1%
**Percentage methylation (CHG context)**	0.8%
**Percentage methylation (CHH context)**	0.6%

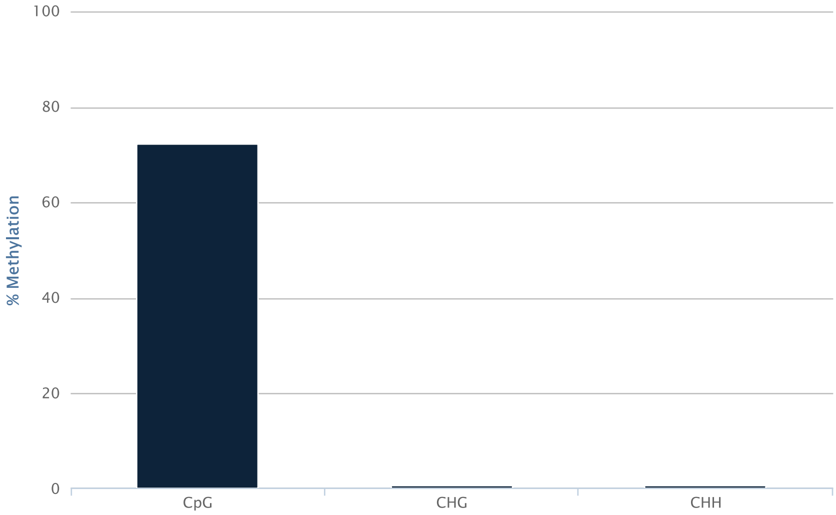

Nucleotide Coverage

Nucleotide Class	Counts Sample	Counts Genome	% in Sample	% in Genome
**A**	2022290181	769902373	27.61	29.06
**T**	1877162156	771539118	25.63	29.12
**C**	1570199364	553941589	21.44	20.91
**G**	1854901016	554298949	25.32	20.92
**AC**	364602933	134825334	5.02	5.09
**CA**	537690479	194999195	7.40	7.36
**TC**	420193031	168302551	5.78	6.35

Nucleotide Class	Counts Sample	Counts Genome	% in Sample	% in Genome
**CT**	491088089	189605870	6.76	7.16
**CC**	407478453	141780183	5.61	5.35
**CG**	119954274	27540367	1.65	1.04
**GC**	366593960	109014748	5.04	4.11
**GG**	548640178	141809778	7.55	5.35
**AG**	586752842	189448103	8.07	7.15
**GA**	523726872	168131316	7.21	6.35
**TG**	583893482	195484730	8.04	7.38
**GT**	395914545	135324159	5.45	5.11
**TT**	513675360	248110054	7.07	9.36
**TA**	346287543	159621424	4.77	6.02
**AT**	464710079	198476252	6.40	7.49
**AA**	595566912	247130183	8.20	9.33

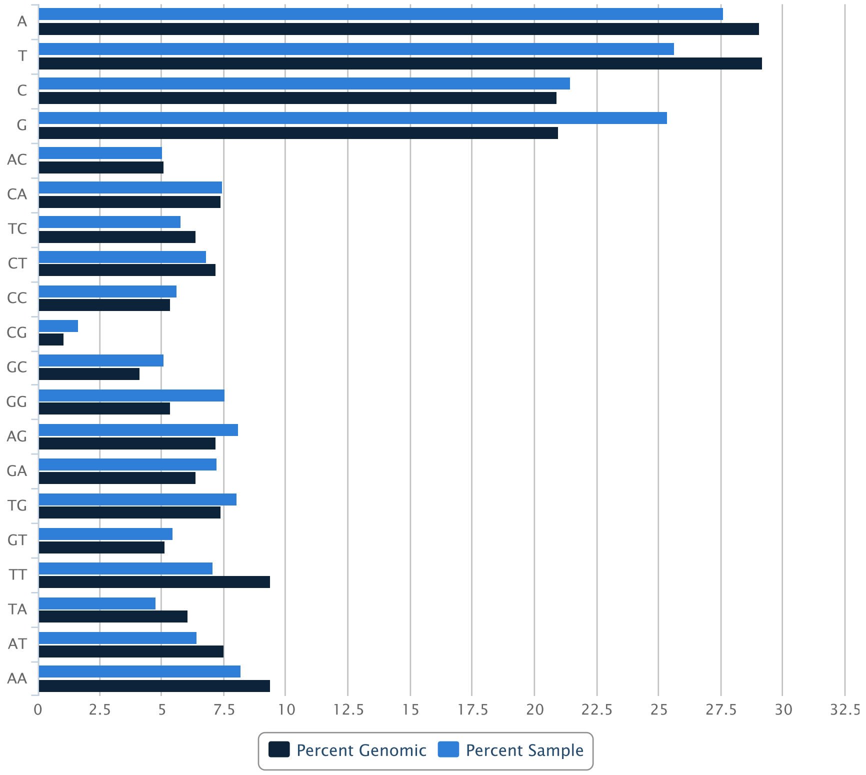

M-Bias Plot

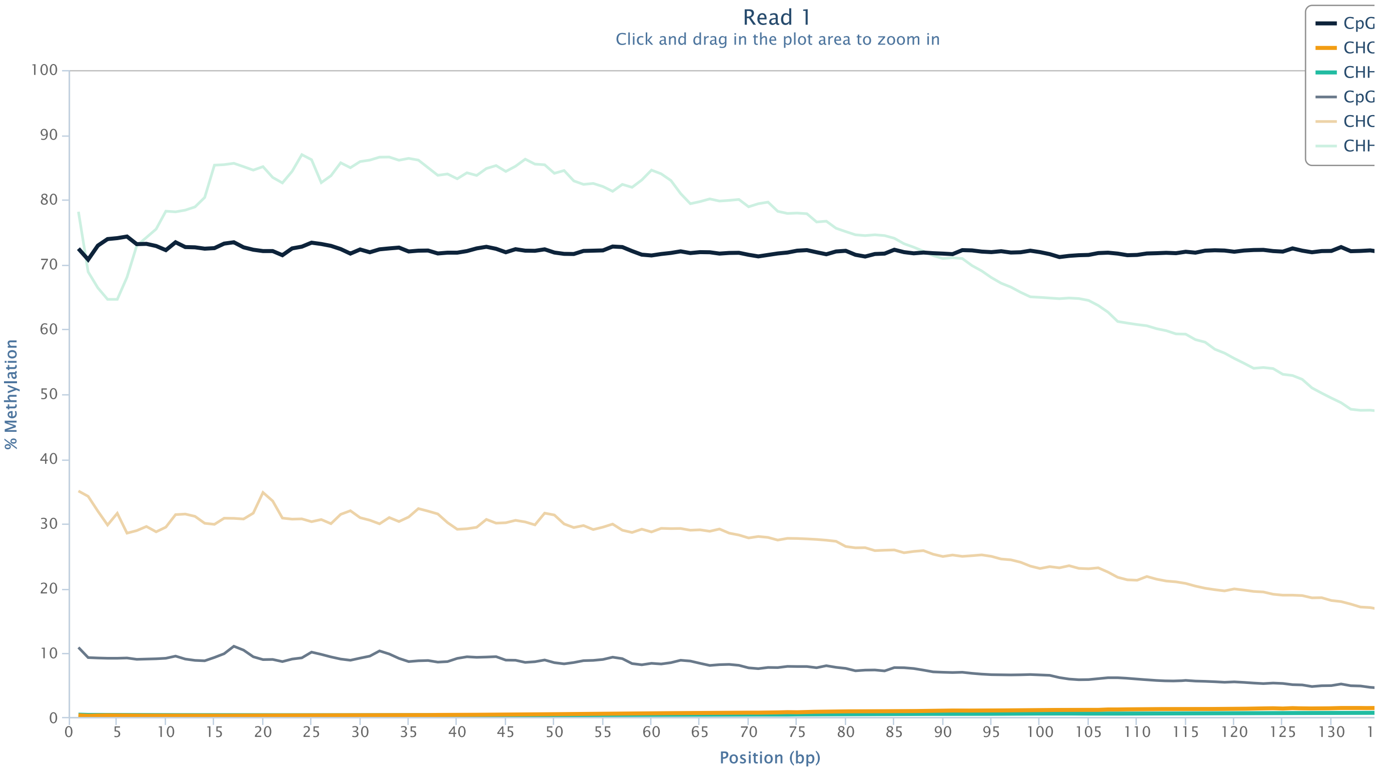

Read 1
Click and drag in the plot area to zoom in
% Methylation
Position (bp)
CpG
CHG
CHH
CpG
CHG
CHH

Bismark Processing Report

trimgalore/Merged/M6_GGCTAC_R1_merged_val_1.fq.gz and trimgalore/Merged/M6_GGCTAC_R2_merged_val_2.fq.gz

Data processed at 16:37 on 2016-05-01

Alignment

**Sequence pairs analysed in total**	47884860
**Paired-end alignments with a unique best hit**	31261958
**Pairs without alignments under any condition**	9586580
**Pairs that did not map uniquely**	7036322
**Genomic sequence context not extractable (edges of chromosomes)**	270

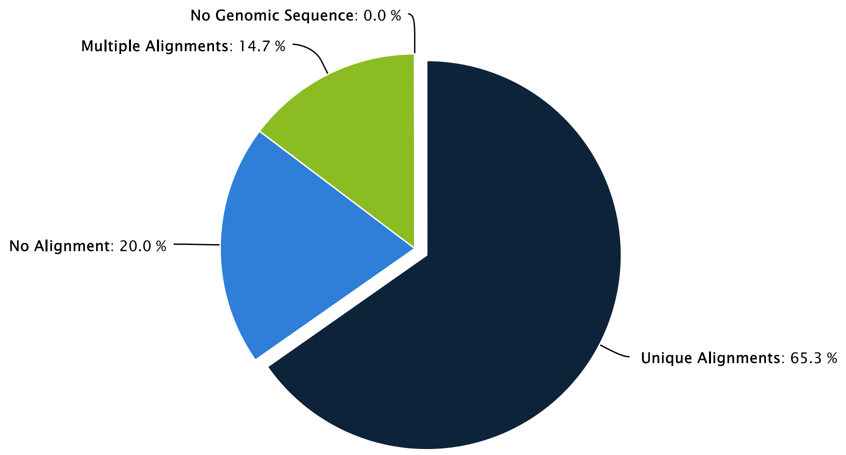

Cytosine Methylation

**Total C's analysed**	1791814926
**Methylated C's in CpG context**	93448938
**Methylated C's in CHG context**	6340559
**Methylated C's in CHH context**	9850425
**Methylated C's in Unknown context**	6989
**Unmethylated C's in CpG context**	33195399
**Unmethylated C's in CHG context**	435186966
**Unmethylated C's in CHH context**	1213792639
**Unmethylated C's in Unknown context**	20771
**Percentage methylation (CpG context)**	73.8%
**Percentage methylation (CHG context)**	1.4%
**Percentage methylation (CHH context)**	0.8%

**Methylated C's in Unknown context**	N/A%

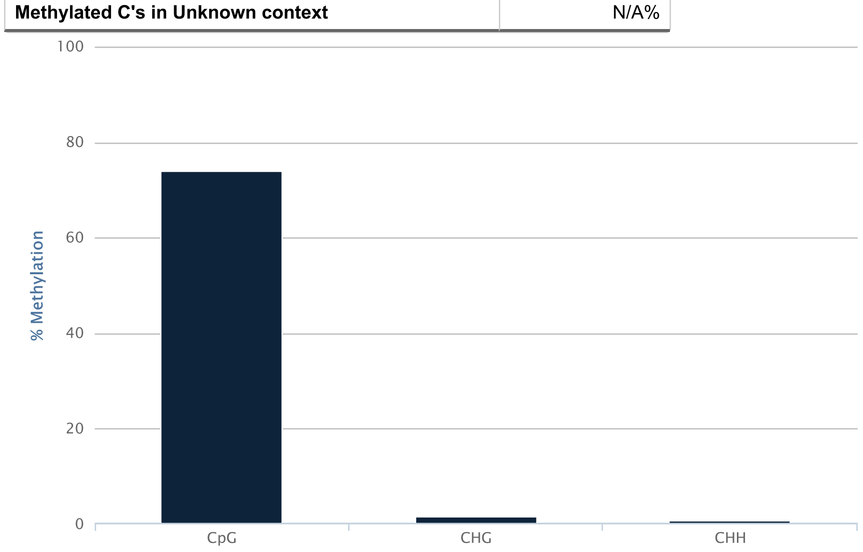

Alignment to Individual Bisulfite Strands

**OT**	15634317	original top strand
**CTOT**	0	complementary to original top strand
**CTOB**	0	complementary to original bottom strand
**OB**	15627371	original bottom strand

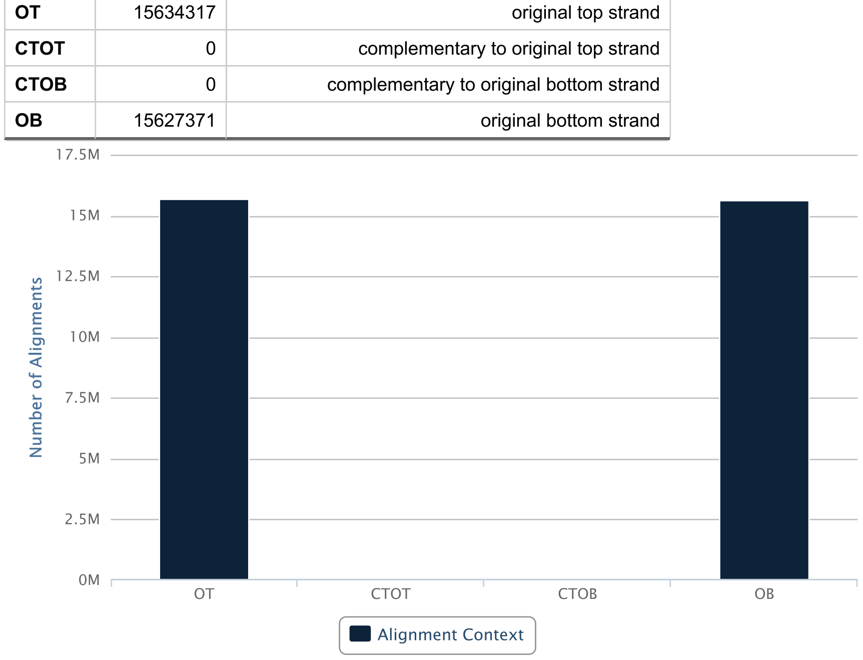

Deduplication

**Alignments analysed**	31261688
**Unique alignments**	28338806
**Duplicates removed**	2922882
Duplicated alignments were found at **2478679** different positions	

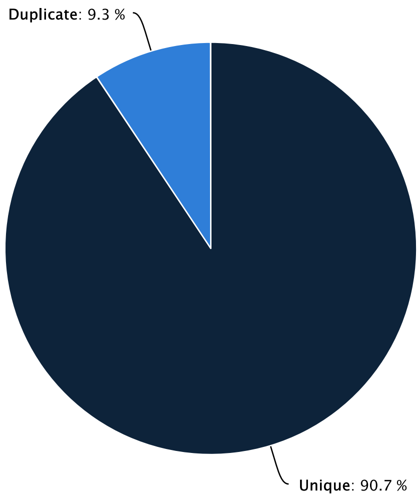

Cytosine Methylation after Extraction

**Total C's analysed**	1056624108
**Methylated C's in CpG context**	50855380
**Methylated C's in CHG context**	1730353
**Methylated C's in CHH context**	3988016
**Unmethylated C's in CpG context**	18259801
**Unmethylated C's in CHG context**	252853599
**Unmethylated C's in CHH context**	728936959
**Percentage methylation (CpG context)**	73.6%
**Percentage methylation (CHG context)**	0.7%
**Percentage methylation (CHH context)**	0.5%

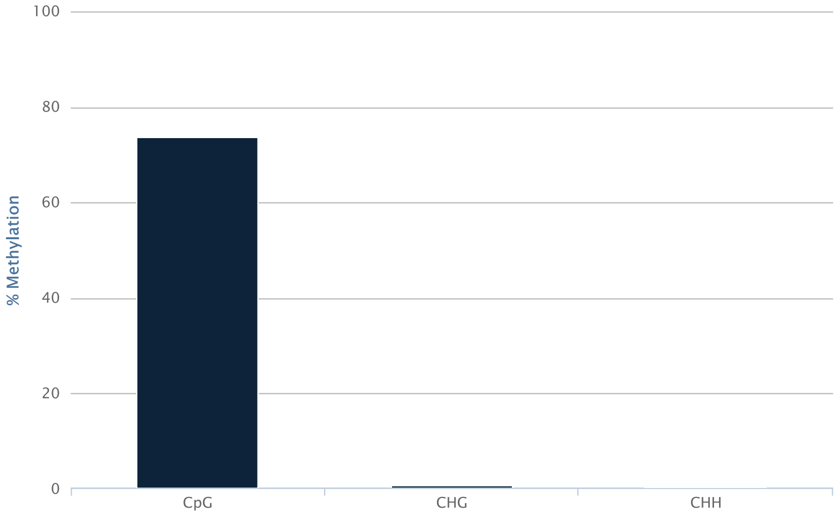

Nucleotide Coverage

Nucleotide Class	Counts Sample	Counts Genome	% in Sample	% in Genome
**A**	2160268205	769902373	28.36	29.06
**T**	1990905646	771539118	26.14	29.12
**C**	1609779152	553941589	21.13	20.91
**G**	1856201619	554298949	24.37	20.92
**AC**	385080607	134825334	5.09	5.09
**CA**	562227435	194999195	7.44	7.36
**TC**	437606638	168302551	5.79	6.35

Nucleotide Class	Counts Sample	Counts Genome	% in Sample	% in Genome
**CT**	508882969	189605870	6.73	7.16
**CC**	411097196	141780183	5.44	5.35
**CG**	113699497	27540367	1.50	1.04
**GC**	365239739	109014748	4.83	4.11
**GG**	528122259	141809778	6.98	5.35
**AG**	601696401	189448103	7.96	7.15
**GA**	539416152	168131316	7.13	6.35
**TG**	597967014	195484730	7.91	7.38
**GT**	404880813	135324159	5.35	5.11
**TT**	558084664	248110054	7.38	9.36
**TA**	383573065	159621424	5.07	6.02
**AT**	507528247	198476252	6.71	7.49
**AA**	655714557	247130183	8.67	9.33

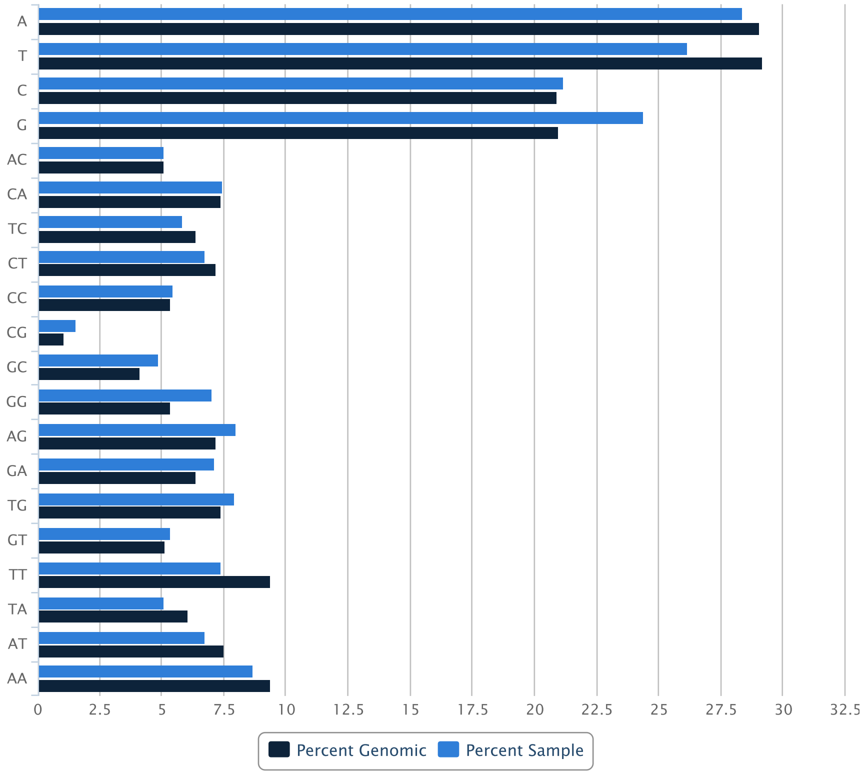

M-Bias Plot

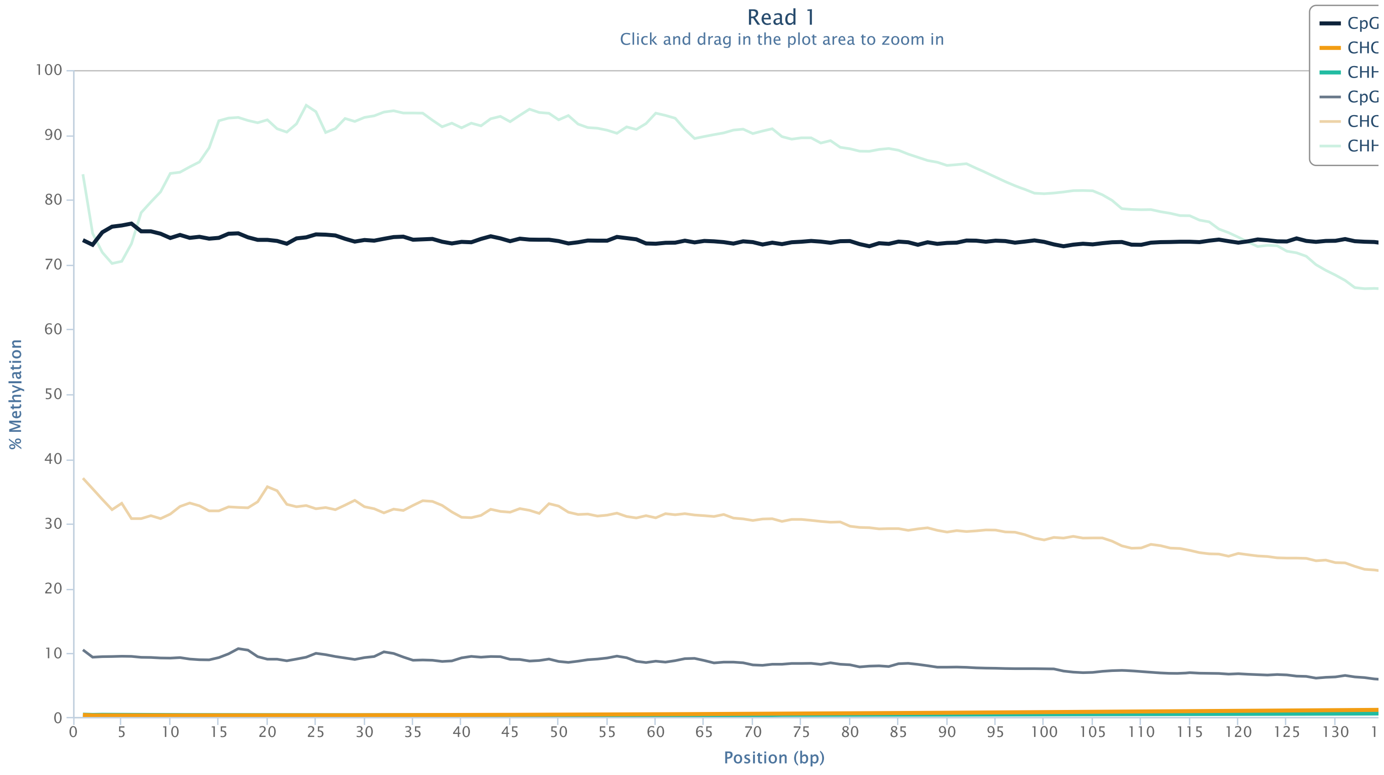

Read 1
Click and drag in the plot area to zoom in
% Methylation
Position (bp)
CpG
CHC
CHH
CpG
CHC
CHH

Bismark Processing Report

trimgalore/Merged/M7_CGATGT_R1_merged_val_1.fq.gz and trimgalore/Merged/M7_CGATGT_R2_merged_val_2.fq.gz

Data processed at 16:37 on 2016-05-01

Alignment

**Sequence pairs analysed in total**	54163984
**Paired-end alignments with a unique best hit**	33413587
**Pairs without alignments under any condition**	12425867
**Pairs that did not map uniquely**	8324530
**Genomic sequence context not extractable (edges of chromosomes)**	303

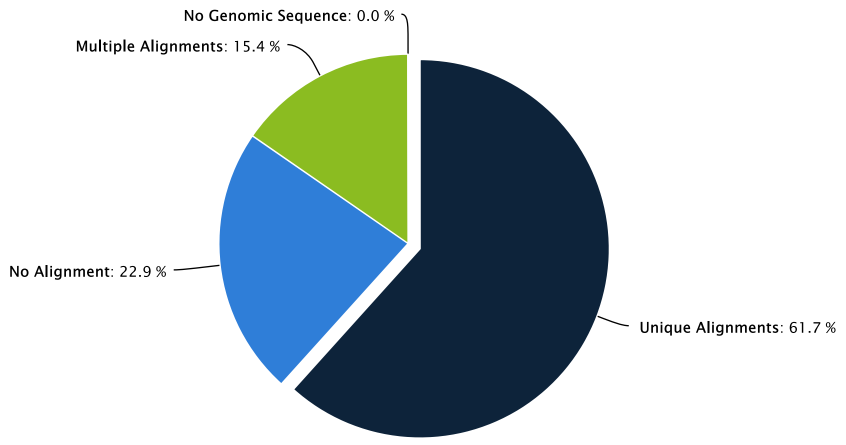

Cytosine Methylation

**Total C's analysed**	1824404255
**Methylated C's in CpG context**	95324788
**Methylated C's in CHG context**	6838197
**Methylated C's in CHH context**	10022825
**Methylated C's in Unknown context**	8304
**Unmethylated C's in CpG context**	36220251
**Unmethylated C's in CHG context**	447892716
**Unmethylated C's in CHH context**	1228105478
**Unmethylated C's in Unknown context**	23384
**Percentage methylation (CpG context)**	72.5%
**Percentage methylation (CHG context)**	1.5%
**Percentage methylation (CHH context)**	0.8%

**Methylated C's in Unknown context**	N/A%

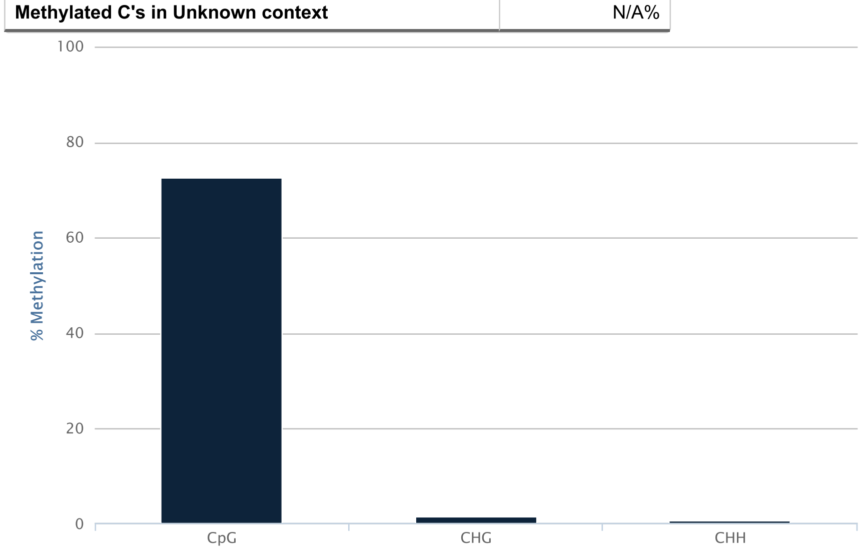

Alignment to Individual Bisulfite Strands

**OT**	16710530	original top strand
**CTOT**	0	complementary to original top strand
**CTOB**	0	complementary to original bottom strand
**OB**	16702754	original bottom strand

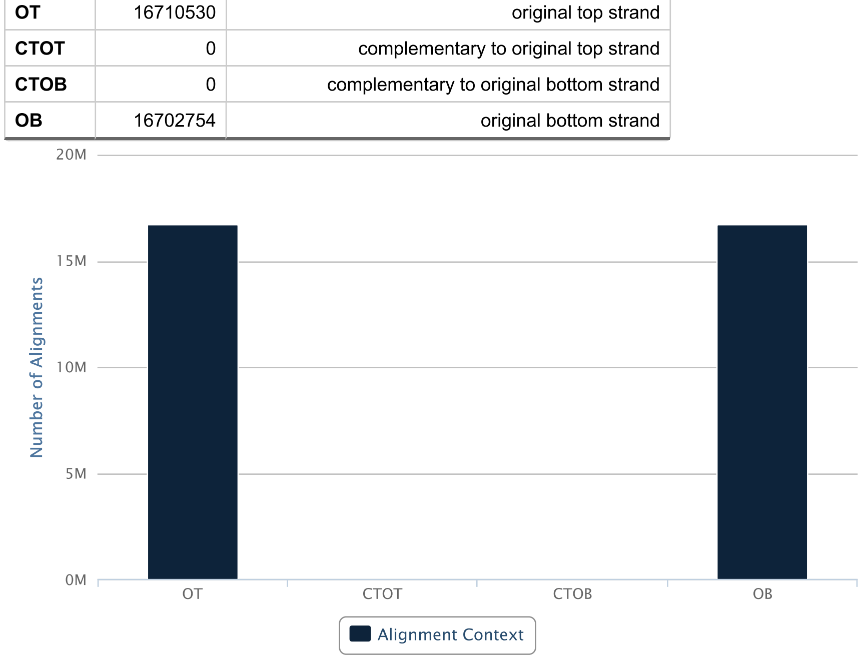

Deduplication

**Alignments analysed**	33413284
**Unique alignments**	28651831
**Duplicates removed**	4761453
Duplicated alignments were found at **3945508** different positions	

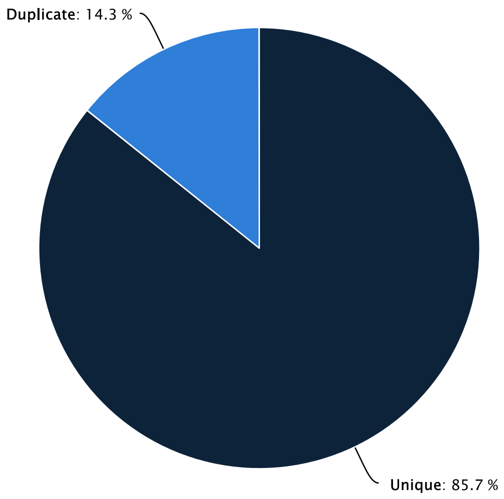

Cytosine Methylation after Extraction

**Total C's analysed**	951648491
**Methylated C's in CpG context**	46046005
**Methylated C's in CHG context**	1755011
**Methylated C's in CHH context**	3528852
**Unmethylated C's in CpG context**	17721761
**Unmethylated C's in CHG context**	229957639
**Unmethylated C's in CHH context**	652639223
**Percentage methylation (CpG context)**	72.2%
**Percentage methylation (CHG context)**	0.8%
**Percentage methylation (CHH context)**	0.5%

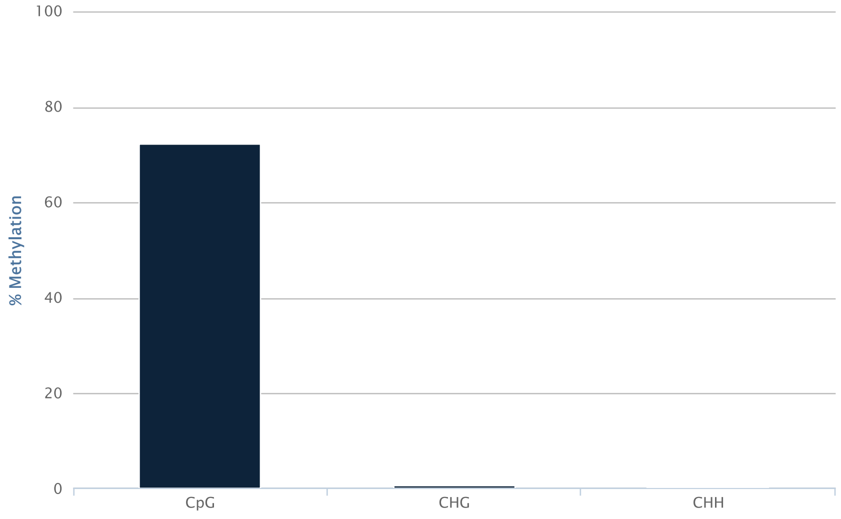

Nucleotide Coverage

Nucleotide Class	Counts Sample	Counts Genome	% in Sample	% in Genome
**A**	2189058228	769902373	28.15	29.06
**T**	2018103485	771539118	25.95	29.12
**C**	1646210764	553941589	21.17	20.91
**G**	1922454905	554298949	24.72	20.92
**AC**	390259708	134825334	5.06	5.09
**CA**	572932201	194999195	7.43	7.36
**TC**	445010842	168302551	5.77	6.35

Nucleotide Class	Counts Sample	Counts Genome	% in Sample	% in Genome
CT	518590908	189605870	6.72	7.16
CC	421463679	141780183	5.46	5.35
CG	118644224	27540367	1.54	1.04
GC	377427508	109014748	4.89	4.11
GG	555707569	141809778	7.20	5.35
AG	618975087	189448103	8.02	7.15
GA	553542225	168131316	7.17	6.35
TG	613675964	195484730	7.95	7.38
GT	415190437	135324159	5.38	5.11
TT	560670569	248110054	7.27	9.36
TA	384688982	159621424	4.99	6.02
AT	510970236	198476252	6.62	7.49
AA	657535831	247130183	8.52	9.33

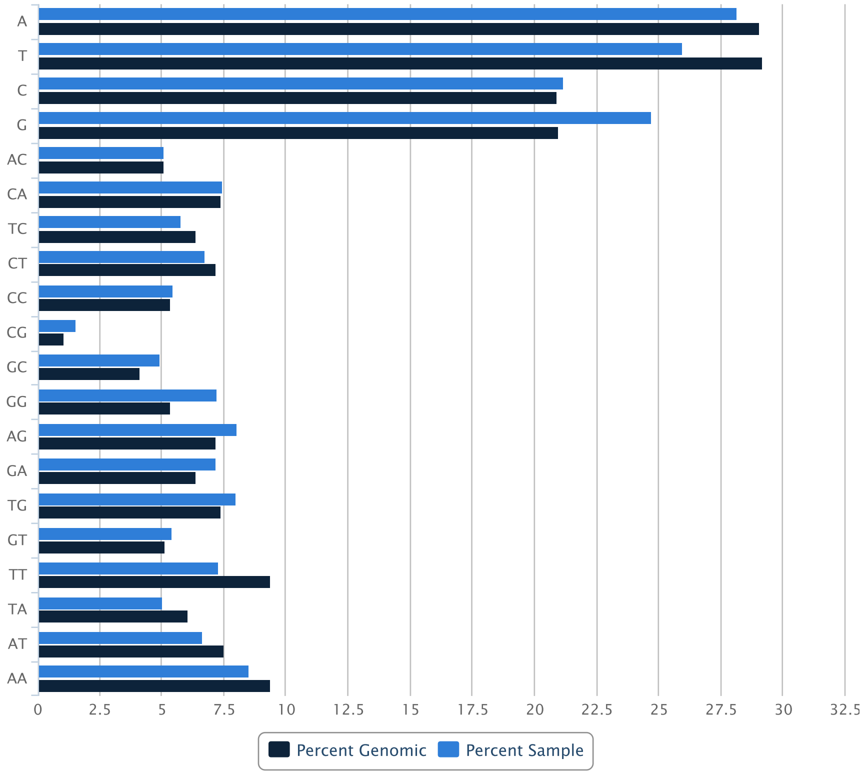

M-Bias Plot

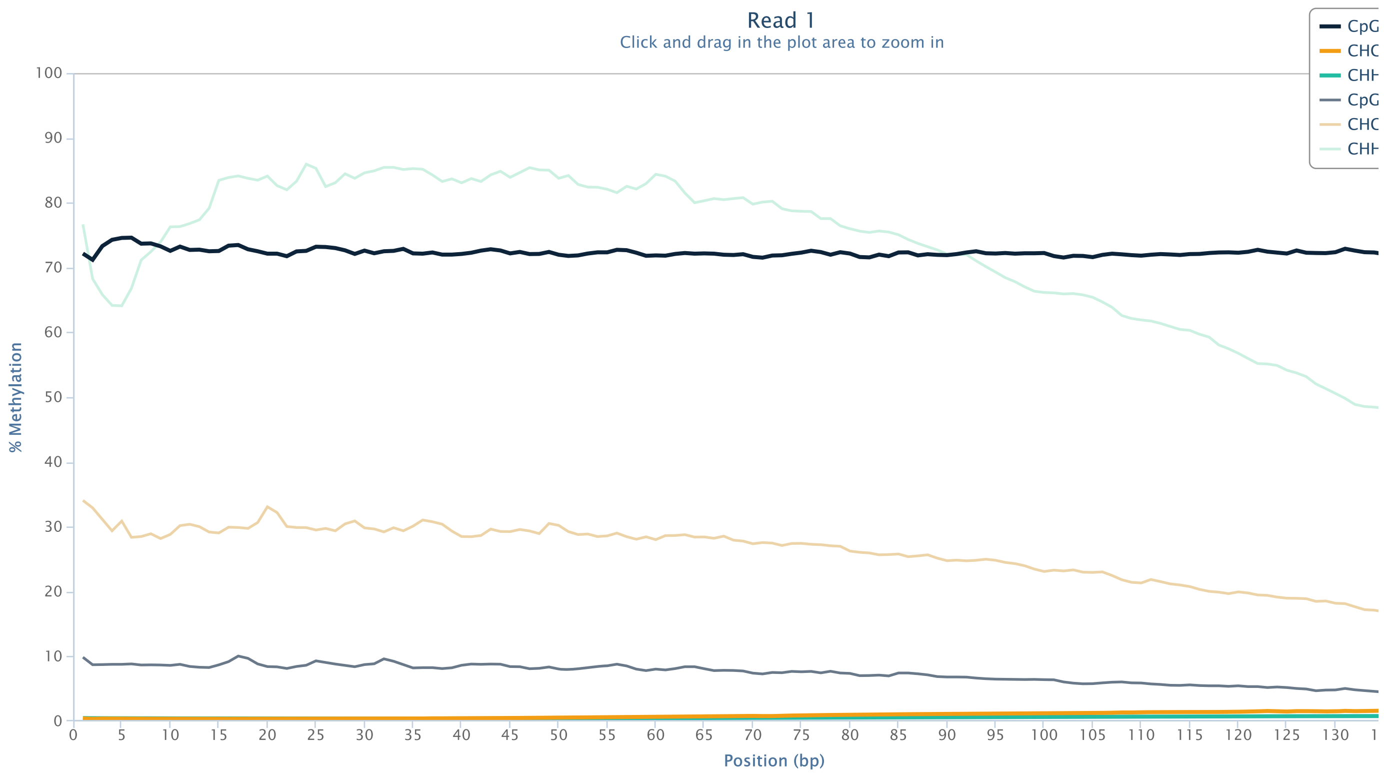
Read 1
Click and drag in the plot area to zoom in
% Methylation
Position (bp)
CpG
CHC
CHH
CpG
CHC
CHH

Bismark Processing Report

trimgalore/Merged/M8_ACAGTG_R1_merged_val_1.fq.gz and trimgalore/Merged/M8_ACAGTG_R2_merged_val_2.fq.gz

Data processed at 16:37 on 2016-05-01

Alignment

**Sequence pairs analysed in total**	50674513
**Paired-end alignments with a unique best hit**	33099431
**Pairs without alignments under any condition**	10155427
**Pairs that did not map uniquely**	7419655
**Genomic sequence context not extractable (edges of chromosomes)**	206

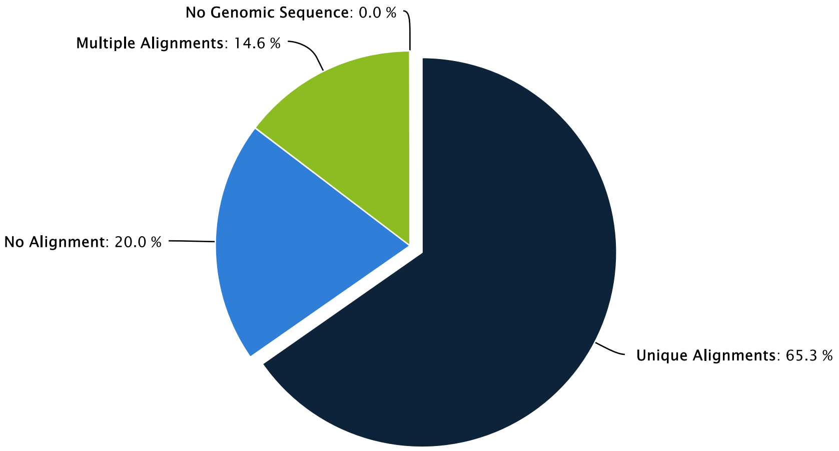

Cytosine Methylation

**Total C's analysed**	1847939703
**Methylated C's in CpG context**	88079935
**Methylated C's in CHG context**	6515062
**Methylated C's in CHH context**	9922547
**Methylated C's in Unknown context**	6825
**Unmethylated C's in CpG context**	38232611
**Unmethylated C's in CHG context**	446740861
**Unmethylated C's in CHH context**	1258448687
**Unmethylated C's in Unknown context**	20747
**Percentage methylation (CpG context)**	69.7%
**Percentage methylation (CHG context)**	1.4%
**Percentage methylation (CHH context)**	0.8%

**Methylated C's in Unknown context**	N/A%

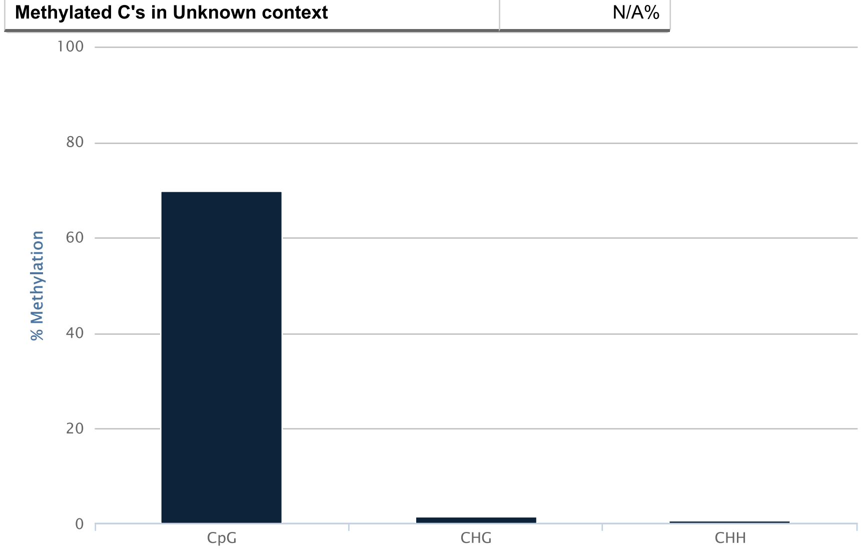

Alignment to Individual Bisulfite Strands

**OT**	16554437	original top strand
**CTOT**	0	complementary to original top strand
**CTOB**	0	complementary to original bottom strand
**OB**	16544788	original bottom strand

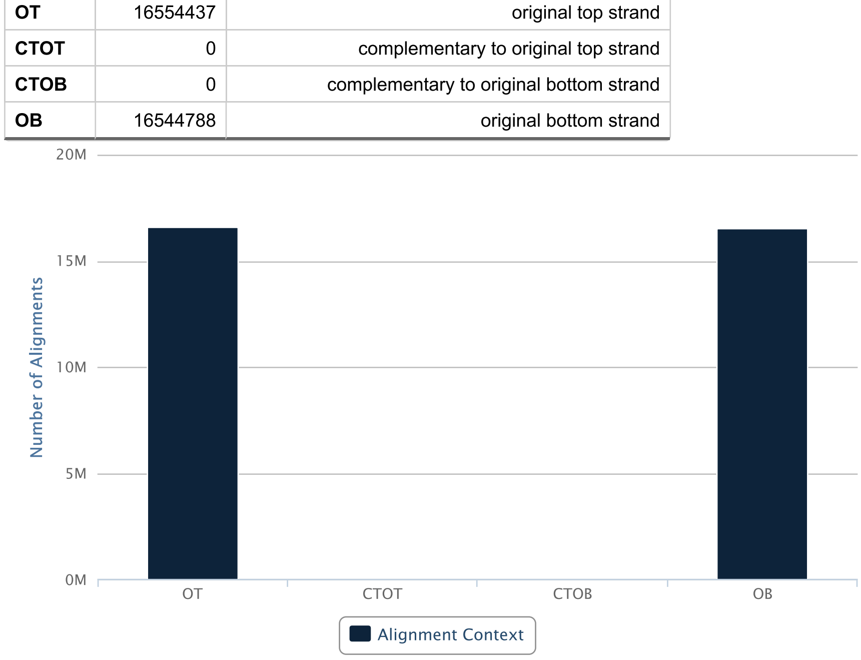

Deduplication

**Alignments analysed**	33099225
**Unique alignments**	30324167
**Duplicates removed**	2775058
Duplicated alignments were found at **2370483** different positions	

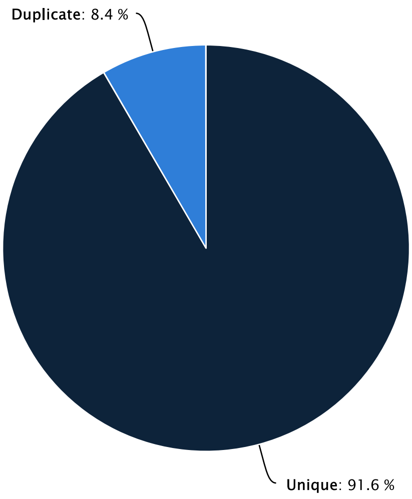

Cytosine Methylation after Extraction

**Total C's analysed**	1055111603
**Methylated C's in CpG context**	46436665
**Methylated C's in CHG context**	1727837
**Methylated C's in CHH context**	3661362
**Unmethylated C's in CpG context**	20446566
**Unmethylated C's in CHG context**	251201072
**Unmethylated C's in CHH context**	731638101
**Percentage methylation (CpG context)**	69.4%
**Percentage methylation (CHG context)**	0.7%
**Percentage methylation (CHH context)**	0.5%

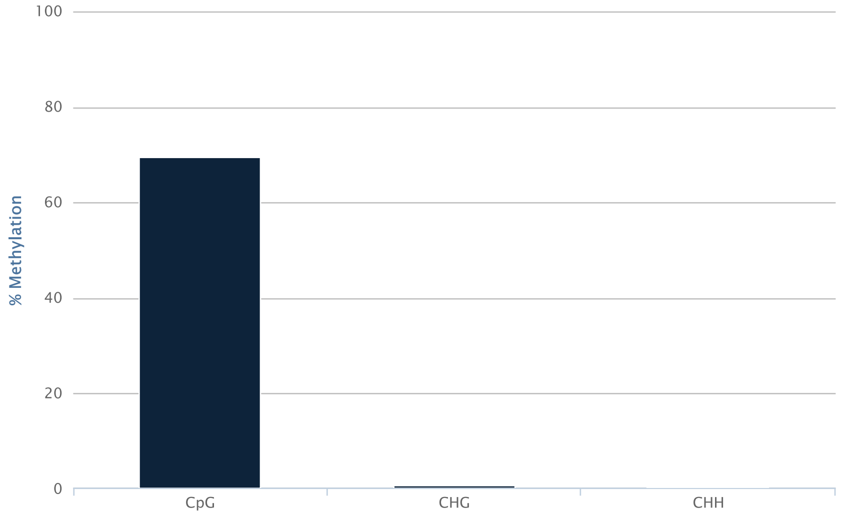

Nucleotide Coverage

Nucleotide Class	Counts Sample	Counts Genome	% in Sample	% in Genome
**A**	2268281986	769902373	28.60	29.06
**T**	2079878725	771539118	26.23	29.12
**C**	1664739773	553941589	20.99	20.91
**G**	1916967728	554298949	24.17	20.92
**AC**	402279208	134825334	5.11	5.09
**CA**	585331661	194999195	7.44	7.36
**TC**	453971111	168302551	5.77	6.35

Nucleotide Class	Counts Sample	Counts Genome	% in Sample	% in Genome
**CT**	528031292	189605870	6.71	7.16
**CC**	423249274	141780183	5.38	5.35
**CG**	113673245	27540367	1.44	1.04
**GC**	373676832	109014748	4.75	4.11
**GG**	542773808	141809778	6.90	5.35
**AG**	626313304	189448103	7.96	7.15
**GA**	561720680	168131316	7.14	6.35
**TG**	619197975	195484730	7.87	7.38
**GT**	418940737	135324159	5.32	5.11
**TT**	585932621	248110054	7.45	9.36
**TA**	406447811	159621424	5.16	6.02
**AT**	534497530	198476252	6.79	7.49
**AA**	694002909	247130183	8.82	9.33

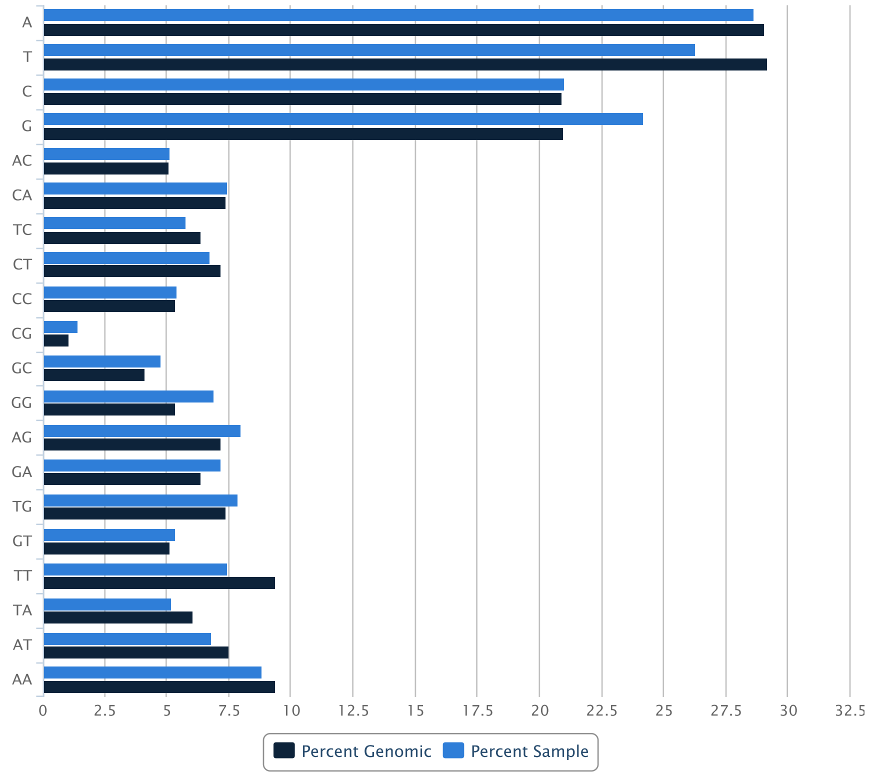

M-Bias Plot

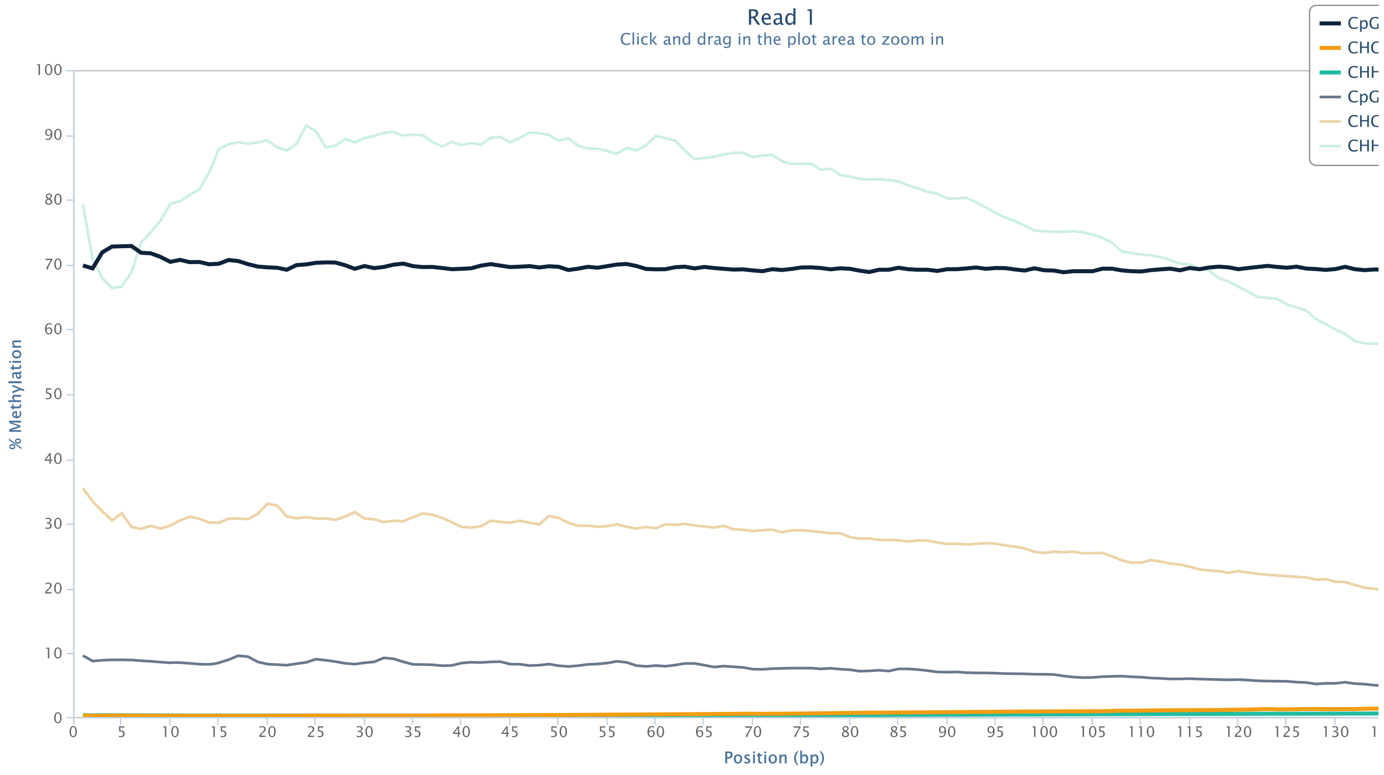

Read 1
Click and drag in the plot area to zoom in
% Methylation
Position (bp)

Bismark Processing Report

trimgalore/Merged/M11_ACTTGA_R1_merged_val_1.fq.gz and trimgalore/Merged/M11_ACTTGA_R2_merged_val_2.fq.gz

Data processed at 16:37 on 2016-05-01

Alignment

**Sequence pairs analysed in total**	57763402
**Paired-end alignments with a unique best hit**	39263322
**Pairs without alignments under any condition**	10514250
**Pairs that did not map uniquely**	7985830
**Genomic sequence context not extractable (edges of chromosomes)**	269

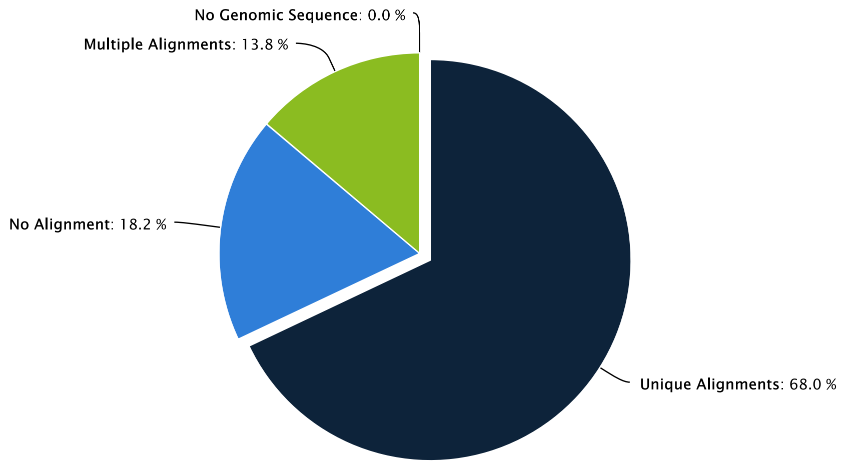

Cytosine Methylation

**Total C's analysed**	2206273014
**Methylated C's in CpG context**	102840964
**Methylated C's in CHG context**	7536342
**Methylated C's in CHH context**	12391833
**Methylated C's in Unknown context**	6899
**Unmethylated C's in CpG context**	39486452
**Unmethylated C's in CHG context**	525794035
**Unmethylated C's in CHH context**	1518223388
**Unmethylated C's in Unknown context**	23460
**Percentage methylation (CpG context)**	72.3%
**Percentage methylation (CHG context)**	1.4%
**Percentage methylation (CHH context)**	0.8%

**Methylated C's in Unknown context**	N/A%

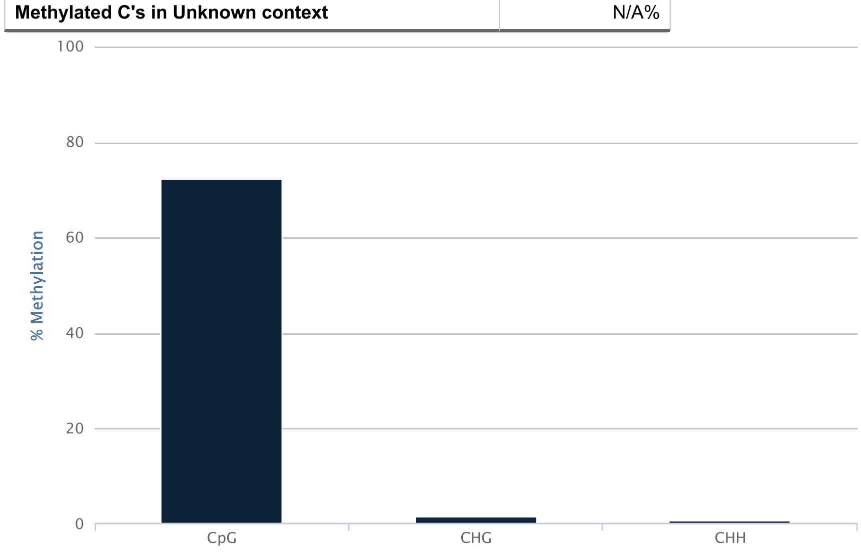

Alignment to Individual Bisulfite Strands

**OT**	19631917	original top strand
**CTOT**	0	complementary to original top strand
**CTOB**	0	complementary to original bottom strand
**OB**	19631136	original bottom strand

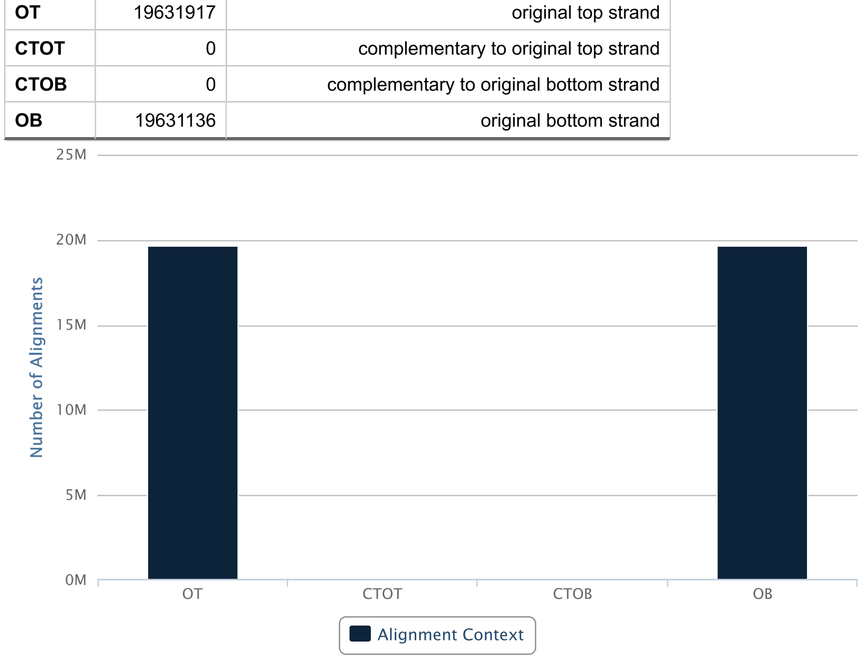

Deduplication

**Alignments analysed**	39263053
**Unique alignments**	36053693
**Duplicates removed**	3209360
Duplicated alignments were found at **2768915** different positions	

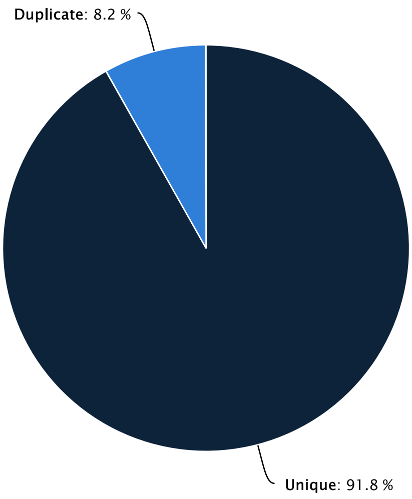

Cytosine Methylation after Extraction

**Total C's analysed**	1248879412
**Methylated C's in CpG context**	54349076
**Methylated C's in CHG context**	2174714
**Methylated C's in CHH context**	4792078
**Unmethylated C's in CpG context**	20931185
**Unmethylated C's in CHG context**	292505059
**Unmethylated C's in CHH context**	874127300
**Percentage methylation (CpG context)**	72.2%
**Percentage methylation (CHG context)**	0.7%
**Percentage methylation (CHH context)**	0.5%

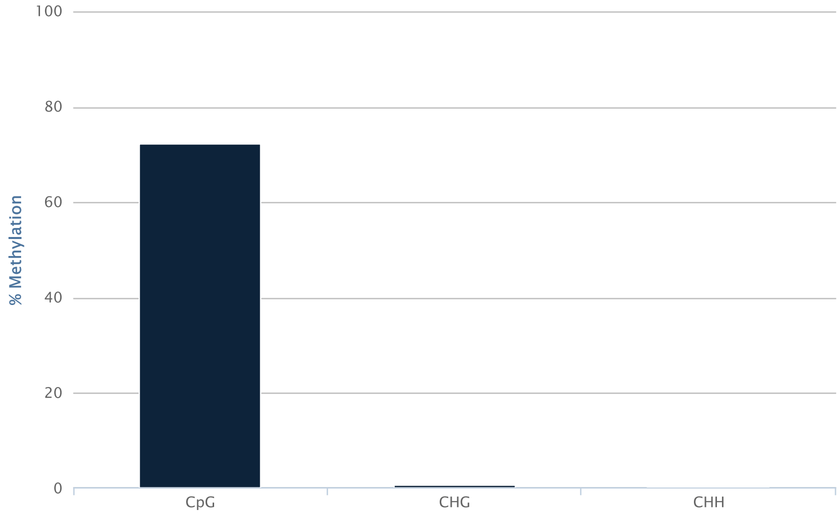

Nucleotide Coverage

Nucleotide Class	Counts Sample	Counts Genome	% in Sample	% in Genome
**A**	2679548437	769902373	28.51	29.06
**T**	2513885890	771539118	26.75	29.12
**C**	1984541988	553941589	21.12	20.91
**G**	2219469569	554298949	23.62	20.92
**AC**	476843633	134825334	5.11	5.09
**CA**	694768464	194999195	7.45	7.36
**TC**	550787989	168302551	5.91	6.35

Nucleotide Class	Counts Sample	Counts Genome	% in Sample	% in Genome
**CT**	637585748	189605870	6.84	7.16
**CC**	507051489	141780183	5.44	5.35
**CG**	127883464	27540367	1.37	1.04
**GC**	435432514	109014748	4.67	4.11
**GG**	617249568	141809778	6.62	5.35
**AG**	727591318	189448103	7.80	7.15
**GA**	649540207	168131316	6.96	6.35
**TG**	730240012	195484730	7.83	7.38
**GT**	494176854	135324159	5.30	5.11
**TT**	723707913	248110054	7.76	9.36
**TA**	491876963	159621424	5.27	6.02
**AT**	642931480	198476252	6.89	7.49
**AA**	818910456	247130183	8.78	9.33

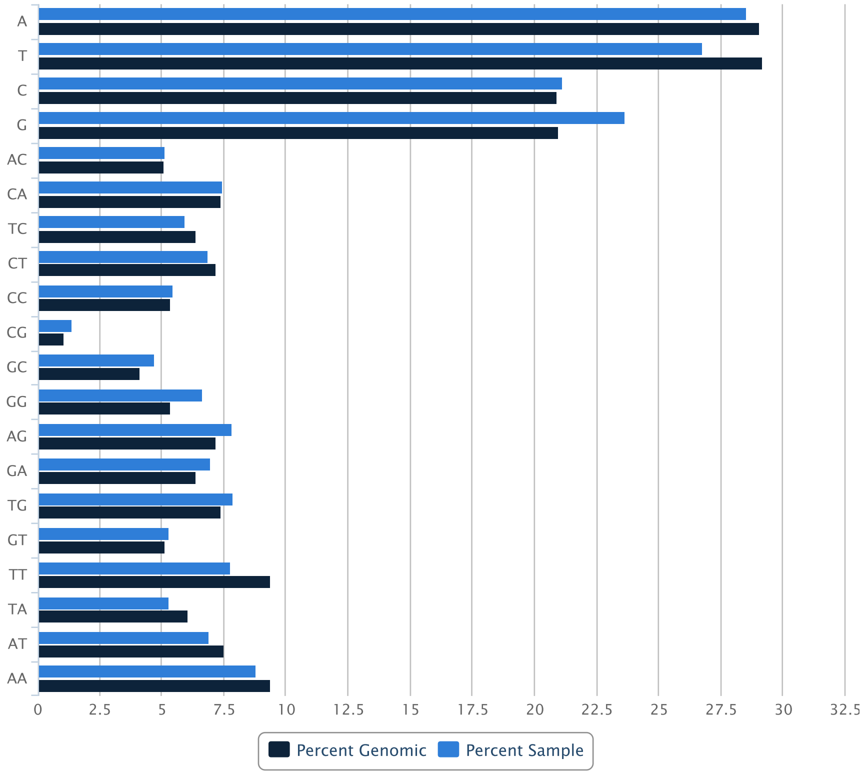

M-Bias Plot

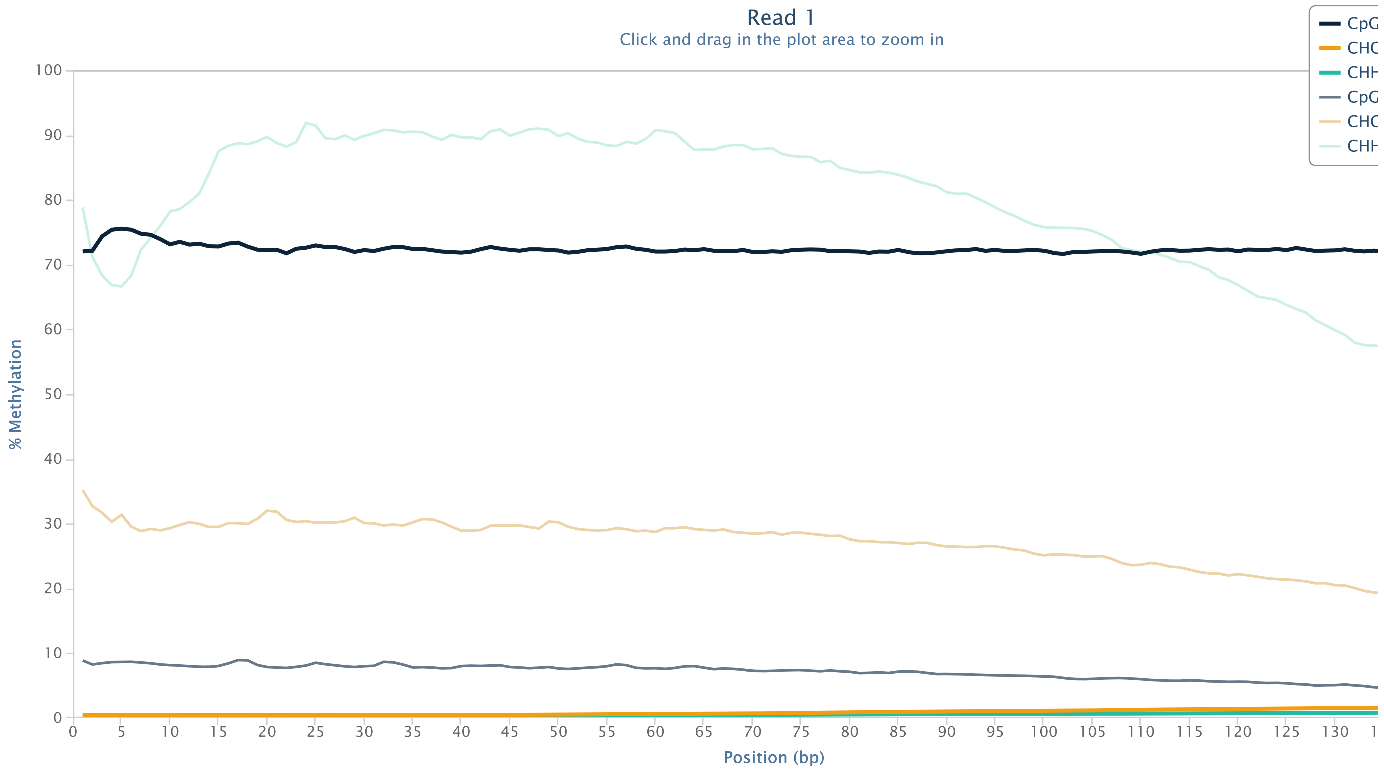

Read 1
Click and drag in the plot area to zoom in
% Methylation
Position (bp)
CpG
CHG
CHH
CpG
CHG
CHH

Bismark Processing Report

trimgalore/Merged/M12_GGCTAC_R1_merged_val_1.fq.gz and trimgalore/Merged/M12_GGCTAC_R2_merged_val_2.fq.gz

Data processed at 16:37 on 2016-05-01

Alignment

**Sequence pairs analysed in total**	59475816
**Paired-end alignments with a unique best hit**	38061115
**Pairs without alignments under any condition**	11751431
**Pairs that did not map uniquely**	9663270
**Genomic sequence context not extractable (edges of chromosomes)**	318

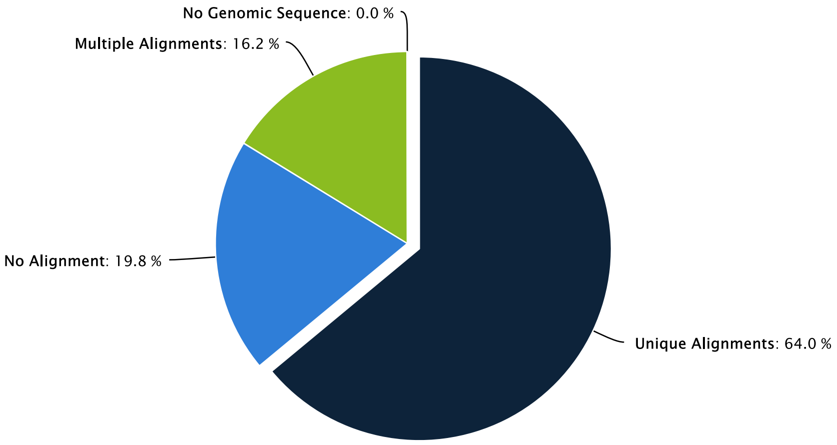

Cytosine Methylation

**Total C's analysed**	1956909314
**Methylated C's in CpG context**	94571053
**Methylated C's in CHG context**	8063168
**Methylated C's in CHH context**	12680632
**Methylated C's in Unknown context**	9939
**Unmethylated C's in CpG context**	35800691
**Unmethylated C's in CHG context**	474664430
**Unmethylated C's in CHH context**	1331129340
**Unmethylated C's in Unknown context**	27427
**Percentage methylation (CpG context)**	72.5%
**Percentage methylation (CHG context)**	1.7%
**Percentage methylation (CHH context)**	0.9%

**Methylated C's in Unknown context**	N/A%

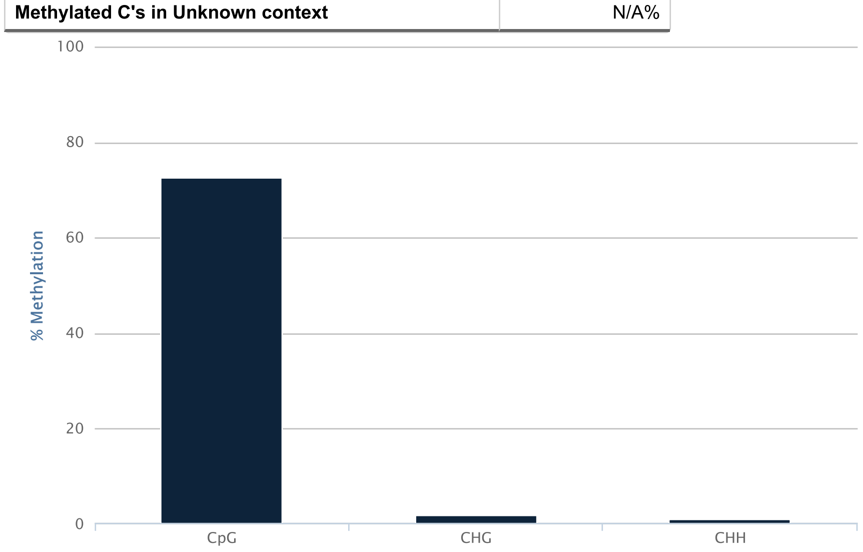

Alignment to Individual Bisulfite Strands

**OT**	19023396	original top strand
**CTOT**	0	complementary to original top strand
**CTOB**	0	complementary to original bottom strand
**OB**	19037401	original bottom strand

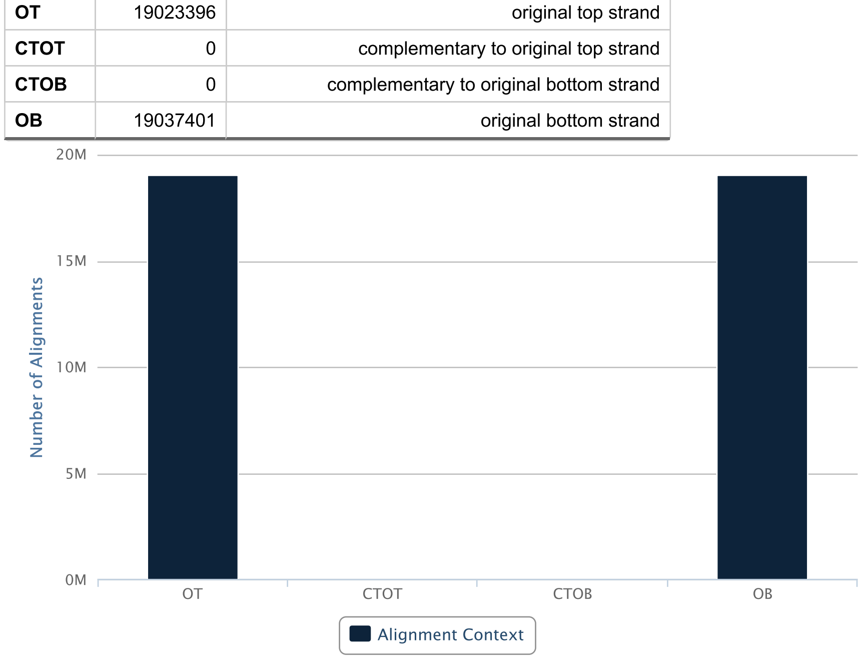

Deduplication

**Alignments analysed**	38060797
**Unique alignments**	34128585
**Duplicates removed**	3932212
Duplicated alignments were found at **3349059** different positions	

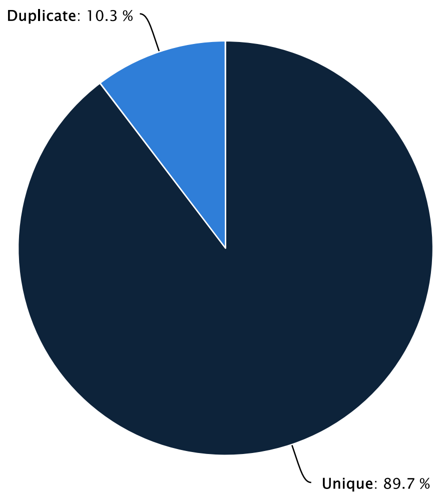

Cytosine Methylation after Extraction

**Total C's analysed**	1016417577
**Methylated C's in CpG context**	45794393
**Methylated C's in CHG context**	2381595
**Methylated C's in CHH context**	4683577
**Unmethylated C's in CpG context**	17337069
**Unmethylated C's in CHG context**	242006777
**Unmethylated C's in CHH context**	704214166
**Percentage methylation (CpG context)**	72.5%
**Percentage methylation (CHG context)**	1.0%
**Percentage methylation (CHH context)**	0.7%

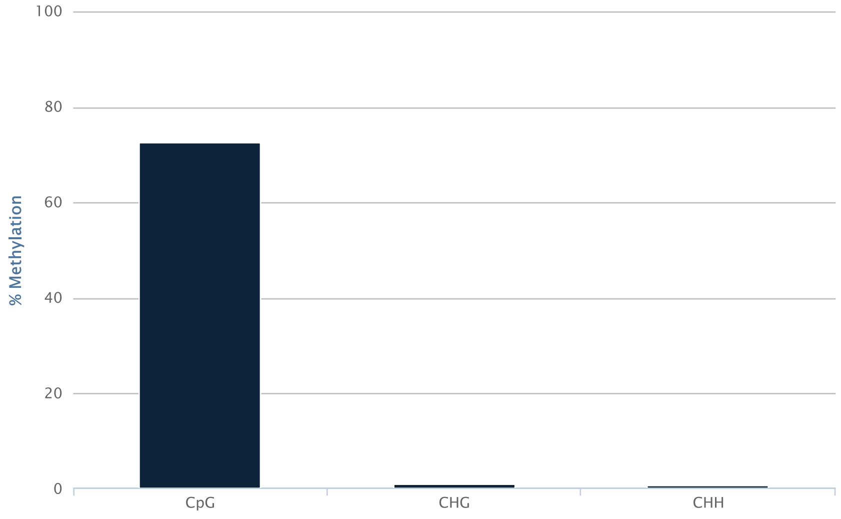

Nucleotide Coverage

Nucleotide Class	Counts Sample	Counts Genome	% in Sample	% in Genome
**A**	2387244695	769902373	28.36	29.06
**T**	2216637625	771539118	26.33	29.12
**C**	1774271201	553941589	21.08	20.91
**G**	2039213294	554298949	24.23	20.92
**AC**	423843133	134825334	5.08	5.09
**CA**	620452020	194999195	7.43	7.36
**TC**	485697133	168302551	5.82	6.35

Nucleotide Class	Counts Sample	Counts Genome	% in Sample	% in Genome
CT	565450332	189605870	6.77	7.16
CC	454012078	141780183	5.44	5.35
CG	118010561	27540367	1.41	1.04
GC	395850867	109014748	4.74	4.11
GG	580354677	141809778	6.95	5.35
AG	664599901	189448103	7.96	7.15
GA	594271312	168131316	7.12	6.35
TG	660418784	195484730	7.91	7.38
GT	445101851	135324159	5.33	5.11
TT	626546644	248110054	7.51	9.36
TA	428084147	159621424	5.13	6.02
AT	564131679	198476252	6.76	7.49
AA	721149371	247130183	8.64	9.33

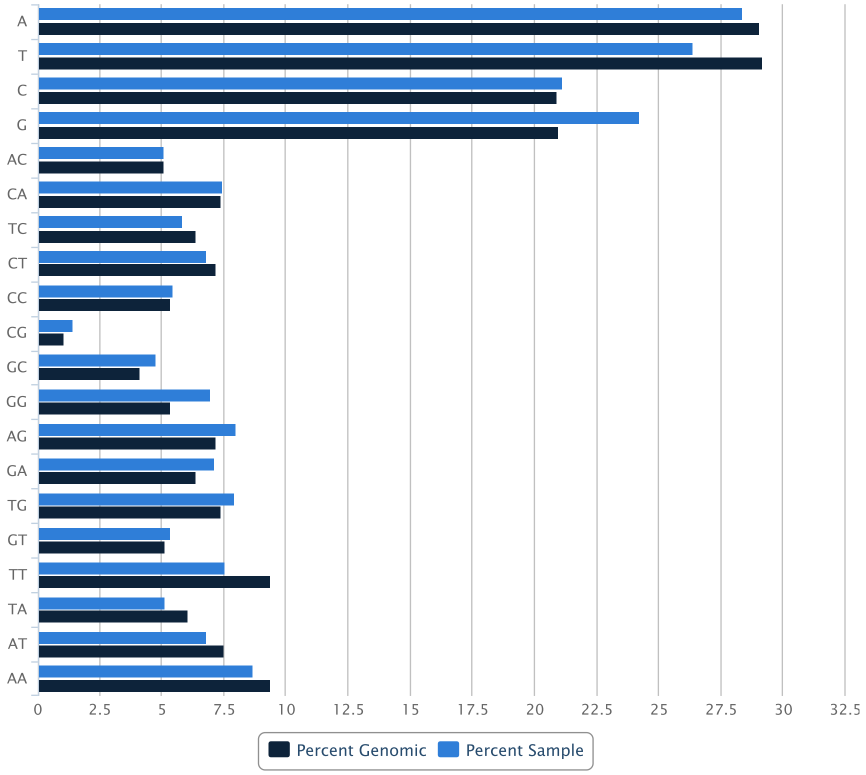

M-Bias Plot

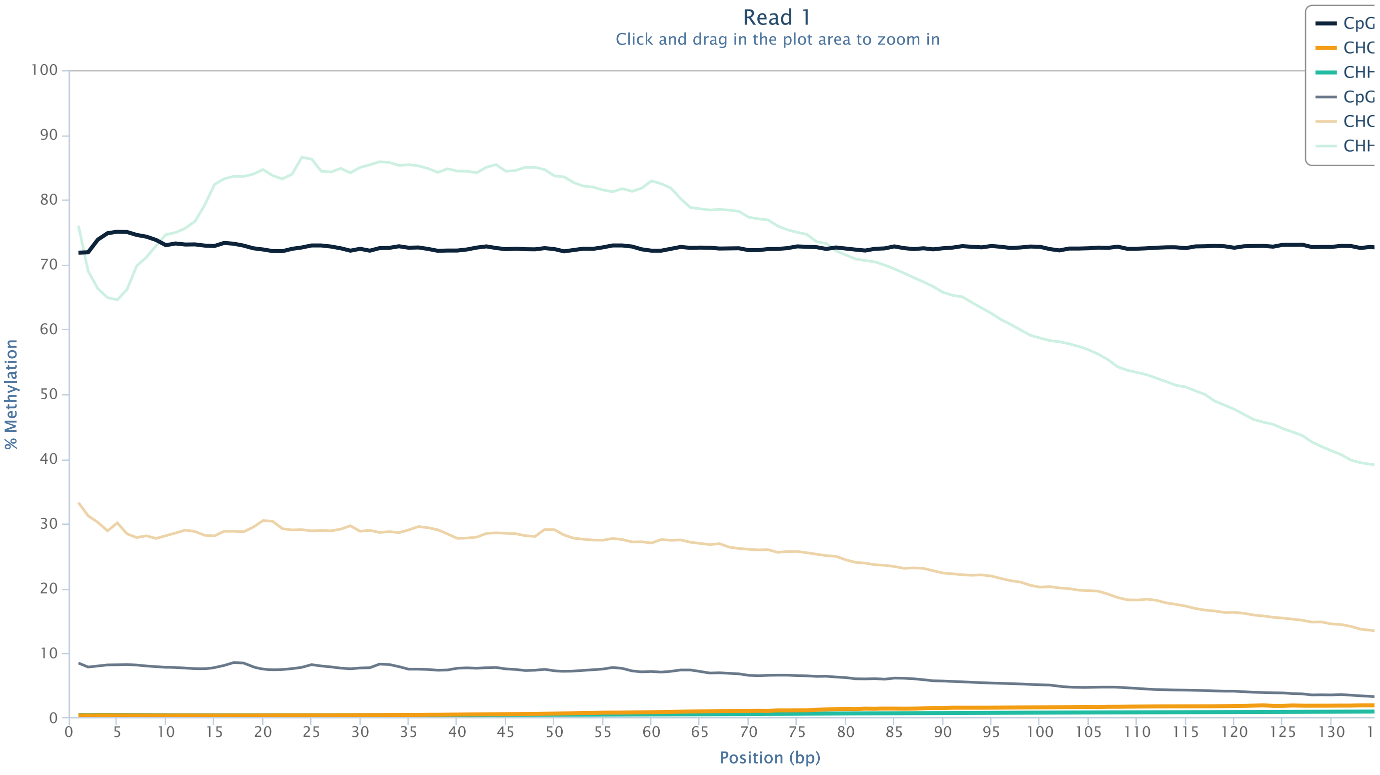

Analysis produced by **Bismark** (version v0.15.0) - a tool to map bisulfite converted sequence reads and determine cytosine methylation states

Report graphs rendered using jQuery and Highcharts. Page design by Phil Ewels.

Babraham Bioinformatics

Supplementary file 2. Mining of RNA-seq data for genes involved with DNA methylation and chromatin structure. Our previously published RNA-seq transcriptomics data was mined using 151 genes previously identified as being involved with histone modifications or DNA methylation. (see supplementary data file).

Supplementary file 3. Genes identified with intermediate methylation promoter patterns in bovine alveolar macrophages. Promoters were defined as regions spanning 1.5 kb upstream and 500 bp downstream of each transcription start site (TSS), with a minimum of 10 CpGs each associated with at least five methylation calls were included (26.8 million loci). Green box = region of the promoter analysed; Red line = infected; Blue line =control. Average methylation is represented on the y-axis (0.2 = 20%, 0.5 = 50% and 0.8 = 80%).

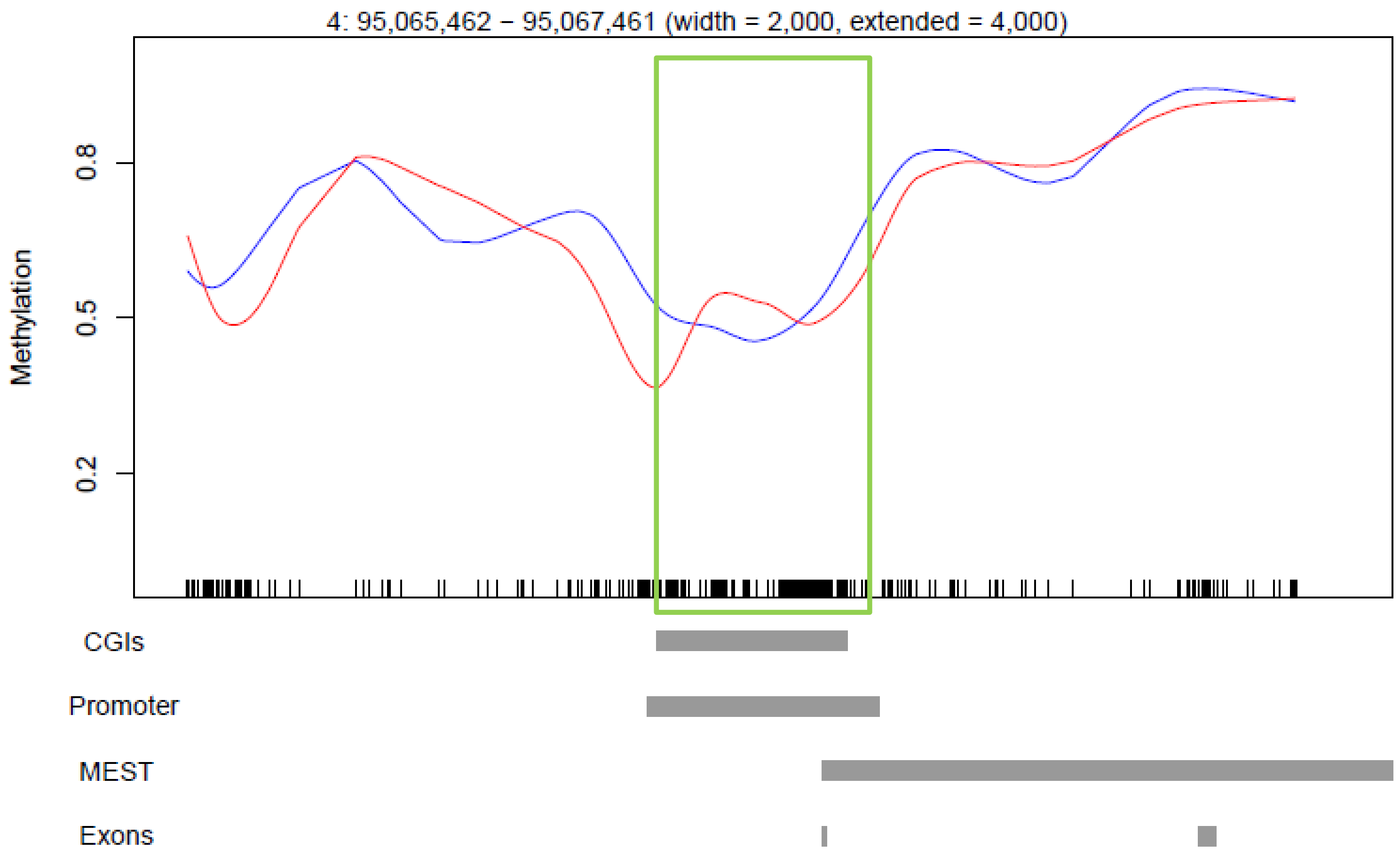
4: 95,065,462 – 95,067,461 (width = 2,000, extended = 4,000)
Methylation
0.8
0.5
0.2
CGIs
Promoter
MEST
Exons

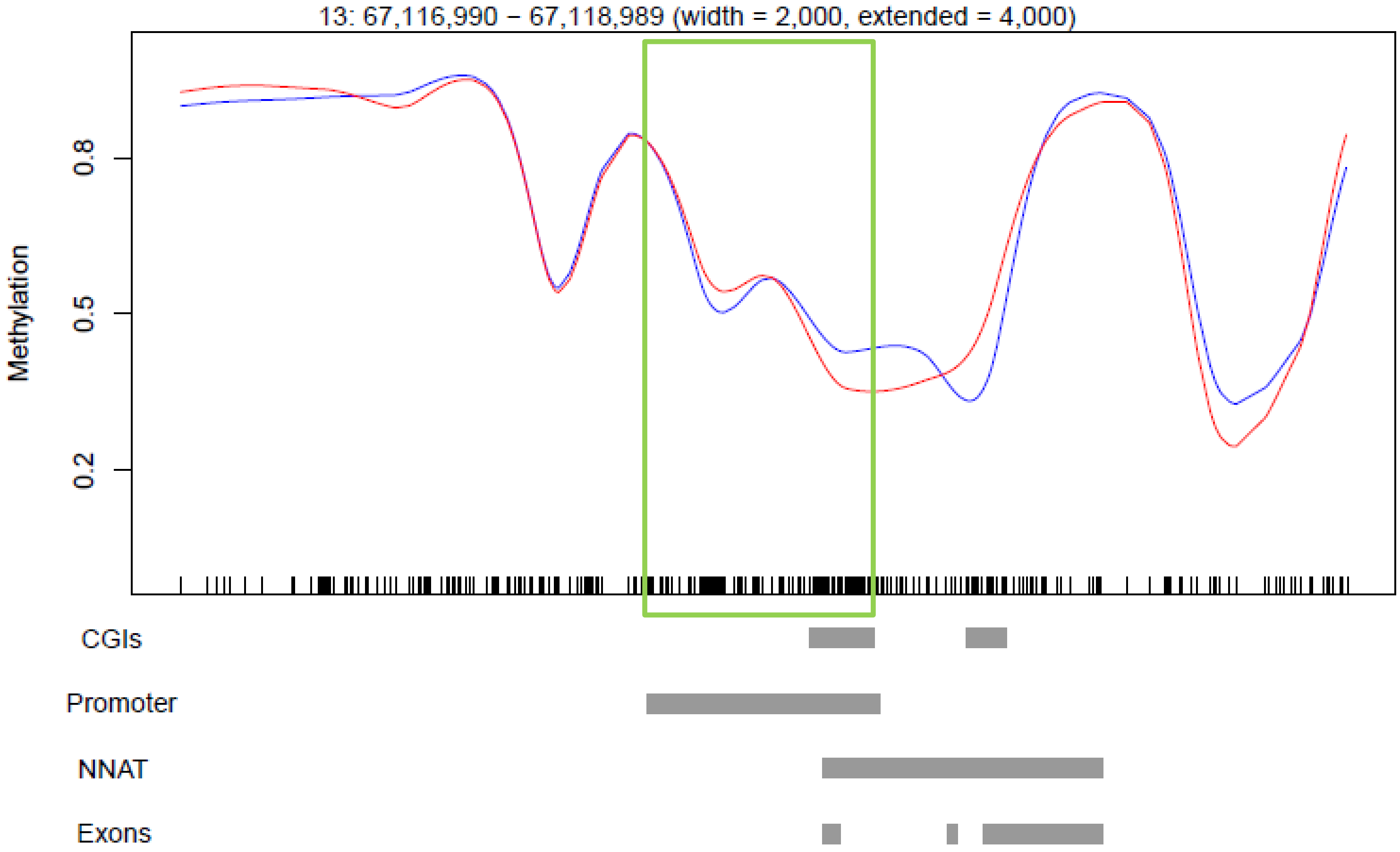

13: 67,116,990 – 67,118,989 (width = 2,000, extended = 4,000)
Methylation
0.8
0.5
0.2
CGIs
Promoter
NNAT
Exons

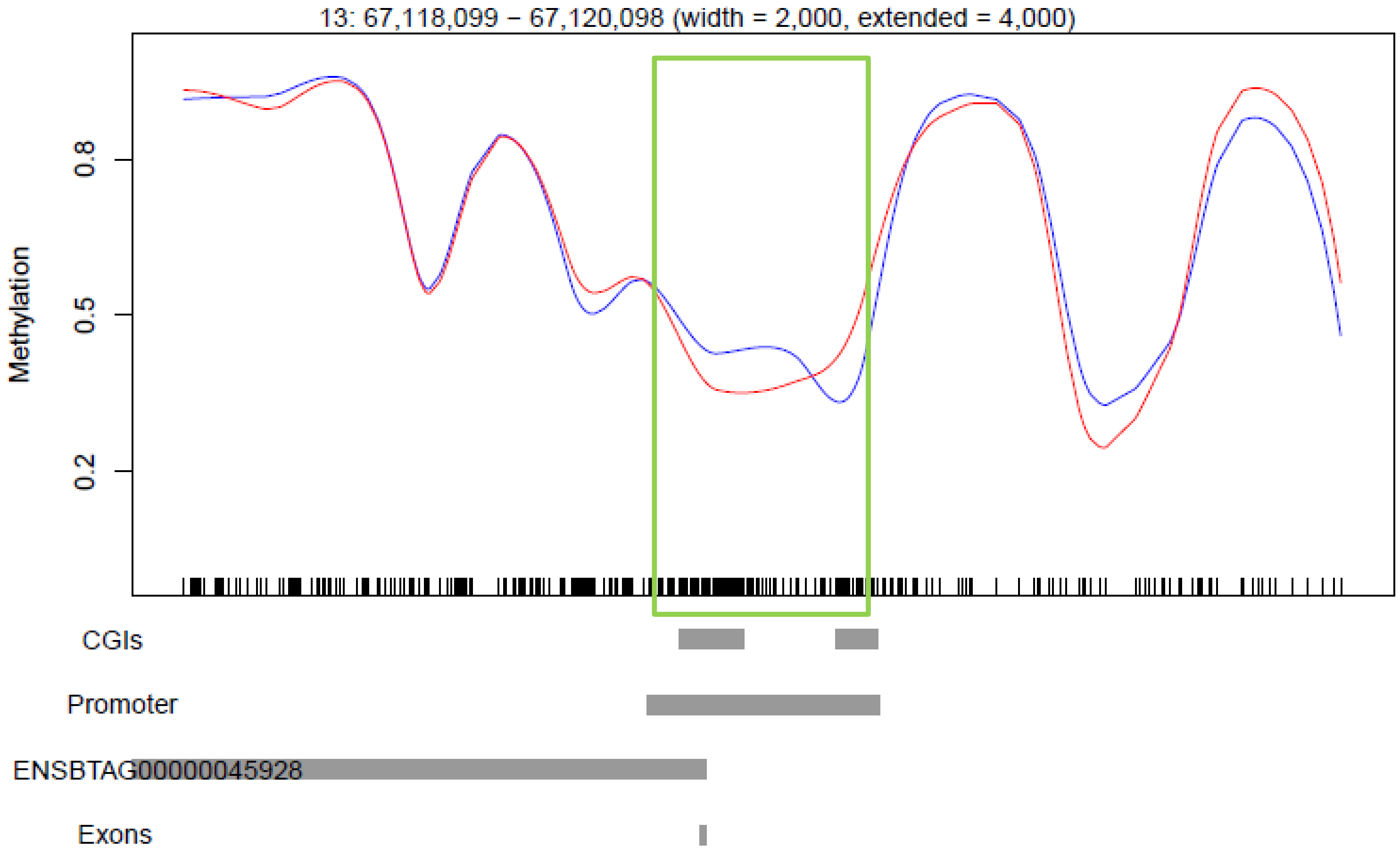

13: 67,118,099 − 67,120,098 (width = 2,000, extended = 4,000)
Methylation
0.8
0.5
0.2
CGIs
Promoter
ENSBTAG00000045928
Exons

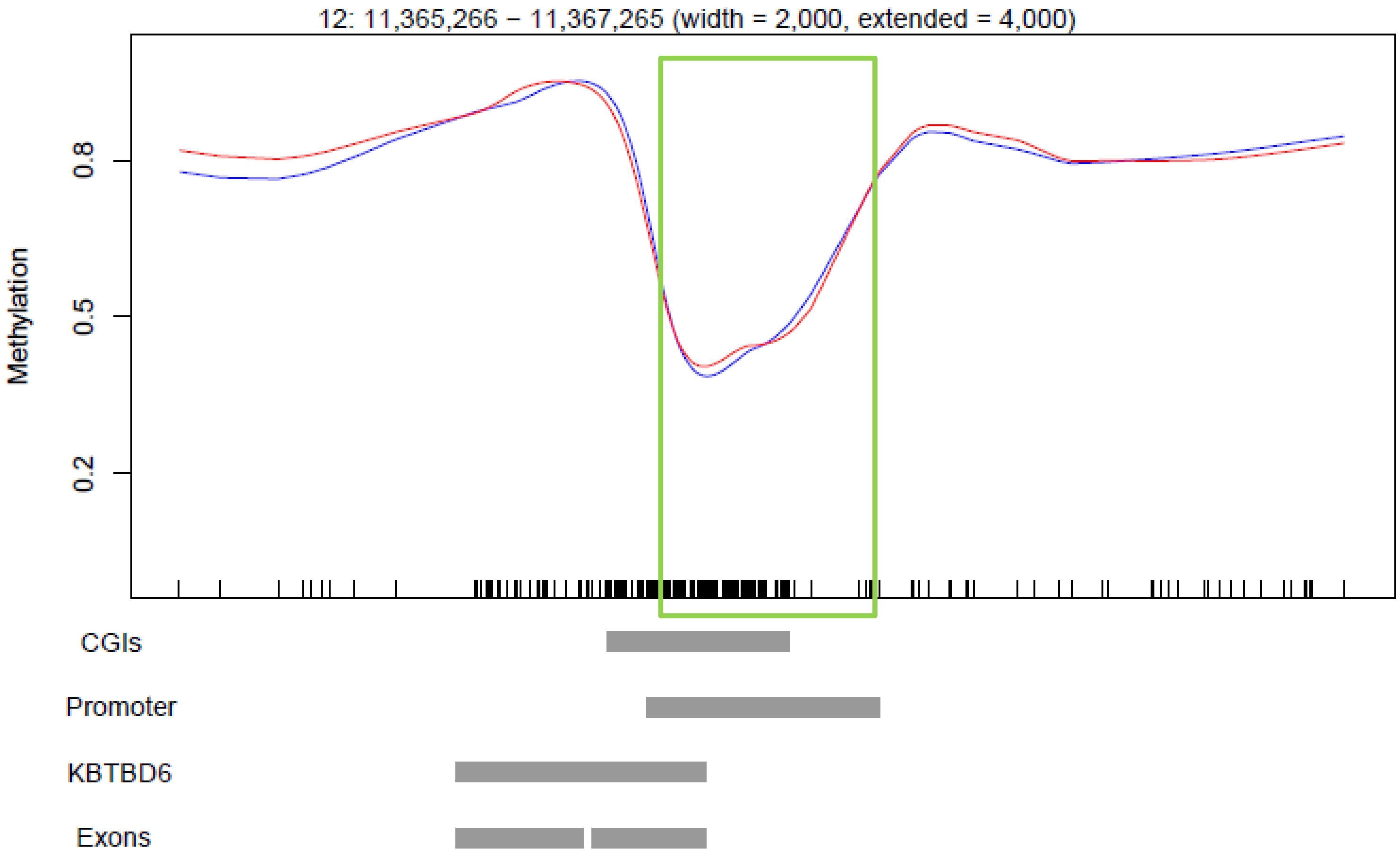
12: 11,365,266 − 11,367,265 (width = 2,000, extended = 4,000)
Methylation
0.8
0.5
0.2
CGIs
Promoter
KBTBD6
Exons

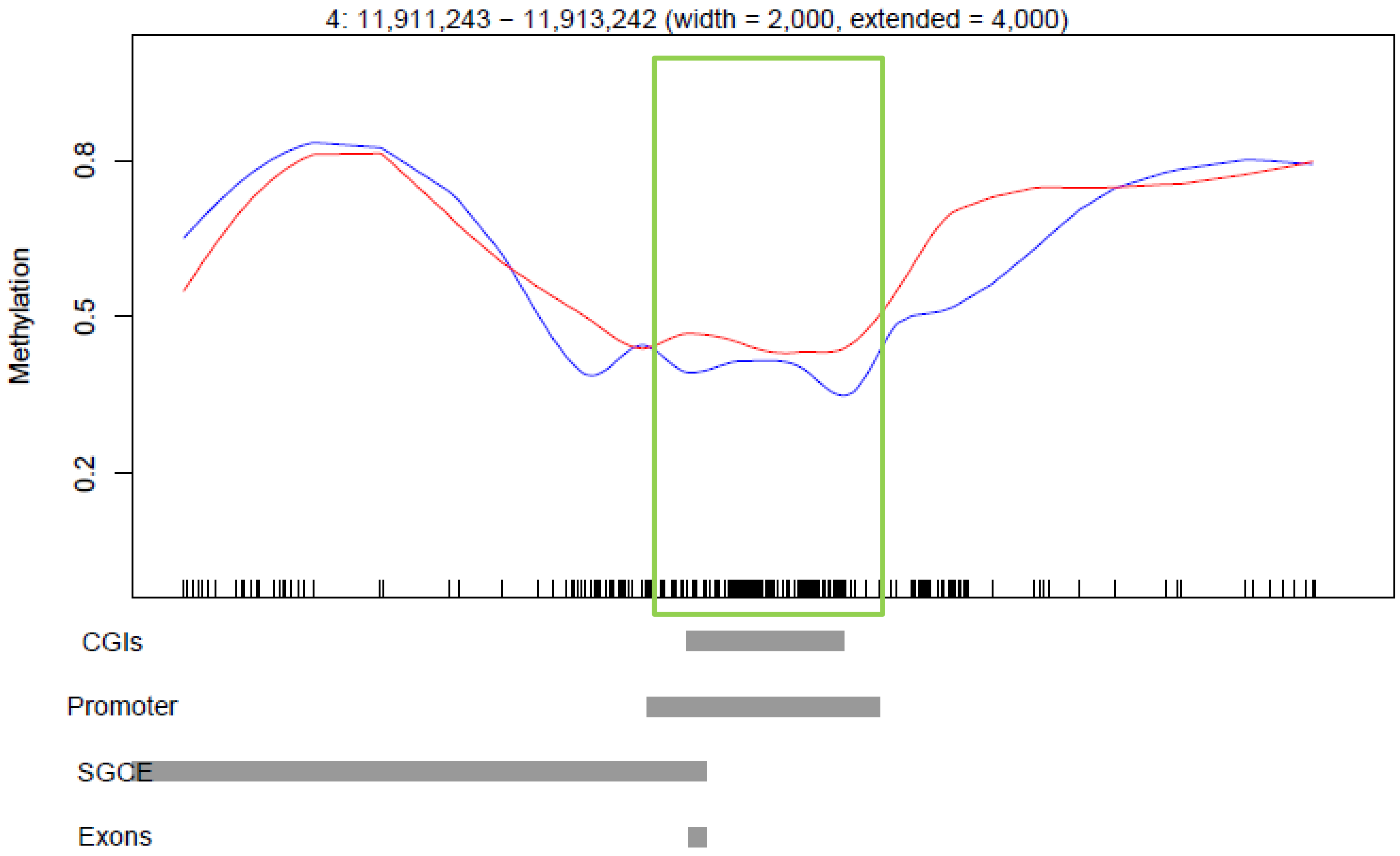
4: 11,911,243 − 11,913,242 (width = 2,000, extended = 4,000)
Methylation
0.8
0.5
0.2
CGIs
Promoter
SGCE
Exons

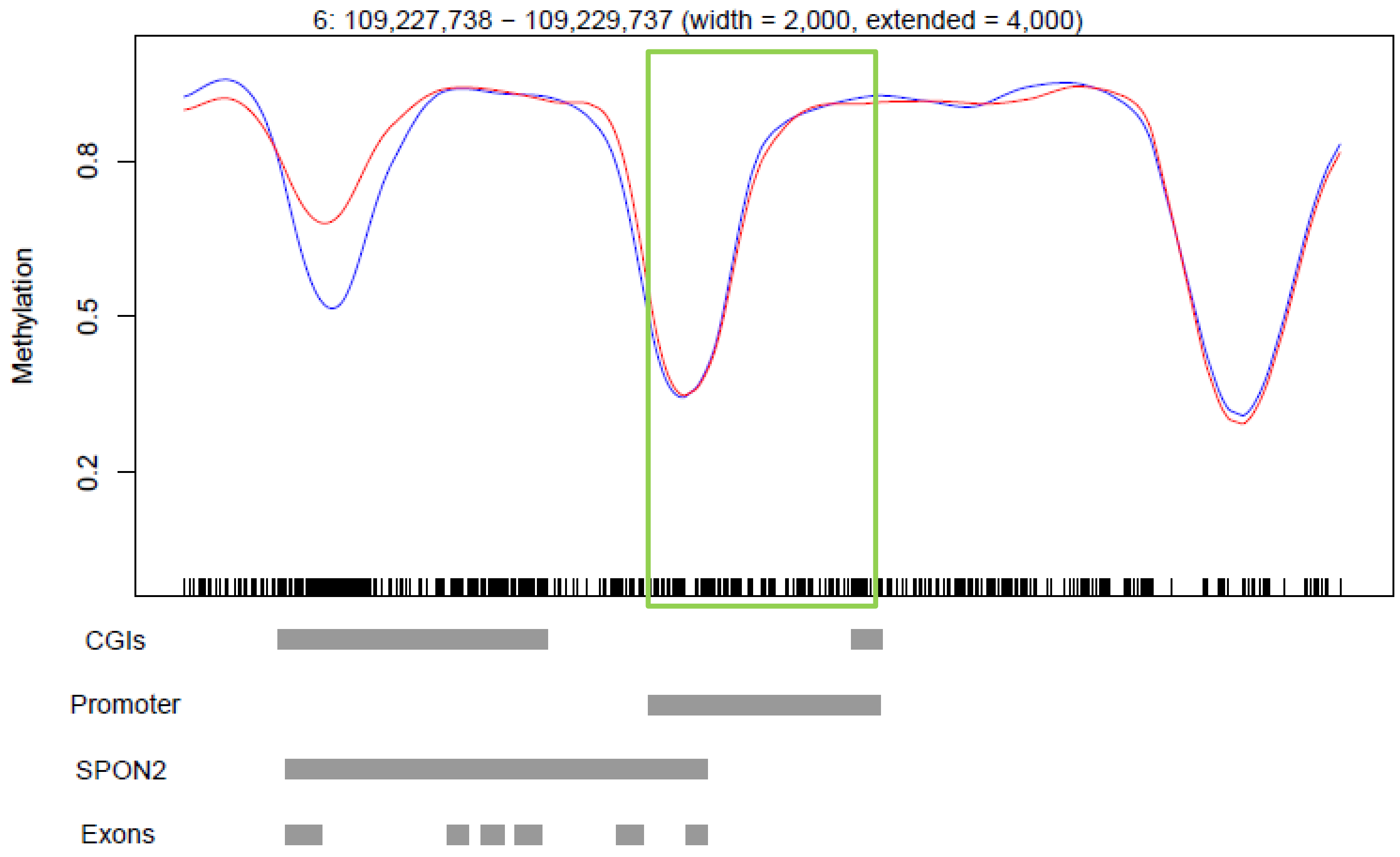

6: 109,227,738 − 109,229,737 (width = 2,000, extended = 4,000)
Methylation
0.8
0.5
0.2
CGIs
Promoter
SPON2
Exons

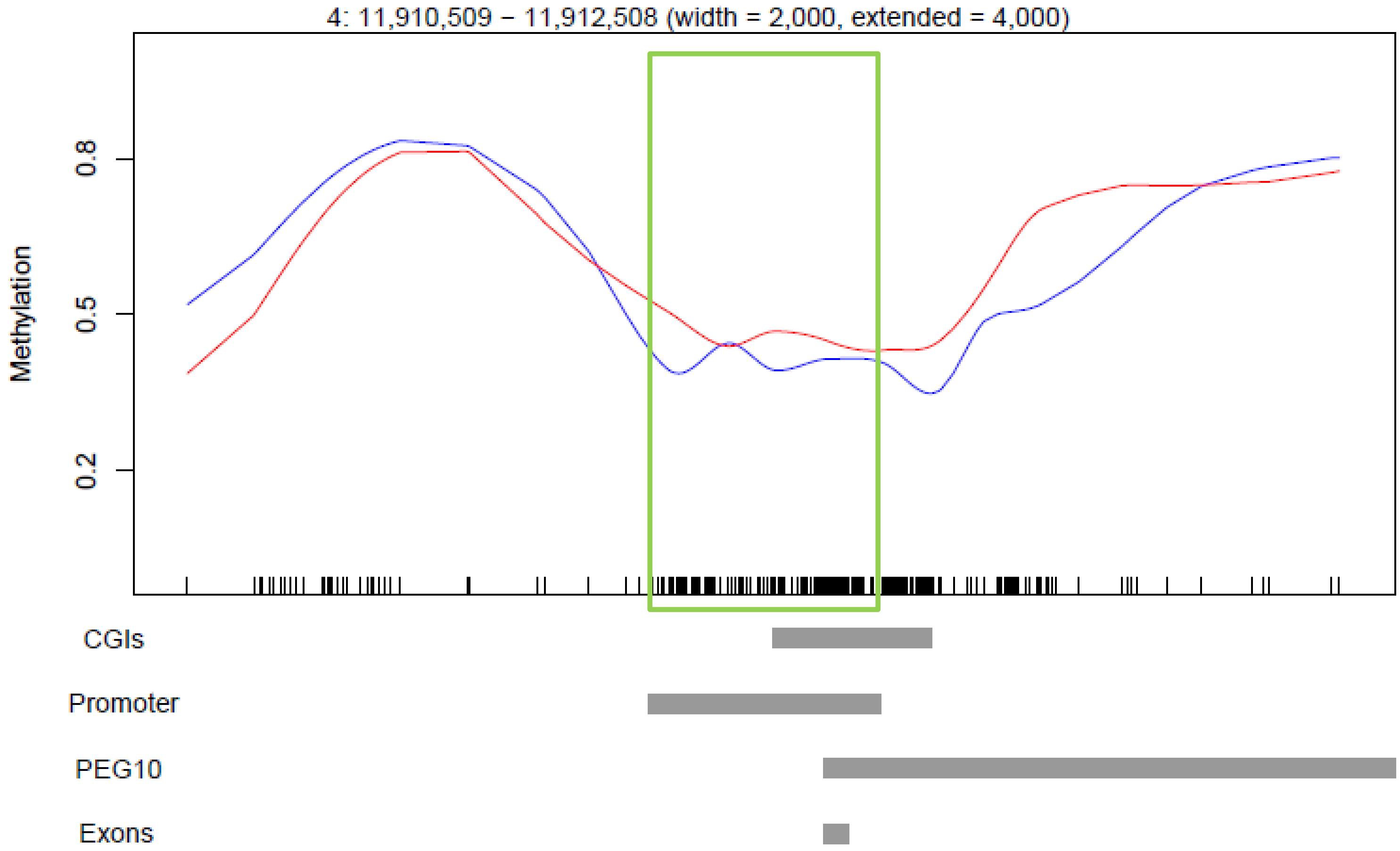

4: 11,910,509 − 11,912,508 (width = 2,000, extended = 4,000)
Methylation
0.8
0.5
0.2
CGIs
Promoter
PEG10
Exons

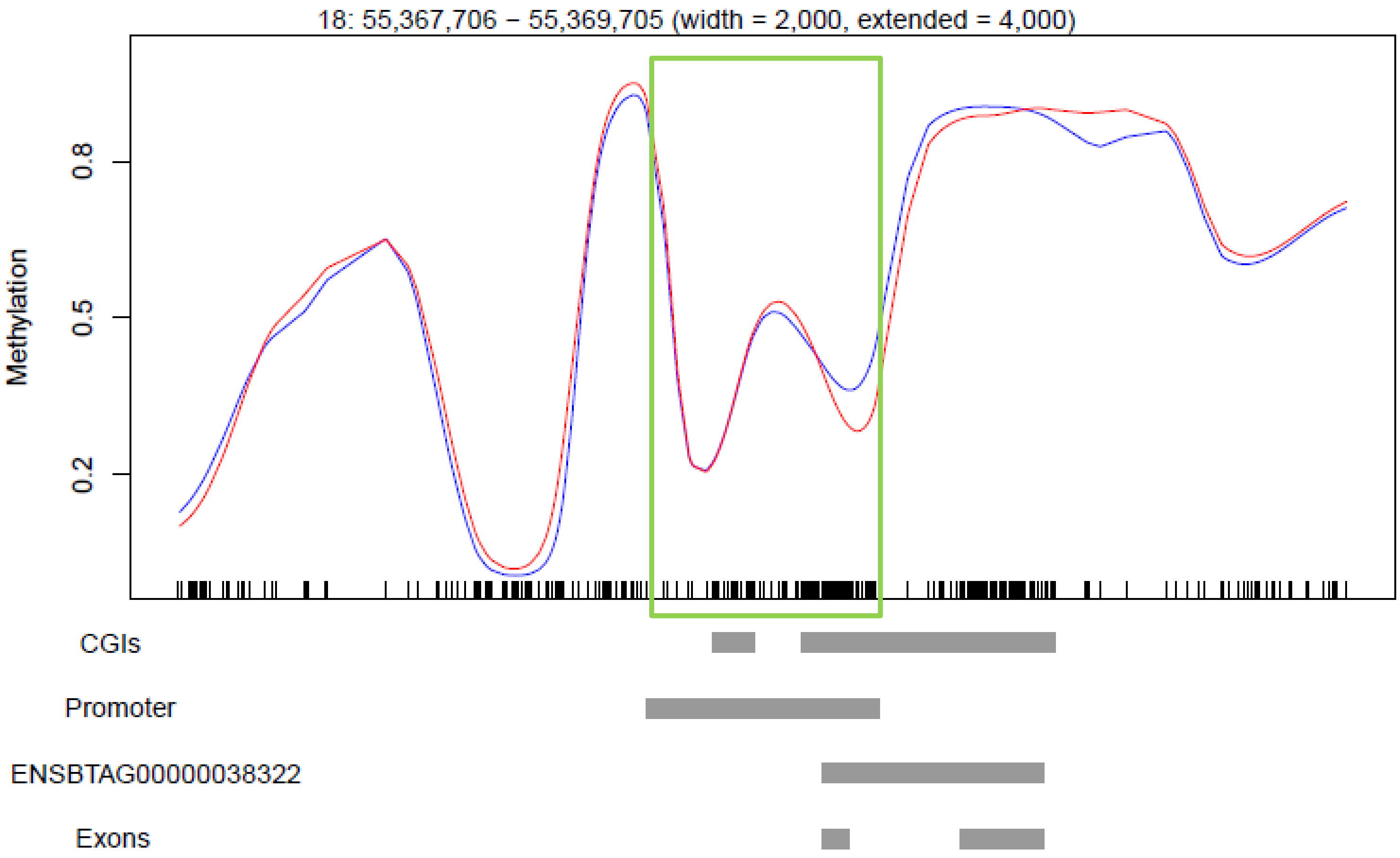
18: 55,367,706 – 55,369,705 (width = 2,000, extended = 4,000)
Methylation
0.2
0.5
0.8
CGIs
Promoter
ENSBTAG00000038322
Exons

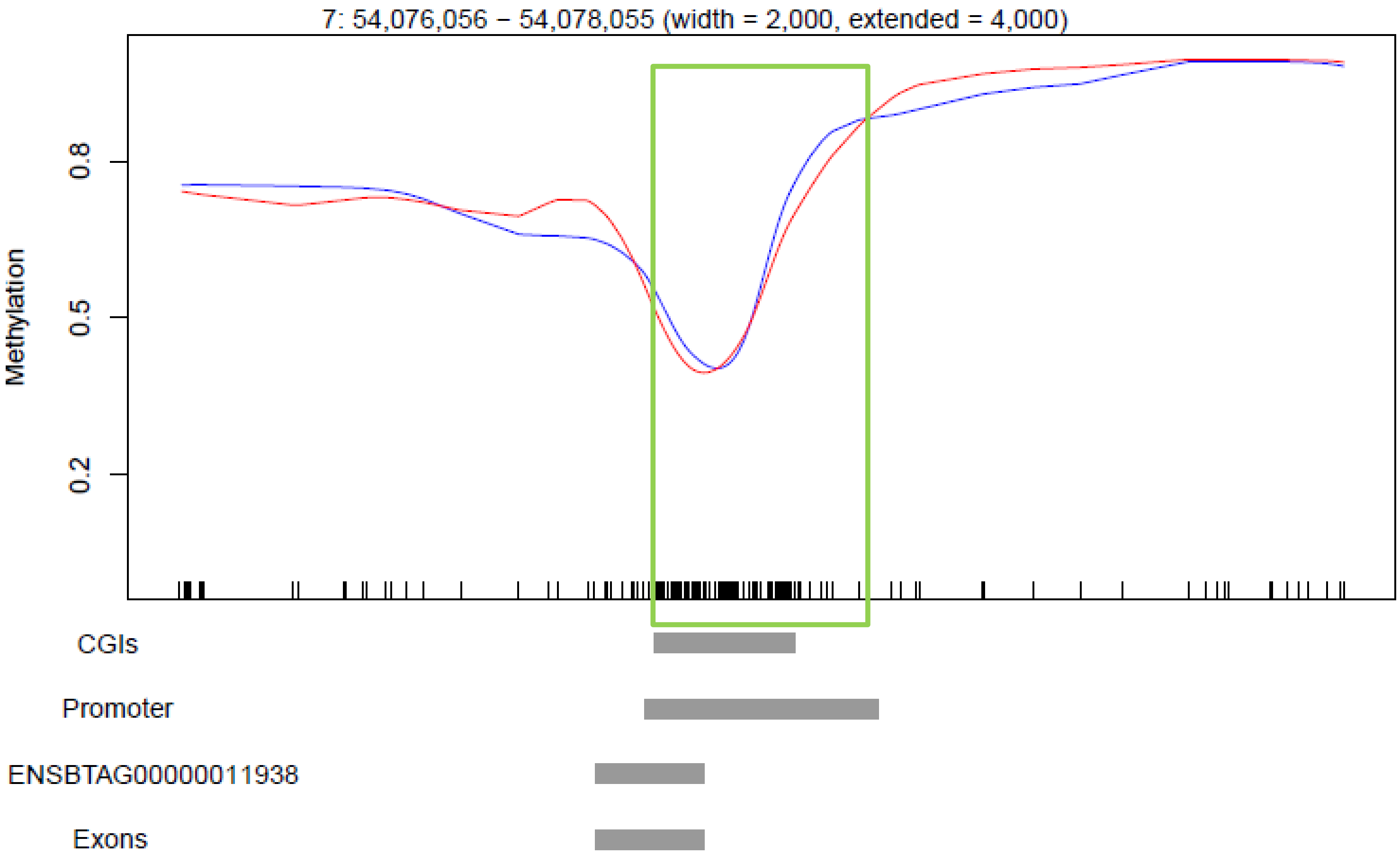

7: 54,076,056 – 54,078,055 (width = 2,000, extended = 4,000)
Methylation
0.8
0.5
0.2
CGIs
Promoter
ENSBTAG00000011938
Exons

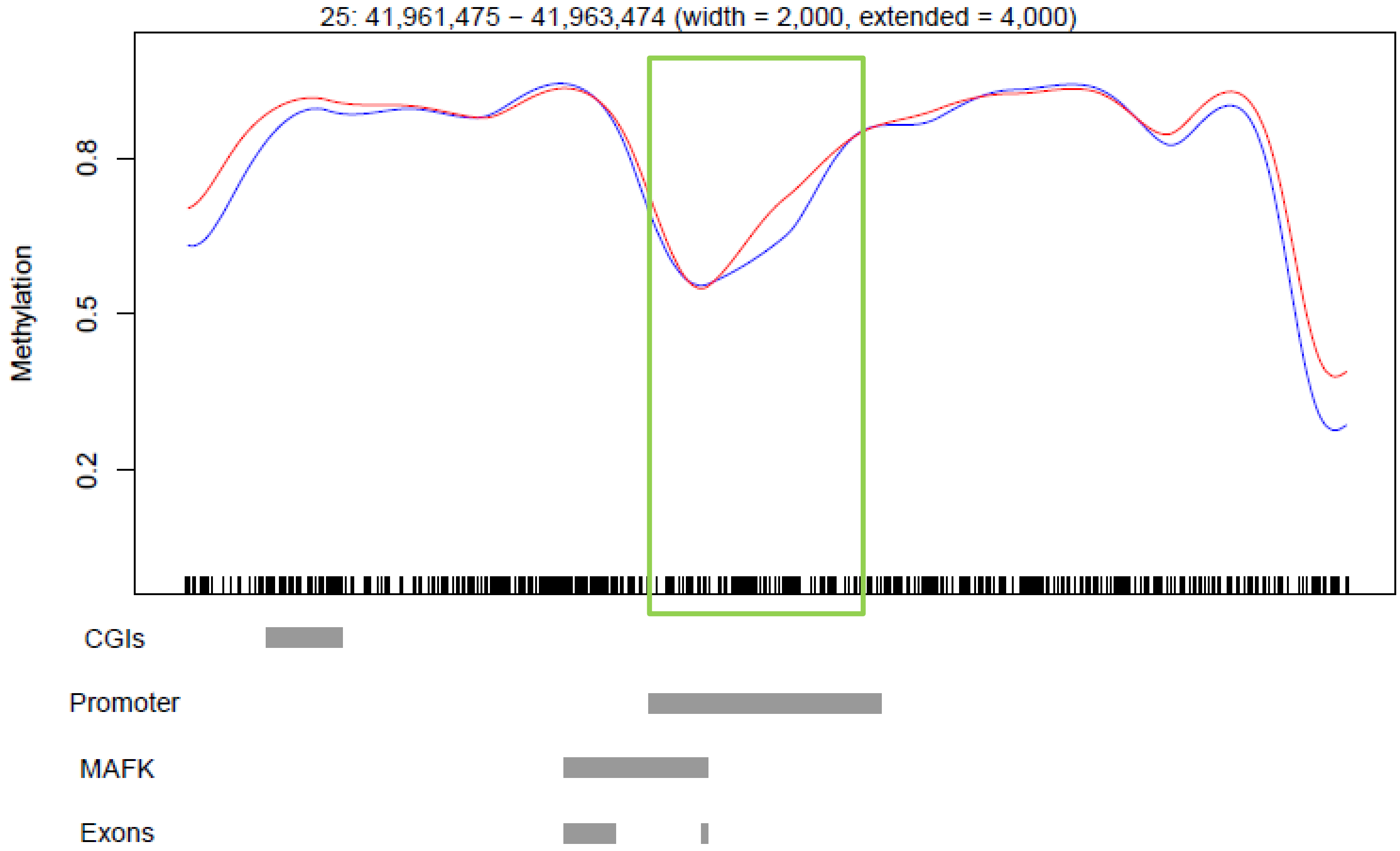
25: 41,961,475 − 41,963,474 (width = 2,000, extended = 4,000)
Methylation
0.8
0.5
0.2
CGIs
Promoter
MAFK
Exons

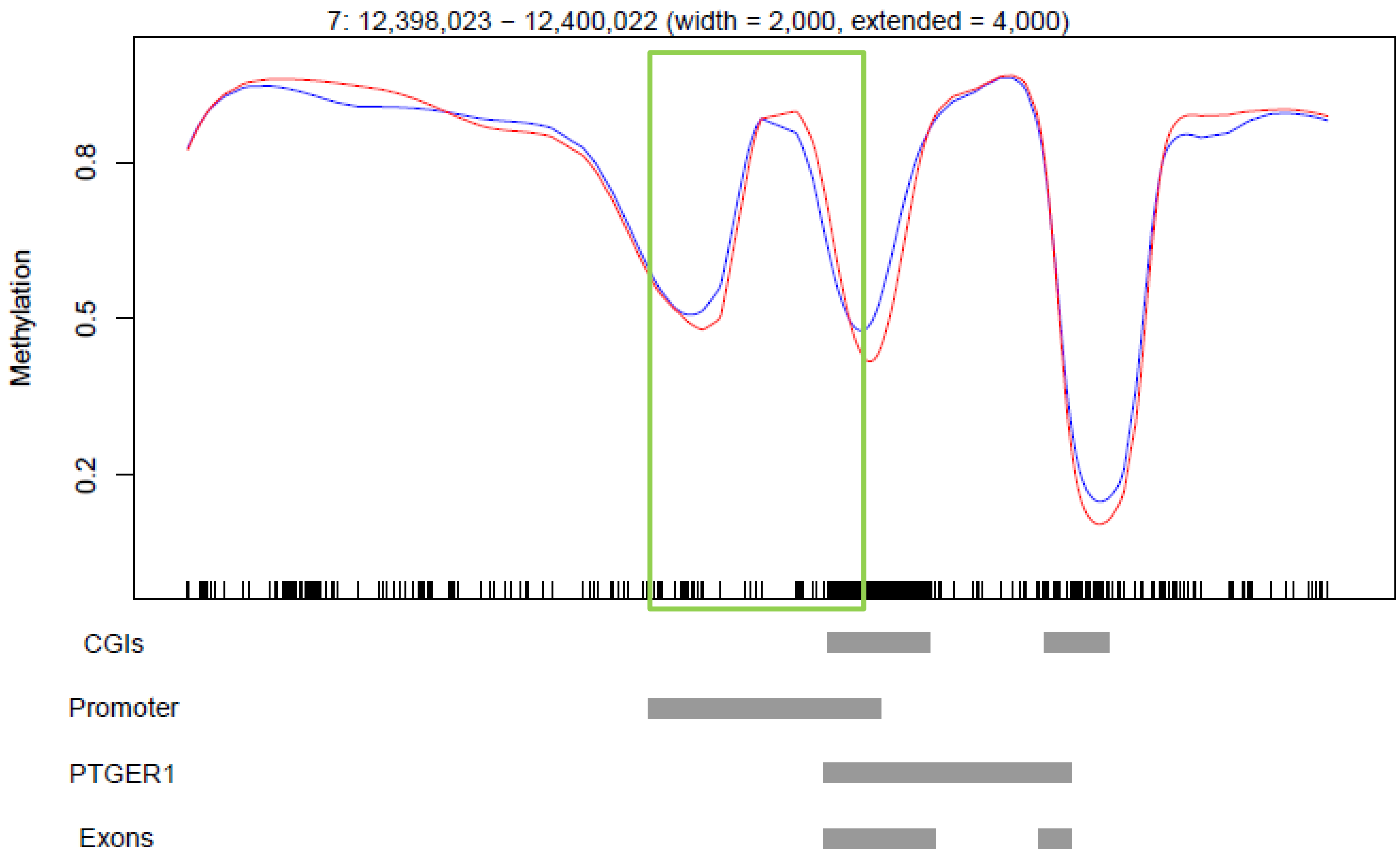
7: 12,398,023 – 12,400,022 (width = 2,000, extended = 4,000)
Methylation
0.8
0.5
0.2
CGIs
Promoter
PTGER1
Exons

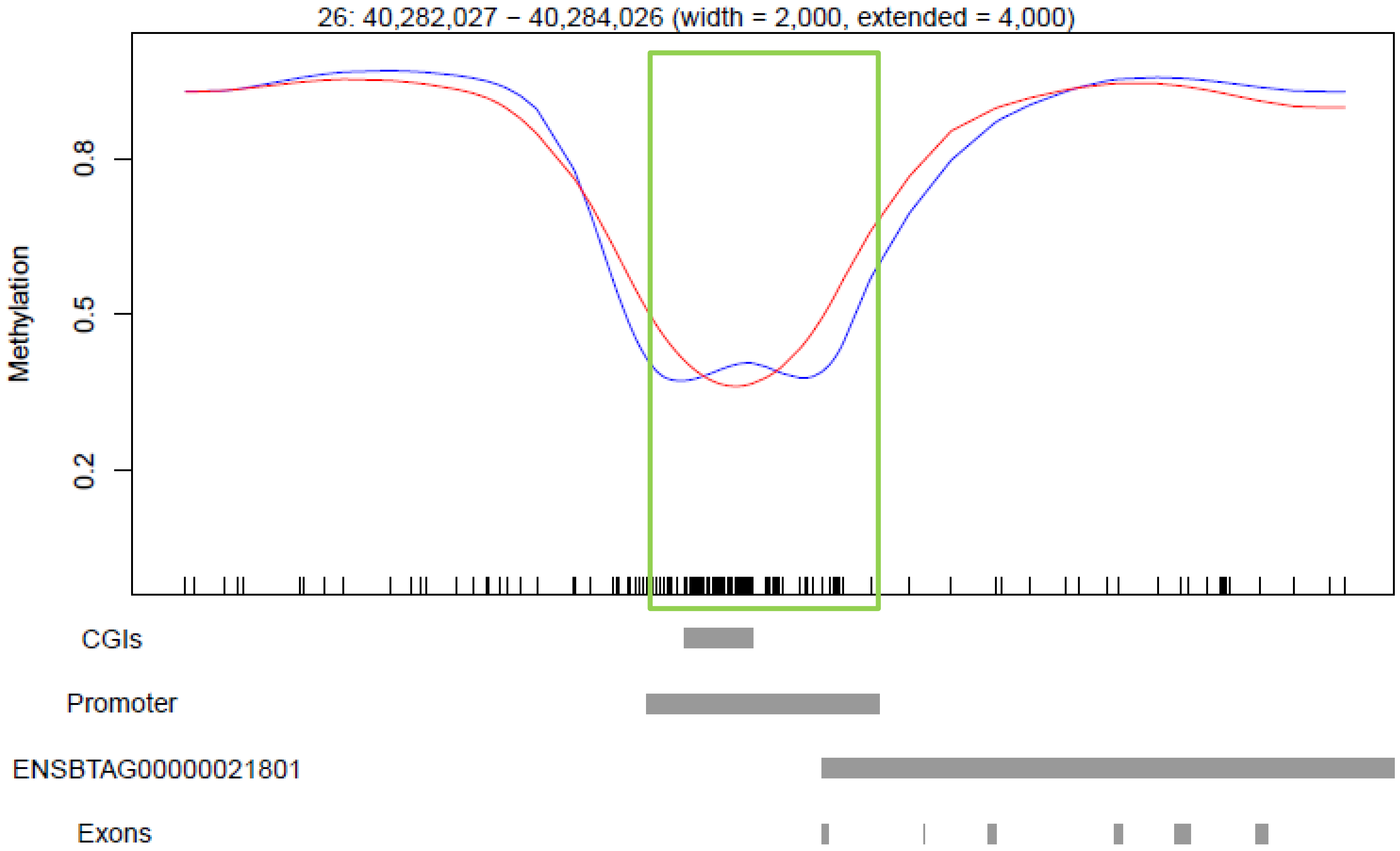

26: 40,282,027 − 40,284,026 (width = 2,000, extended = 4,000)
Methylation
0.8
0.5
0.2
CGIs
Promoter
ENSBTAG00000021801
Exons

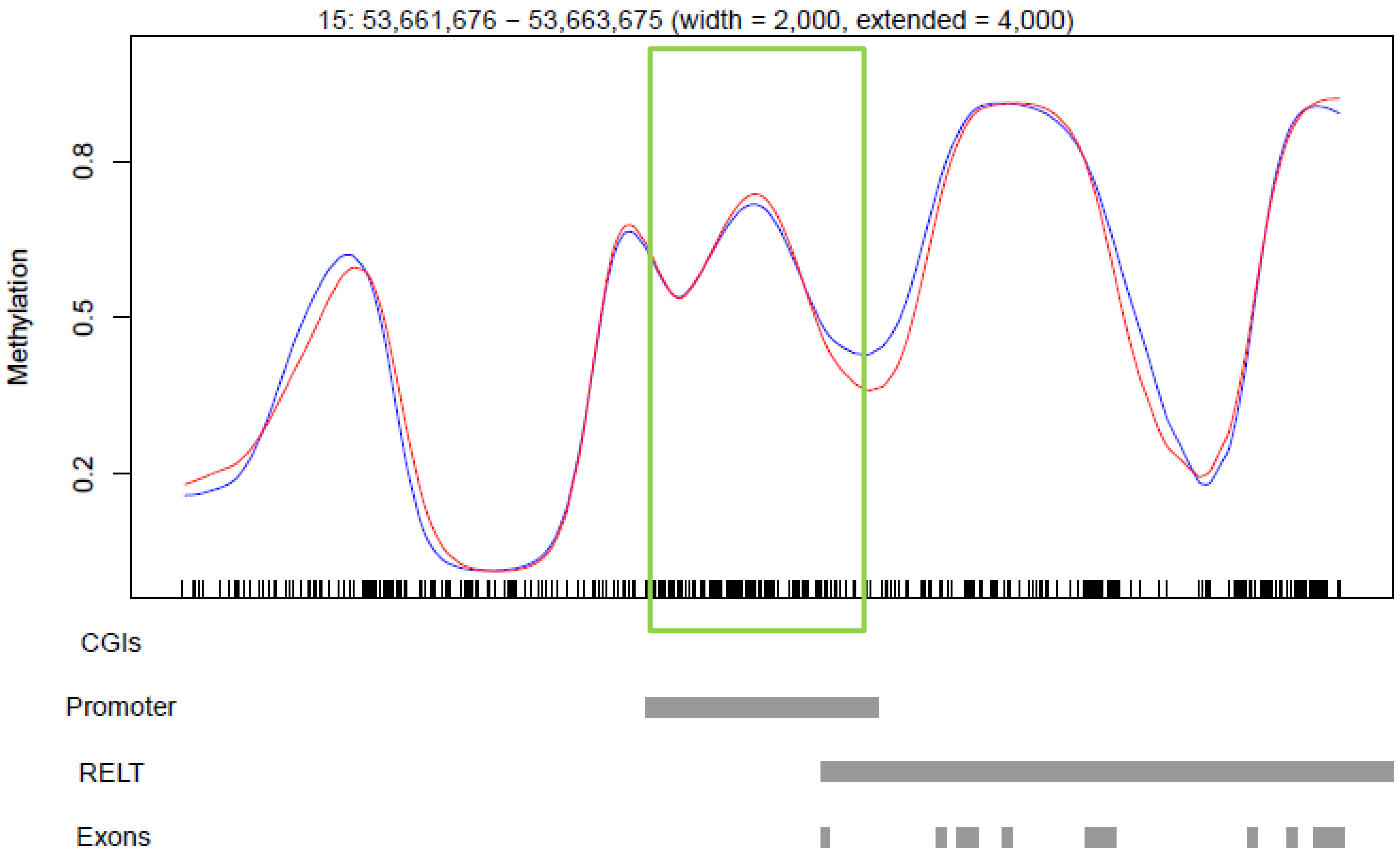
15: 53,661,676 − 53,663,675 (width = 2,000, extended = 4,000)
Methylation
0.2
0.5
0.8
CGIs
Promoter
RELT
Exons

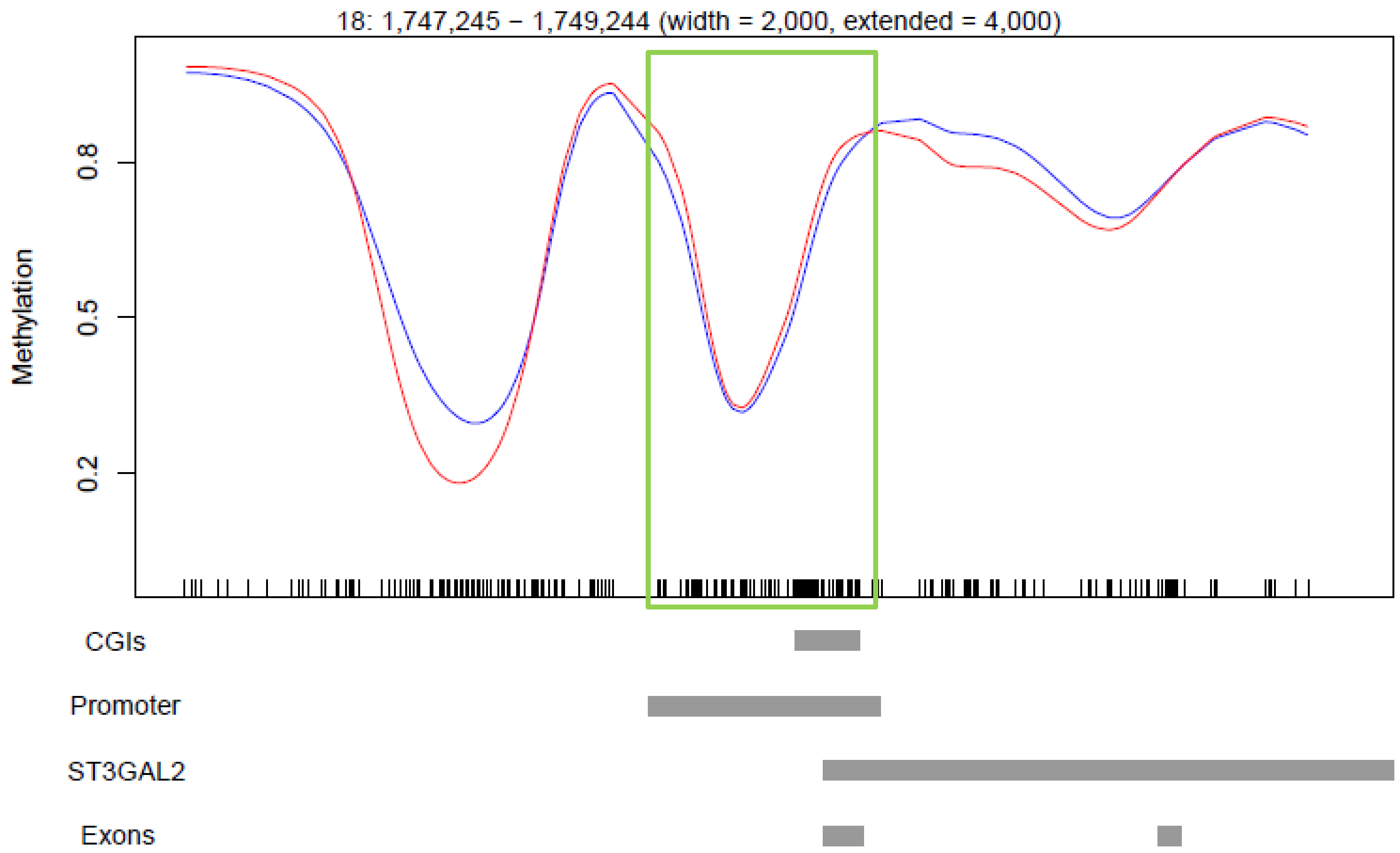

18: 1,747,245 – 1,749,244 (width = 2,000, extended = 4,000)
Methylation
0.8
0.5
0.2
CGIs
Promoter
ST3GAL2
Exons

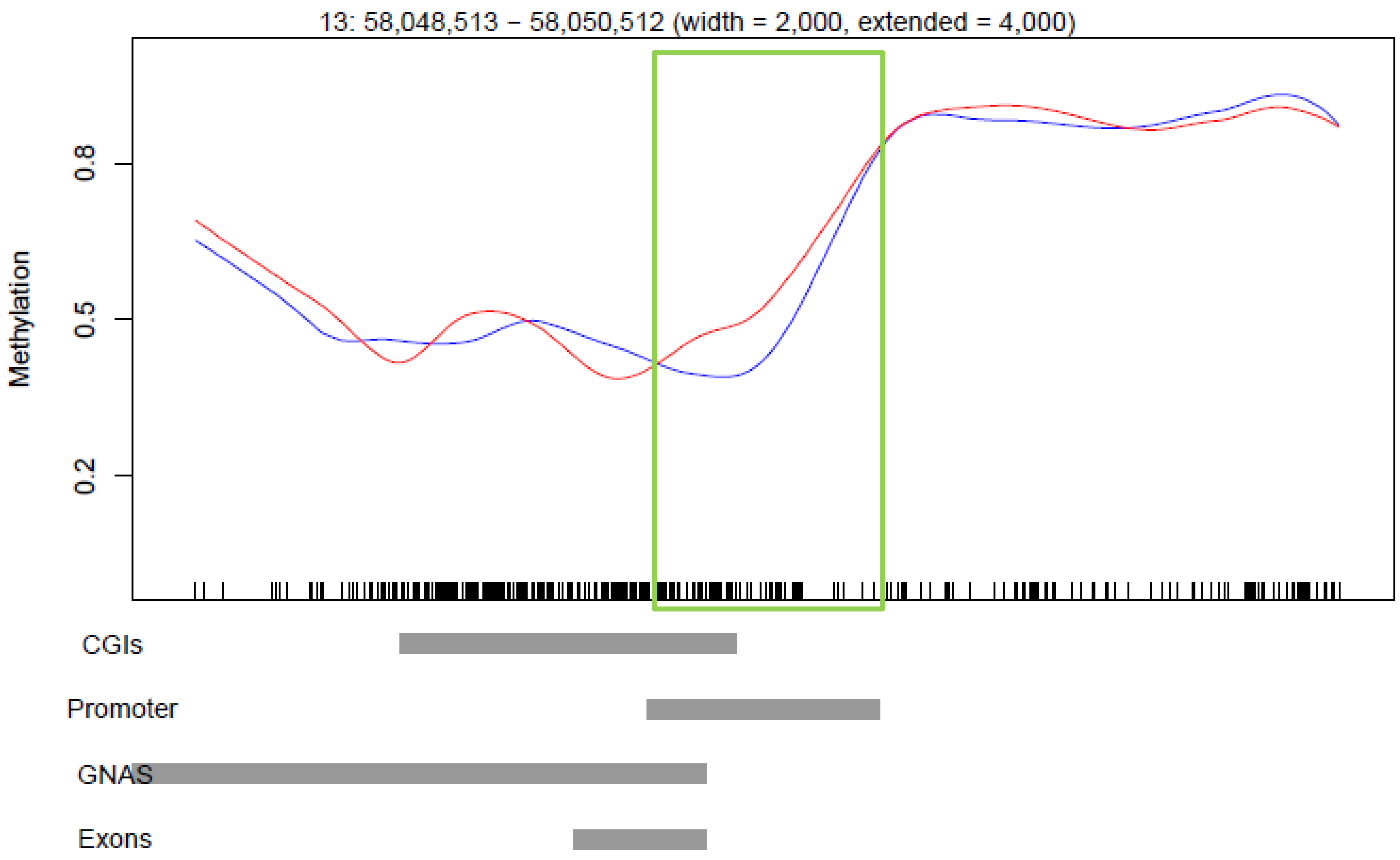

13: 58,048,513 − 58,050,512 (width = 2,000, extended = 4,000)
Methylation
0.2
0.5
0.8
CGIs
Promoter
GNAS
Exons

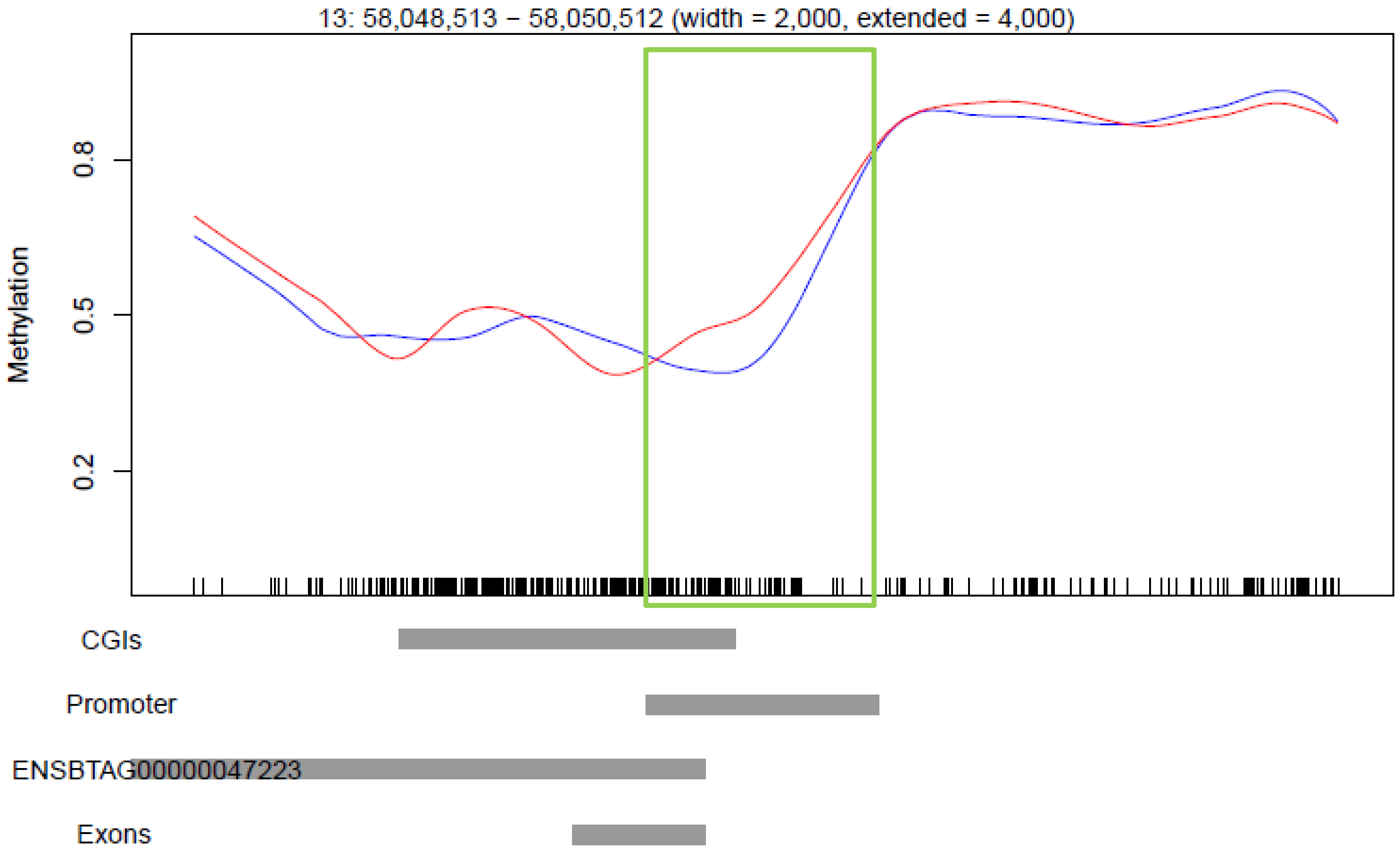
13: 58,048,513 − 58,050,512 (width = 2,000, extended = 4,000)
Methylation
0.2
0.5
0.8
CGIs
Promoter
ENSBTAG00000047223
Exons

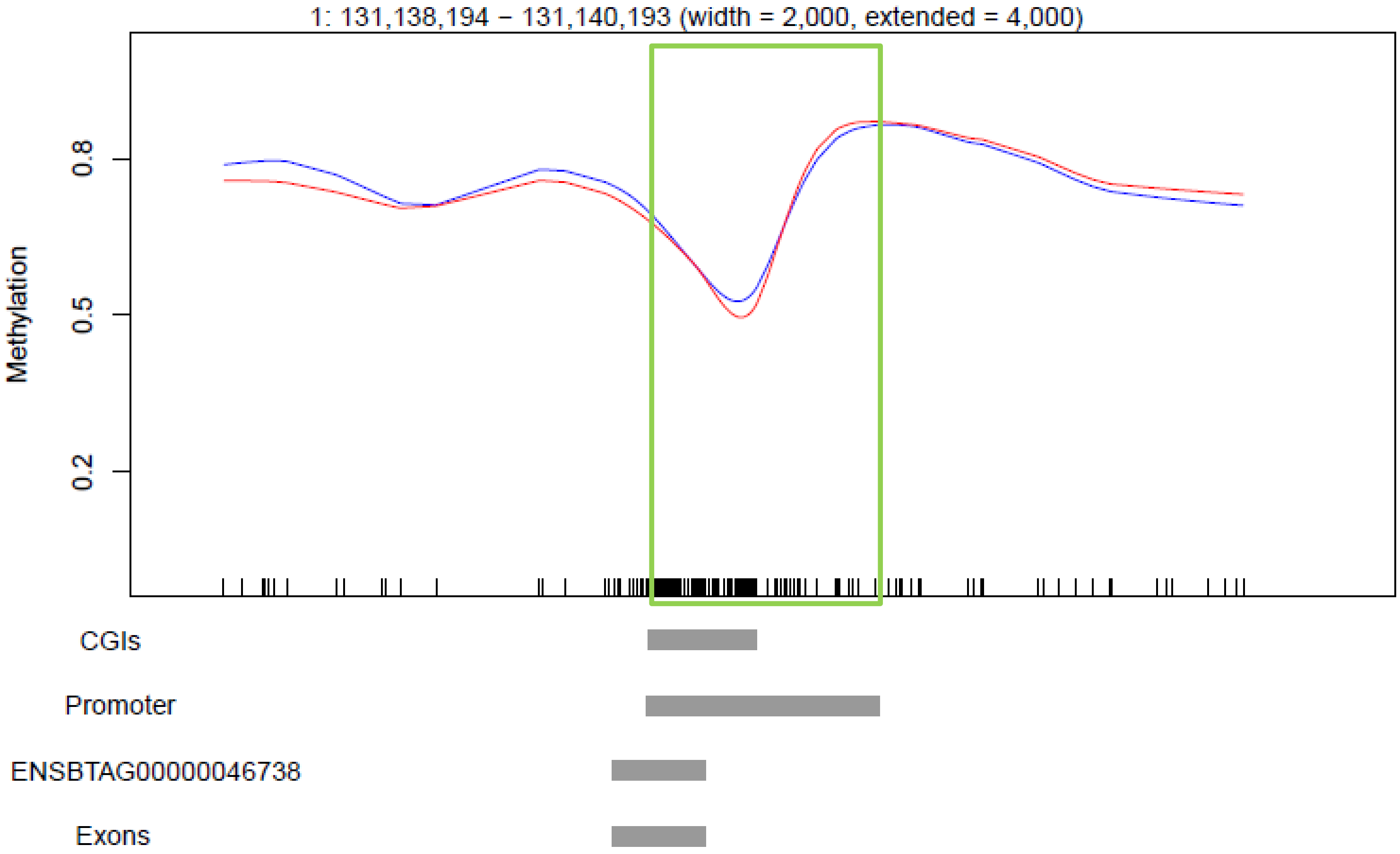
1: 131,138,194 − 131,140,193 (width = 2,000, extended = 4,000)
Methylation
0.2
0.5
0.8
CGIs
Promoter
ENSBTAG00000046738
Exons

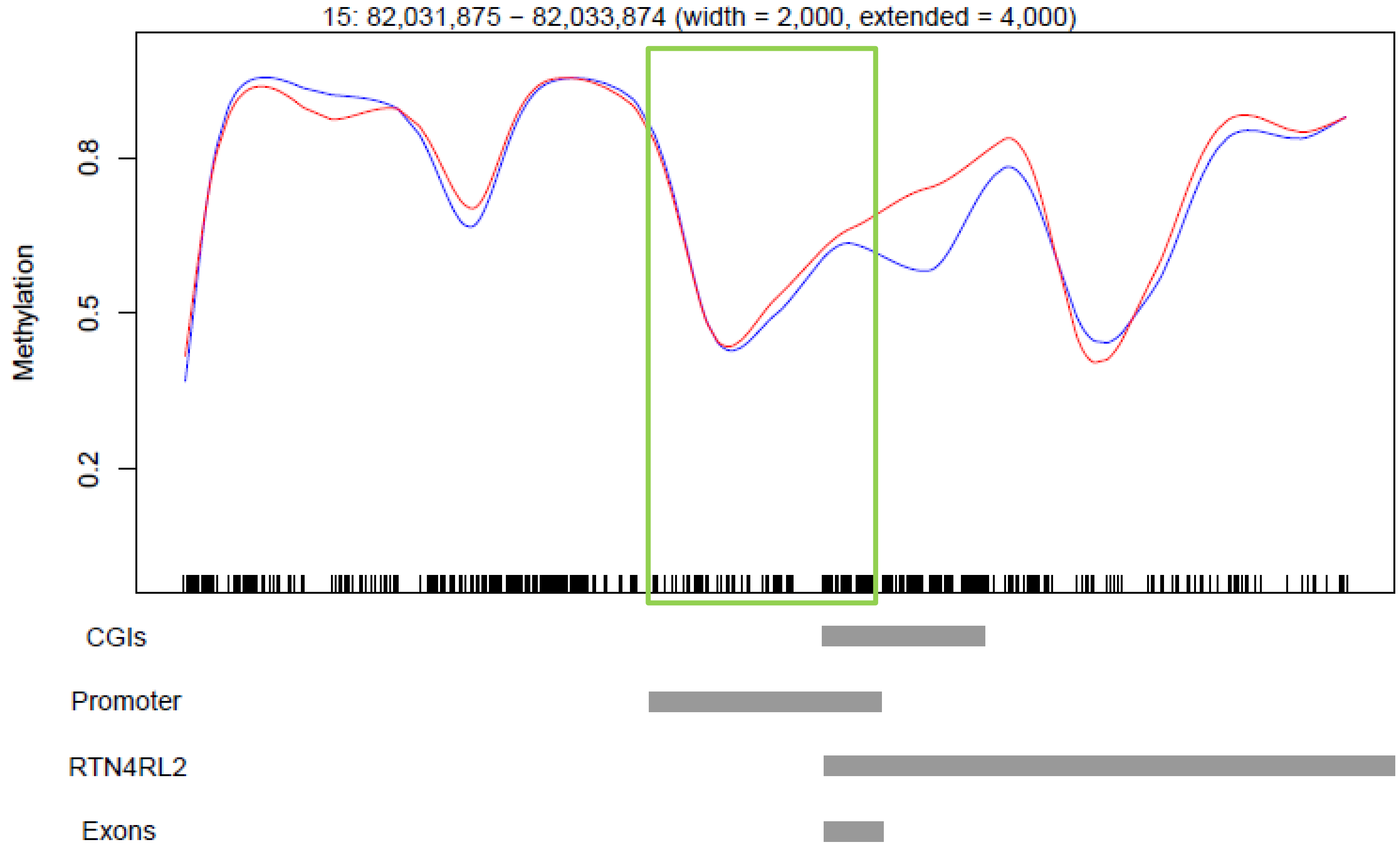
15: 82,031,875 − 82,033,874 (width = 2,000, extended = 4,000)
Methylation
0.8
0.5
0.2
CGIs
Promoter
RTN4RL2
Exons

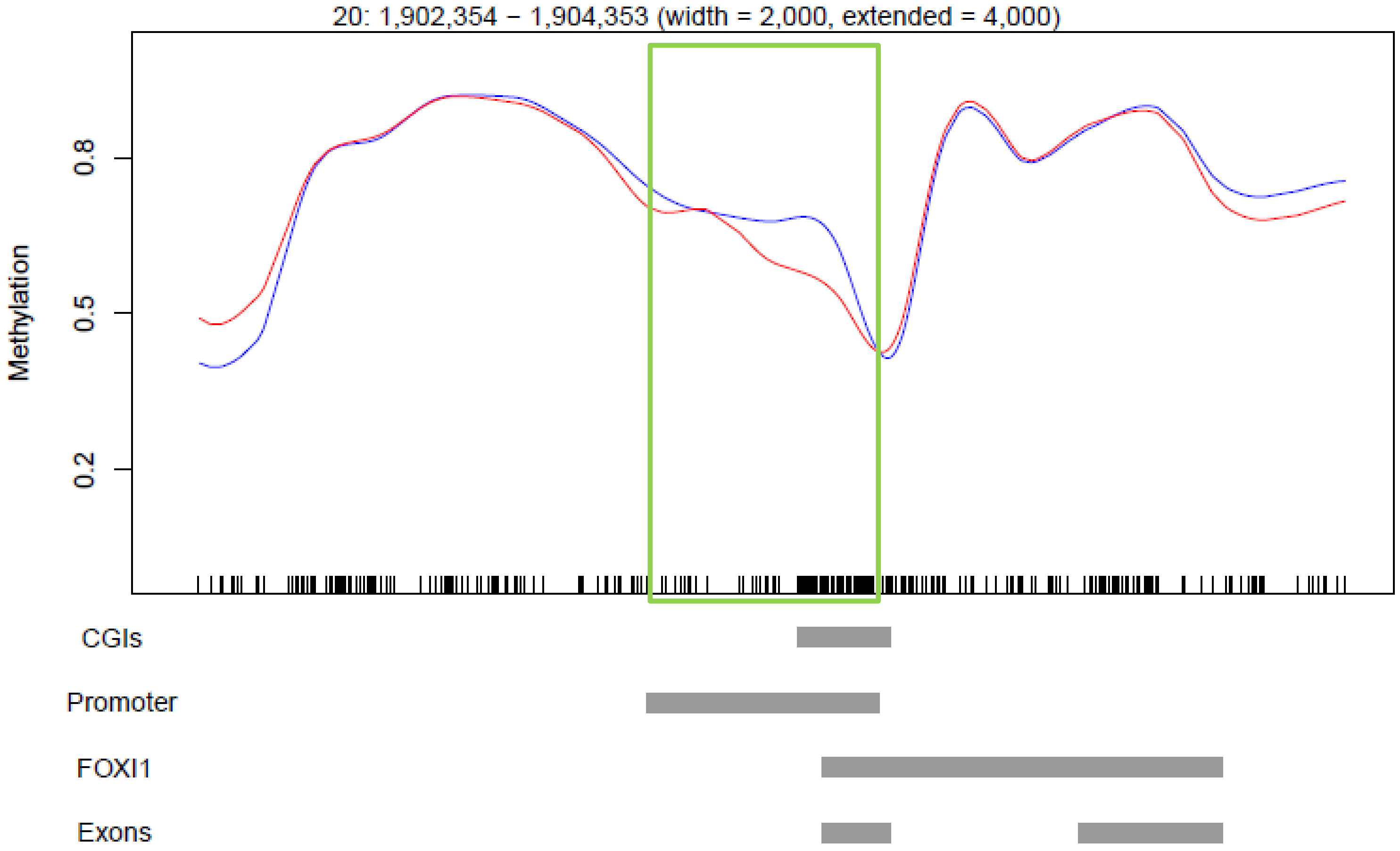

20: 1,902,354 − 1,904,353 (width = 2,000, extended = 4,000)
Methylation
0.8
0.5
0.2
CGIs
Promoter
FOXI1
Exons

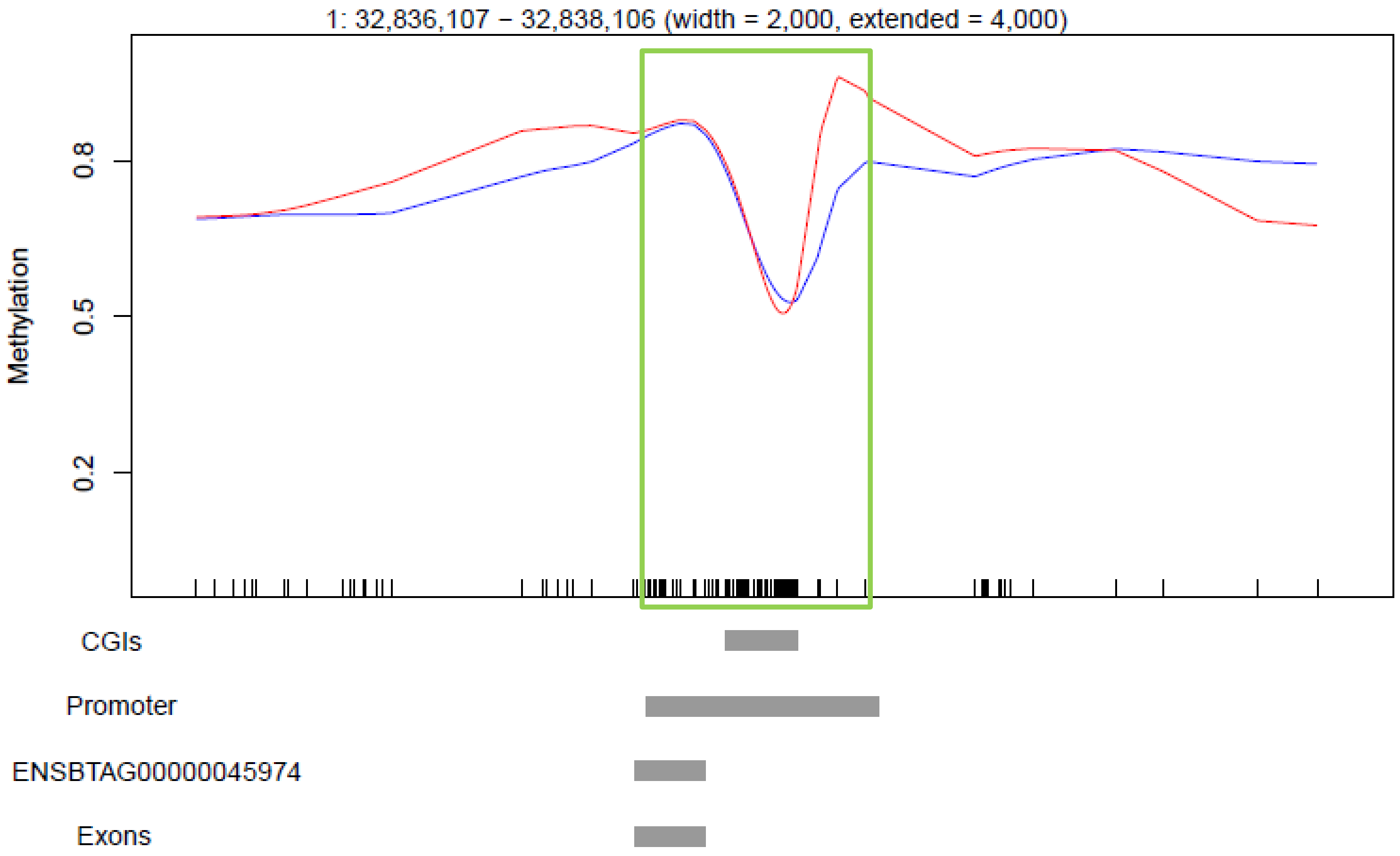
1: 32,836,107 − 32,838,106 (width = 2,000, extended = 4,000)
Methylation
0.8
0.5
0.2
CGIs
Promoter
ENSBTAG00000045974
Exons

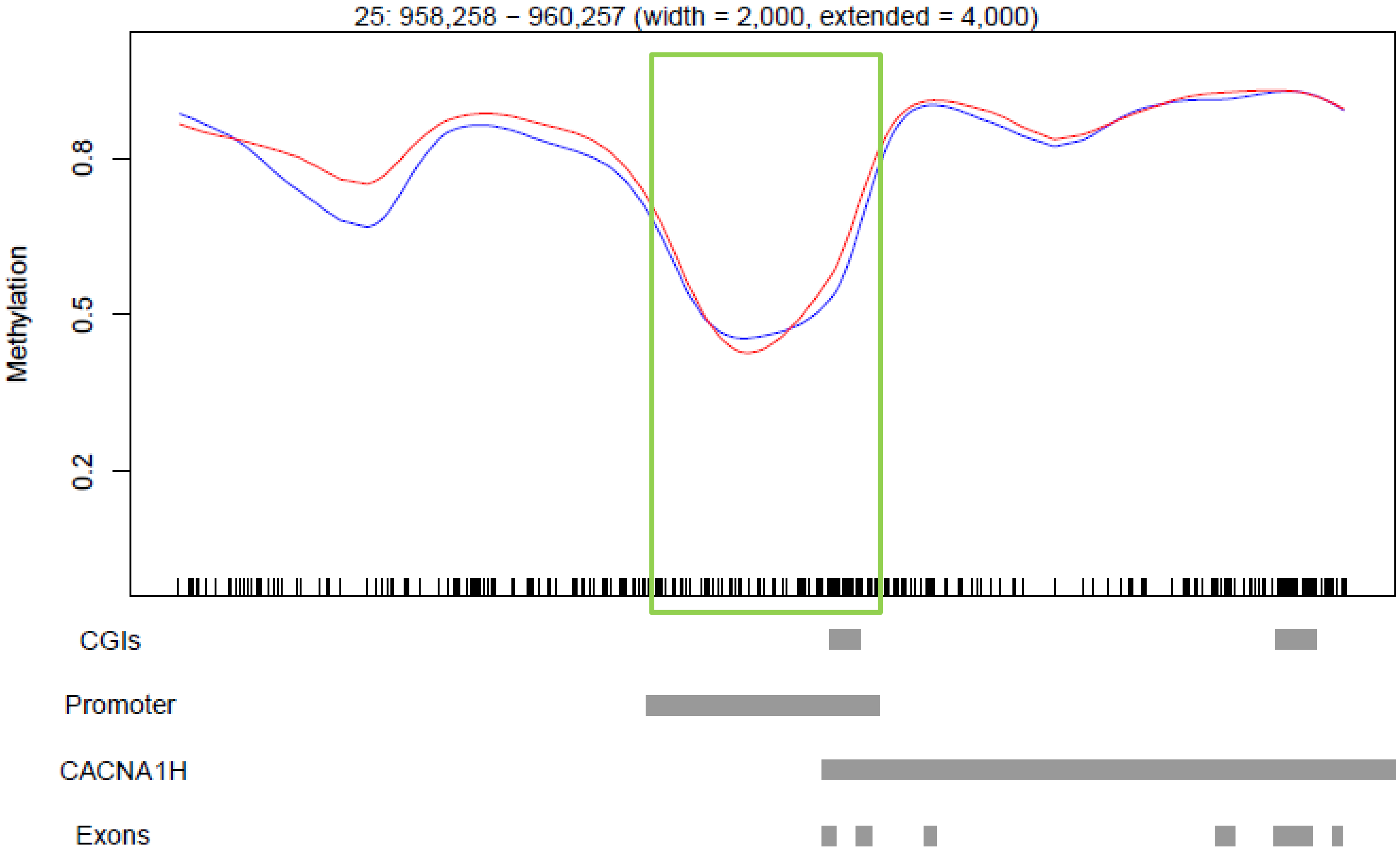

25: 958,258 – 960,257 (width = 2,000, extended = 4,000)
Methylation
0.8
0.5
0.2
CGIs
Promoter
CACNA1H
Exons

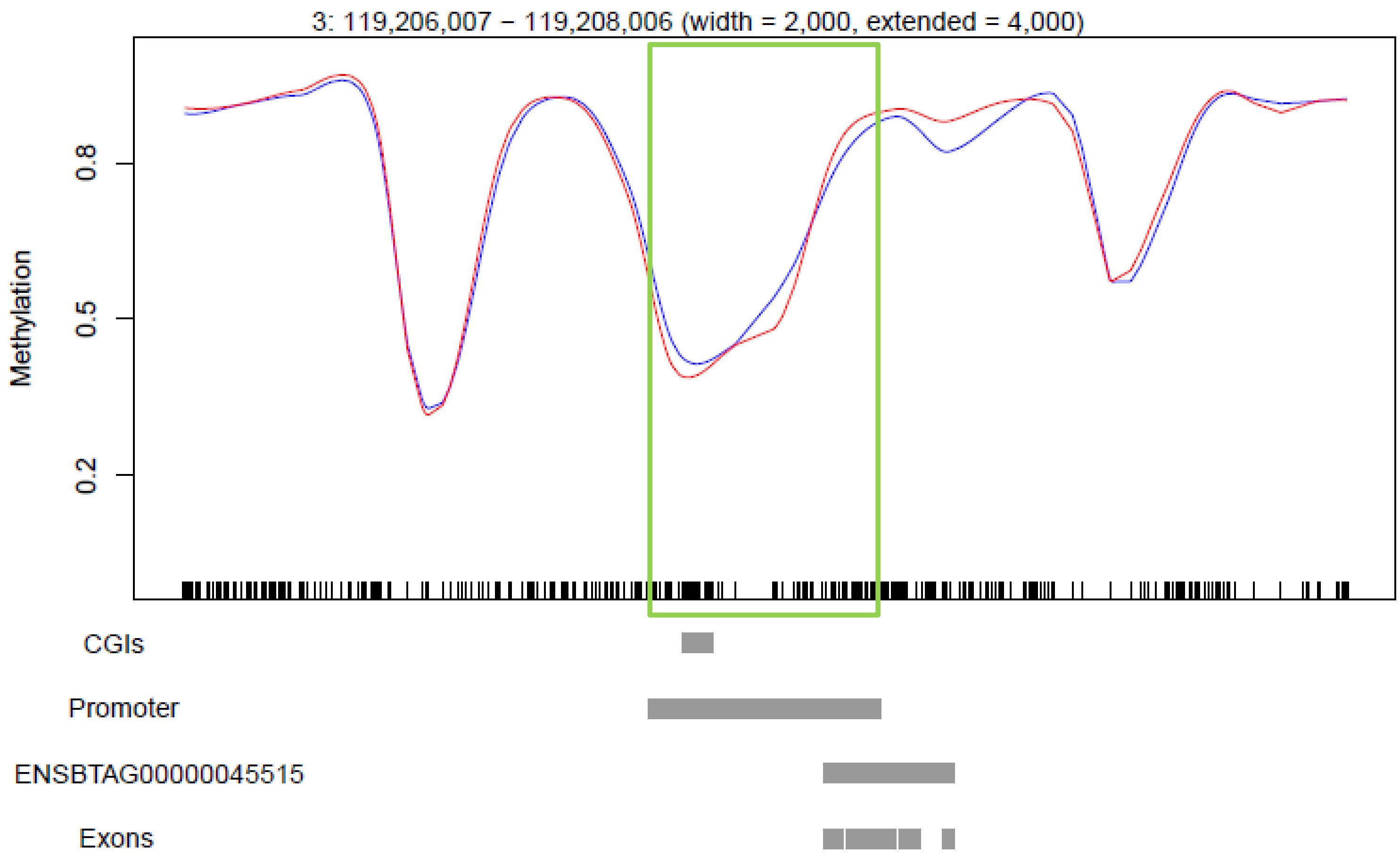
3: 119,206,007 − 119,208,006 (width = 2,000, extended = 4,000)
Methylation
0.8
0.5
0.2
CGIs
Promoter
ENSBTAG00000045515
Exons

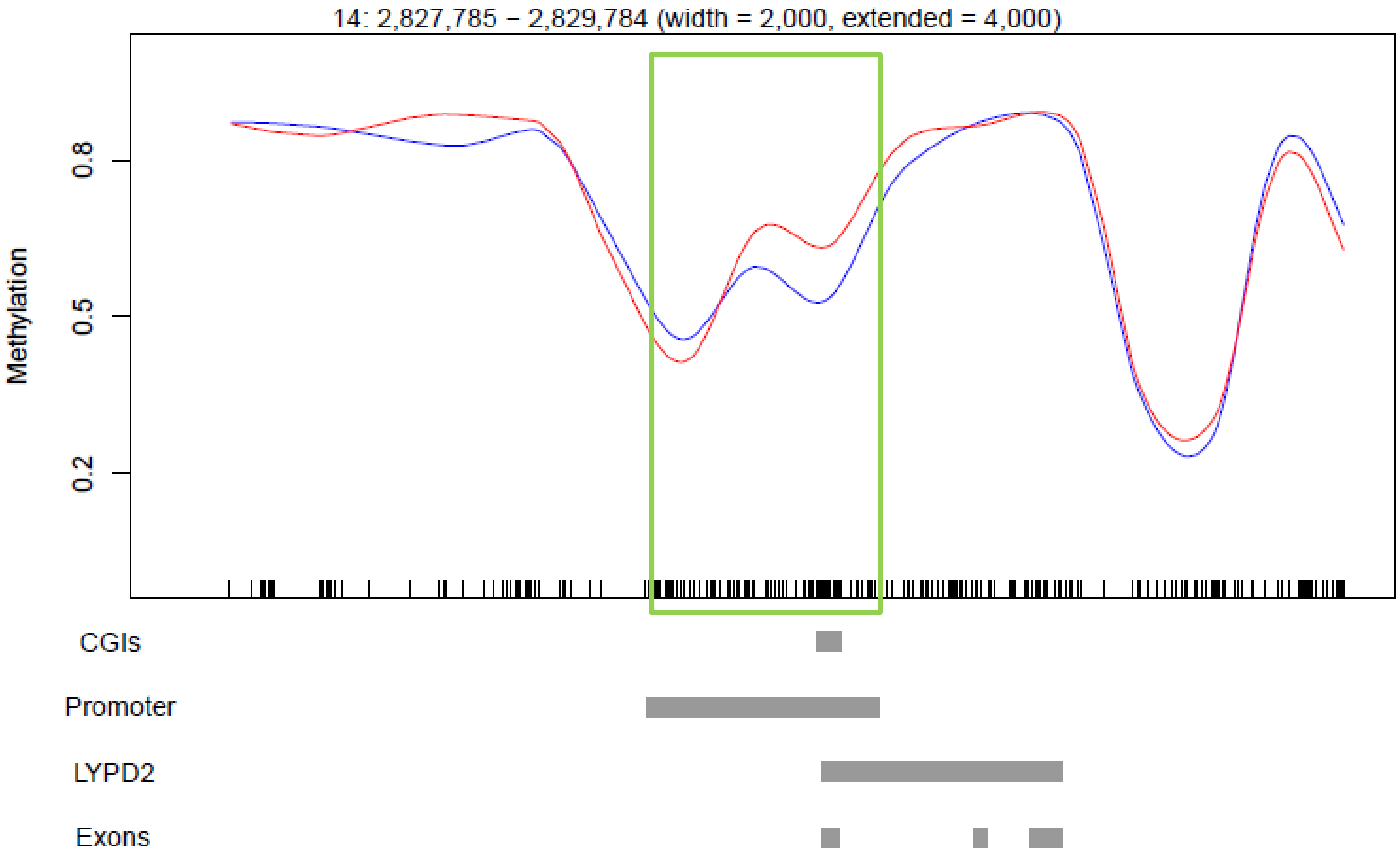
14: 2,827,785 − 2,829,784 (width = 2,000, extended = 4,000)
Methylation
0.8
0.5
0.2
CGIs
Promoter
LYPD2
Exons

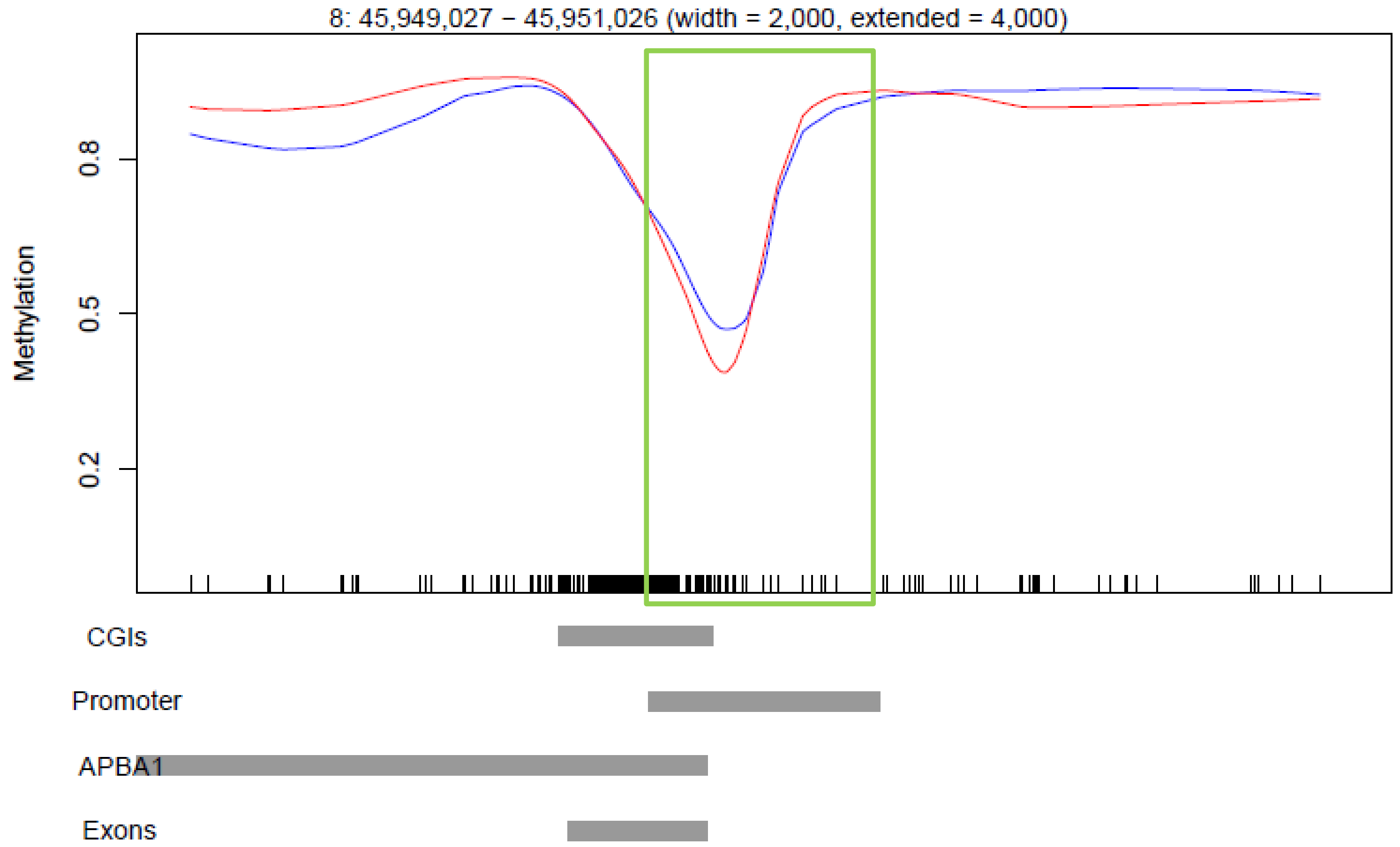

8: 45,949,027 − 45,951,026 (width = 2,000, extended = 4,000)
Methylation
0.8
0.5
0.2
CGIs
Promoter
APBA1
Exons

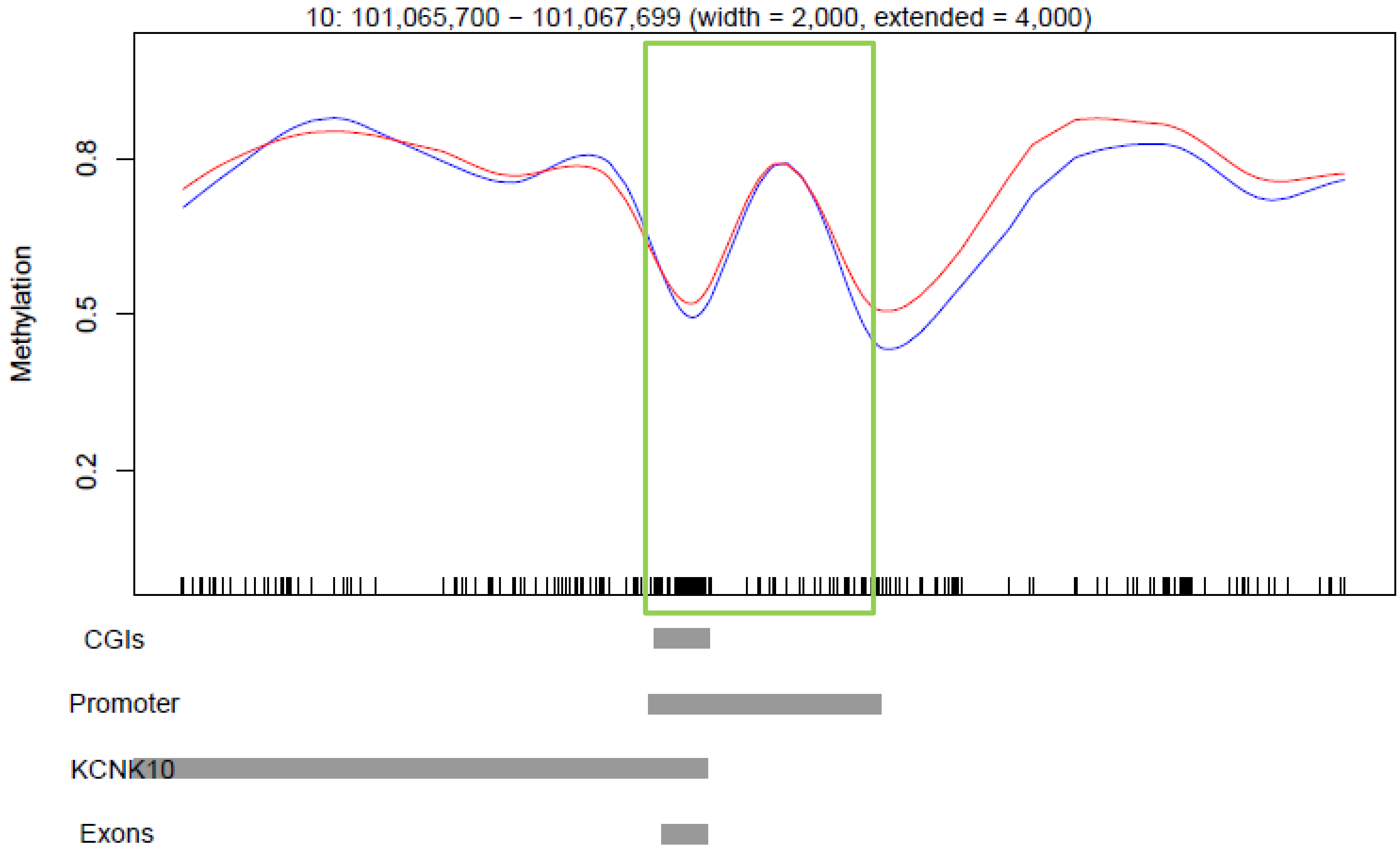

10: 101,065,700 – 101,067,699 (width = 2,000, extended = 4,000)
Methylation
0.2
0.5
0.8
CGIs
Promoter
KCNK10
Exons

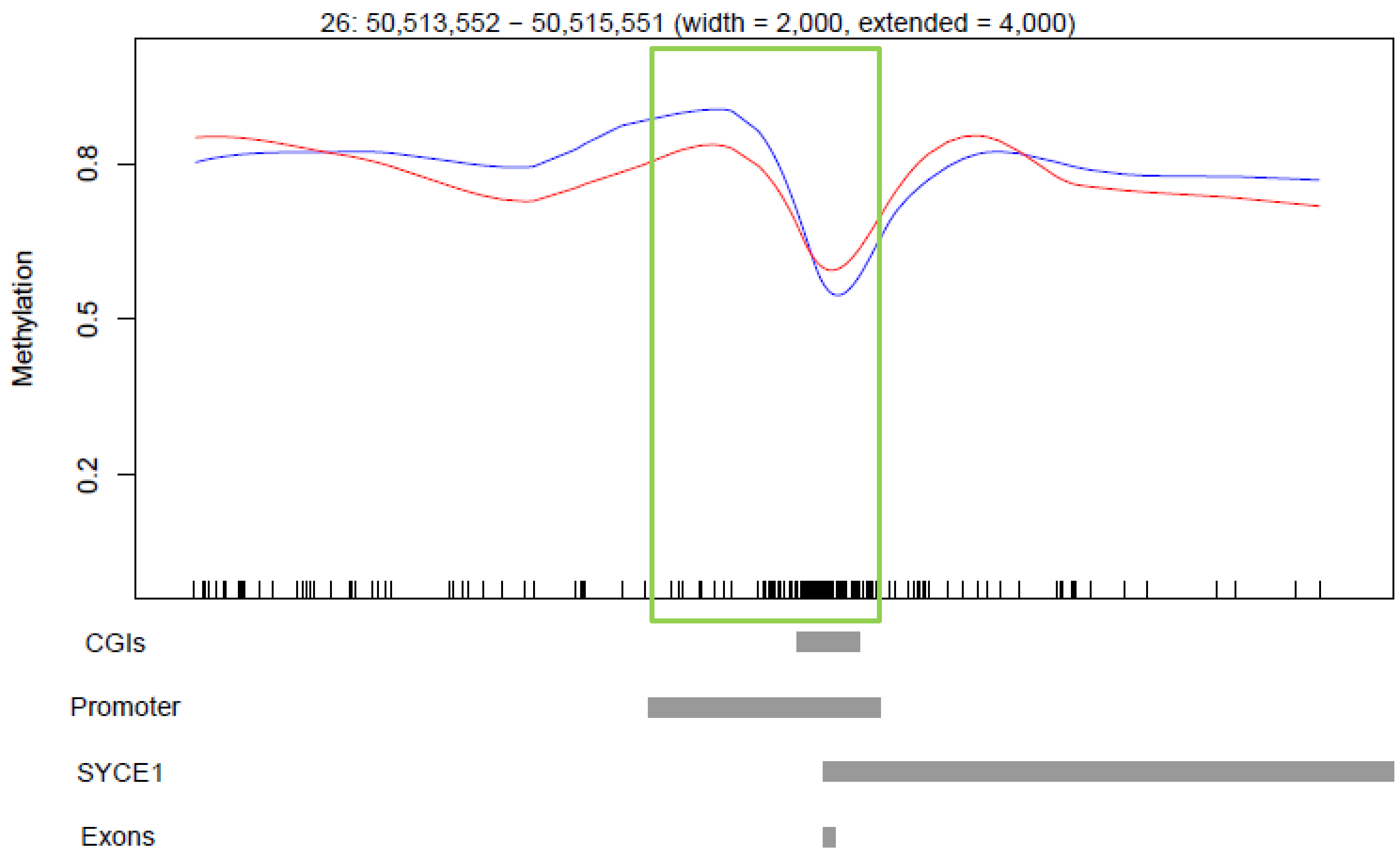
26: 50,513,552 − 50,515,551 (width = 2,000, extended = 4,000)
Methylation
0.8
0.5
0.2
CGIs
Promoter
SYCE1
Exons

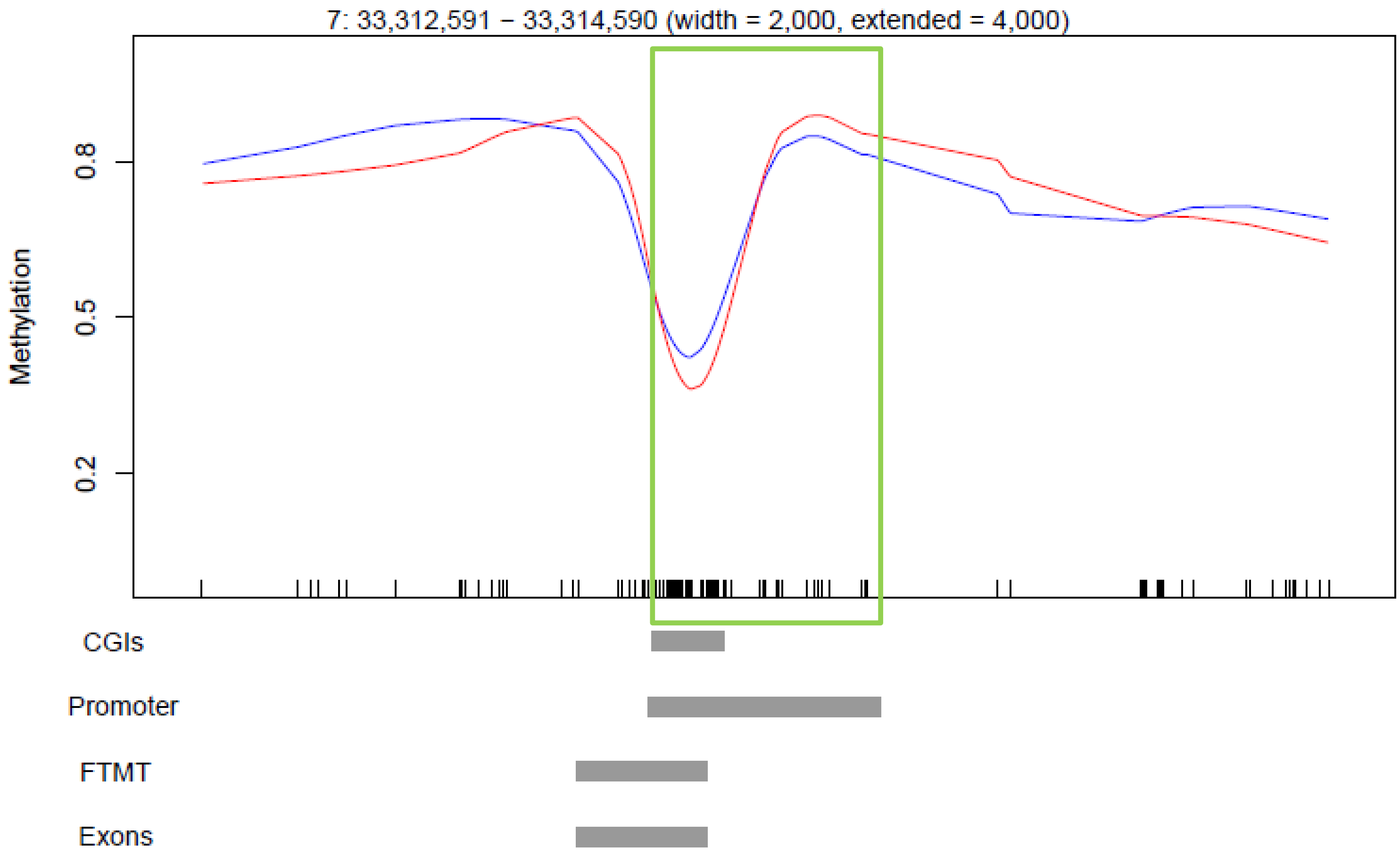
7: 33,312,591 − 33,314,590 (width = 2,000, extended = 4,000)
Methylation
0.2
0.5
0.8
CGIs
Promoter
FTMT
Exons

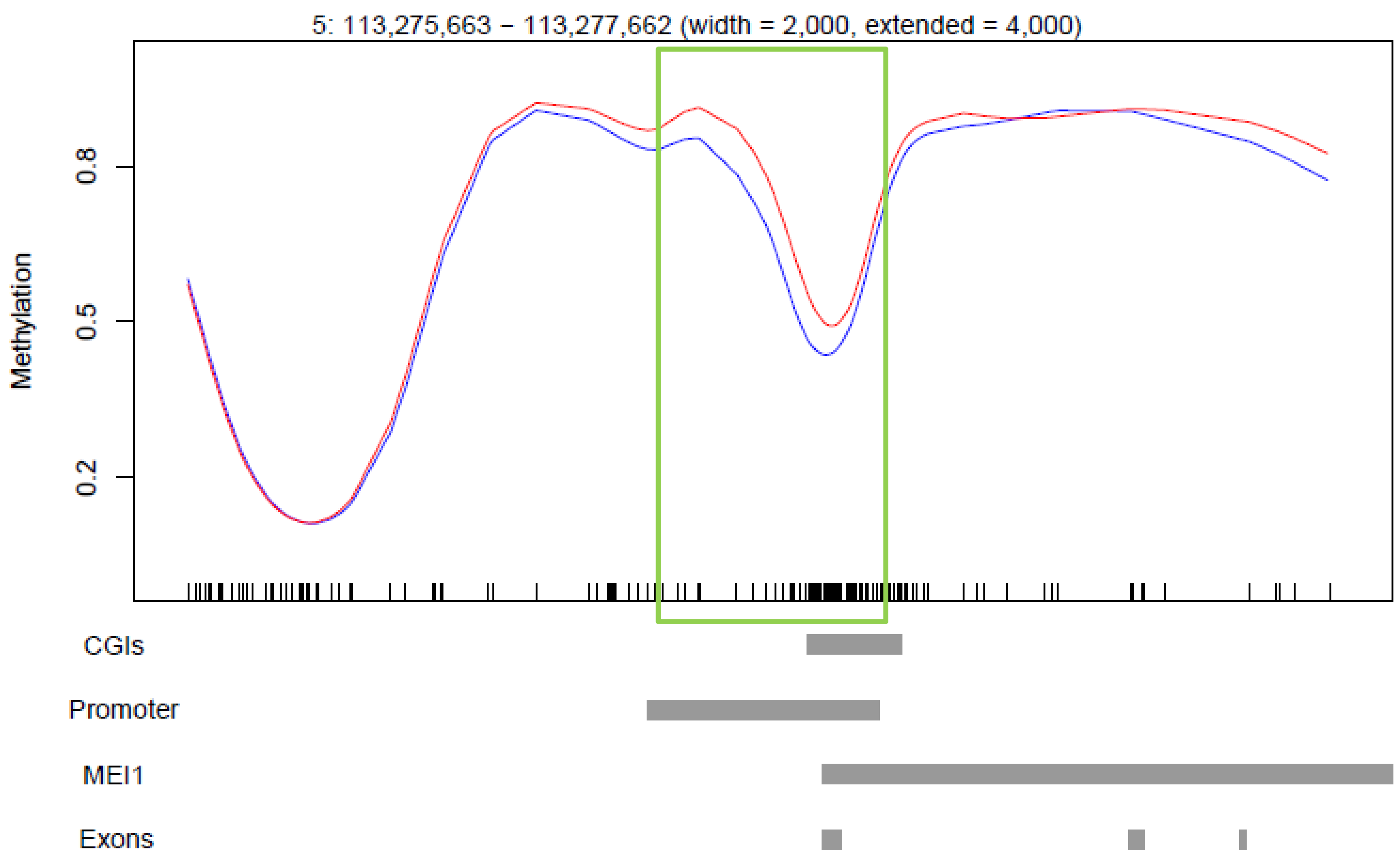
5: 113,275,663 − 113,277,662 (width = 2,000, extended = 4,000)
Methylation
0.2
0.5
0.8
CGIs
Promoter
MEI1
Exons

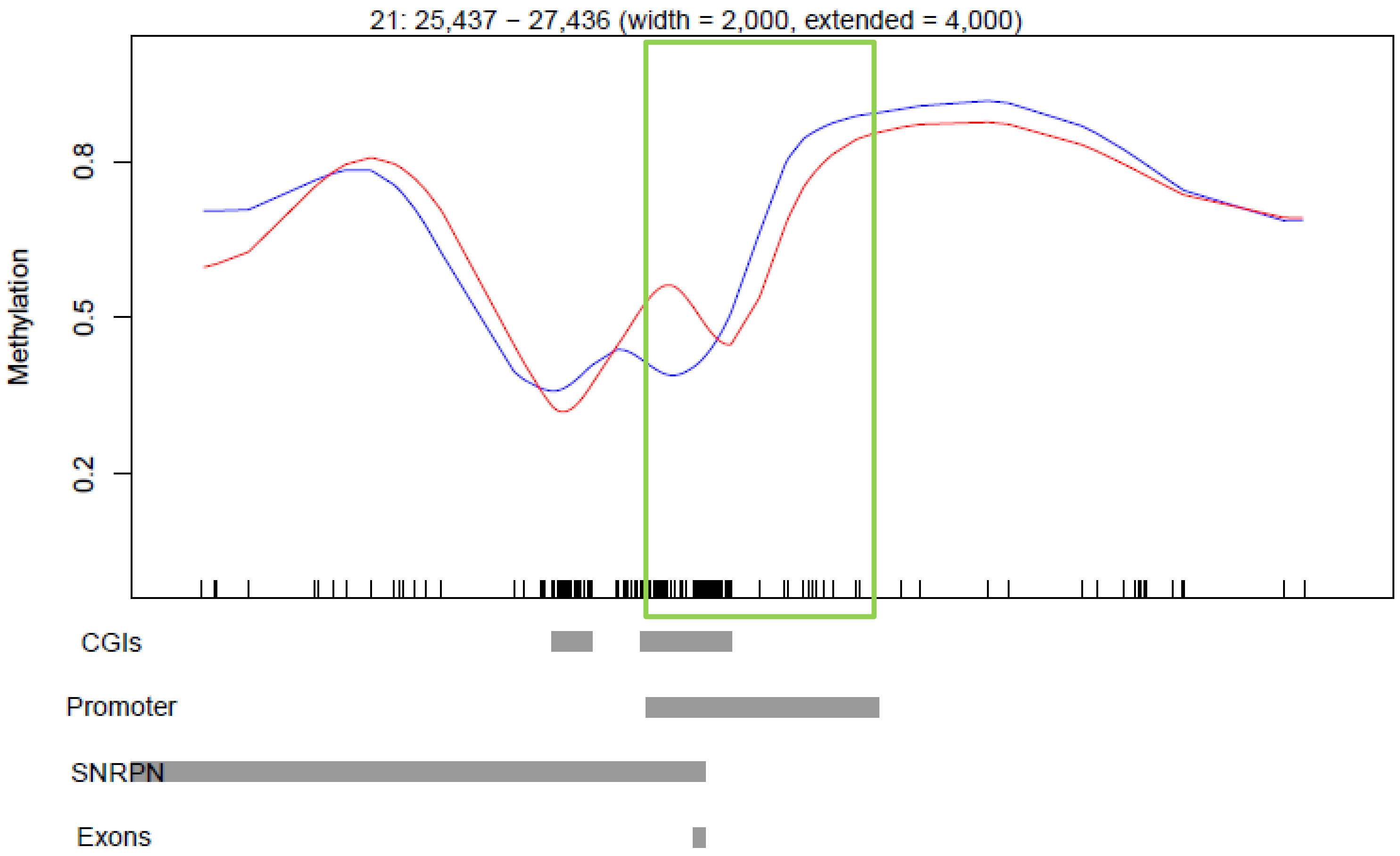

21: 25,437 − 27,436 (width = 2,000, extended = 4,000)
Methylation
0.8
0.5
0.2
CGIs
Promoter
SNRPN
Exons

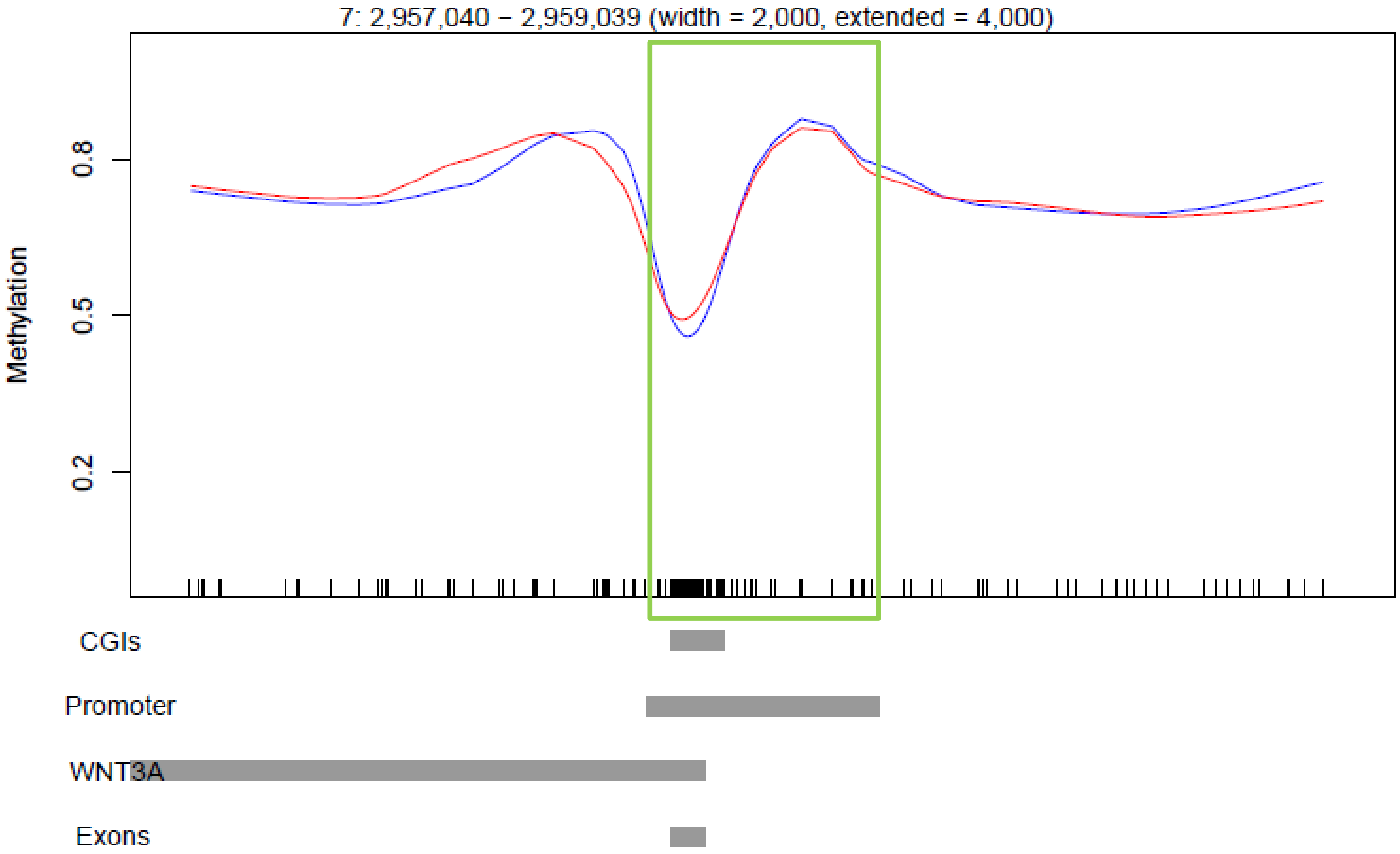
7: 2,957,040 − 2,959,039 (width = 2,000, extended = 4,000)
Methylation
0.8
0.5
0.2
CGIs
Promoter
WNT3A
Exons

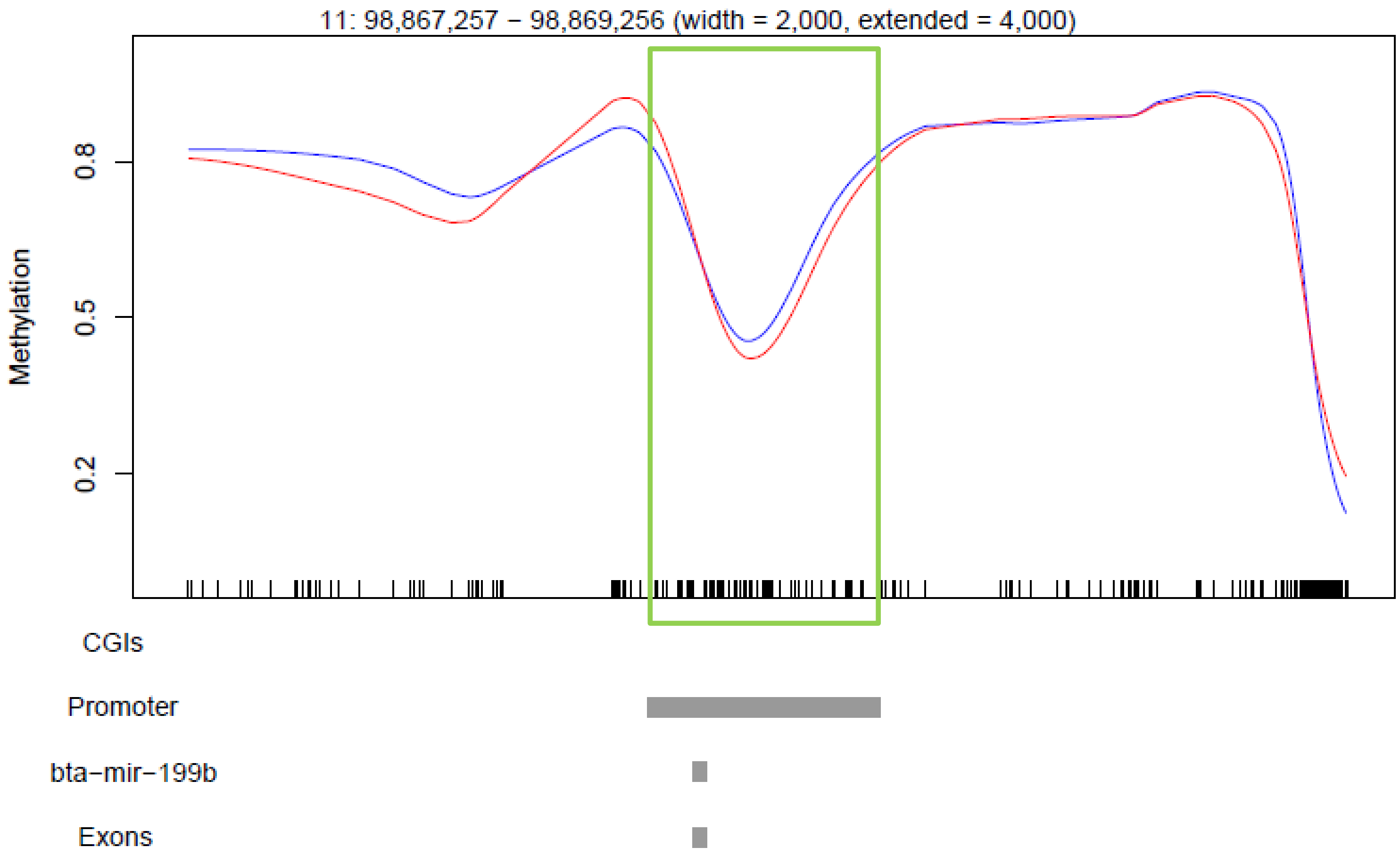
11: 98,867,257 − 98,869,256 (width = 2,000, extended = 4,000)
Methylation
0.8
0.5
0.2
CGIs
Promoter
bta−mir−199b
Exons

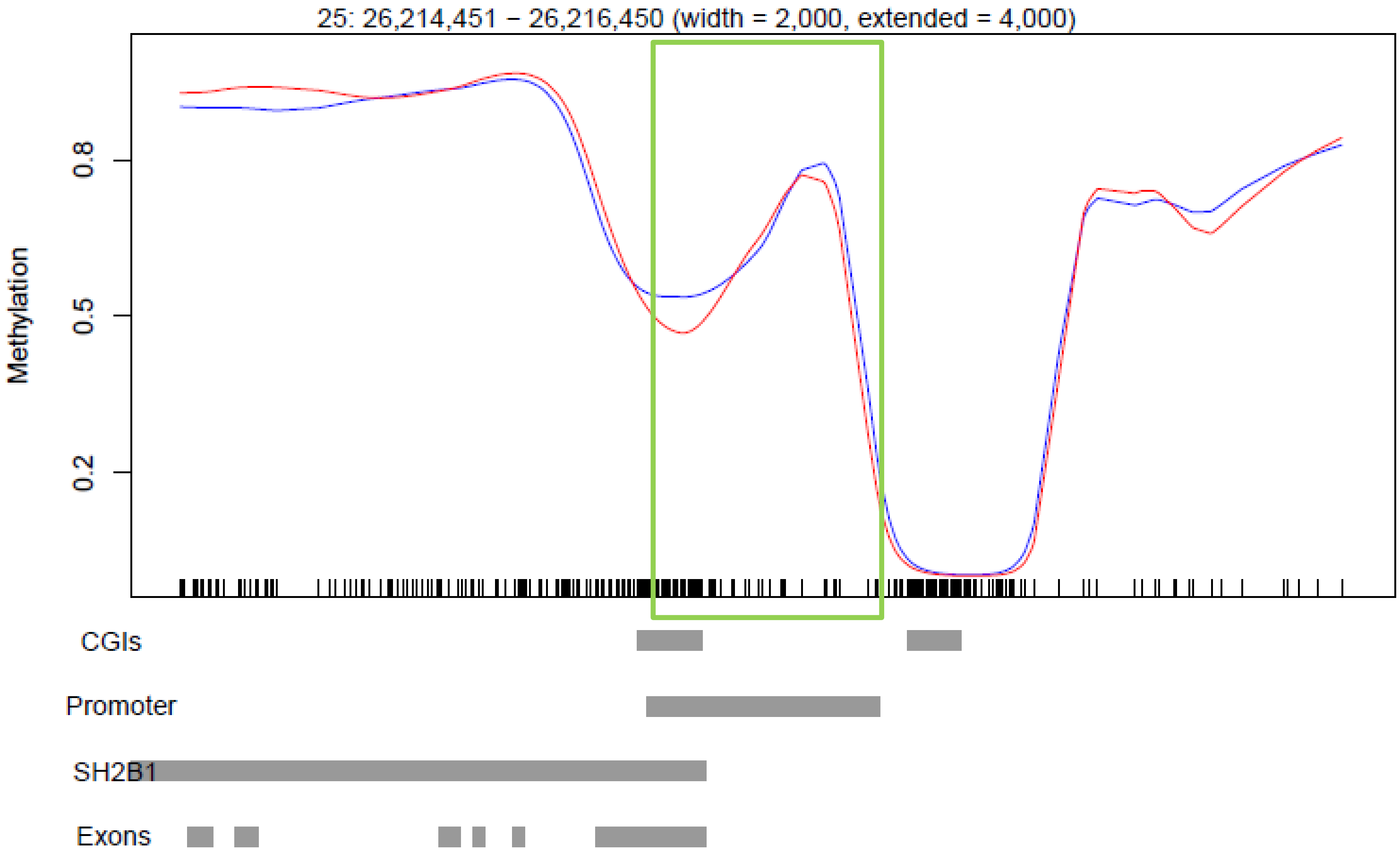
25: 26,214,451 – 26,216,450 (width = 2,000, extended = 4,000)
Methylation
0.8
0.5
0.2
CGIs
Promoter
SH2B1
Exons

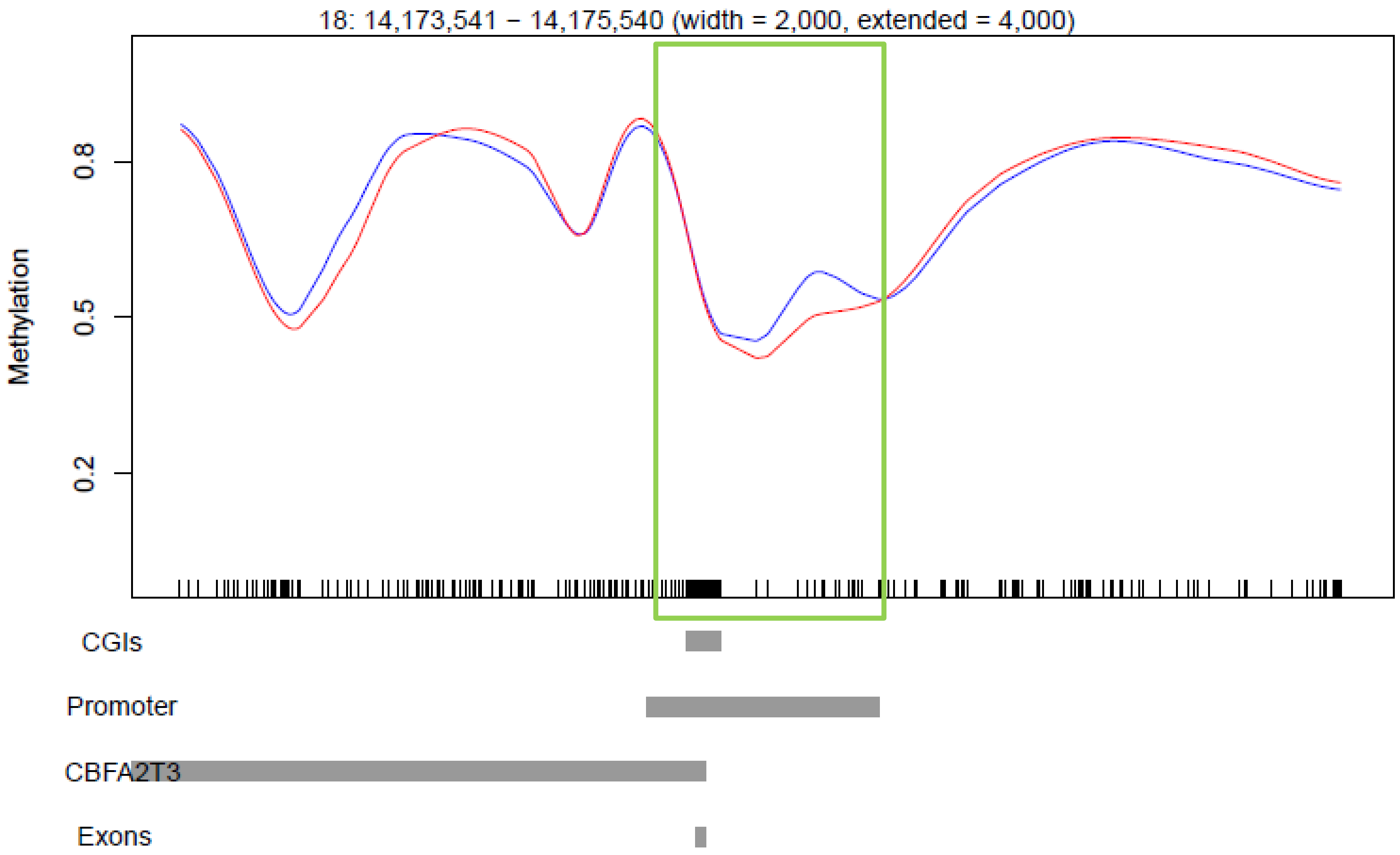

18: 14,173,541 − 14,175,540 (width = 2,000, extended = 4,000)
Methylation
0.8
0.5
0.2
CGIs
Promoter
CBFA2T3
Exons

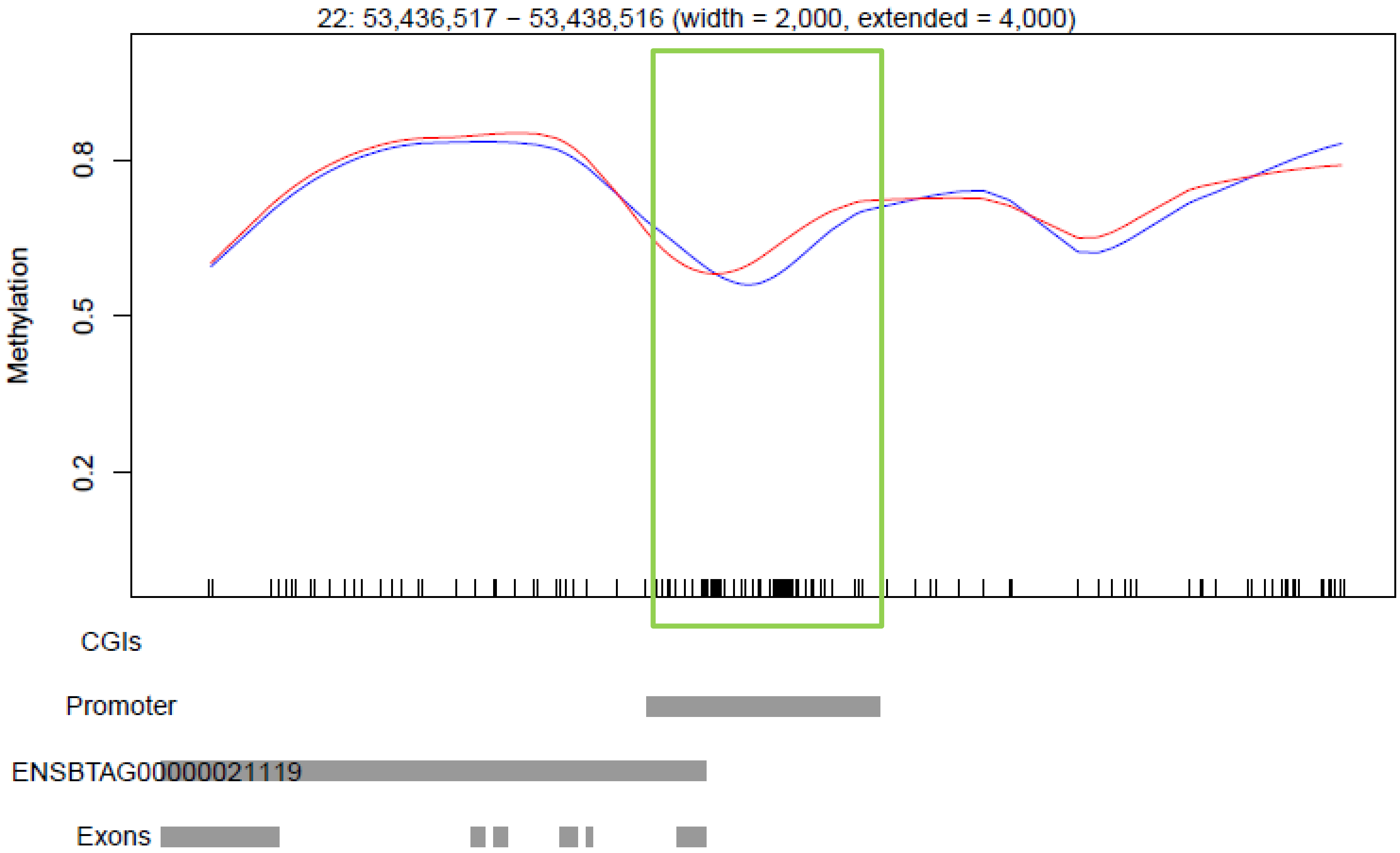

22: 53,436,517 − 53,438,516 (width = 2,000, extended = 4,000)
Methylation
0.8
0.5
0.2
CGIs
Promoter
ENSBTAG00000021119
Exons

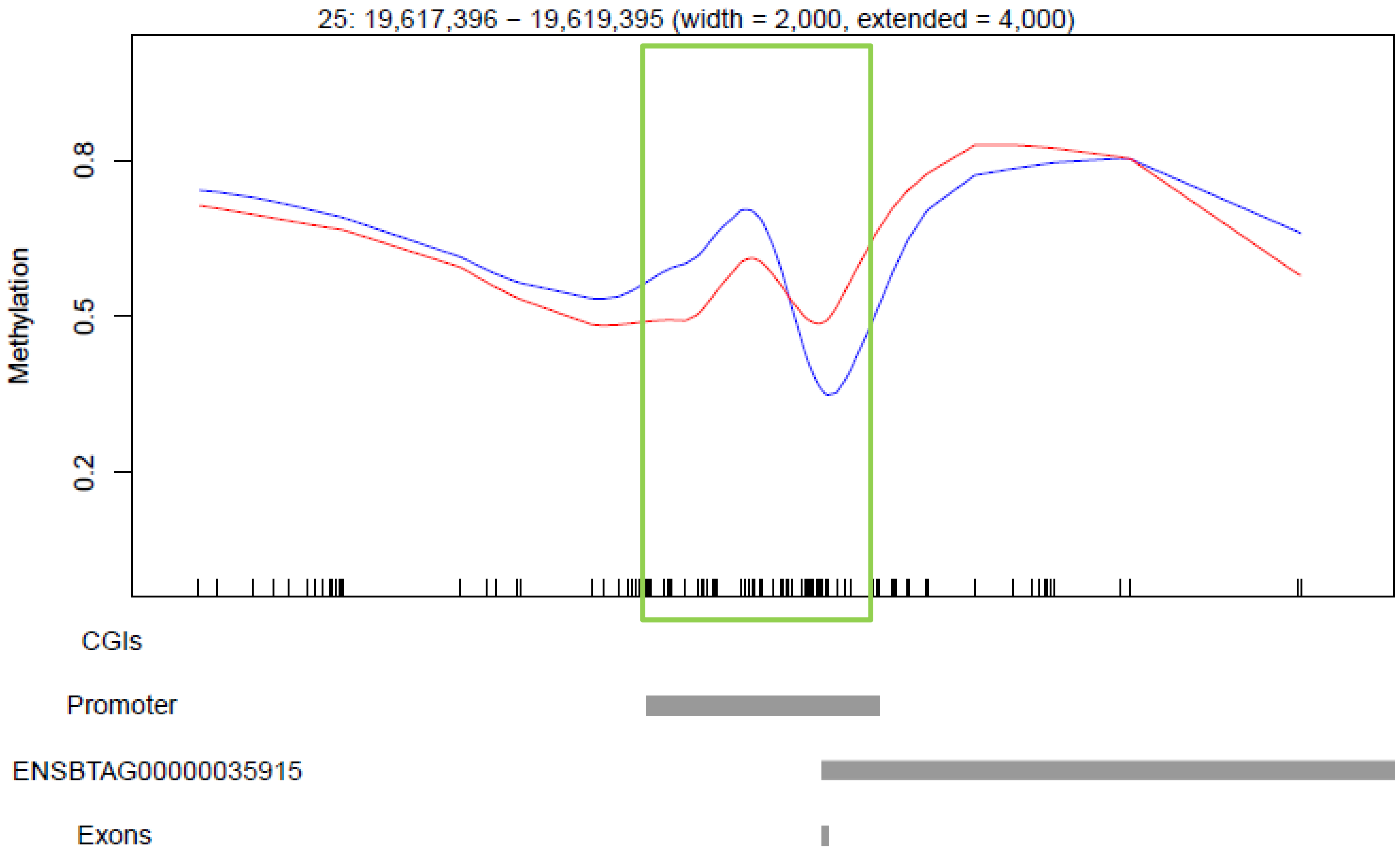
25: 19,617,396 − 19,619,395 (width = 2,000, extended = 4,000)
Methylation
0.8
0.5
0.2
CGIs
Promoter
ENSBTAG00000035915
Exons

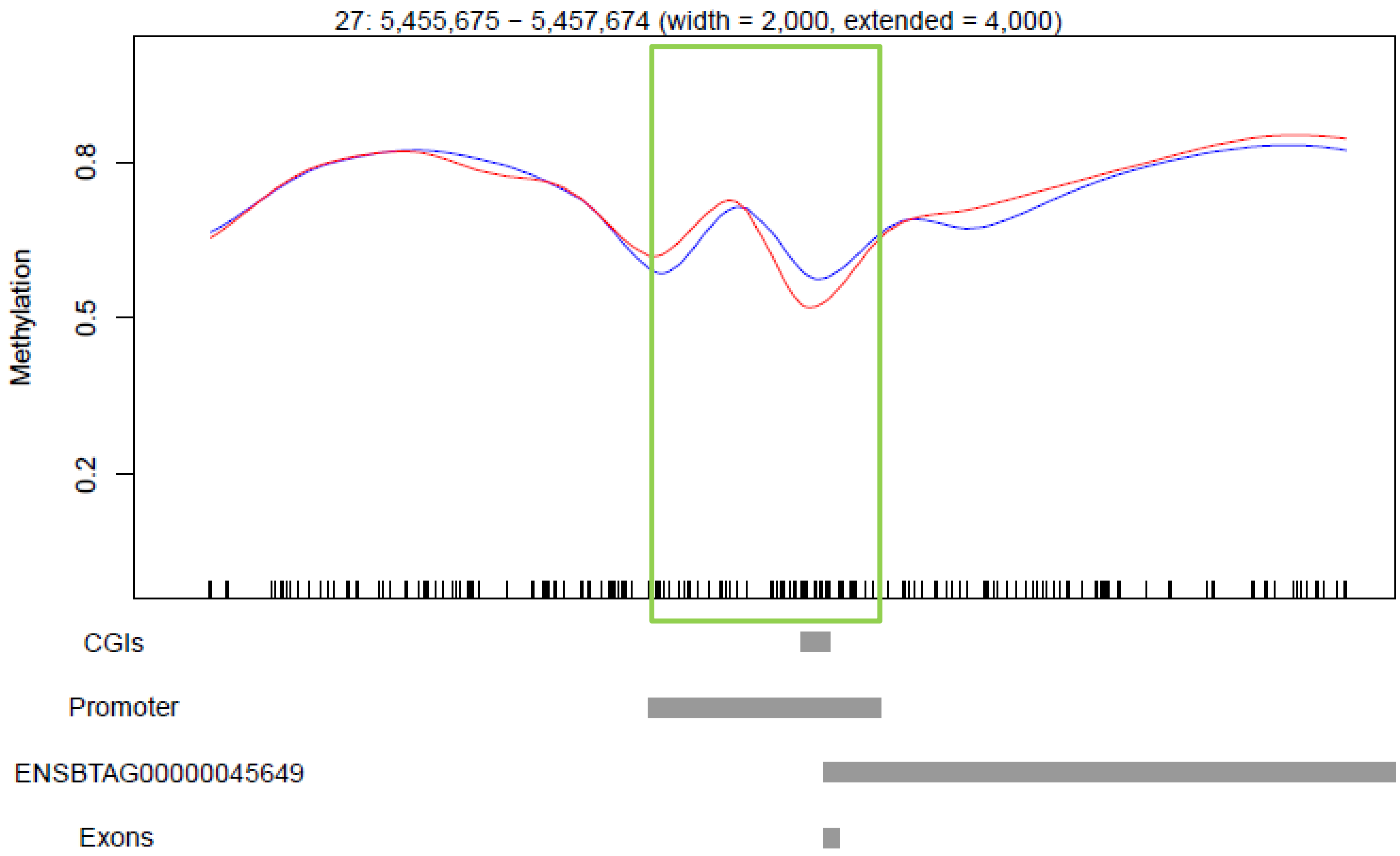

27: 5,455,675 − 5,457,674 (width = 2,000, extended = 4,000)
Methylation
0.8
0.5
0.2
CGIs
Promoter
ENSBTAG00000045649
Exons

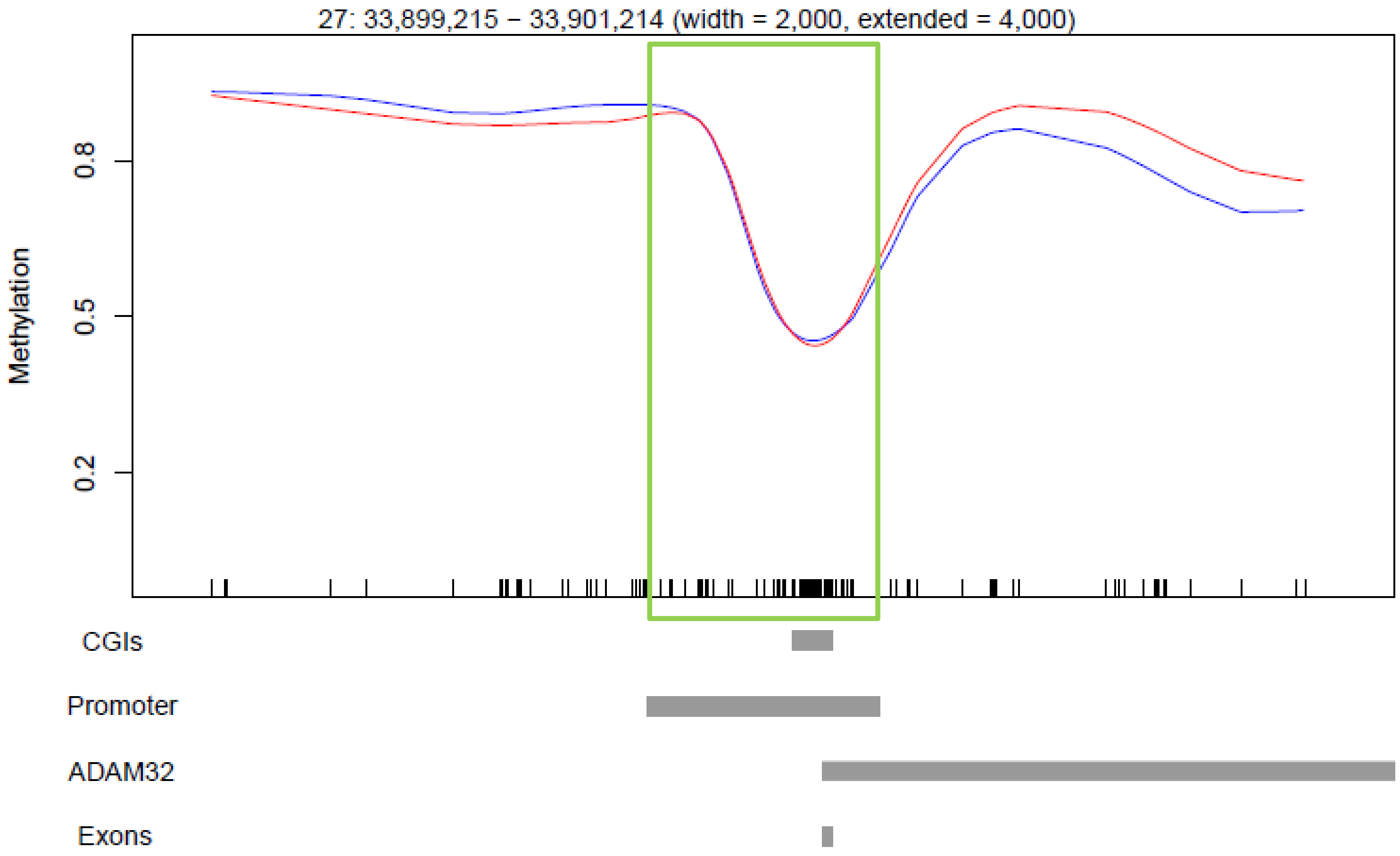
27: 33,899,215 – 33,901,214 (width = 2,000, extended = 4,000)
Methylation
0.8
0.5
0.2
CGIs
Promoter
ADAM32
Exons

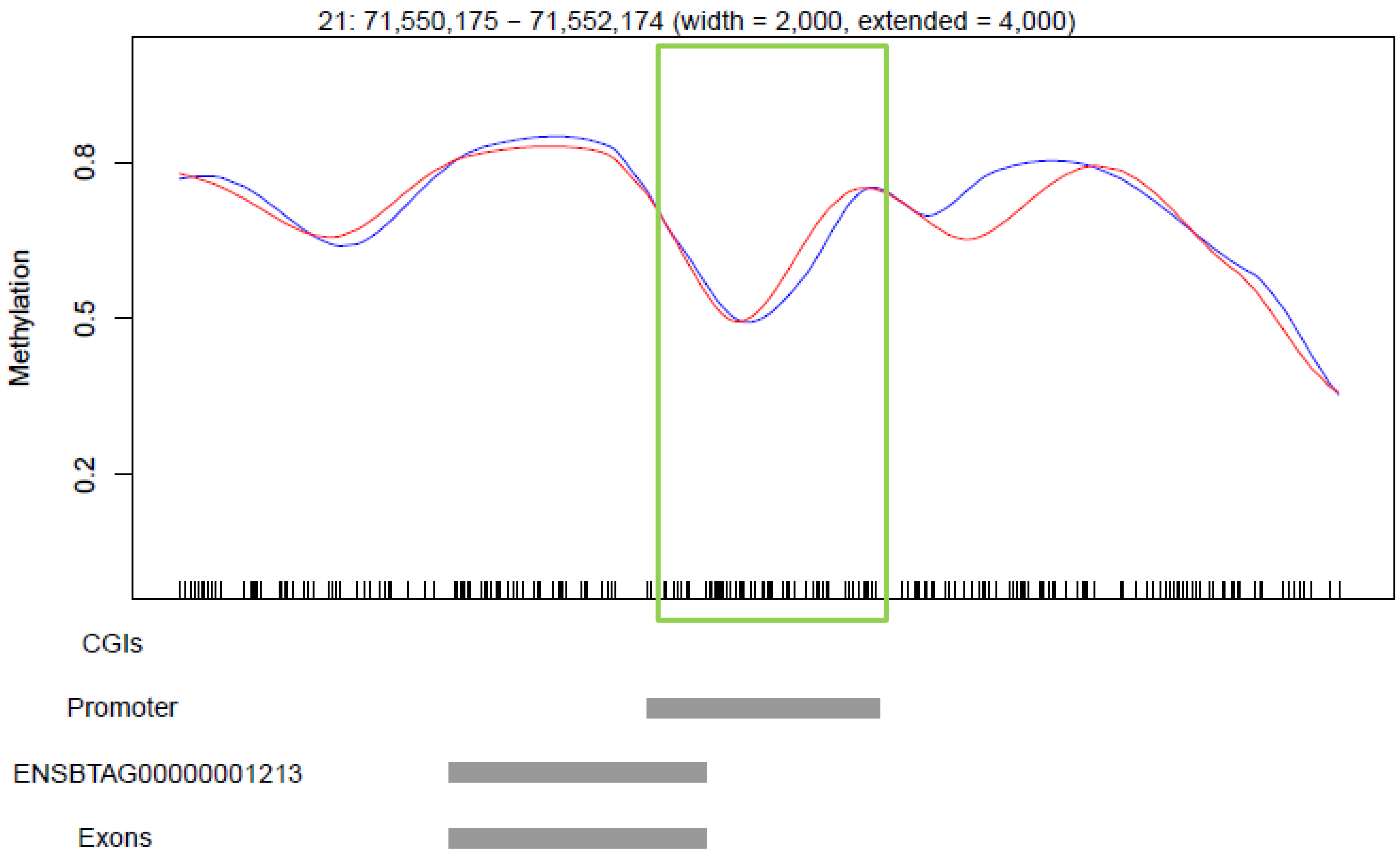

21: 71,550,175 – 71,552,174 (width = 2,000, extended = 4,000)
Methylation
0.8
0.5
0.2
CGIs
Promoter
ENSBTAG00000001213
Exons

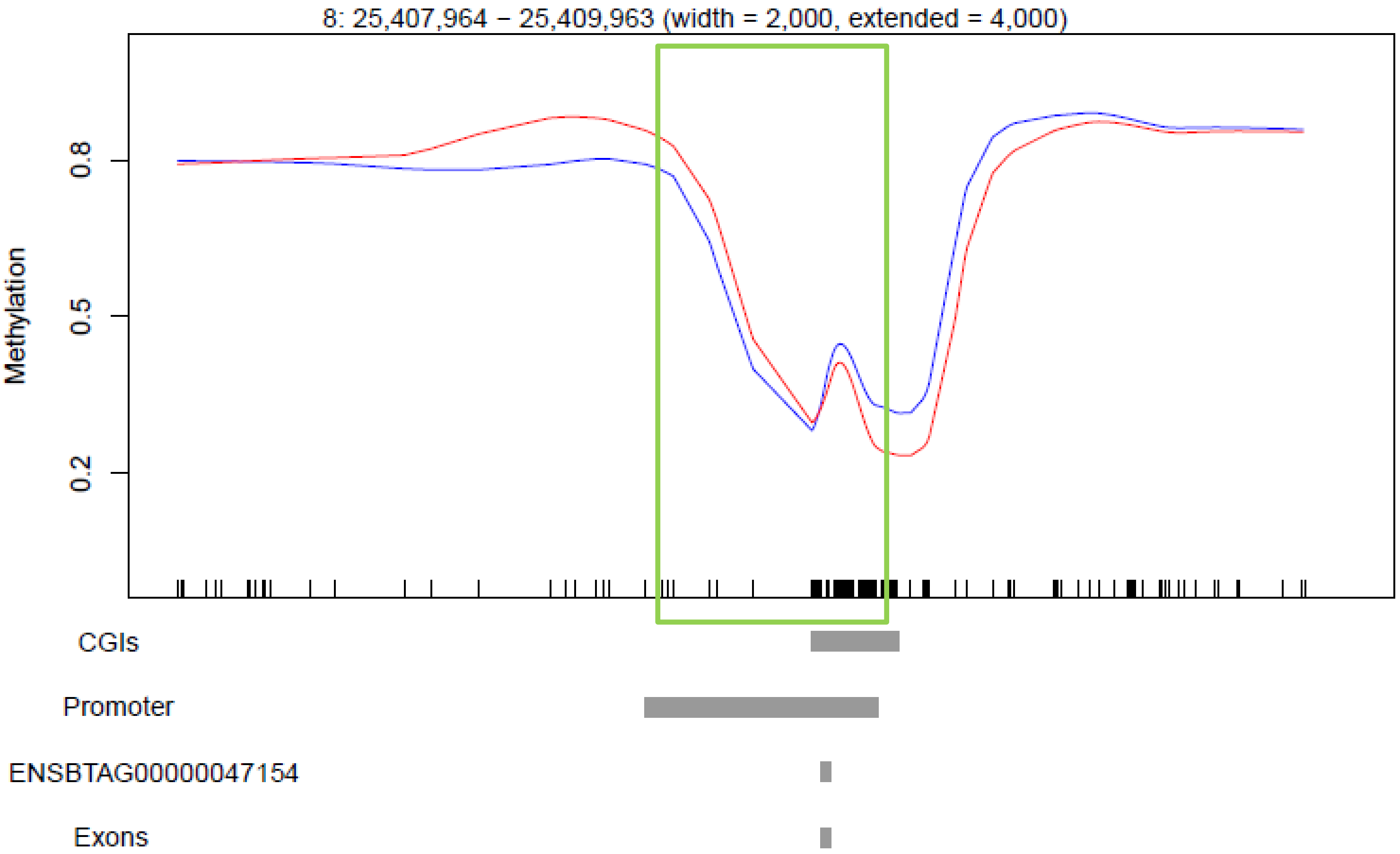
8: 25,407,964 − 25,409,963 (width = 2,000, extended = 4,000)
Methylation
0.8
0.5
0.2
CGIs
Promoter
ENSBTAG00000047154
Exons

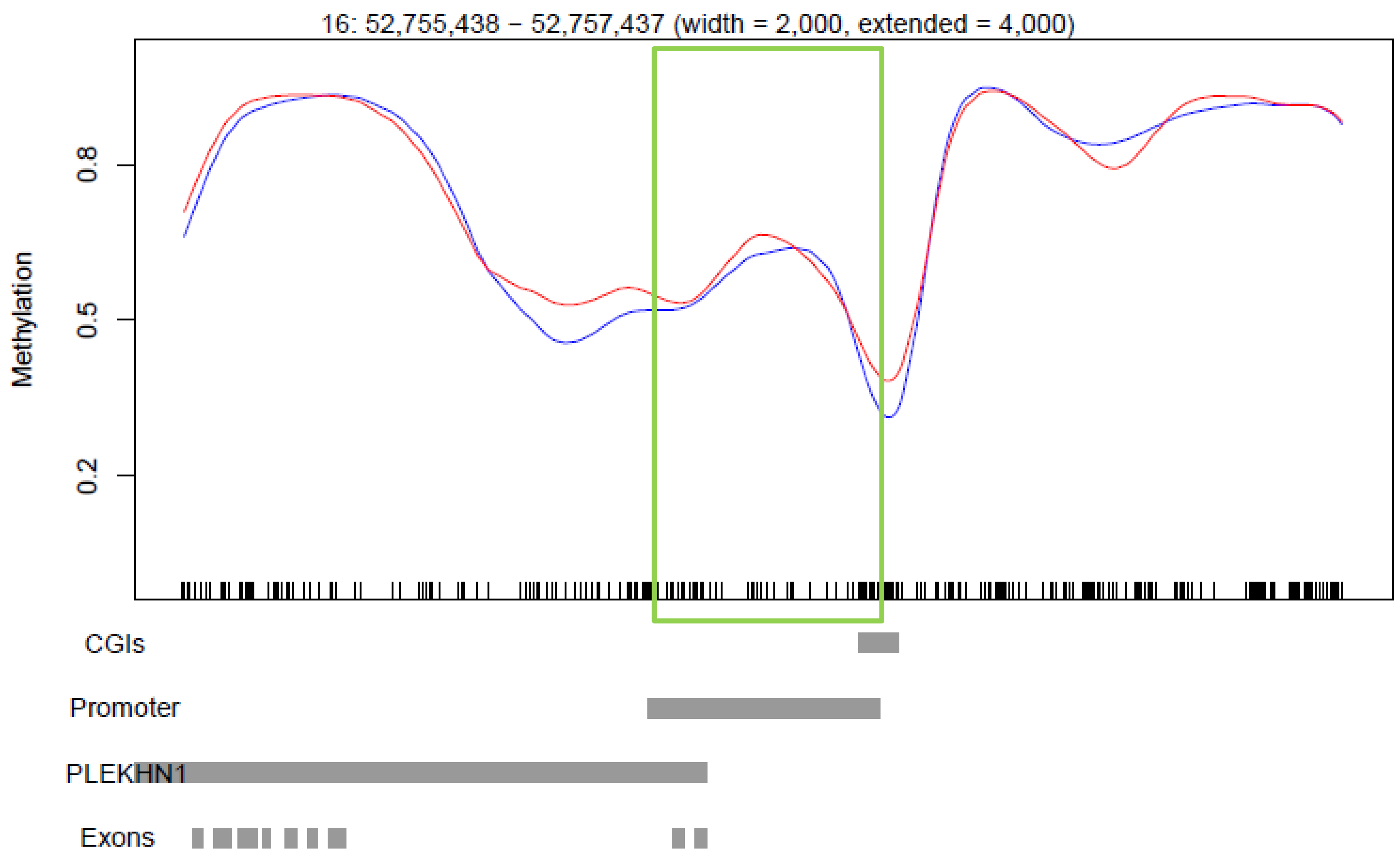

16: 52,755,438 − 52,757,437 (width = 2,000, extended = 4,000)
Methylation
0.8
0.5
0.2
CGIs
Promoter
PLEKHN1
Exons

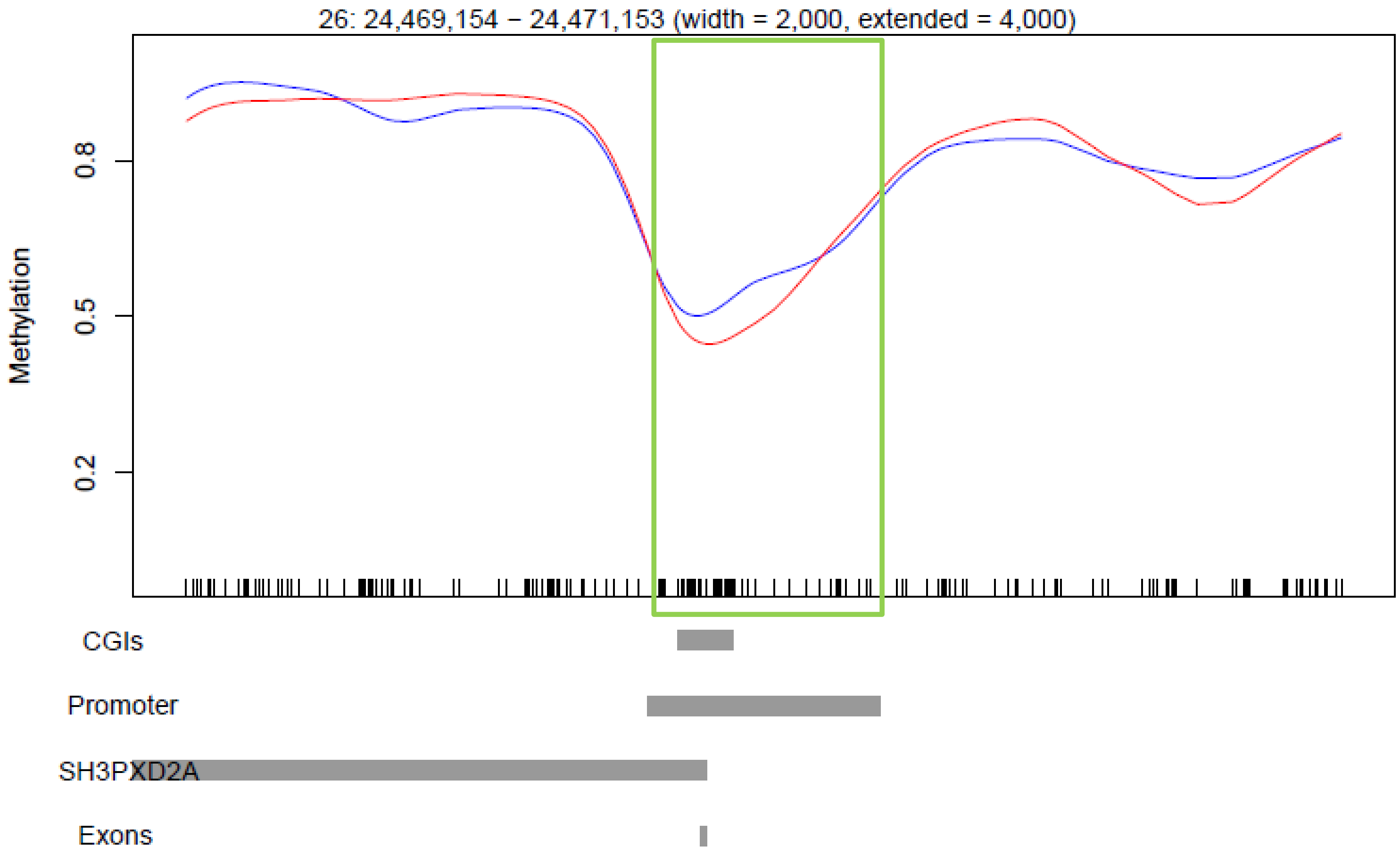
26: 24,469,154 − 24,471,153 (width = 2,000, extended = 4,000)
Methylation
0.8
0.5
0.2
CGIs
Promoter
SH3PXD2A
Exons

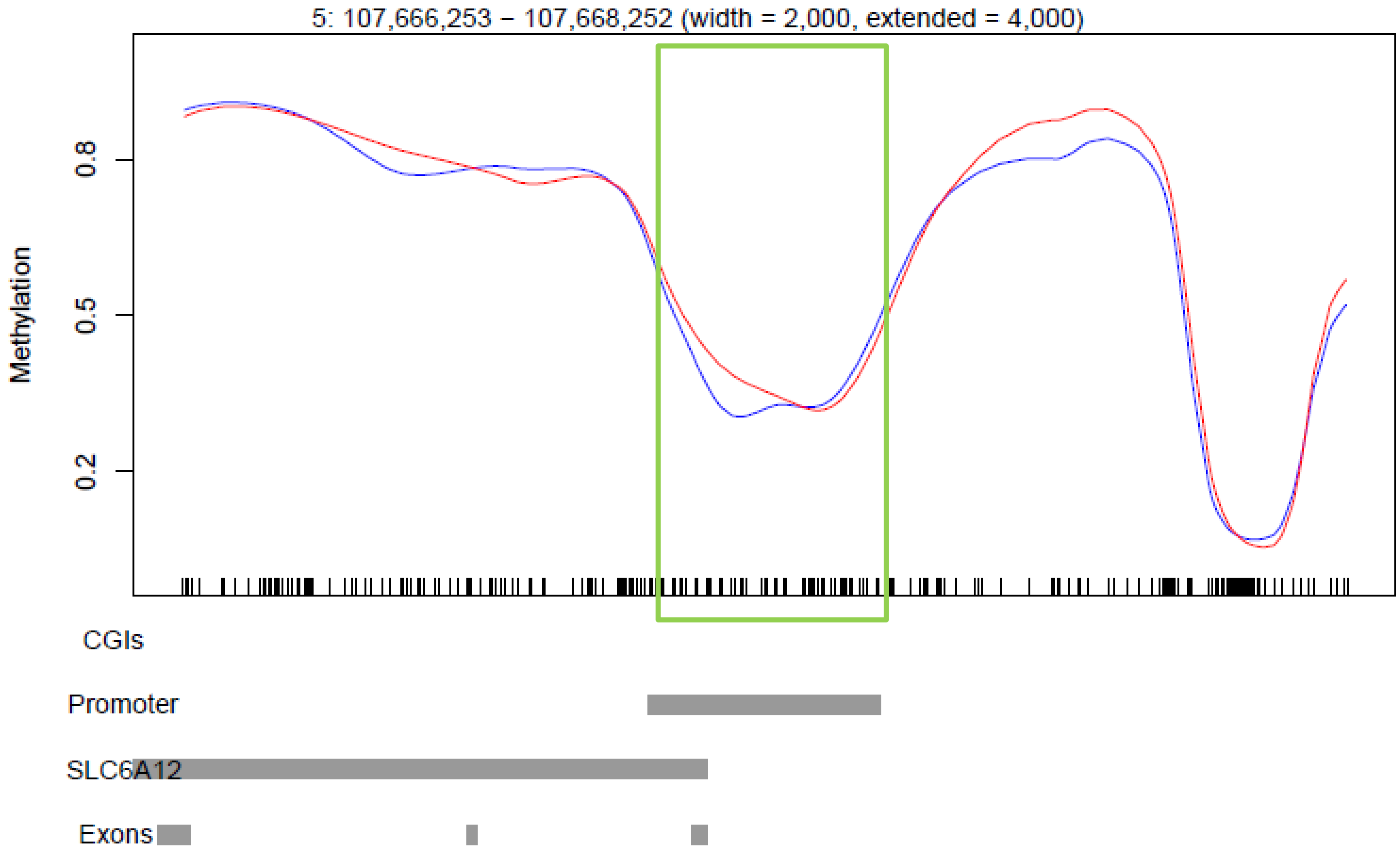

5: 107,666,253 – 107,668,252 (width = 2,000, extended = 4,000)
Methylation
0.2
0.5
0.8
CGIs
Promoter
SLC6A12
Exons

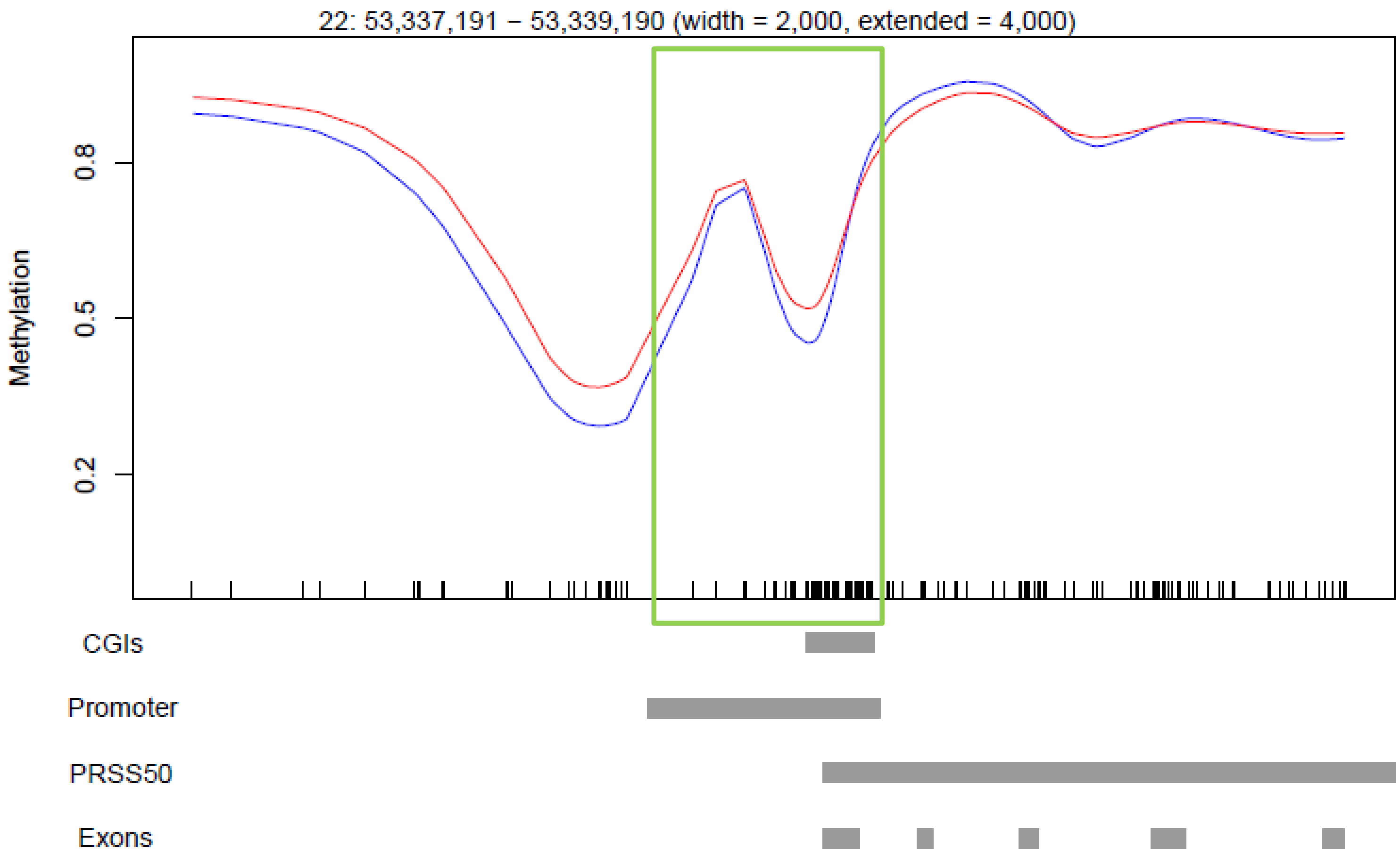
22: 53,337,191 − 53,339,190 (width = 2,000, extended = 4,000)
Methylation
0.2
0.5
0.8
CGIs
Promoter
PRSS50
Exons

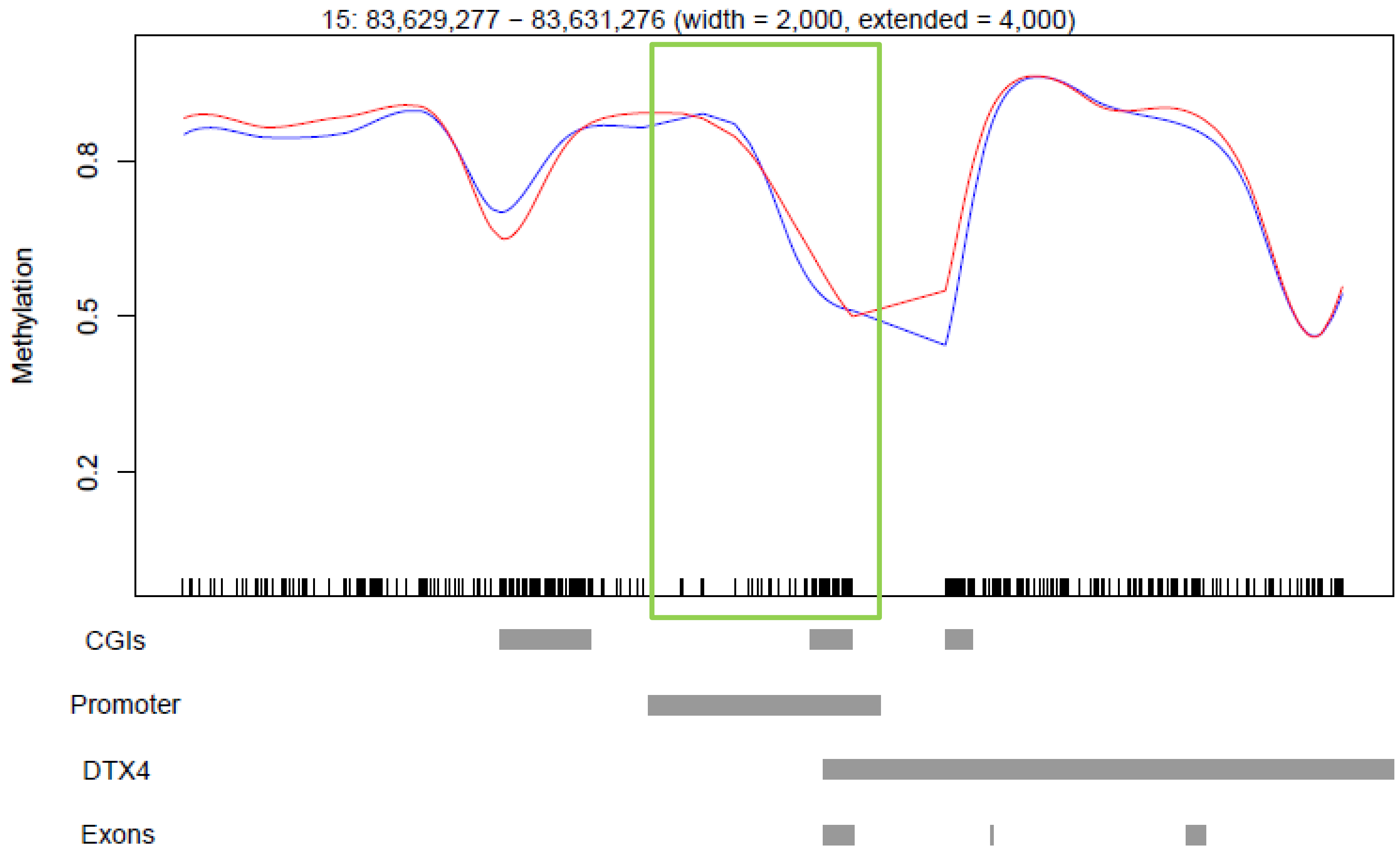

15: 83,629,277 − 83,631,276 (width = 2,000, extended = 4,000)
Methylation
0.8
0.5
0.2
CGIs
Promoter
DTX4
Exons

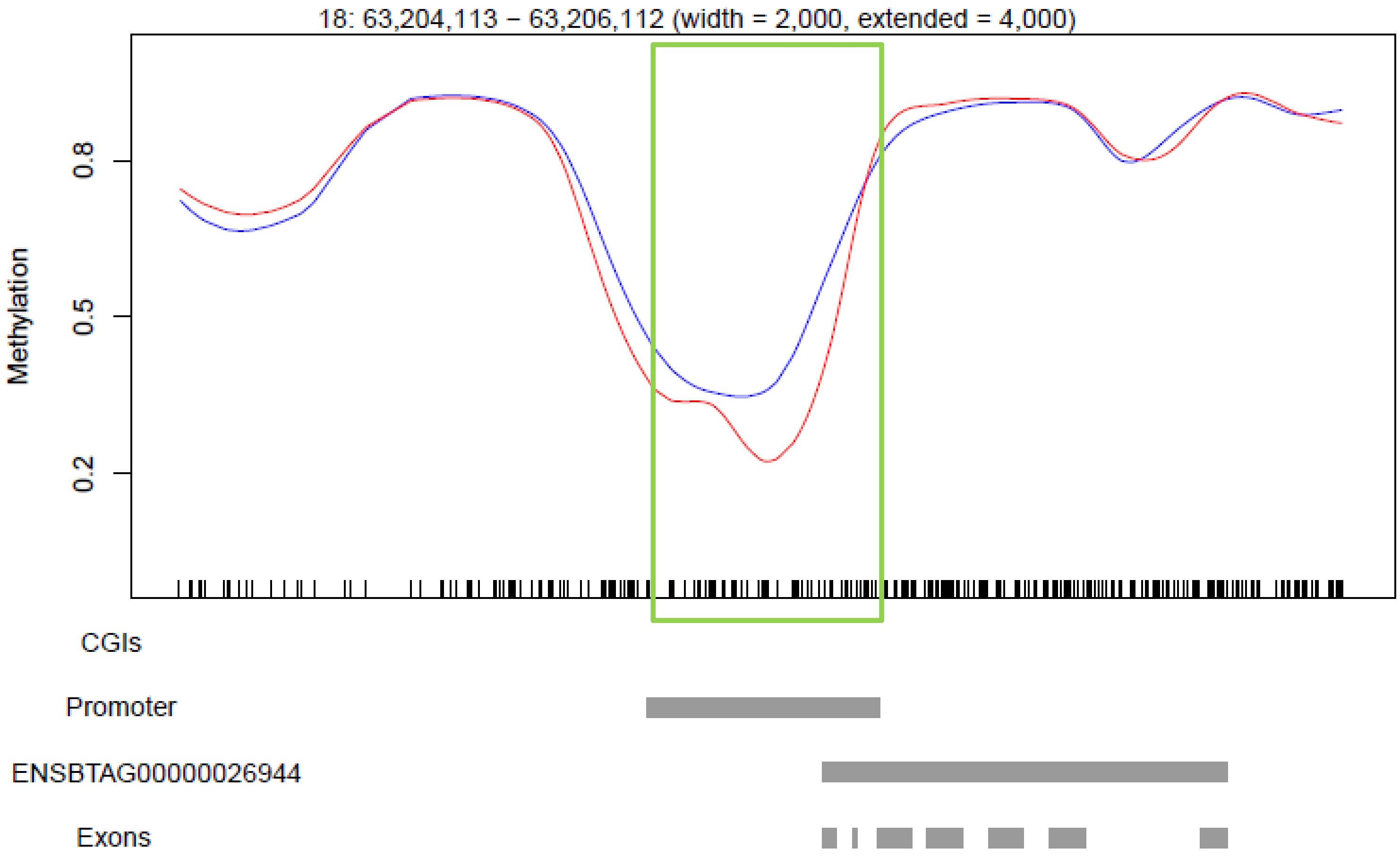

18: 63,204,113 − 63,206,112 (width = 2,000, extended = 4,000)
Methylation
0.8
0.5
0.2
CGIs
Promoter
ENSBTAG00000026944
Exons

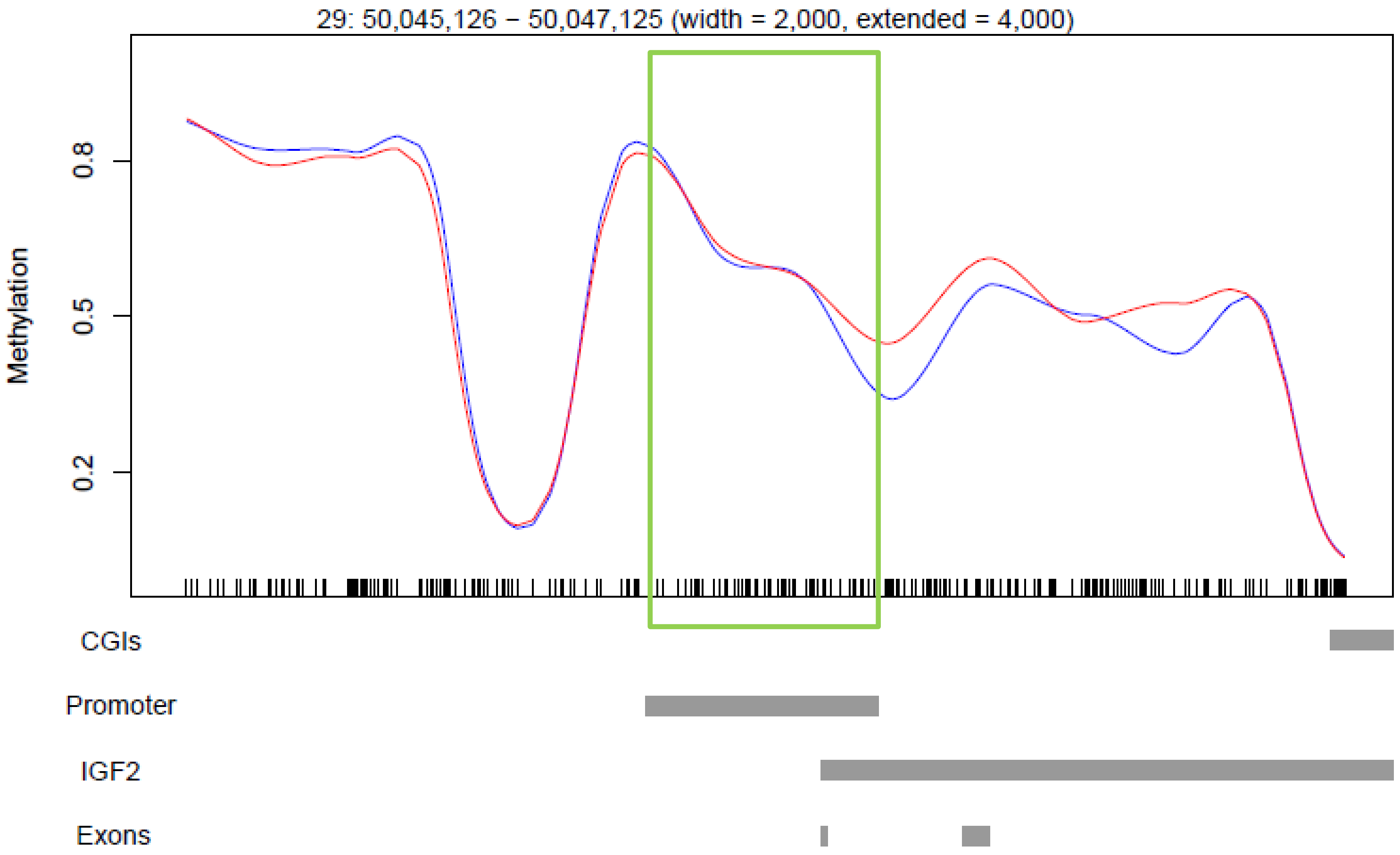
29: 50,045,126 − 50,047,125 (width = 2,000, extended = 4,000)
Methylation
0.8
0.5
0.2
CGIs
Promoter
IGF2
Exons

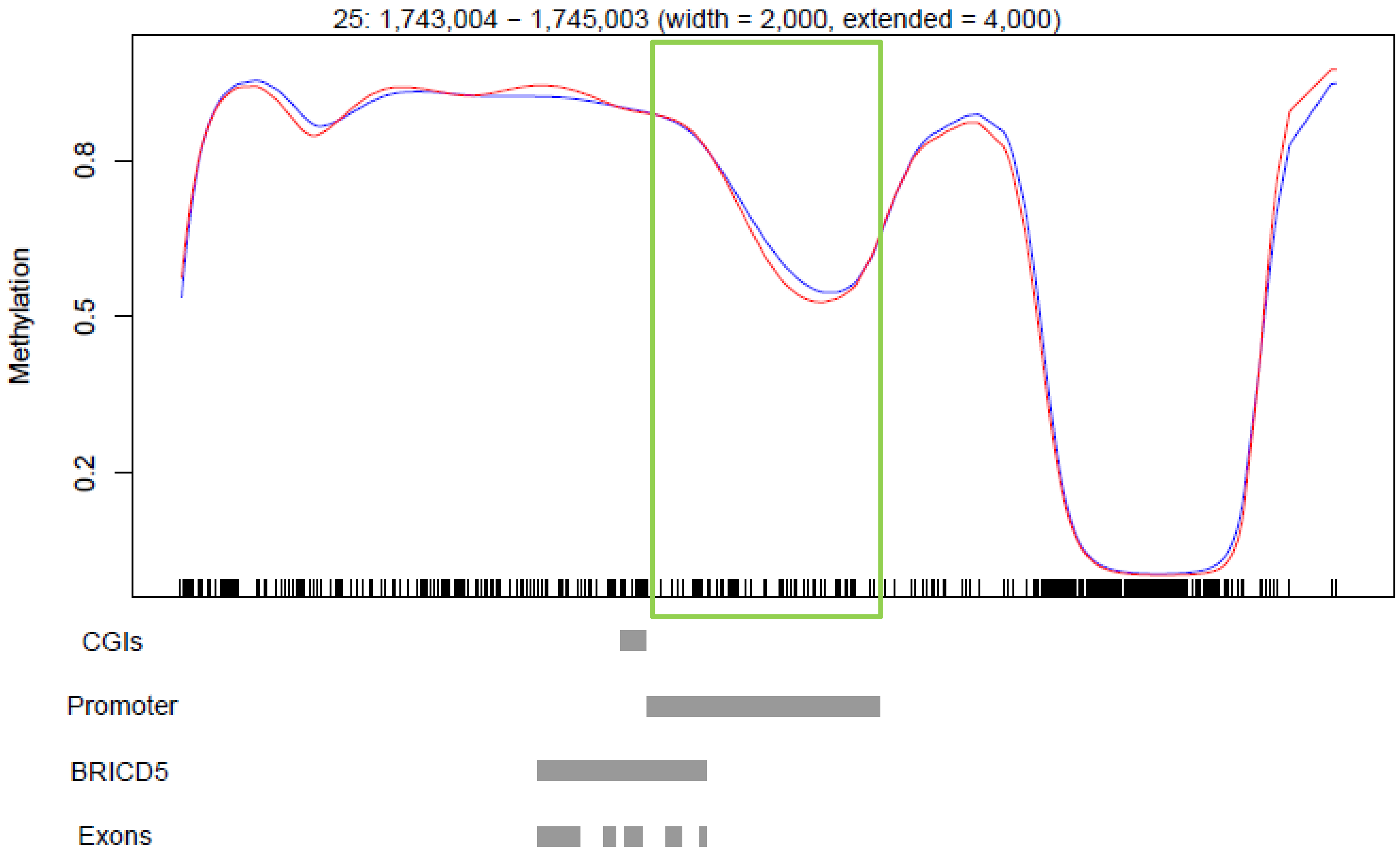
25: 1,743,004 − 1,745,003 (width = 2,000, extended = 4,000)
Methylation
0.2
0.5
0.8
CGIs
Promoter
BRICD5
Exons

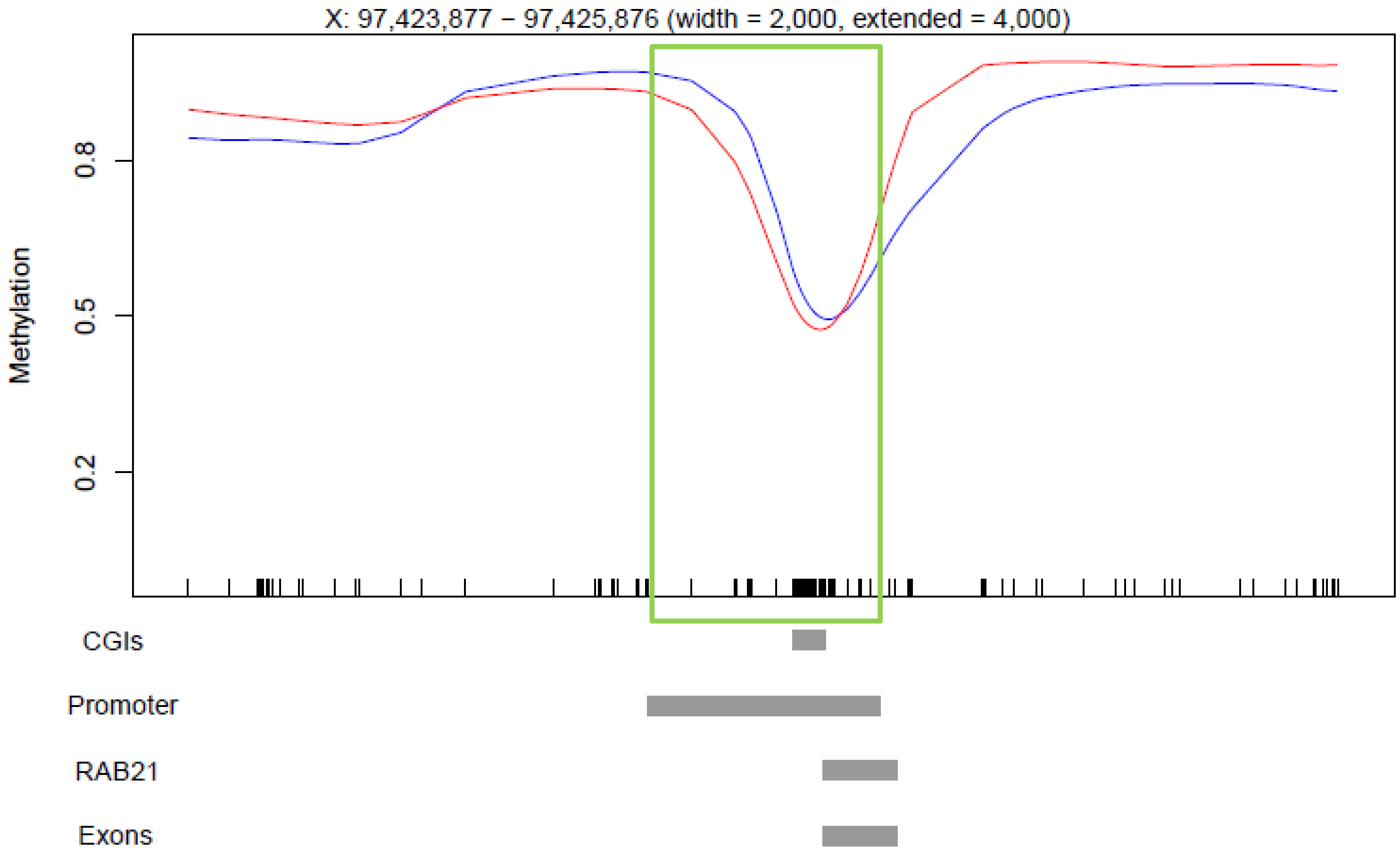
X: 97,423,877 − 97,425,876 (width = 2,000, extended = 4,000)
Methylation
0.8
0.5
0.2
CGIs
Promoter
RAB21
Exons

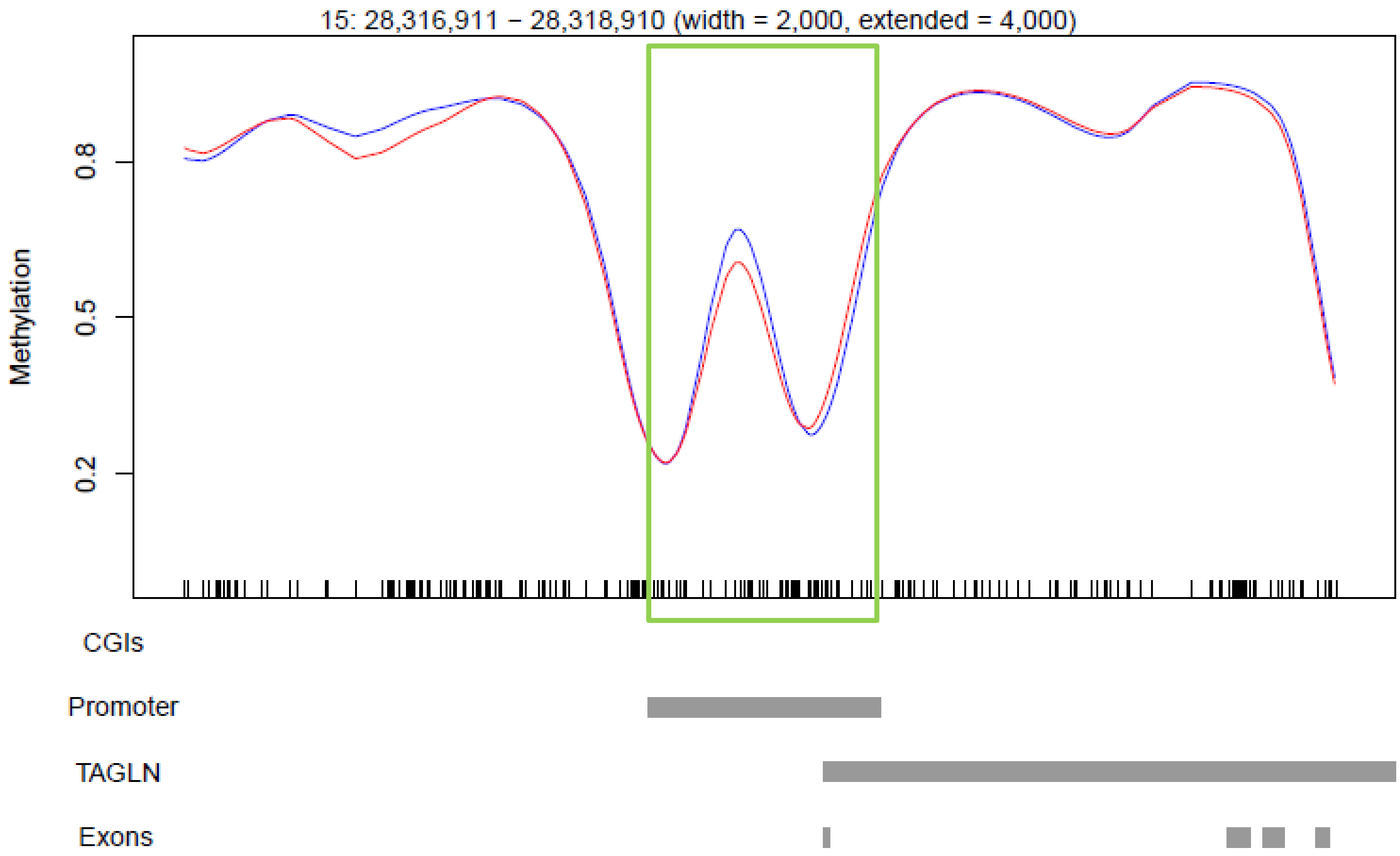

15: 28,316,911 − 28,318,910 (width = 2,000, extended = 4,000)
Methylation
0.2
0.5
0.8
CGIs
Promoter
TAGLN
Exons

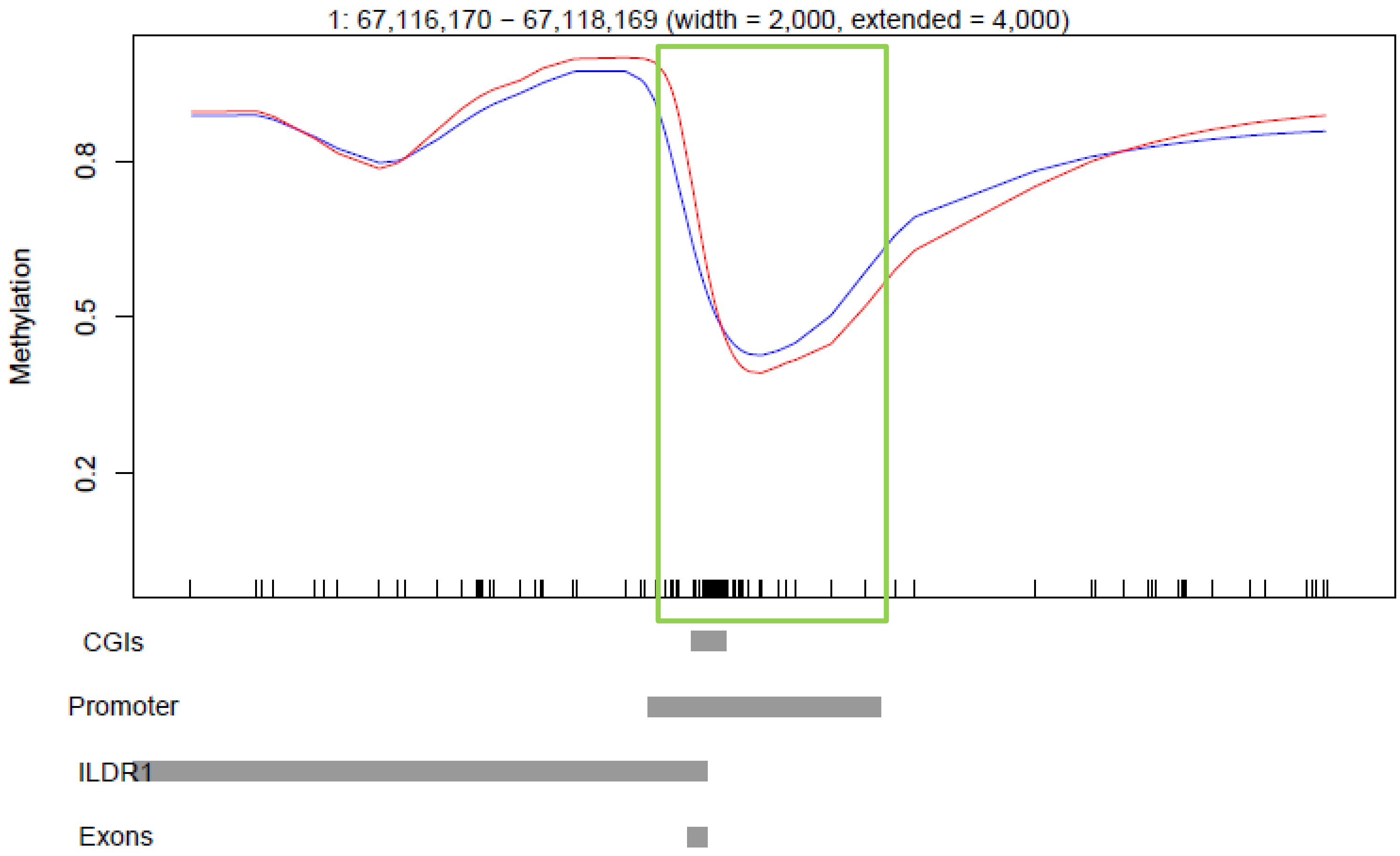

1: 67,116,170 − 67,118,169 (width = 2,000, extended = 4,000)
Methylation
0.2
0.5
0.8
CGIs
Promoter
ILDR1
Exons

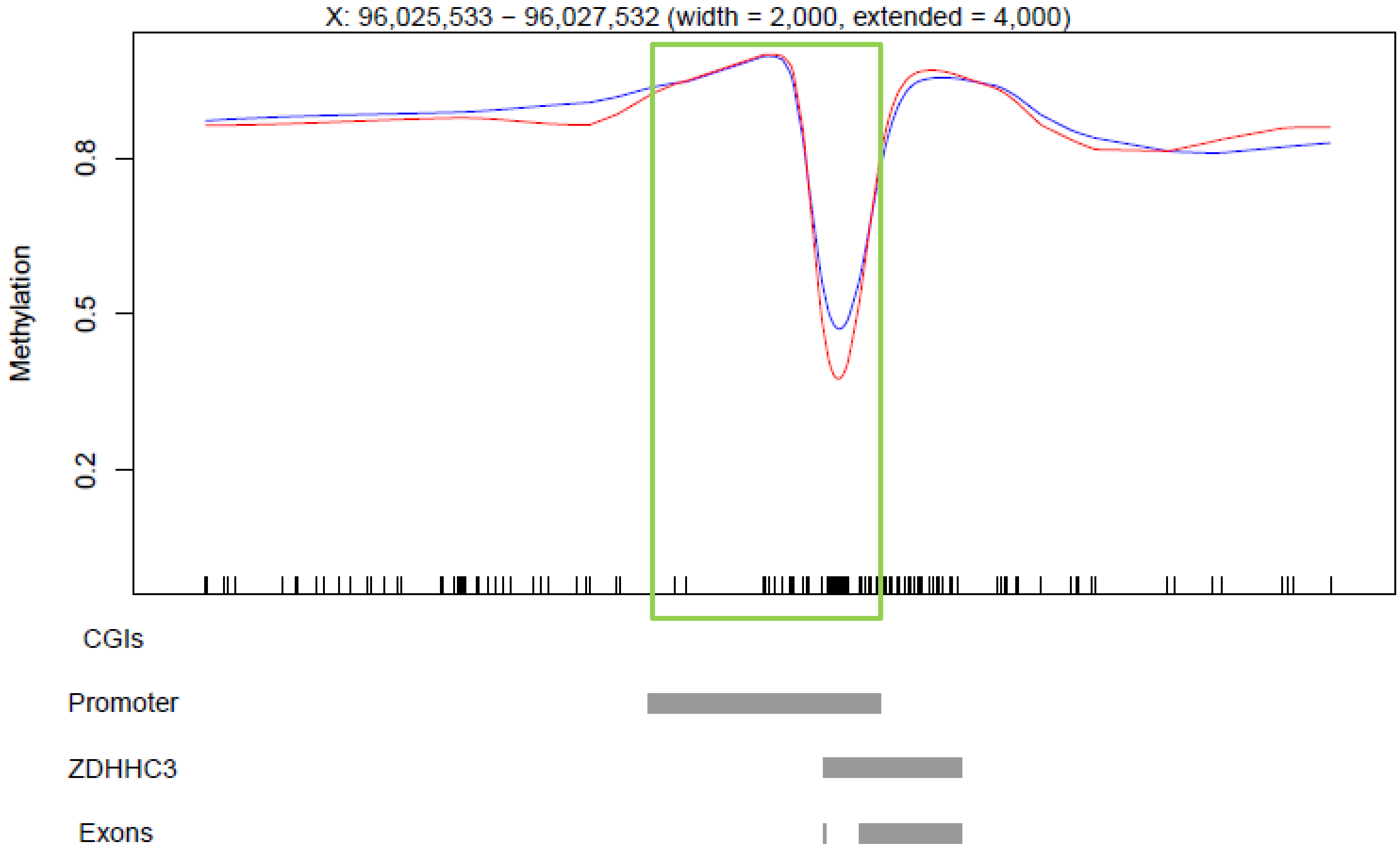

X: 96,025,533 − 96,027,532 (width = 2,000, extended = 4,000)
Methylation
0.2
0.5
0.8
CGIs
Promoter
ZDHHC3
Exons

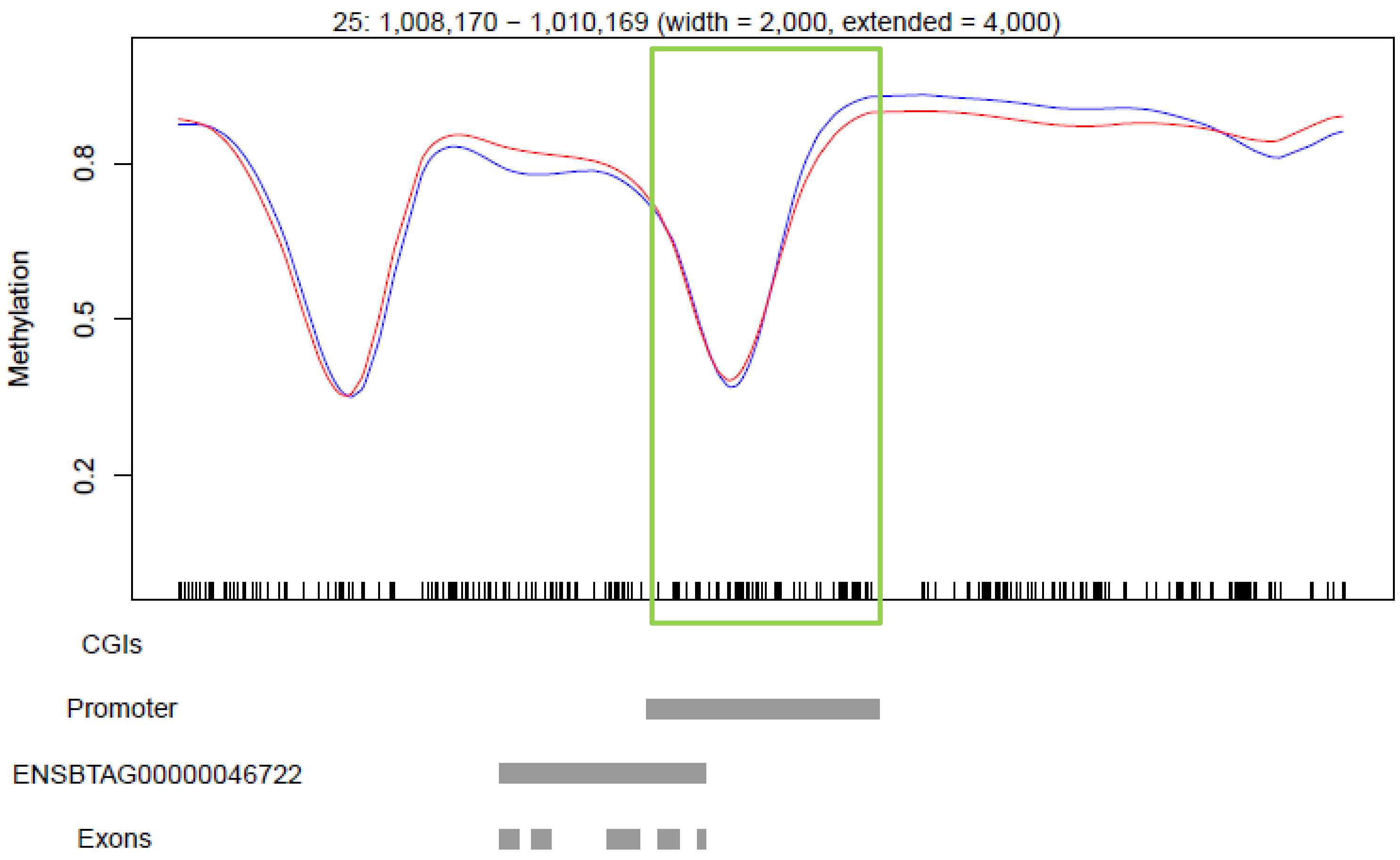
25: 1,008,170 − 1,010,169 (width = 2,000, extended = 4,000)
Methylation
0.8
0.5
0.2
CGIs
Promoter
ENSBTAG00000046722
Exons

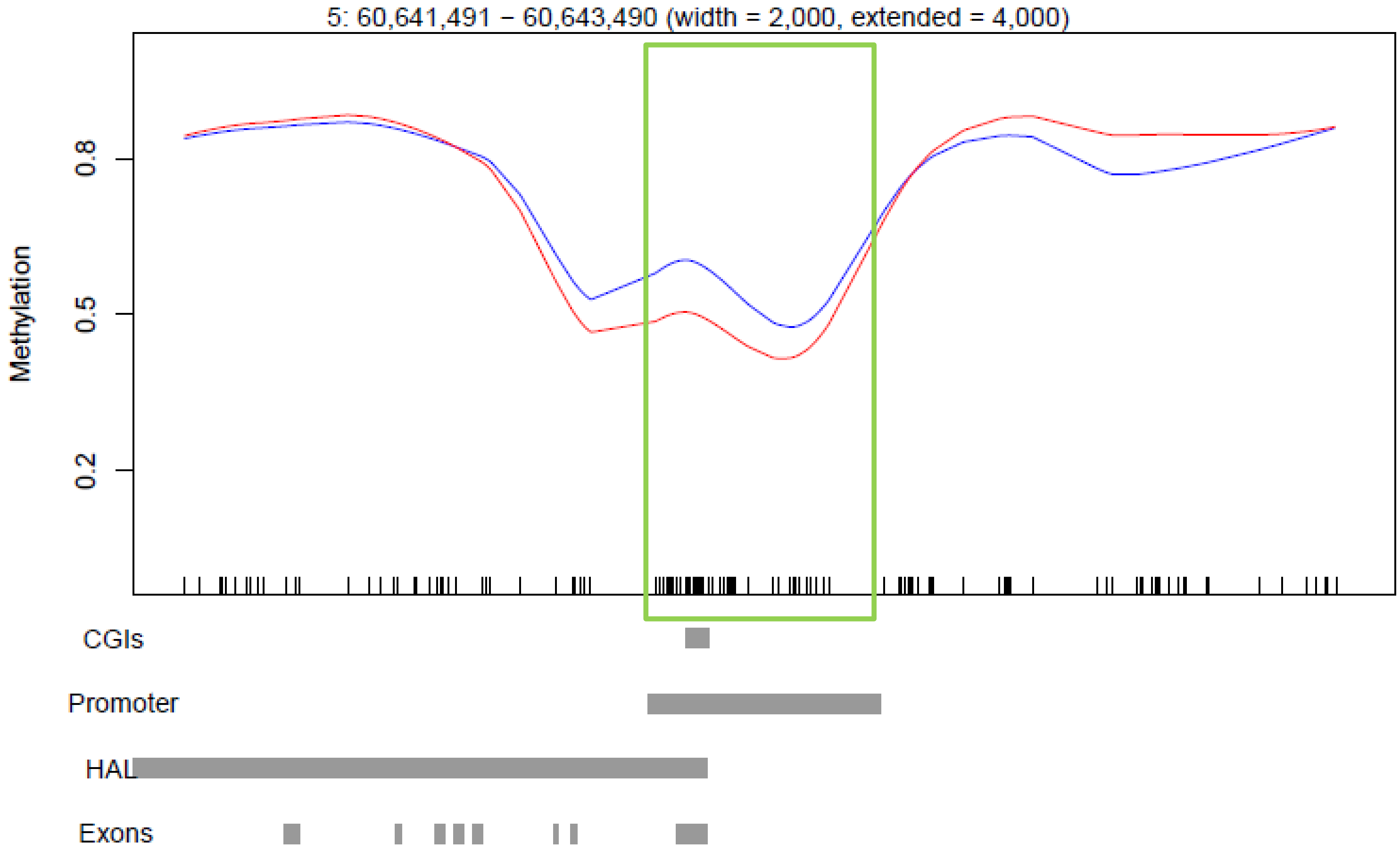

5: 60,641,491 − 60,643,490 (width = 2,000, extended = 4,000)
Methylation
0.8
0.5
0.2
CGIs
Promoter
HAL
Exons

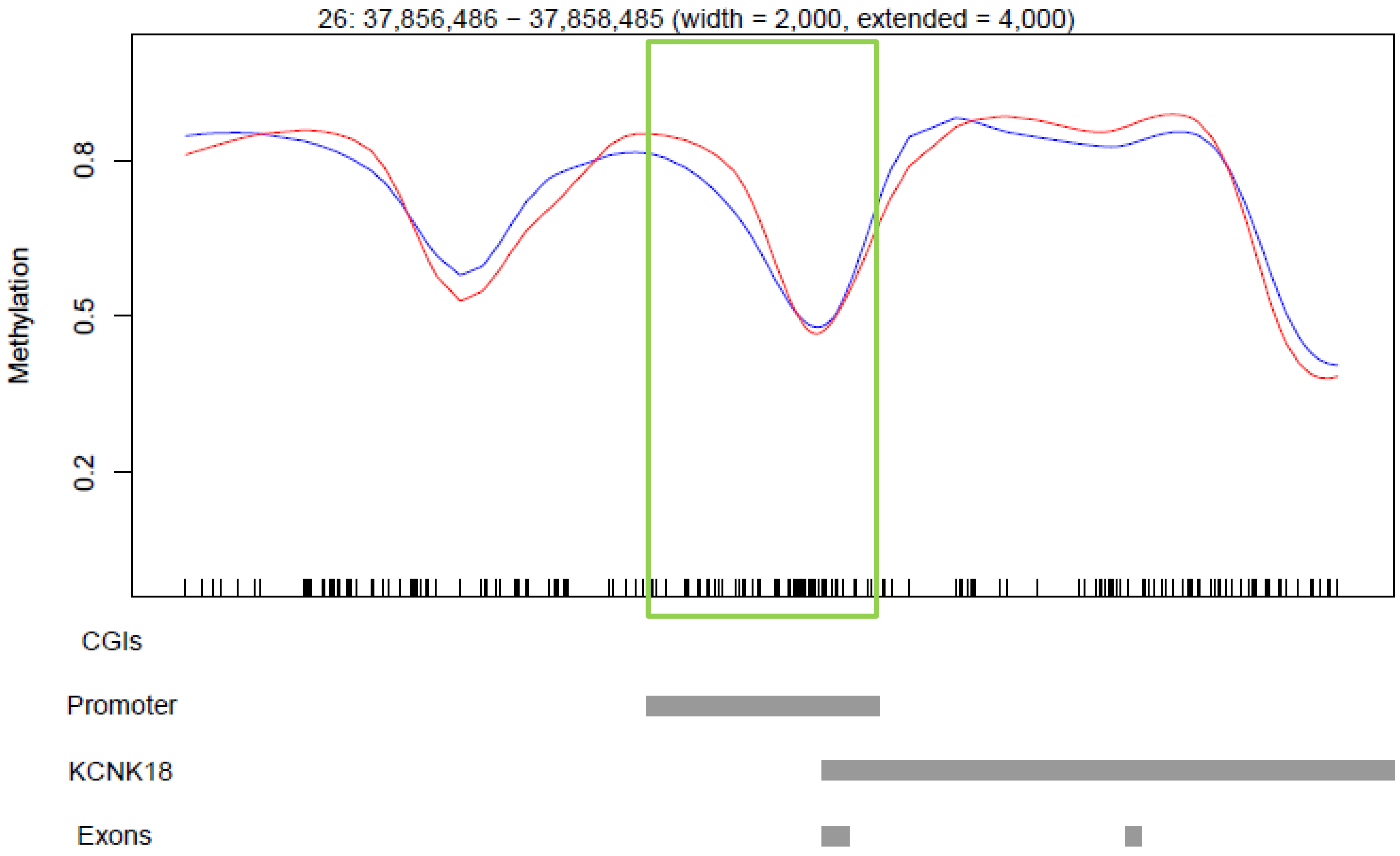
26: 37,856,486 – 37,858,485 (width = 2,000, extended = 4,000)
Methylation
0.8
0.5
0.2
CGIs
Promoter
KCNK18
Exons

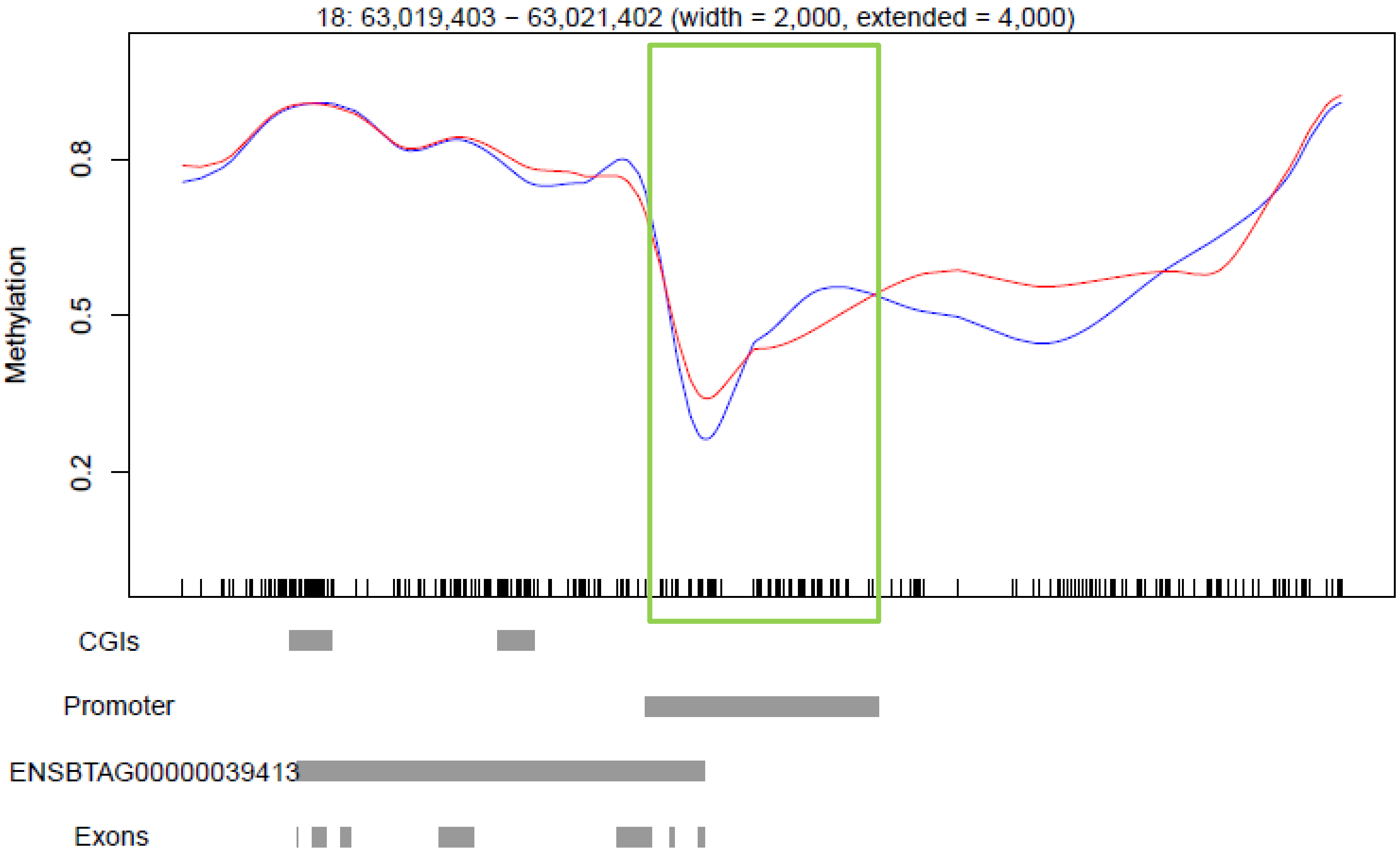
18: 63,019,403 − 63,021,402 (width = 2,000, extended = 4,000)
Methylation
0.2
0.5
0.8
CGIs
Promoter
ENSBTAG00000039413
Exons

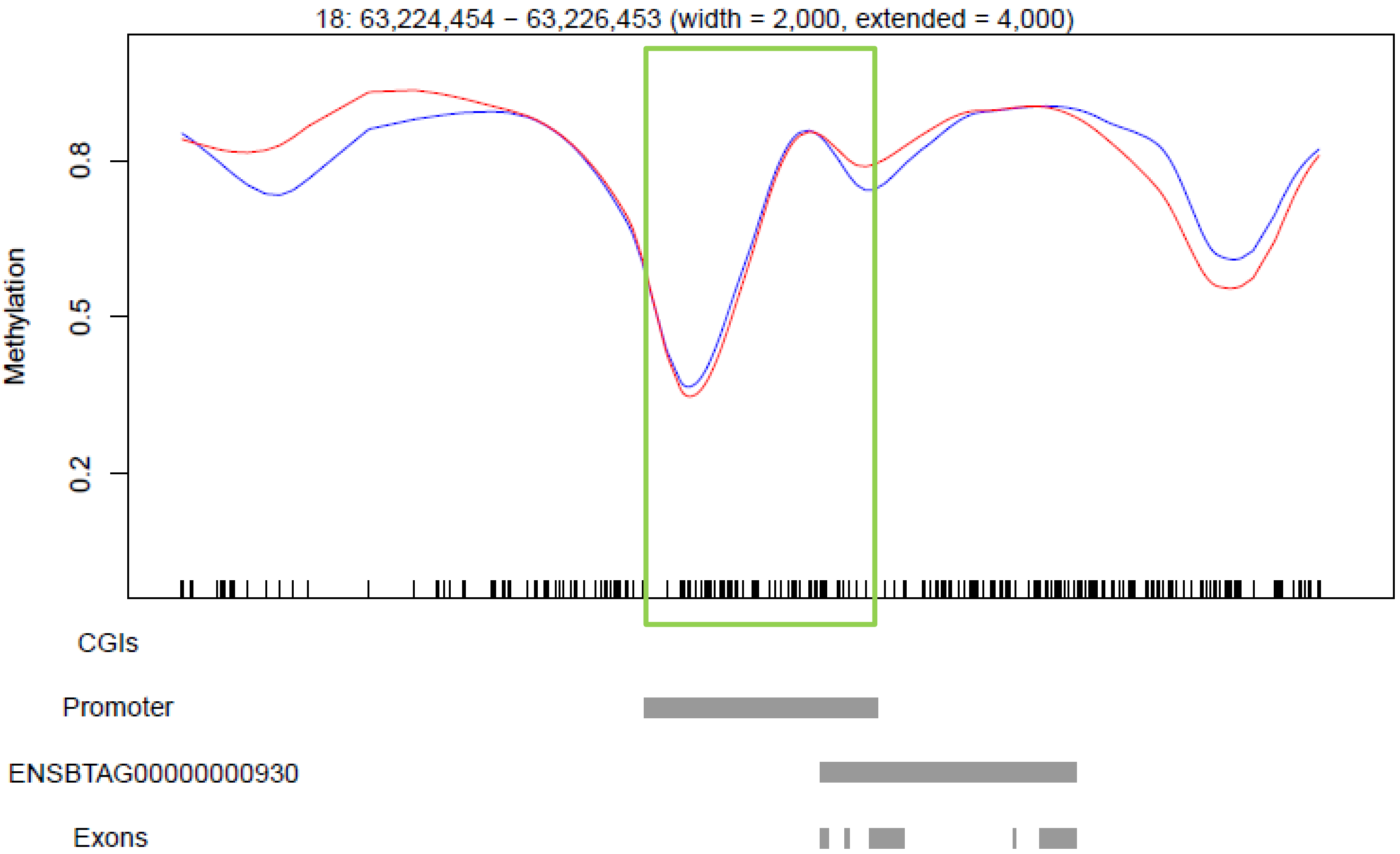

18: 63,224,454 − 63,226,453 (width = 2,000, extended = 4,000)
Methylation
0.2
0.5
0.8
CGIs
Promoter
ENSBTAG00000000930
Exons

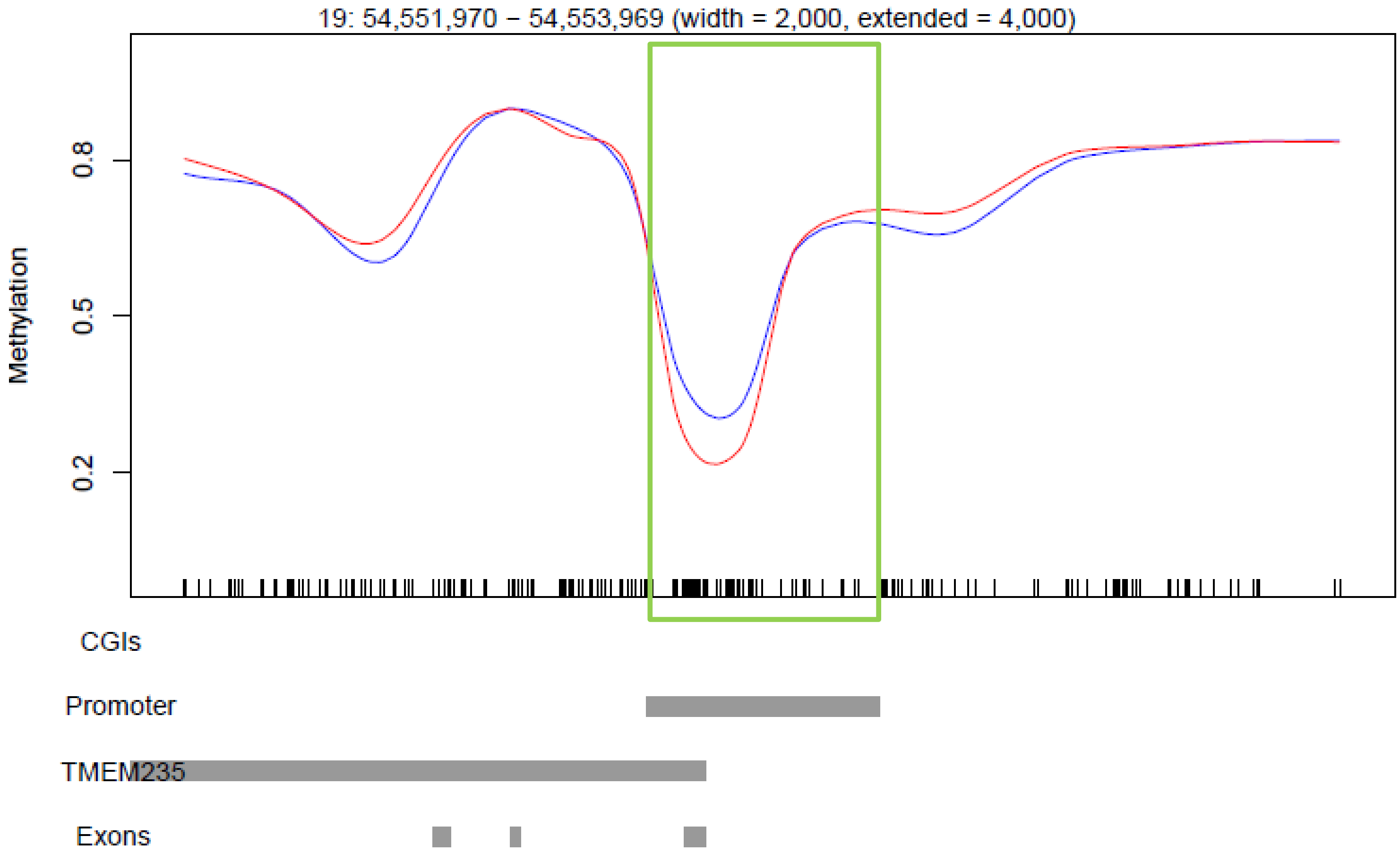
19: 54,551,970 – 54,553,969 (width = 2,000, extended = 4,000)
Methylation
0.8
0.5
0.2
CGIs
Promoter
TMEM235
Exons

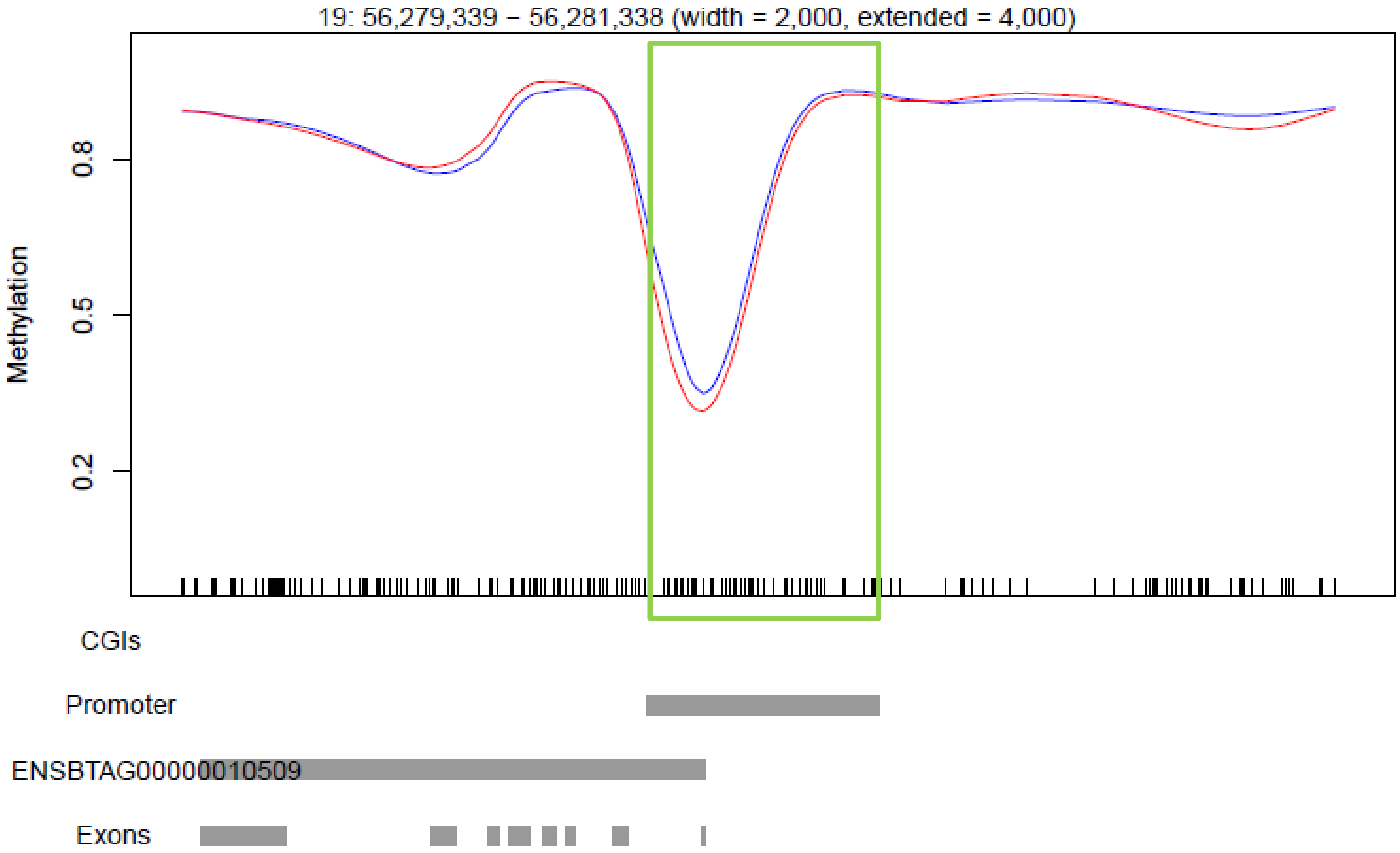
19: 56,279,339 – 56,281,338 (width = 2,000, extended = 4,000)
Methylation
0.2
0.5
0.8
CGIs
Promoter
ENSBTAG00000010509
Exons

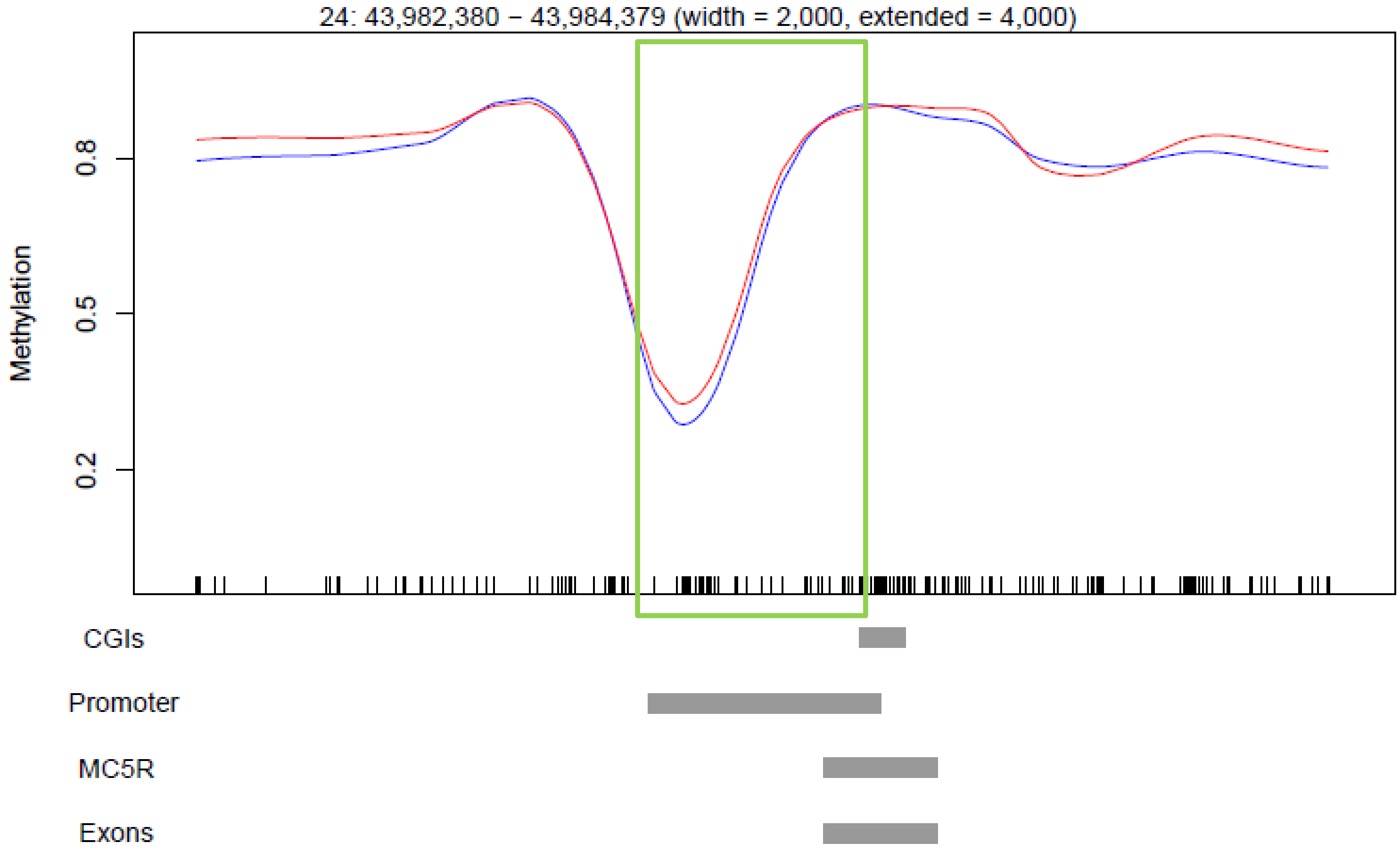

24: 43,982,380 − 43,984,379 (width = 2,000, extended = 4,000)
Methylation
0.2
0.5
0.8
CGIs
Promoter
MC5R
Exons

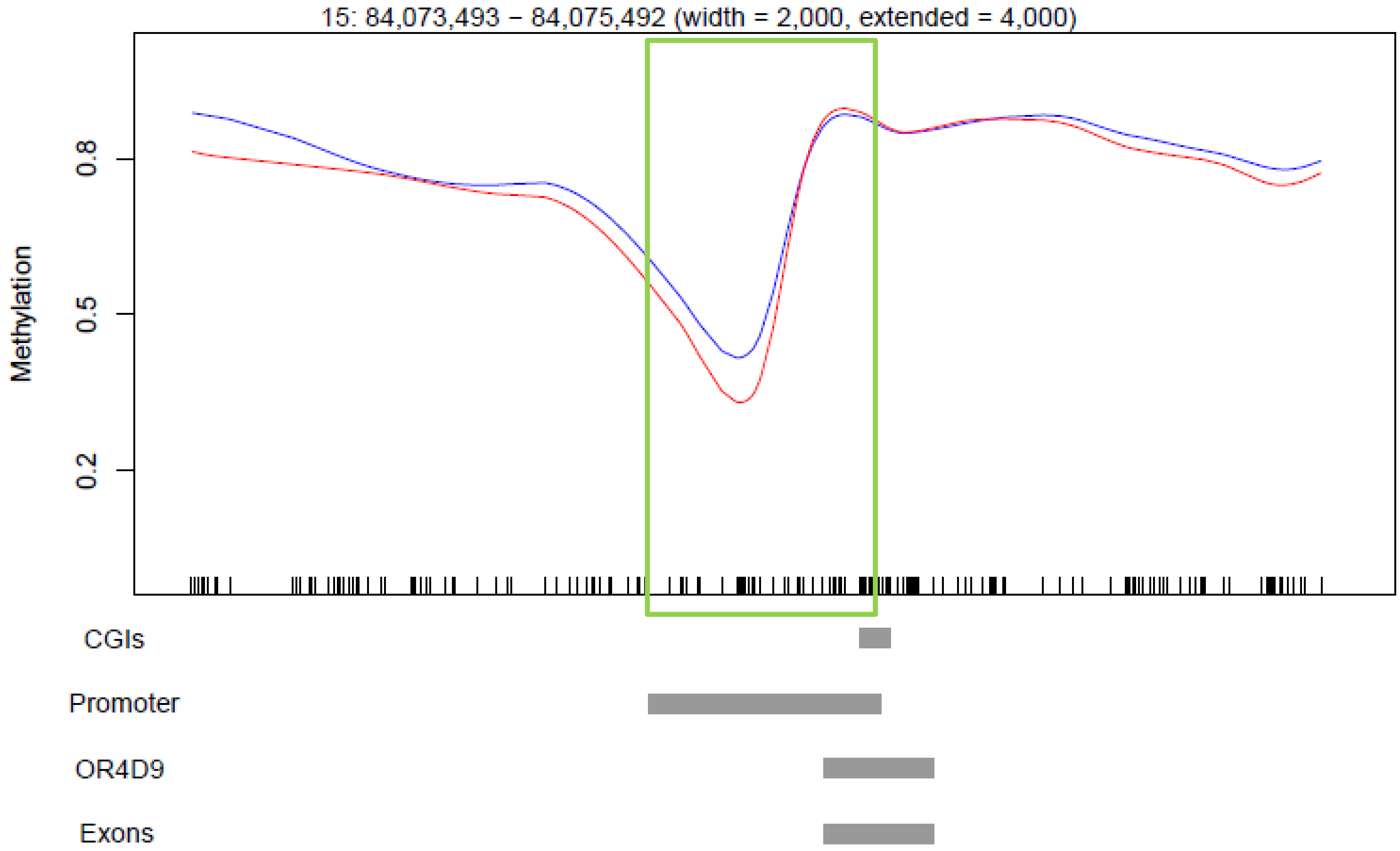

15: 84,073,493 − 84,075,492 (width = 2,000, extended = 4,000)
Methylation
0.8
0.5
0.2
CGIs
Promoter
OR4D9
Exons

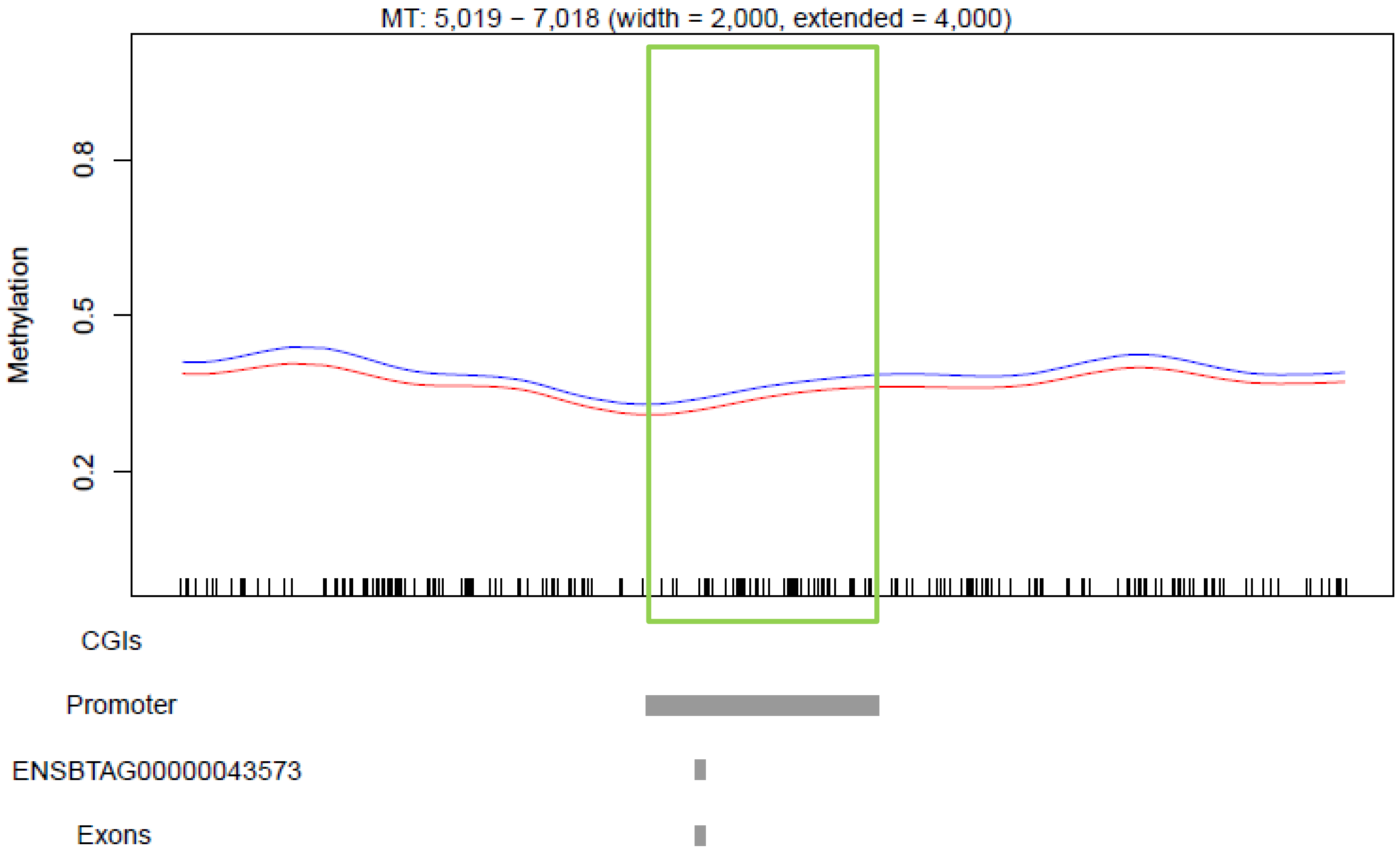
MT: 5,019 − 7,018 (width = 2,000, extended = 4,000)
Methylation
0.8
0.5
0.2
CGIs
Promoter
ENSBTAG00000043573
Exons

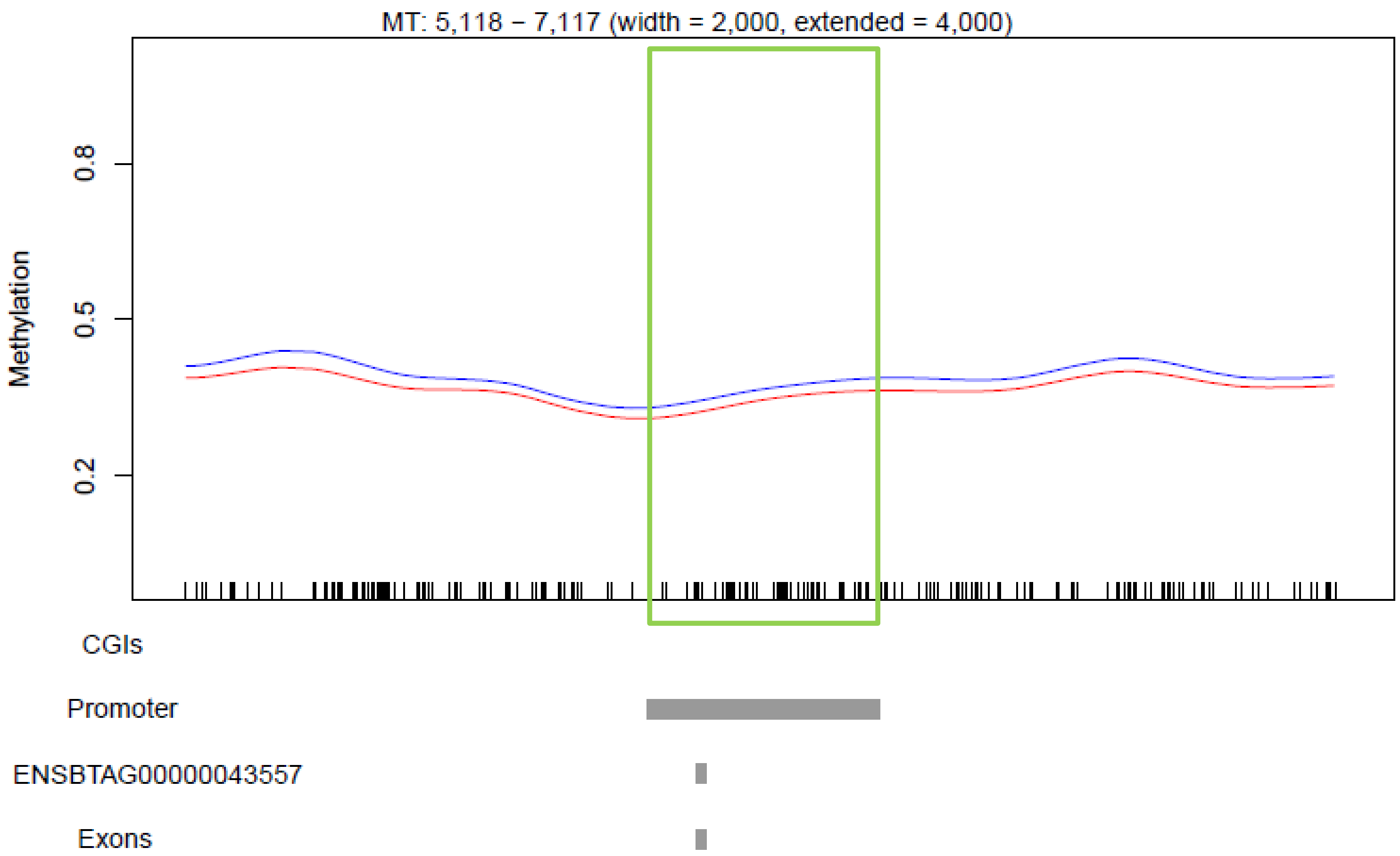
MT: 5,118 – 7,117 (width = 2,000, extended = 4,000)
Methylation
0.8
0.5
0.2
CGIs
Promoter
ENSBTAG00000043557
Exons

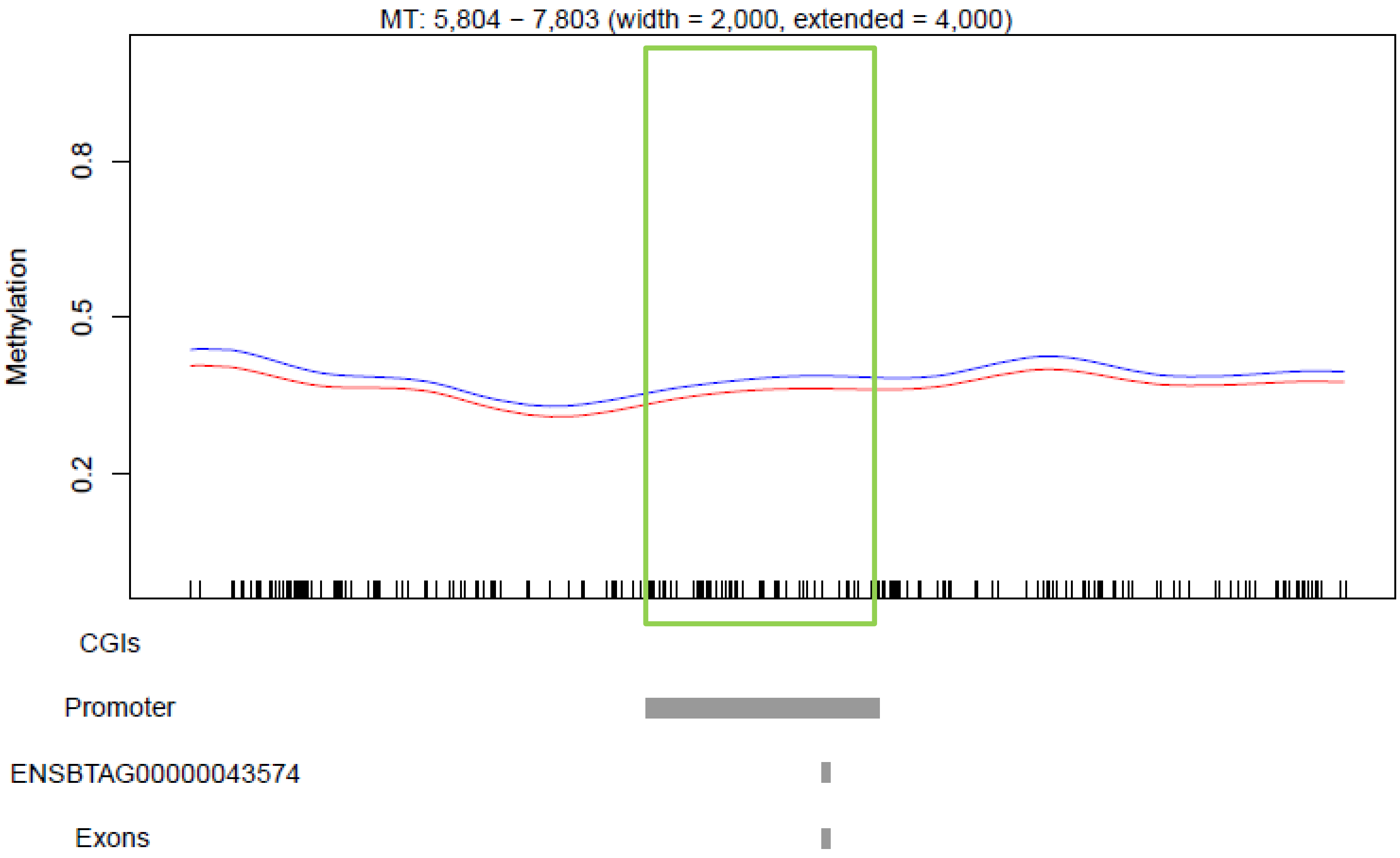

MT: 5,804 − 7,803 (width = 2,000, extended = 4,000)
Methylation
0.8
0.5
0.2
CGIs
Promoter
ENSBTAG00000043574
Exons

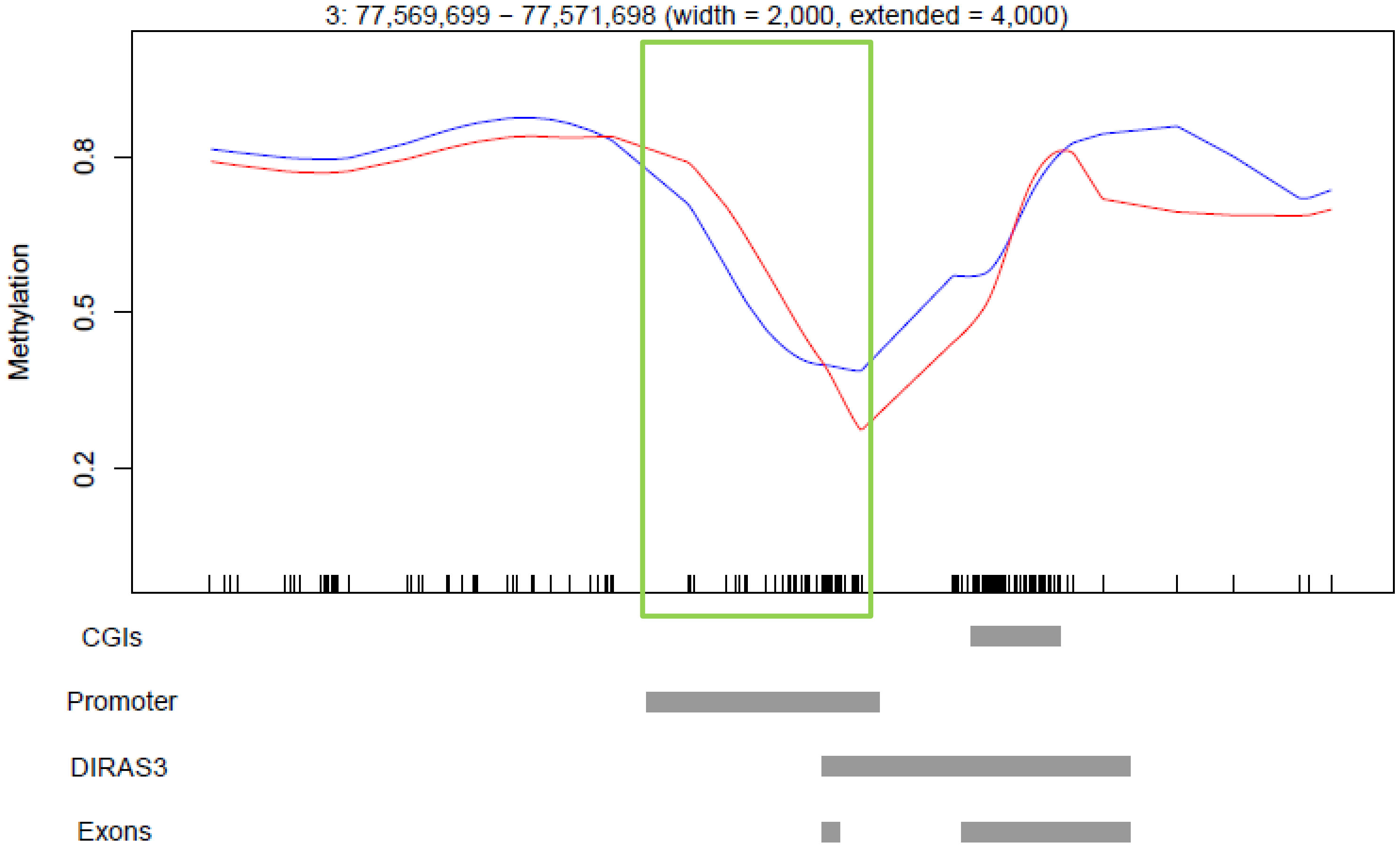
3: 77,569,699 – 77,571,698 (width = 2,000, extended = 4,000)
Methylation
0.8
0.5
0.2
CGIs
Promoter
DIRAS3
Exons

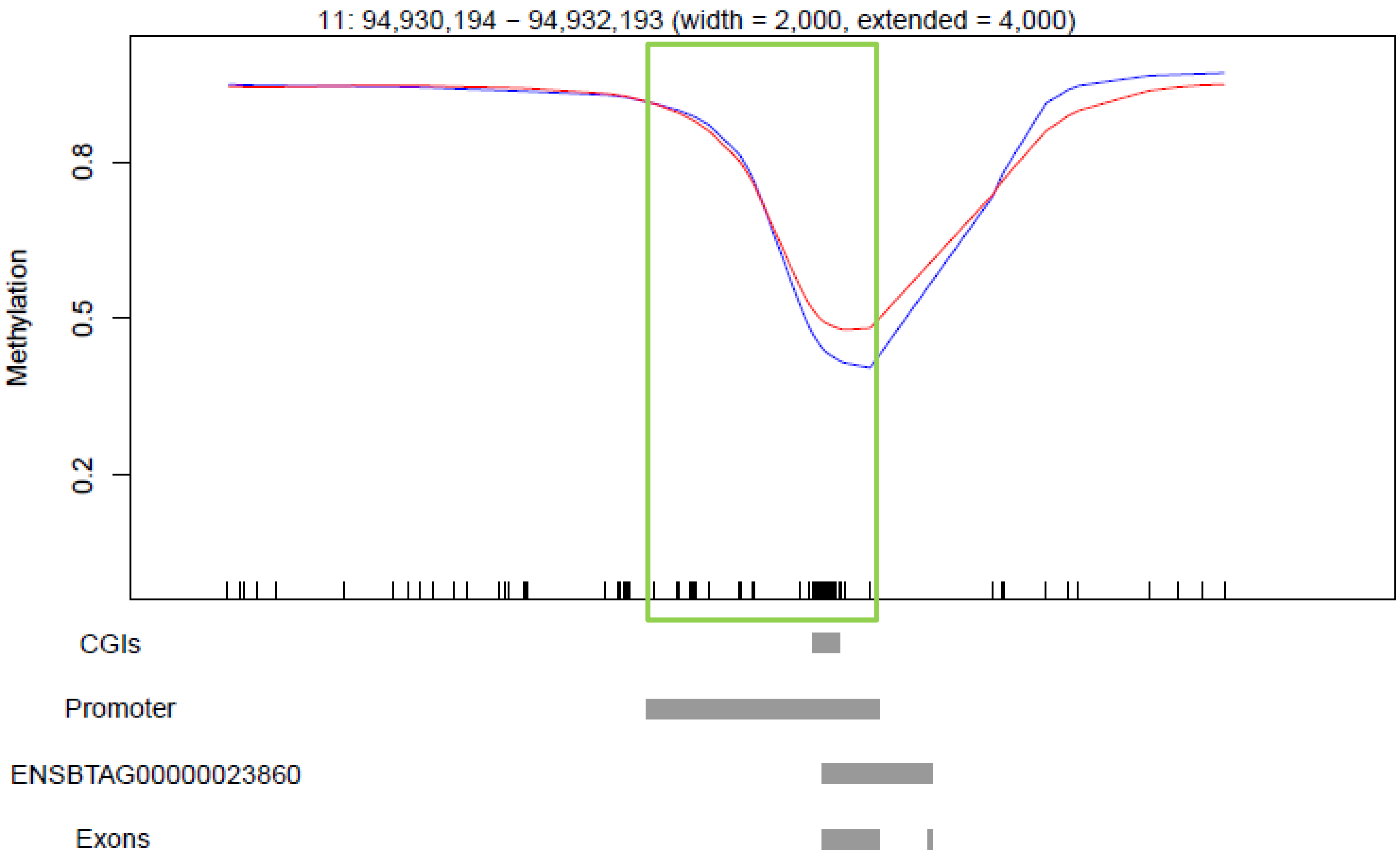

11: 94,930,194 − 94,932,193 (width = 2,000, extended = 4,000)
Methylation
0.8
0.5
0.2
CGIs
Promoter
ENSBTAG00000023860
Exons

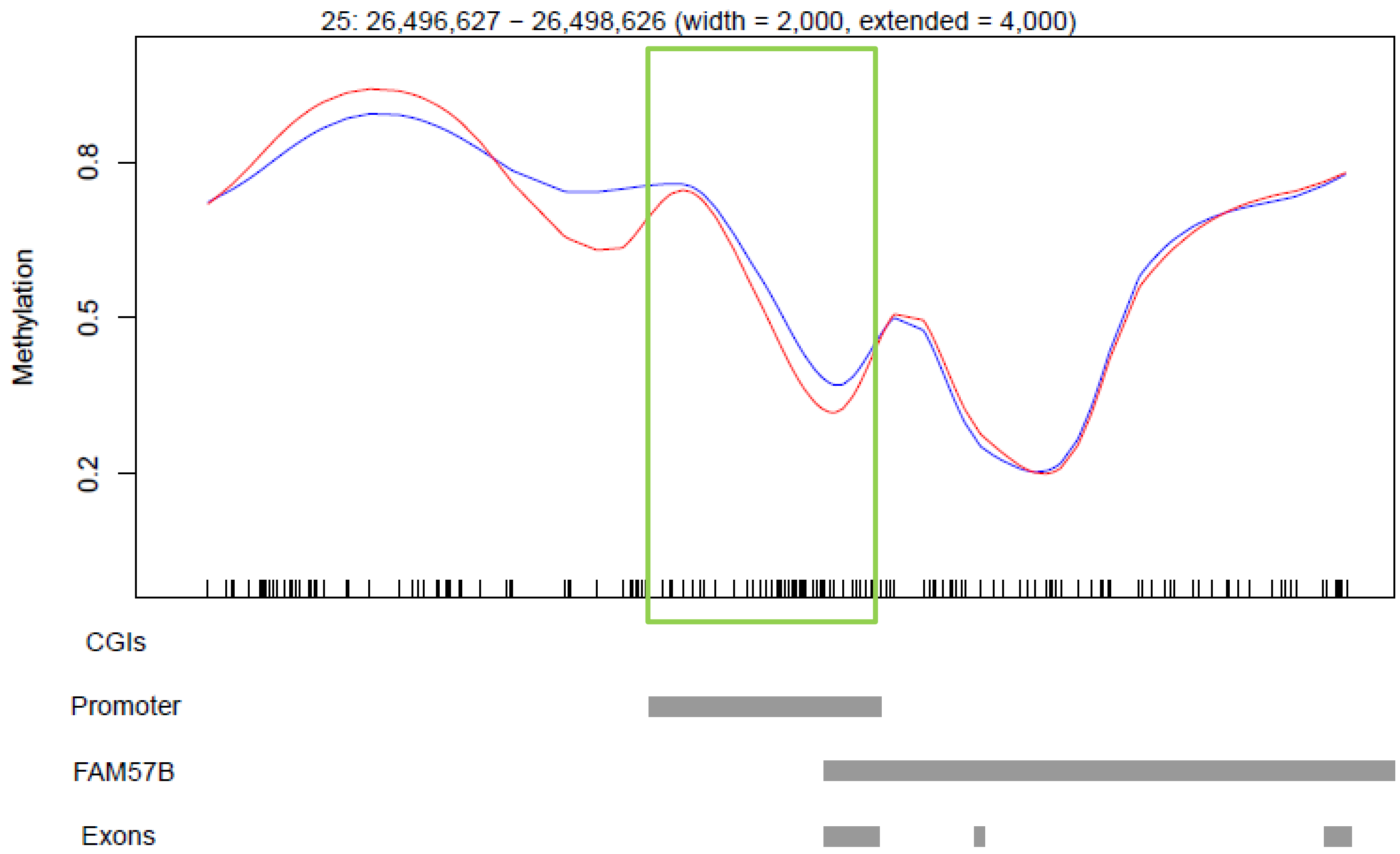
25: 26,496,627 − 26,498,626 (width = 2,000, extended = 4,000)
Methylation
0.8
0.5
0.2
CGIs
Promoter
FAM57B
Exons

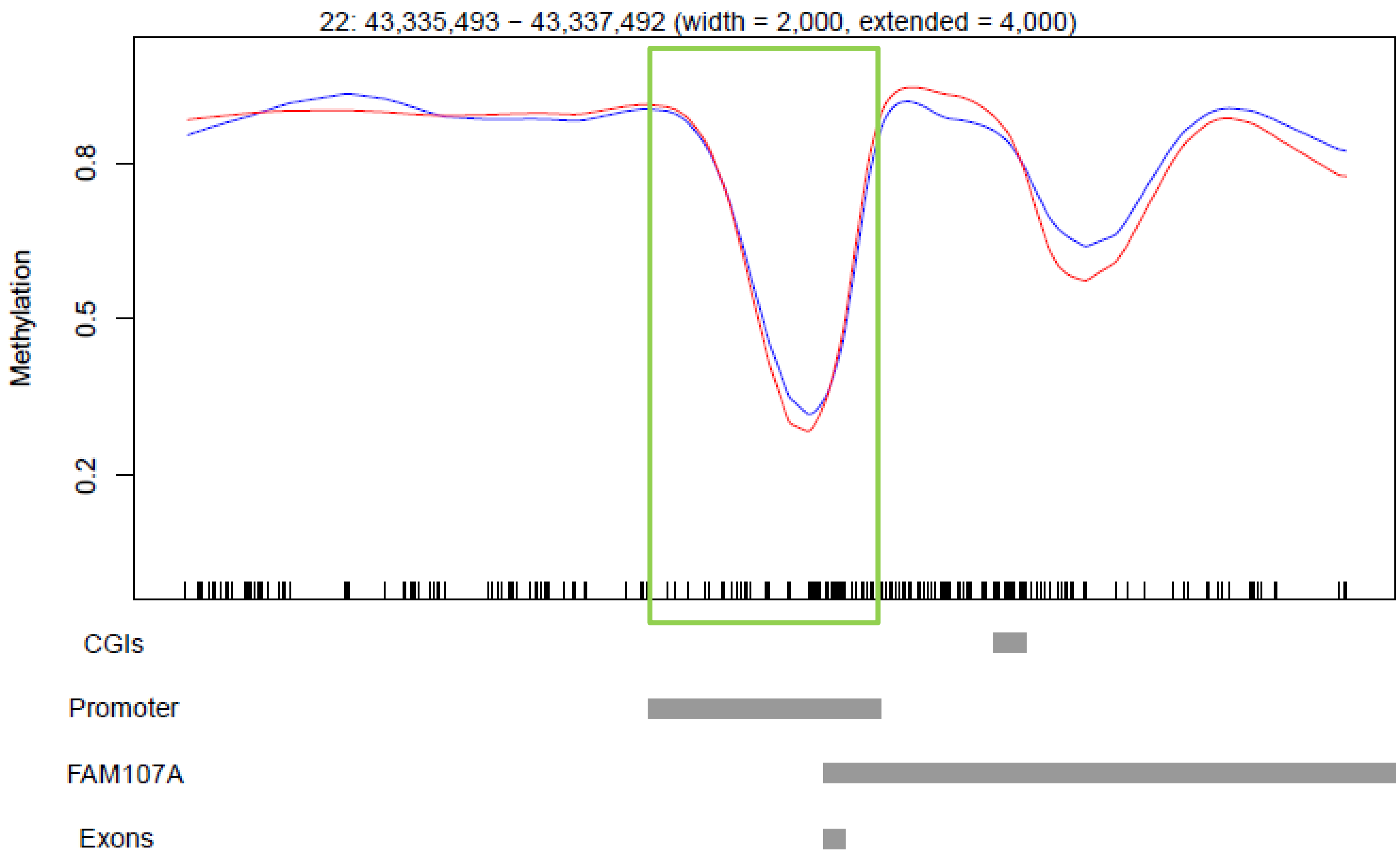
22: 43,335,493 – 43,337,492 (width = 2,000, extended = 4,000)
Methylation
0.8
0.5
0.2
CGIs
Promoter
FAM107A
Exons

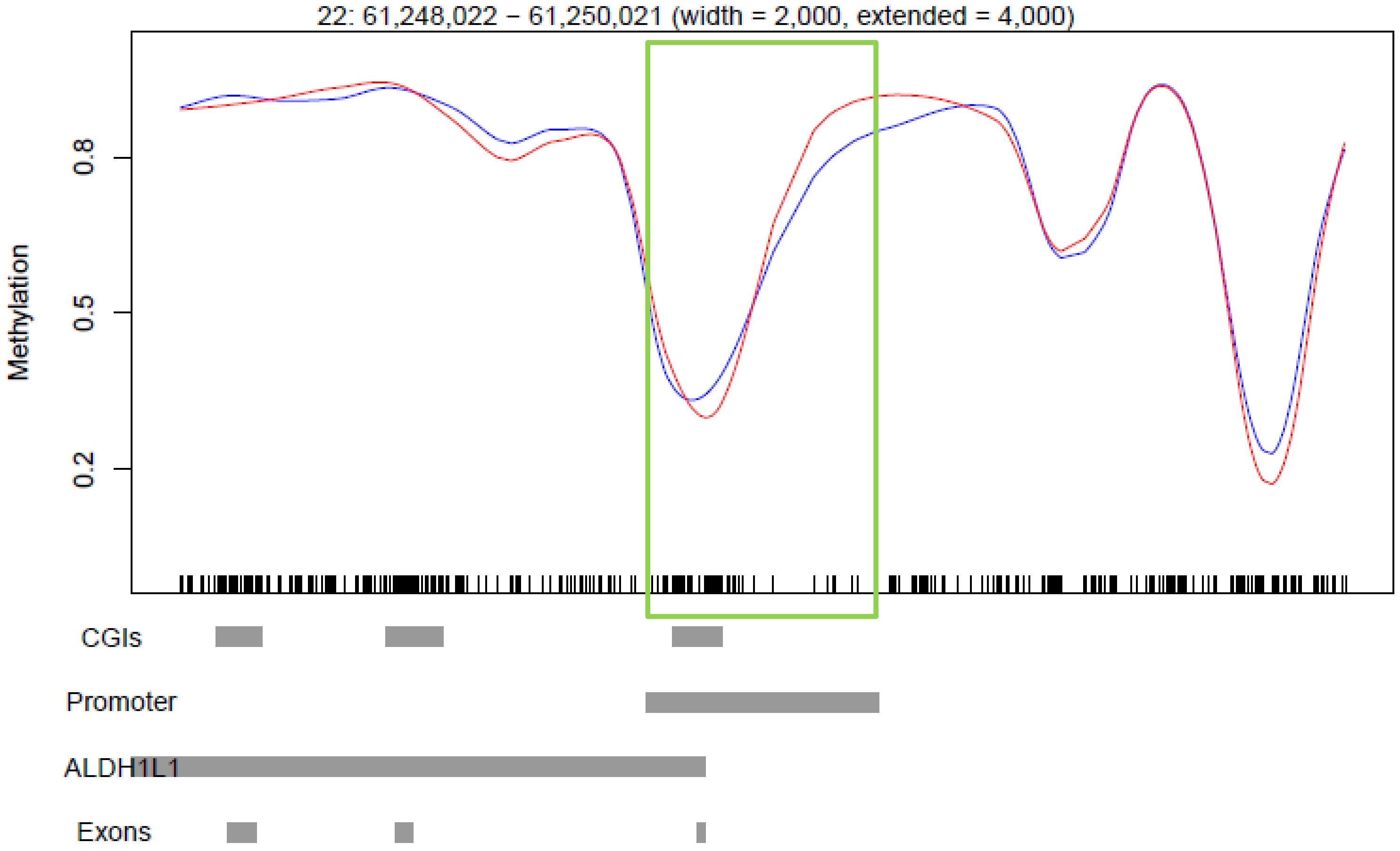

22: 61,248,022 − 61,250,021 (width = 2,000, extended = 4,000)
Methylation
0.8
0.5
0.2
CGIs
Promoter
ALDH1L1
Exons

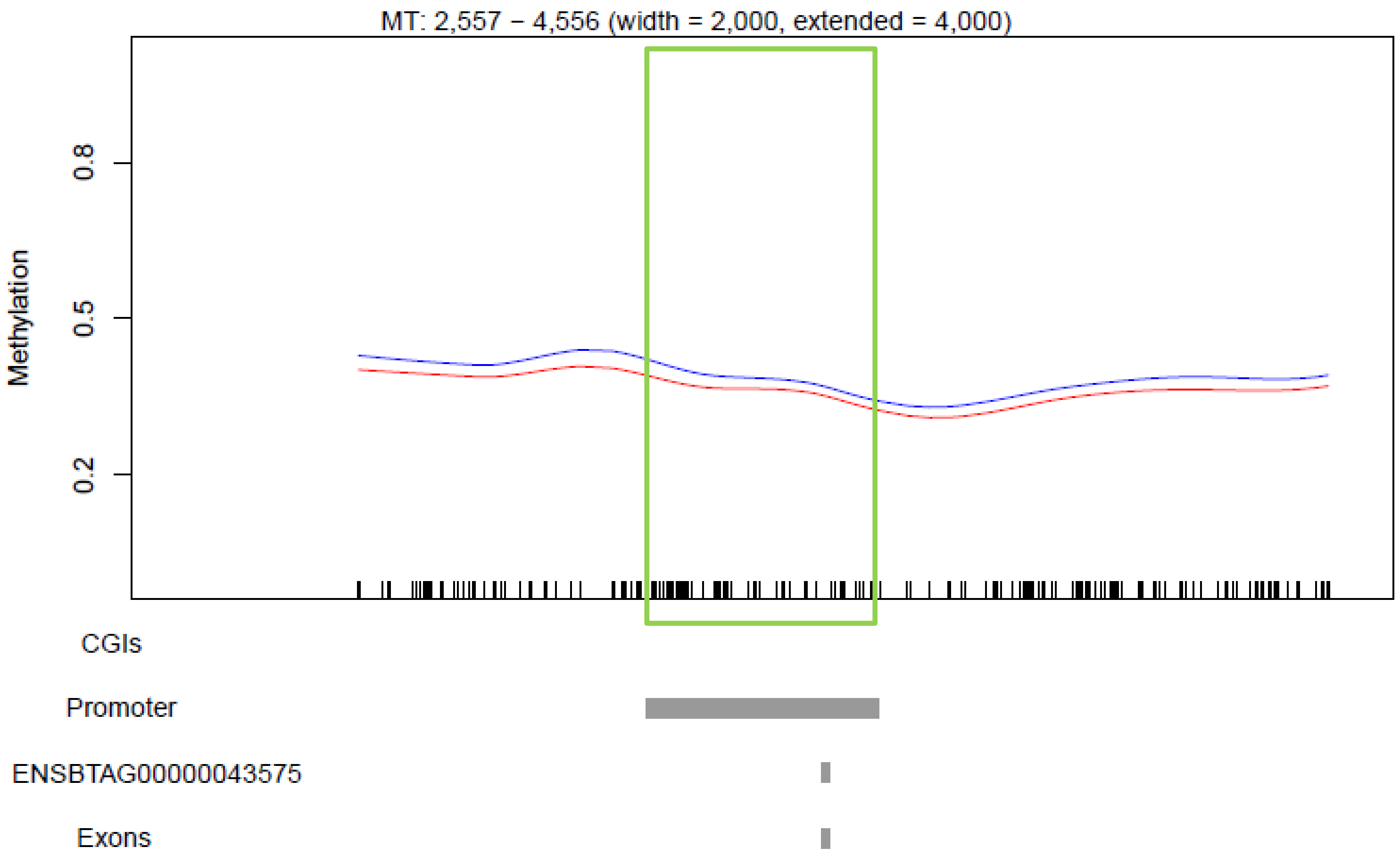
MT: 2,557 − 4,556 (width = 2,000, extended = 4,000)
Methylation
0.8
0.5
0.2
CGIs
Promoter
ENSBTAG00000043575
Exons

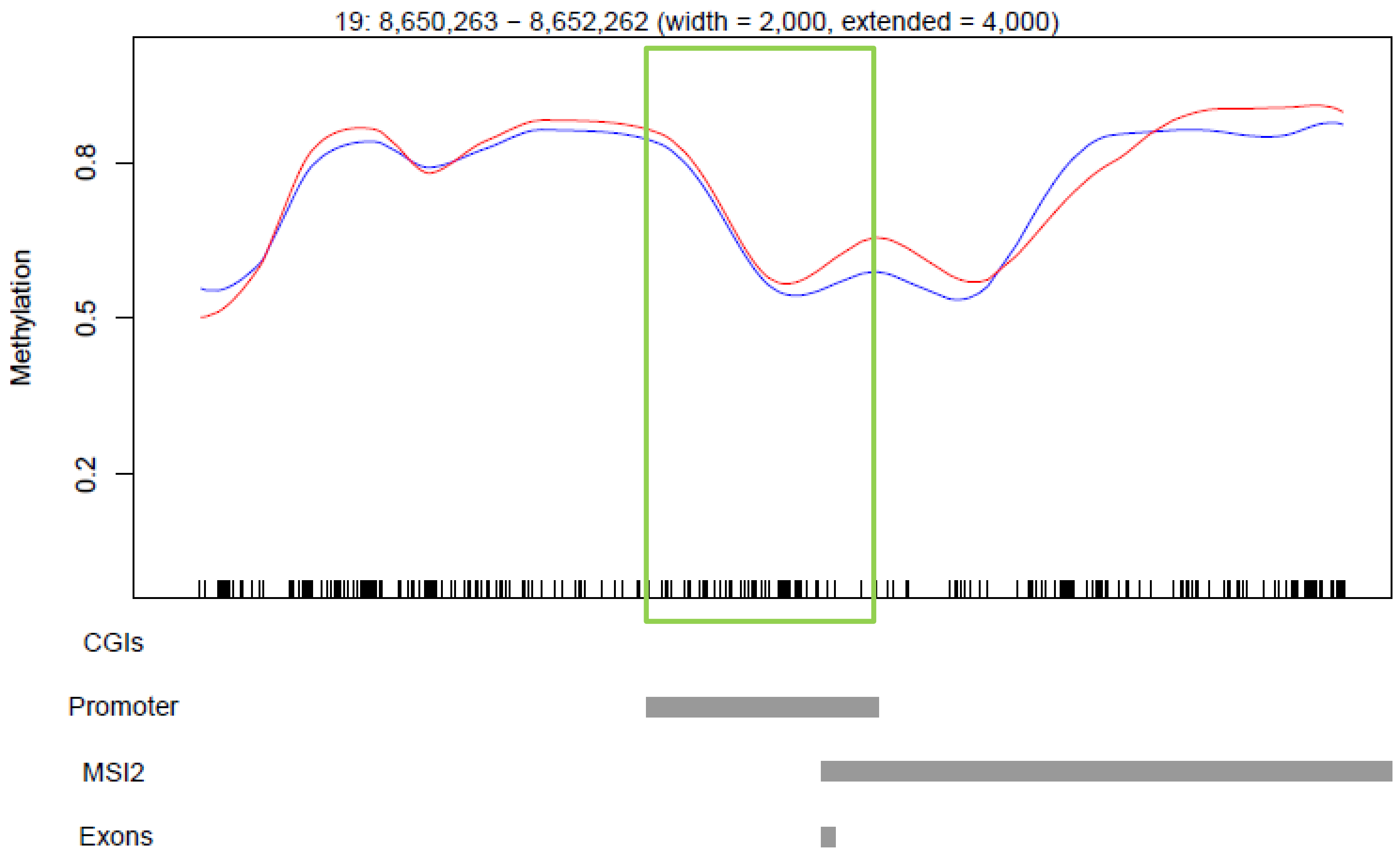

19: 8,650,263 – 8,652,262 (width = 2,000, extended = 4,000)
Methylation
0.8
0.5
0.2
CGIs
Promoter
MSI2
Exons

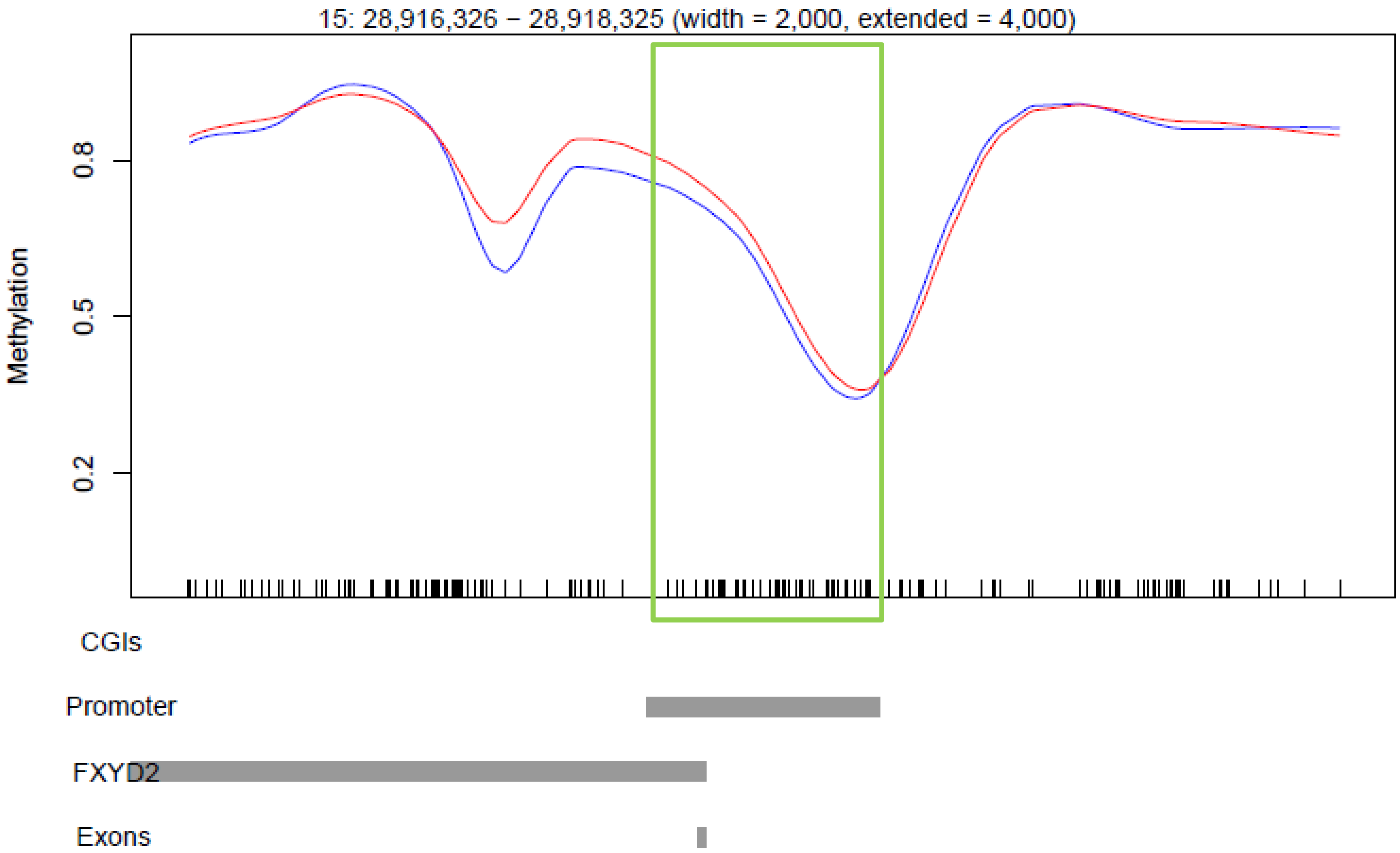
15: 28,916,326 − 28,918,325 (width = 2,000, extended = 4,000)
Methylation
0.8
0.5
0.2
CGIs
Promoter
FXYD2
Exons

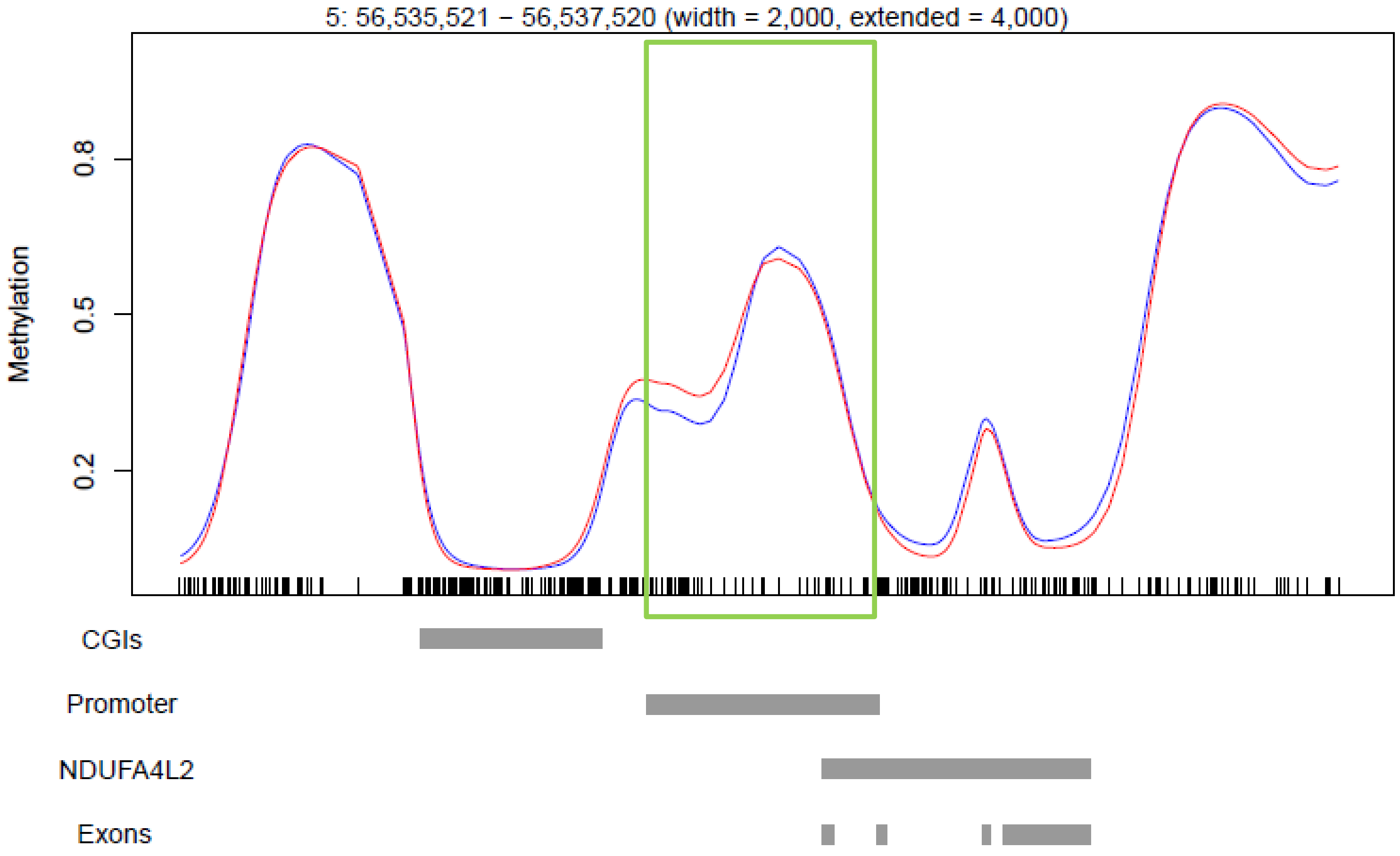
5: 56,535,521 − 56,537,520 (width = 2,000, extended = 4,000)
Methylation
0.8
0.5
0.2
CGIs
Promoter
NDUFA4L2
Exons

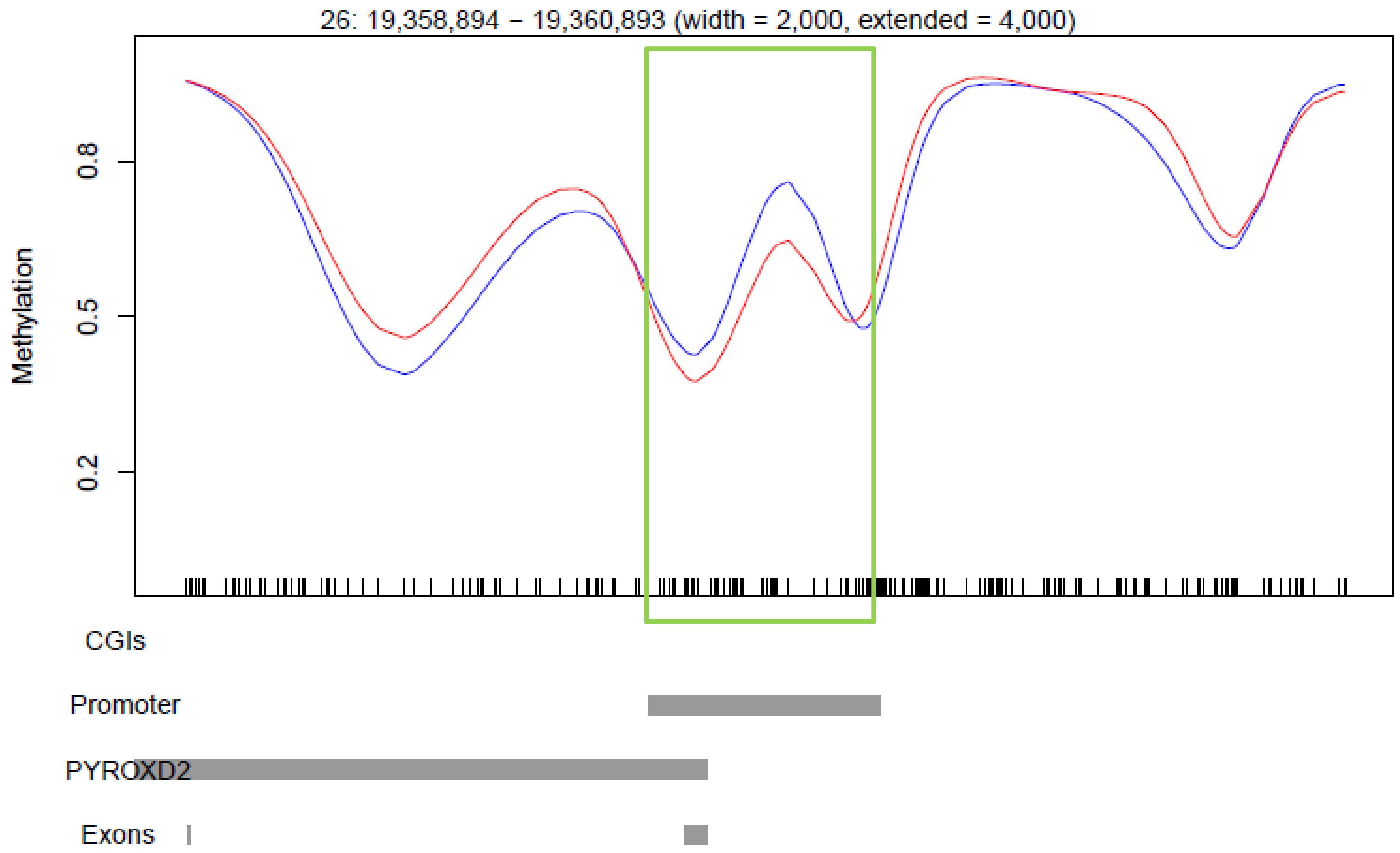

26: 19,358,894 − 19,360,893 (width = 2,000, extended = 4,000)
Methylation
0.2
0.5
0.8
CGIs
Promoter
PYROXD2
Exons

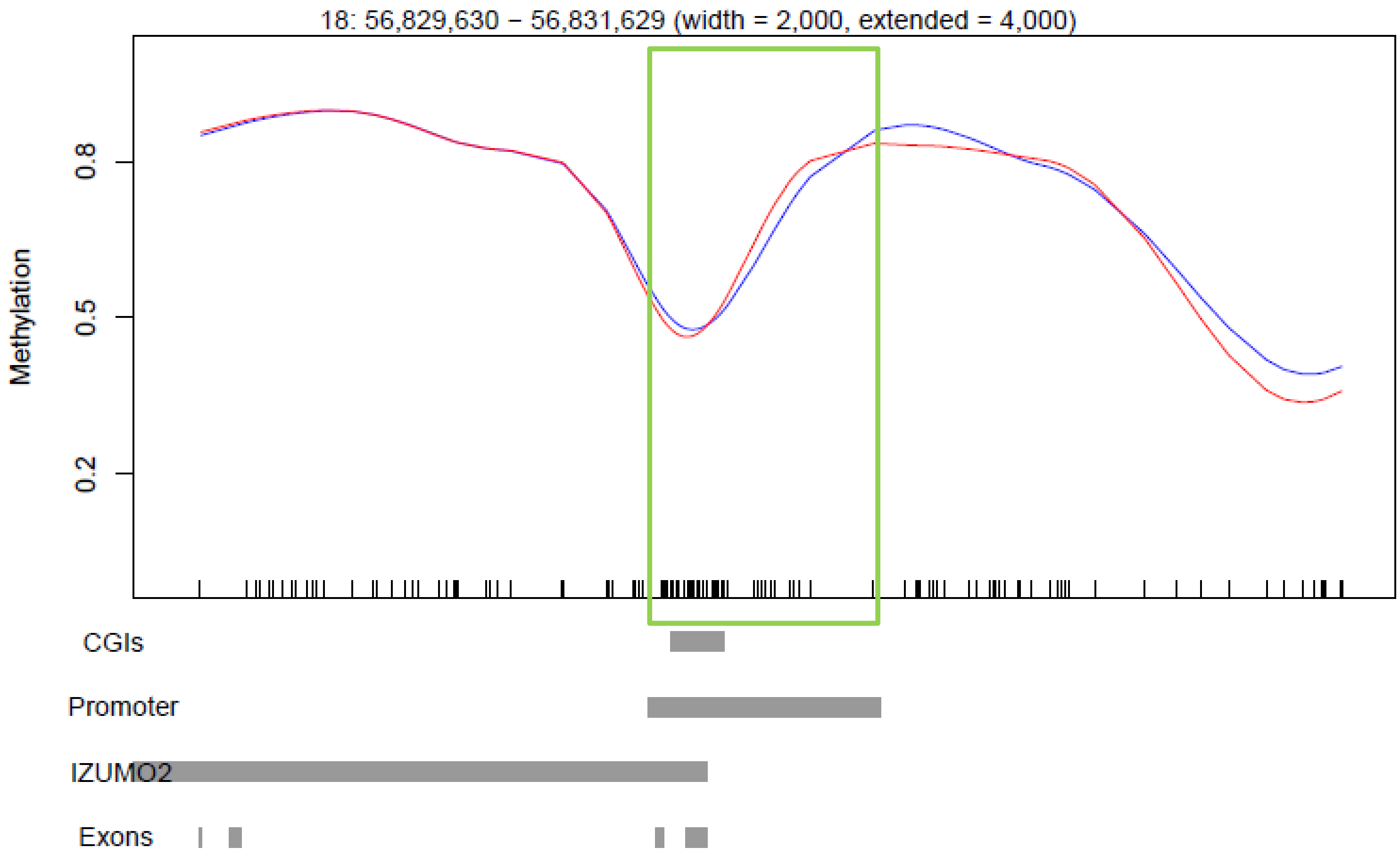
18: 56,829,630 − 56,831,629 (width = 2,000, extended = 4,000)
Methylation
0.8
0.5
0.2
CGIs
Promoter
IZUMO2
Exons

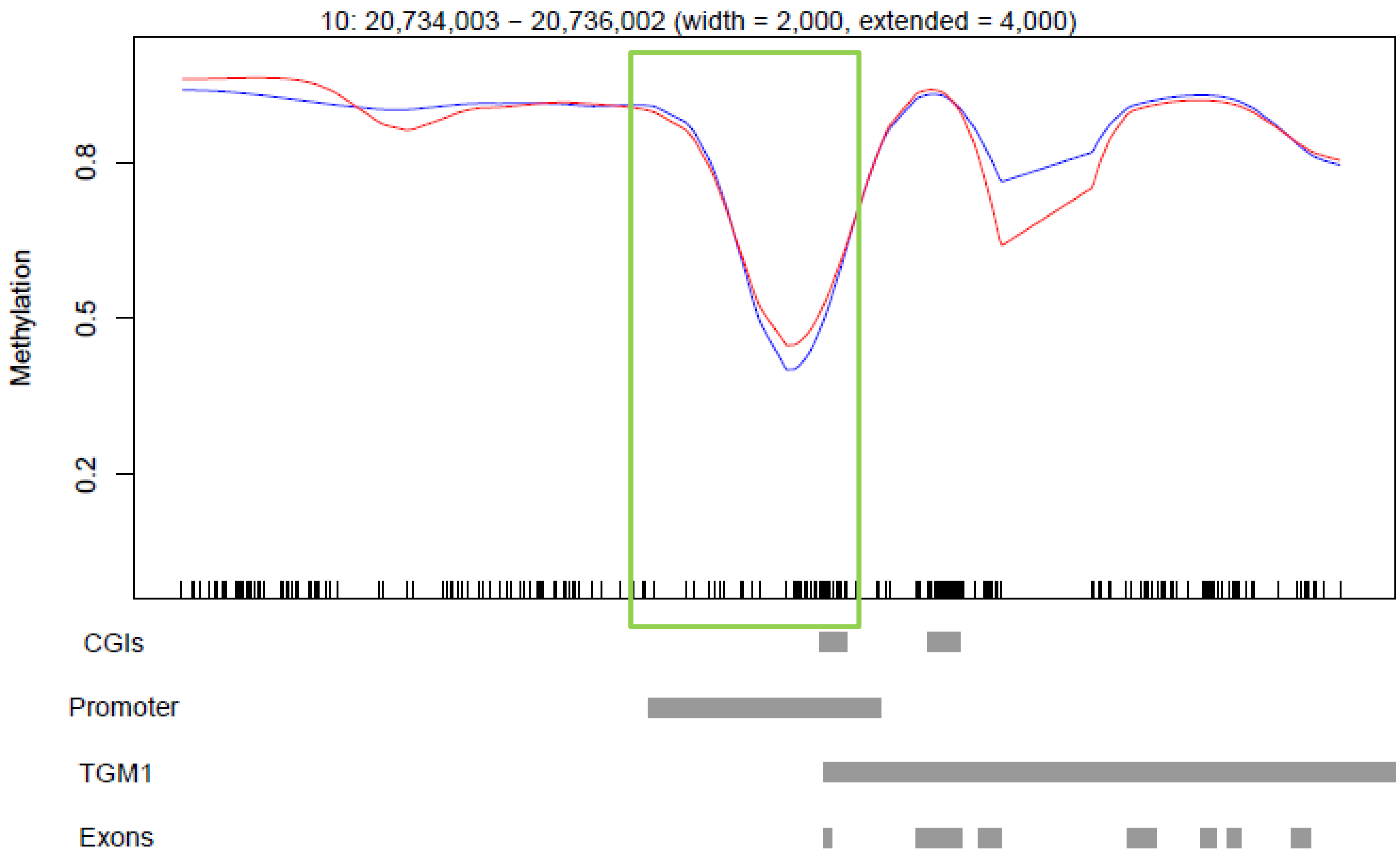
10: 20,734,003 − 20,736,002 (width = 2,000, extended = 4,000)
Methylation
0.8
0.5
0.2
CGIs
Promoter
TGM1
Exons

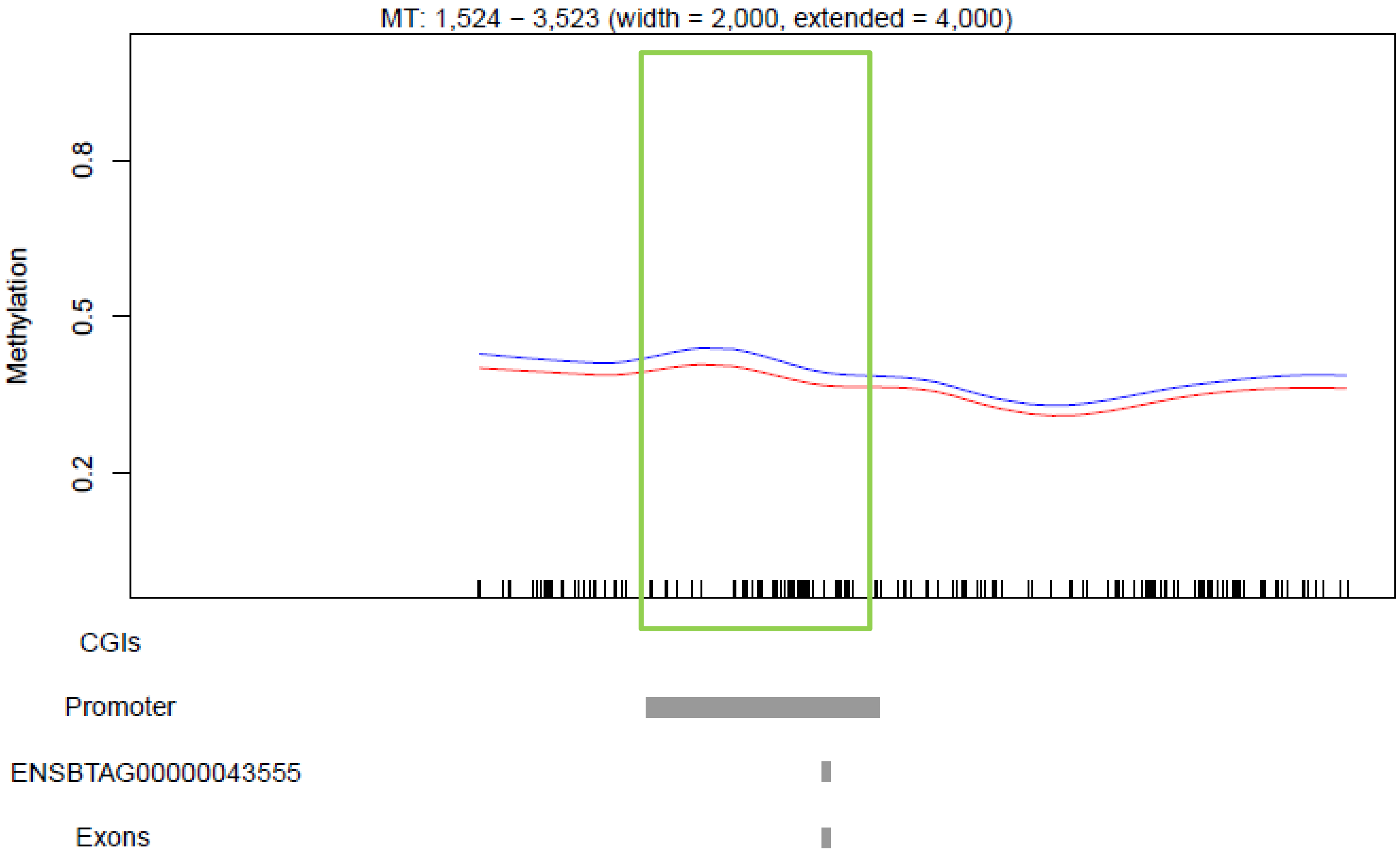
MT: 1,524 − 3,523 (width = 2,000, extended = 4,000)
Methylation
0.8
0.5
0.2
CGIs
Promoter
ENSBTAG00000043555
Exons

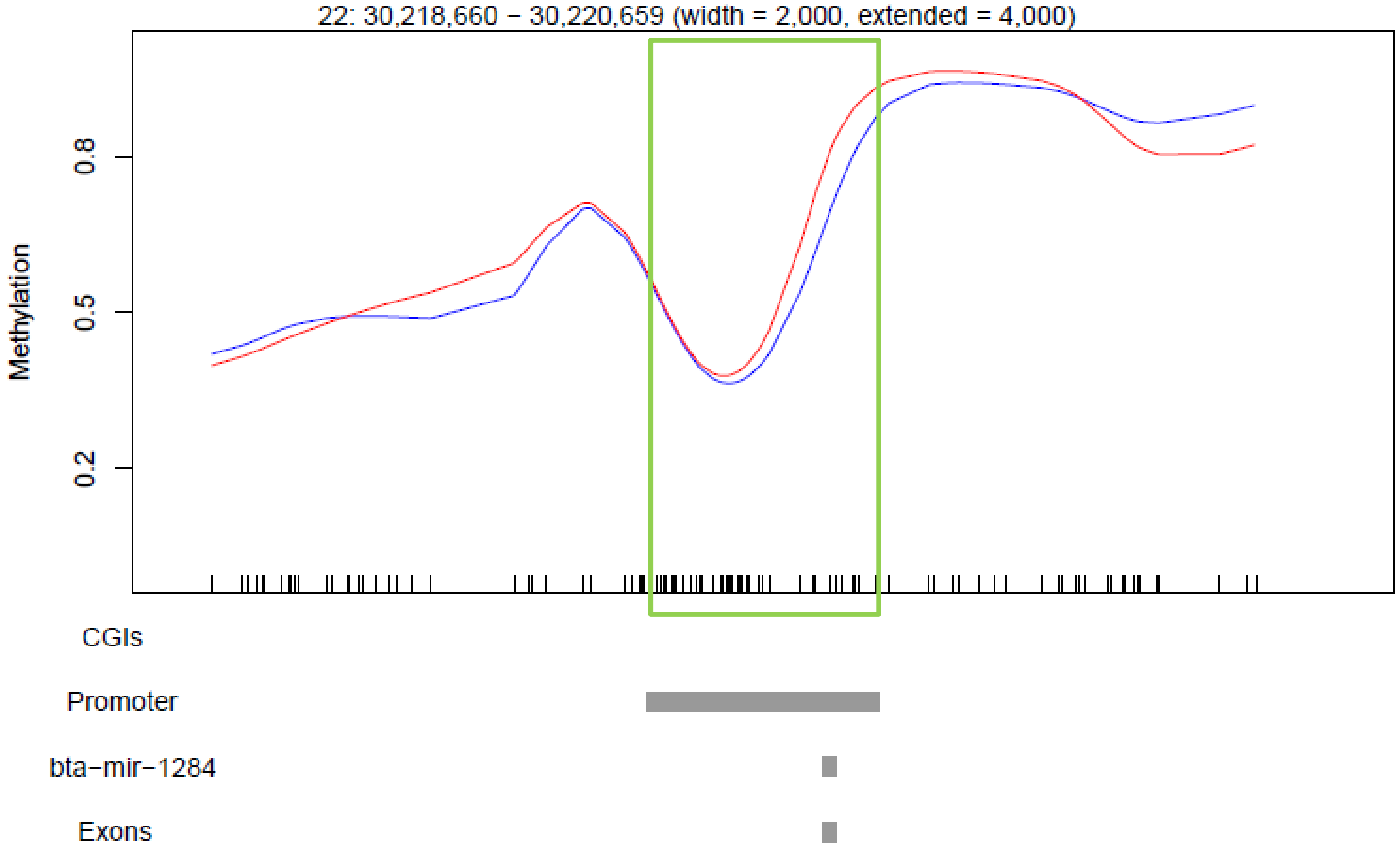
22: 30,218,660 − 30,220,659 (width = 2,000, extended = 4,000)
Methylation
0.8
0.5
0.2
CGIs
Promoter
bta−mir−1284
Exons

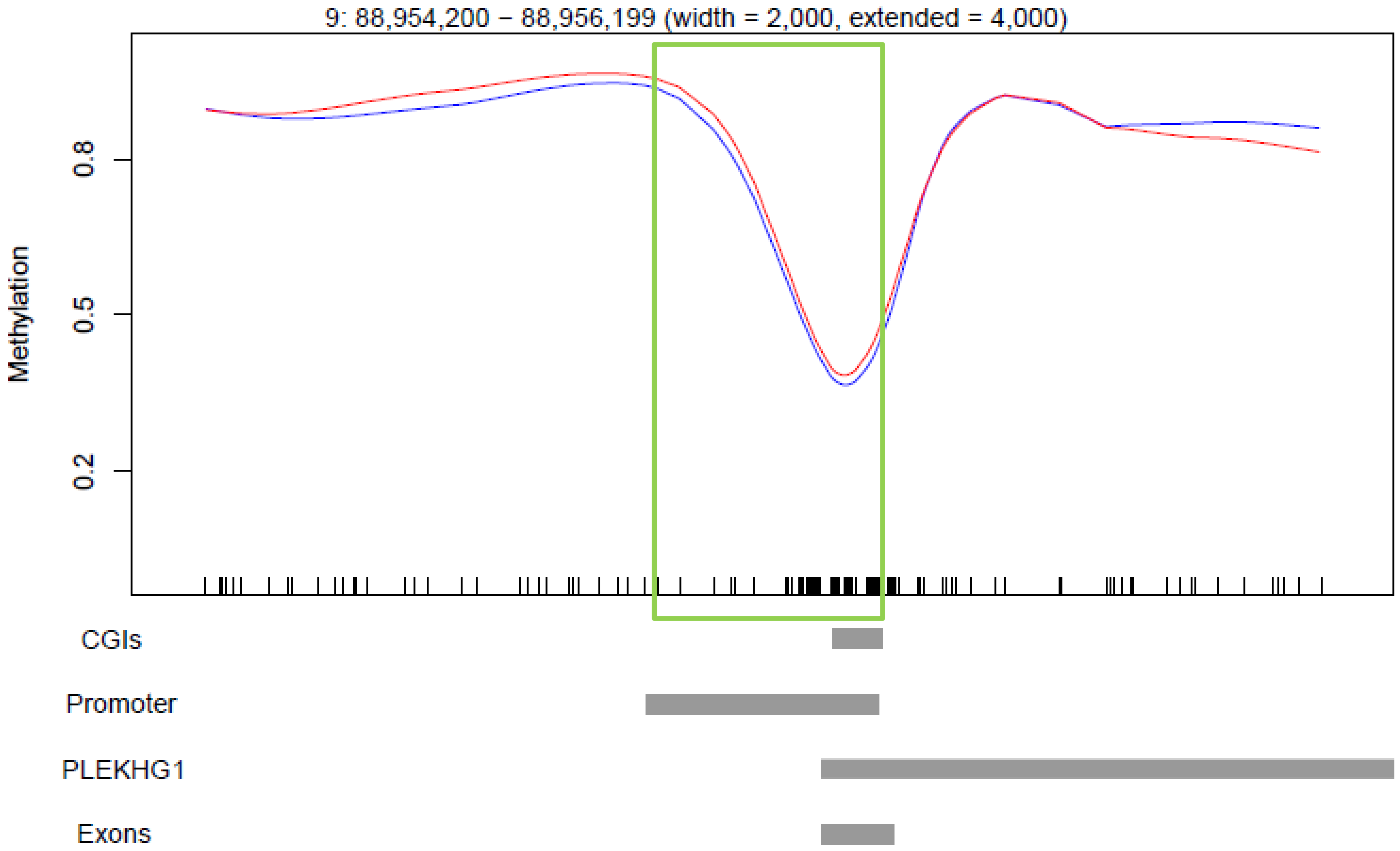
9: 88,954,200 – 88,956,199 (width = 2,000, extended = 4,000)
Methylation
0.8
0.5
0.2
CGIs
Promoter
PLEKHG1
Exons

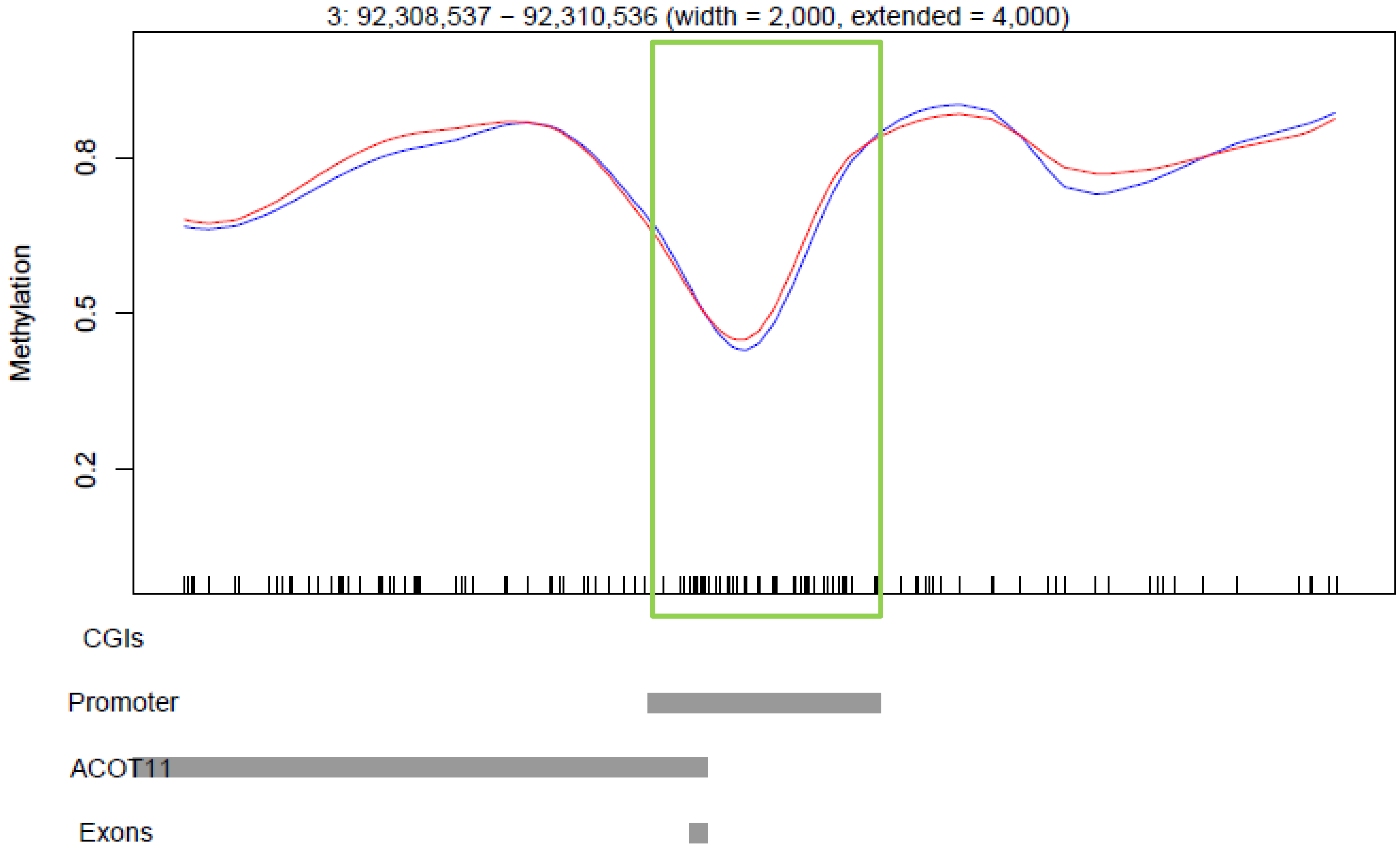
3: 92,308,537 − 92,310,536 (width = 2,000, extended = 4,000)
Methylation
0.8
0.5
0.2
CGIs
Promoter
ACOT11
Exons

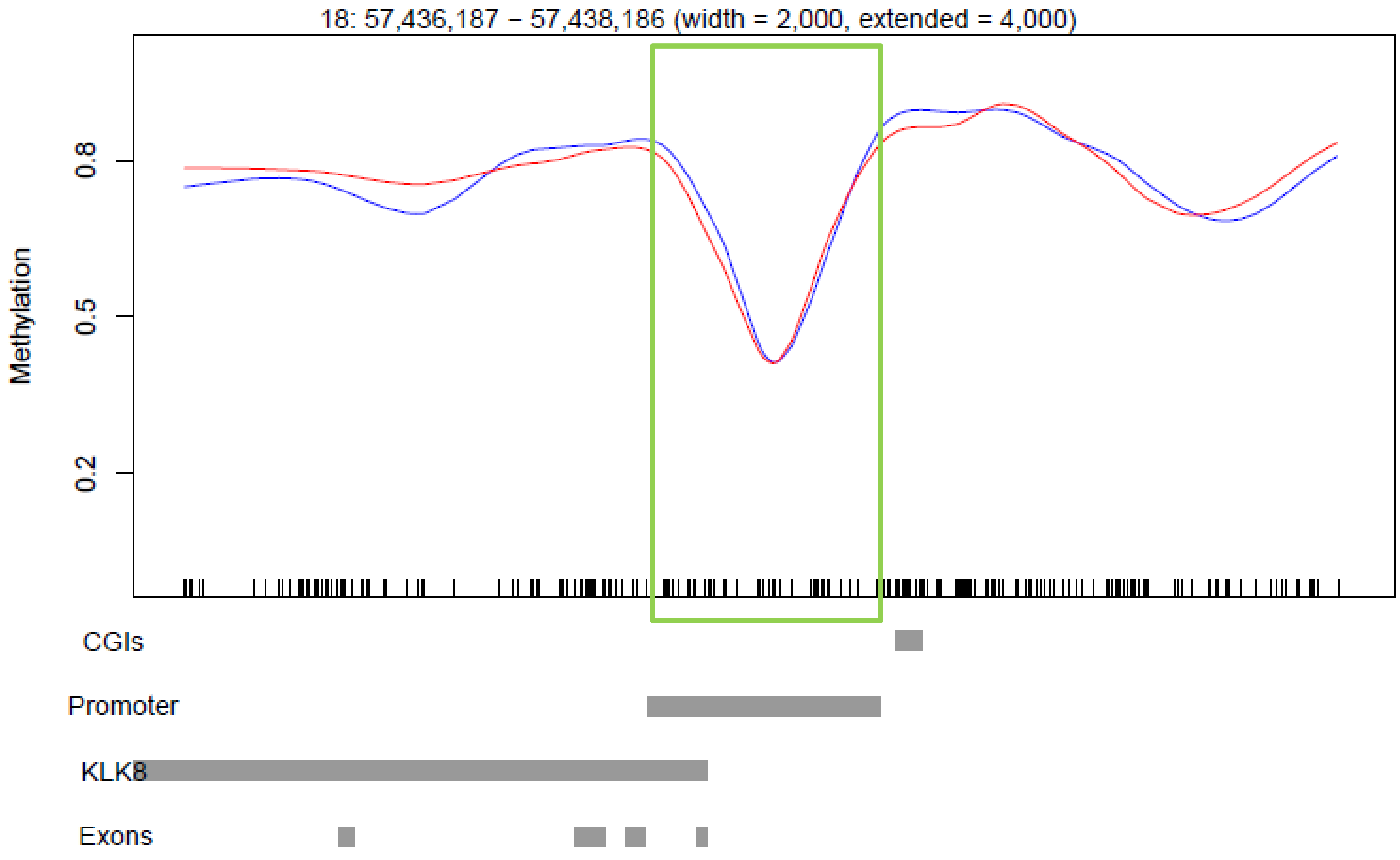

18: 57,436,187 − 57,438,186 (width = 2,000, extended = 4,000)
Methylation
0.2
0.5
0.8
CGIs
Promoter
KLK8
Exons

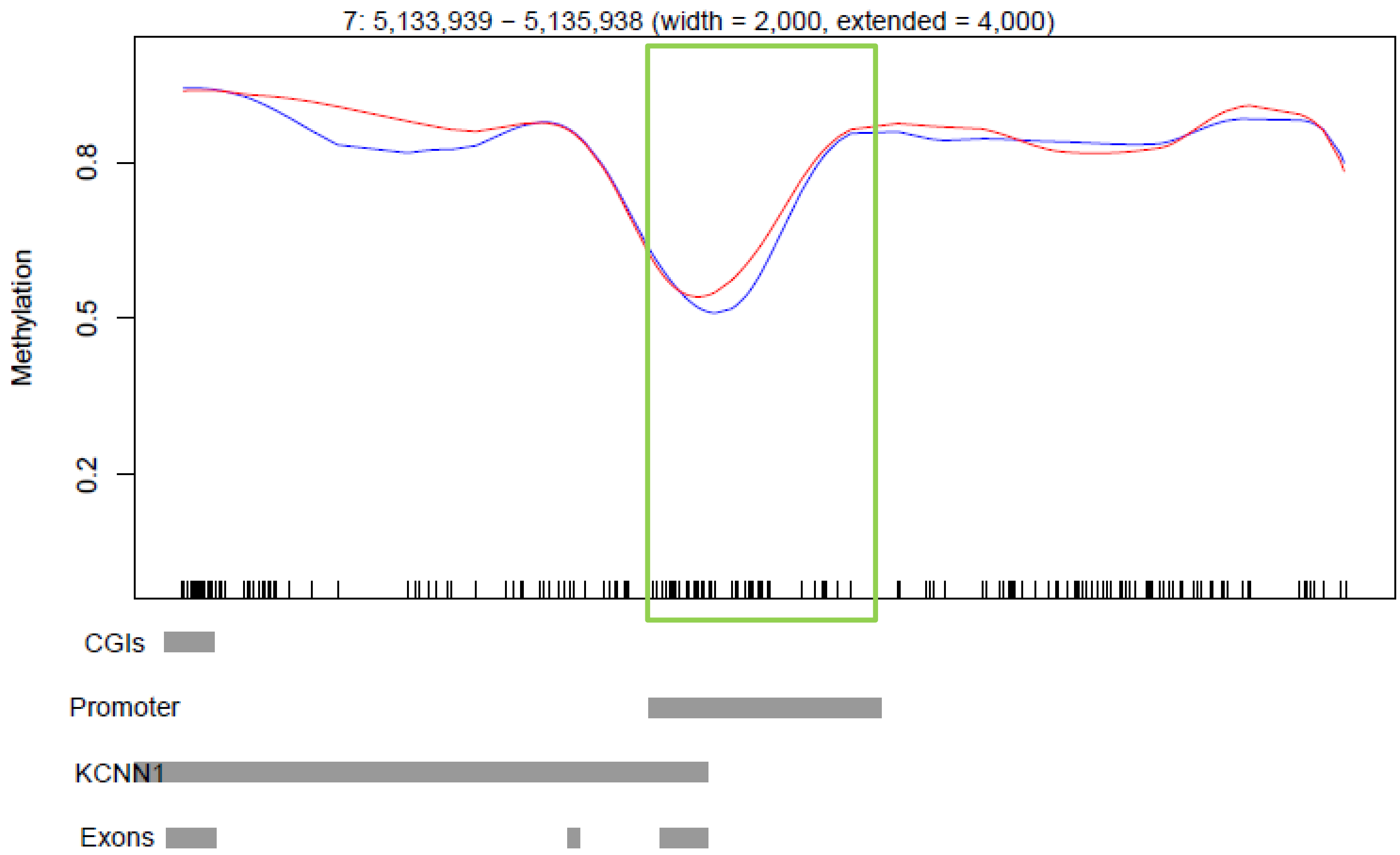
7: 5,133,939 − 5,135,938 (width = 2,000, extended = 4,000)
Methylation
0.8
0.5
0.2
CGIs
Promoter
KCNN1
Exons

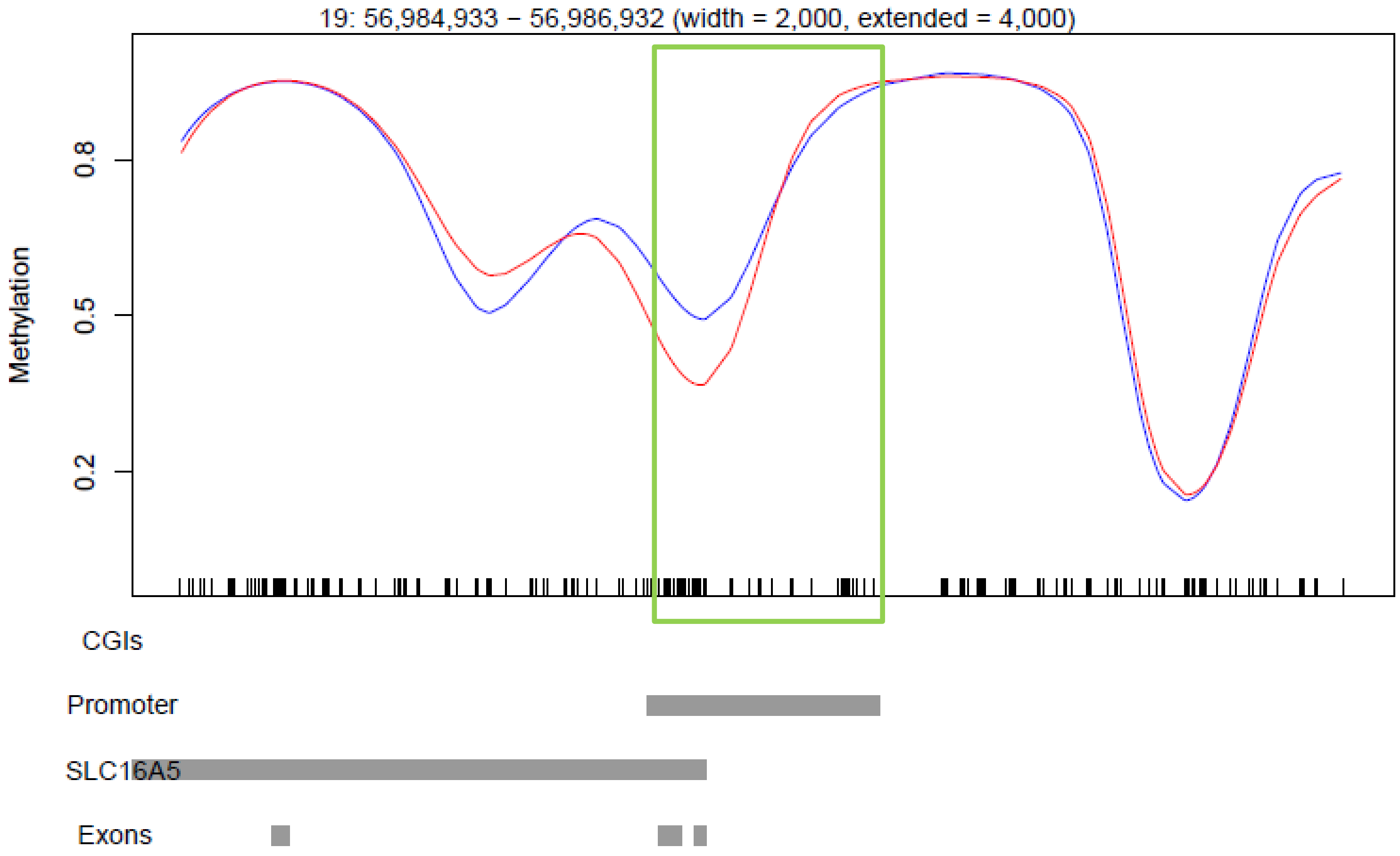

19: 56,984,933 − 56,986,932 (width = 2,000, extended = 4,000)
Methylation
0.8
0.5
0.2
CGIs
Promoter
SLC16A5
Exons

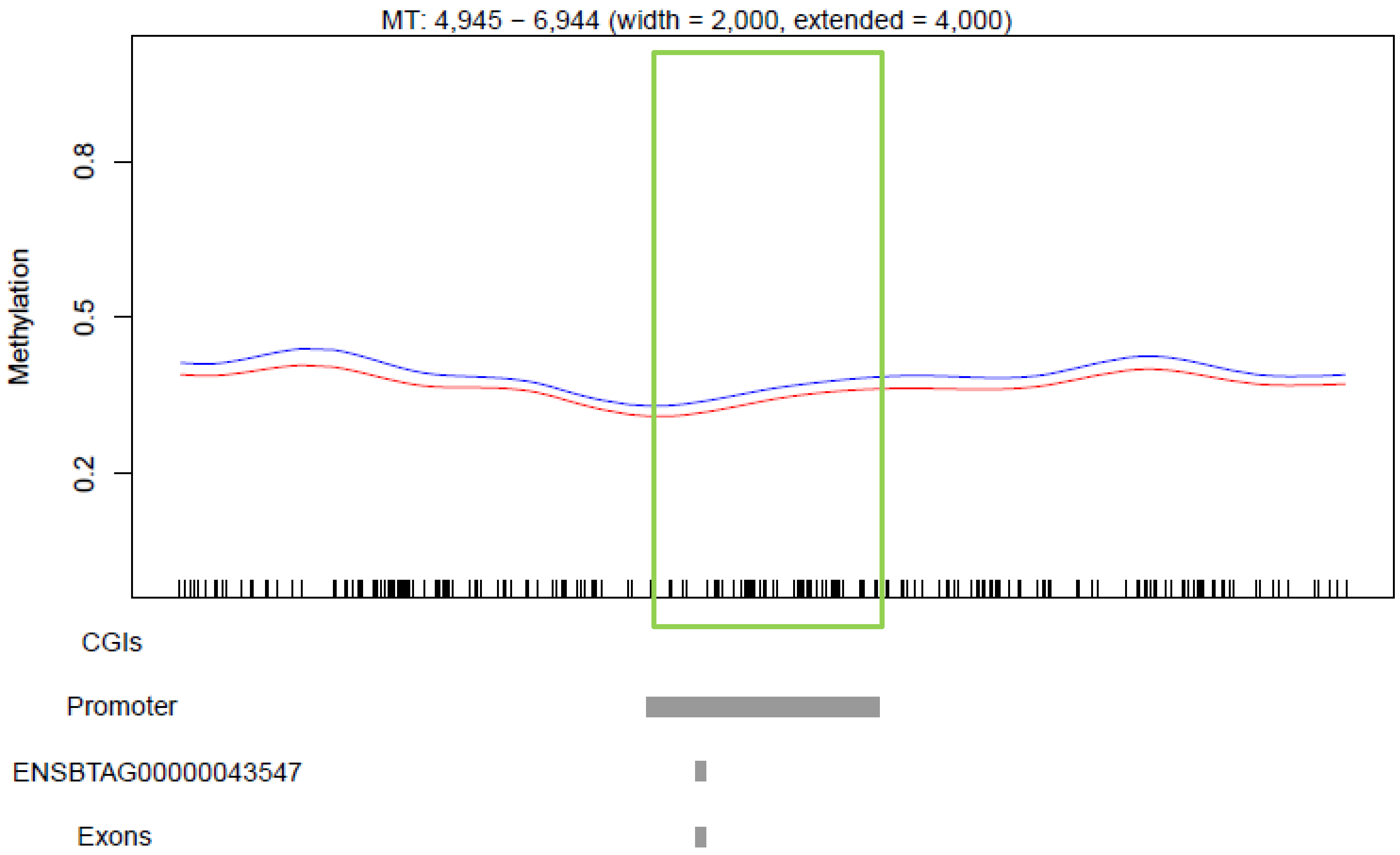
MT: 4,945 − 6,944 (width = 2,000, extended = 4,000)
Methylation
0.8
0.5
0.2
CGIs
Promoter
ENSBTAG00000043547
Exons

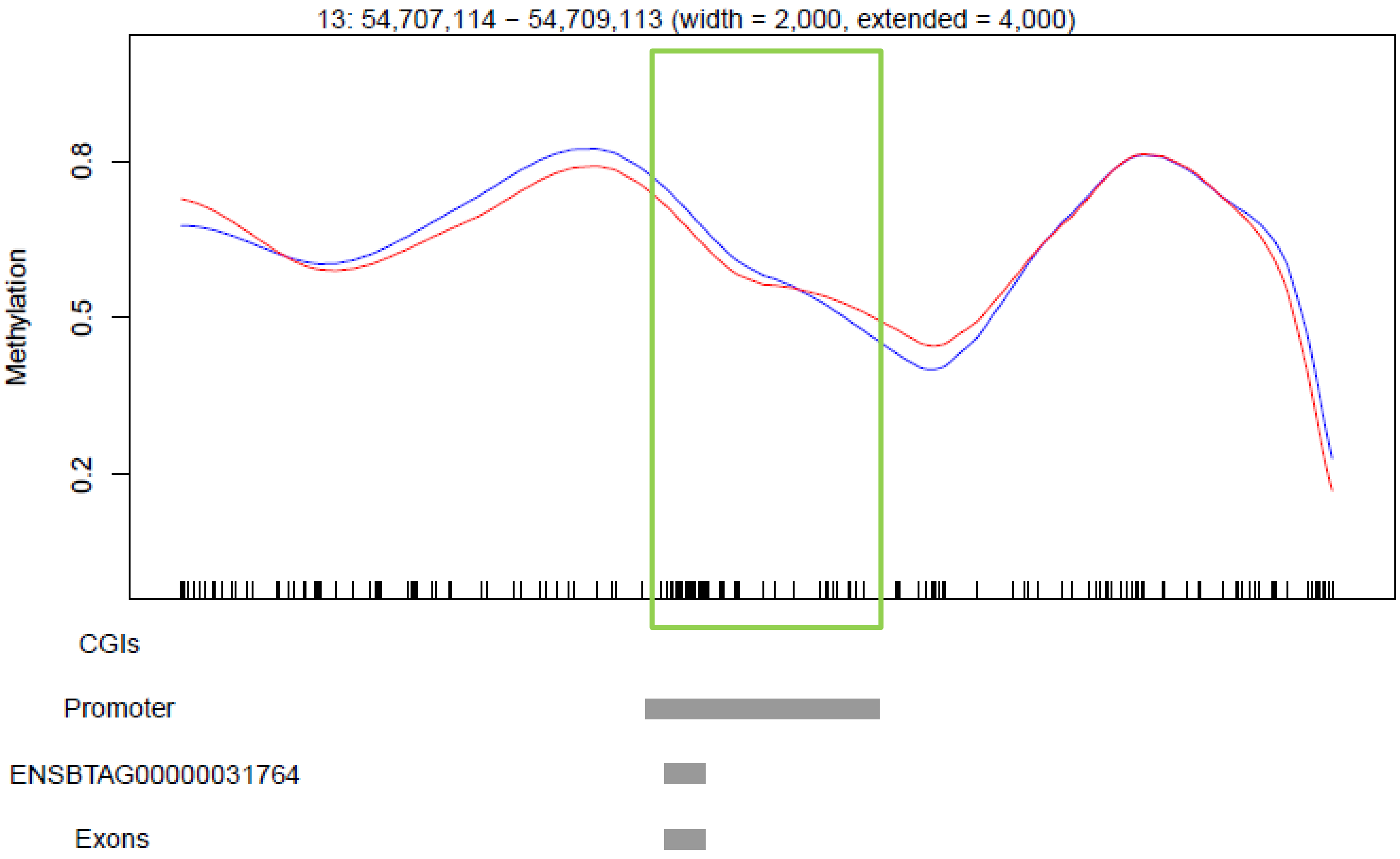
13: 54,707,114 − 54,709,113 (width = 2,000, extended = 4,000)
Methylation
0.2
0.5
0.8
CGIs
Promoter
ENSBTAG00000031764
Exons

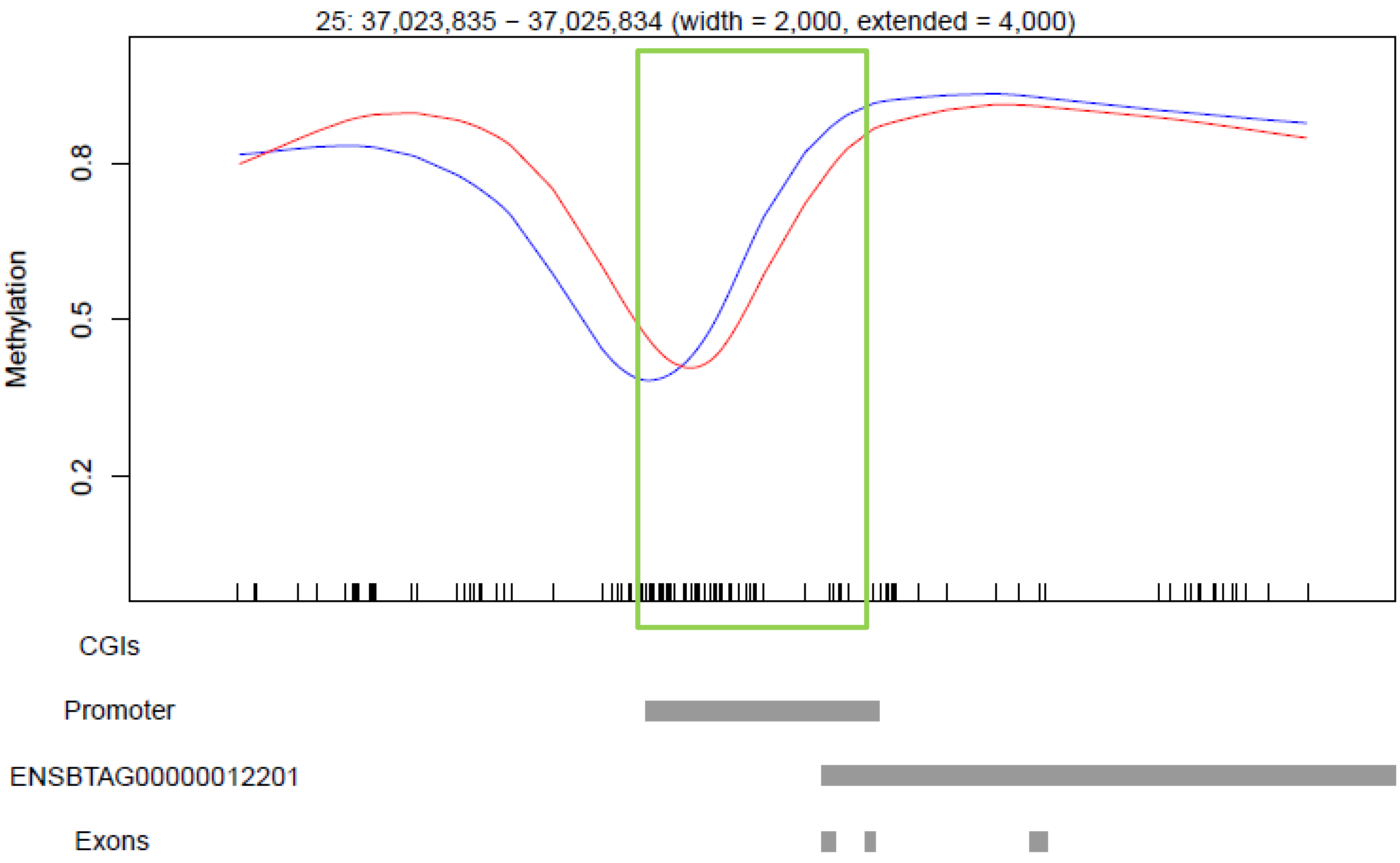

25: 37,023,835 – 37,025,834 (width = 2,000, extended = 4,000)
Methylation
0.8
0.5
0.2
CGIs
Promoter
ENSBTAG00000012201
Exons

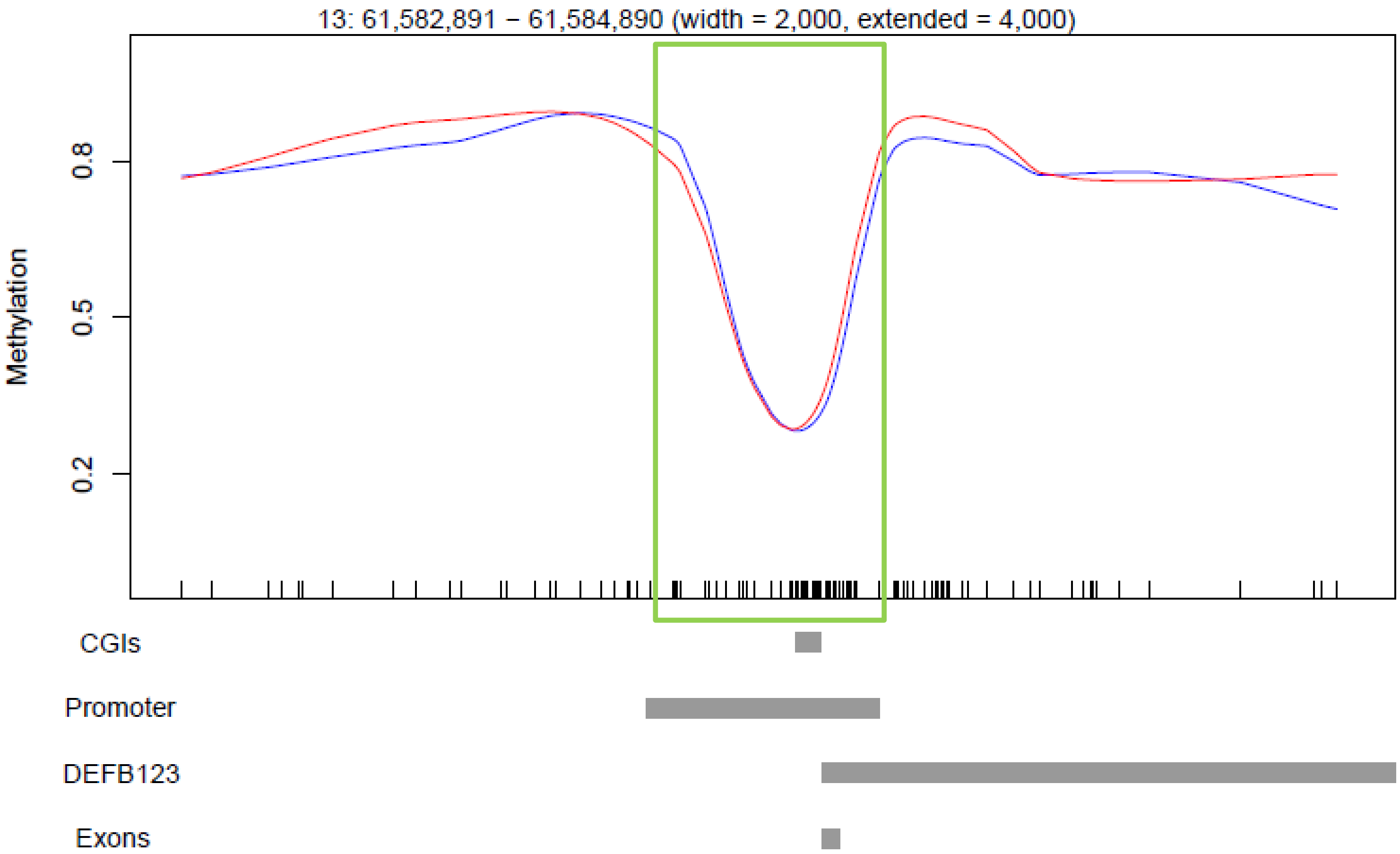
13: 61,582,891 − 61,584,890 (width = 2,000, extended = 4,000)
Methylation
0.8
0.5
0.2
CGIs
Promoter
DEFB123
Exons

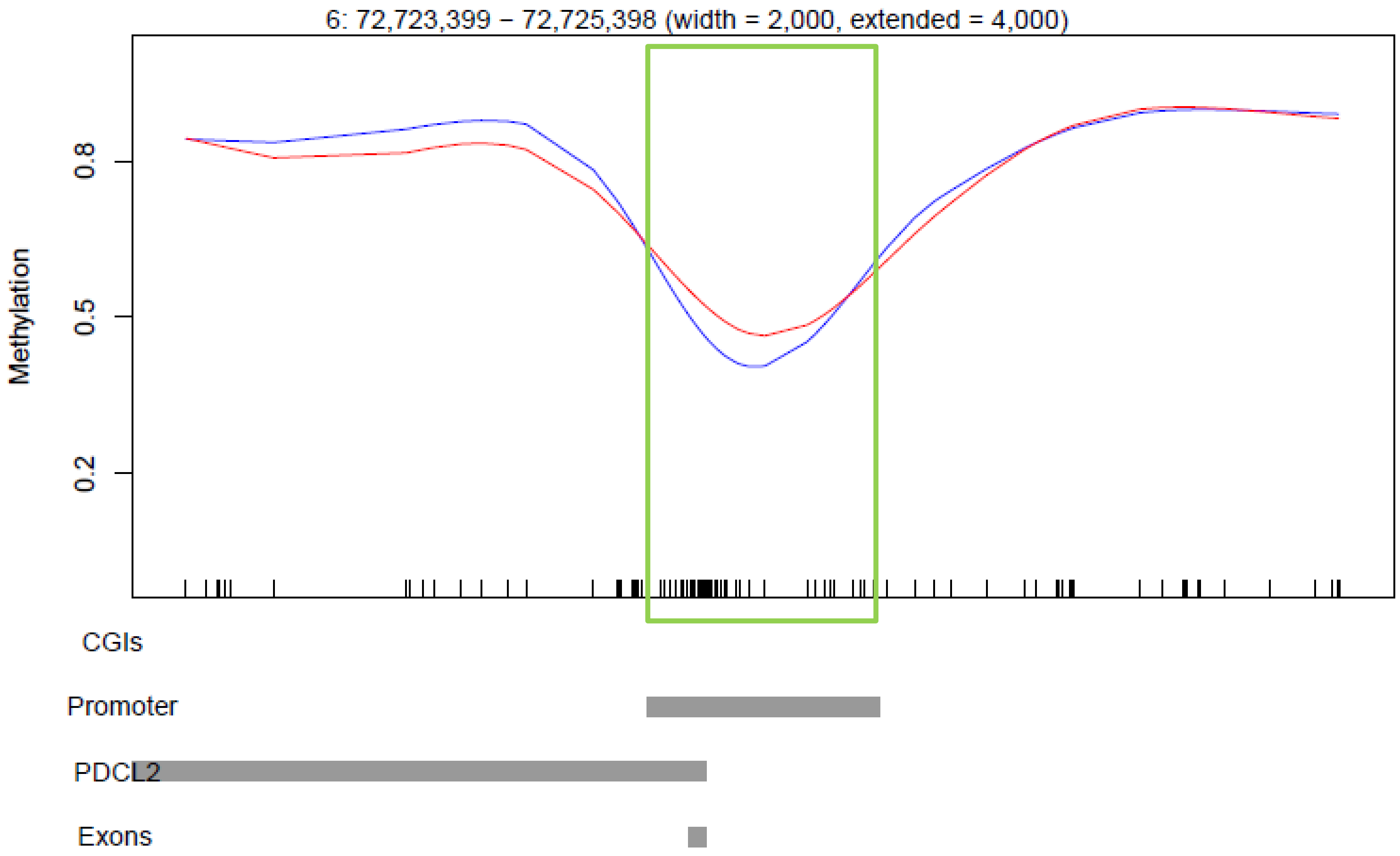
6: 72,723,399 − 72,725,398 (width = 2,000, extended = 4,000)
Methylation
0.8
0.5
0.2
CGIs
Promoter
PDCL2
Exons

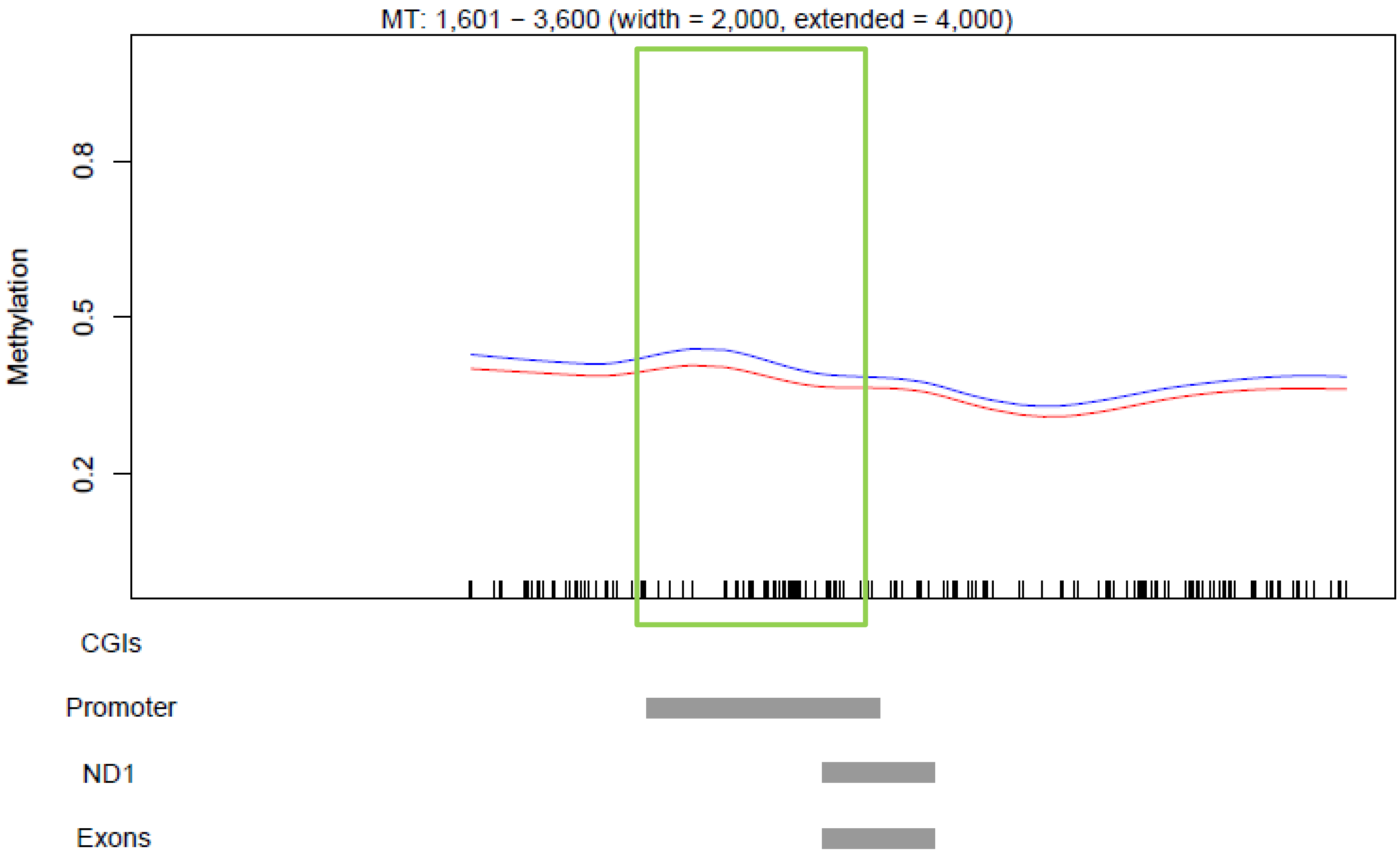

MT: 1,601 − 3,600 (width = 2,000, extended = 4,000)
Methylation
0.2
0.5
0.8
CGIs
Promoter
ND1
Exons

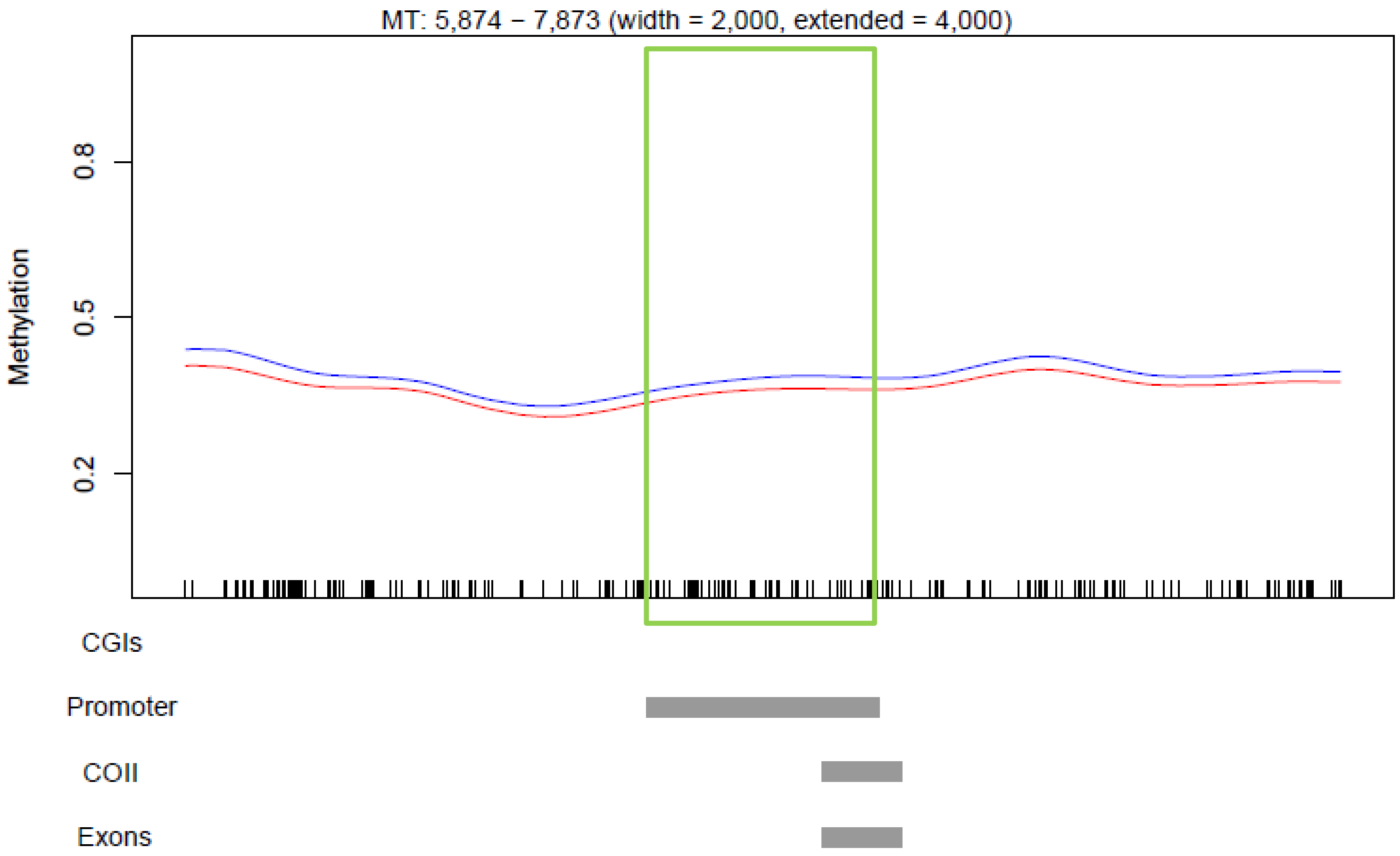
MT: 5,874 − 7,873 (width = 2,000, extended = 4,000)
Methylation
0.2
0.5
0.8
CGIs
Promoter
COII
Exons

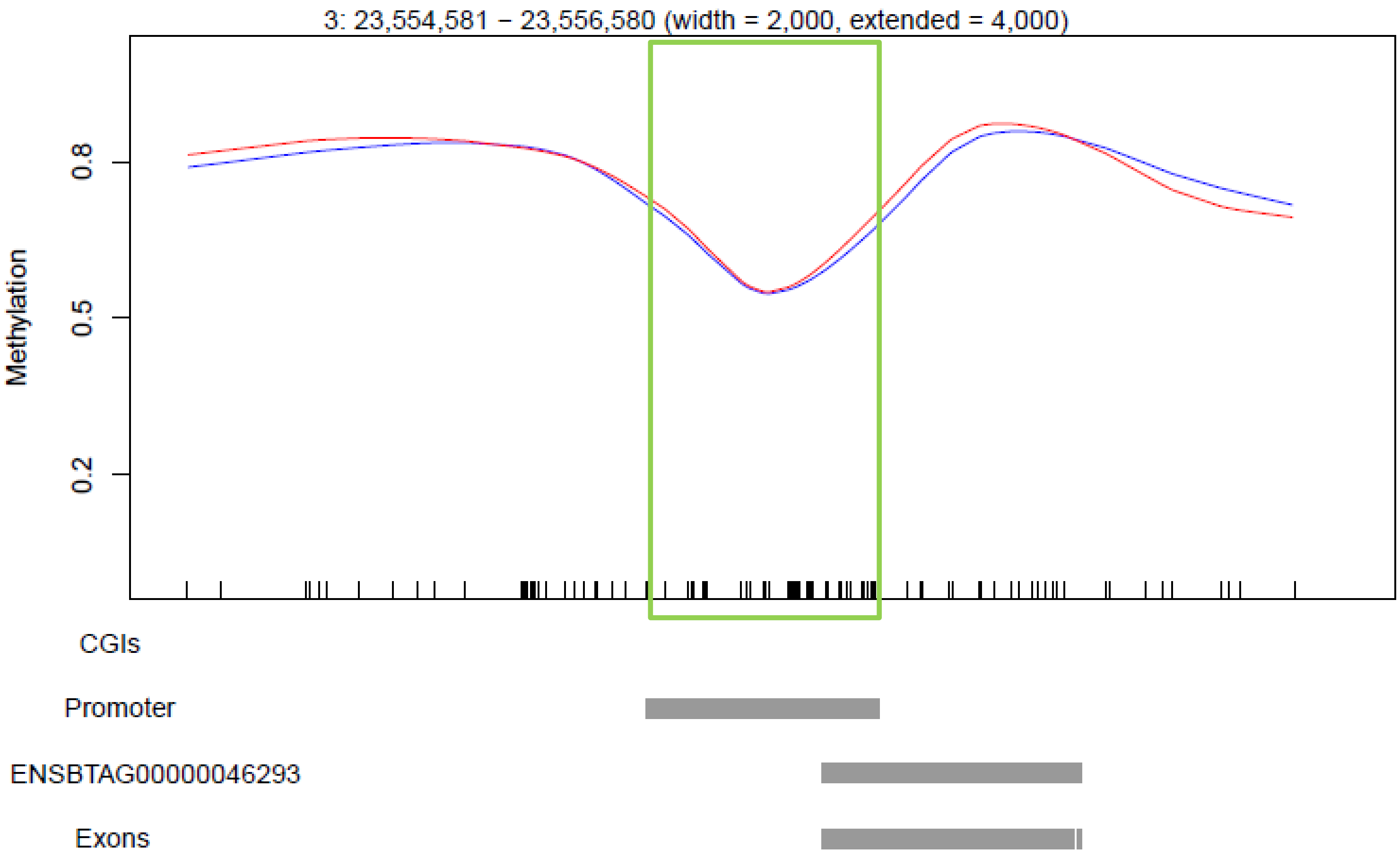

3: 23,554,581 − 23,556,580 (width = 2,000, extended = 4,000)
Methylation
0.8
0.5
0.2
CGIs
Promoter
ENSBTAG00000046293
Exons

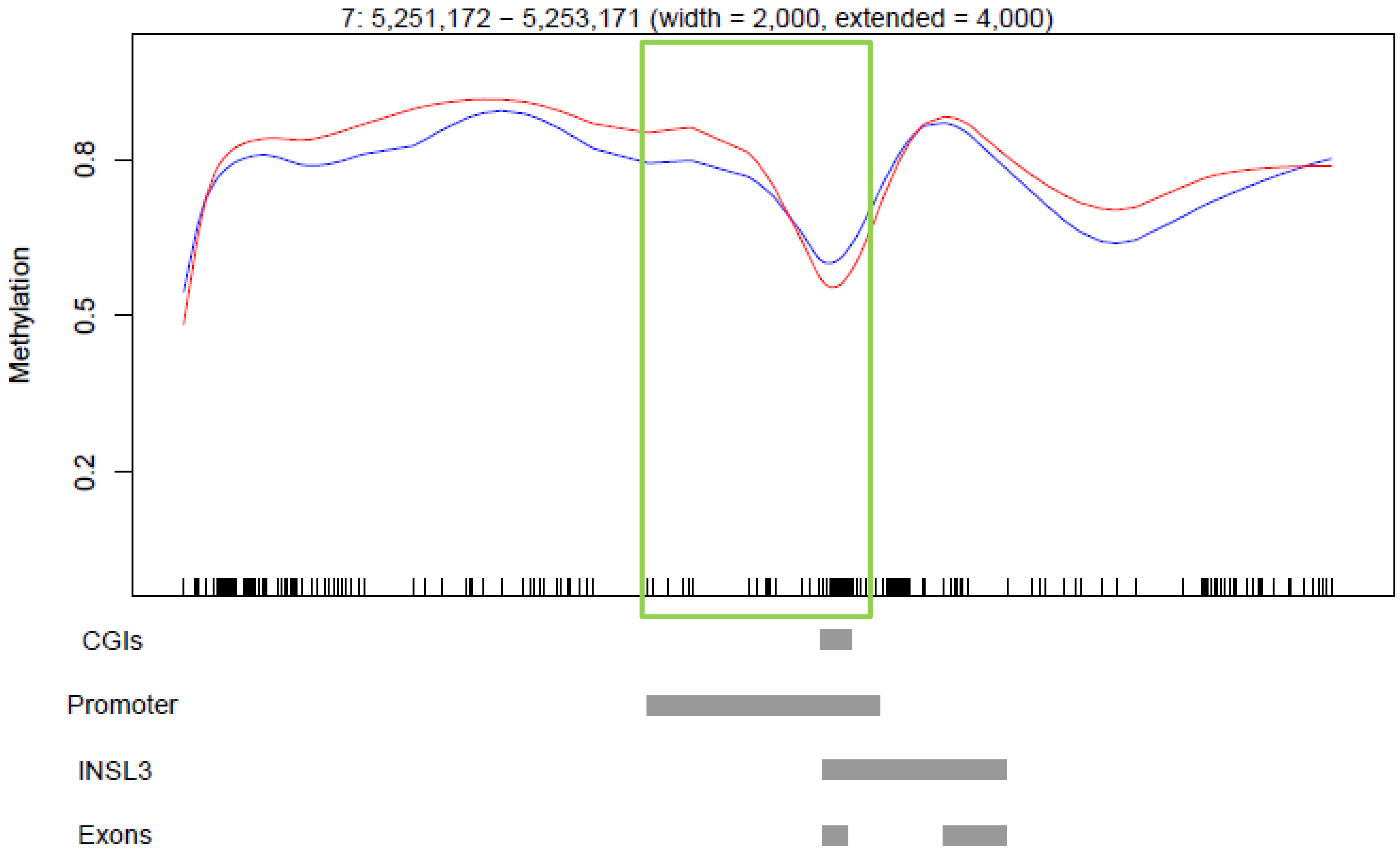
7: 5,251,172 − 5,253,171 (width = 2,000, extended = 4,000)
Methylation
0.2
0.5
0.8
CGIs
Promoter
INSL3
Exons

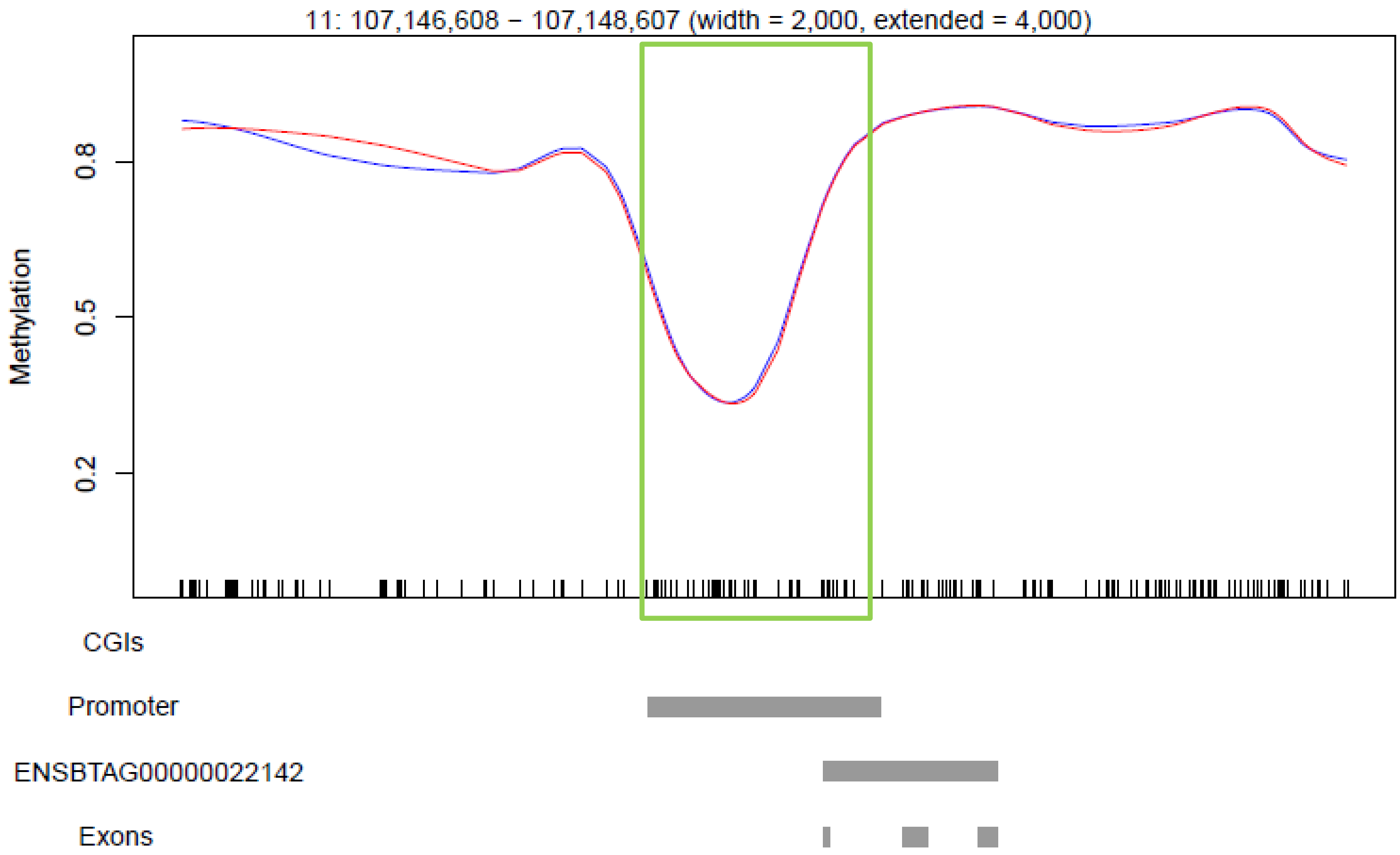
11: 107,146,608 – 107,148,607 (width = 2,000, extended = 4,000)
Methylation
0.8
0.5
0.2
CGIs
Promoter
ENSBTAG00000022142
Exons

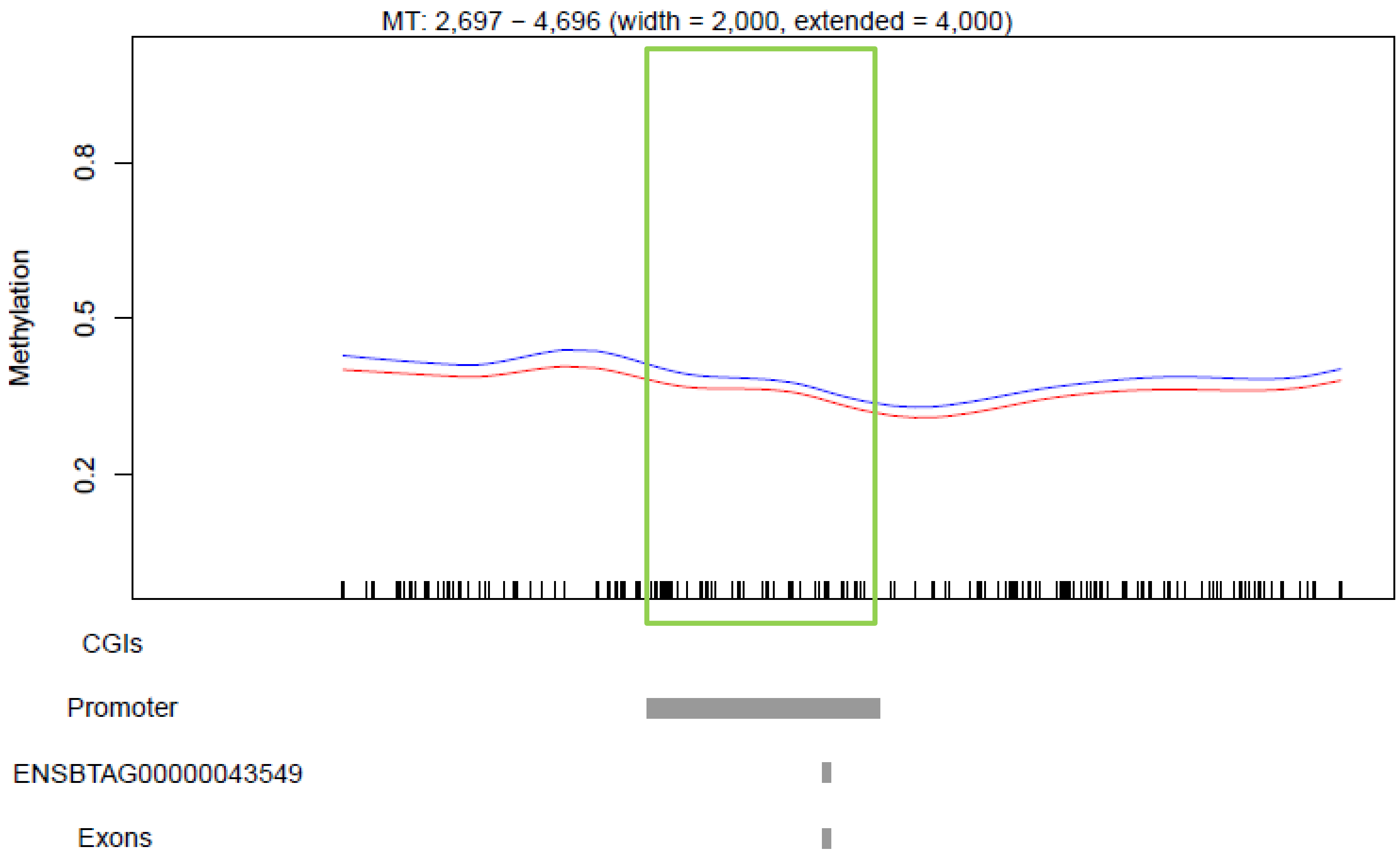

MT: 2,697 − 4,696 (width = 2,000, extended = 4,000)
Methylation
0.2
0.5
0.8
CGIs
Promoter
ENSBTAG00000043549
Exons

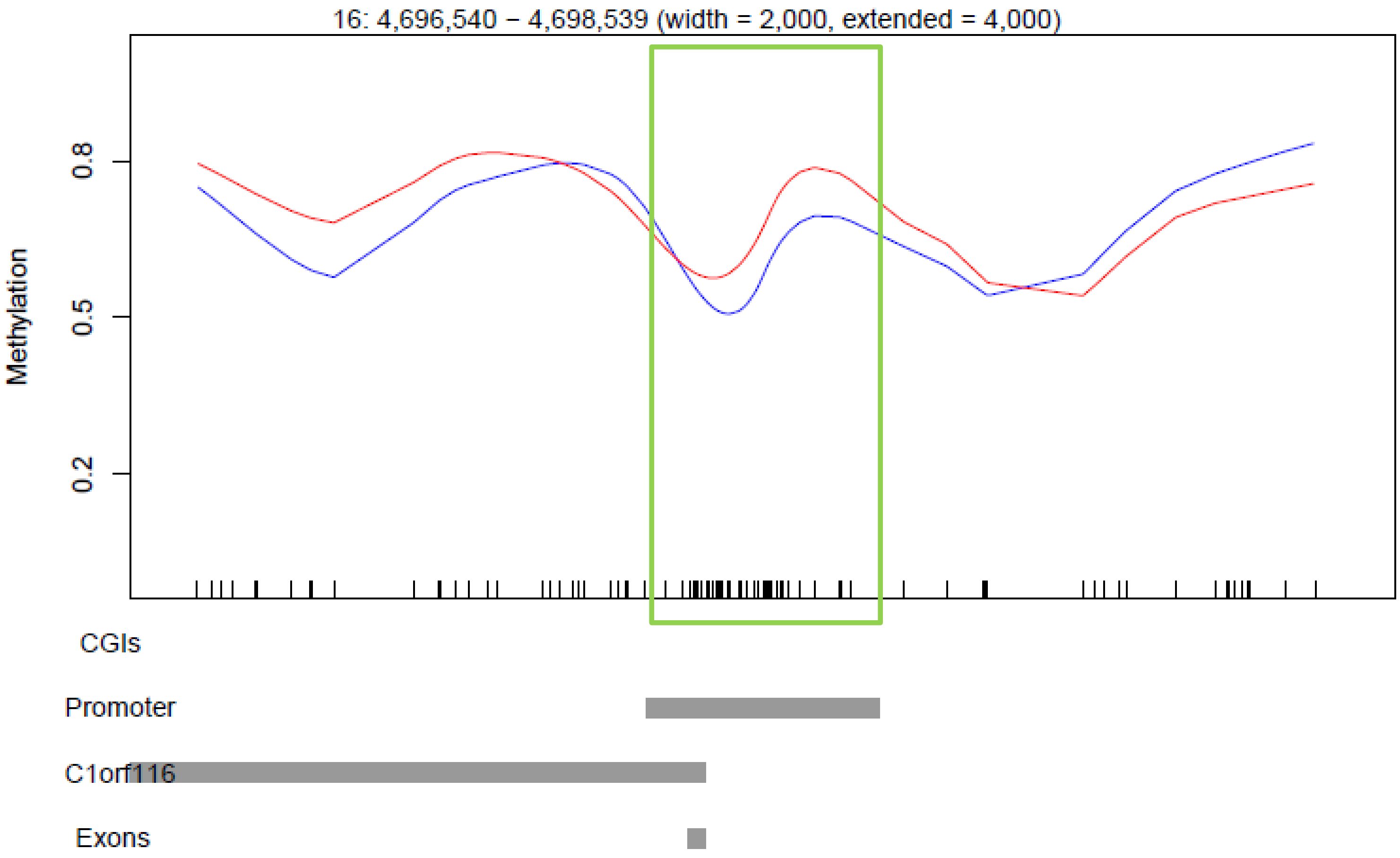
16: 4,696,540 − 4,698,539 (width = 2,000, extended = 4,000)
Methylation
0.8
0.5
0.2
CGIs
Promoter
C1orf116
Exons

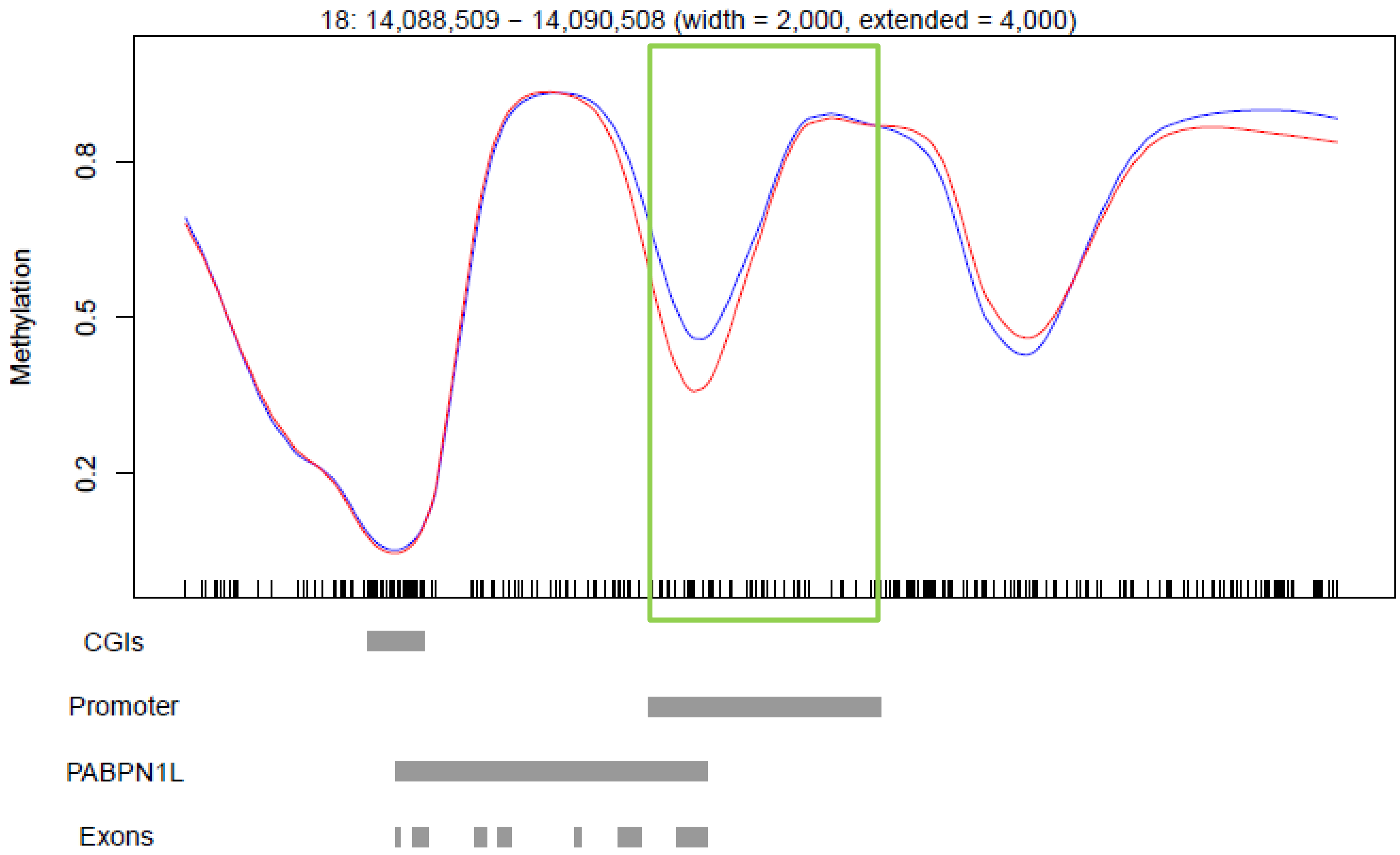

18: 14,088,509 − 14,090,508 (width = 2,000, extended = 4,000)
Methylation
0.2
0.5
0.8
CGIs
Promoter
PABPN1L
Exons

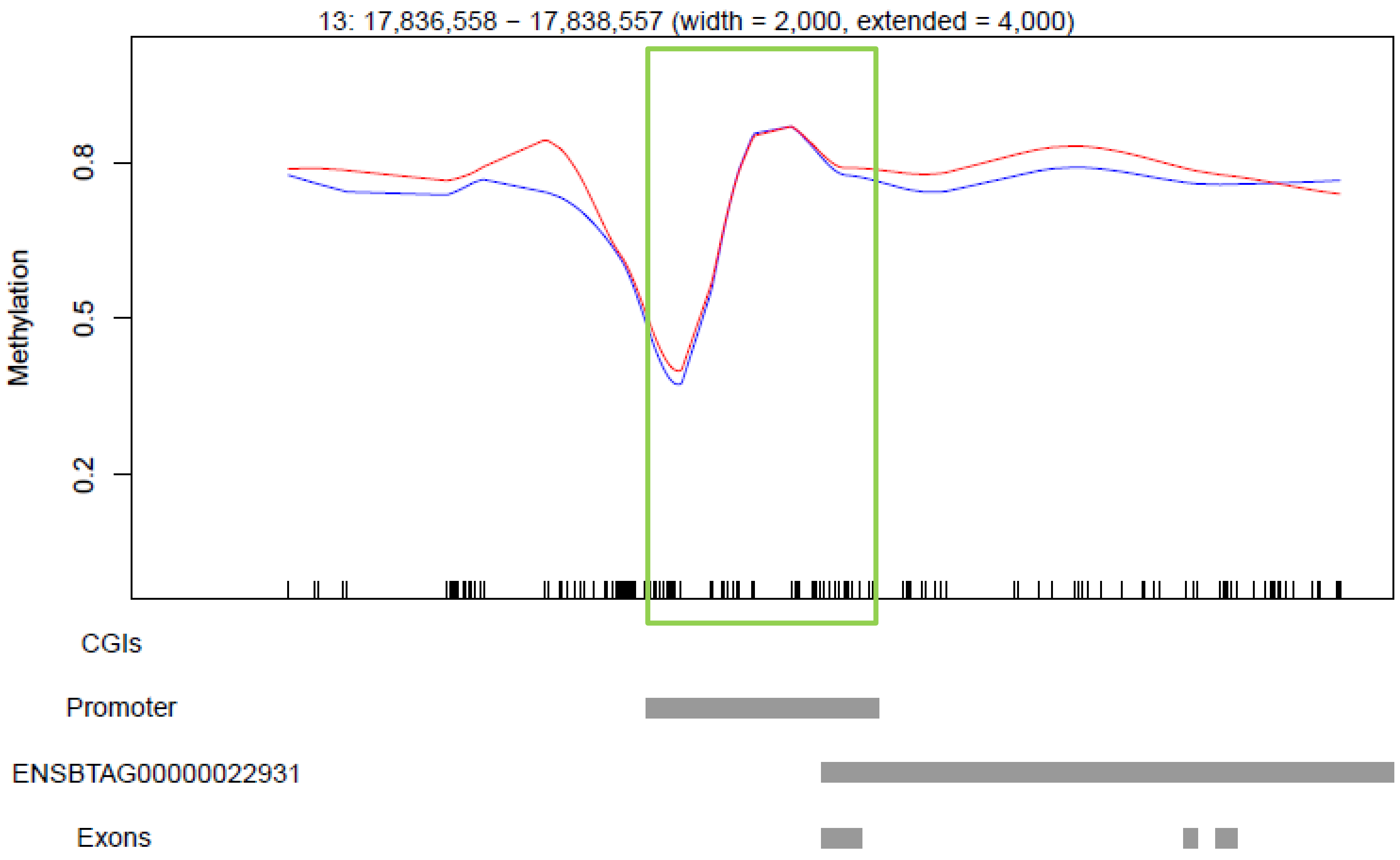

13: 17,836,558 – 17,838,557 (width = 2,000, extended = 4,000)
Methylation
0.8
0.5
0.2
CGIs
Promoter
ENSBTAG00000022931
Exons

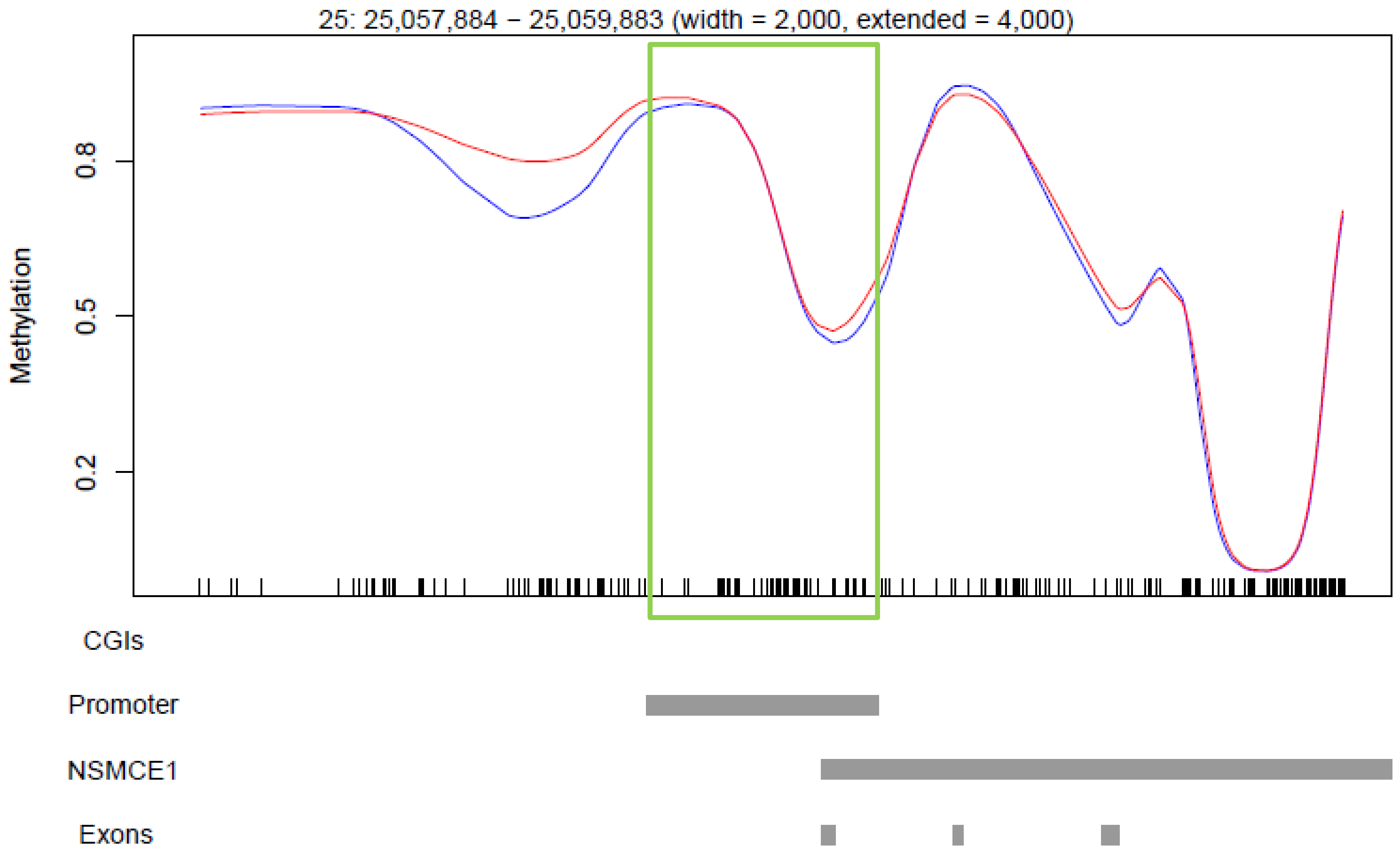

25: 25,057,884 – 25,059,883 (width = 2,000, extended = 4,000)
Methylation
0.8
0.5
0.2
CGIs
Promoter
NSMCE1
Exons

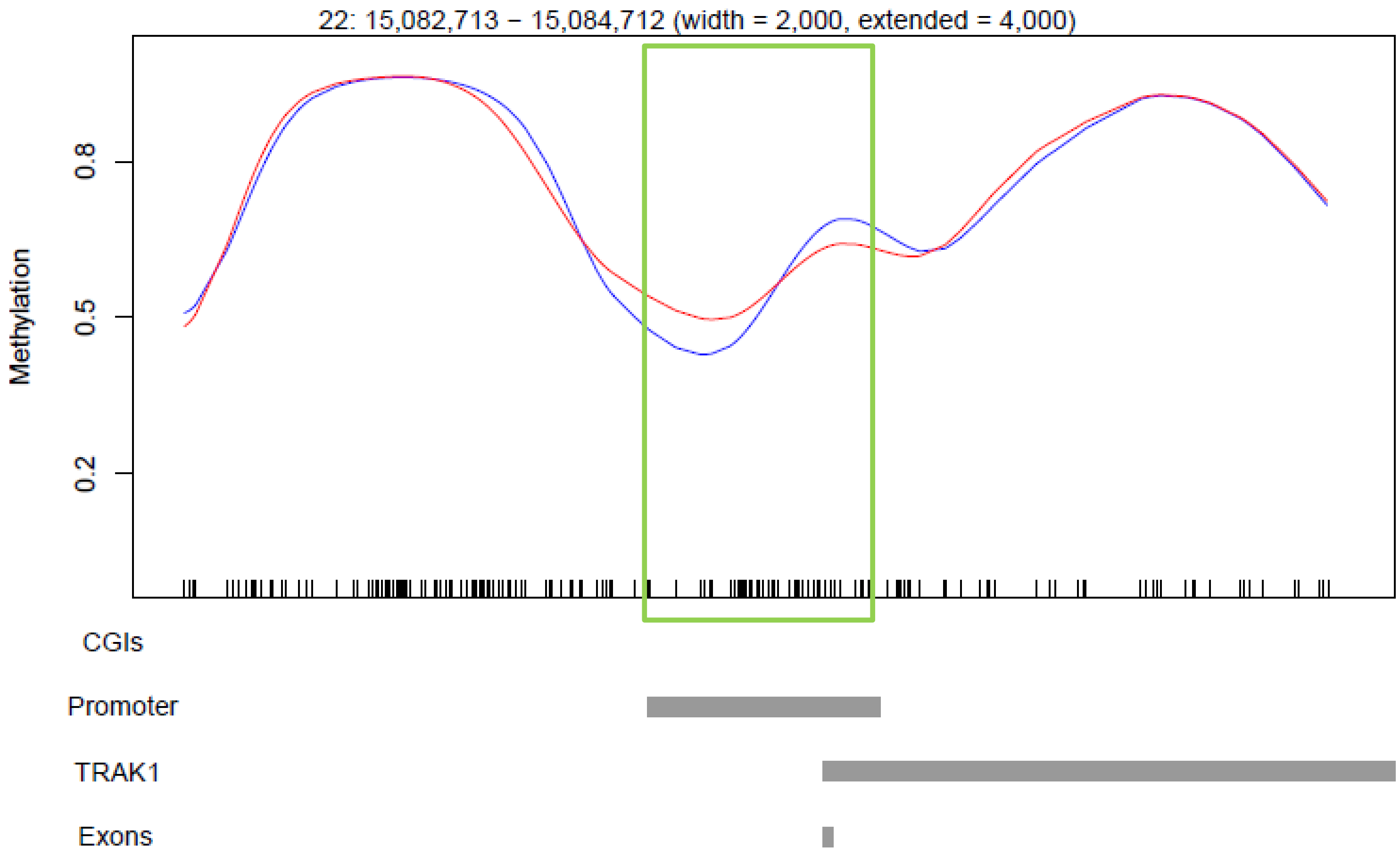

22: 15,082,713 − 15,084,712 (width = 2,000, extended = 4,000)
Methylation
0.8
0.5
0.2
CGIs
Promoter
TRAK1
Exons

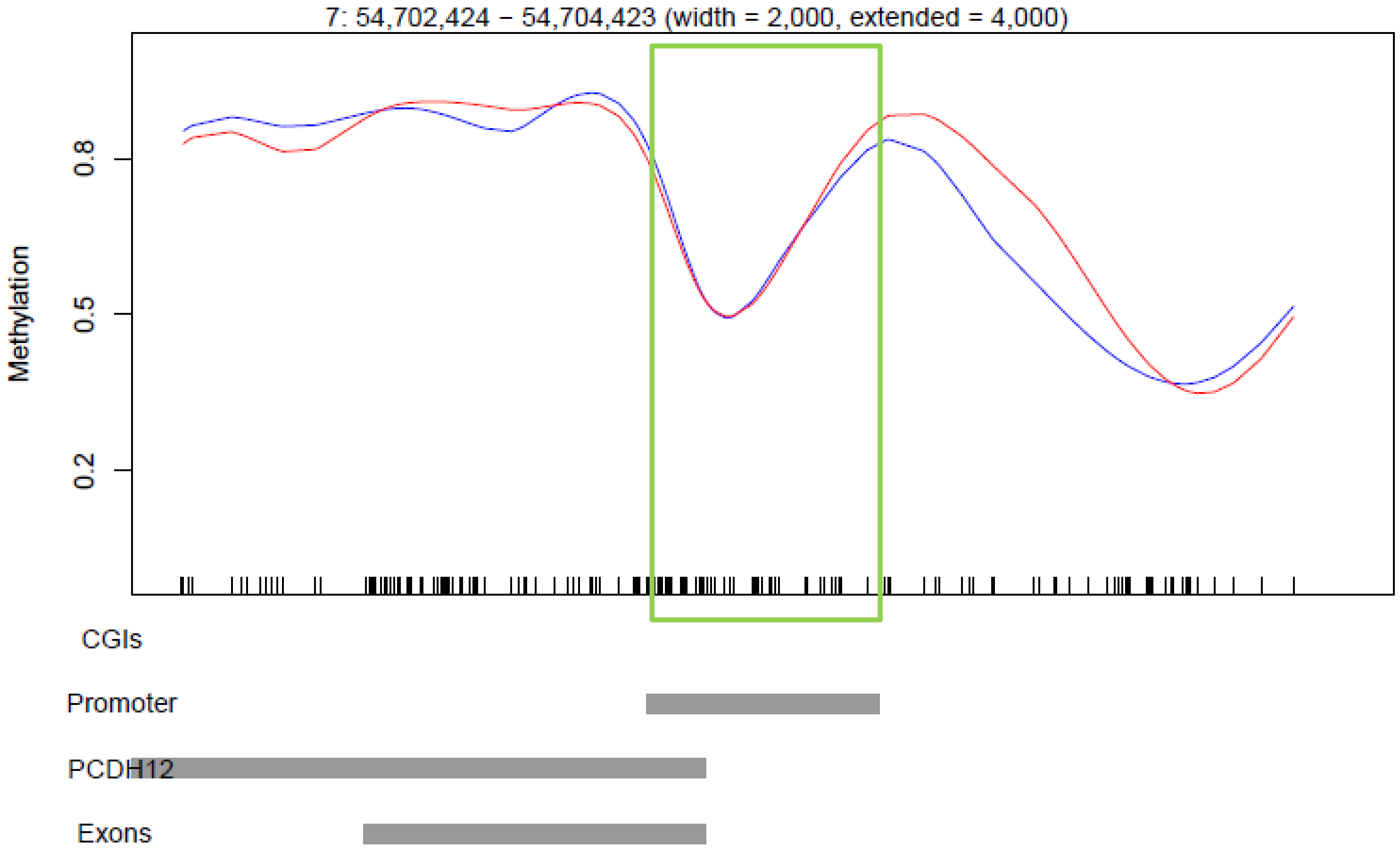
7: 54,702,424 − 54,704,423 (width = 2,000, extended = 4,000)
Methylation
0.8
0.5
0.2
CGIs
Promoter
PCDH12
Exons

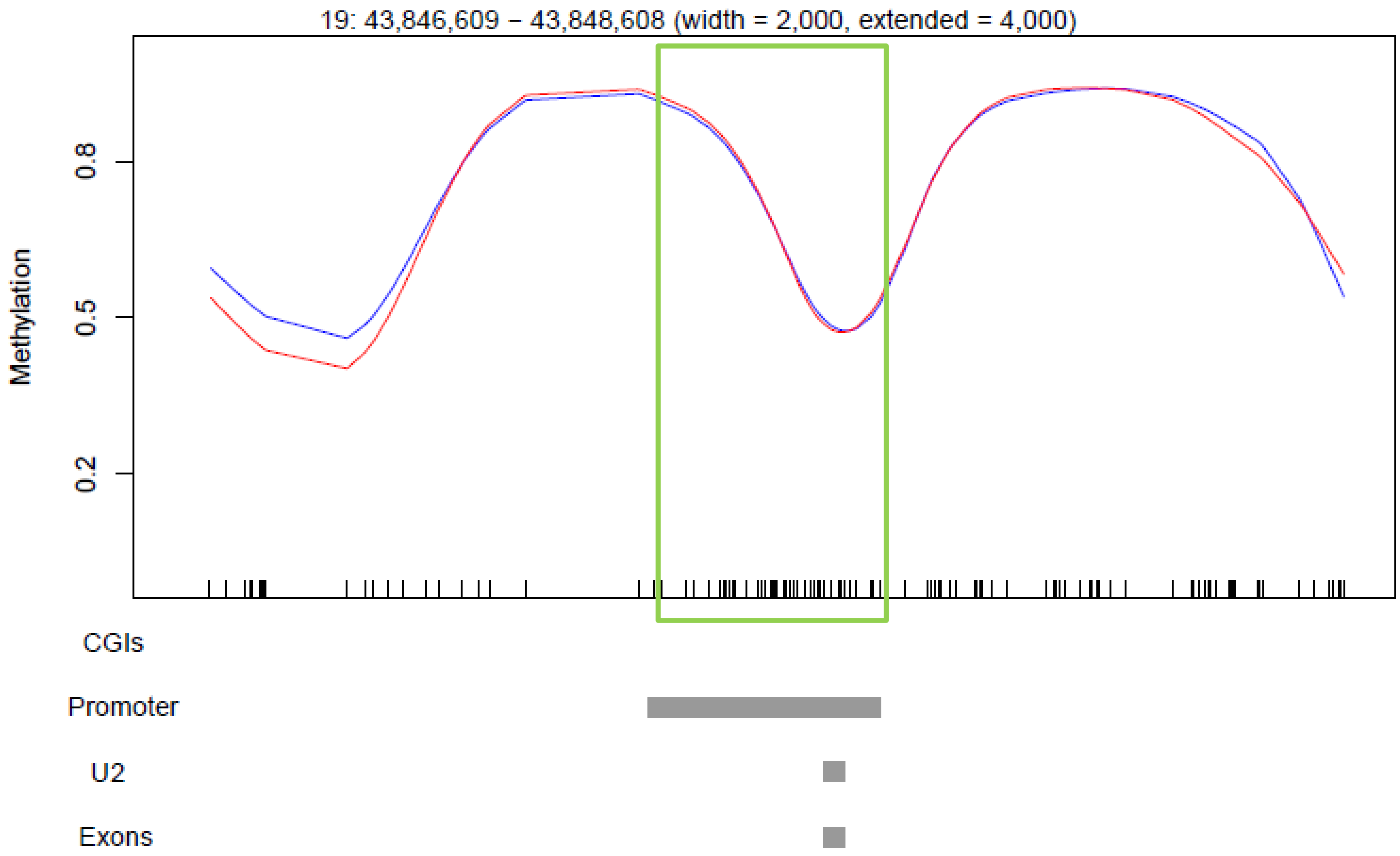
19: 43,846,609 − 43,848,608 (width = 2,000, extended = 4,000)
Methylation
0.8
0.5
0.2
CGIs
Promoter
U2
Exons

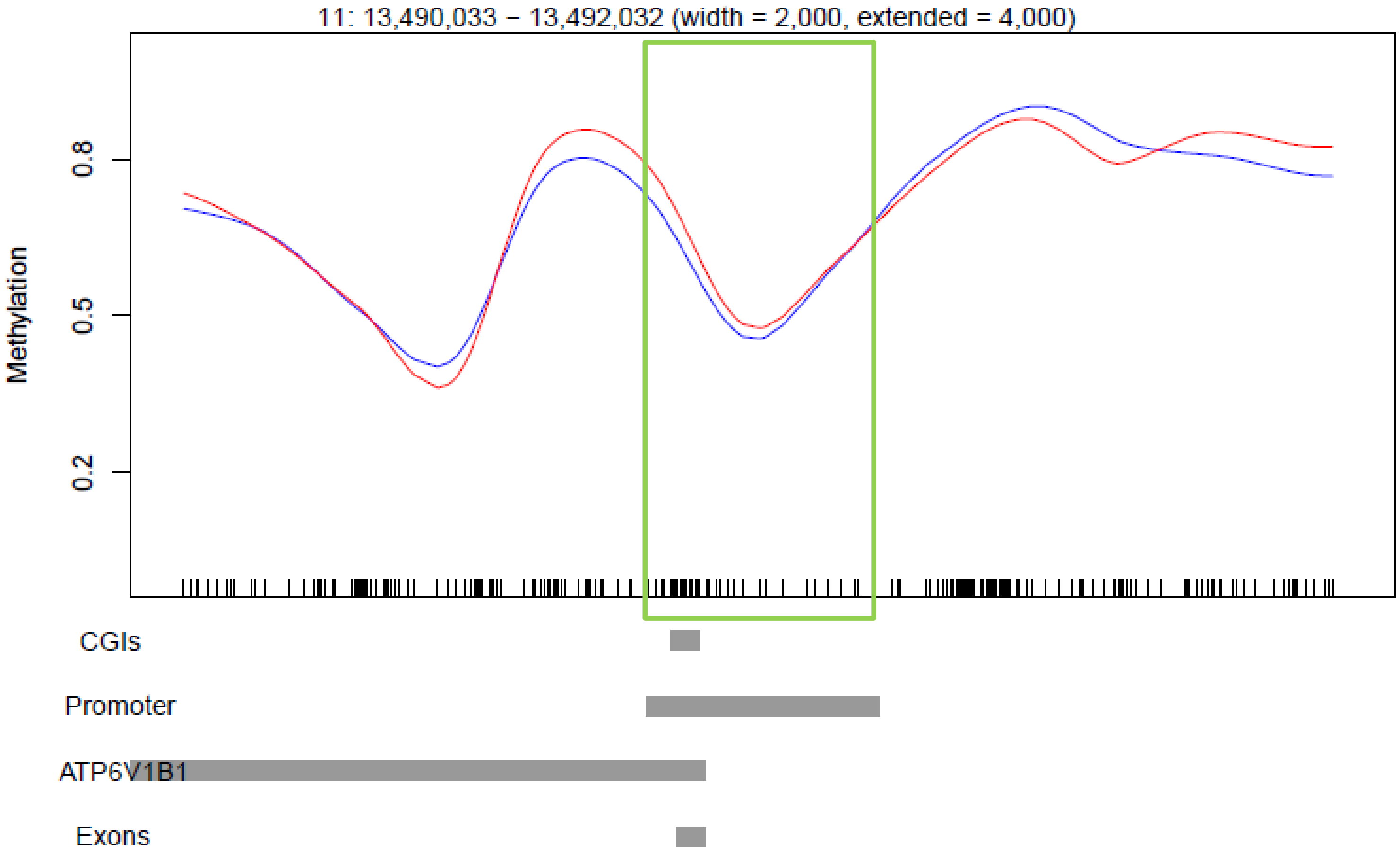

11: 13,490,033 – 13,492,032 (width = 2,000, extended = 4,000)
Methylation
0.8
0.5
0.2
CGIs
Promoter
ATP6V1B1
Exons

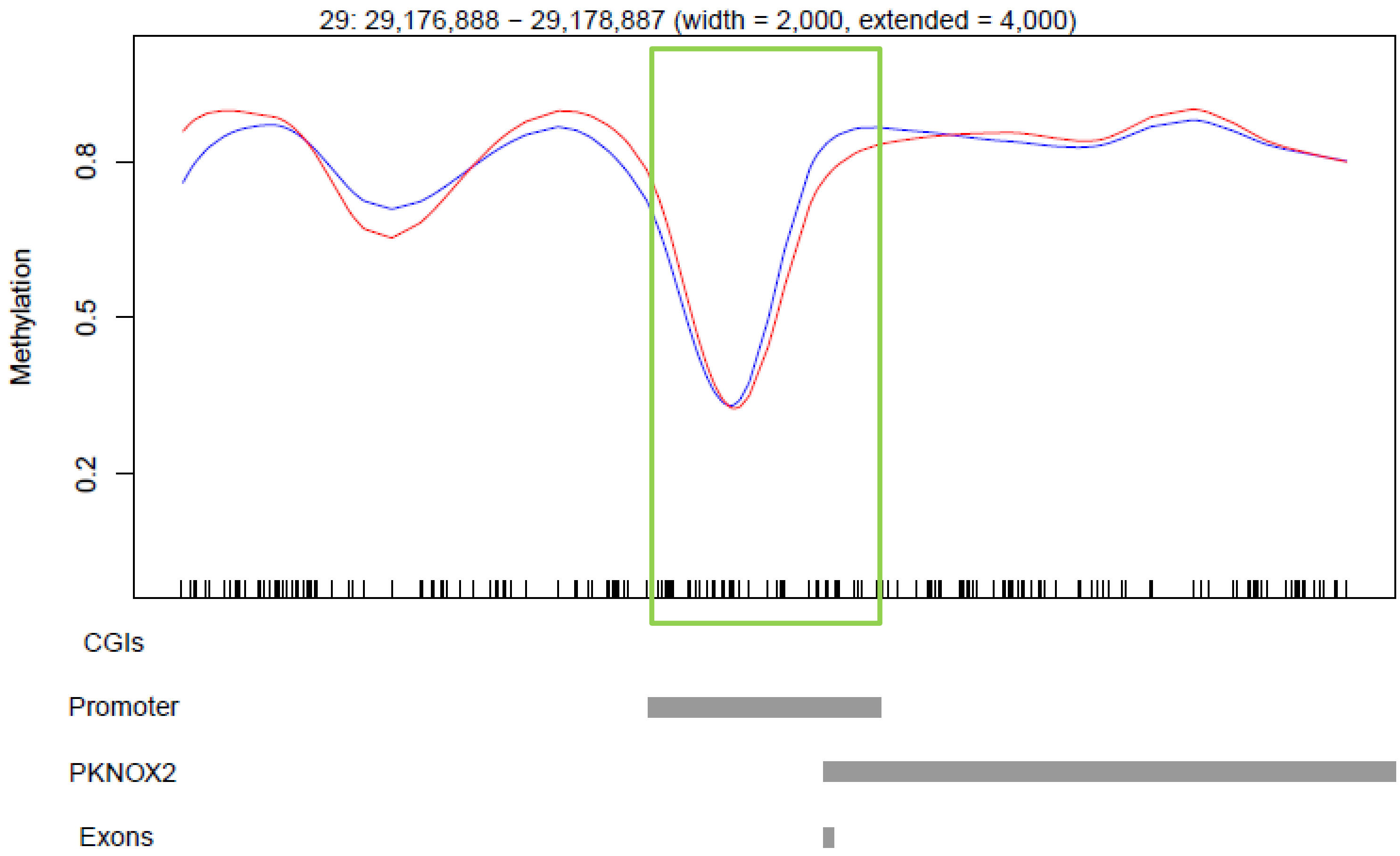
29: 29,176,888 − 29,178,887 (width = 2,000, extended = 4,000)
Methylation
0.8
0.5
0.2
CGIs
Promoter
PKNOX2
Exons

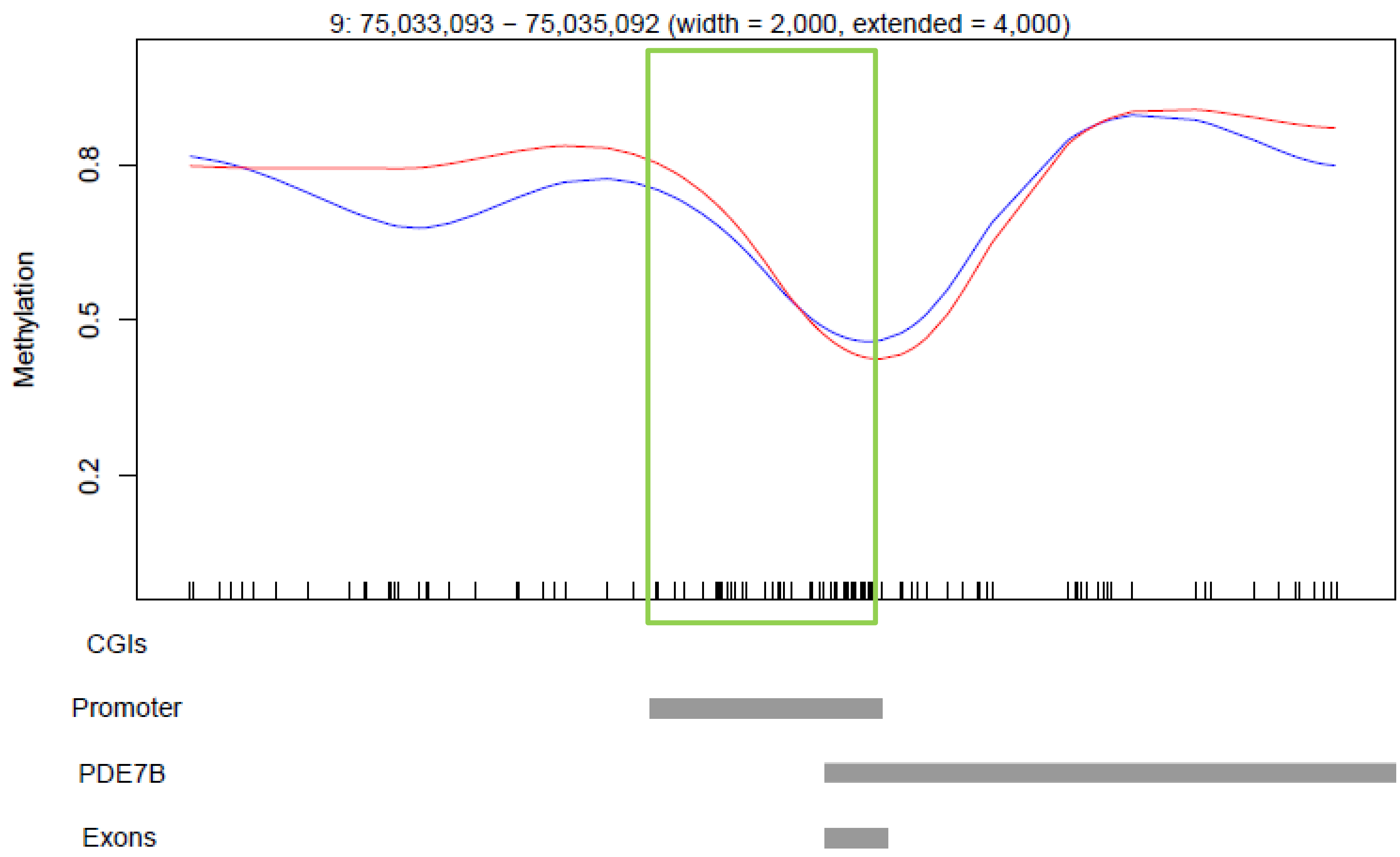

9: 75,033,093 – 75,035,092 (width = 2,000, extended = 4,000)
Methylation
0.8
0.5
0.2
CGIs
Promoter
PDE7B
Exons

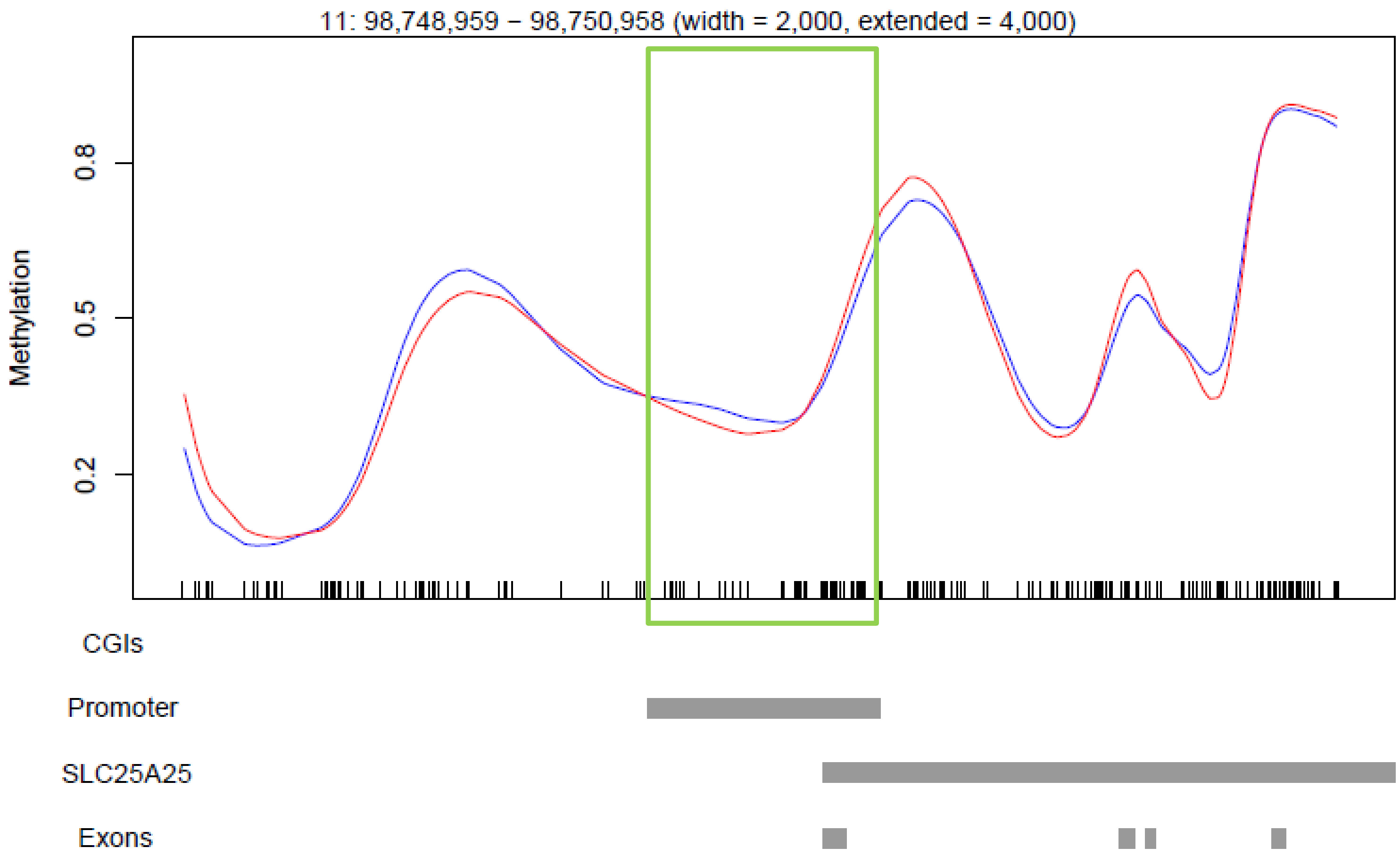

11: 98,748,959 – 98,750,958 (width = 2,000, extended = 4,000)
Methylation
0.2
0.5
0.8
CGIs
Promoter
SLC25A25
Exons

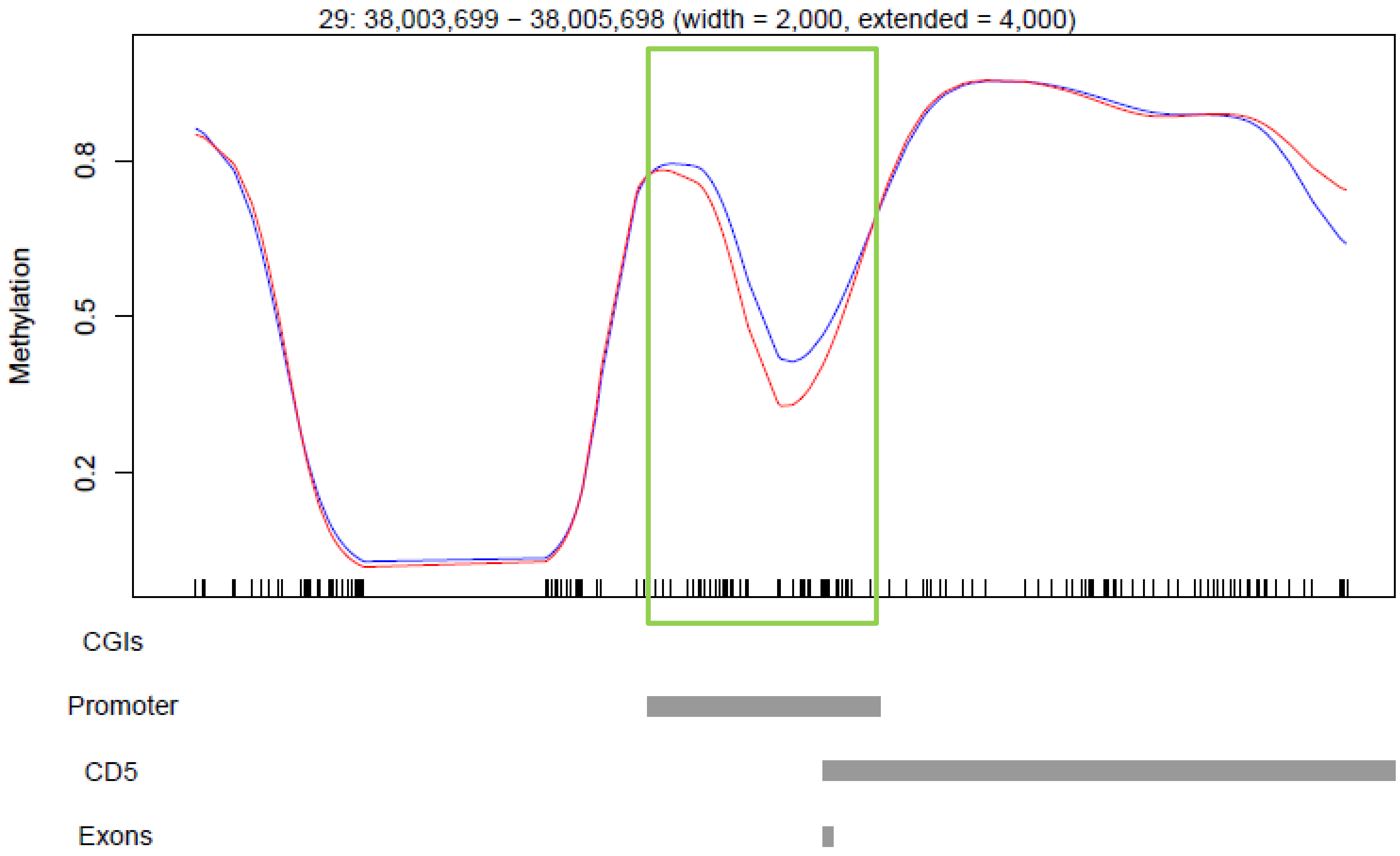

29: 38,003,699 – 38,005,698 (width = 2,000, extended = 4,000)
Methylation
0.8
0.5
0.2
CGIs
Promoter
CD5
Exons

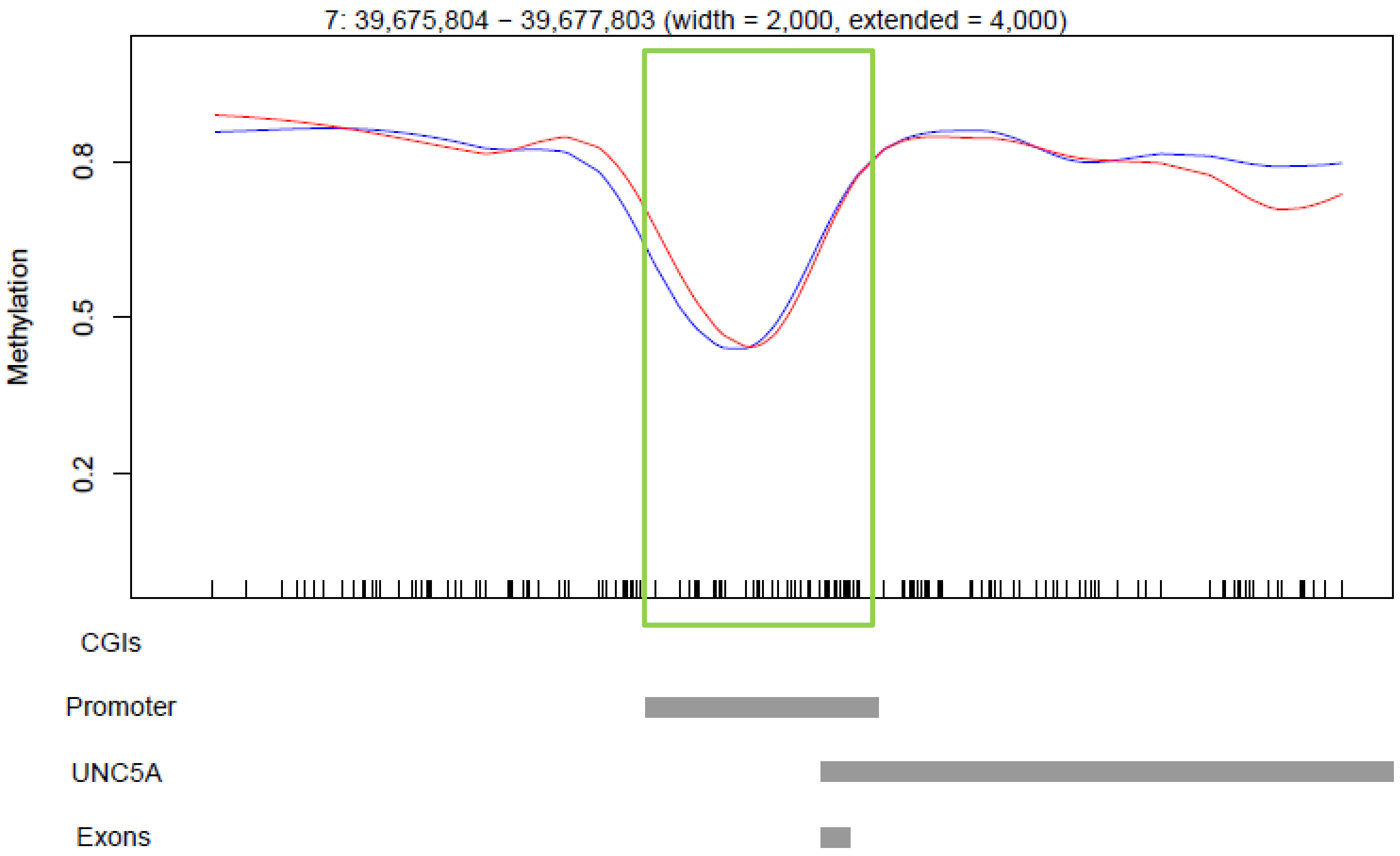

7: 39,675,804 − 39,677,803 (width = 2,000, extended = 4,000)
Methylation
0.8
0.5
0.2
CGIs
Promoter
UNC5A
Exons

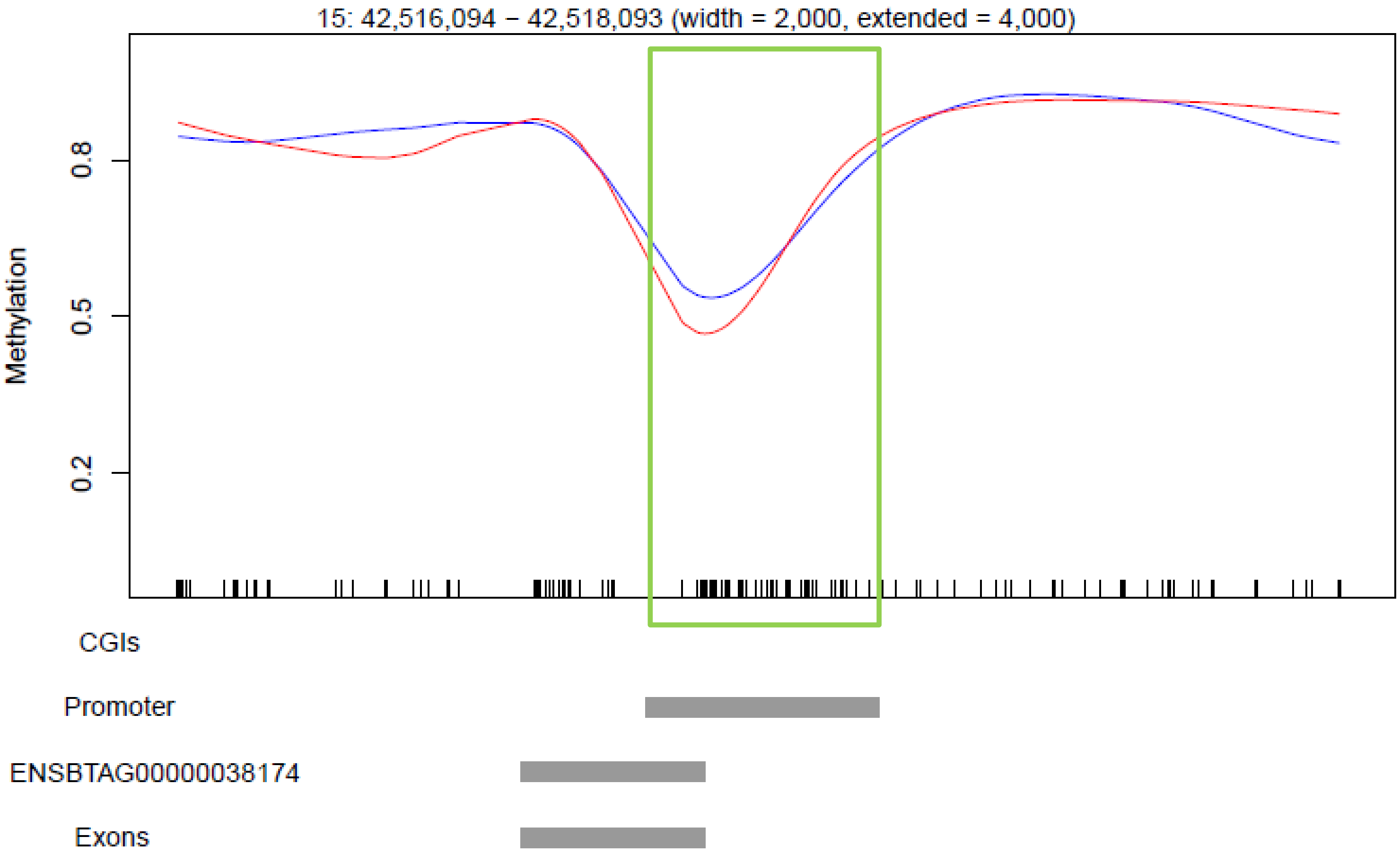

15: 42,516,094 − 42,518,093 (width = 2,000, extended = 4,000)
Methylation
0.8
0.5
0.2
CGIs
Promoter
ENSBTAG00000038174
Exons

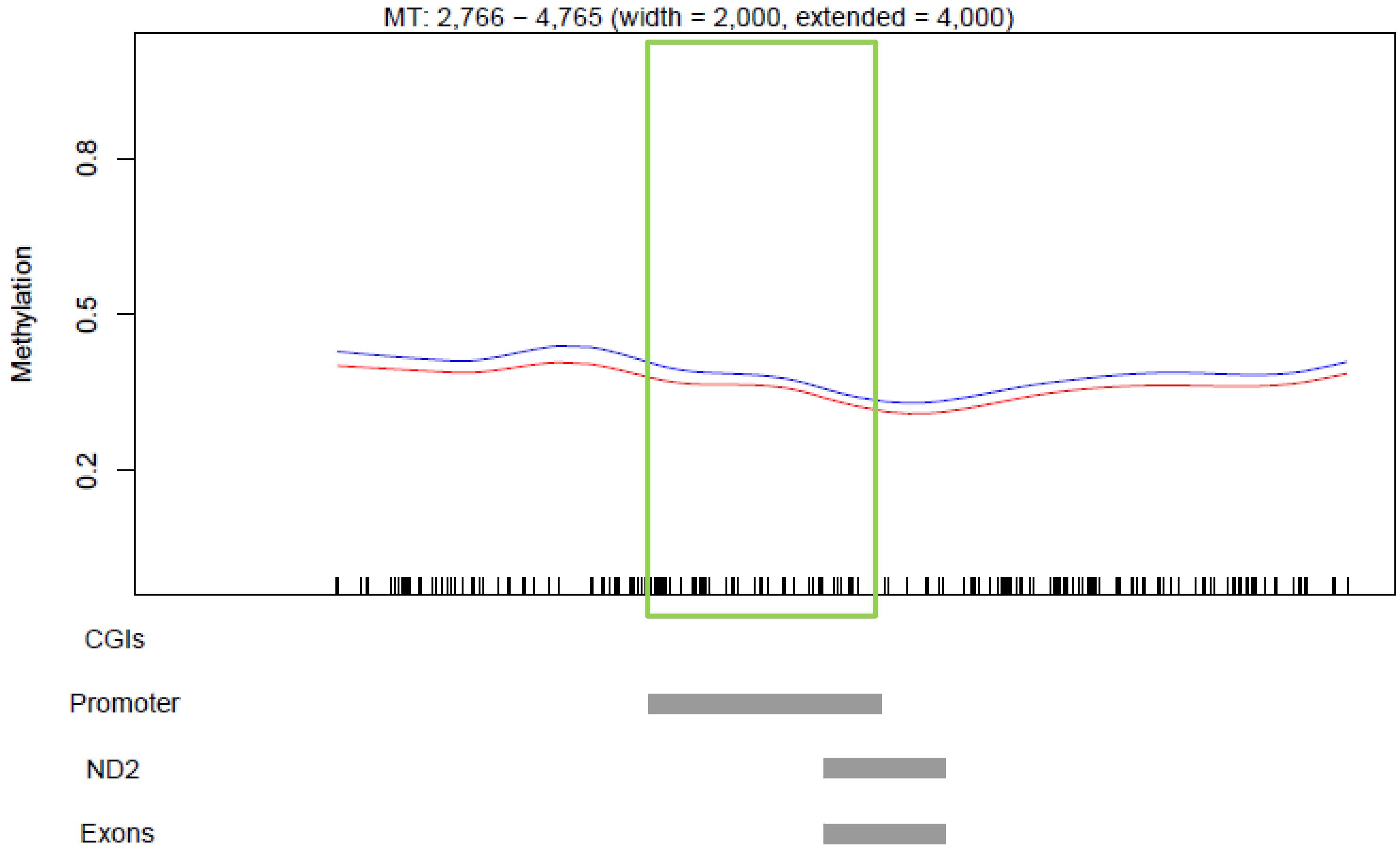
MT: 2,766 − 4,765 (width = 2,000, extended = 4,000)
Methylation
0.8
0.5
0.2
CGIs
Promoter
ND2
Exons

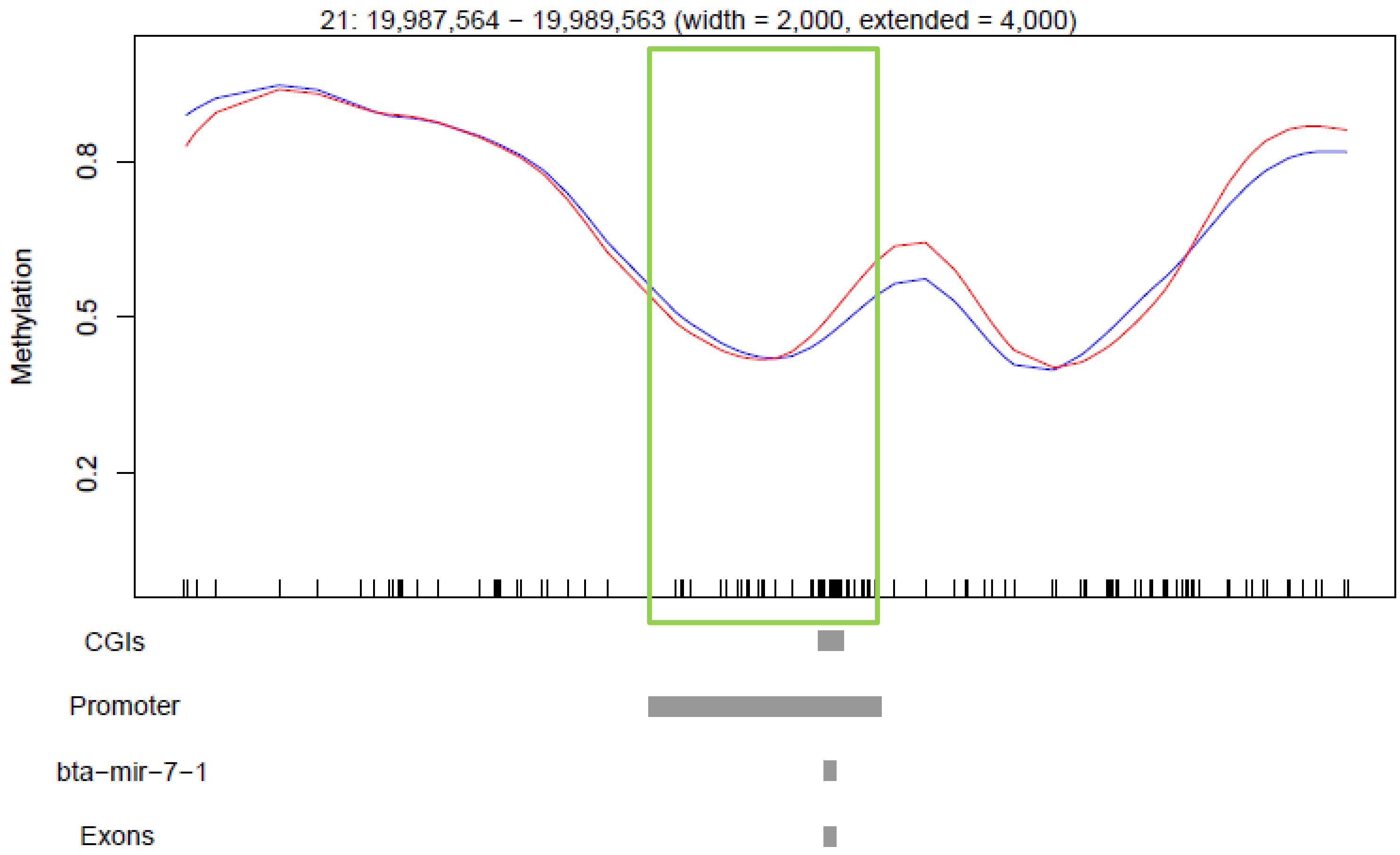
21: 19,987,564 − 19,989,563 (width = 2,000, extended = 4,000)
Methylation
0.2
0.5
0.8
CGIs
Promoter
bta−mir−7−1
Exons

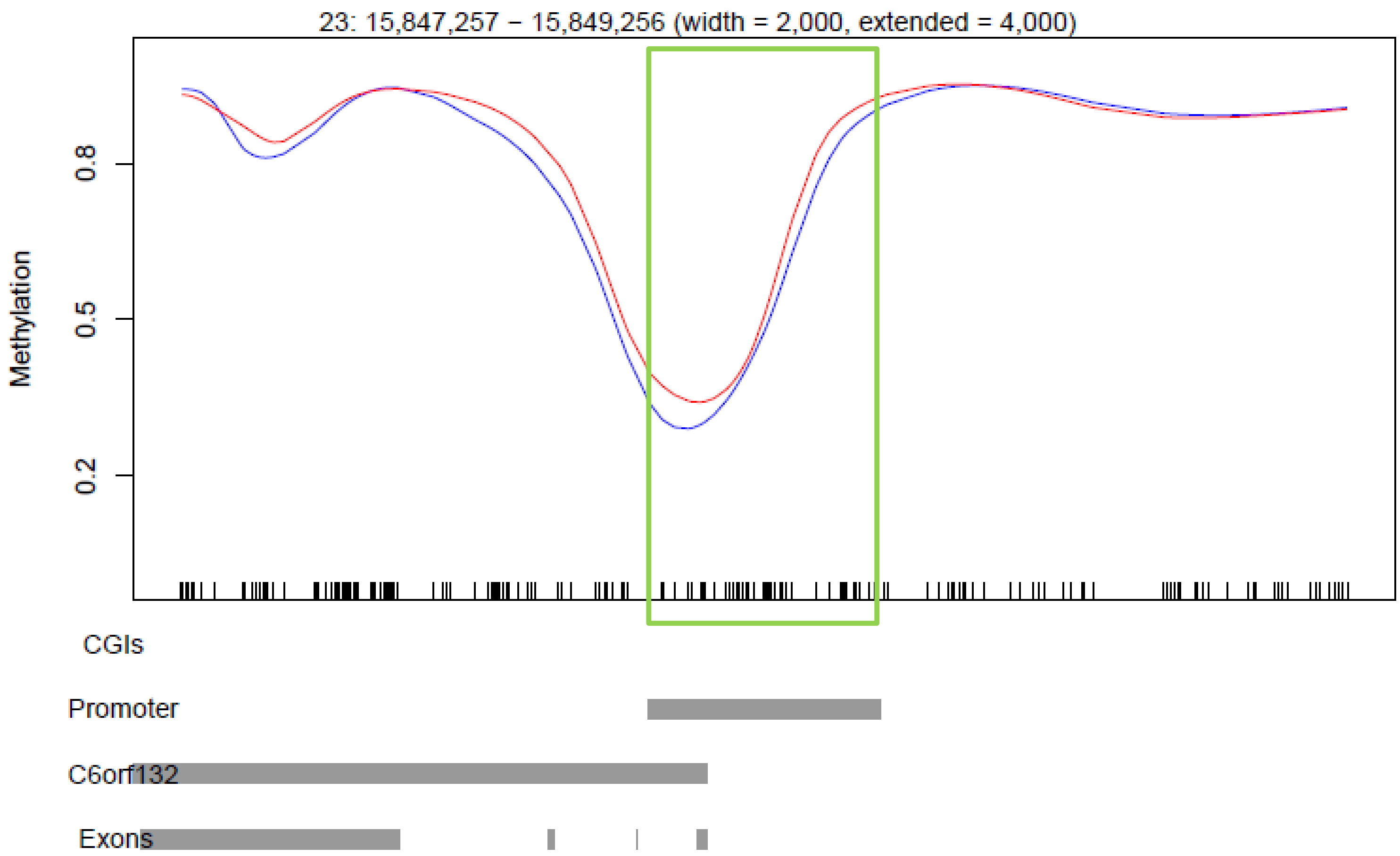

23: 15,847,257 − 15,849,256 (width = 2,000, extended = 4,000)
Methylation
0.8
0.5
0.2
CGIs
Promoter
C6orf132
Exons

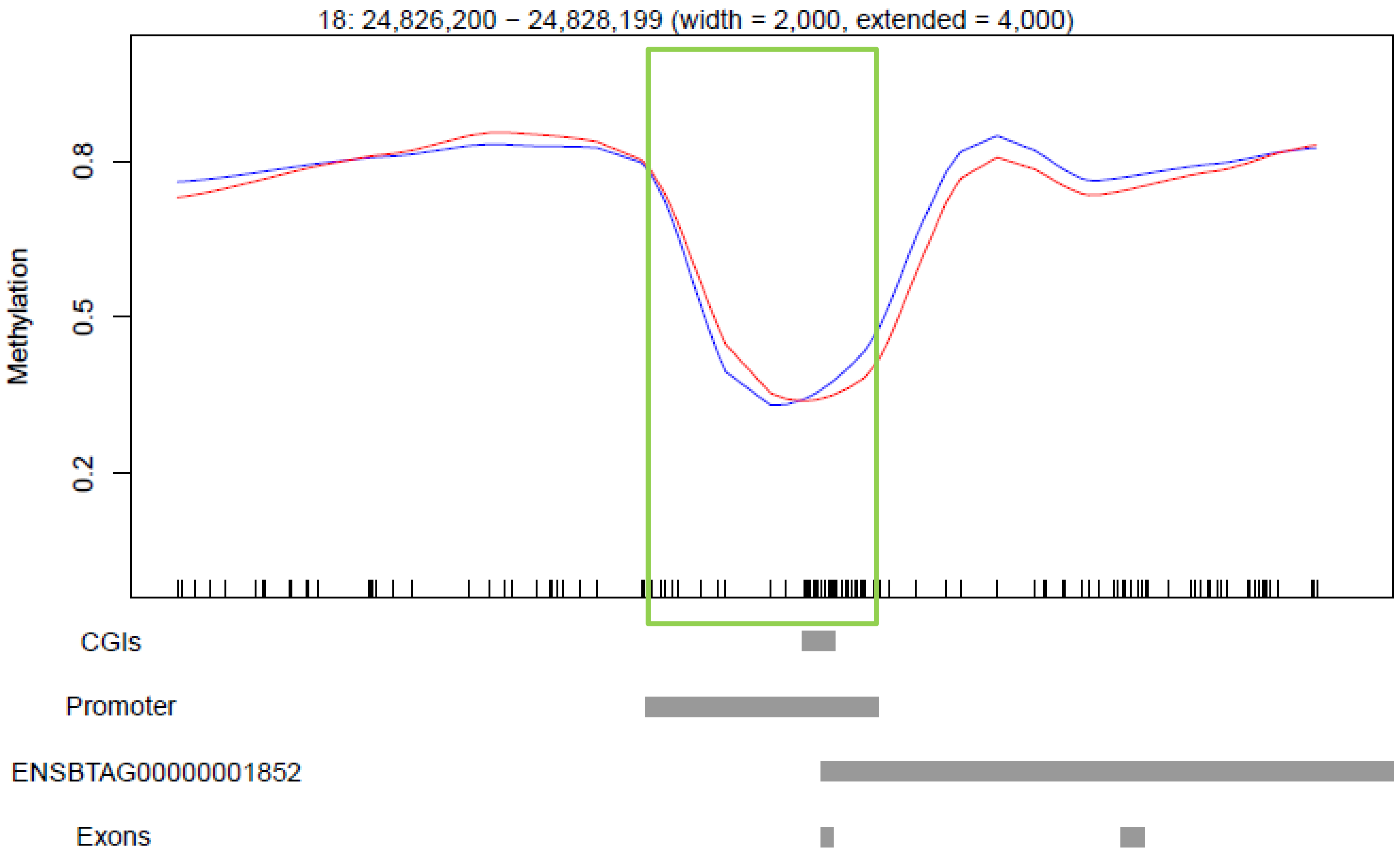
18: 24,826,200 − 24,828,199 (width = 2,000, extended = 4,000)
Methylation
0.8
0.5
0.2
CGIs
Promoter
ENSBTAG00000001852
Exons

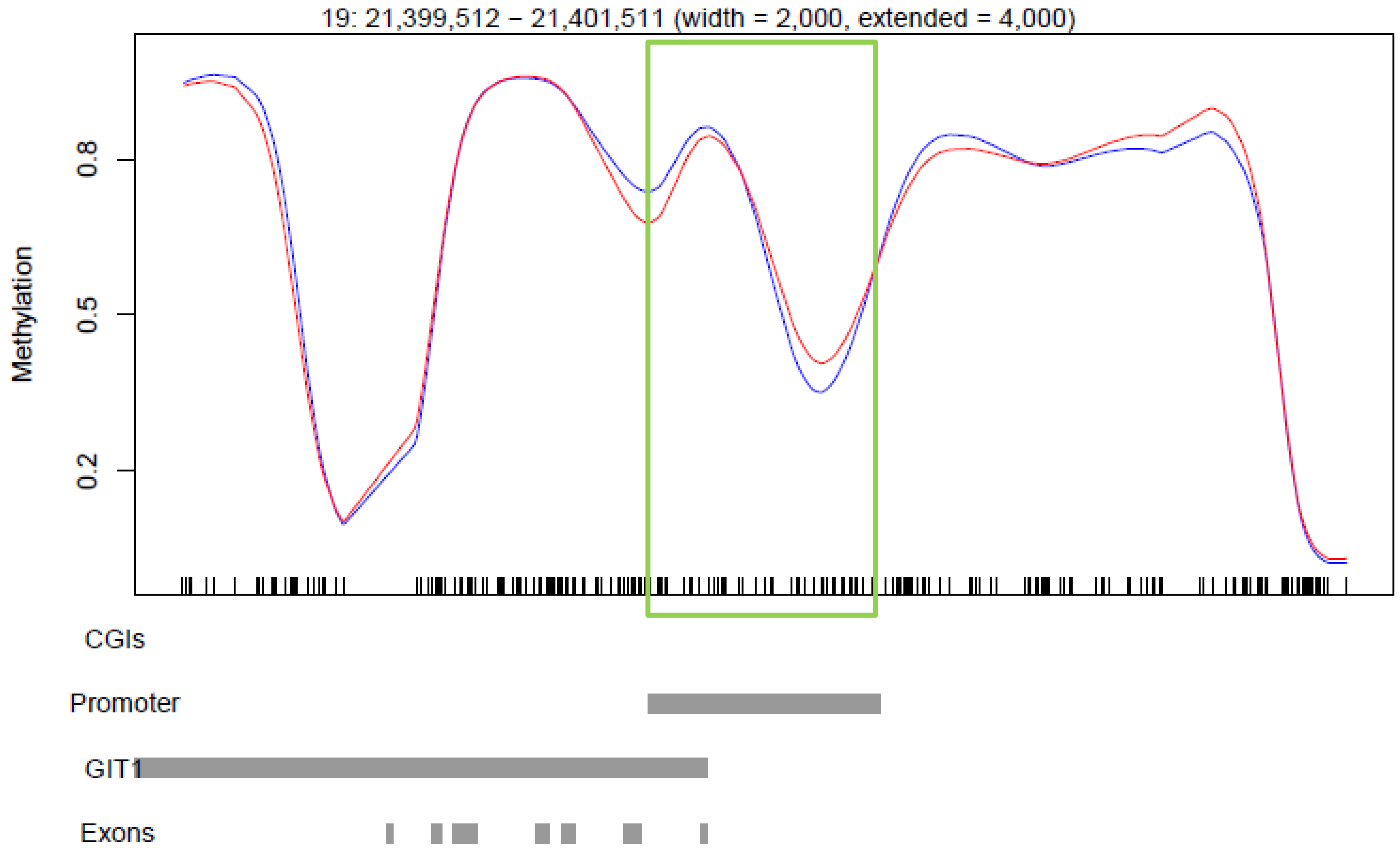

19: 21,399,512 – 21,401,511 (width = 2,000, extended = 4,000)
Methylation
0.8
0.5
0.2
CGIs
Promoter
GIT1
Exons

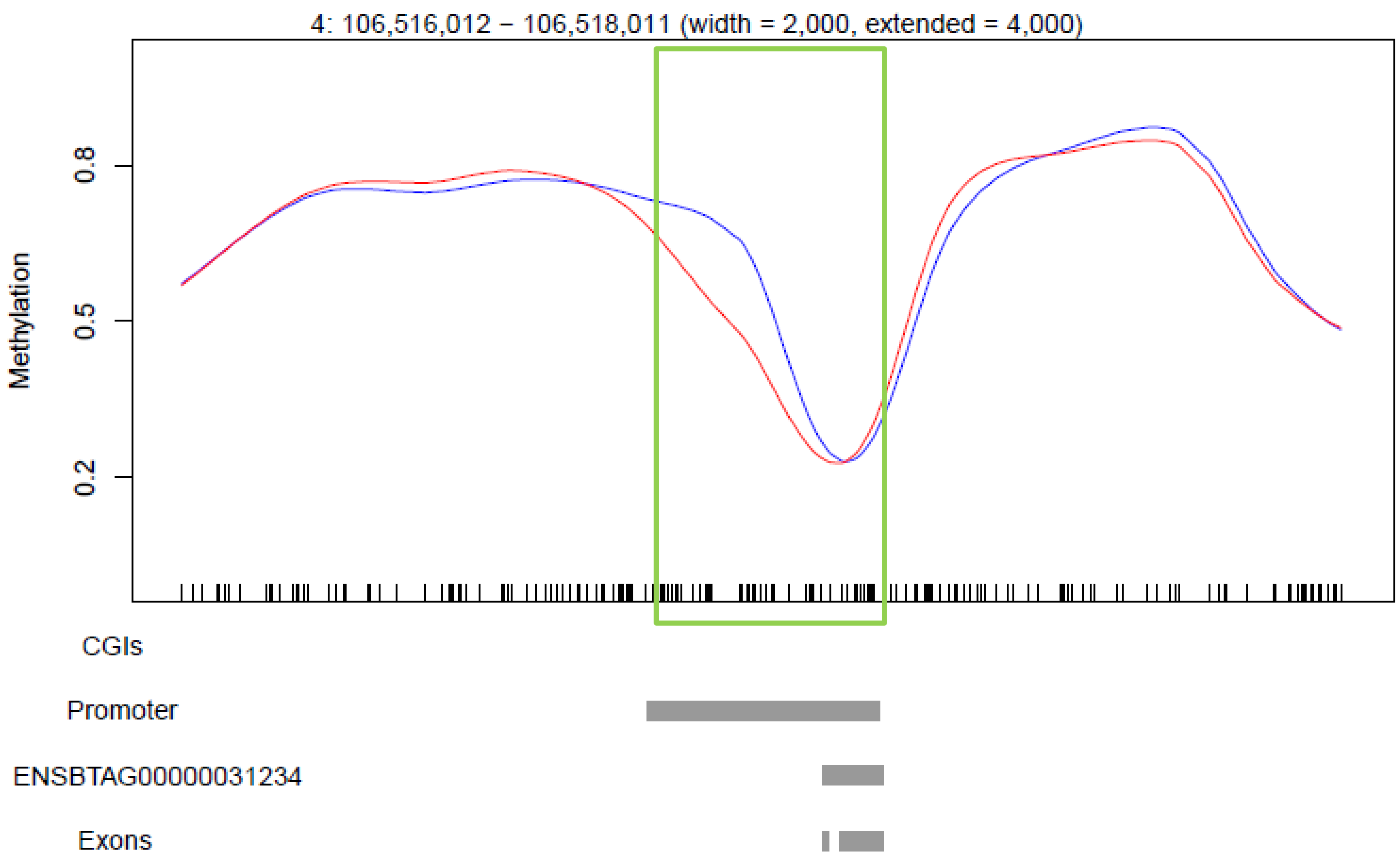
4: 106,516,012 – 106,518,011 (width = 2,000, extended = 4,000)
Methylation
0.2
0.5
0.8
CGIs
Promoter
ENSBTAG00000031234
Exons

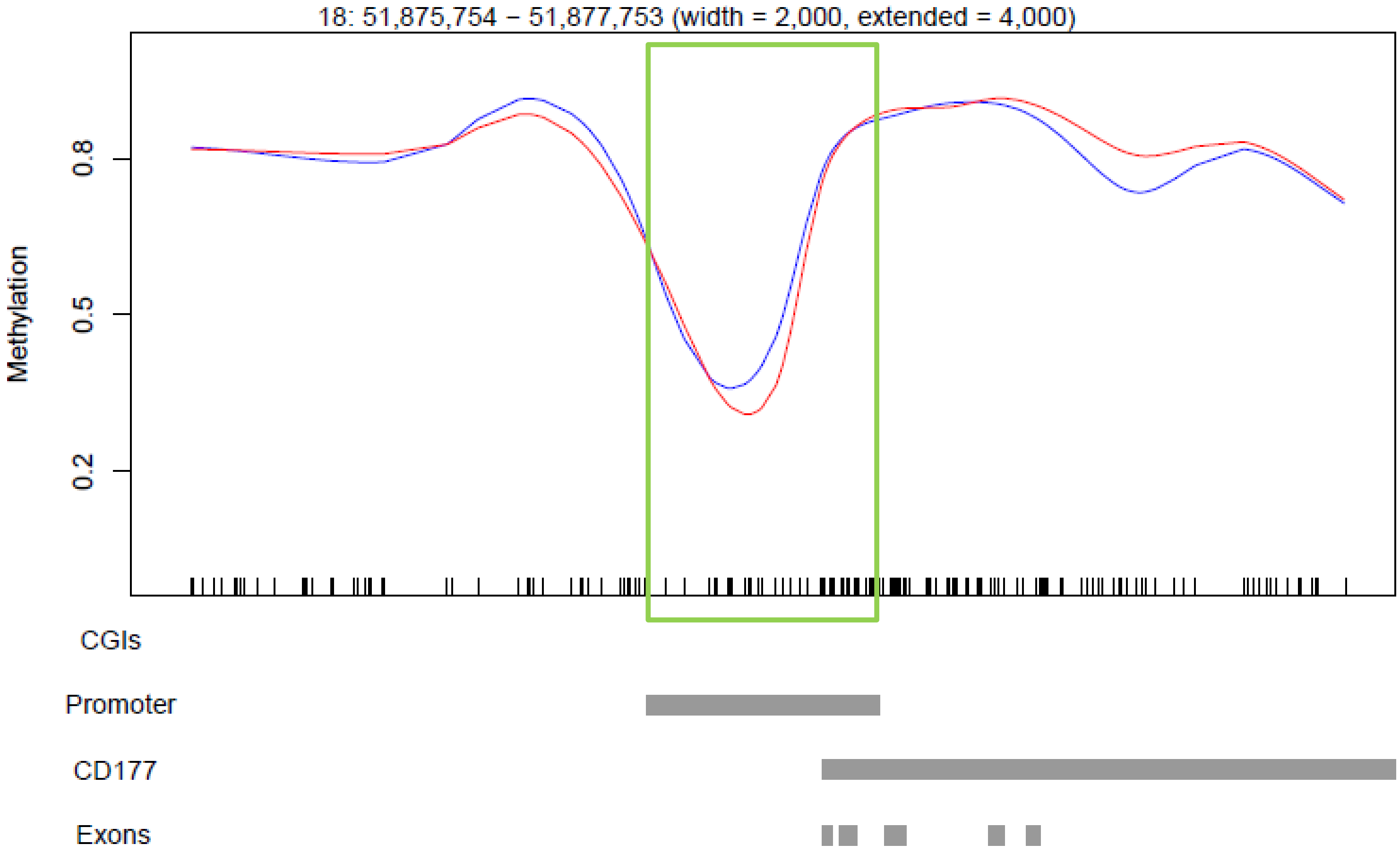
18: 51,875,754 − 51,877,753 (width = 2,000, extended = 4,000)
Methylation
0.8
0.5
0.2
CGIs
Promoter
CD177
Exons

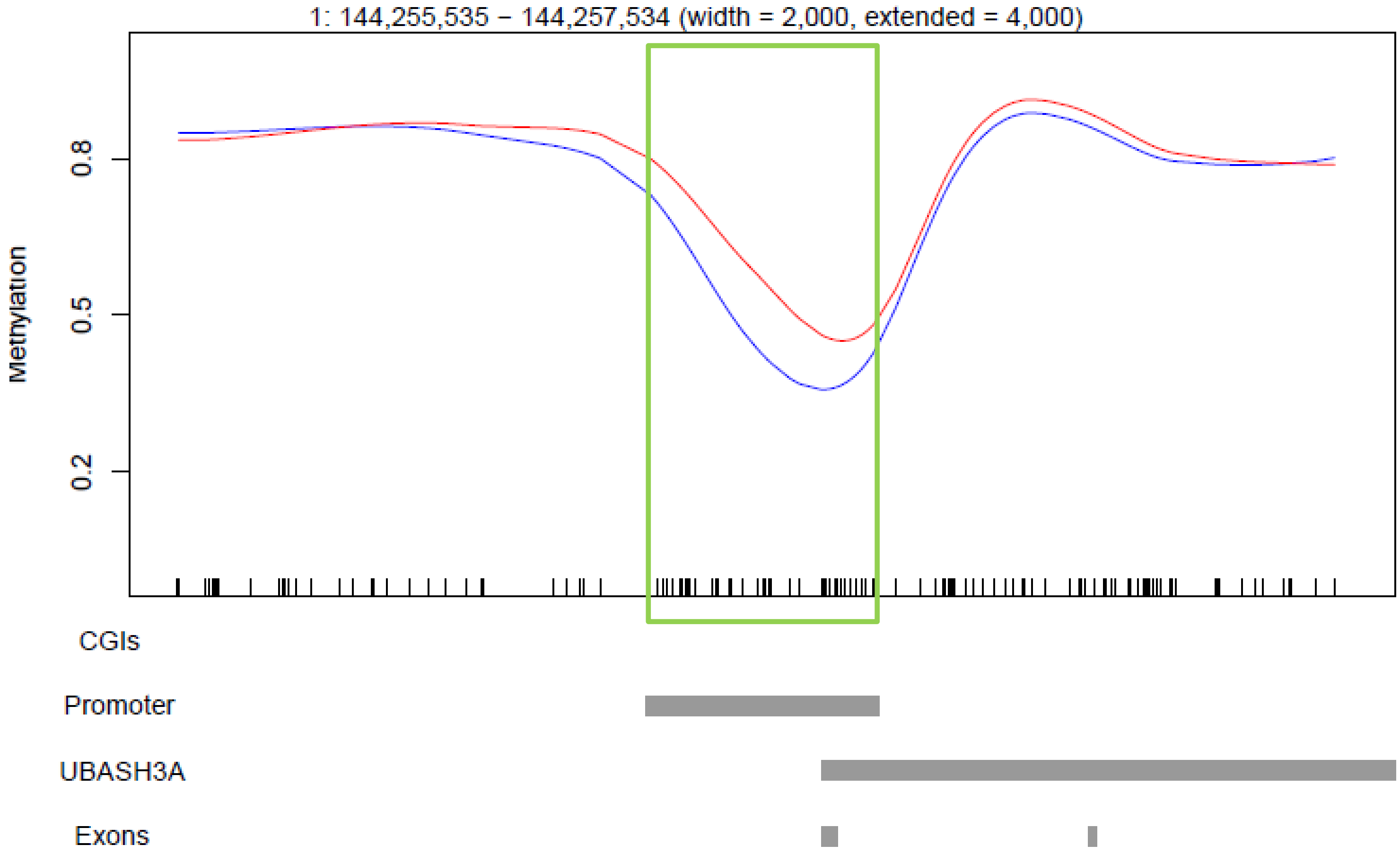

1: 144,255,535 − 144,257,534 (width = 2,000, extended = 4,000)
Methylation
0.8
0.5
0.2
CGIs
Promoter
UBASH3A
Exons

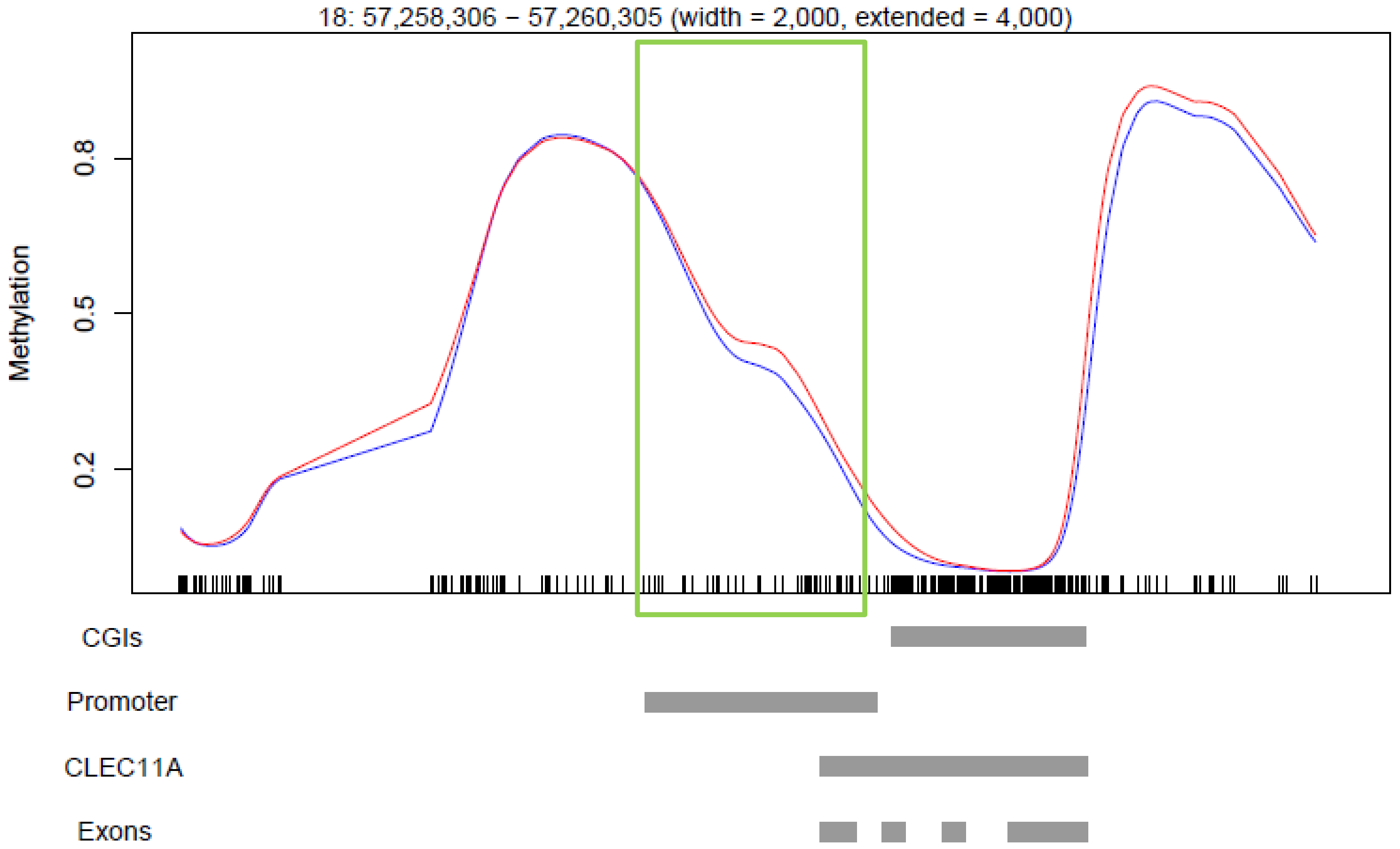

18: 57,258,306 − 57,260,305 (width = 2,000, extended = 4,000)
Methylation
0.2
0.5
0.8
CGIs
Promoter
CLEC11A
Exons

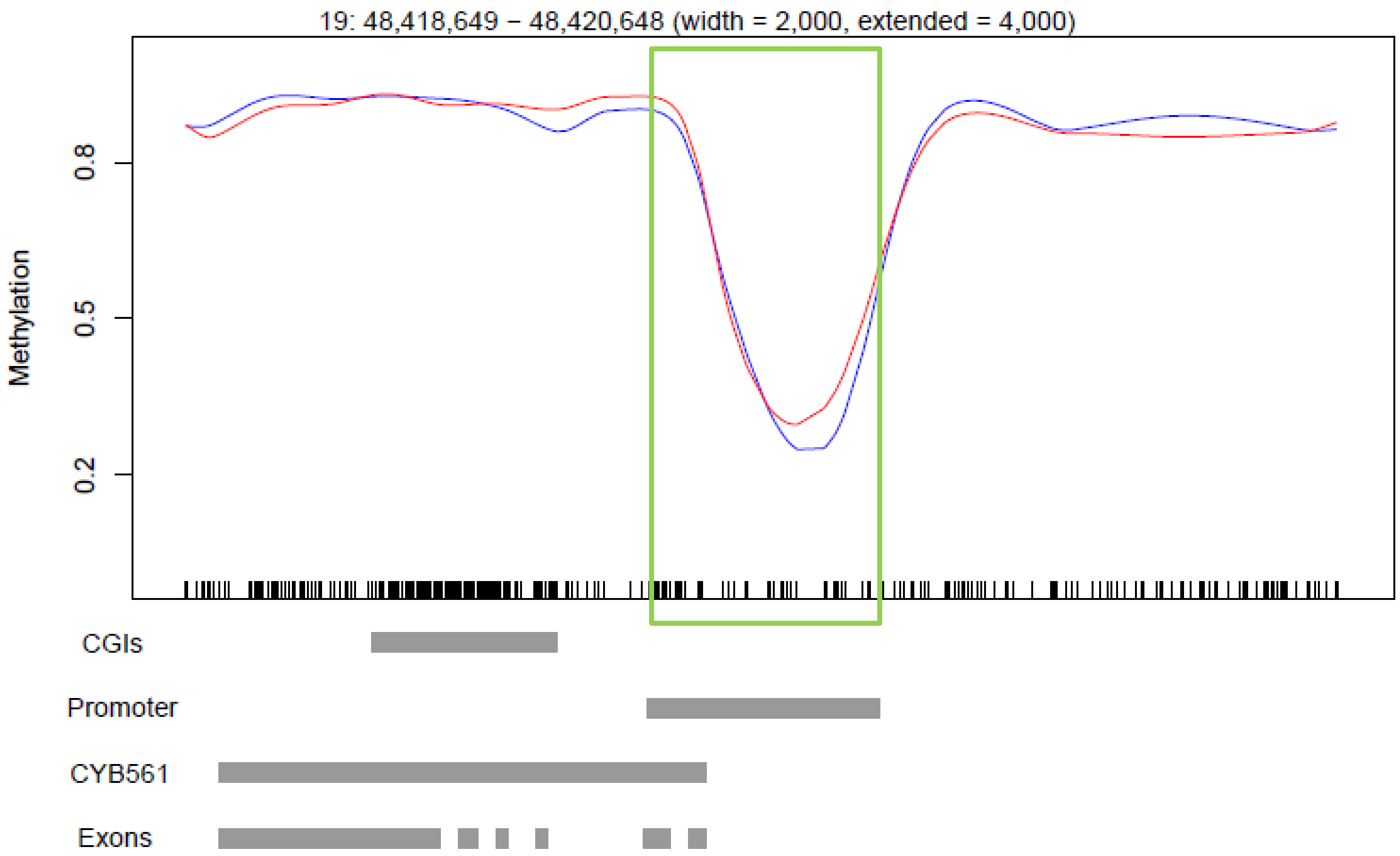
19: 48,418,649 − 48,420,648 (width = 2,000, extended = 4,000)
Methylation
0.8
0.5
0.2
CGIs
Promoter
CYB561
Exons

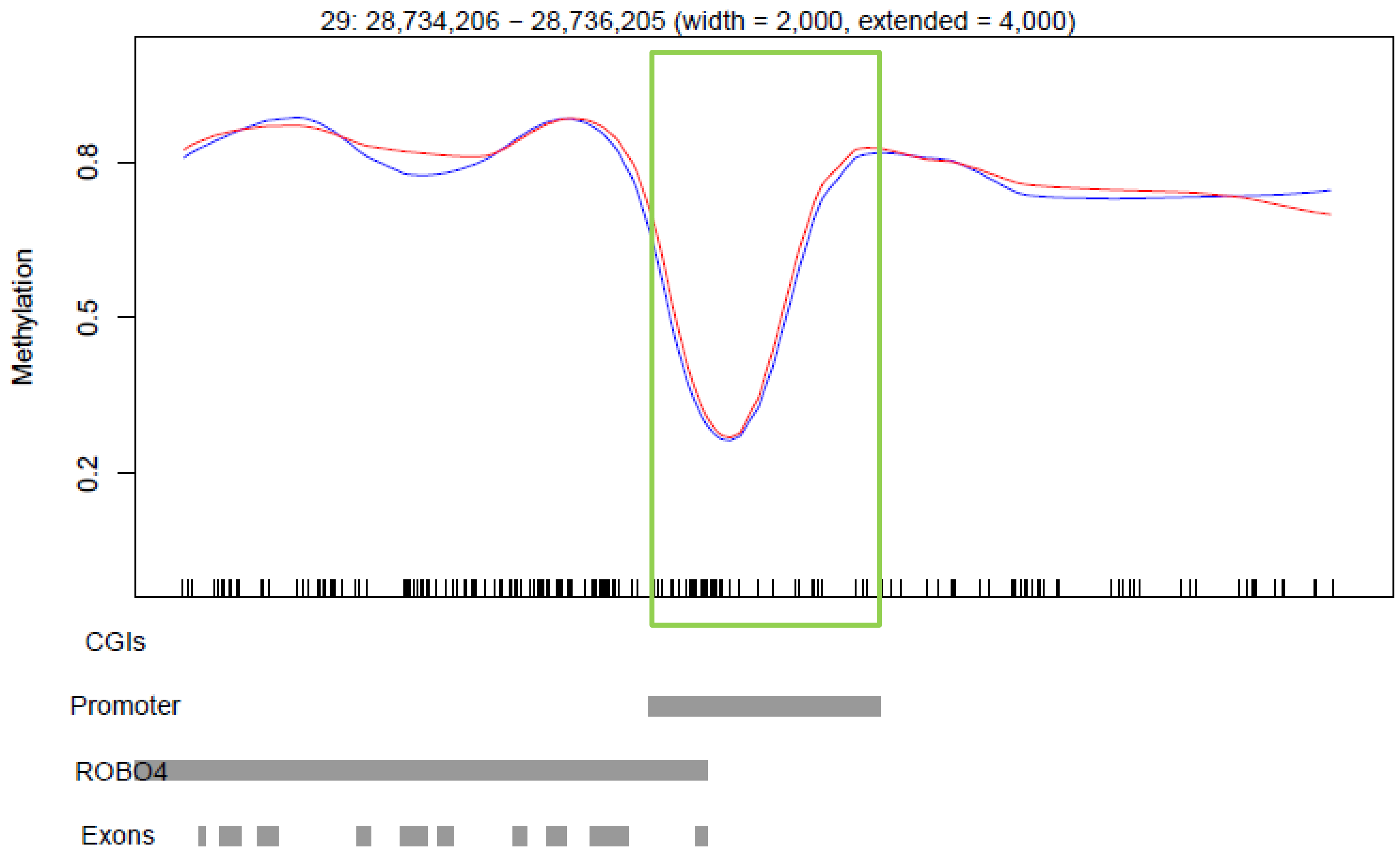

29: 28,734,206 − 28,736,205 (width = 2,000, extended = 4,000)
Methylation
0.8
0.5
0.2
CGIs
Promoter
ROBO4
Exons

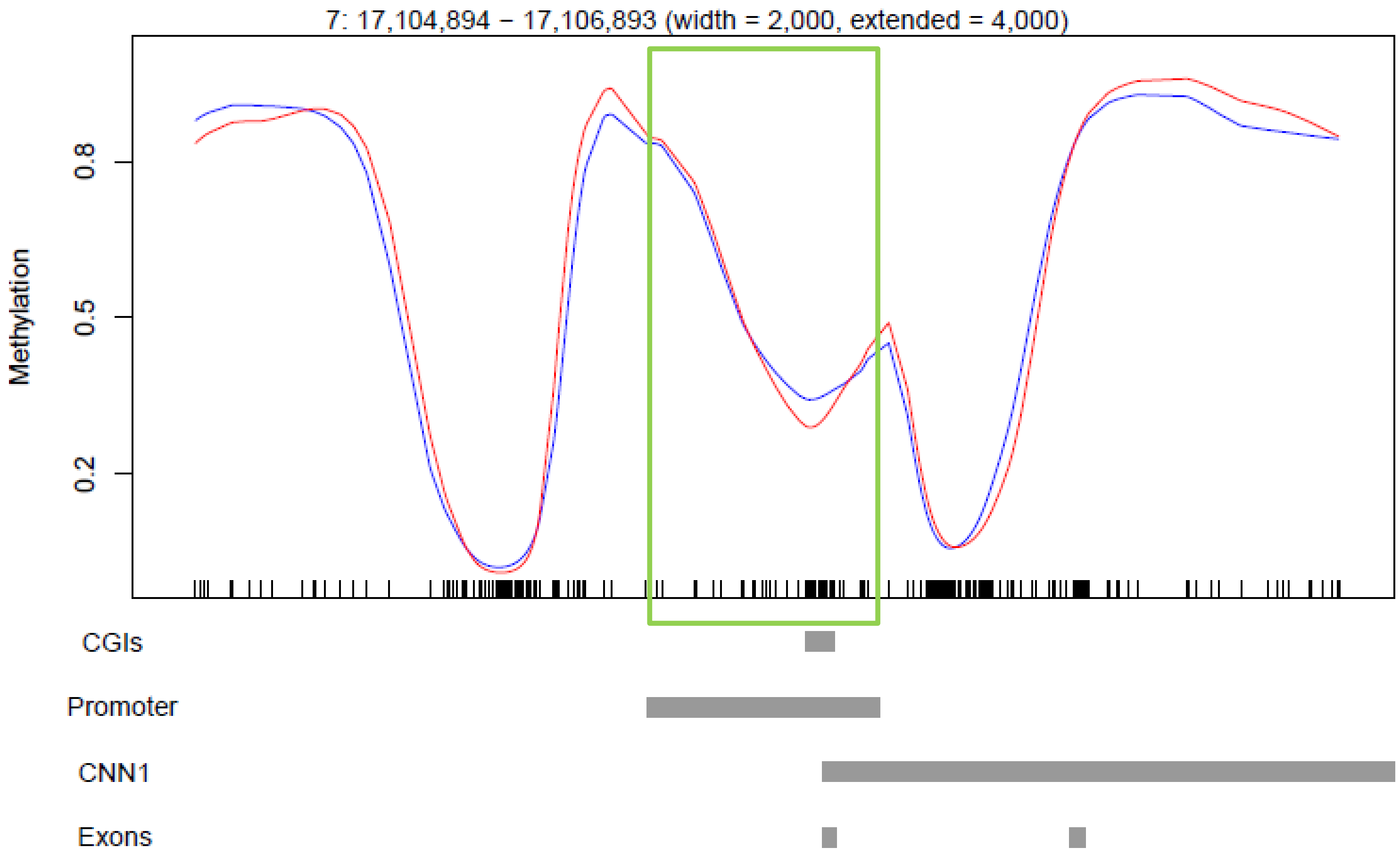
7: 17,104,894 − 17,106,893 (width = 2,000, extended = 4,000)
Methylation
0.8
0.5
0.2
CGIs
Promoter
CNN1
Exons

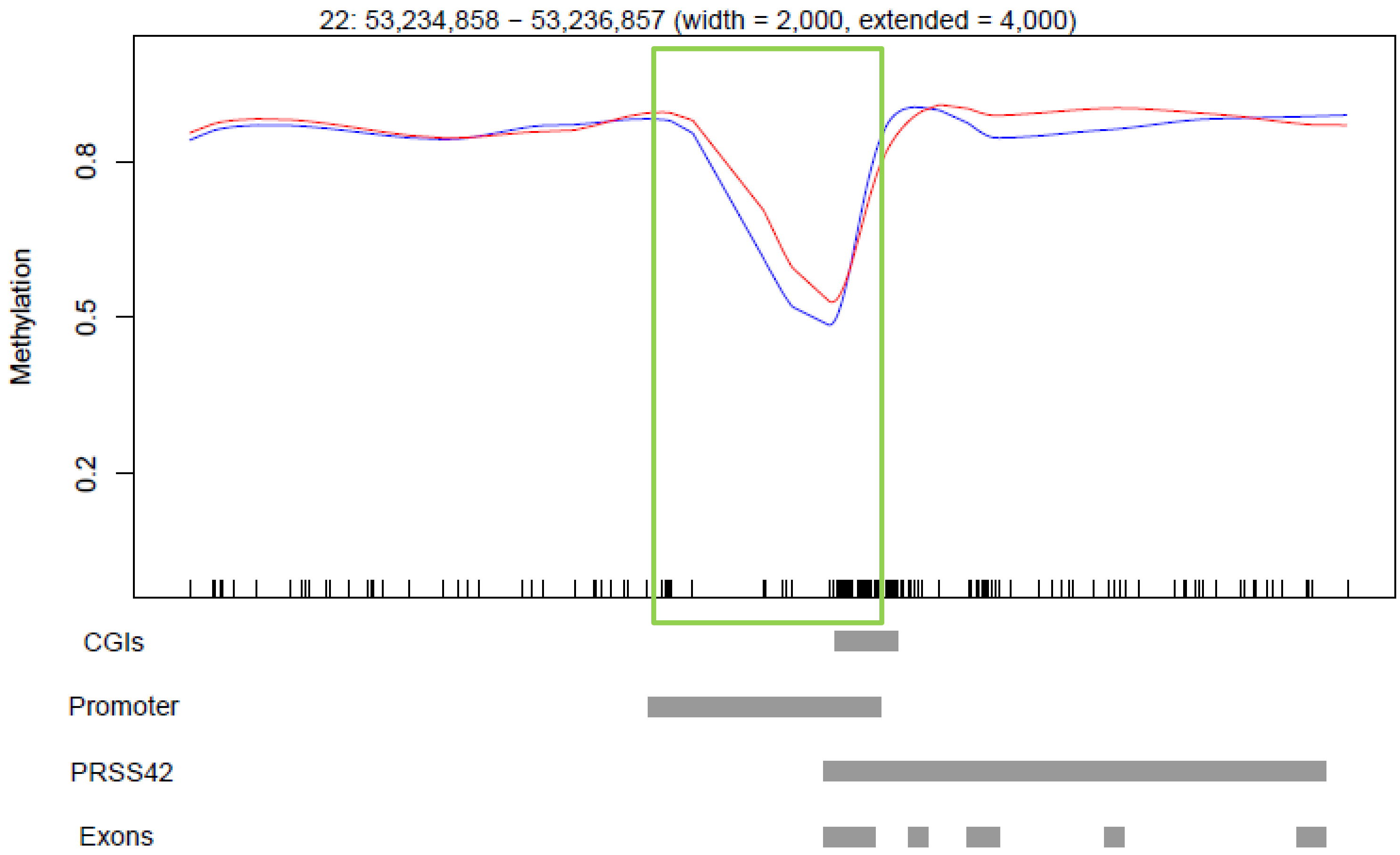

22: 53,234,858 − 53,236,857 (width = 2,000, extended = 4,000)
Methylation
0.8
0.5
0.2
CGIs
Promoter
PRSS42
Exons

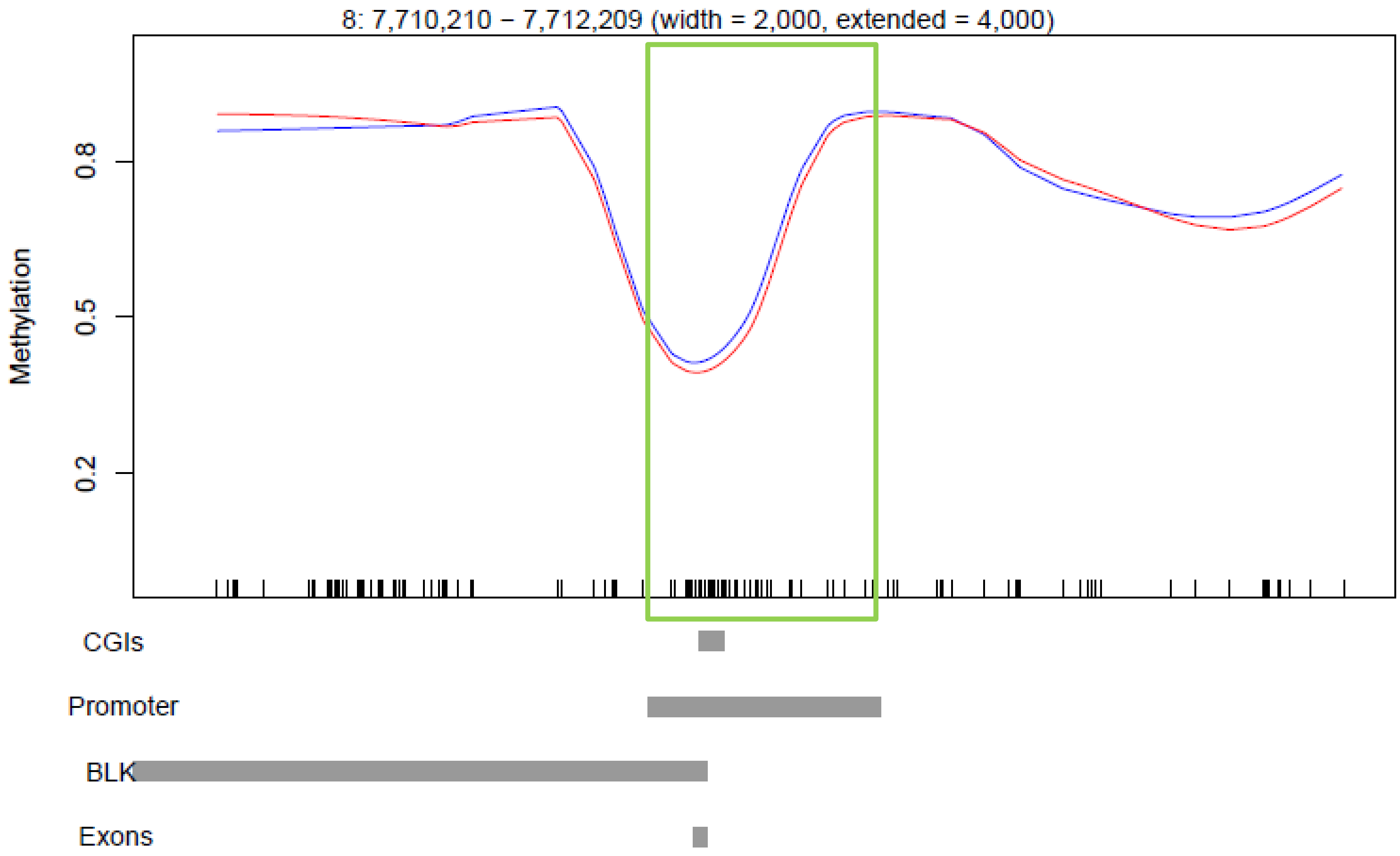

8: 7,710,210 – 7,712,209 (width = 2,000, extended = 4,000)
Methylation
0.8
0.5
0.2
CGIs
Promoter
BLK
Exons

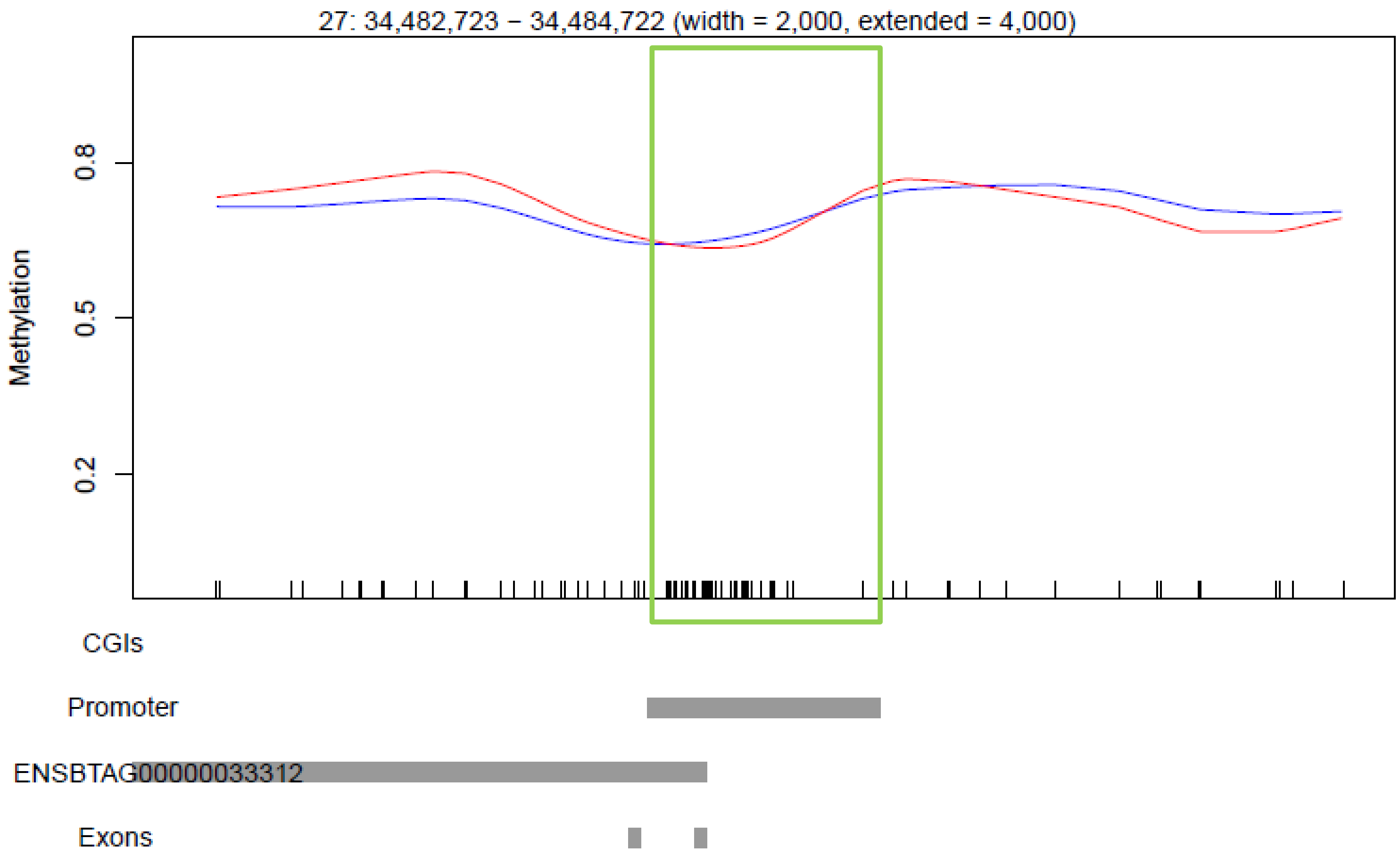
27: 34,482,723 − 34,484,722 (width = 2,000, extended = 4,000)
Methylation
0.8
0.5
0.2
CGIs
Promoter
ENSBTAG00000033312
Exons

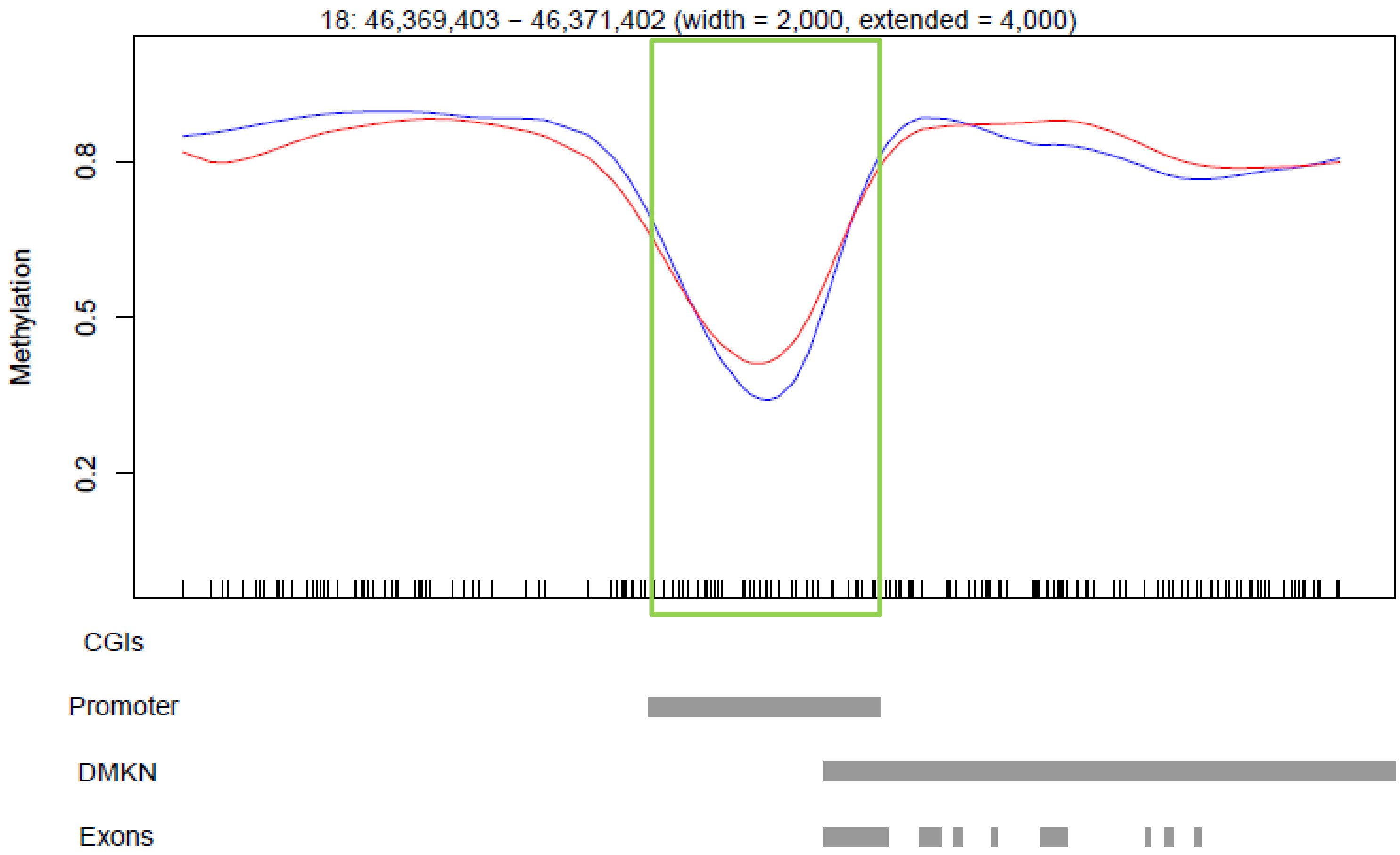

18: 46,369,403 − 46,371,402 (width = 2,000, extended = 4,000)
Methylation
0.8
0.5
0.2
CGIs
Promoter
DMKN
Exons

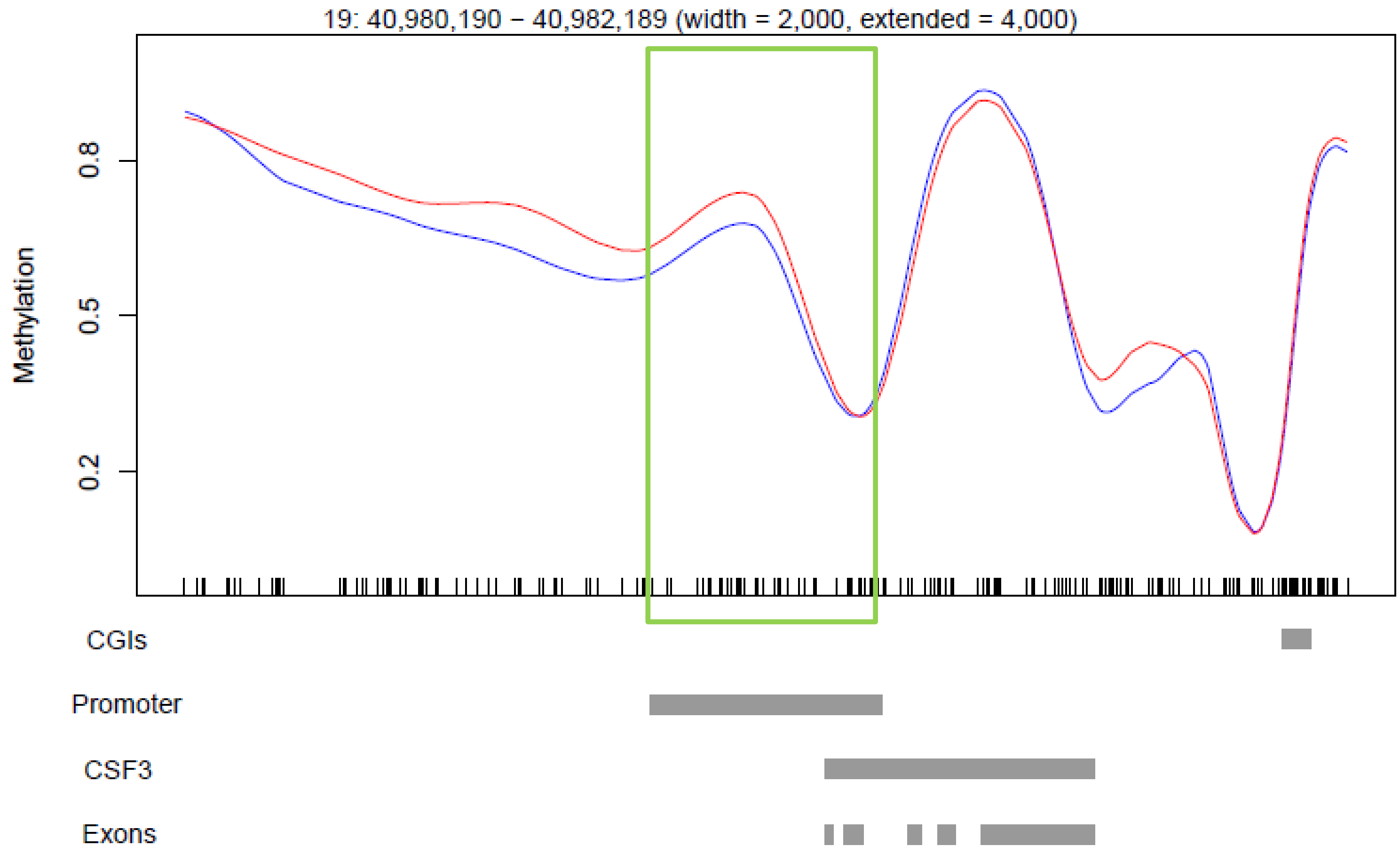
19: 40,980,190 − 40,982,189 (width = 2,000, extended = 4,000)
Methylation
0.8
0.5
0.2
CGIs
Promoter
CSF3
Exons

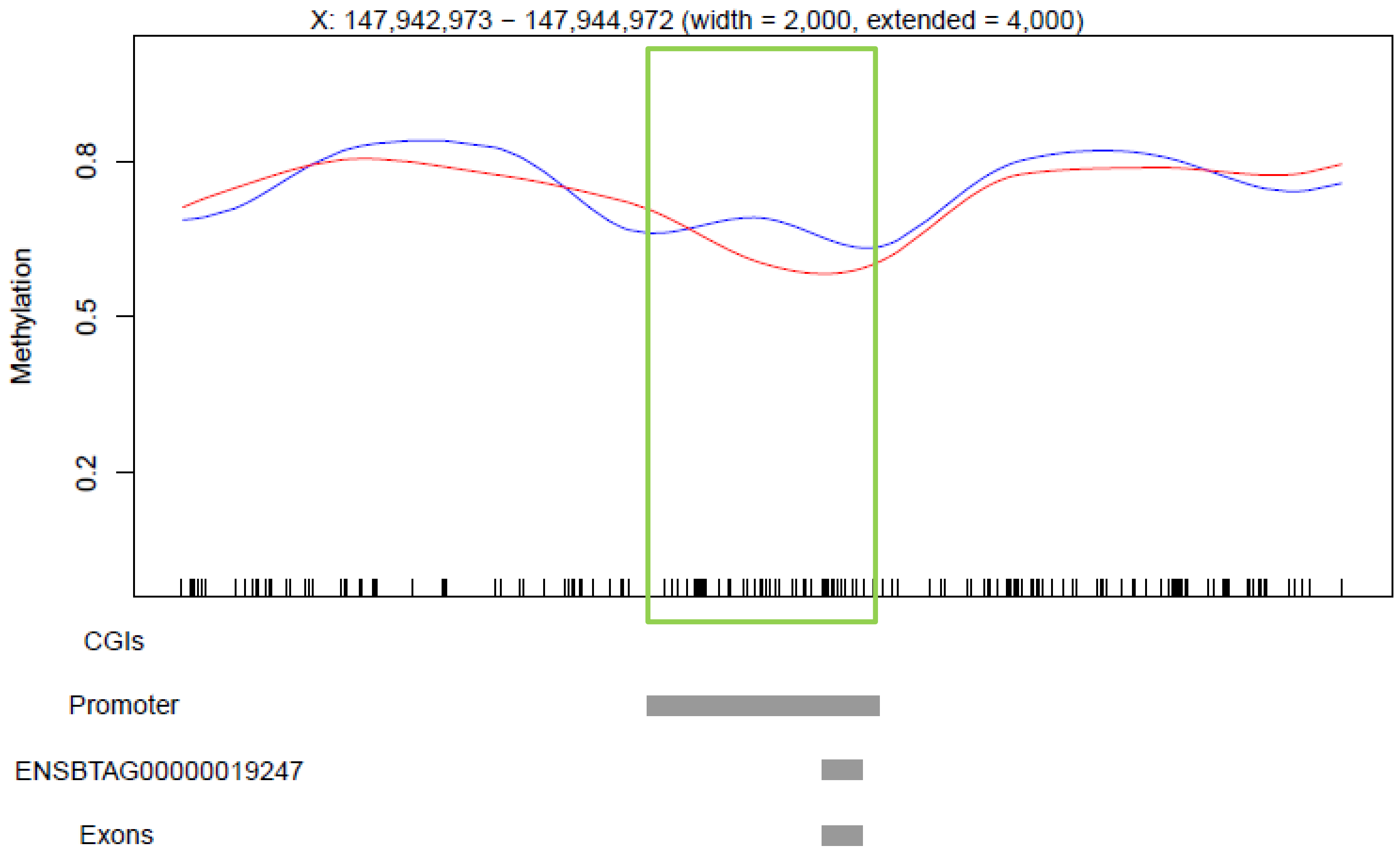

X: 147,942,973 − 147,944,972 (width = 2,000, extended = 4,000)
Methylation
0.8
0.5
0.2
CGIs
Promoter
ENSBTAG00000019247
Exons

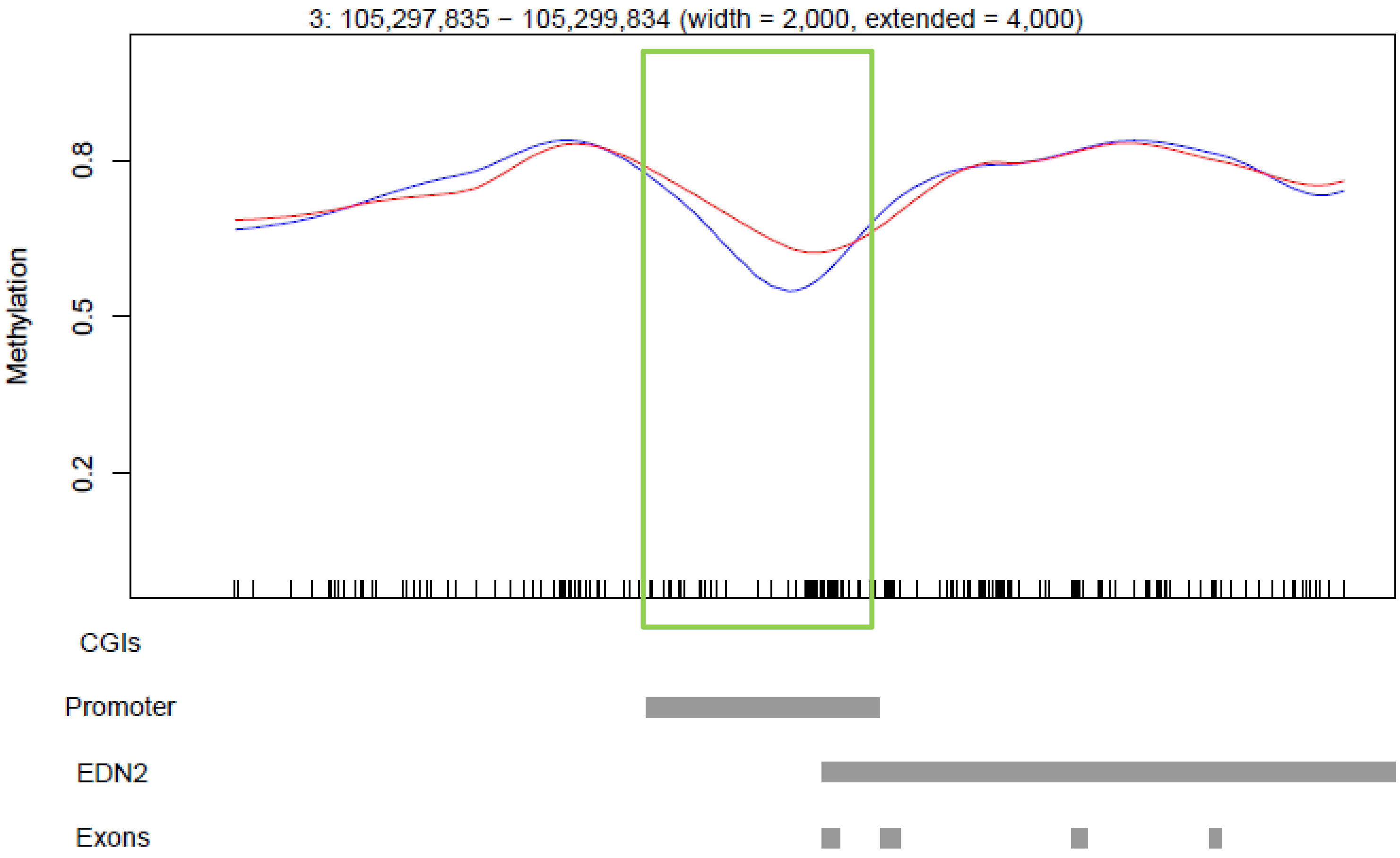
3: 105,297,835 − 105,299,834 (width = 2,000, extended = 4,000)
Methylation
0.2
0.5
0.8
CGIs
Promoter
EDN2
Exons

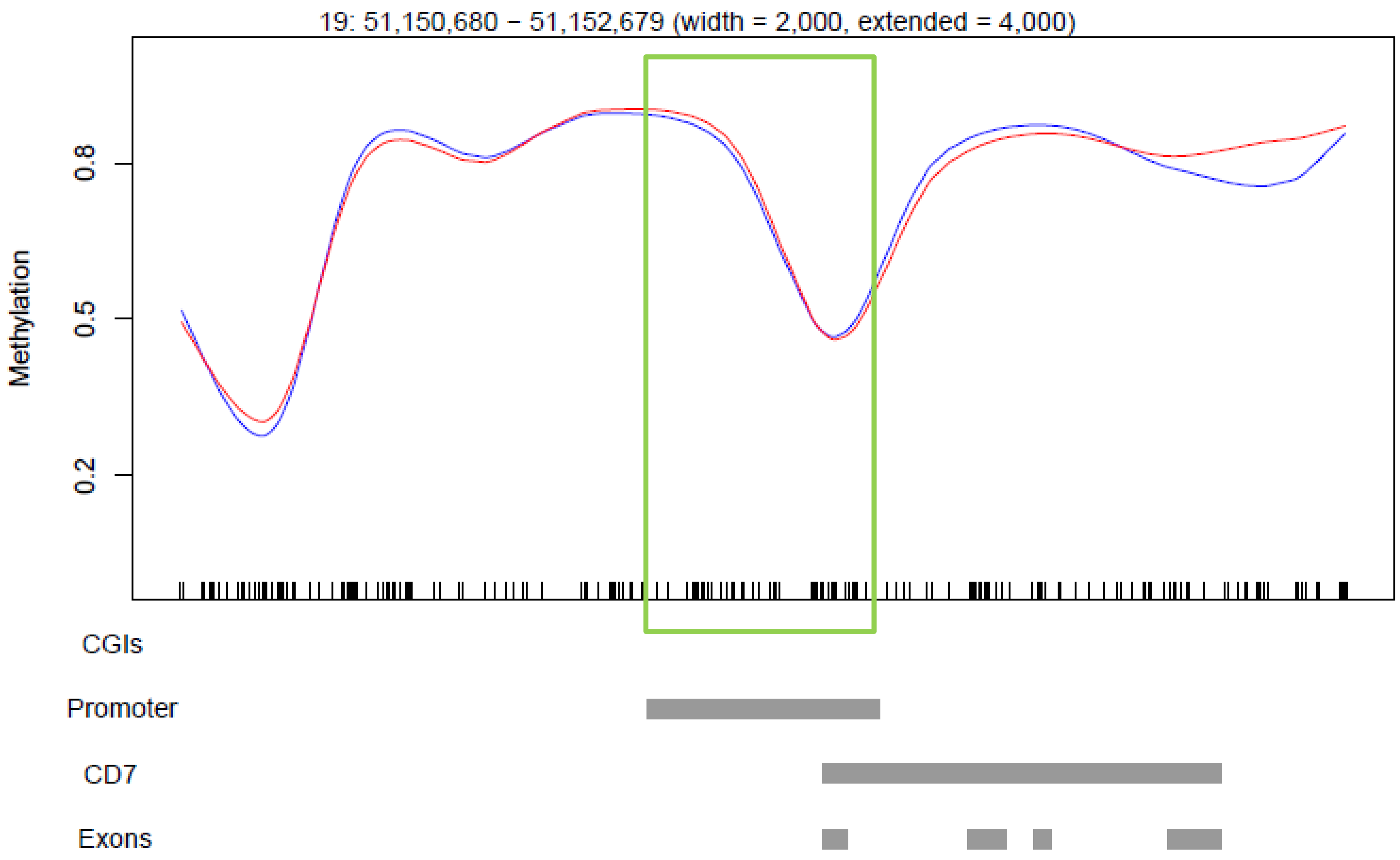

19: 51,150,680 – 51,152,679 (width = 2,000, extended = 4,000)
Methylation
0.2
0.5
0.8
CGIs
Promoter
CD7
Exons

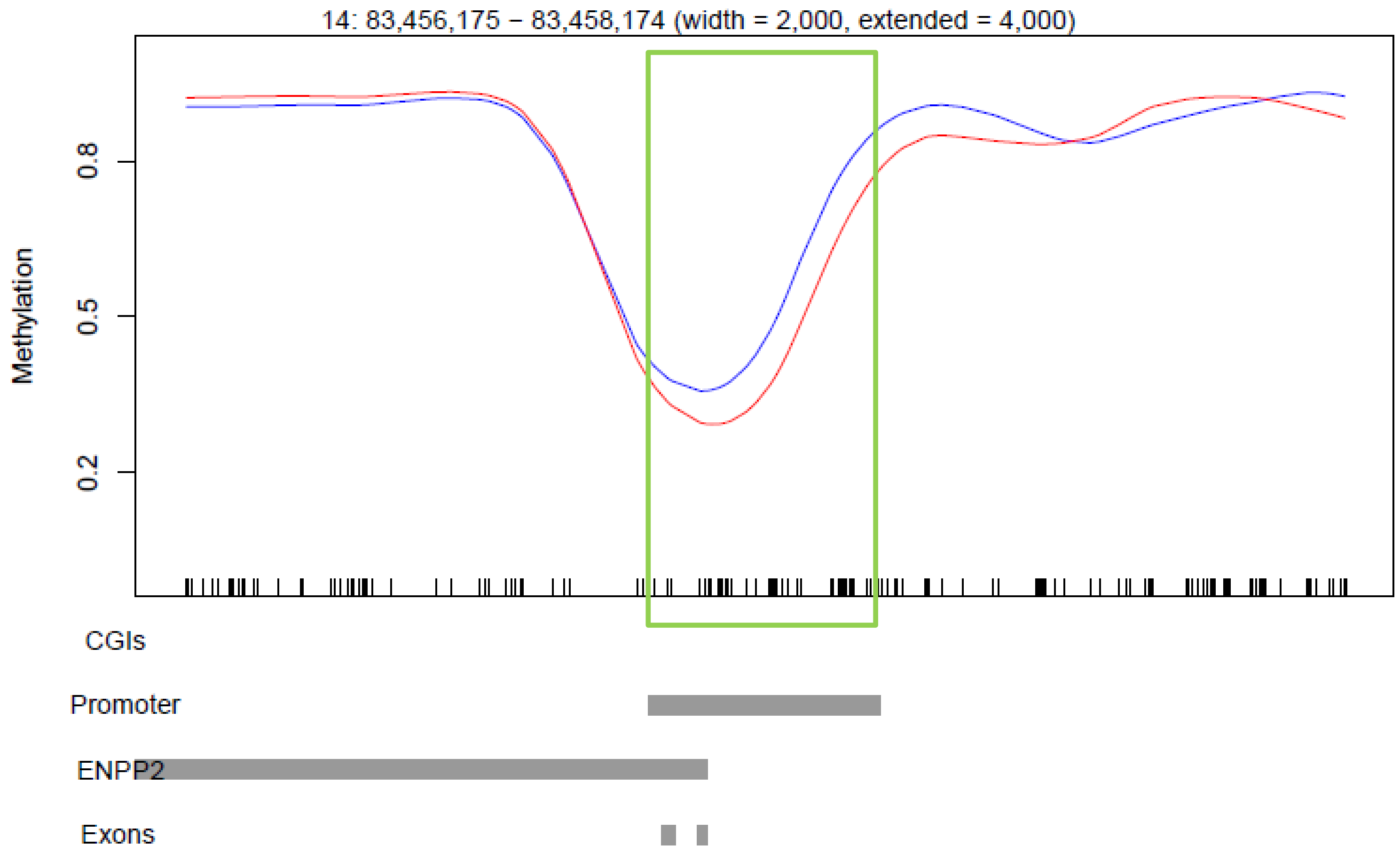

14: 83,456,175 − 83,458,174 (width = 2,000, extended = 4,000)
Methylation
0.8
0.5
0.2
CGIs
Promoter
ENPP2
Exons

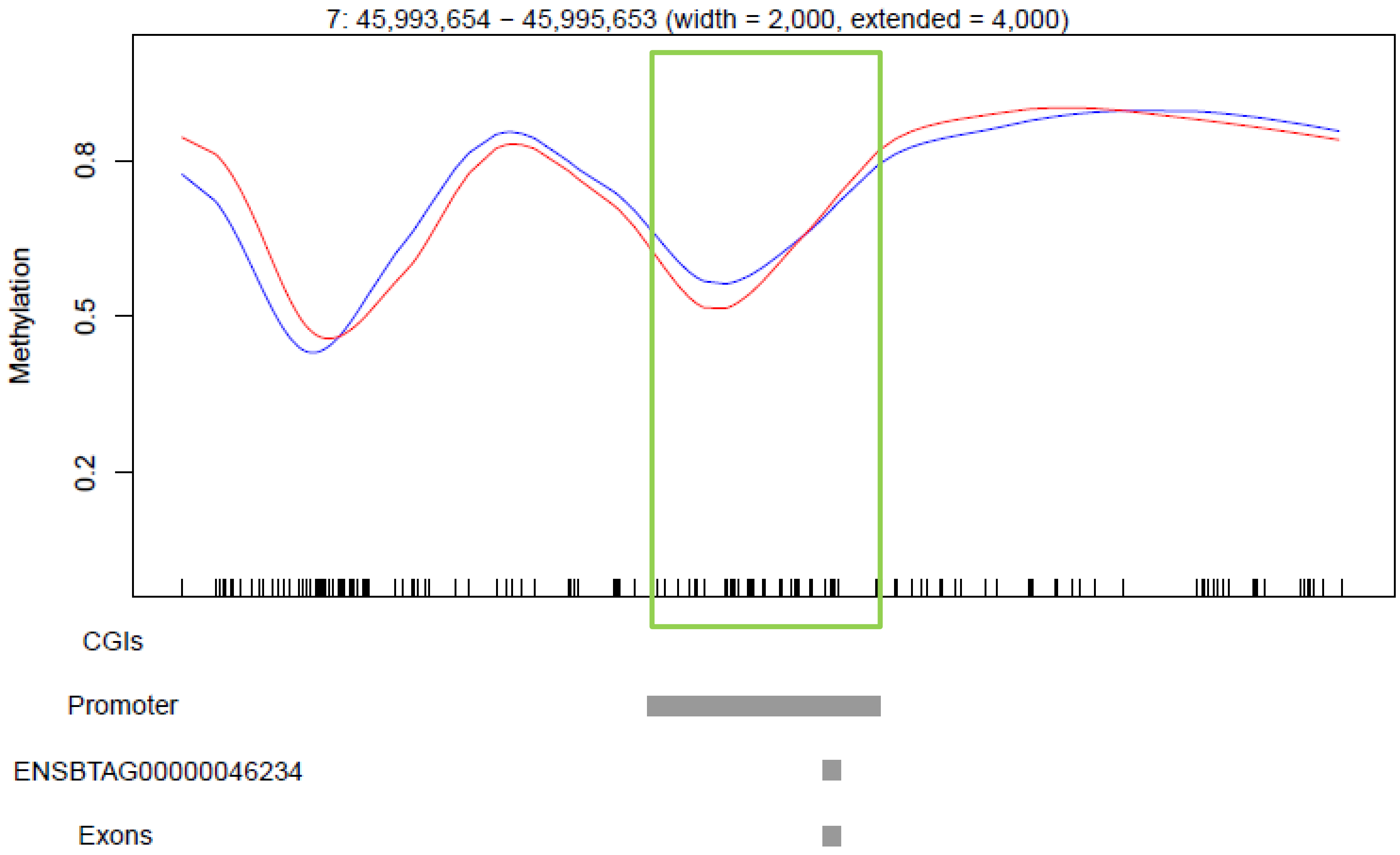

7: 45,993,654 − 45,995,653 (width = 2,000, extended = 4,000)
Methylation
0.2
0.5
0.8
CGIs
Promoter
ENSBTAG00000046234
Exons

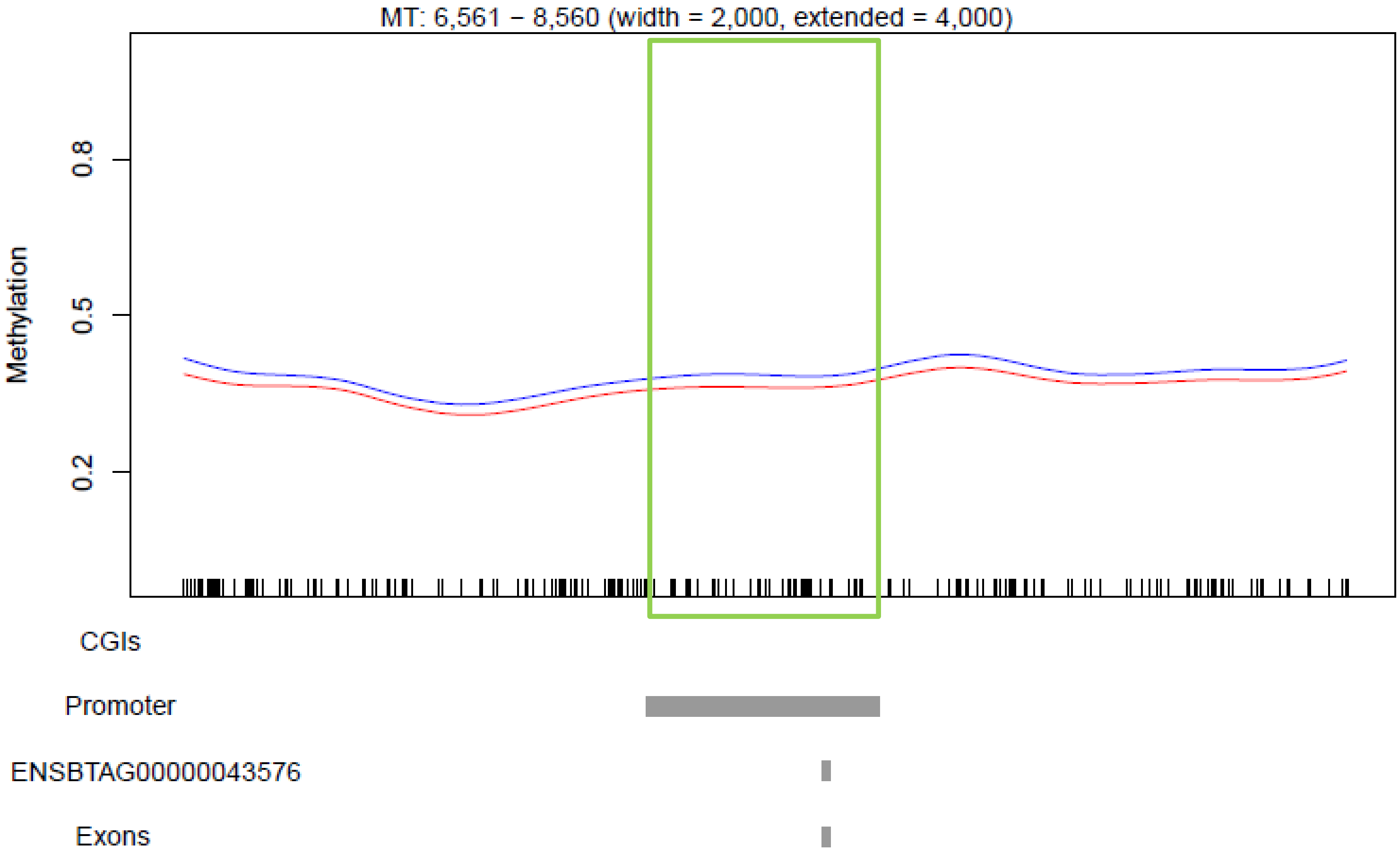
MT: 6,561 − 8,560 (width = 2,000, extended = 4,000)
Methylation
0.8
0.5
0.2
CGIs
Promoter
ENSBTAG00000043576
Exons

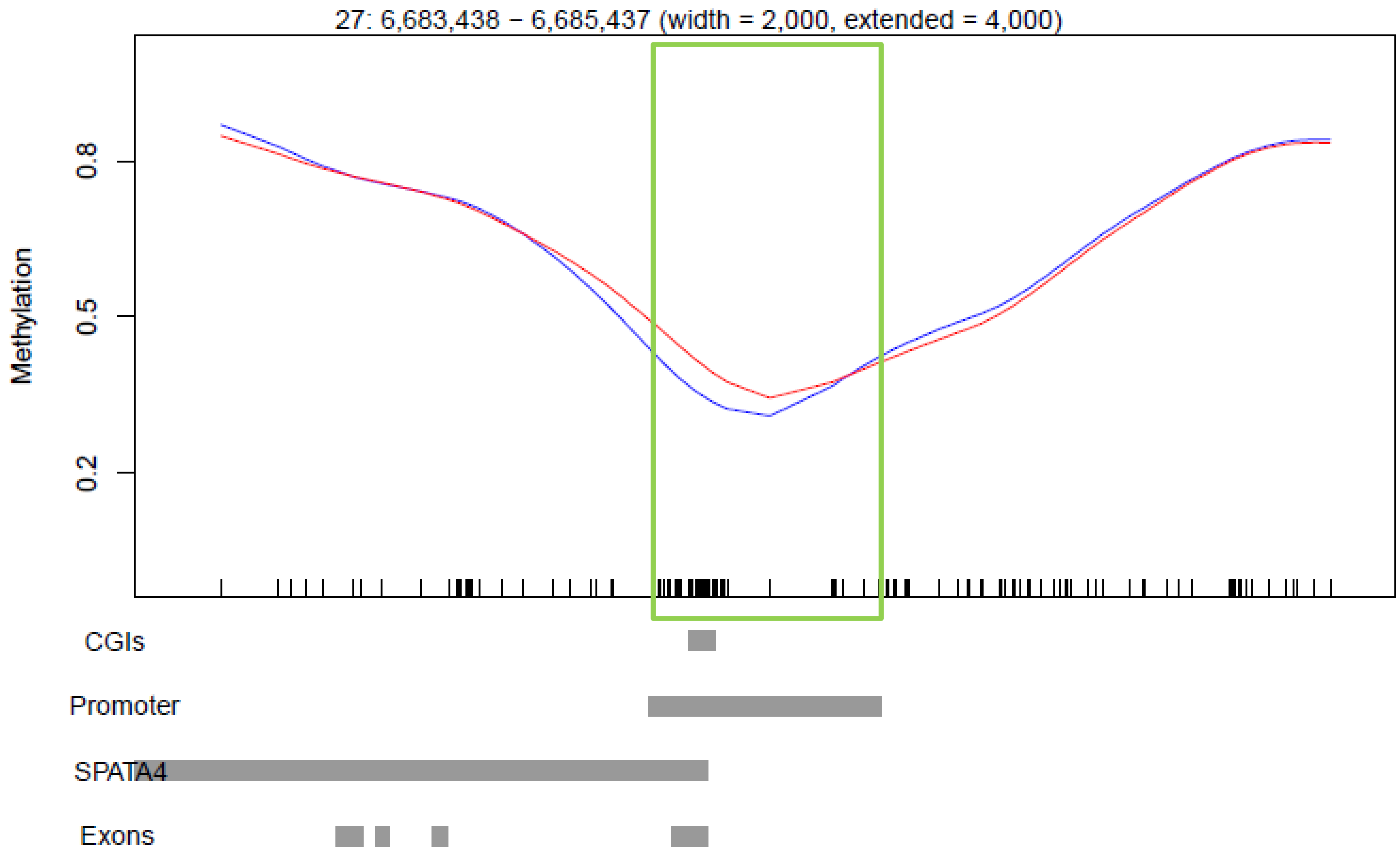

27: 6,683,438 − 6,685,437 (width = 2,000, extended = 4,000)
Methylation
0.8
0.5
0.2
CGIs
Promoter
SPATA4
Exons

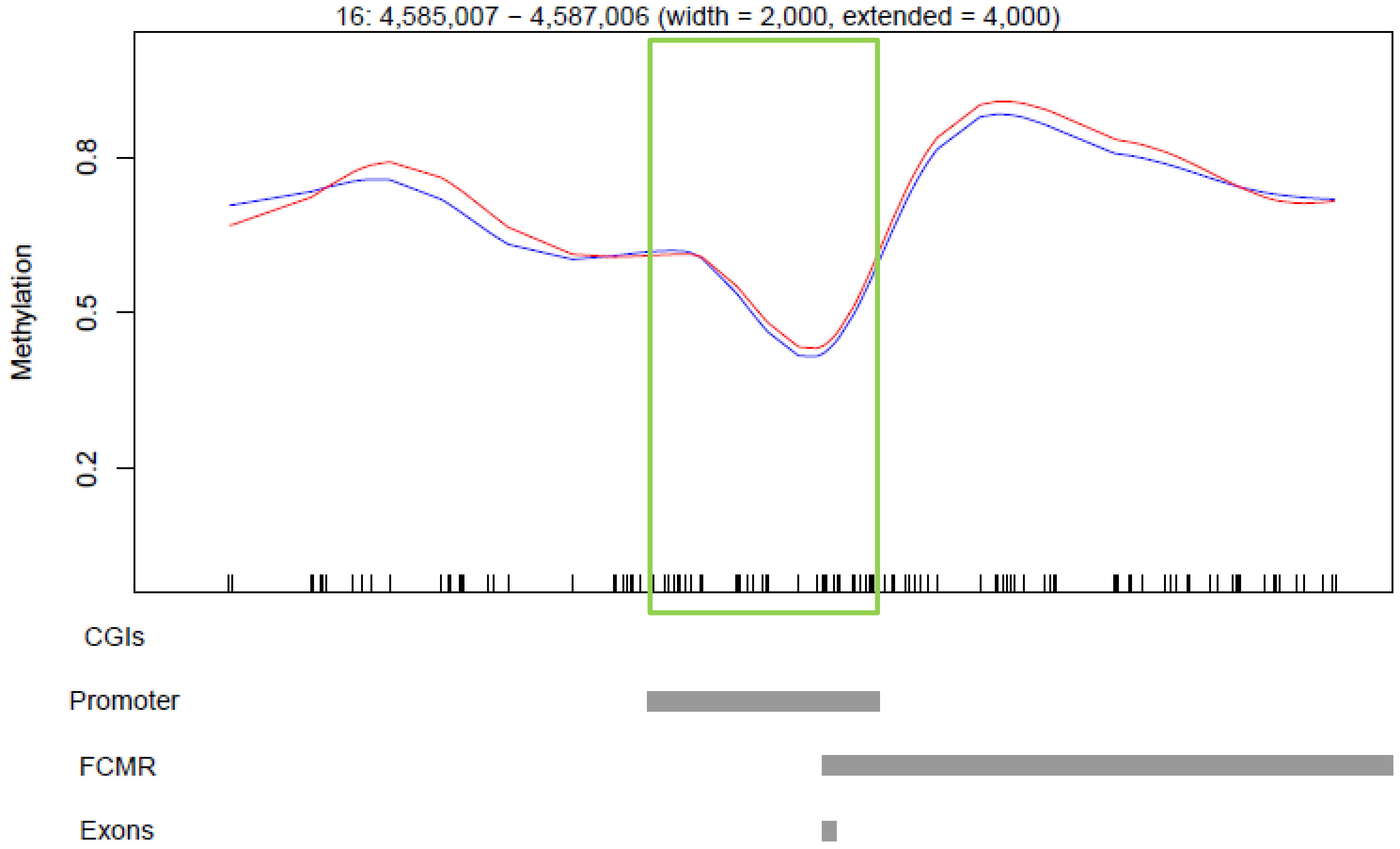
16: 4,585,007 − 4,587,006 (width = 2,000, extended = 4,000)
Methylation
0.8
0.5
0.2
CGIs
Promoter
FCMR
Exons

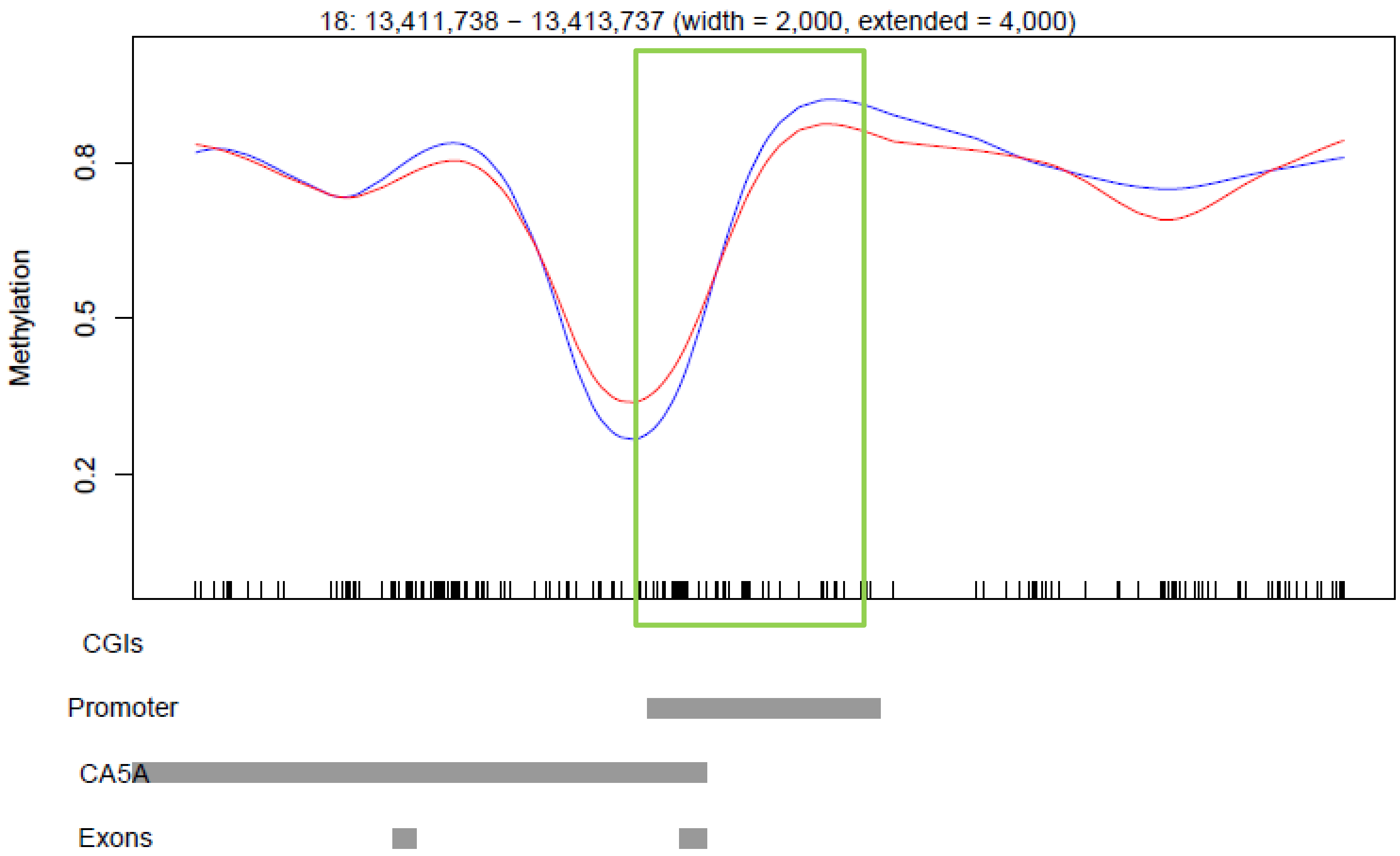

18: 13,411,738 − 13,413,737 (width = 2,000, extended = 4,000)
Methylation
0.8
0.5
0.2
CGIs
Promoter
CA5A
Exons

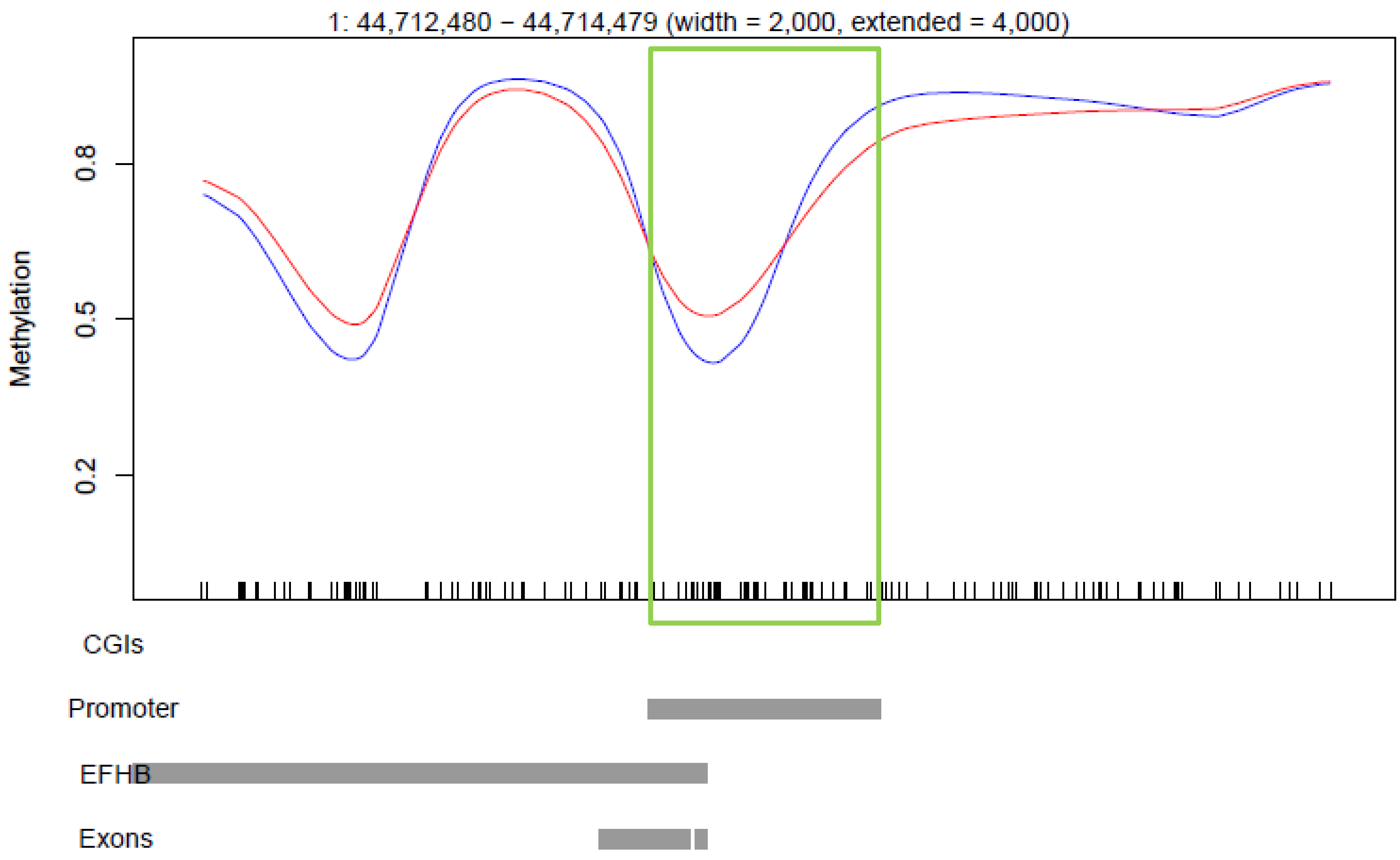

1: 44,712,480 − 44,714,479 (width = 2,000, extended = 4,000)
Methylation
0.2
0.5
0.8
CGIs
Promoter
EFHB
Exons

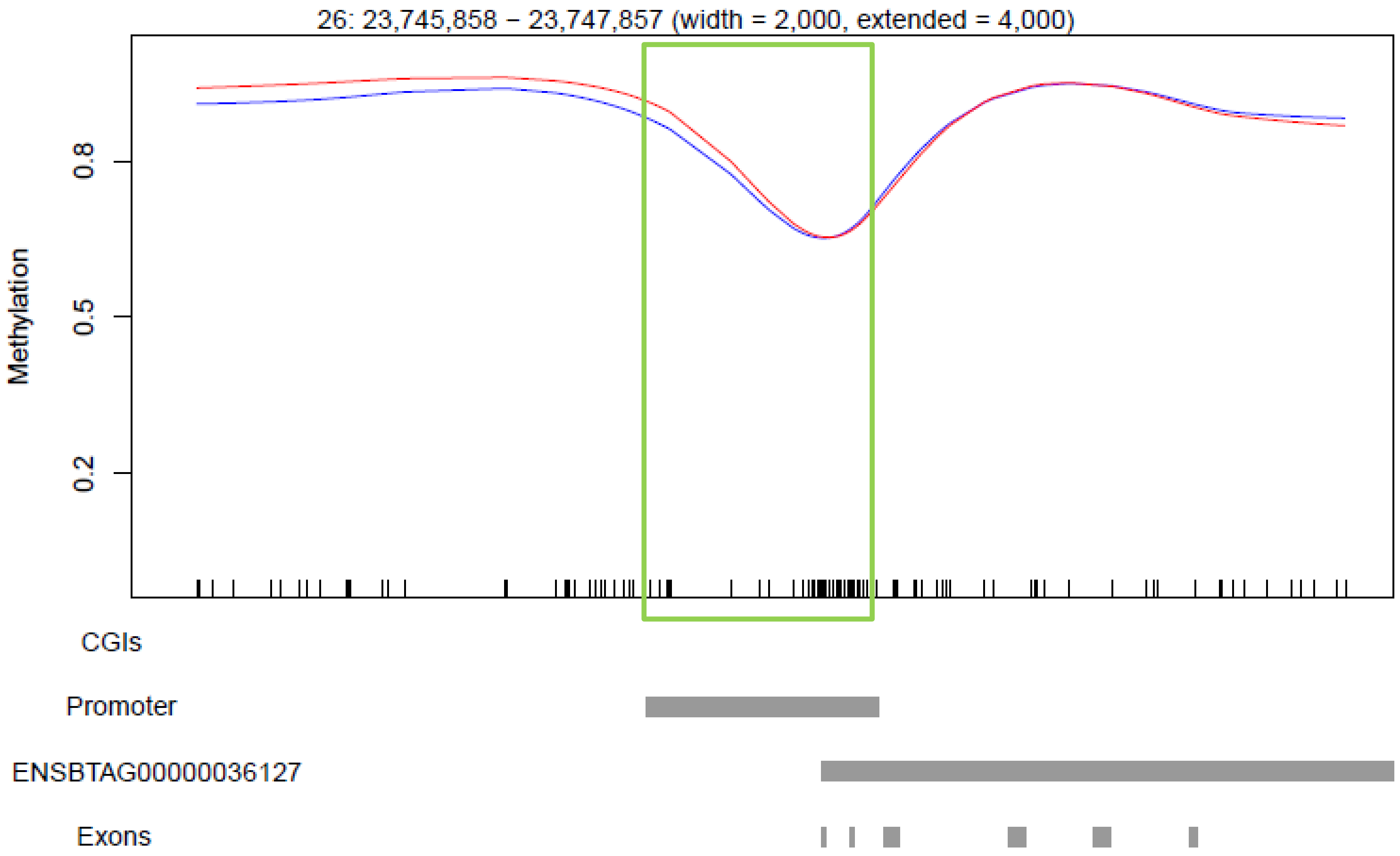

26: 23,745,858 – 23,747,857 (width = 2,000, extended = 4,000)
Methylation
0.8
0.5
0.2
CGIs
Promoter
ENSBTAG00000036127
Exons

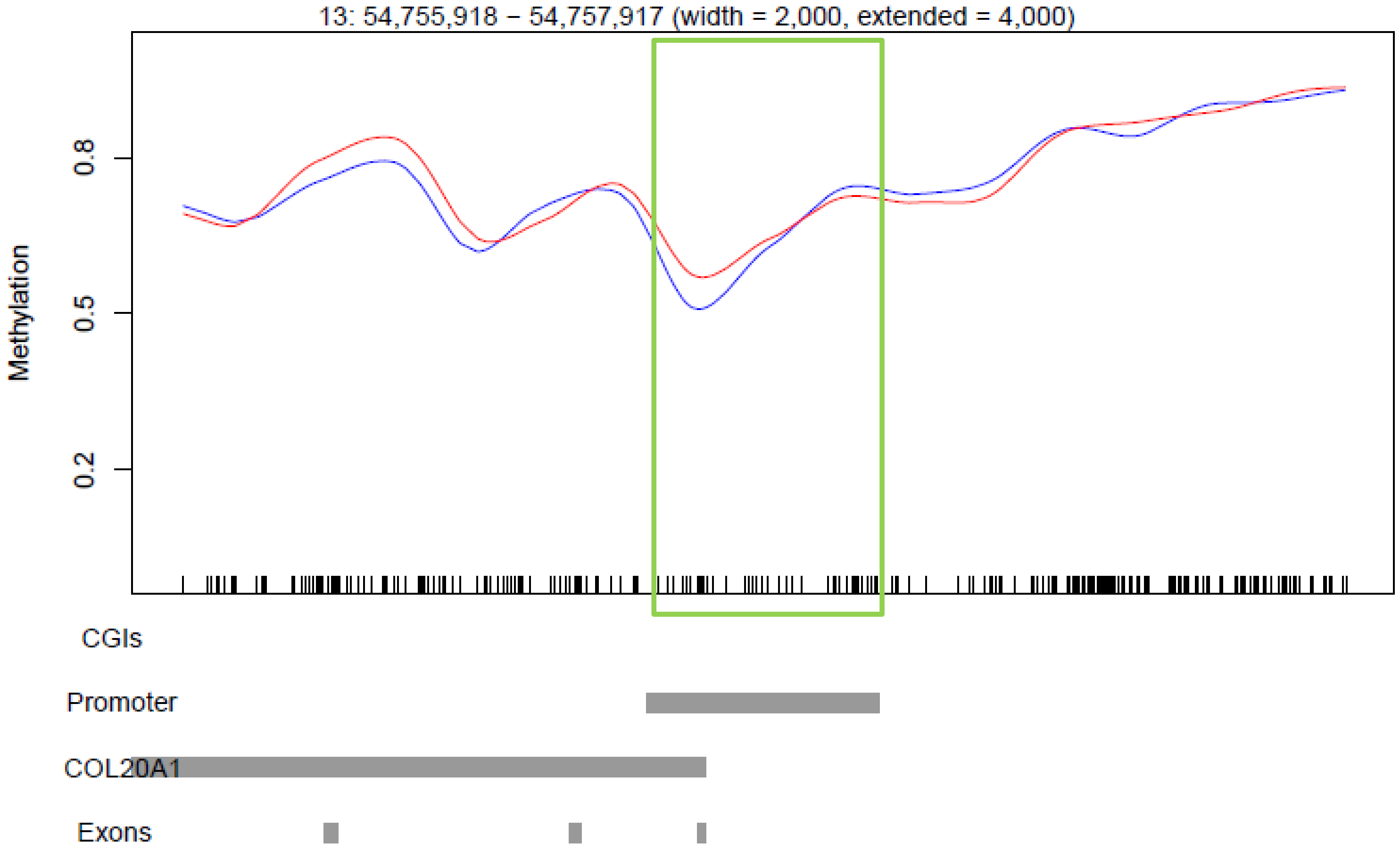
13: 54,755,918 − 54,757,917 (width = 2,000, extended = 4,000)
Methylation
0.8
0.5
0.2
CGIs
Promoter
COL20A1
Exons

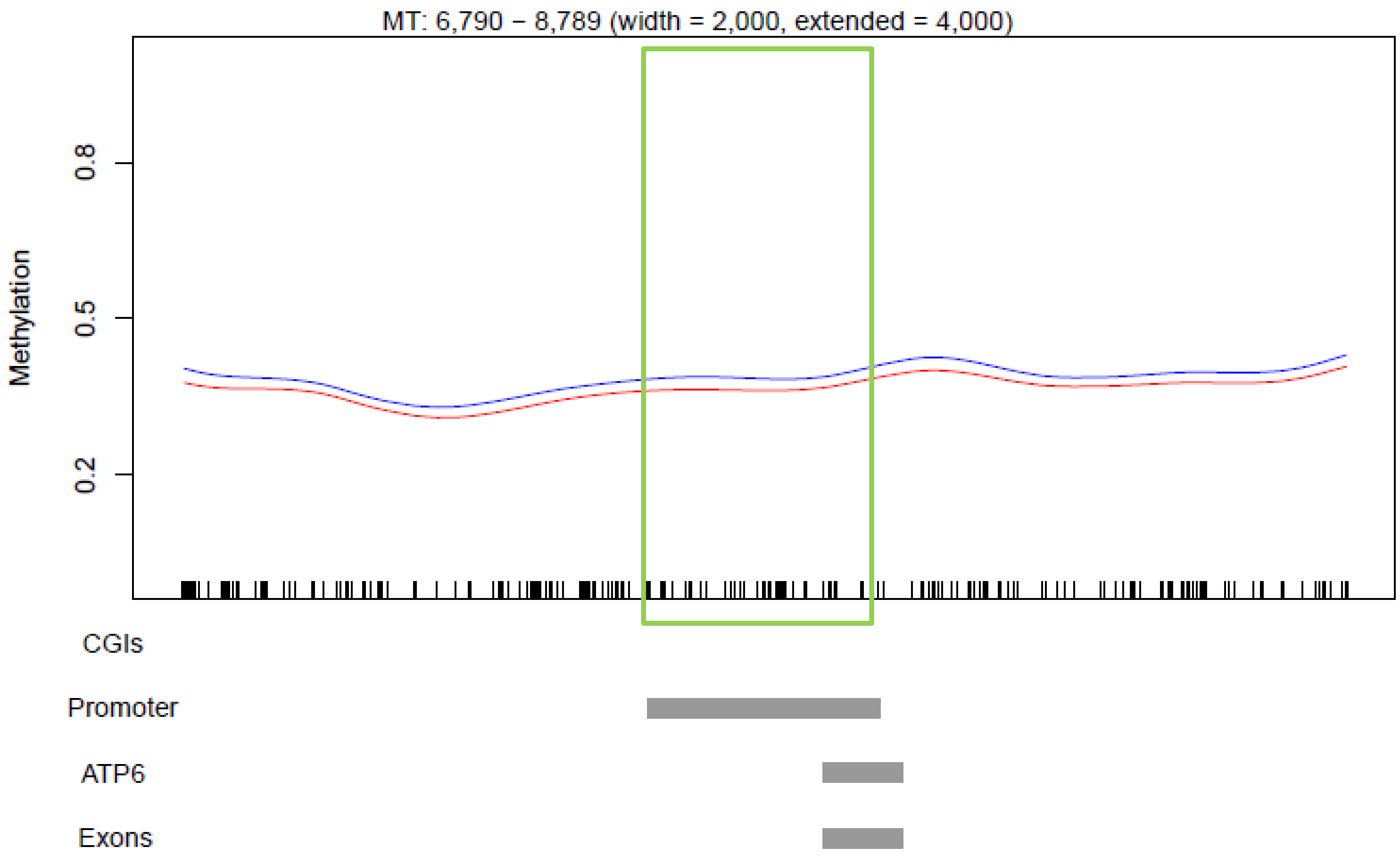
MT: 6,790 − 8,789 (width = 2,000, extended = 4,000)
Methylation
0.8
0.5
0.2
CGIs
Promoter
ATP6
Exons

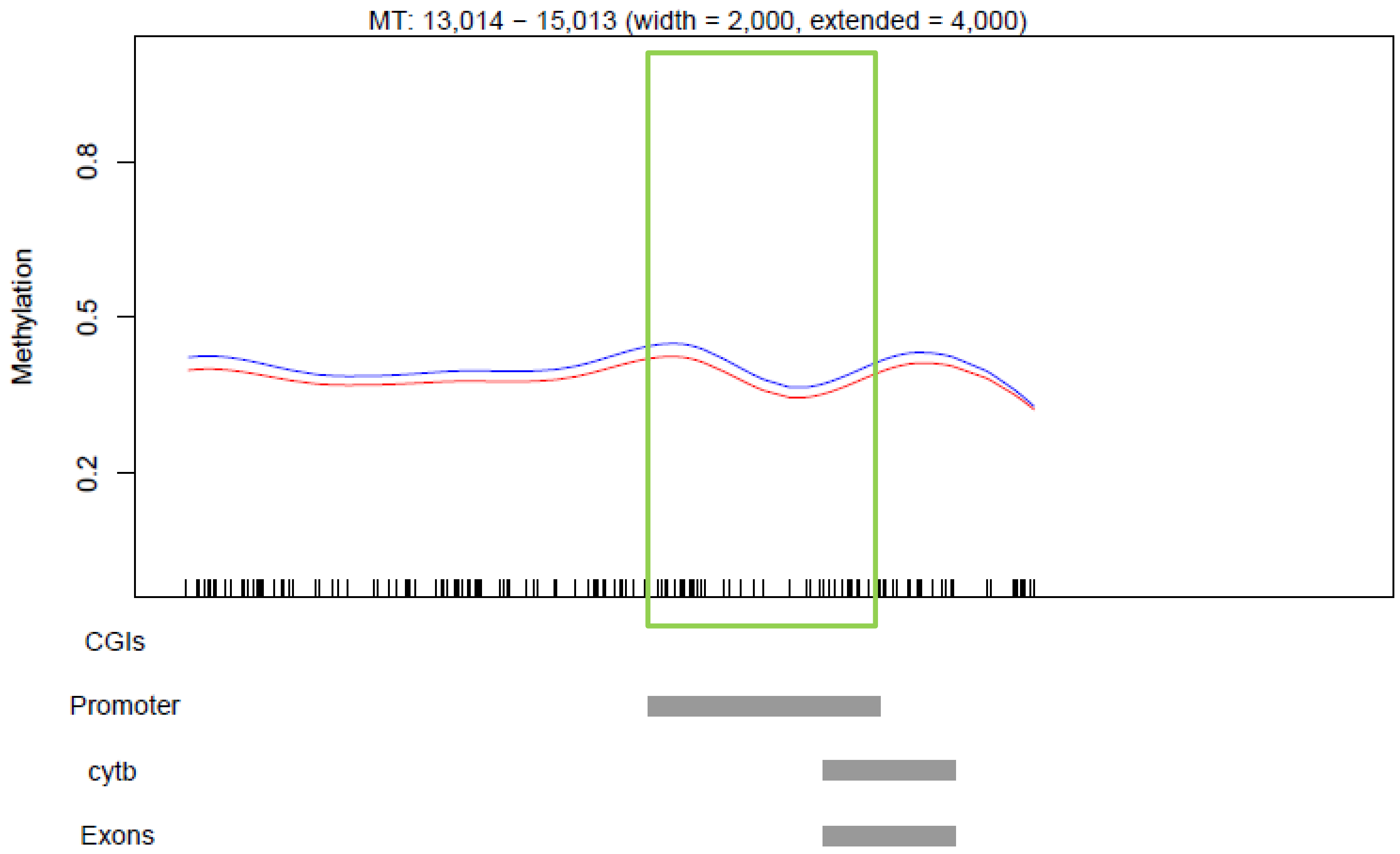

MT: 13,014 − 15,013 (width = 2,000, extended = 4,000)
Methylation
0.2
0.5
0.8
CGIs
Promoter
cytb
Exons

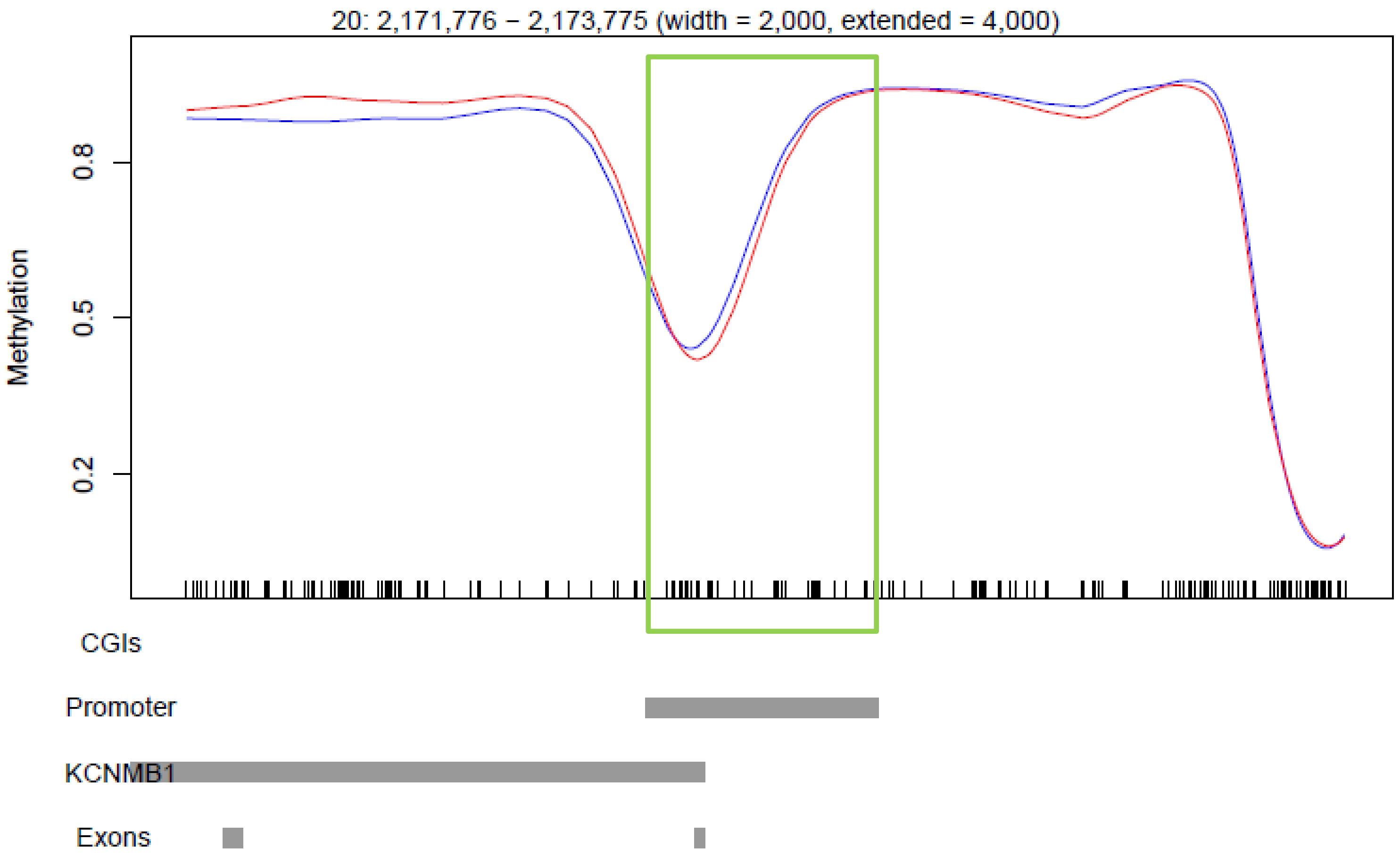

20: 2,171,776 − 2,173,775 (width = 2,000, extended = 4,000)
Methylation
0.2
0.5
0.8
CGIs
Promoter
KCNMB1
Exons

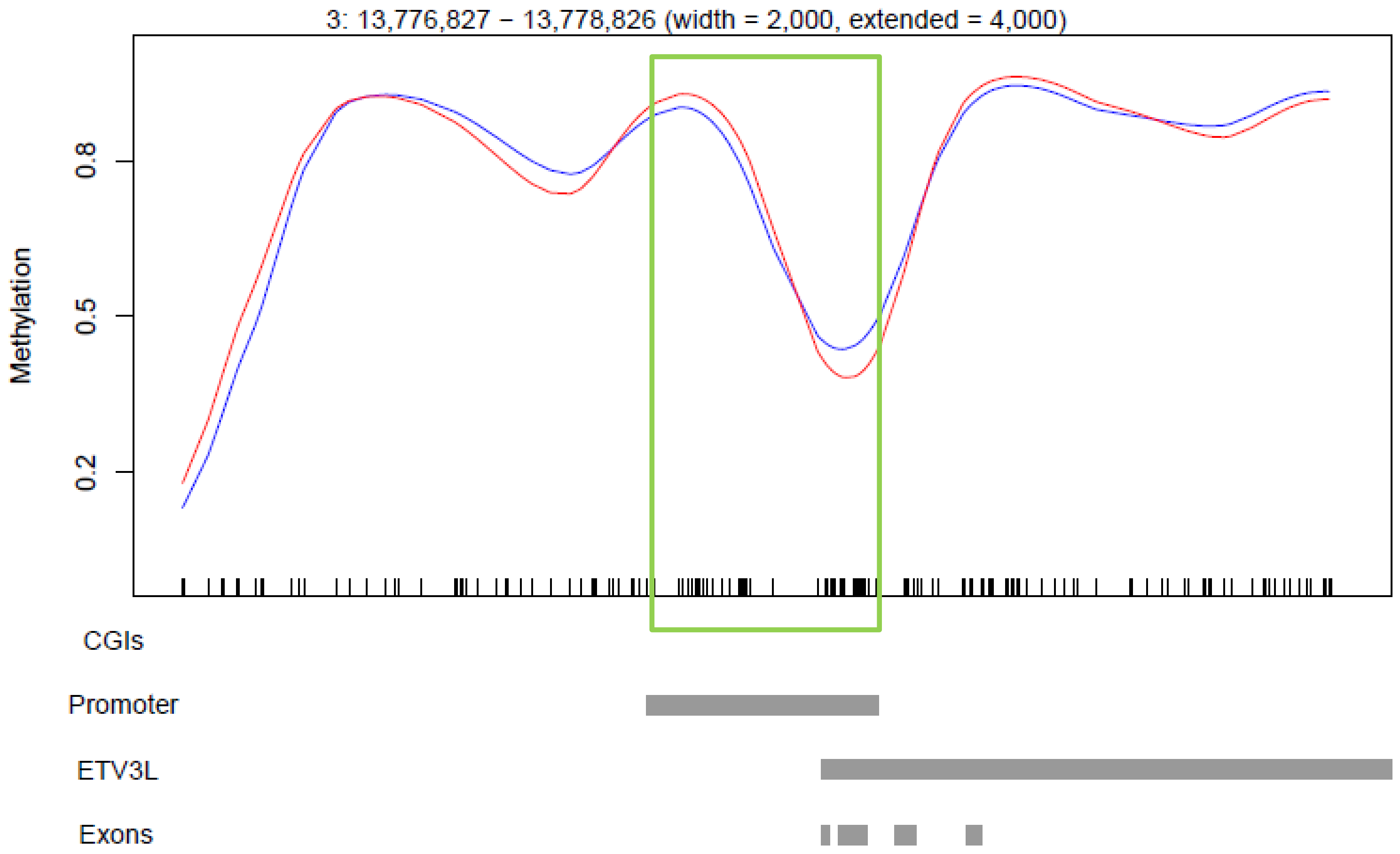

3: 13,776,827 − 13,778,826 (width = 2,000, extended = 4,000)
Methylation
0.2
0.5
0.8
CGIs
Promoter
ETV3L
Exons

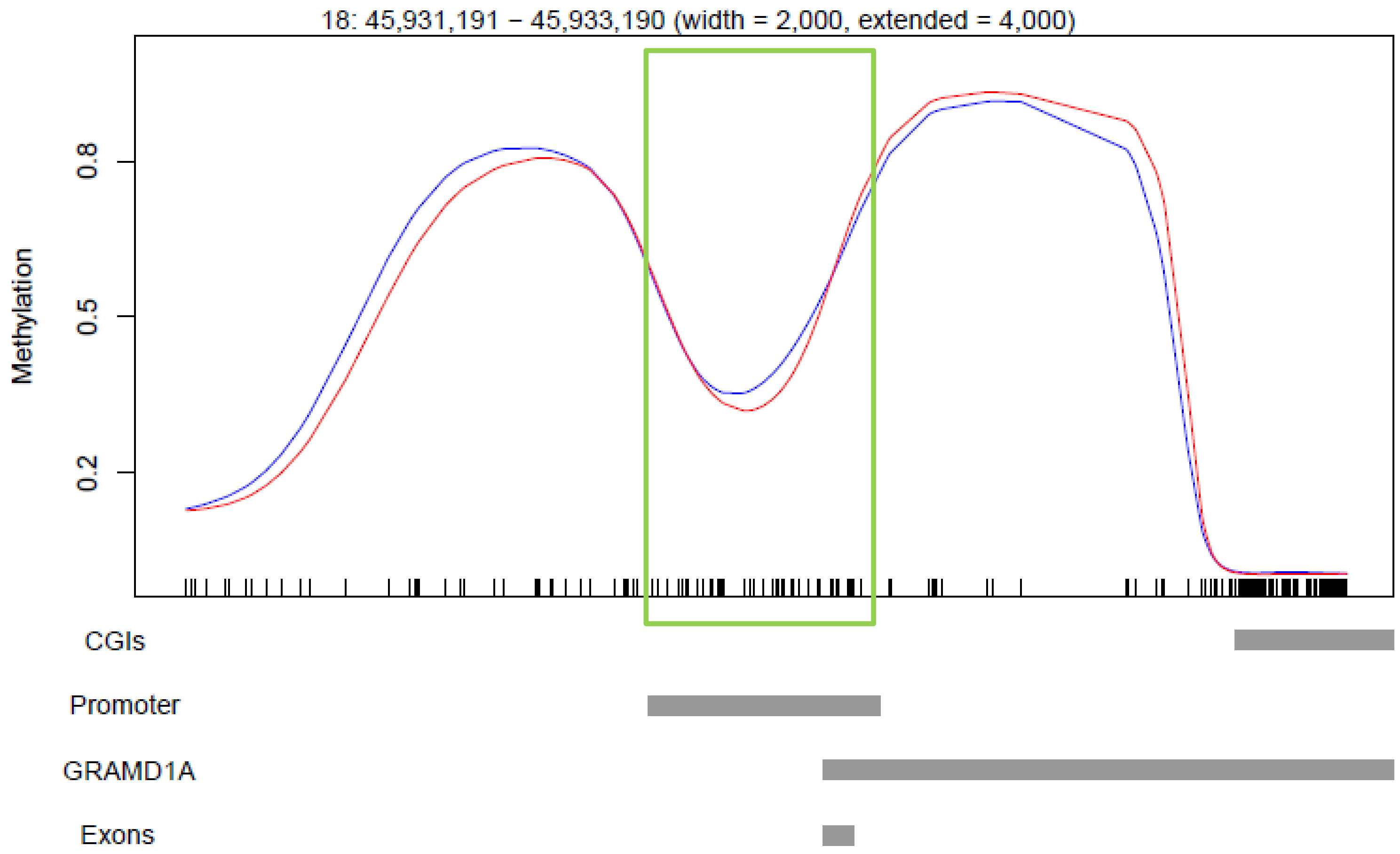

18: 45,931,191 − 45,933,190 (width = 2,000, extended = 4,000)
Methylation
0.8
0.5
0.2
CGIs
Promoter
GRAMD1A
Exons

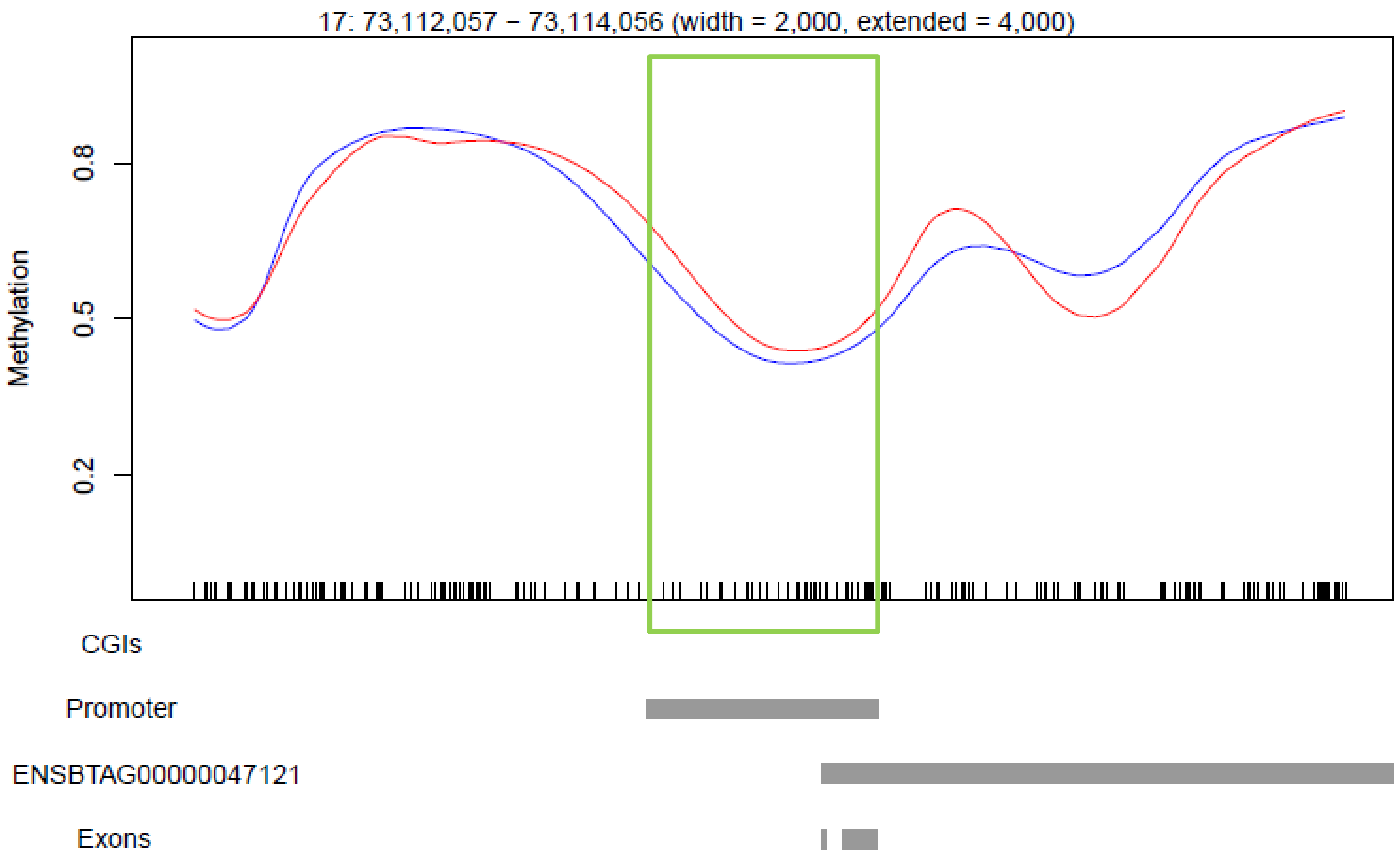
17: 73,112,057 − 73,114,056 (width = 2,000, extended = 4,000)
Methylation
0.8
0.5
0.2
CGIs
Promoter
ENSBTAG00000047121
Exons

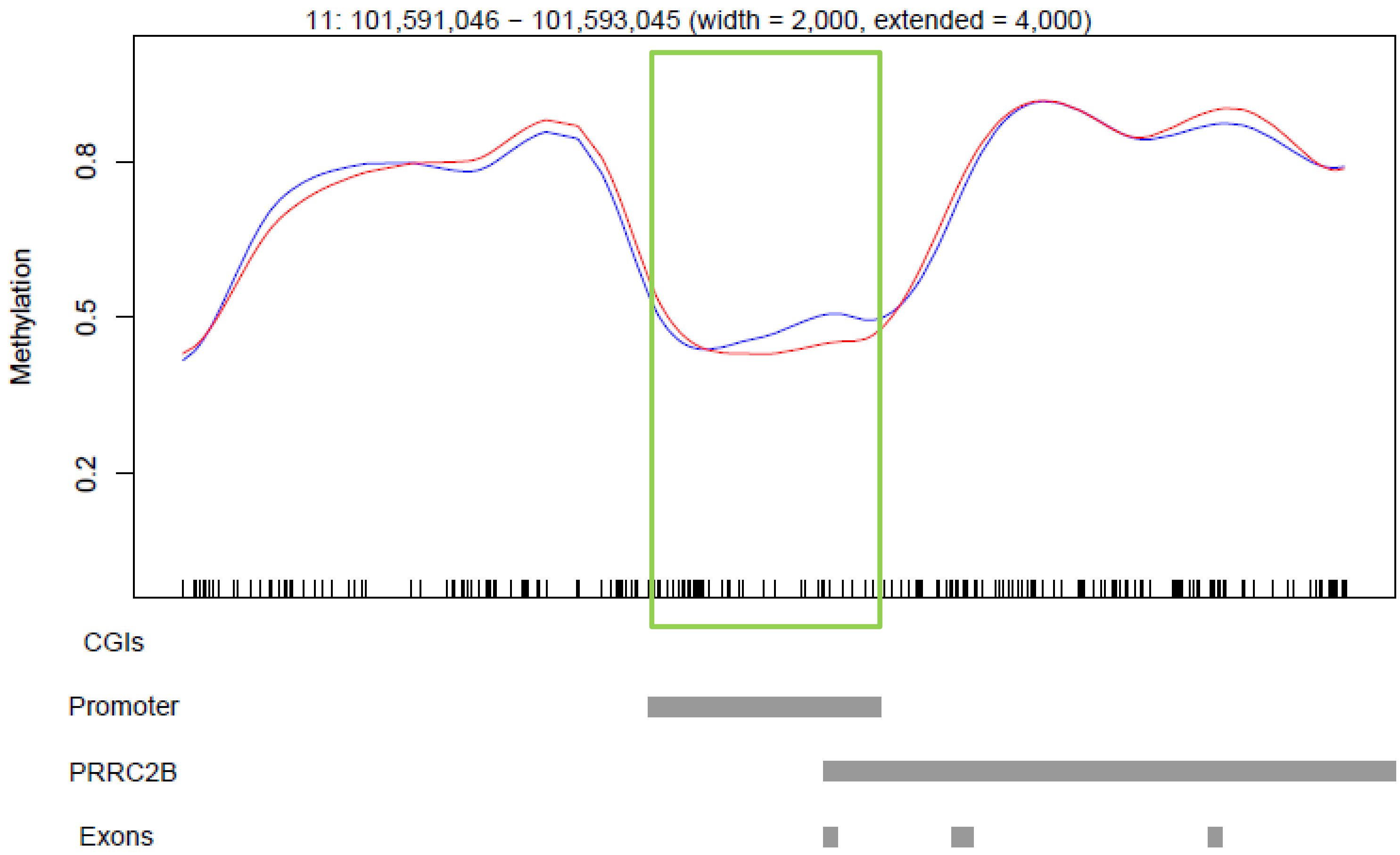
11: 101,591,046 – 101,593,045 (width = 2,000, extended = 4,000)
Methylation
0.8
0.5
0.2
CGIs
Promoter
PRRC2B
Exons

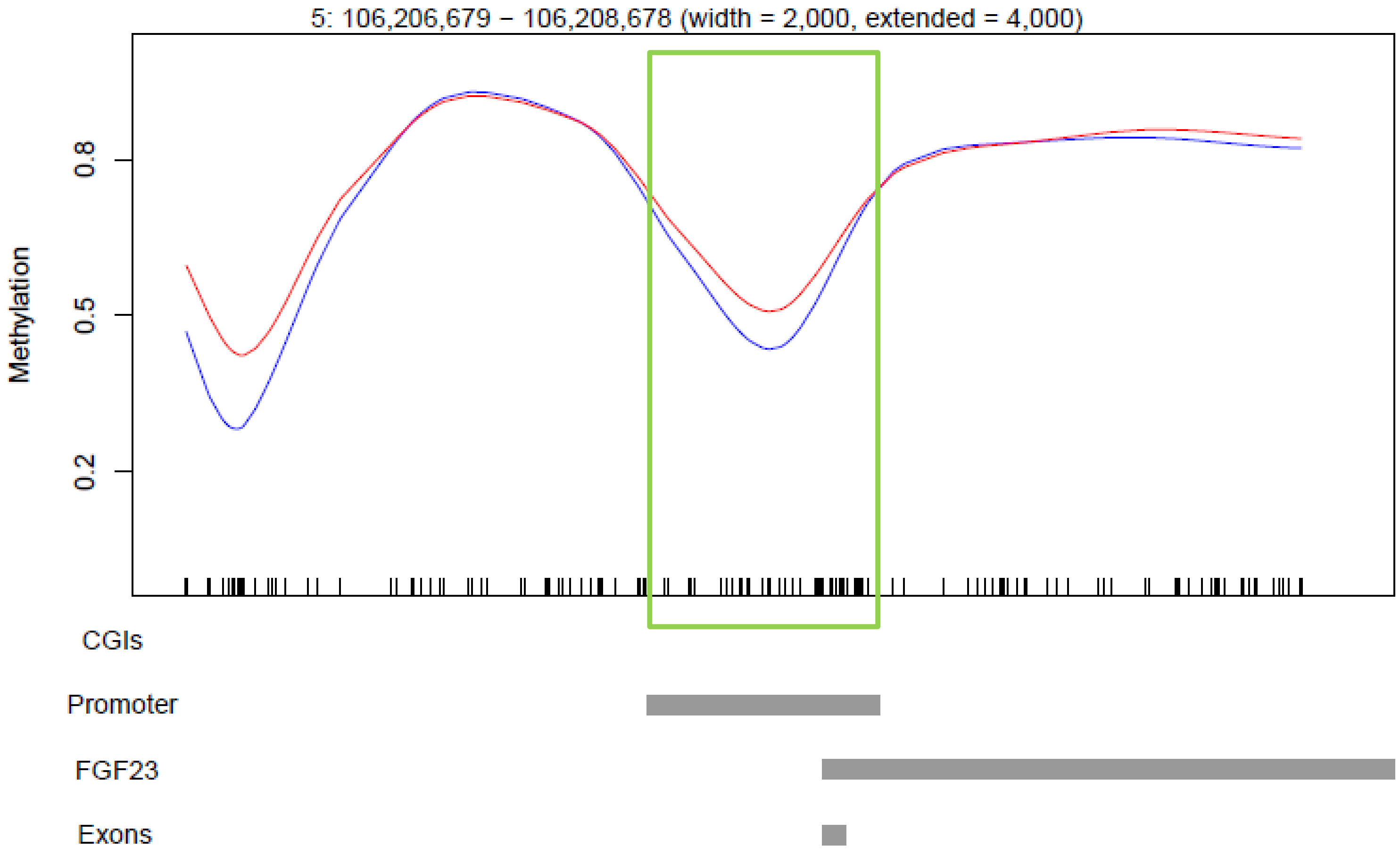
5: 106,206,679 – 106,208,678 (width = 2,000, extended = 4,000)
Methylation
0.8
0.5
0.2
CGIs
Promoter
FGF23
Exons

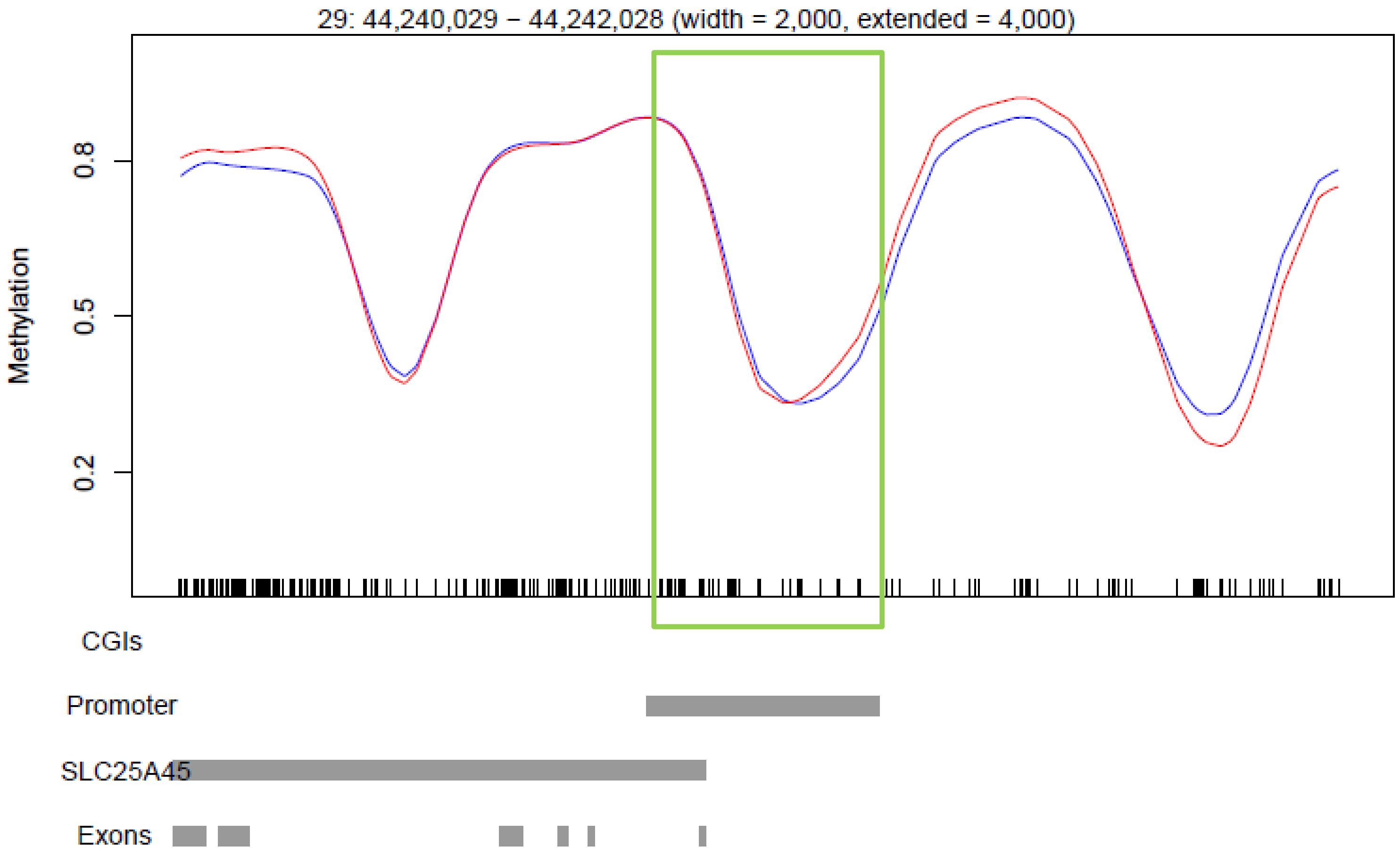

29: 44,240,029 − 44,242,028 (width = 2,000, extended = 4,000)
Methylation
0.2
0.5
0.8
CGIs
Promoter
SLC25A45
Exons

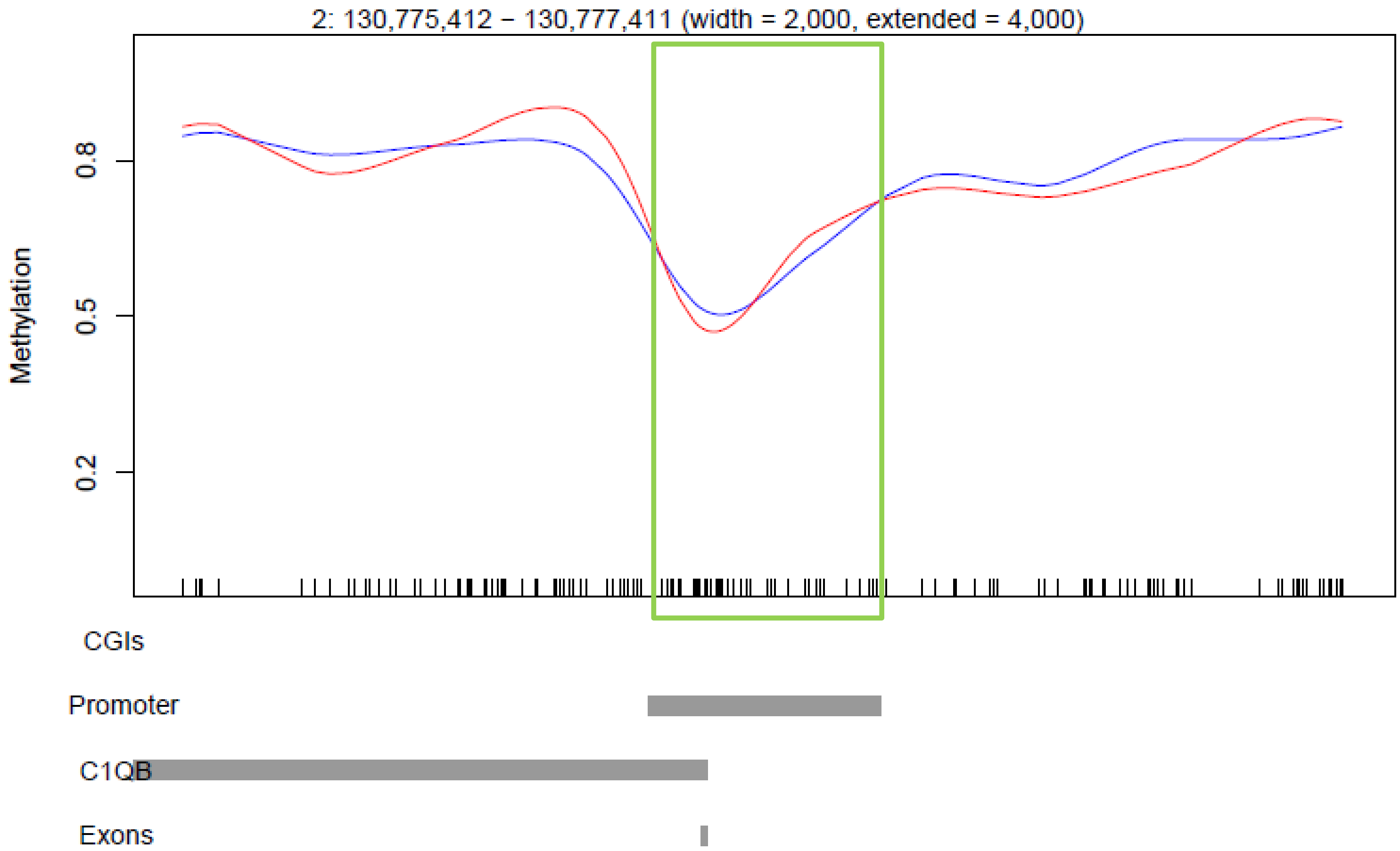

2: 130,775,412 − 130,777,411 (width = 2,000, extended = 4,000)
Methylation
0.8
0.5
0.2
CGIs
Promoter
C1QB
Exons

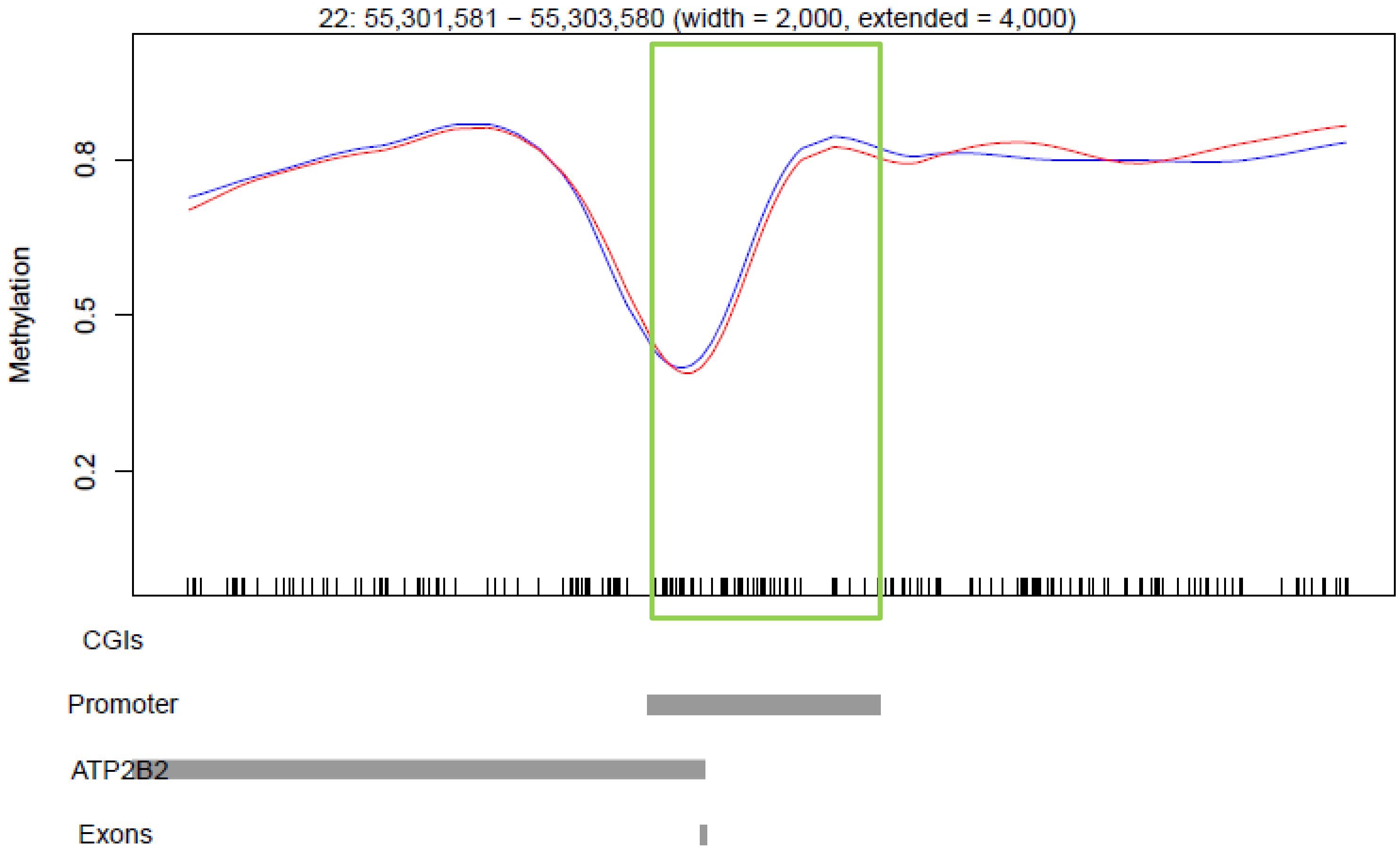

22: 55,301,581 − 55,303,580 (width = 2,000, extended = 4,000)
Methylation
0.8
0.5
0.2
CGIs
Promoter
ATP2B2
Exons

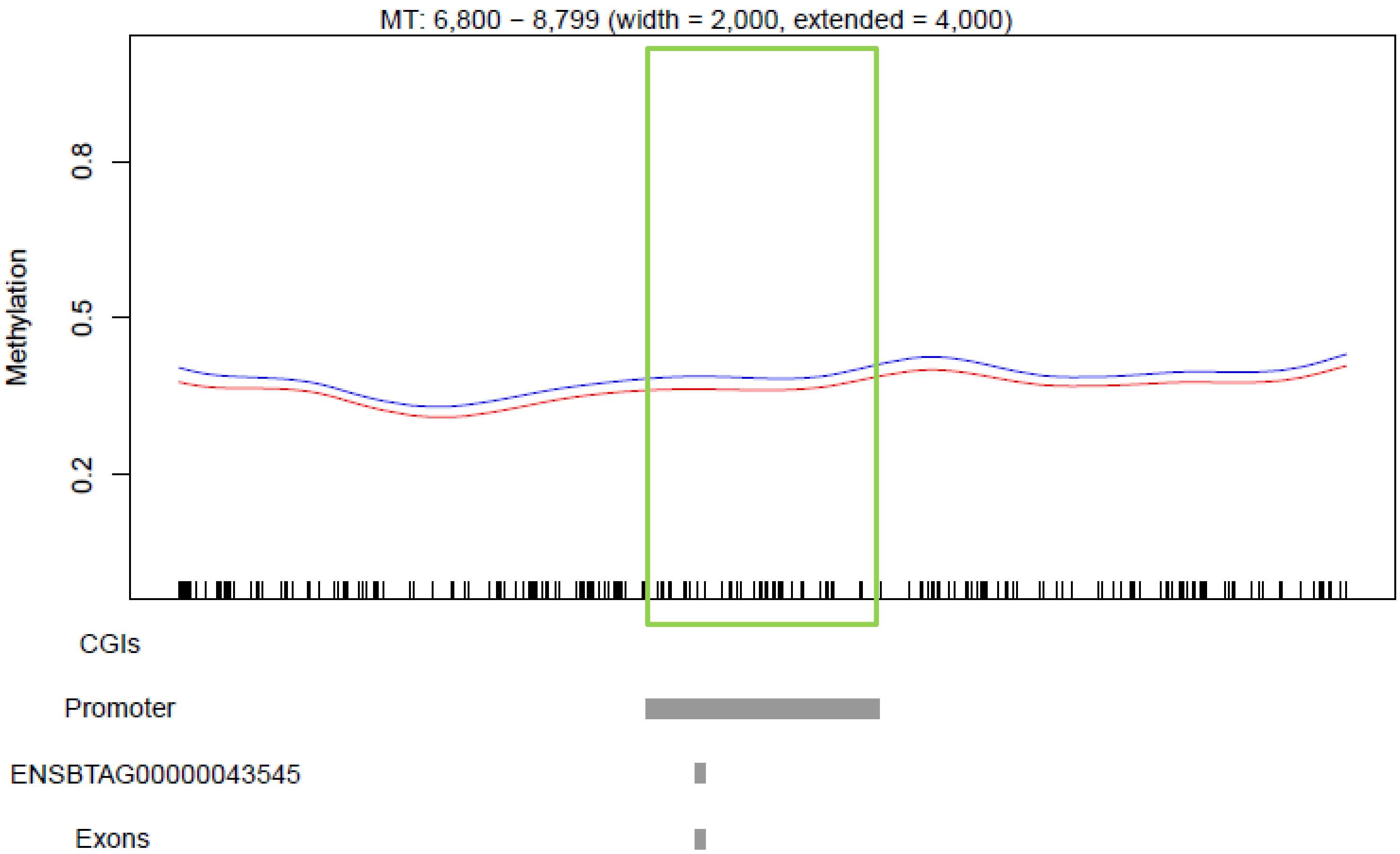

MT: 6,800 − 8,799 (width = 2,000, extended = 4,000)
Methylation
0.8
0.5
0.2
CGIs
Promoter
ENSBTAG00000043545
Exons

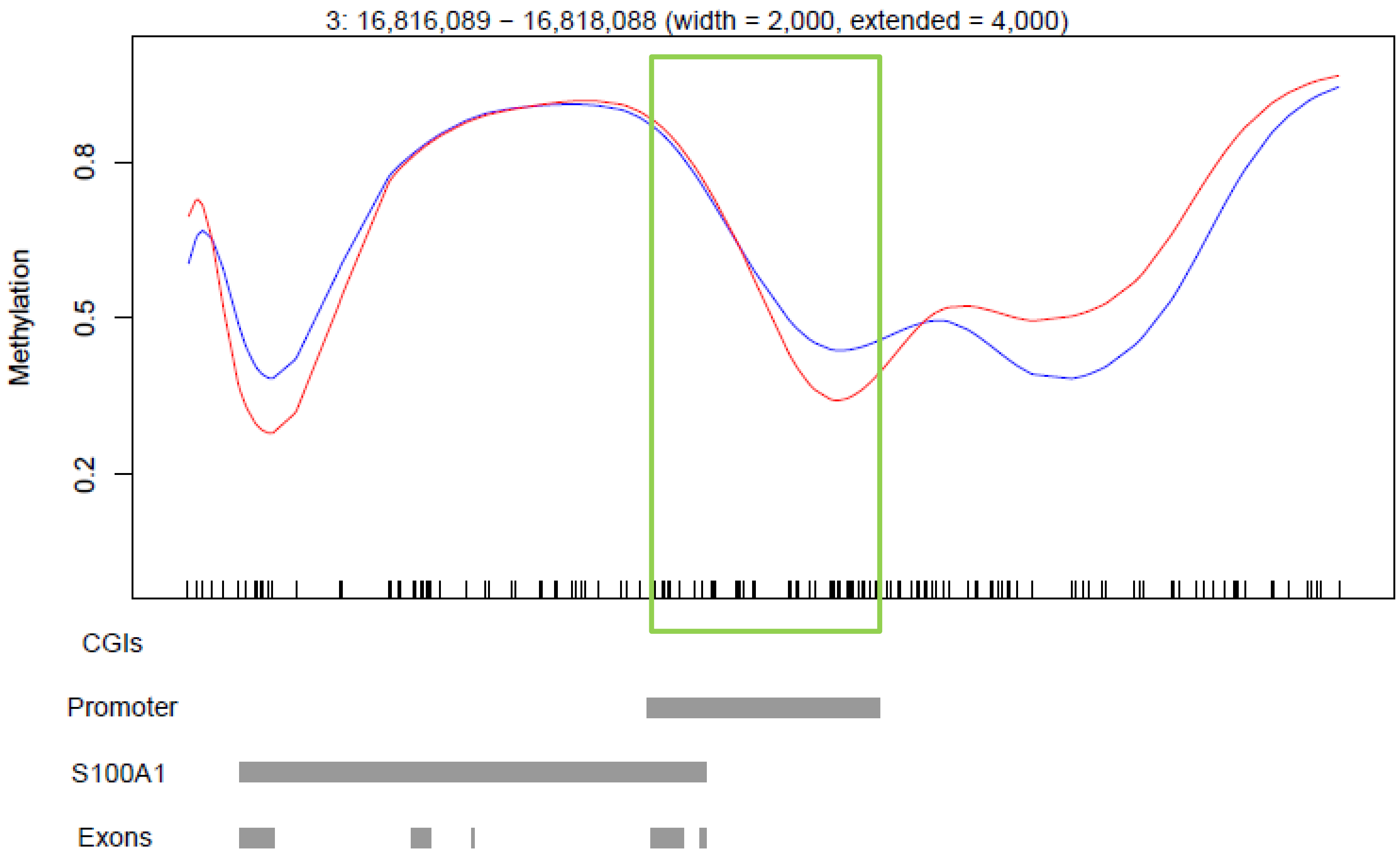

3: 16,816,089 – 16,818,088 (width = 2,000, extended = 4,000)
Methylation
0.2
0.5
0.8
CGIs
Promoter
S100A1
Exons

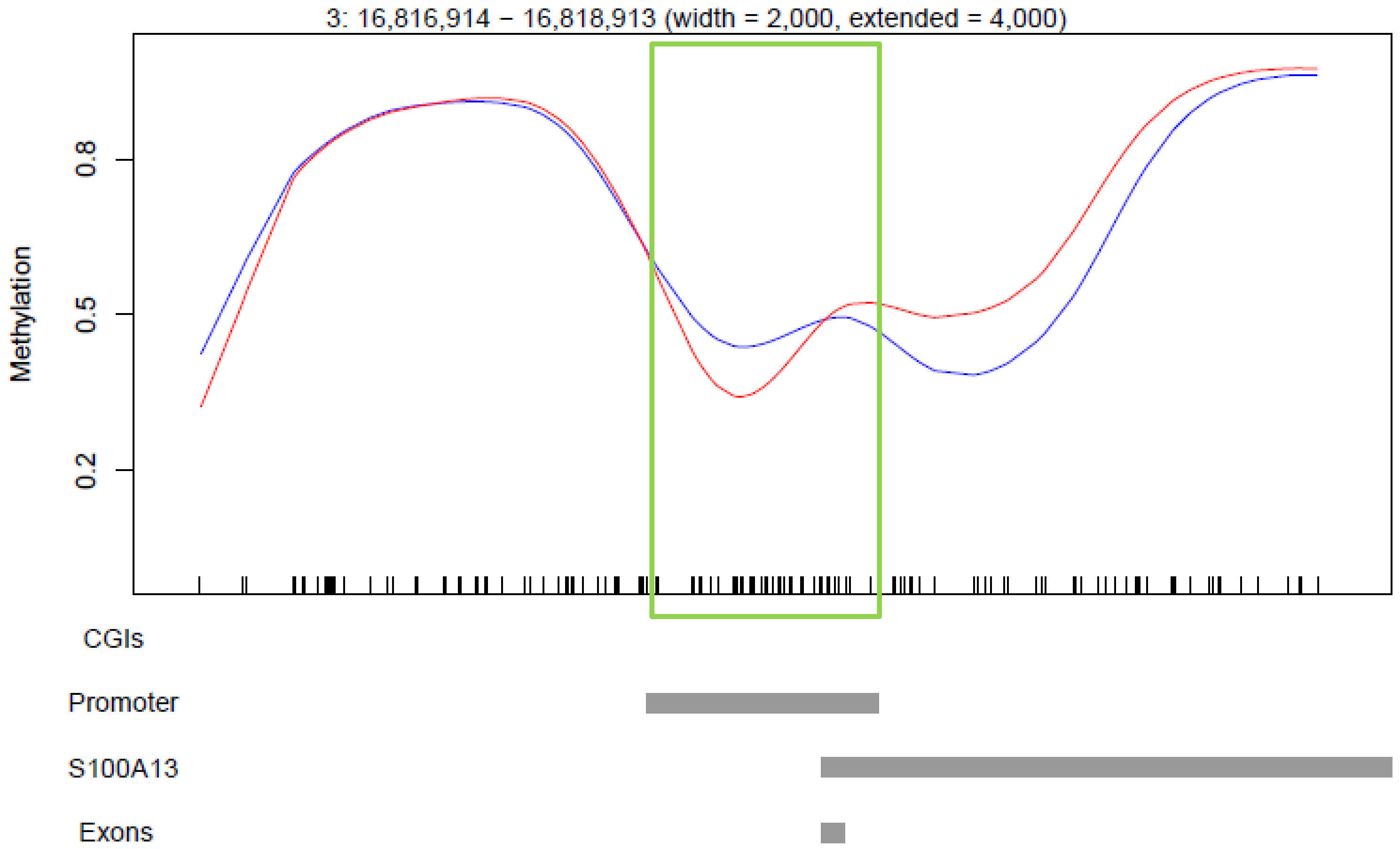
3: 16,816,914 – 16,818,913 (width = 2,000, extended = 4,000)
Methylation
0.8
0.5
0.2
CGIs
Promoter
S100A13
Exons

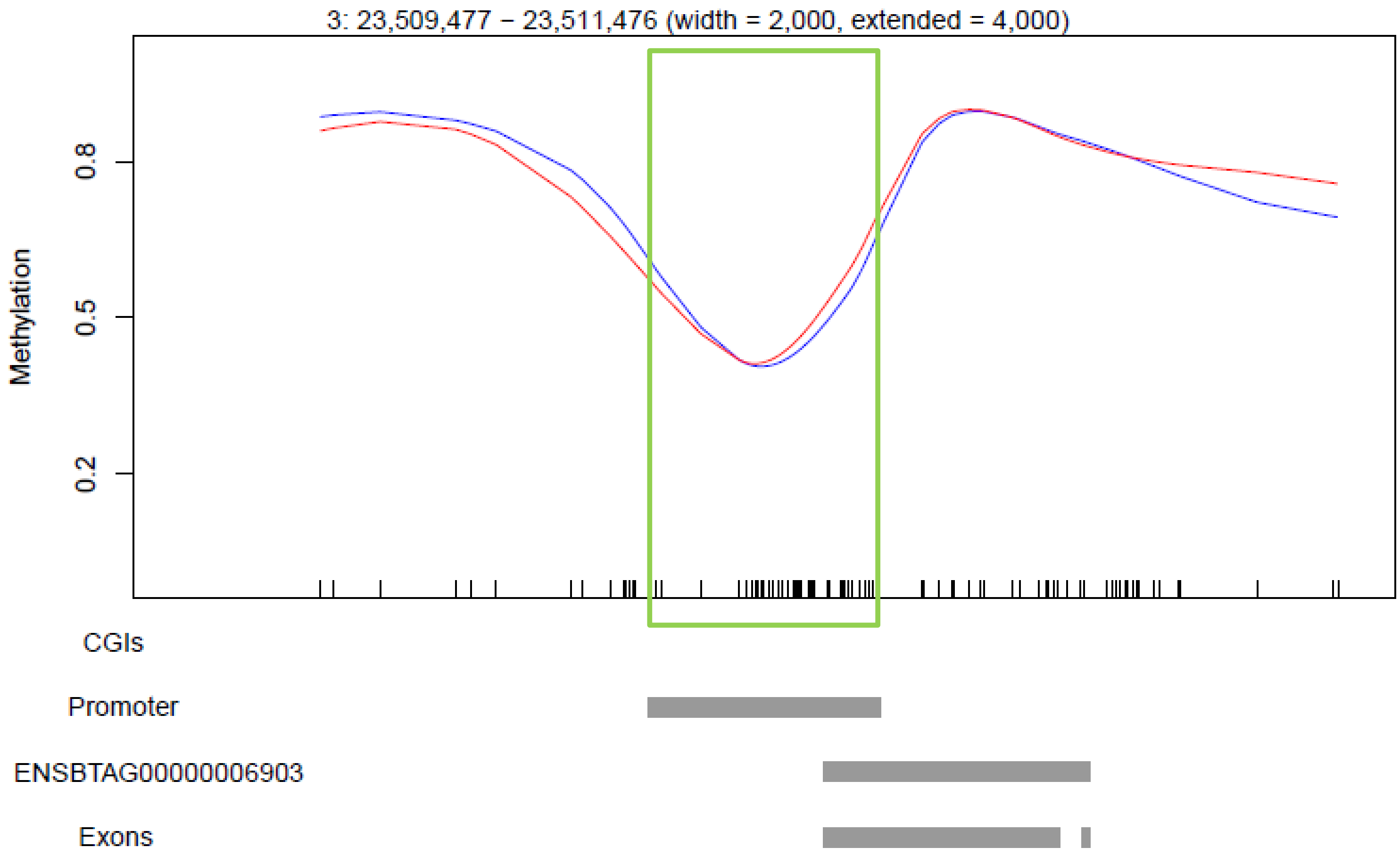

3: 23,509,477 − 23,511,476 (width = 2,000, extended = 4,000)
Methylation
0.2
0.5
0.8
CGIs
Promoter
ENSBTAG00000006903
Exons

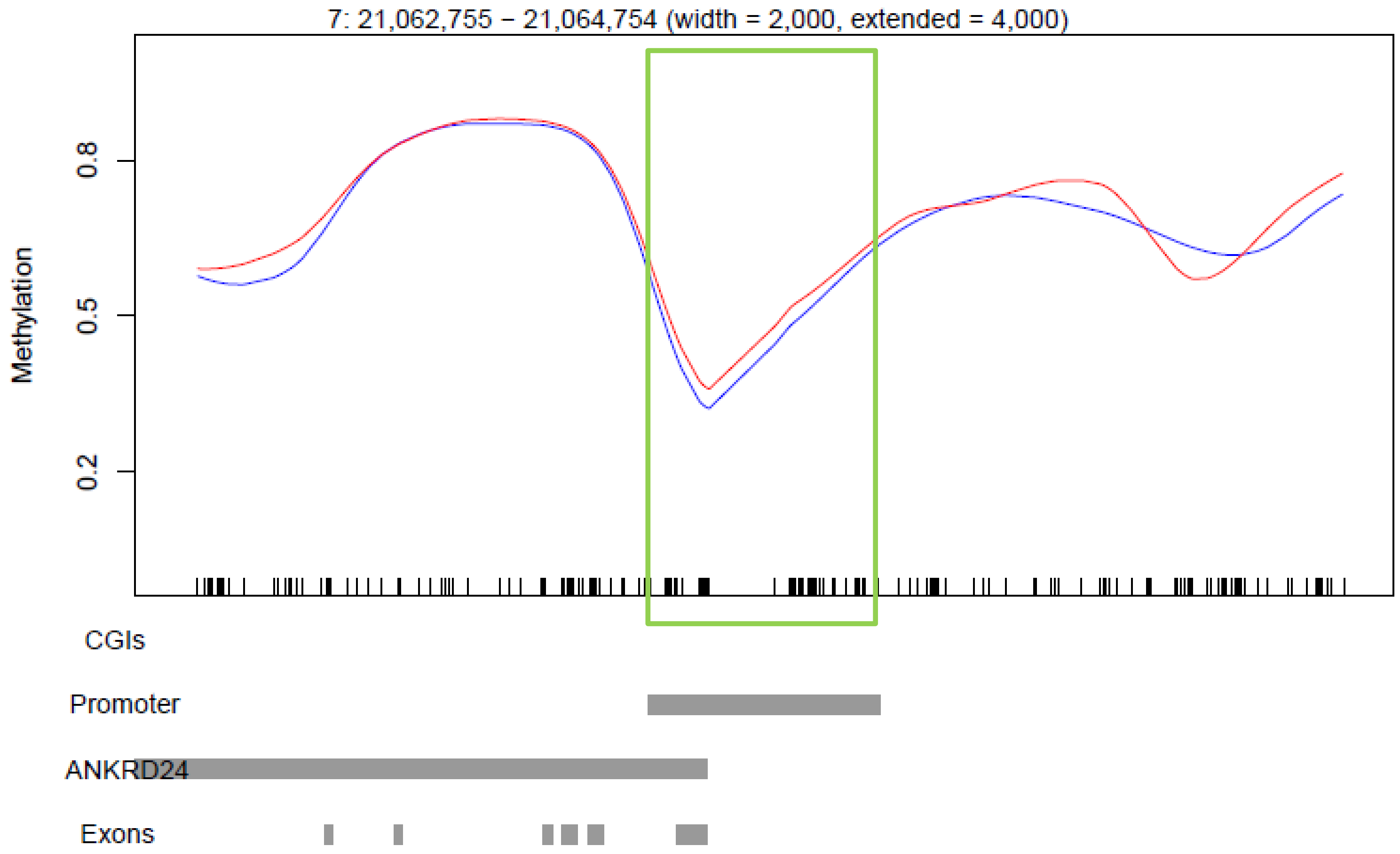
7: 21,062,755 − 21,064,754 (width = 2,000, extended = 4,000)
Methylation
0.8
0.5
0.2
CGIs
Promoter
ANKRD24
Exons

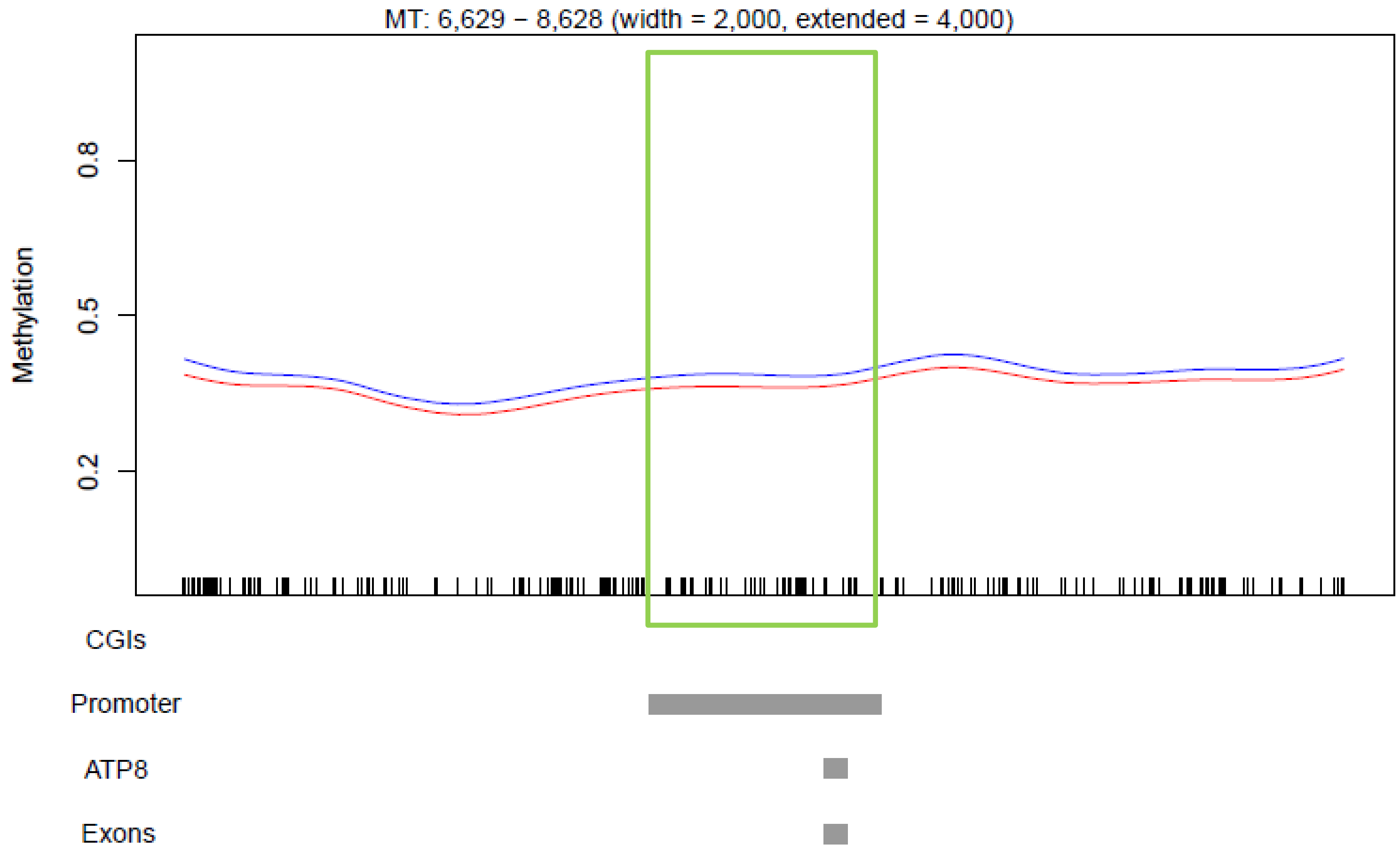
MT: 6,629 − 8,628 (width = 2,000, extended = 4,000)
Methylation
0.8
0.5
0.2
CGIs
Promoter
ATP8
Exons

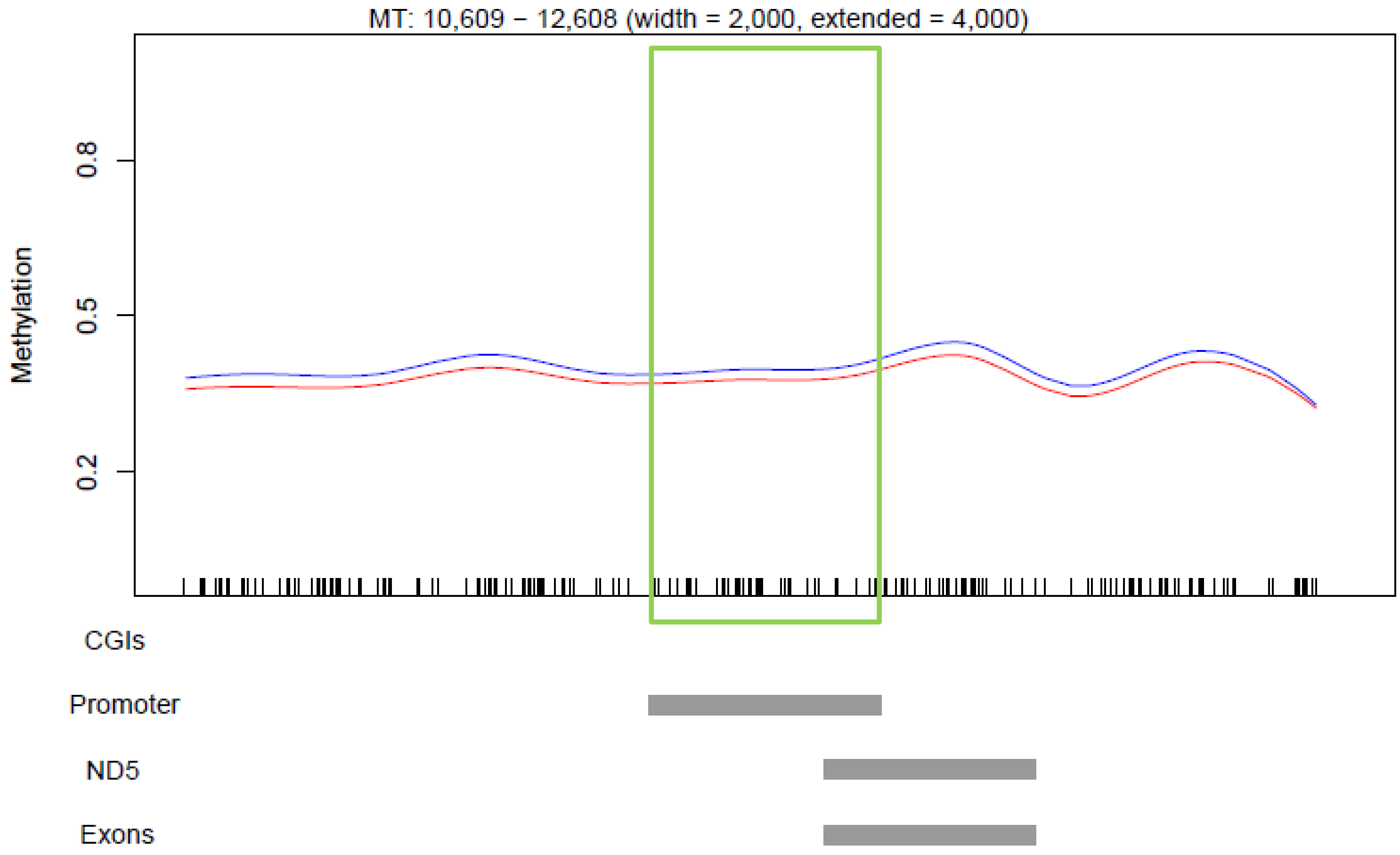

MT: 10,609 − 12,608 (width = 2,000, extended = 4,000)
Methylation
0.8
0.5
0.2
CGIs
Promoter
ND5
Exons

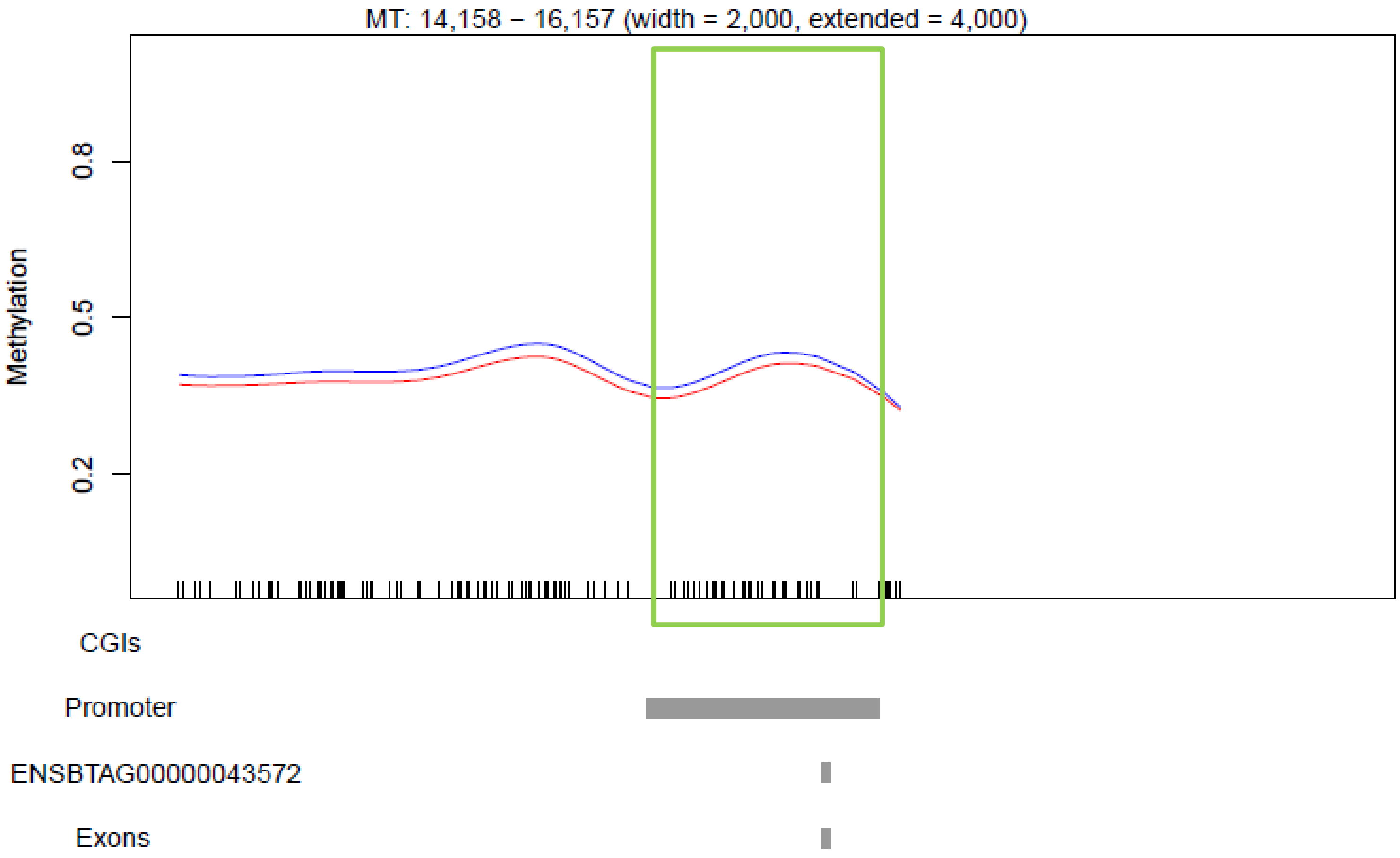
MT: 14,158 − 16,157 (width = 2,000, extended = 4,000)
Methylation
0.2
0.5
0.8
CGIs
Promoter
ENSBTAG00000043572
Exons

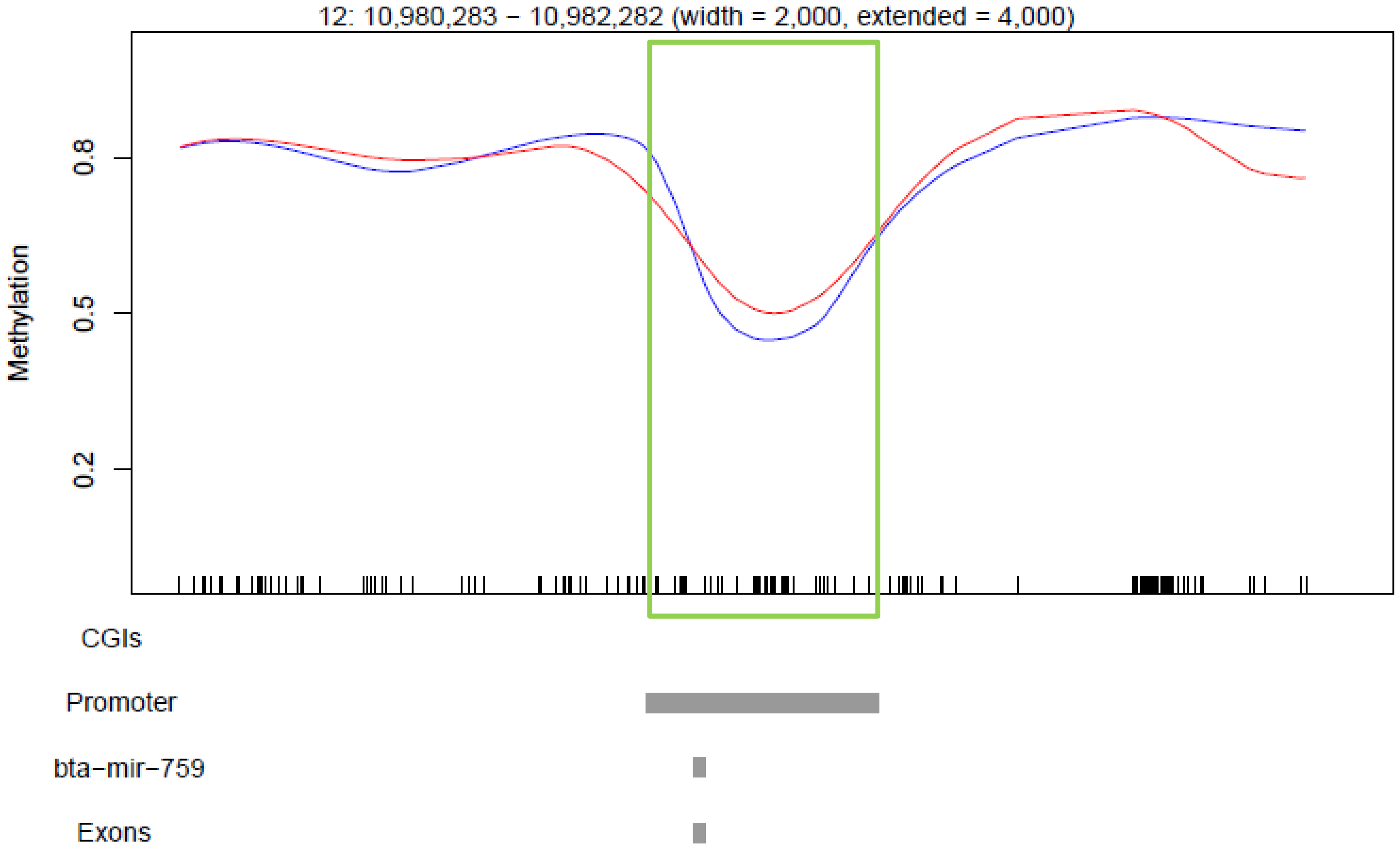

12: 10,980,283 − 10,982,282 (width = 2,000, extended = 4,000)
Methylation
0.8
0.5
0.2
CGIs
Promoter
bta−mir−759
Exons

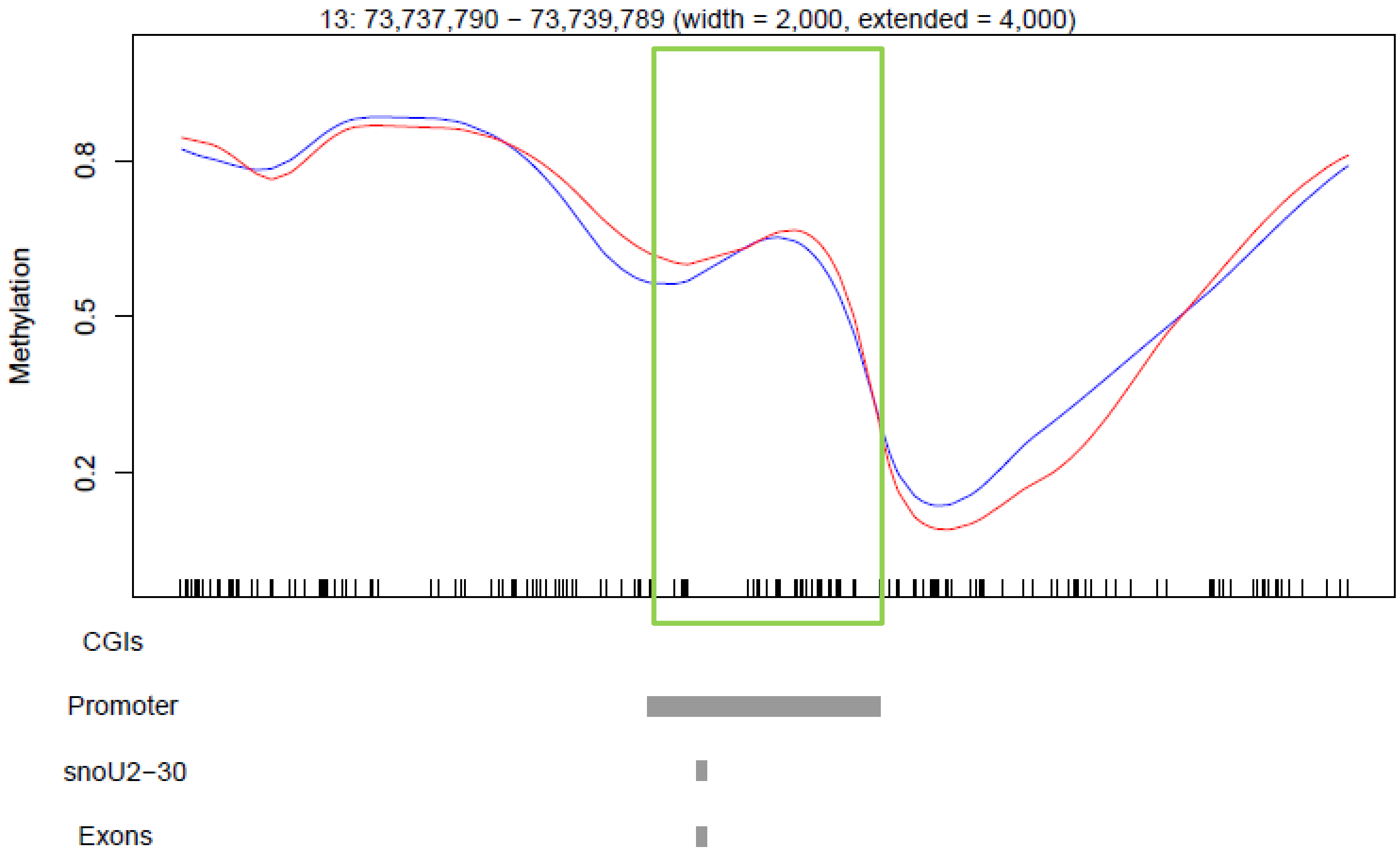
13: 73,737,790 − 73,739,789 (width = 2,000, extended = 4,000)
Methylation
0.8
0.5
0.2
CGIs
Promoter
snoU2−30
Exons

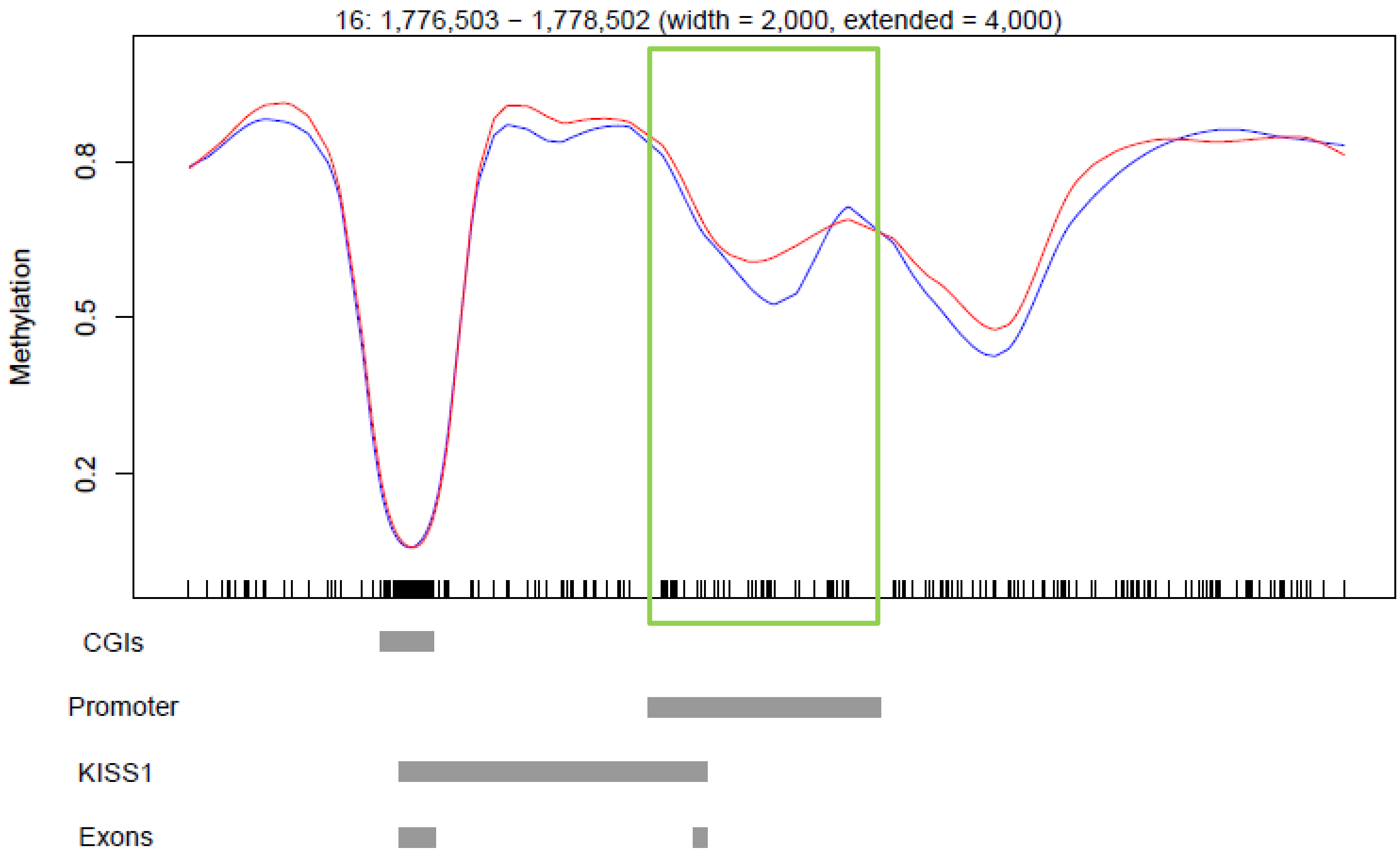

16: 1,776,503 – 1,778,502 (width = 2,000, extended = 4,000)
Methylation
0.8
0.5
0.2
CGIs
Promoter
KISS1
Exons

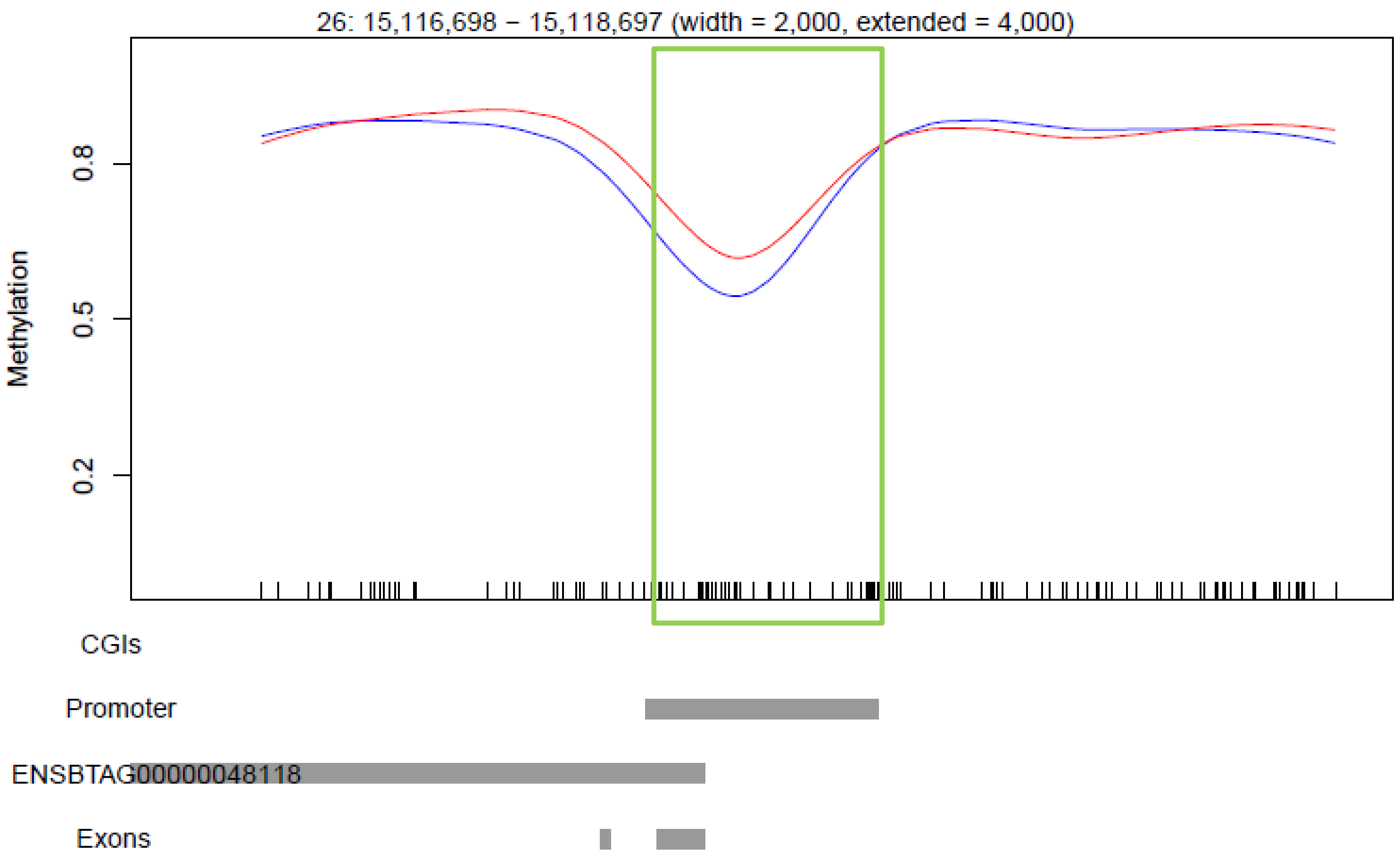
26: 15,116,698 − 15,118,697 (width = 2,000, extended = 4,000)
Methylation
0.8
0.5
0.2
CGIs
Promoter
ENSBTAG00000048118
Exons

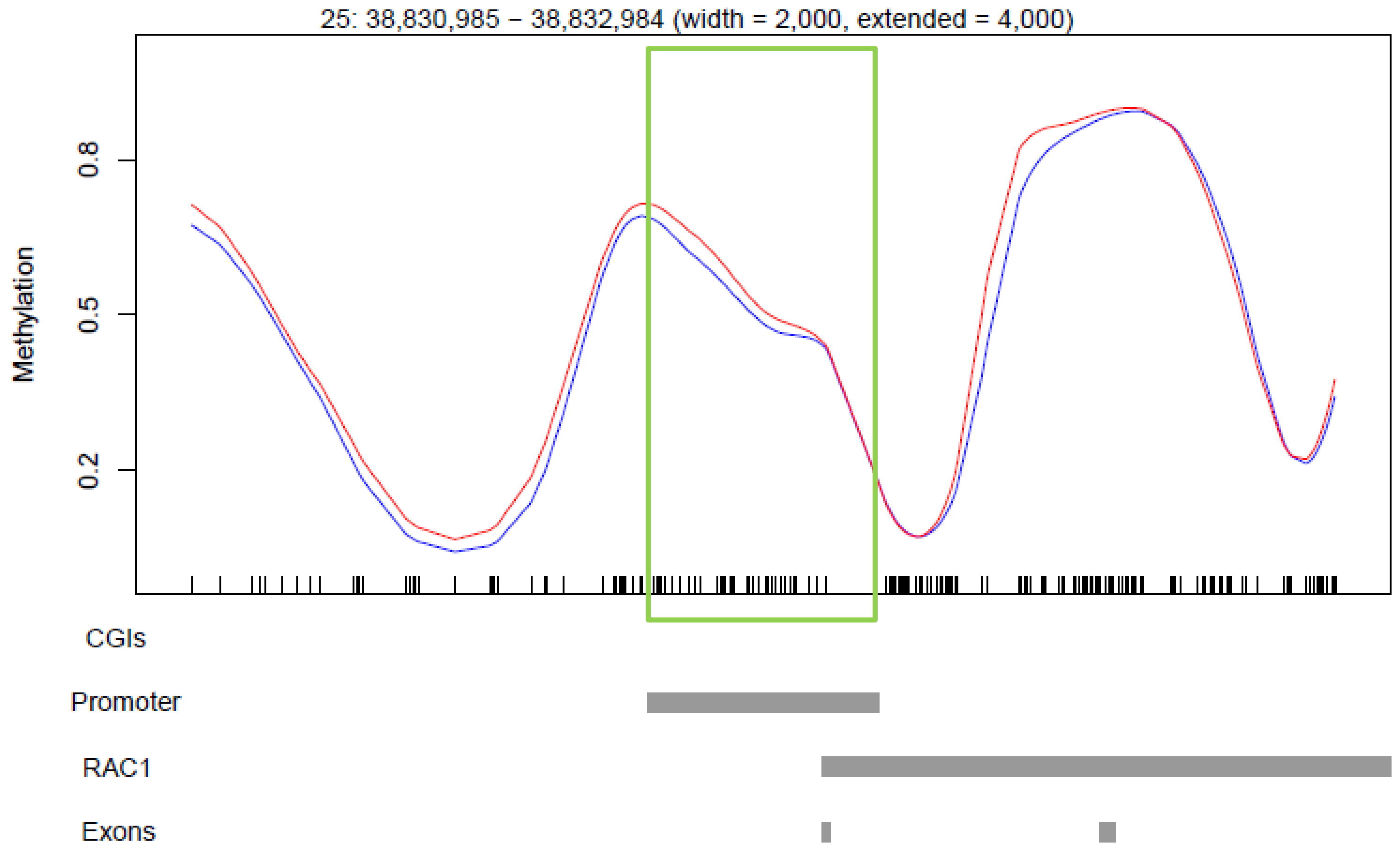

25: 38,830,985 − 38,832,984 (width = 2,000, extended = 4,000)
Methylation
0.2
0.5
0.8
CGIs
Promoter
RAC1
Exons

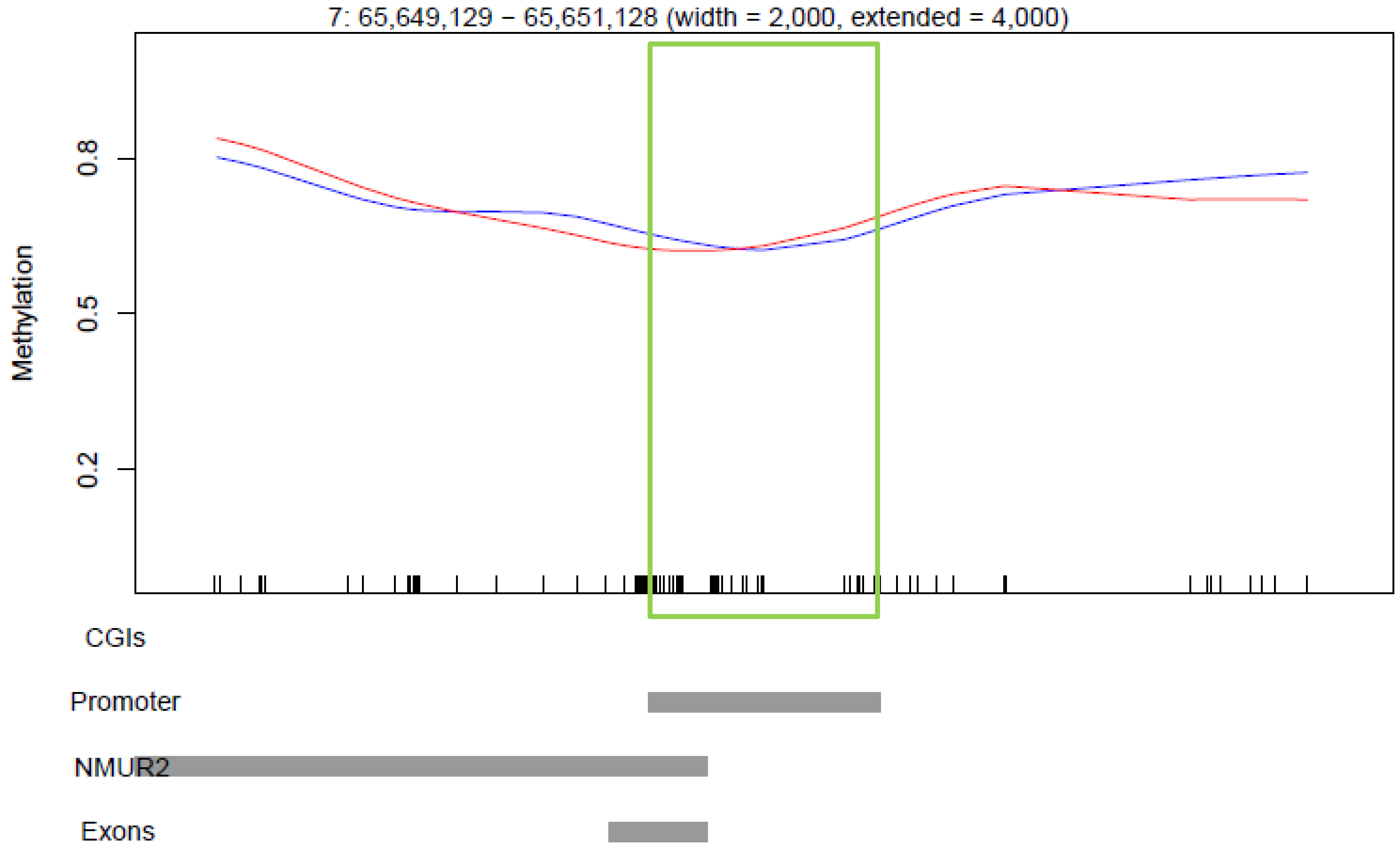
7: 65,649,129 − 65,651,128 (width = 2,000, extended = 4,000)
Methylation
0.2
0.5
0.8
CGIs
Promoter
NMUR2
Exons

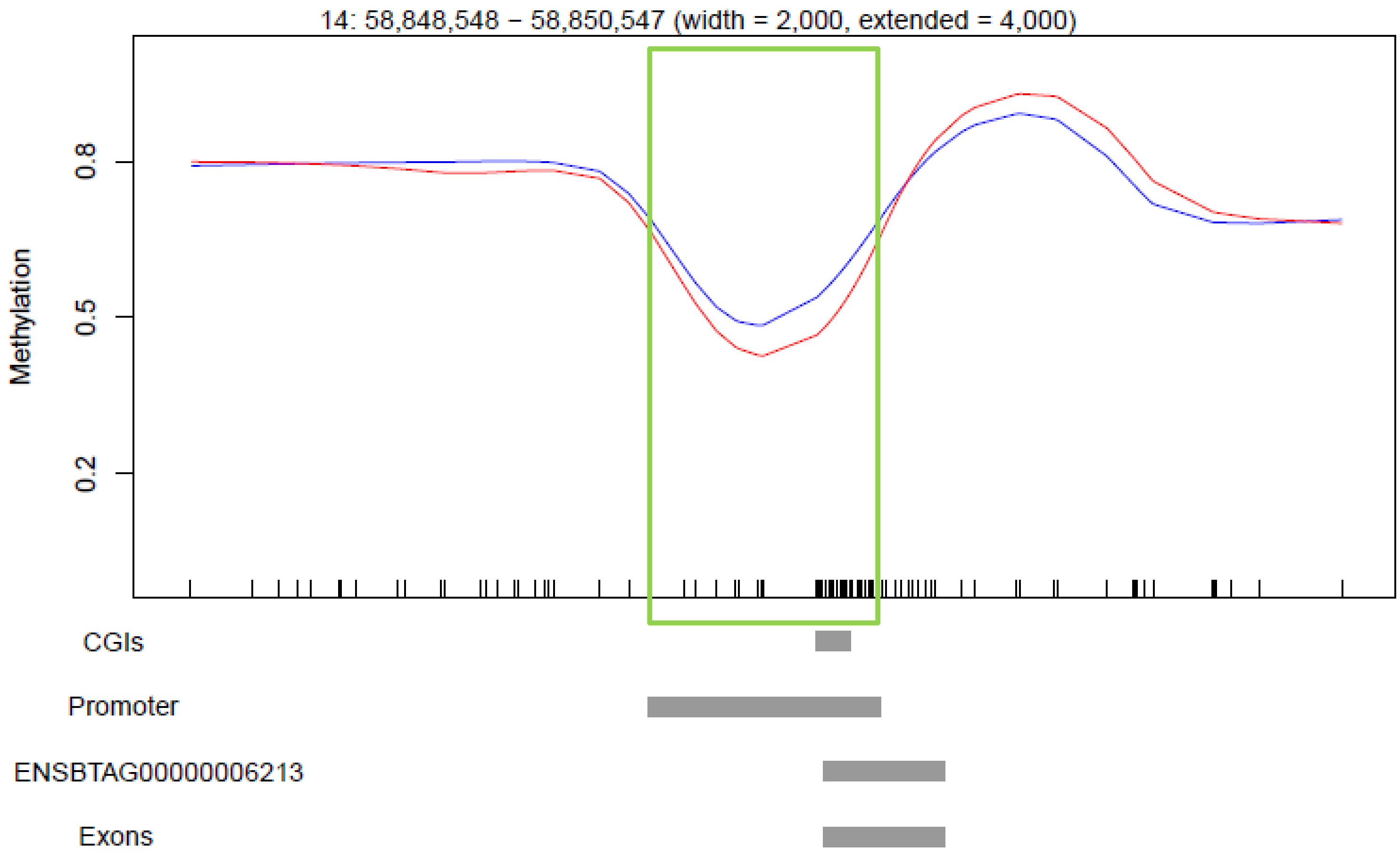
14: 58,848,548 − 58,850,547 (width = 2,000, extended = 4,000)
Methylation
0.8
0.5
0.2
CGIs
Promoter
ENSBTAG00000006213
Exons

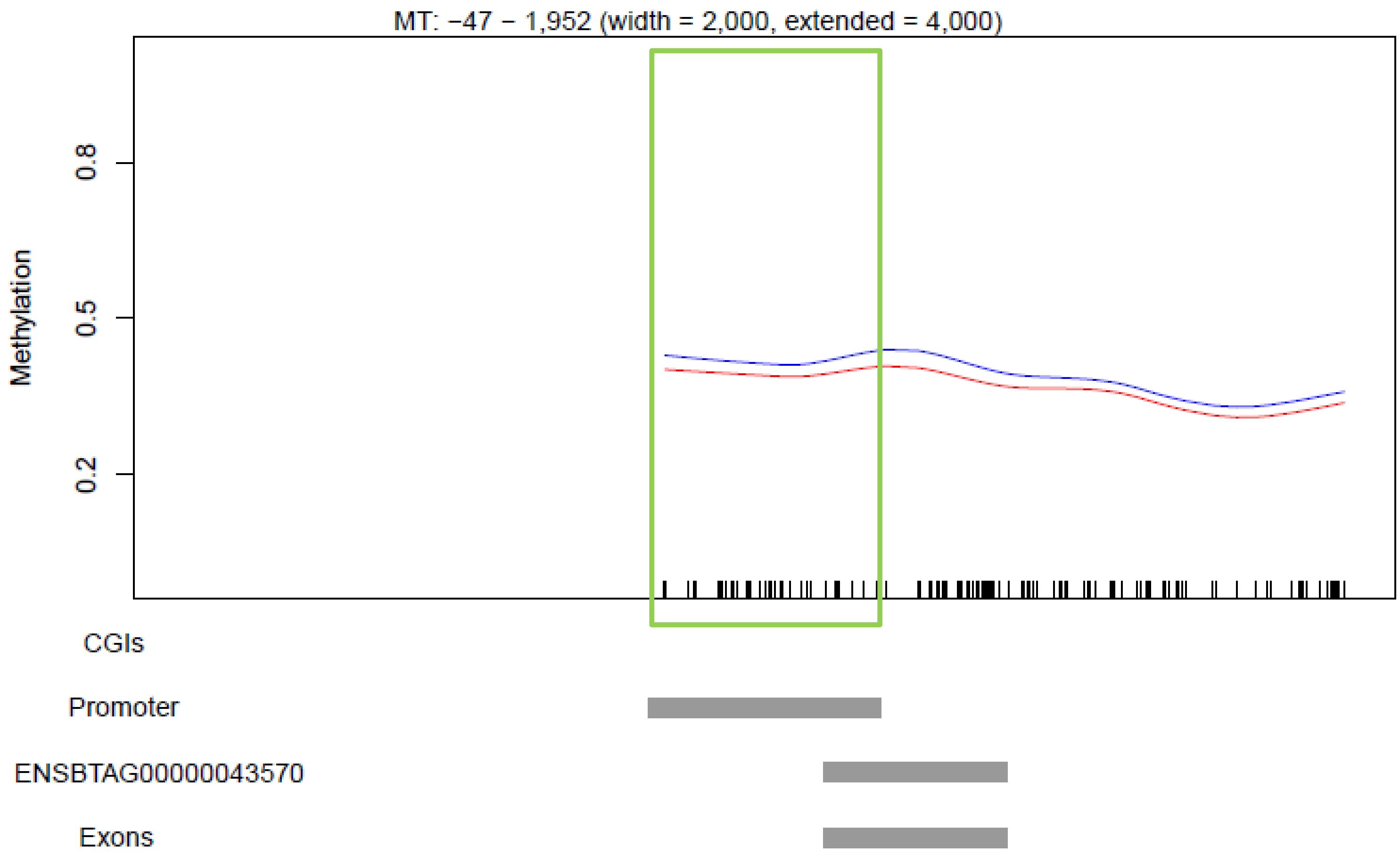

MT: −47 − 1,952 (width = 2,000, extended = 4,000)
Methylation
0.8
0.5
0.2
CGIs
Promoter
ENSBTAG00000043570
Exons

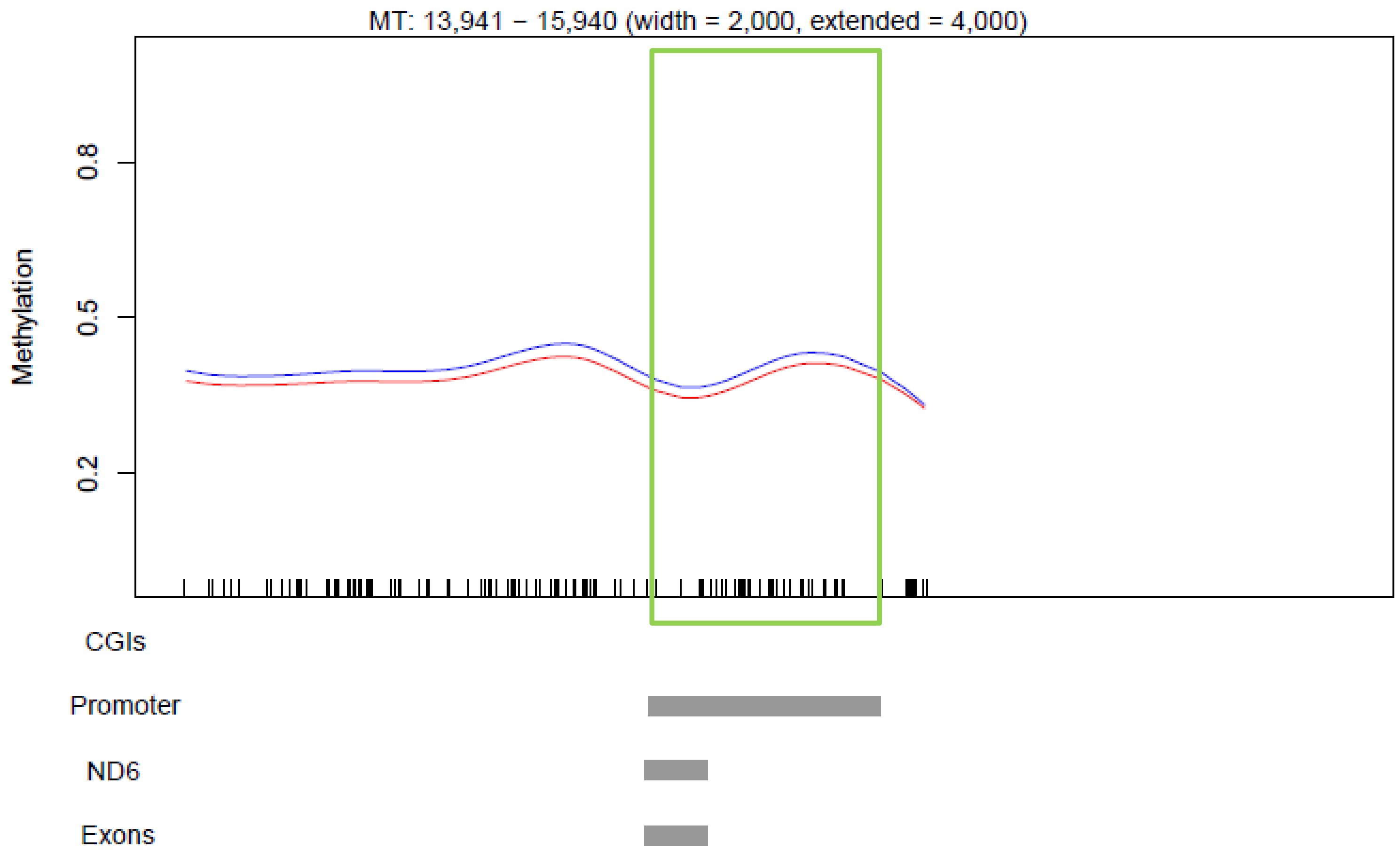
MT: 13,941 − 15,940 (width = 2,000, extended = 4,000)
Methylation
0.8
0.5
0.2
CGIs
Promoter
ND6
Exons

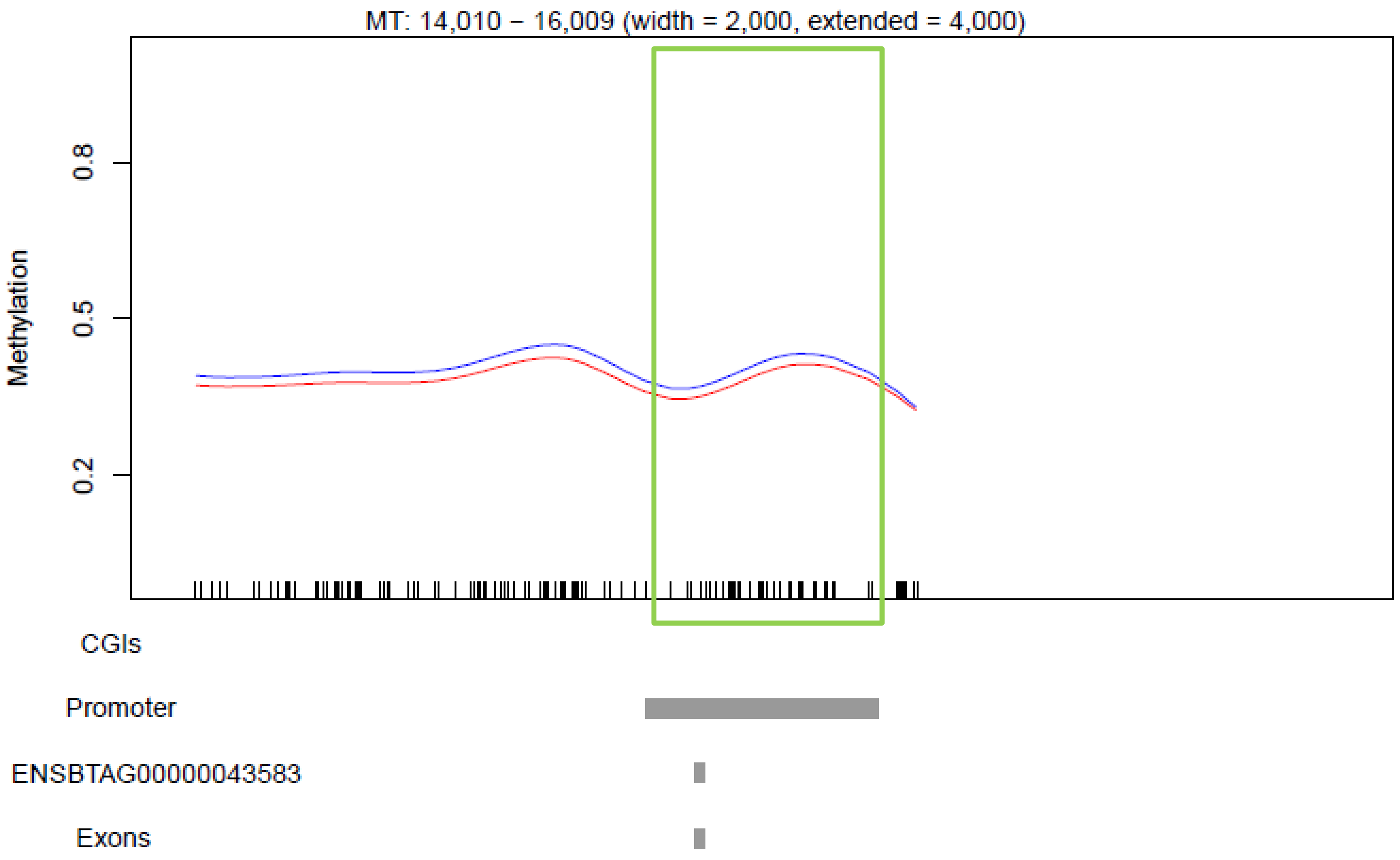
MT: 14,010 − 16,009 (width = 2,000, extended = 4,000)
Methylation
0.8
0.5
0.2
CGIs
Promoter
ENSBTAG00000043583
Exons

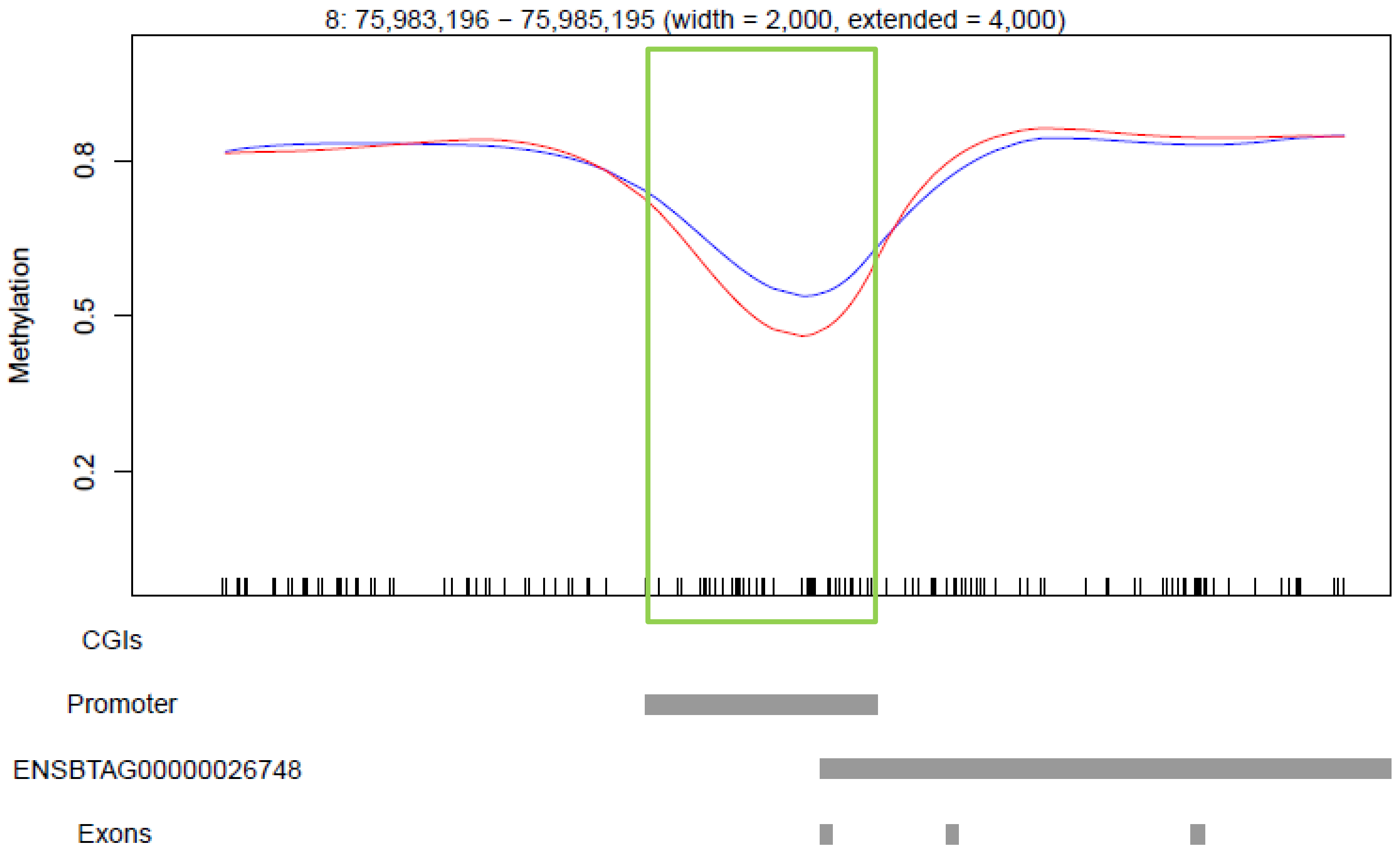

8: 75,983,196 − 75,985,195 (width = 2,000, extended = 4,000)
Methylation
0.2
0.5
0.8
CGIs
Promoter
ENSBTAG00000026748
Exons

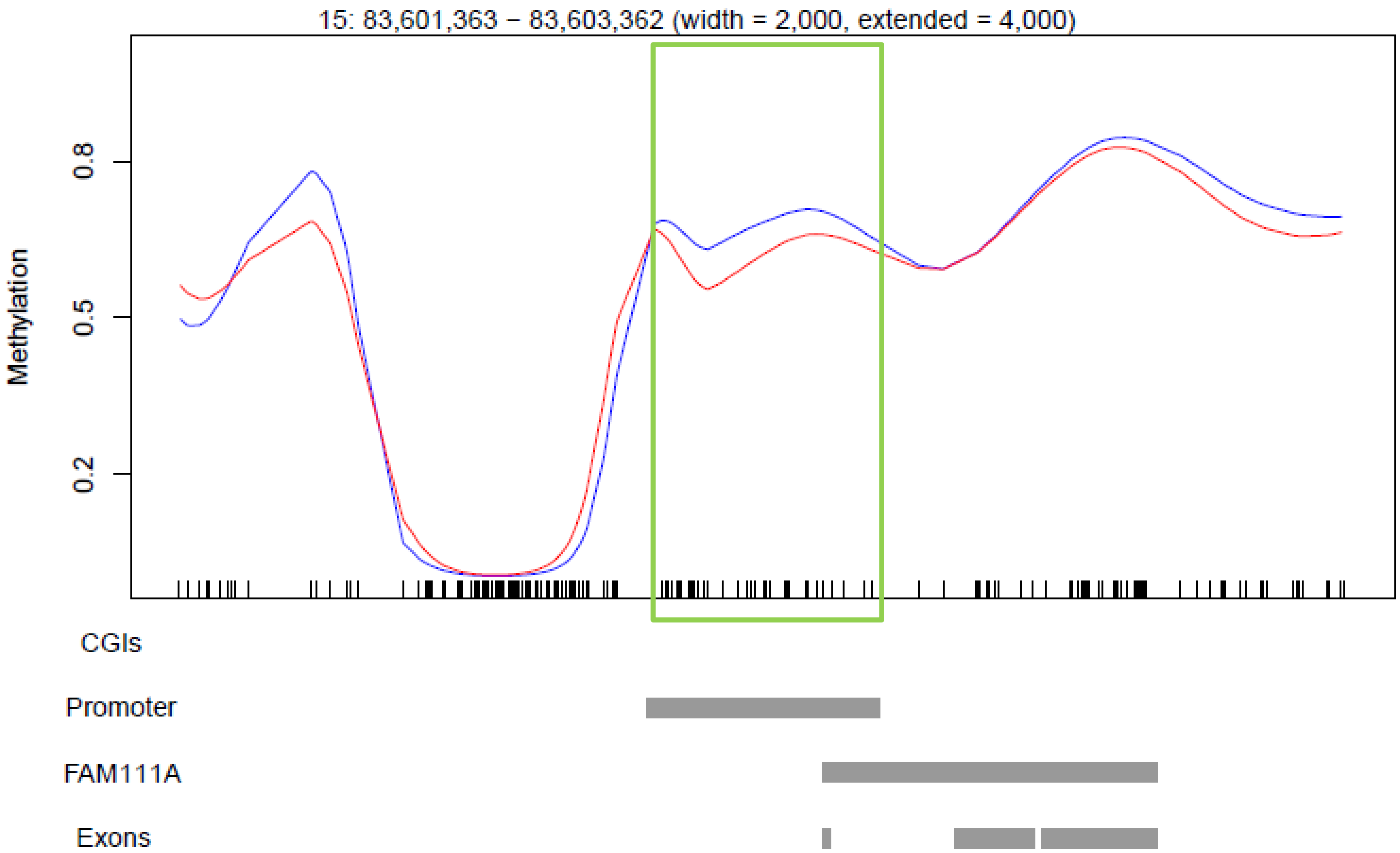
15: 83,601,363 − 83,603,362 (width = 2,000, extended = 4,000)
Methylation
0.8
0.5
0.2
CGIs
Promoter
FAM111A
Exons

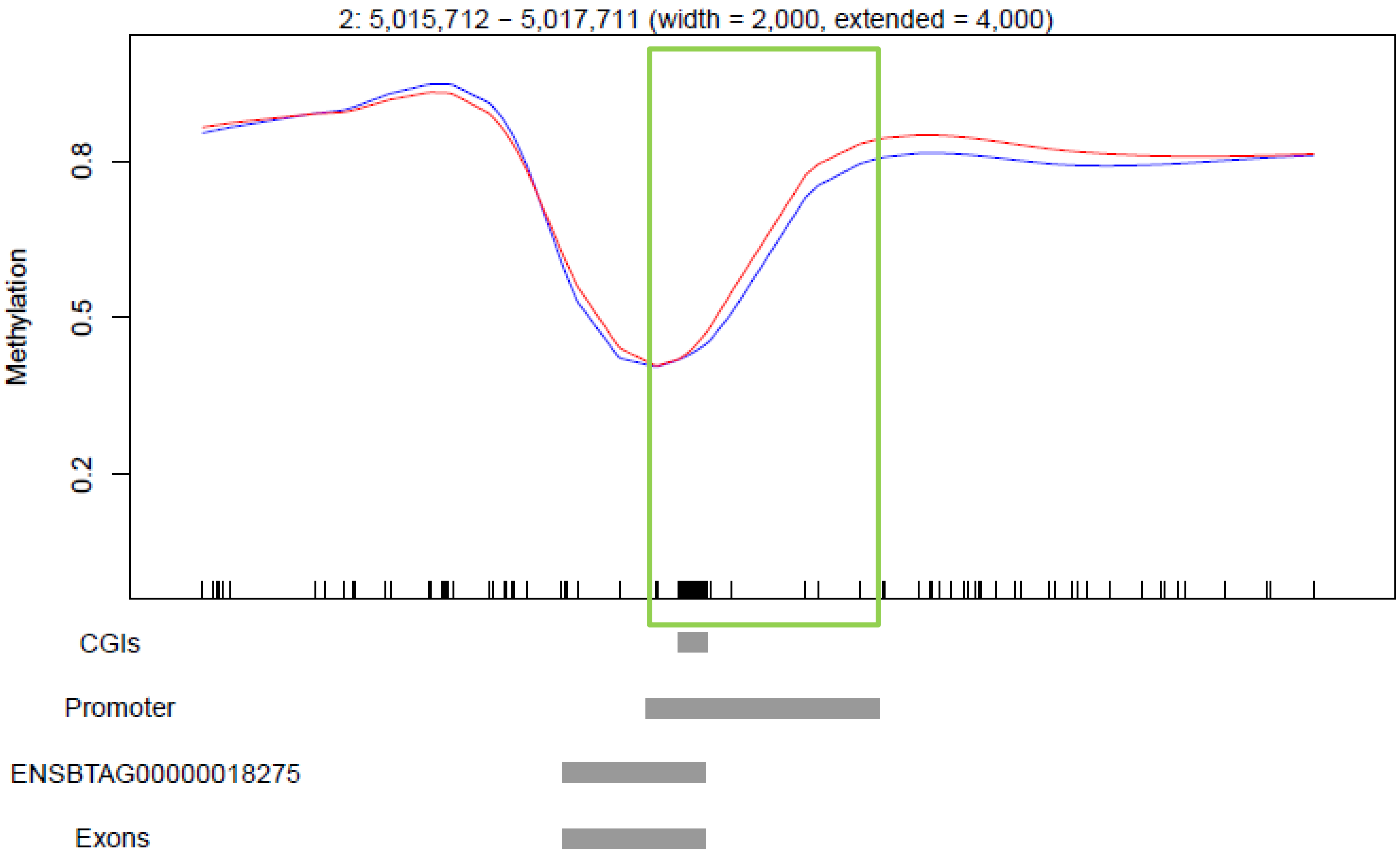
2: 5,015,712 − 5,017,711 (width = 2,000, extended = 4,000)
Methylation
0.2
0.5
0.8
CGIs
Promoter
ENSBTAG00000018275
Exons

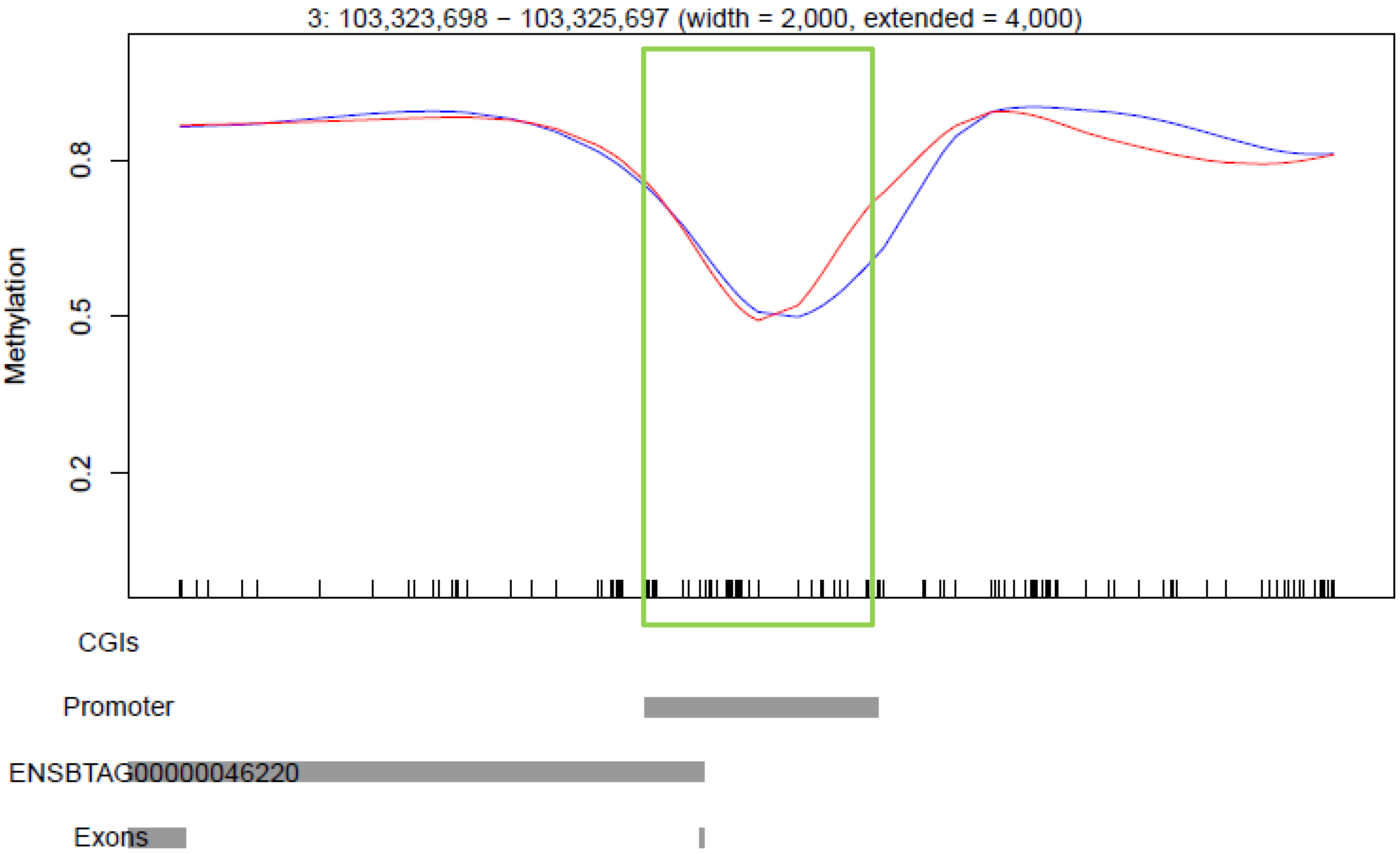

3: 103,323,698 − 103,325,697 (width = 2,000, extended = 4,000)
Methylation
0.8
0.5
0.2
CGIs
Promoter
ENSBTAG00000046220
Exons

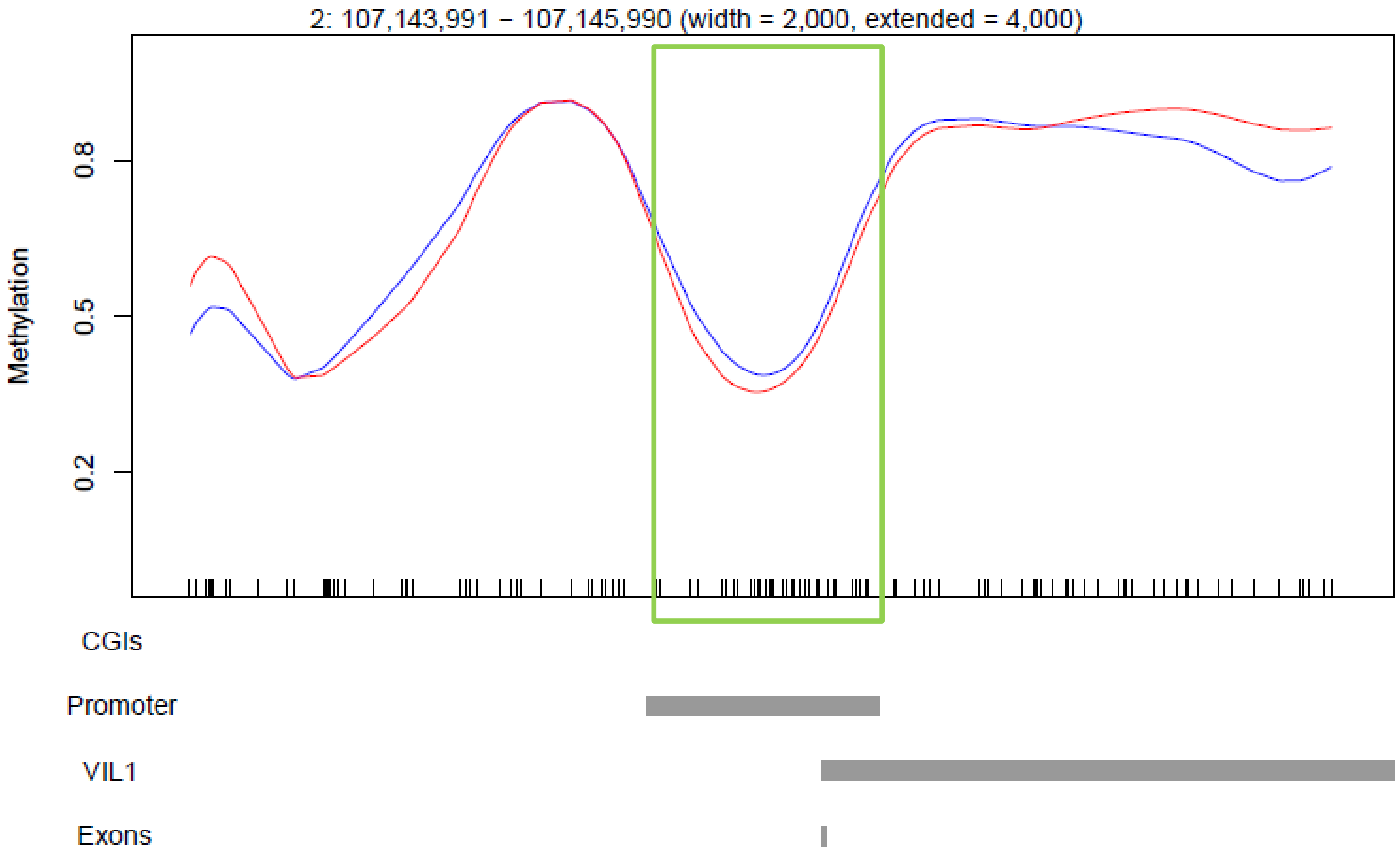
2: 107,143,991 − 107,145,990 (width = 2,000, extended = 4,000)
Methylation
0.2
0.5
0.8
CGIs
Promoter
VIL1
Exons

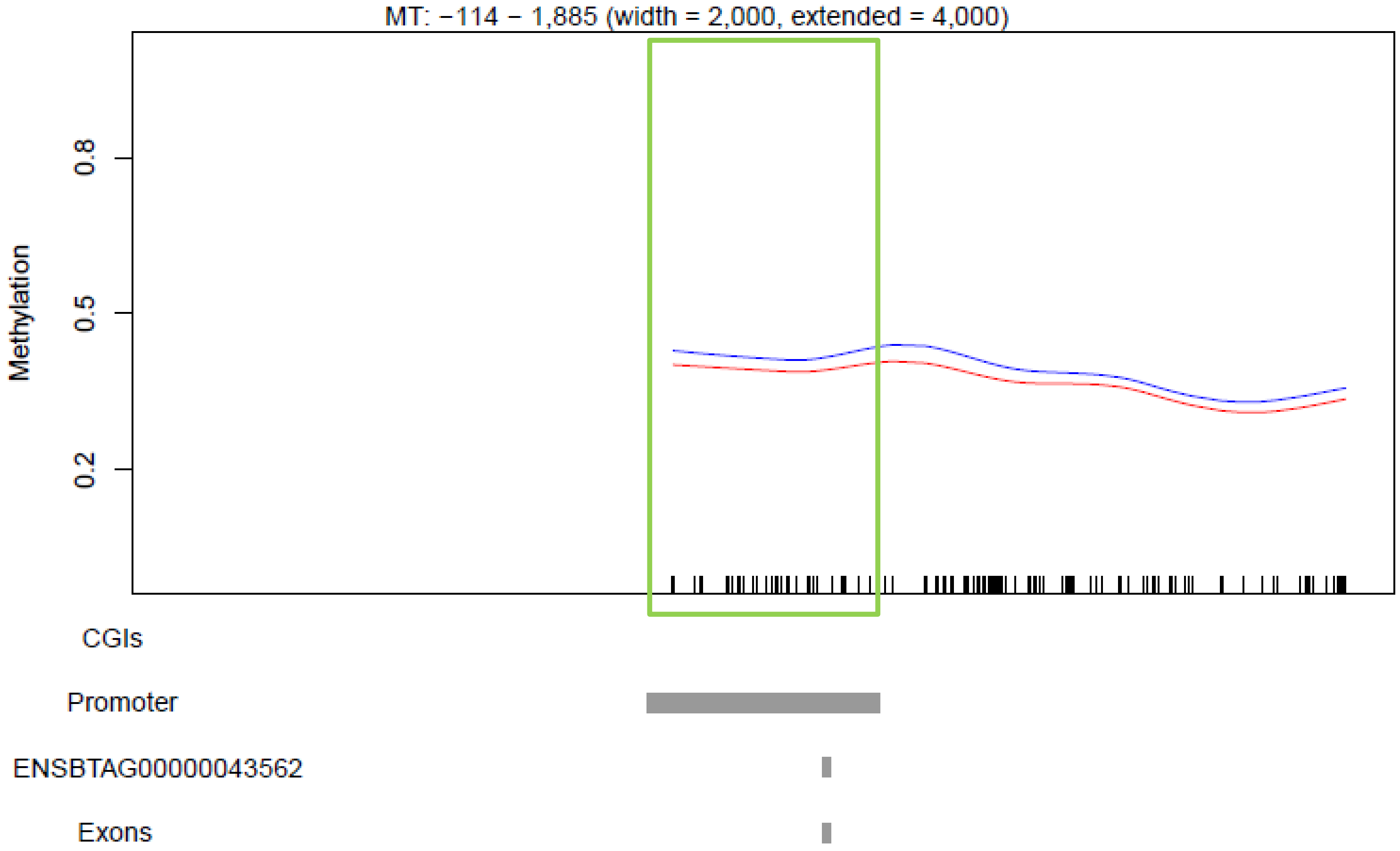
MT: −114 − 1,885 (width = 2,000, extended = 4,000)
Methylation
0.8
0.5
0.2
CGIs
Promoter
ENSBTAG00000043562
Exons

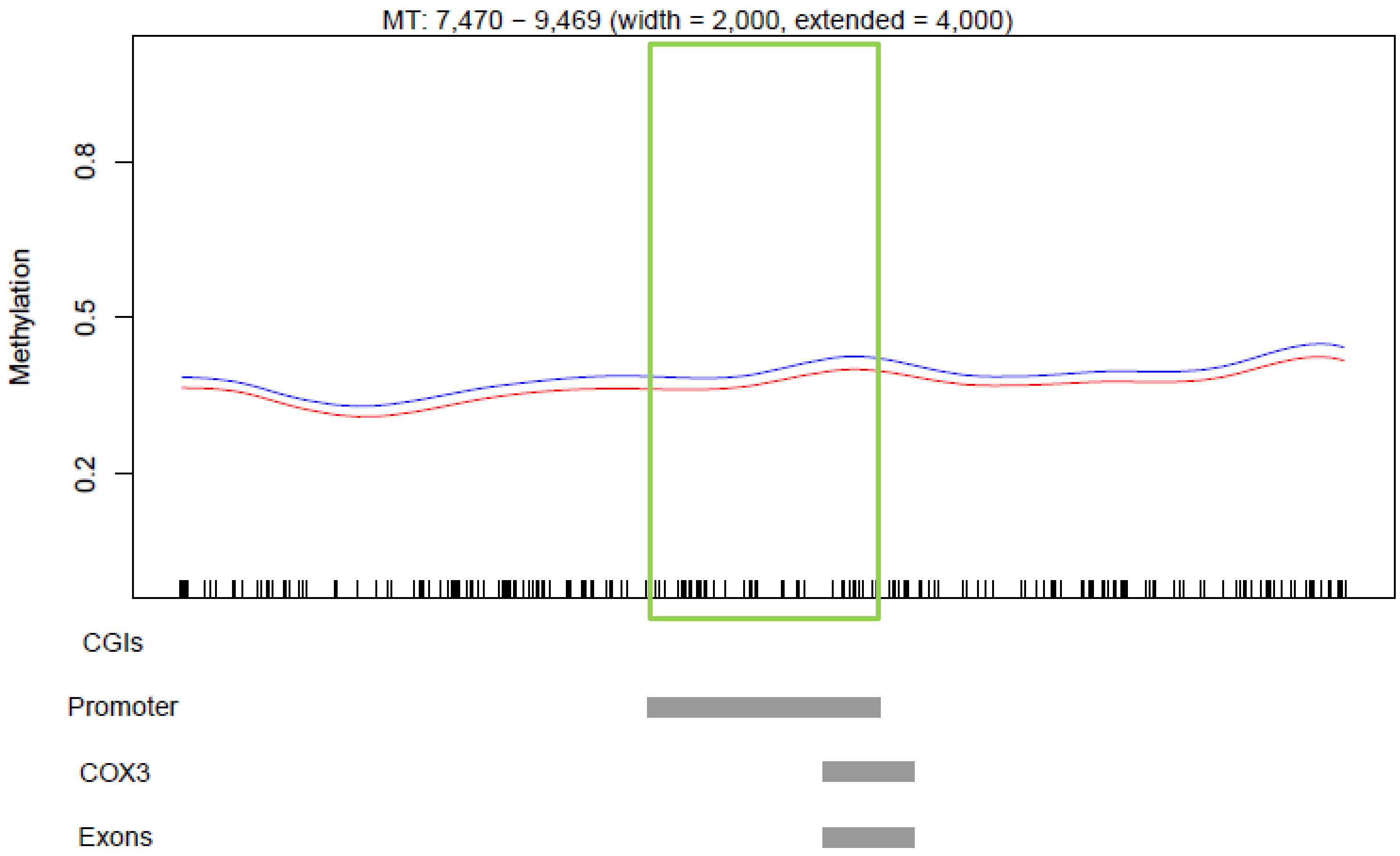
MT: 7,470 − 9,469 (width = 2,000, extended = 4,000)
Methylation
0.8
0.5
0.2
CGIs
Promoter
COX3
Exons

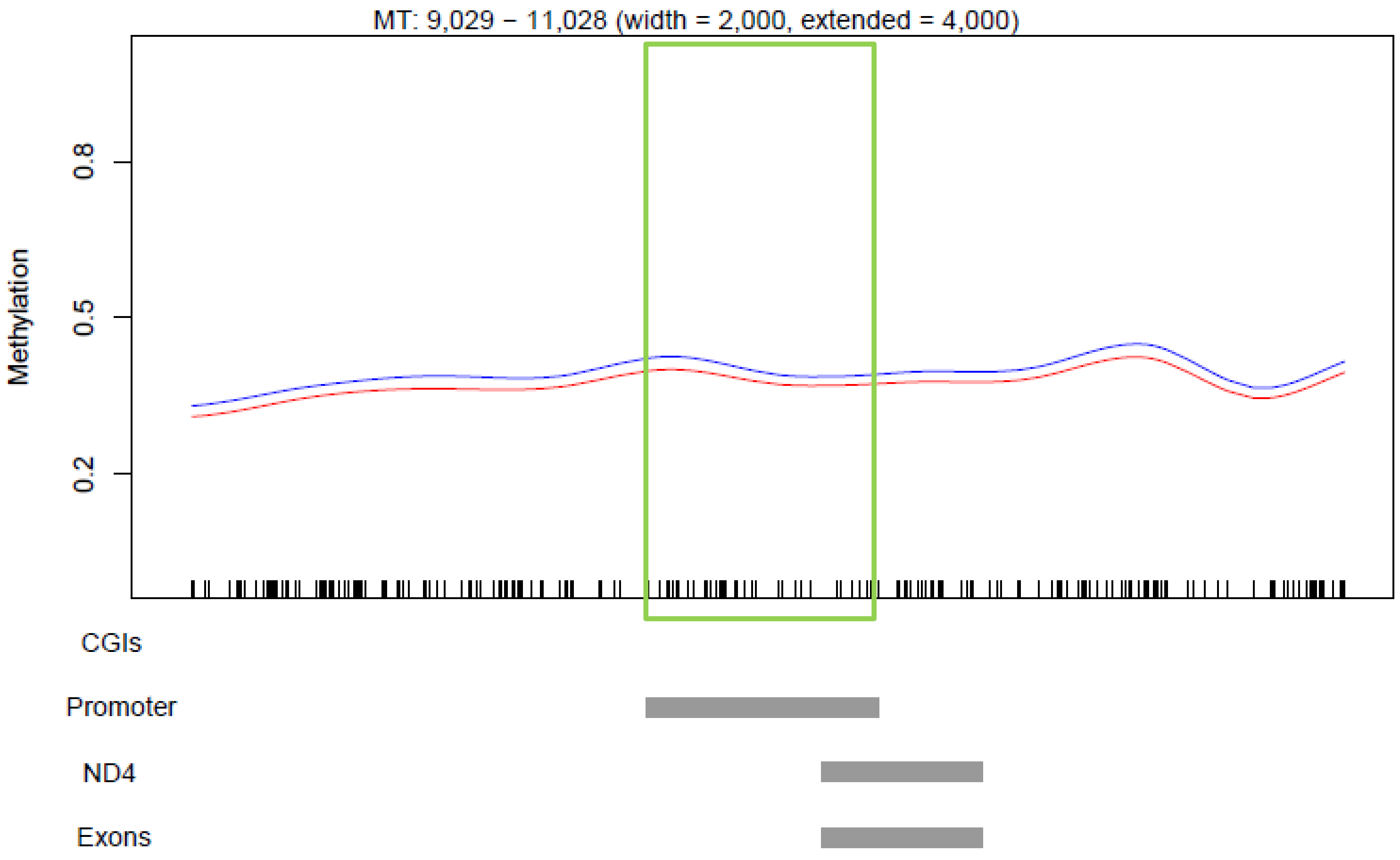
MT: 9,029 − 11,028 (width = 2,000, extended = 4,000)
Methylation
0.8
0.5
0.2
CGIs
Promoter
ND4
Exons

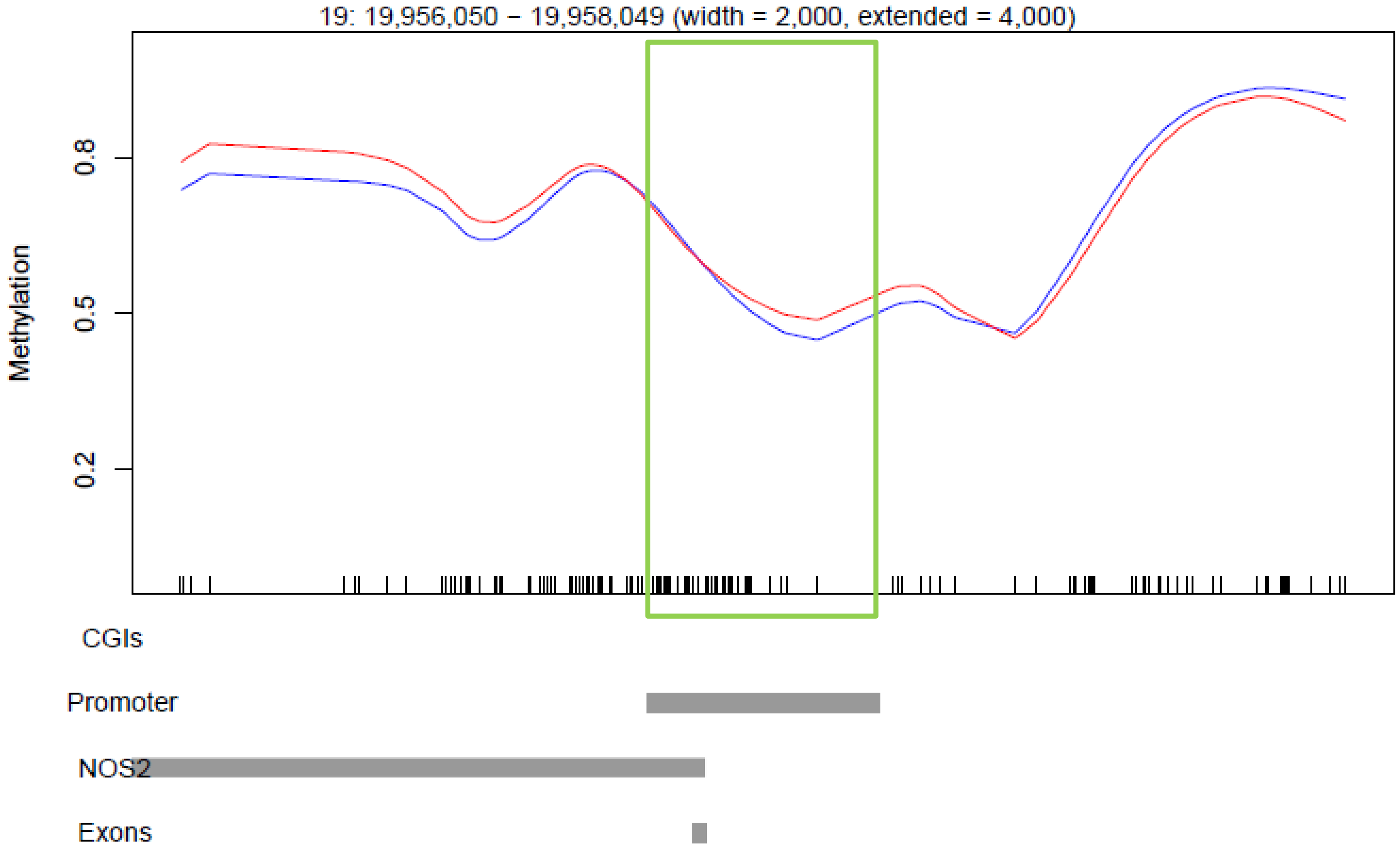
19: 19,956,050 − 19,958,049 (width = 2,000, extended = 4,000)
Methylation
0.8
0.5
0.2
CGIs
Promoter
NOS2
Exons

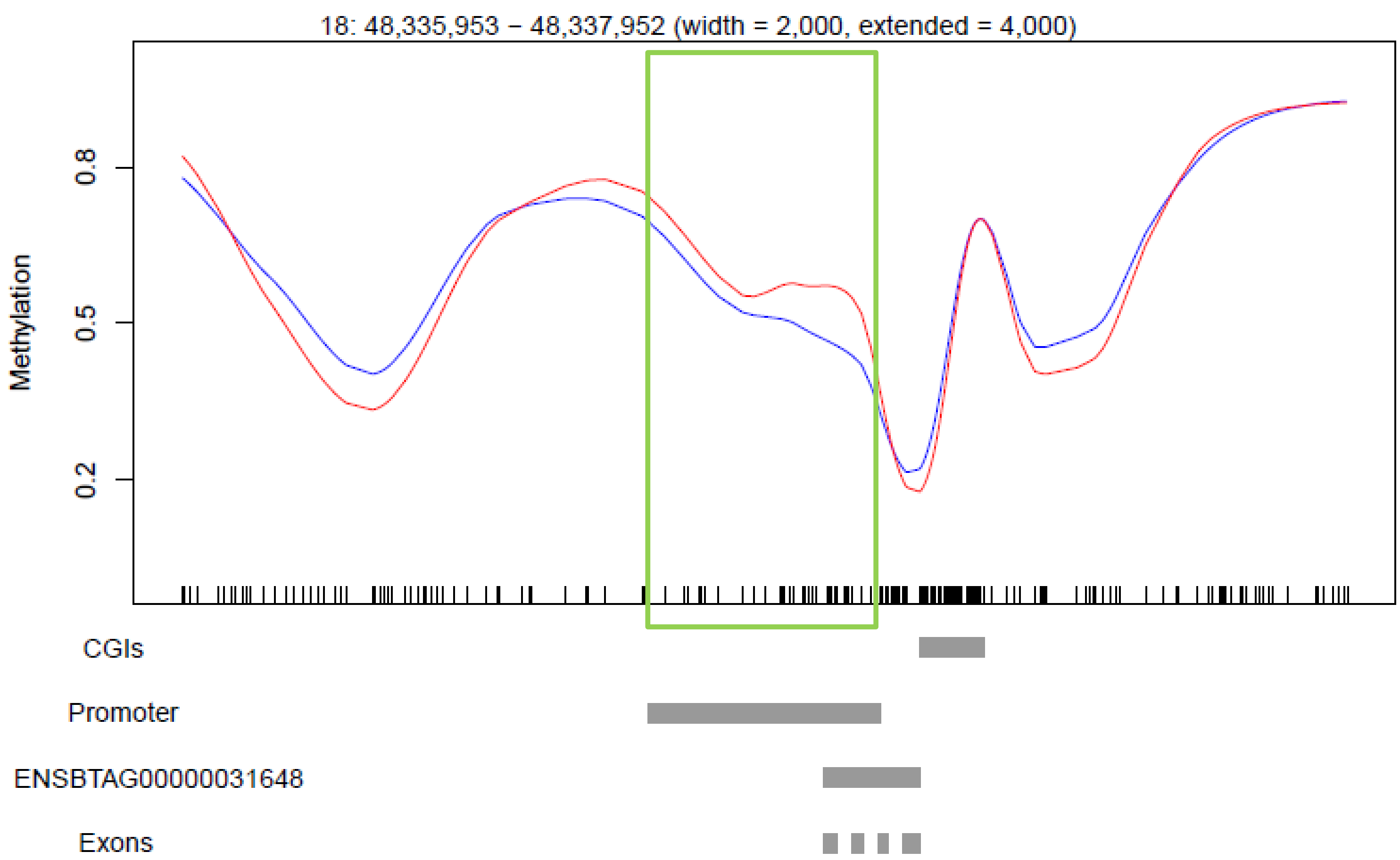
18: 48,335,953 – 48,337,952 (width = 2,000, extended = 4,000)
Methylation
0.8
0.5
0.2
CGIs
Promoter
ENSBTAG00000031648
Exons

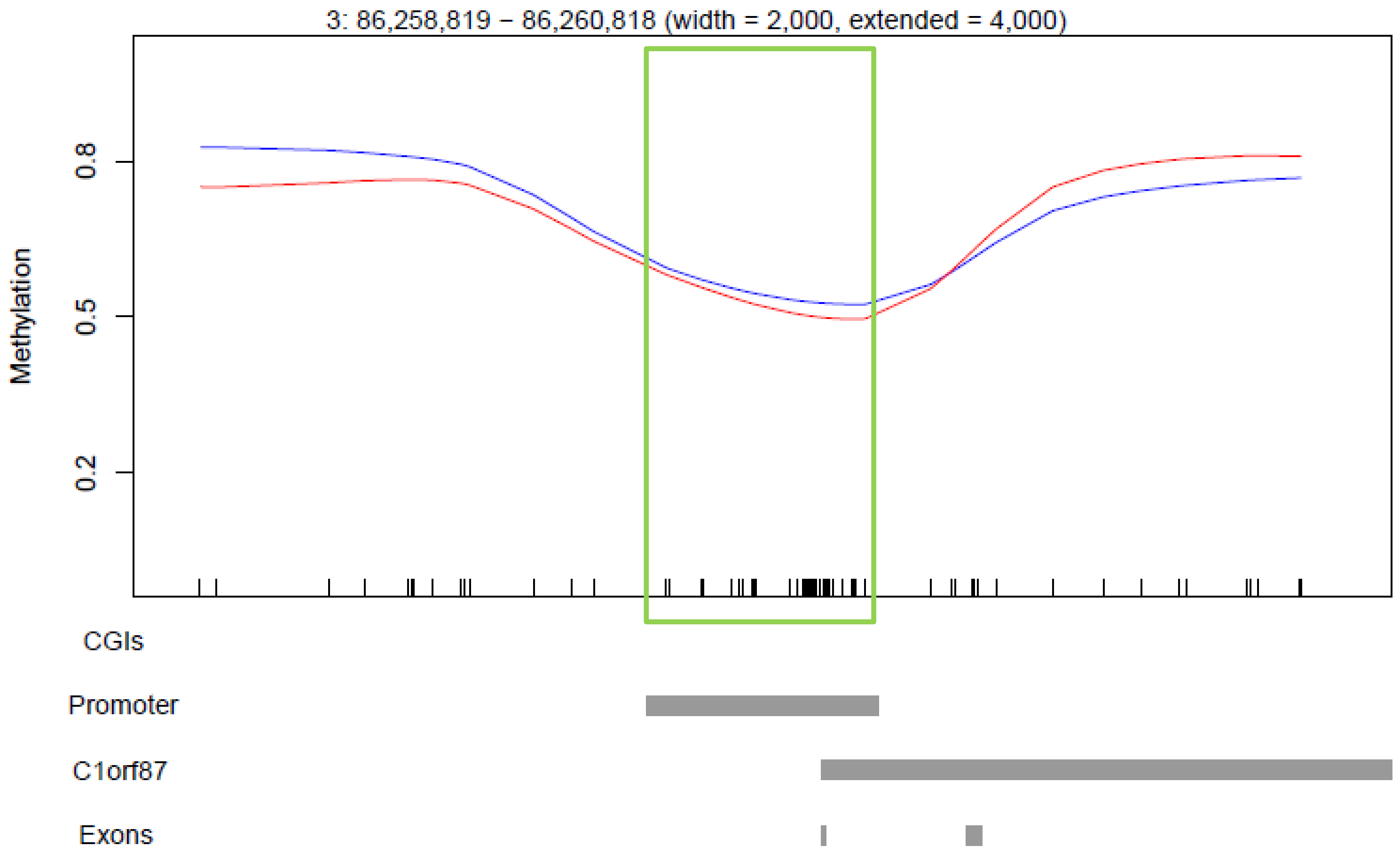
3: 86,258,819 − 86,260,818 (width = 2,000, extended = 4,000)
Methylation
0.8
0.5
0.2
CGIs
Promoter
C1orf87
Exons

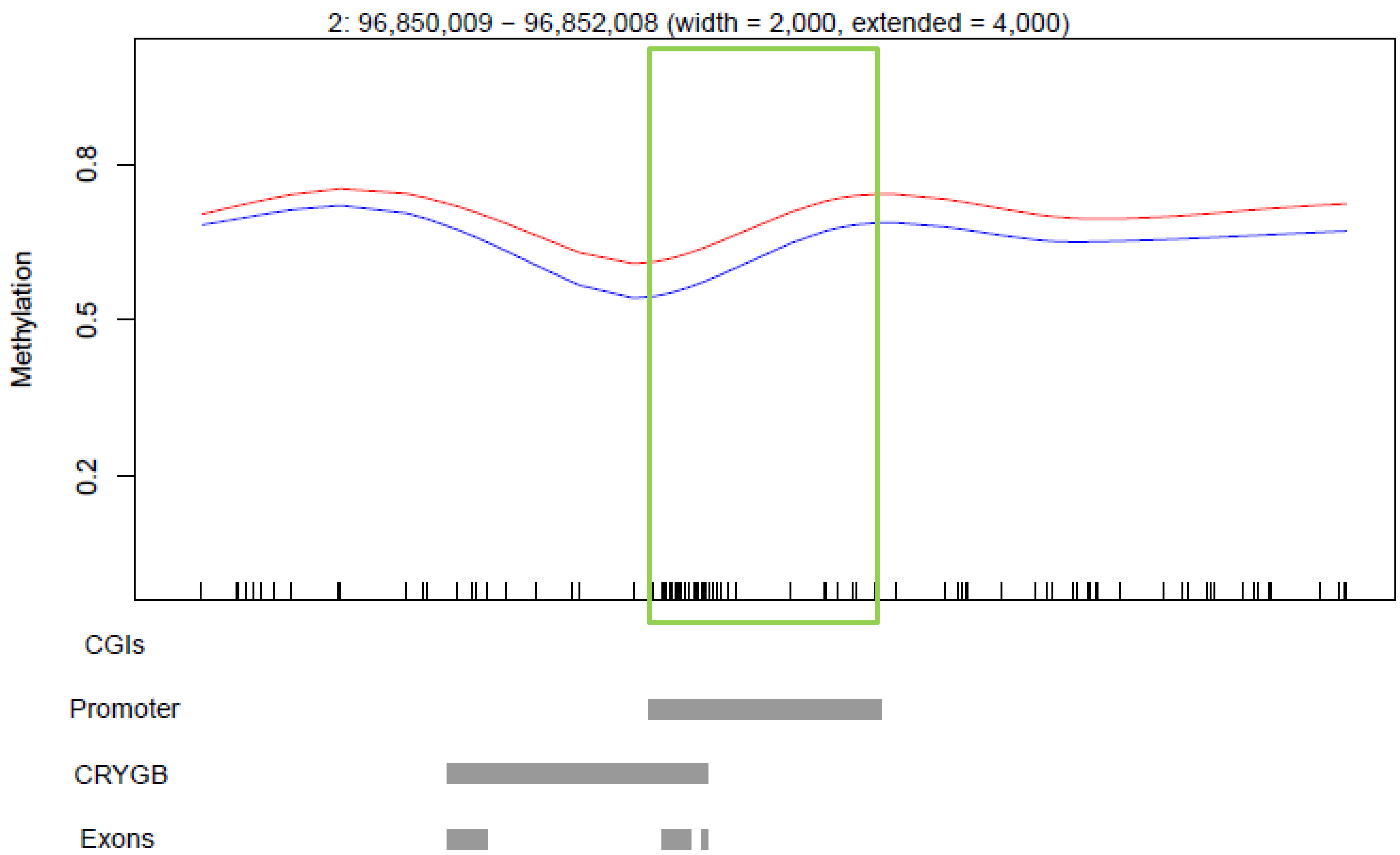

2: 96,850,009 − 96,852,008 (width = 2,000, extended = 4,000)
Methylation
0.8
0.5
0.2
CGIs
Promoter
CRYGB
Exons

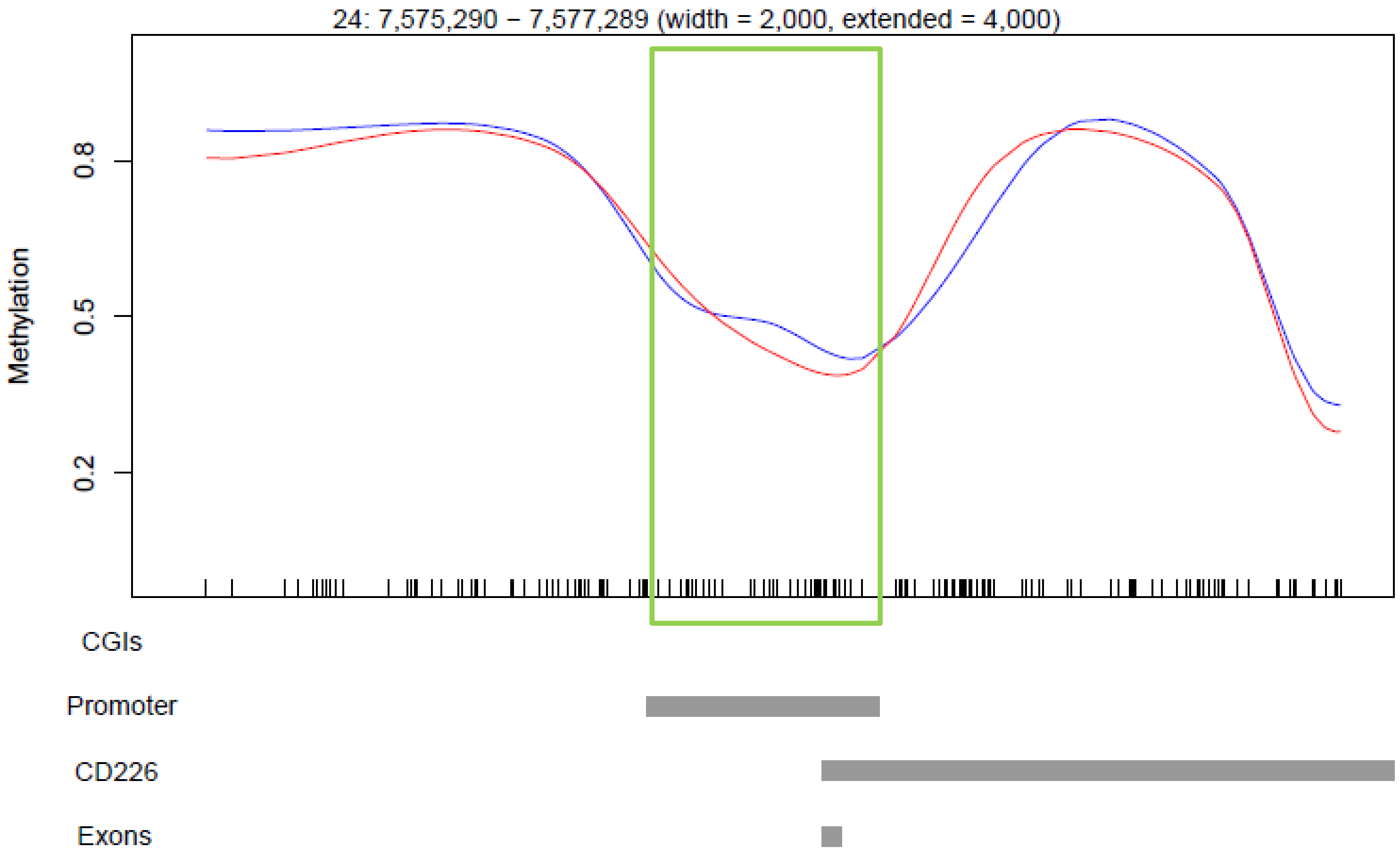
24: 7,575,290 − 7,577,289 (width = 2,000, extended = 4,000)
Methylation
0.8
0.5
0.2
CGIs
Promoter
CD226
Exons

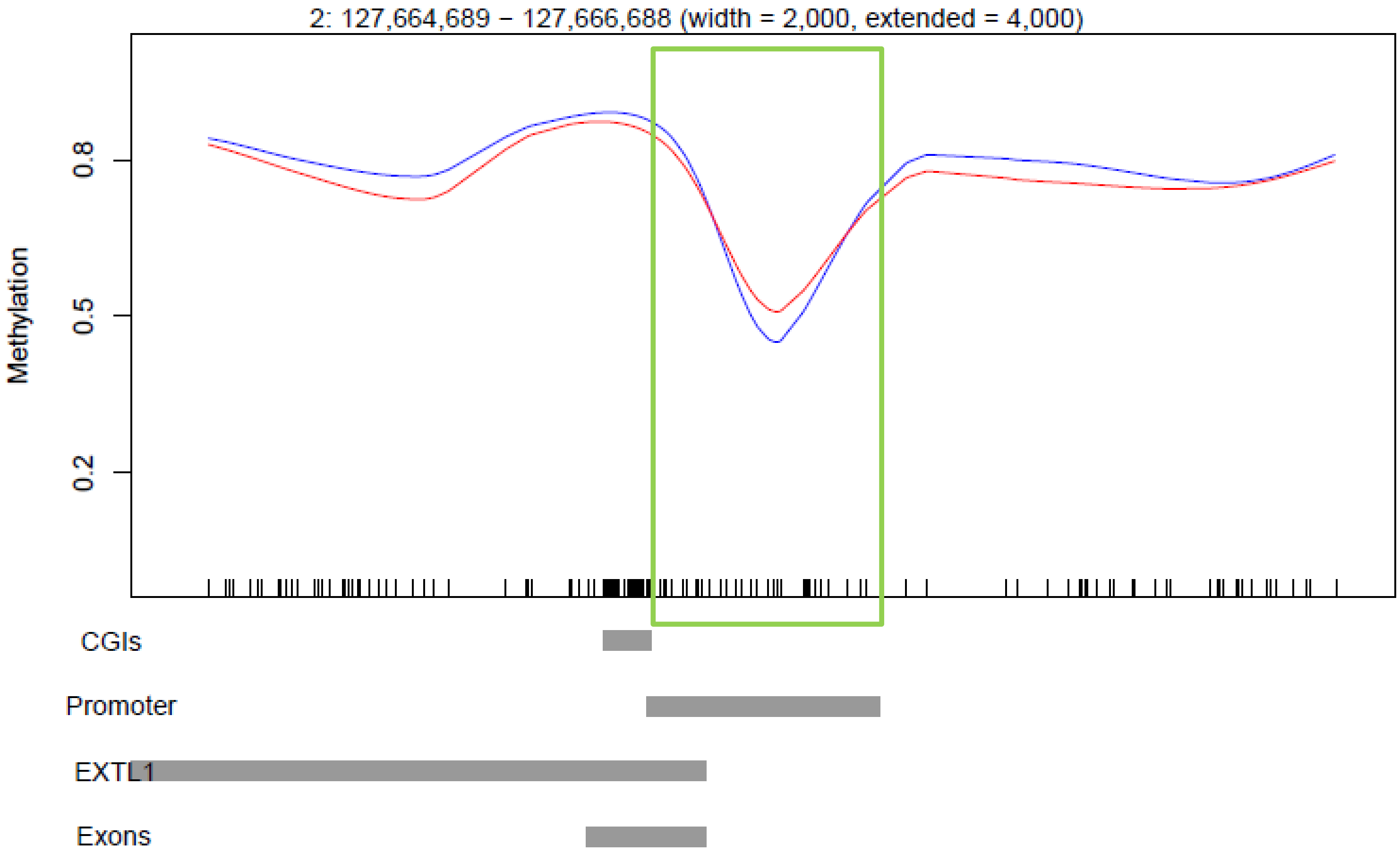

2: 127,664,689 − 127,666,688 (width = 2,000, extended = 4,000)
Methylation
0.8
0.5
0.2
CGIs
Promoter
EXTL1
Exons

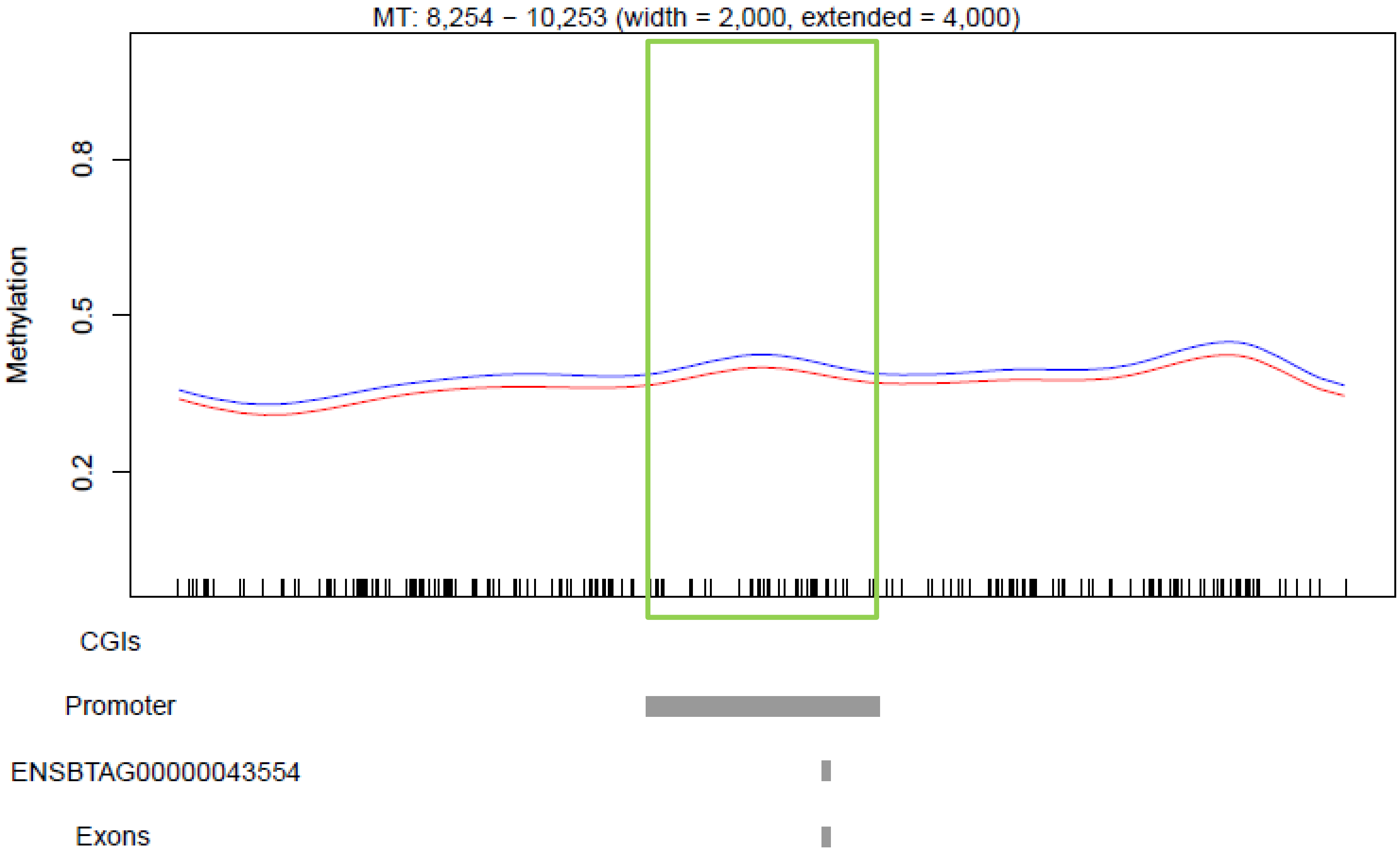
MT: 8,254 − 10,253 (width = 2,000, extended = 4,000)
Methylation
0.8
0.5
0.2
CGIs
Promoter
ENSBTAG00000043554
Exons

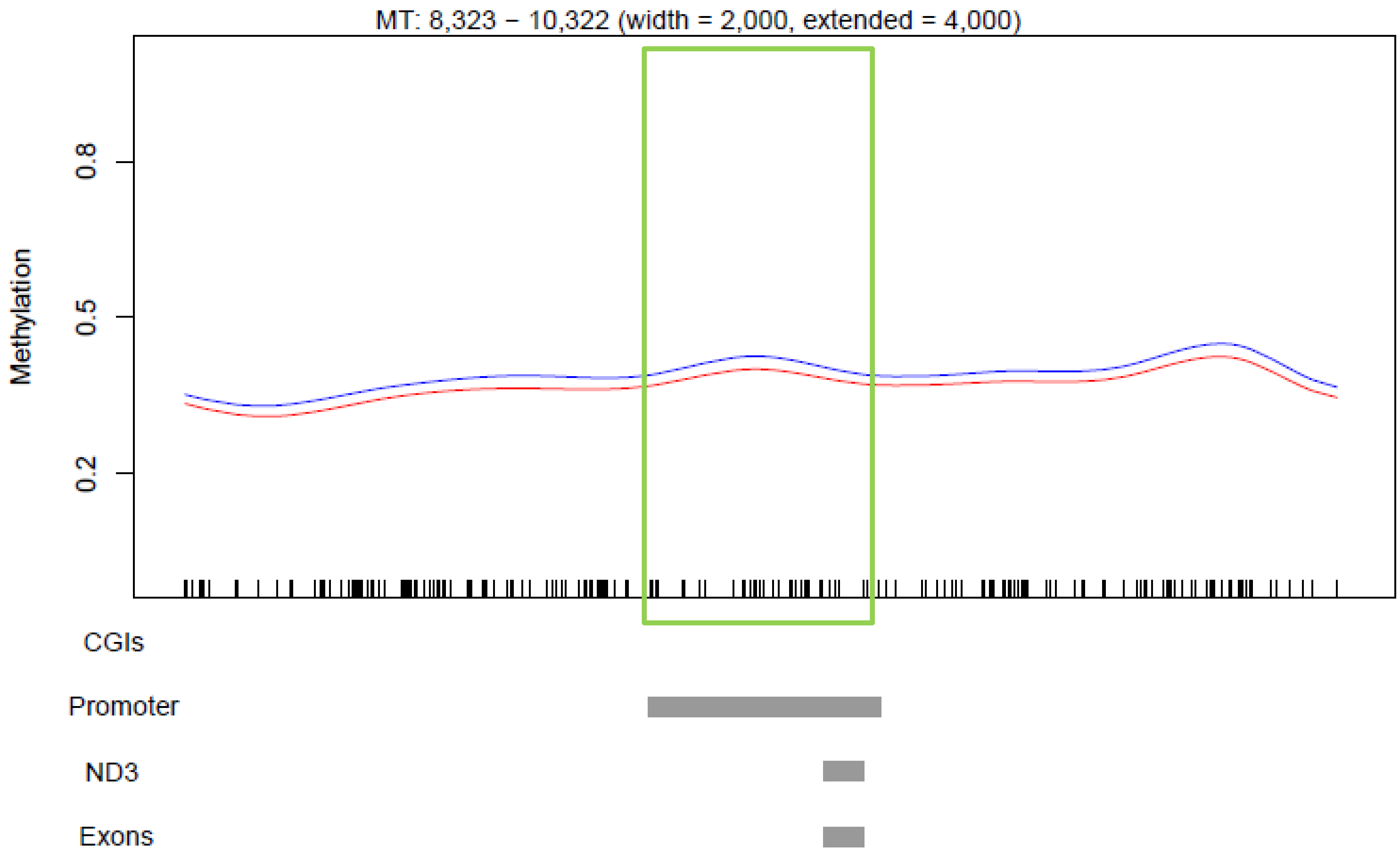
MT: 8,323 − 10,322 (width = 2,000, extended = 4,000)
Methylation
0.8
0.5
0.2
CGIs
Promoter
ND3
Exons

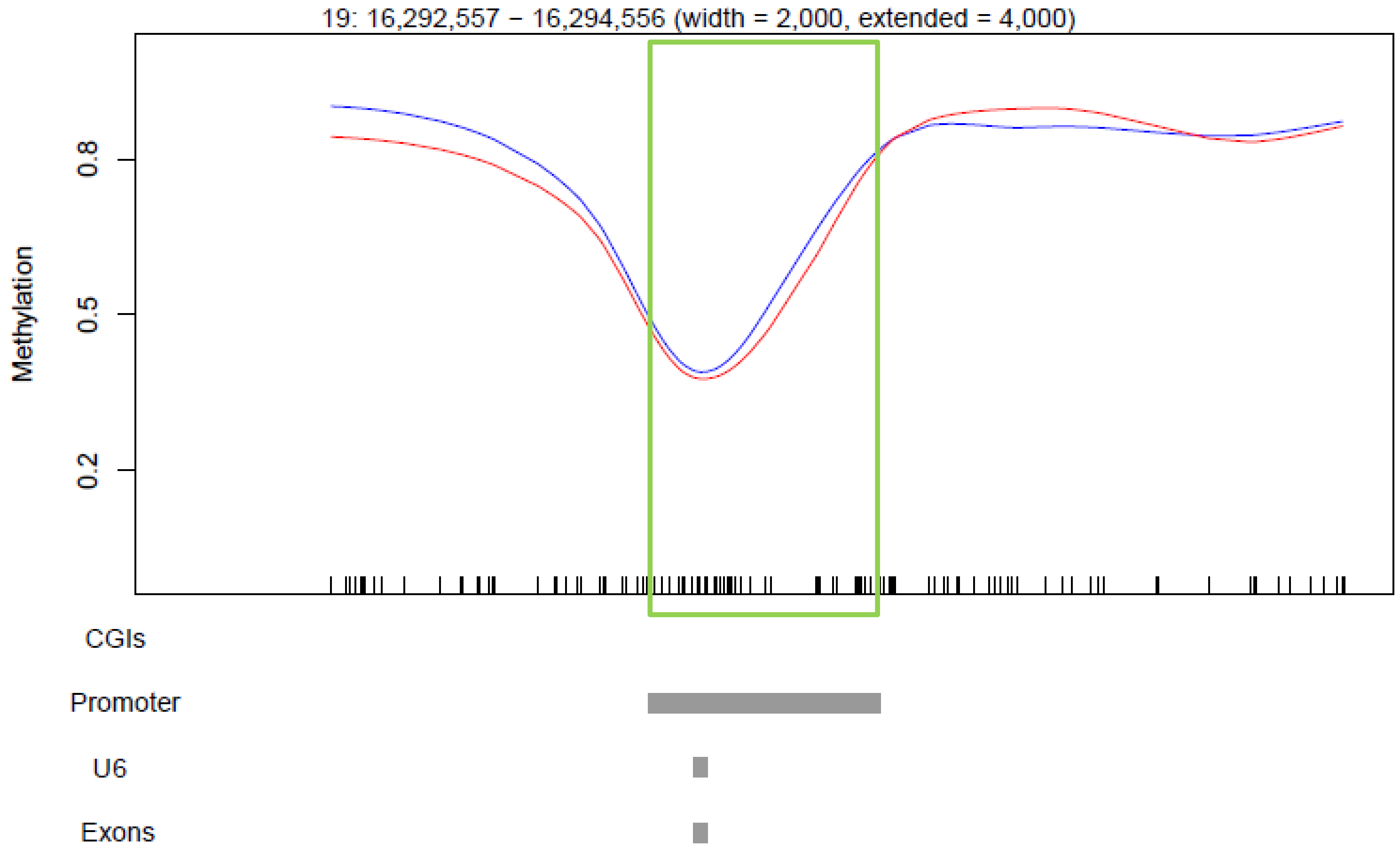

19: 16,292,557 − 16,294,556 (width = 2,000, extended = 4,000)
Methylation
0.8
0.5
0.2
CGIs
Promoter
U6
Exons

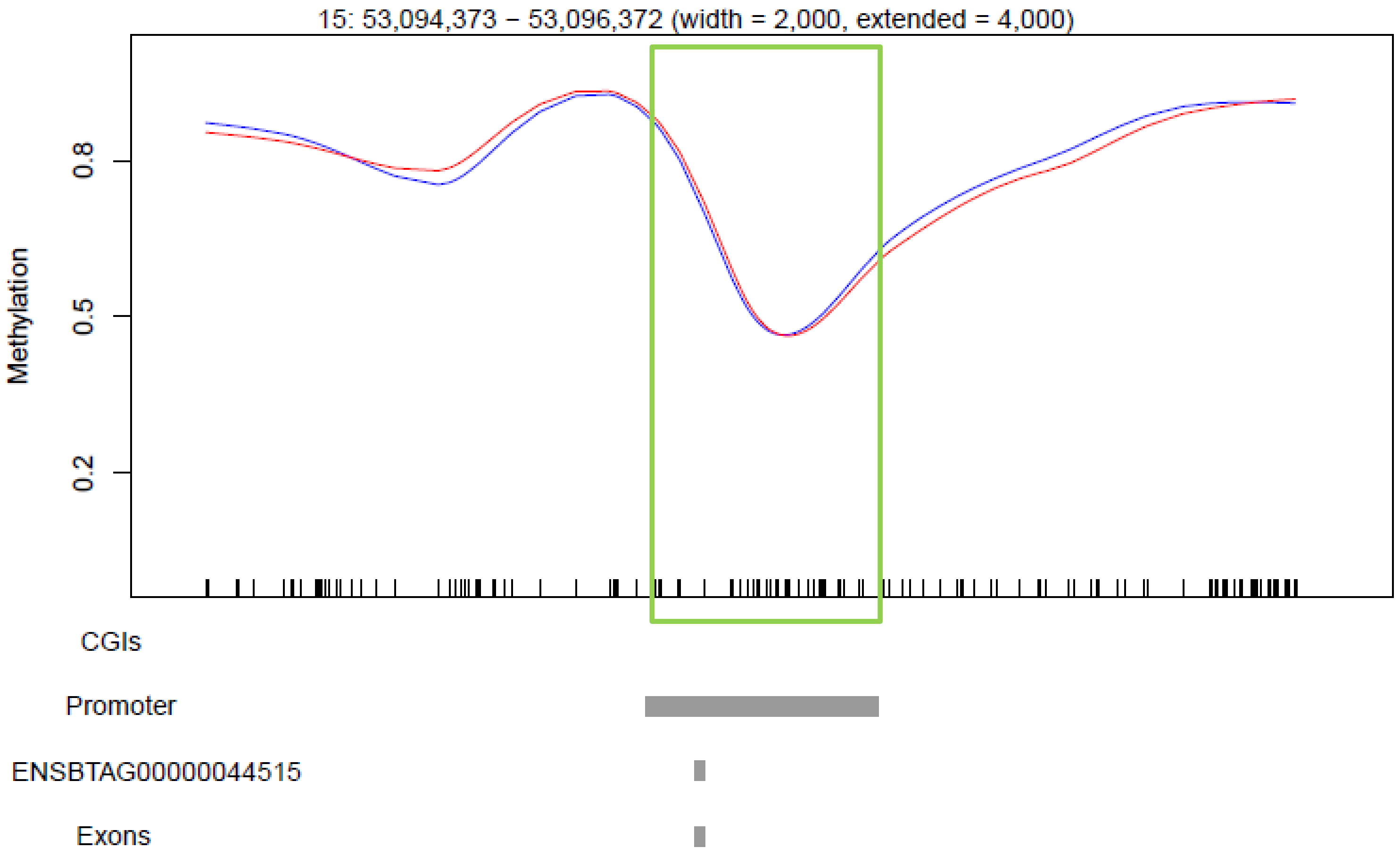

15: 53,094,373 − 53,096,372 (width = 2,000, extended = 4,000)
Methylation
0.8
0.5
0.2
CGIs
Promoter
ENSBTAG00000044515
Exons

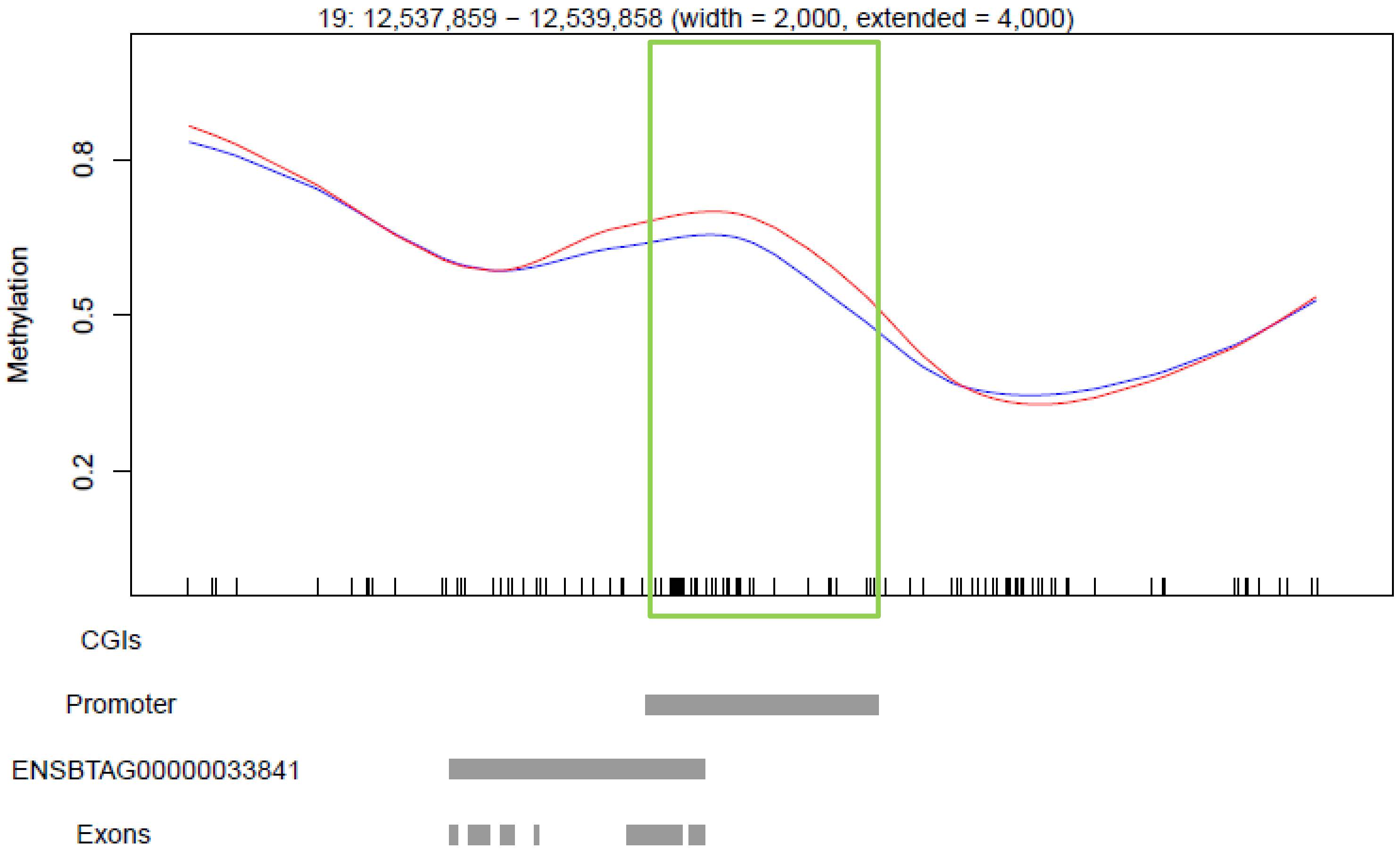

19: 12,537,859 − 12,539,858 (width = 2,000, extended = 4,000)
Methylation
0.8
0.5
0.2
CGIs
Promoter
ENSBTAG00000033841
Exons

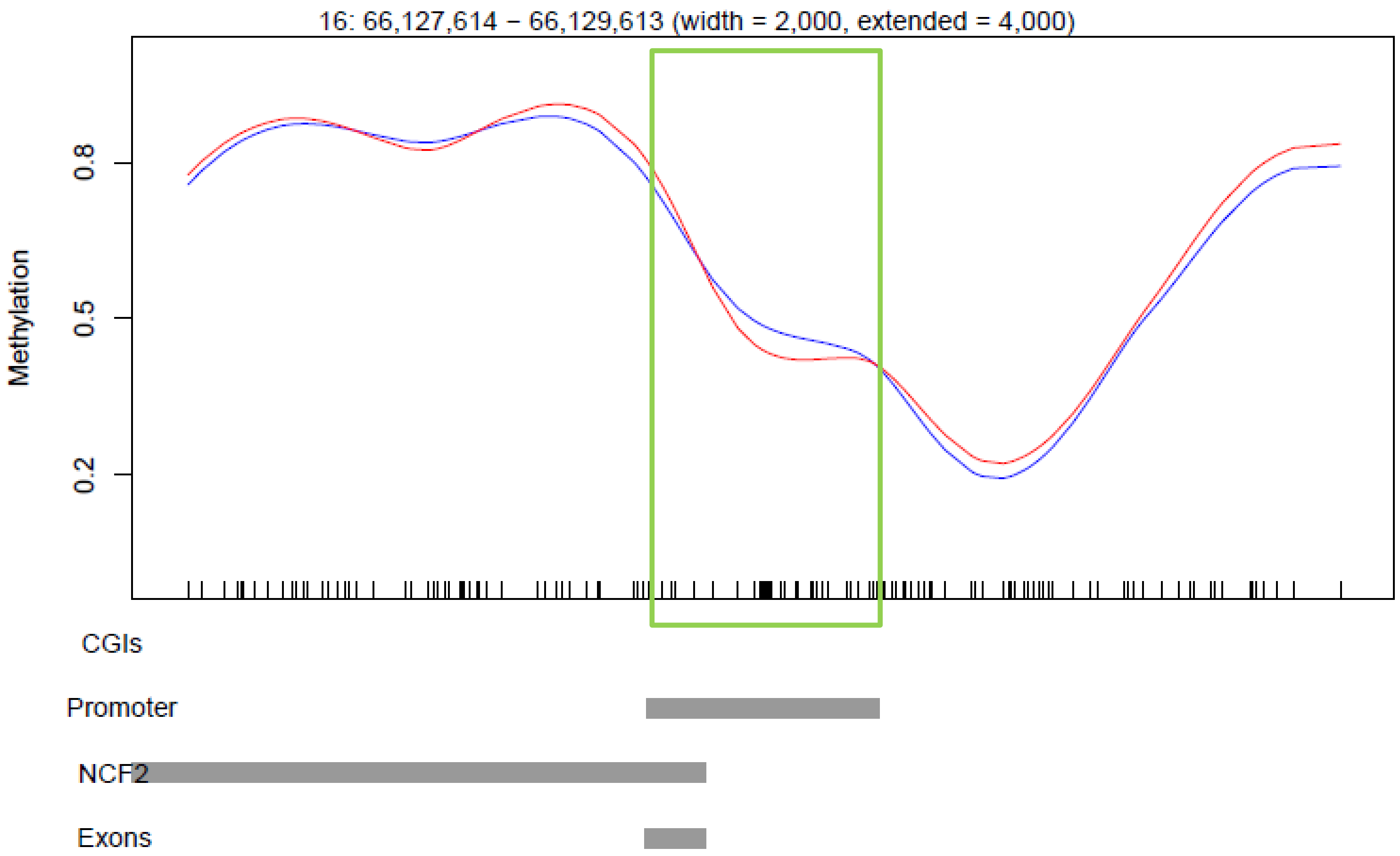

16: 66,127,614 − 66,129,613 (width = 2,000, extended = 4,000)
Methylation
0.8
0.5
0.2
CGIs
Promoter
NCF2
Exons

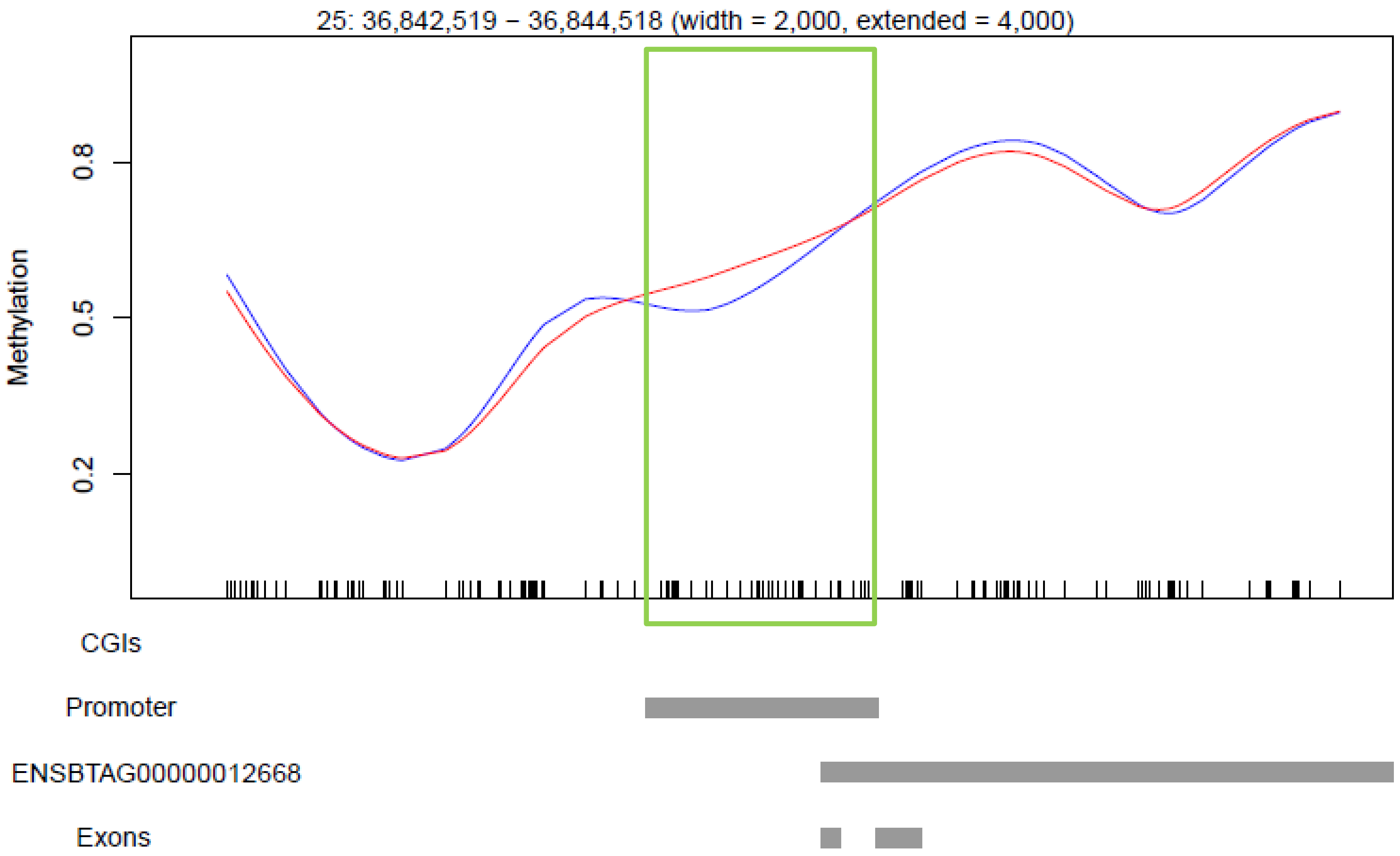
25: 36,842,519 − 36,844,518 (width = 2,000, extended = 4,000)
Methylation
0.8
0.5
0.2
CGIs
Promoter
ENSBTAG00000012668
Exons

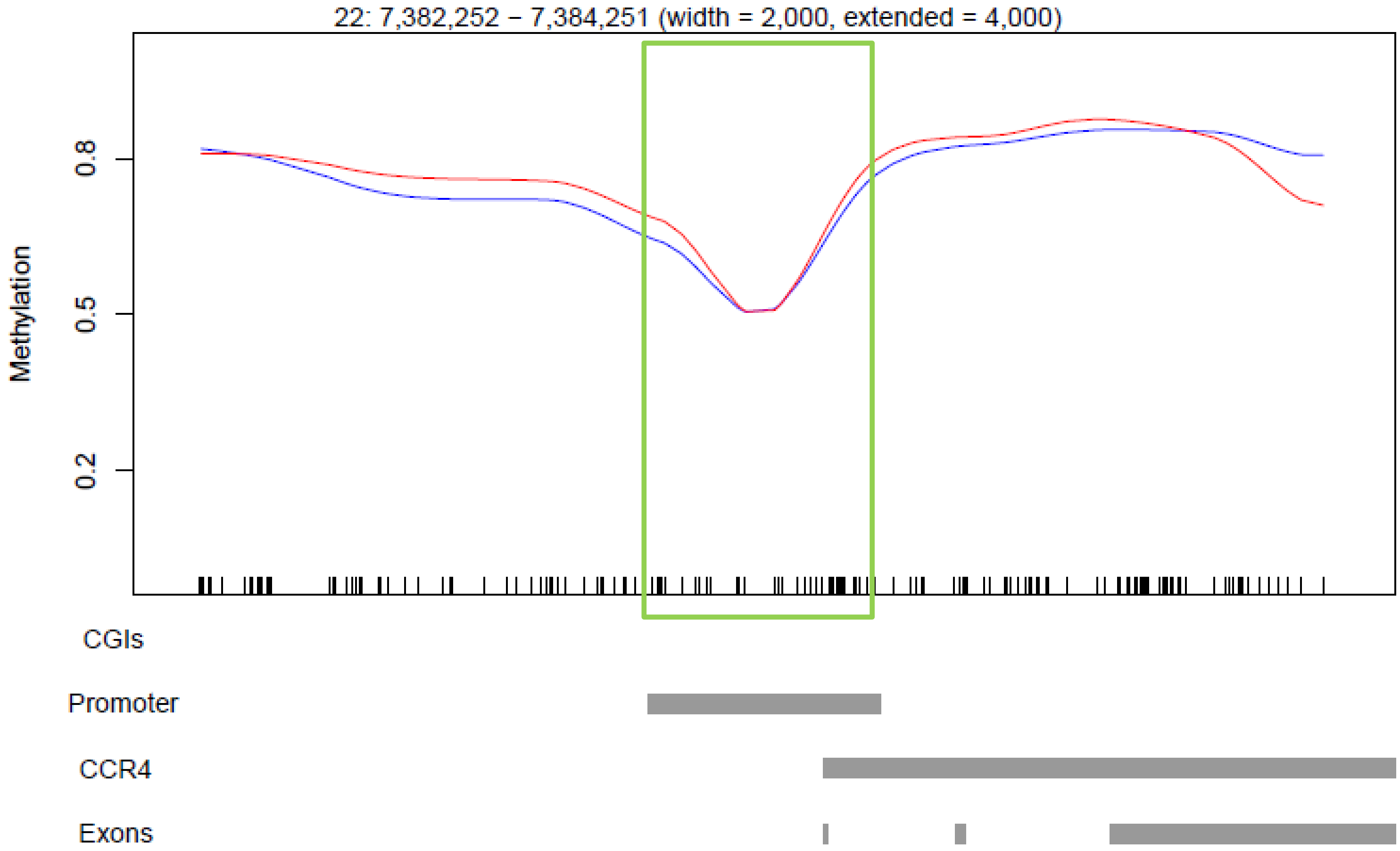

22: 7,382,252 – 7,384,251 (width = 2,000, extended = 4,000)
Methylation
0.8
0.5
0.2
CGIs
Promoter
CCR4
Exons

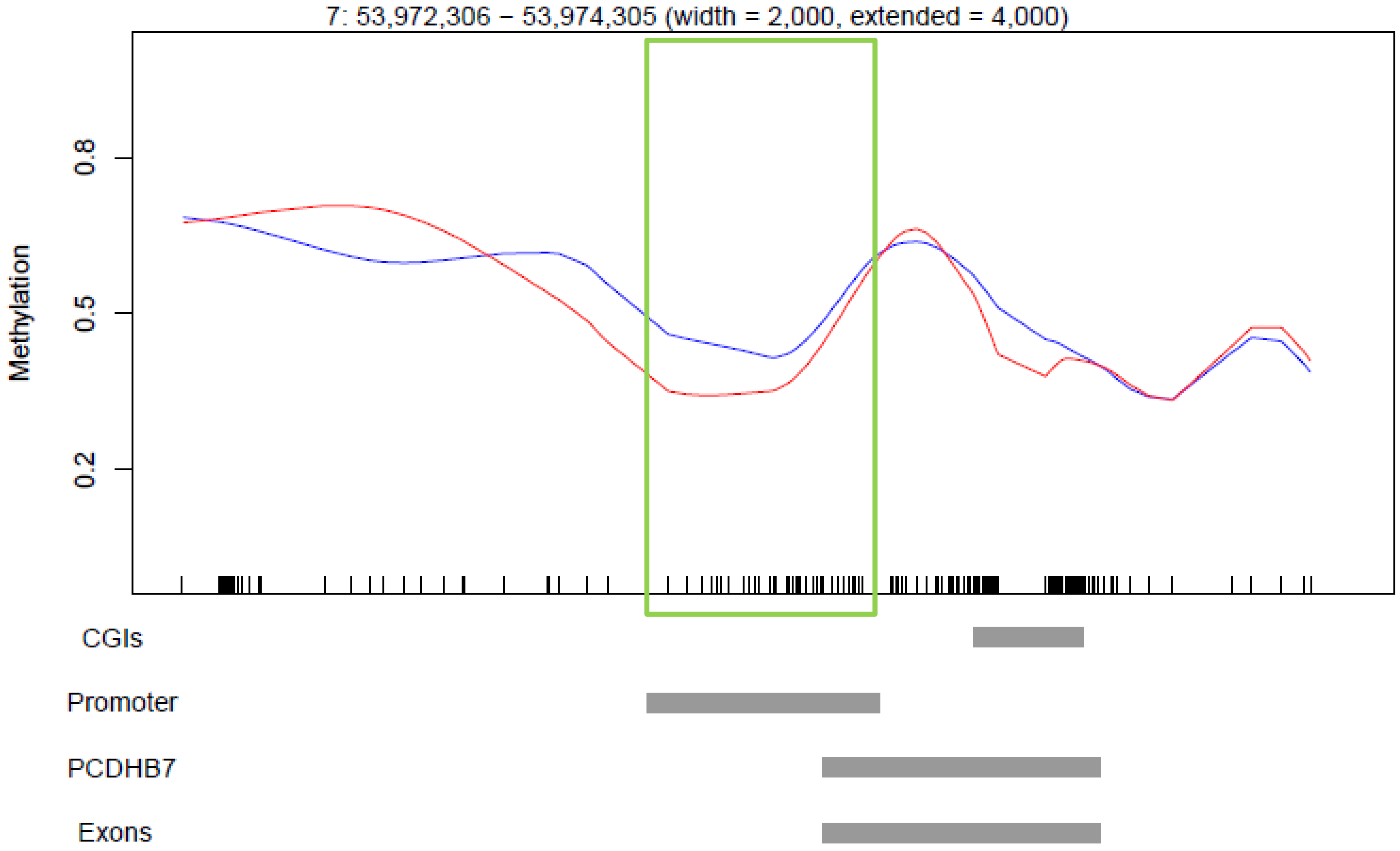
7: 53,972,306 – 53,974,305 (width = 2,000, extended = 4,000)
Methylation
0.8
0.5
0.2
CGIs
Promoter
PCDHB7
Exons

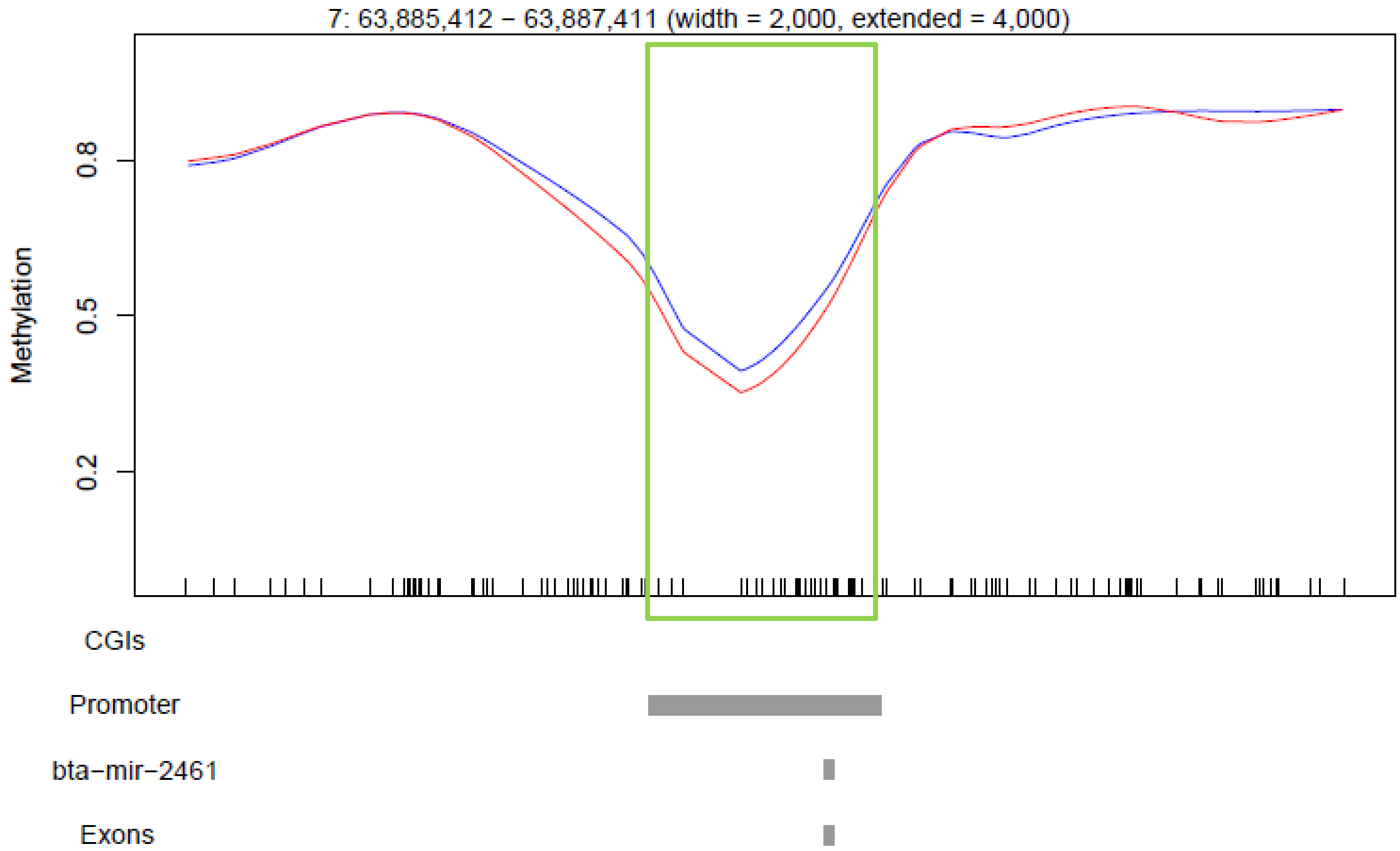
7: 63,885,412 − 63,887,411 (width = 2,000, extended = 4,000)
Methylation
0.8
0.5
0.2
CGIs
Promoter
bta−mir−2461
Exons

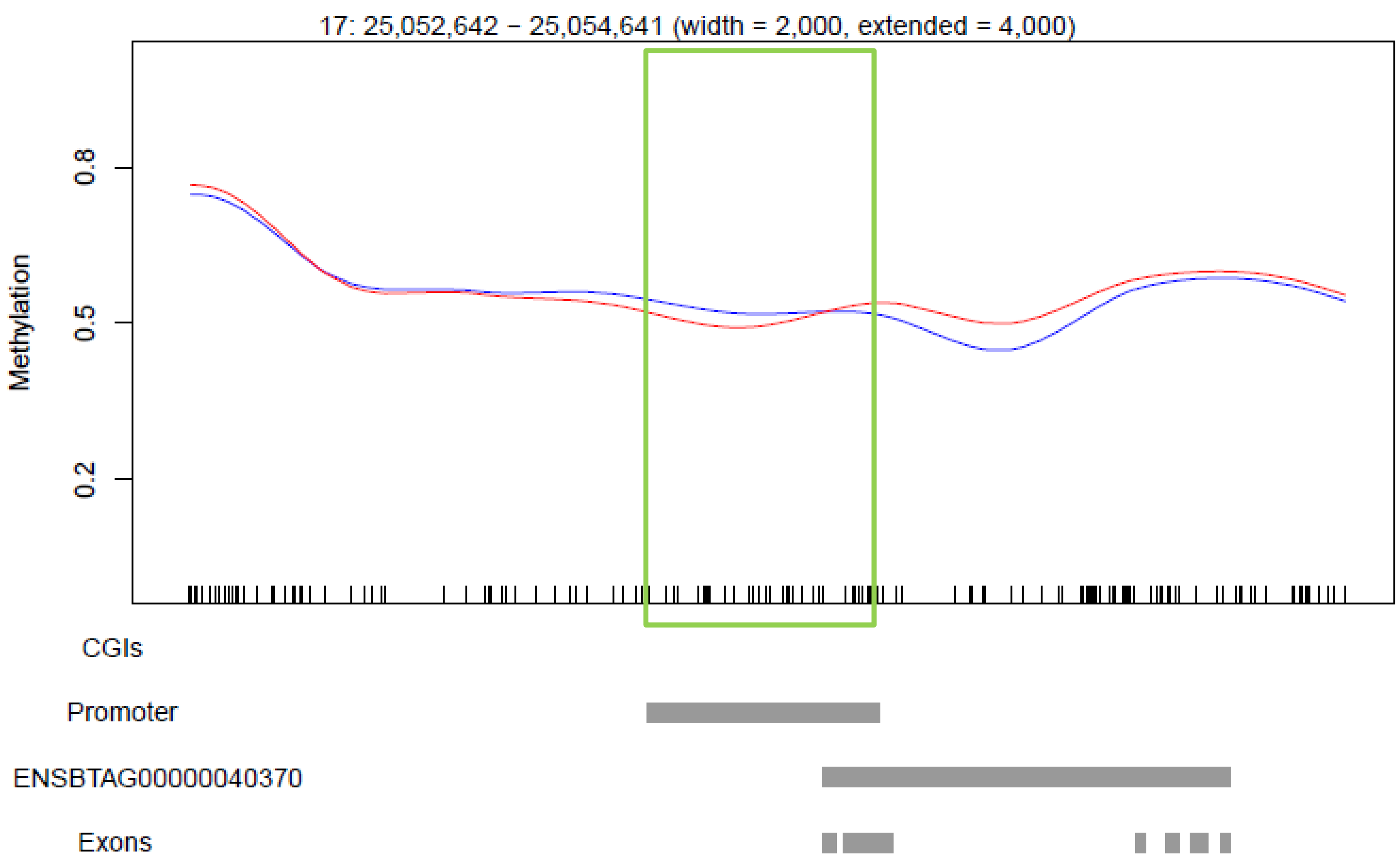
17: 25,052,642 − 25,054,641 (width = 2,000, extended = 4,000)
Methylation
0.2
0.5
0.8
CGIs
Promoter
ENSBTAG00000040370
Exons

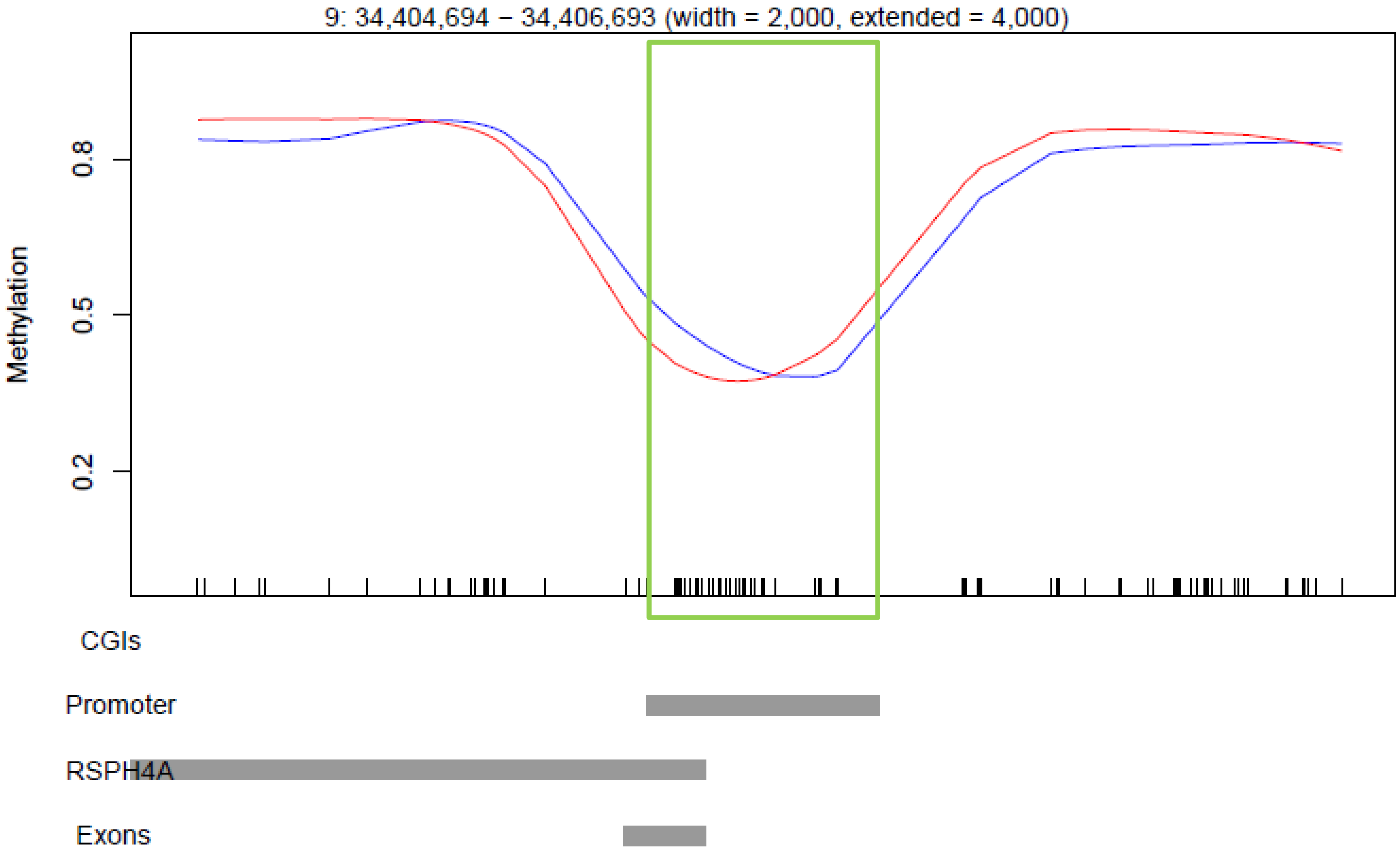
9: 34,404,694 − 34,406,693 (width = 2,000, extended = 4,000)
Methylation
0.8
0.5
0.2
CGIs
Promoter
RSPH4A
Exons

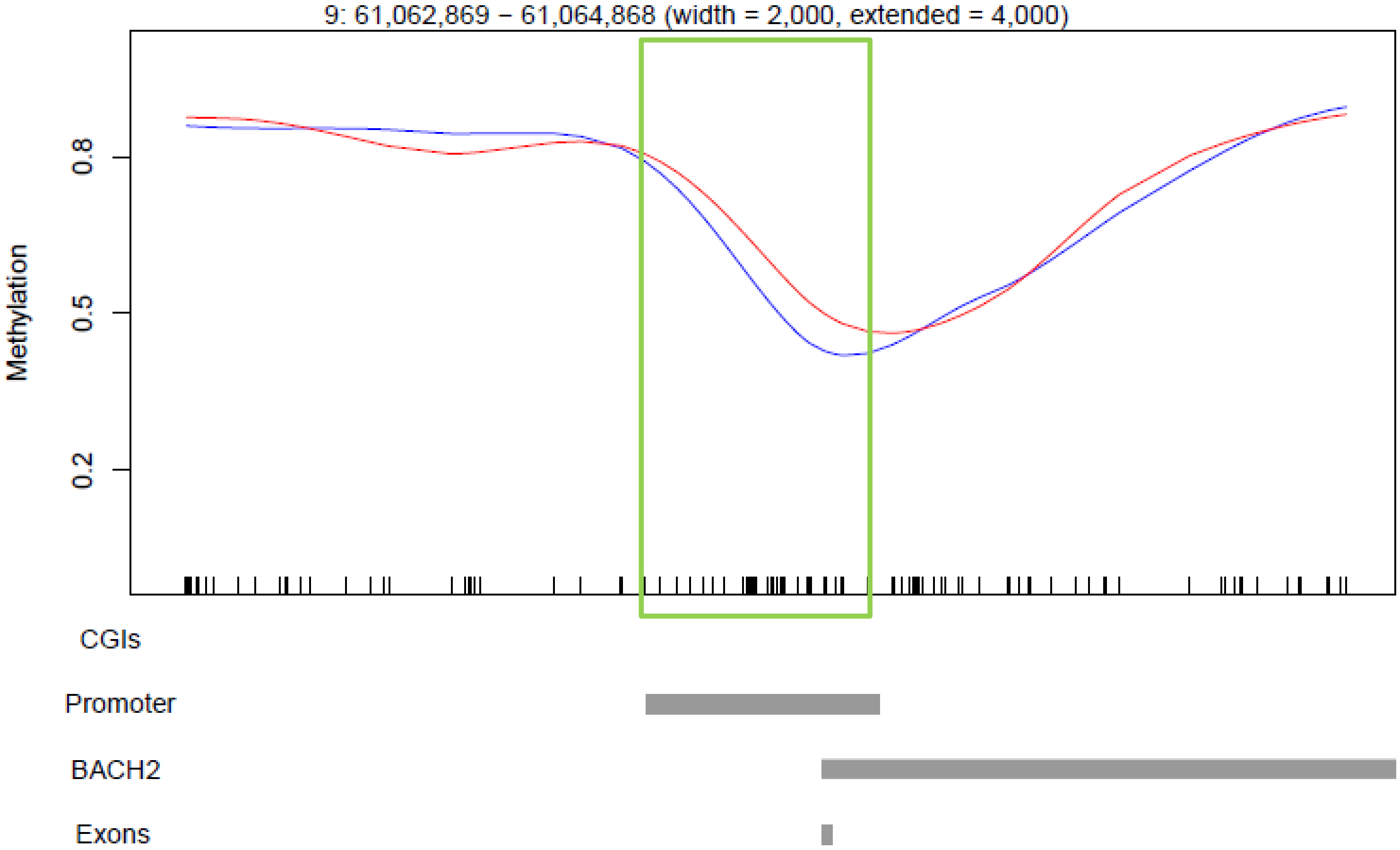

9: 61,062,869 − 61,064,868 (width = 2,000, extended = 4,000)
Methylation
0.8
0.5
0.2
CGIs
Promoter
BACH2
Exons

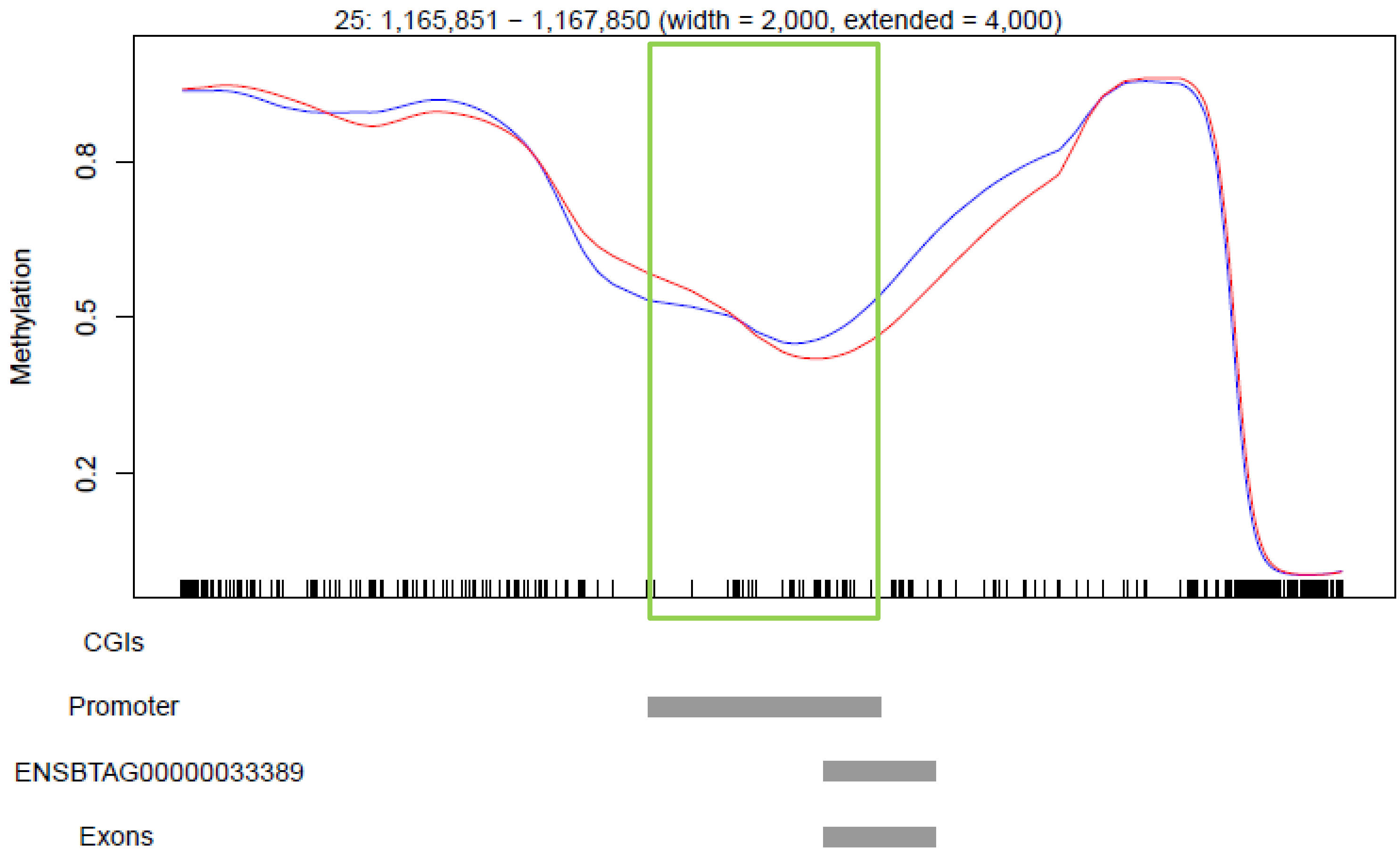
25: 1,165,851 – 1,167,850 (width = 2,000, extended = 4,000)
Methylation
0.8
0.5
0.2
CGIs
Promoter
ENSBTAG00000033389
Exons

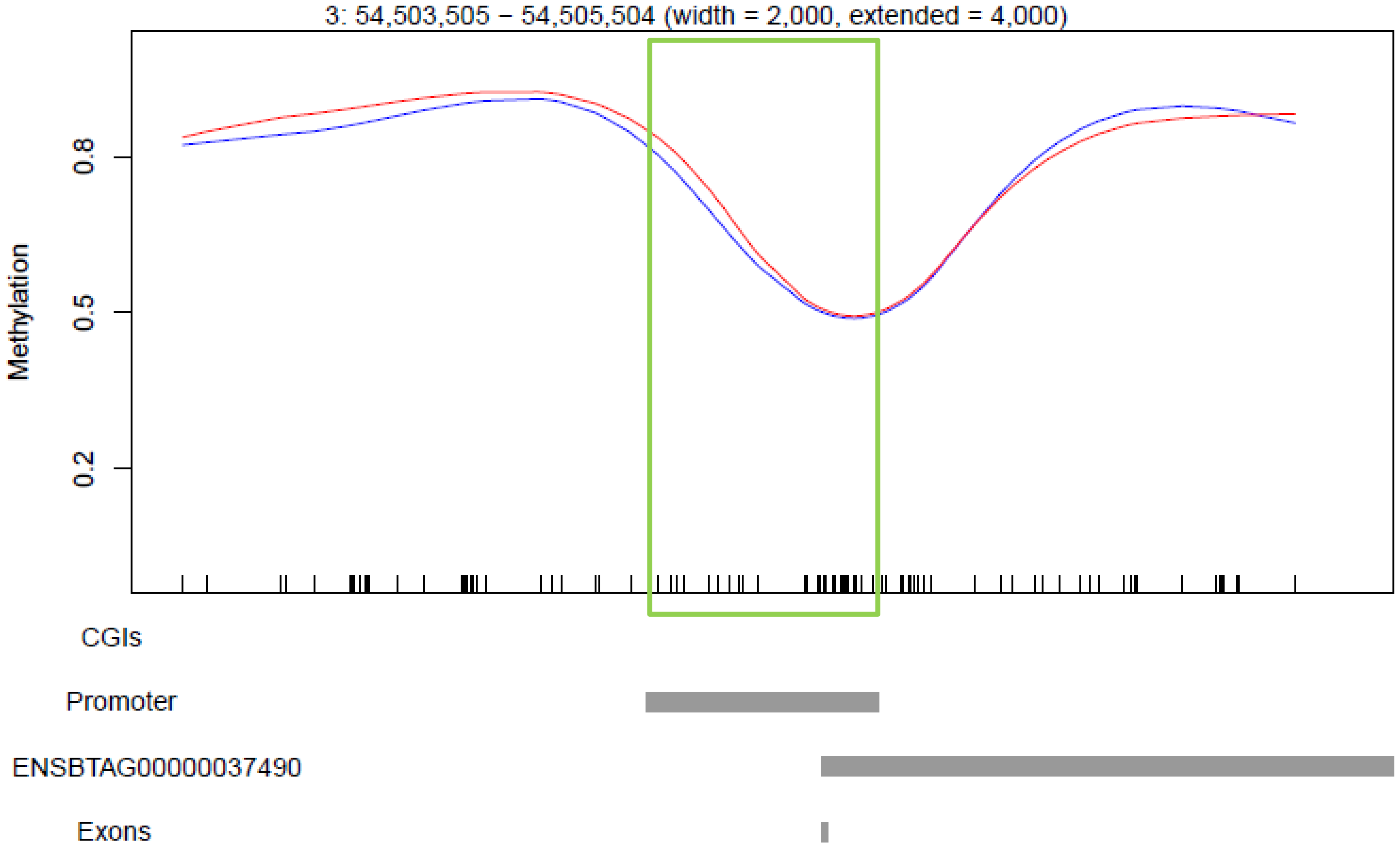

3: 54,503,505 – 54,505,504 (width = 2,000, extended = 4,000)
Methylation
0.8
0.5
0.2
CGIs
Promoter
ENSBTAG00000037490
Exons

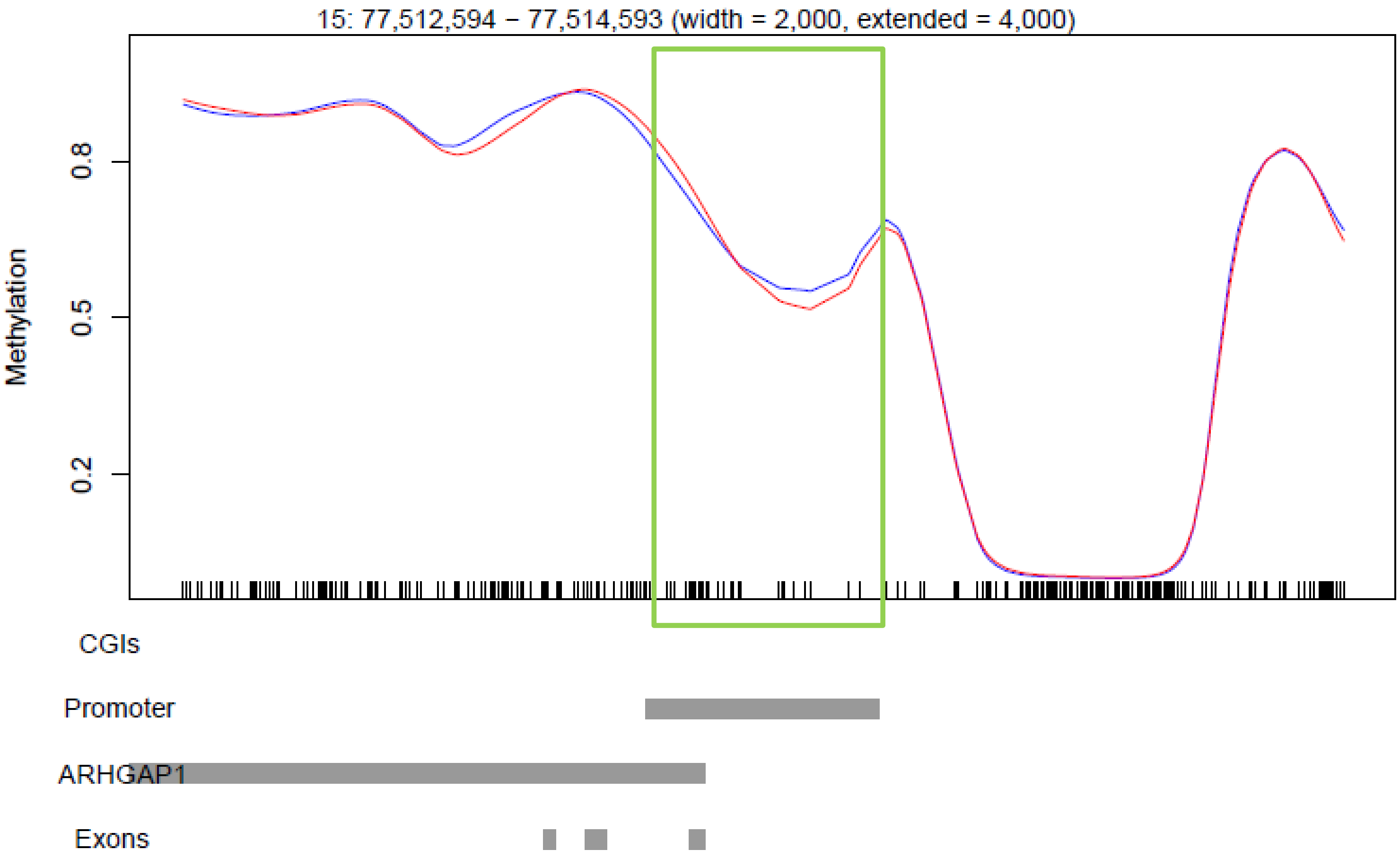
15: 77,512,594 − 77,514,593 (width = 2,000, extended = 4,000)
Methylation
0.8
0.5
0.2
CGIs
Promoter
ARHGAP1
Exons

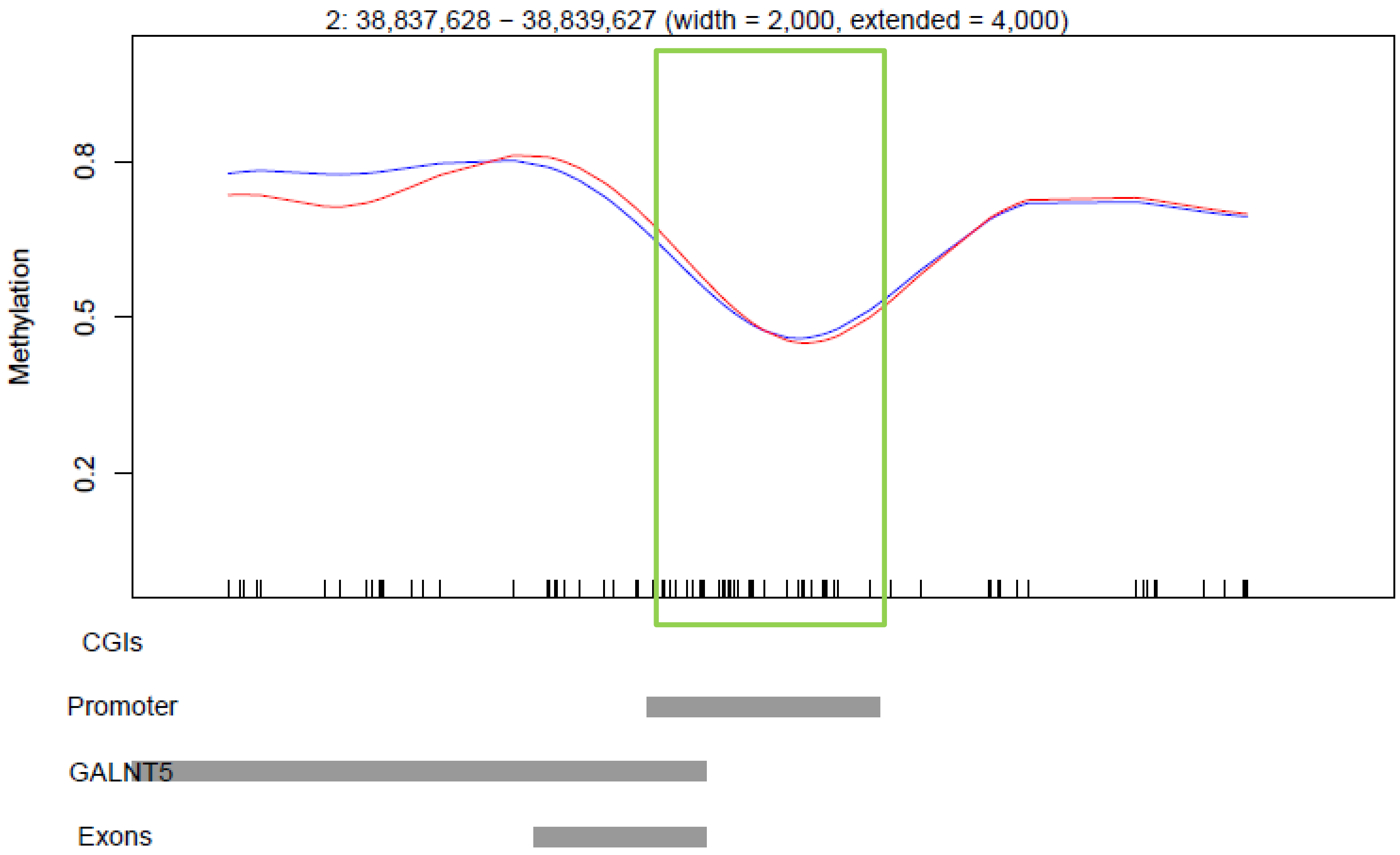
2: 38,837,628 – 38,839,627 (width = 2,000, extended = 4,000)
Methylation
0.8
0.5
0.2
CGIs
Promoter
GALNT5
Exons

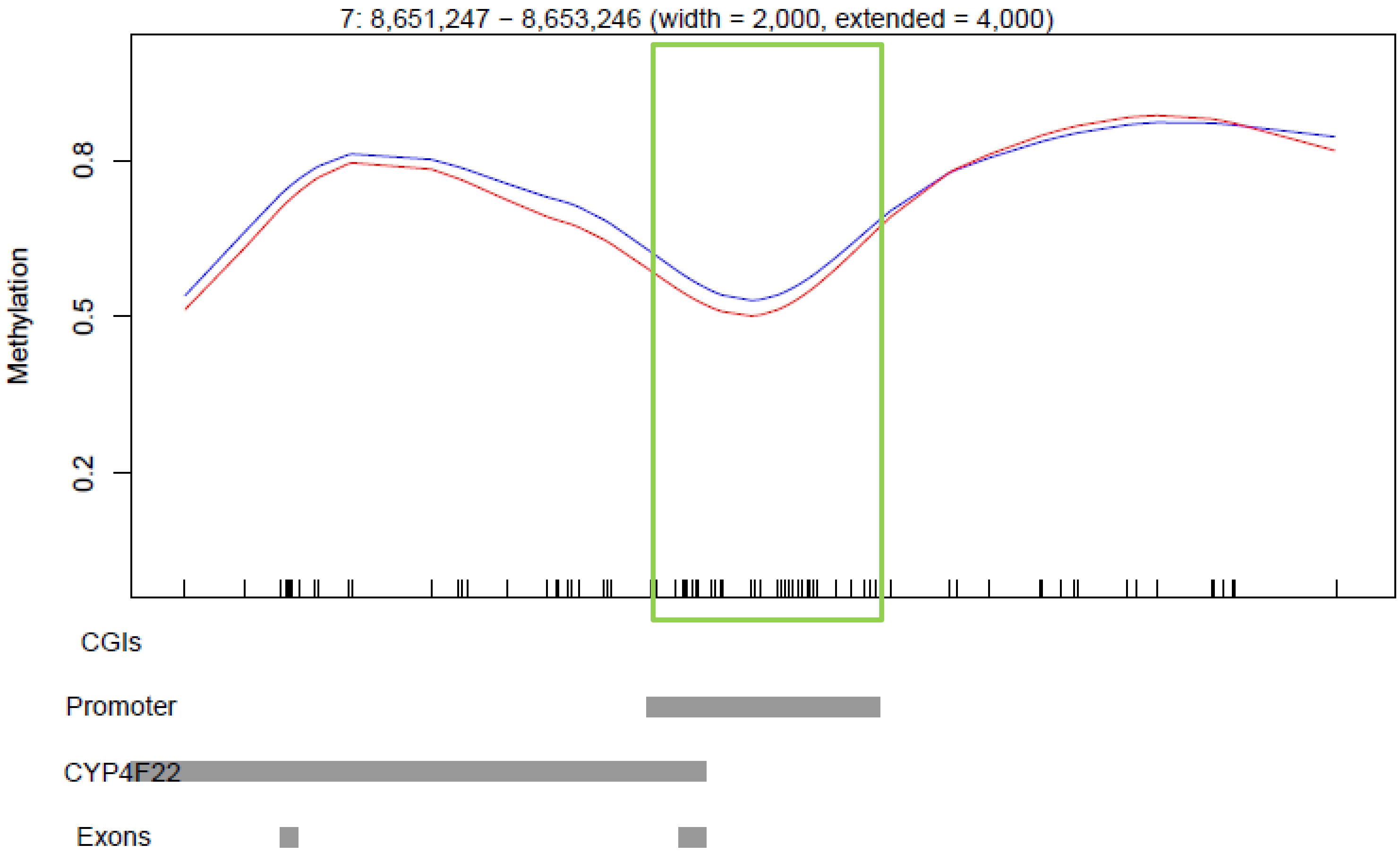
7: 8,651,247 − 8,653,246 (width = 2,000, extended = 4,000)
Methylation
0.8
0.5
0.2
CGIs
Promoter
CYP4F22
Exons

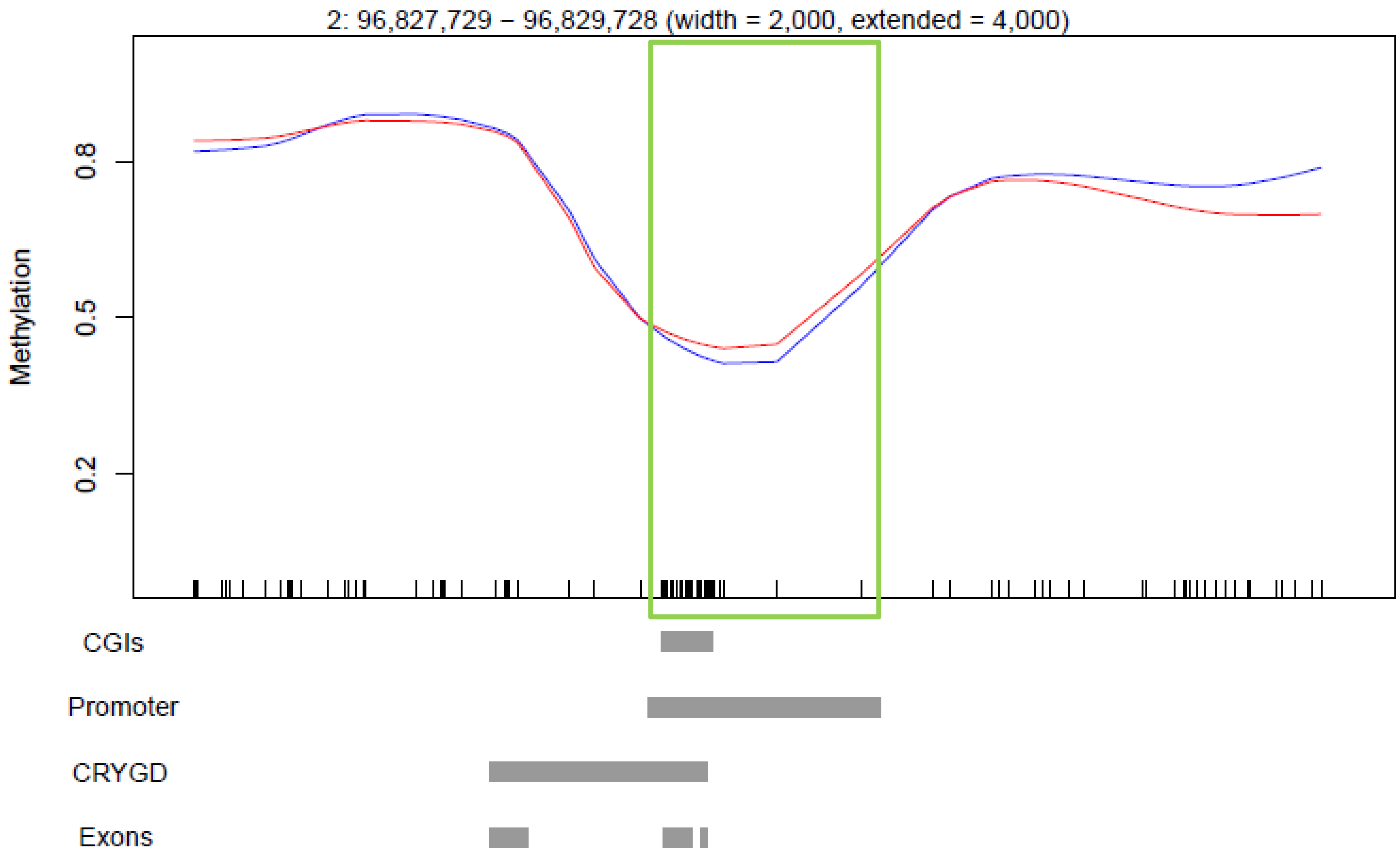
2: 96,827,729 – 96,829,728 (width = 2,000, extended = 4,000)
Methylation
0.8
0.5
0.2
CGIs
Promoter
CRYGD
Exons

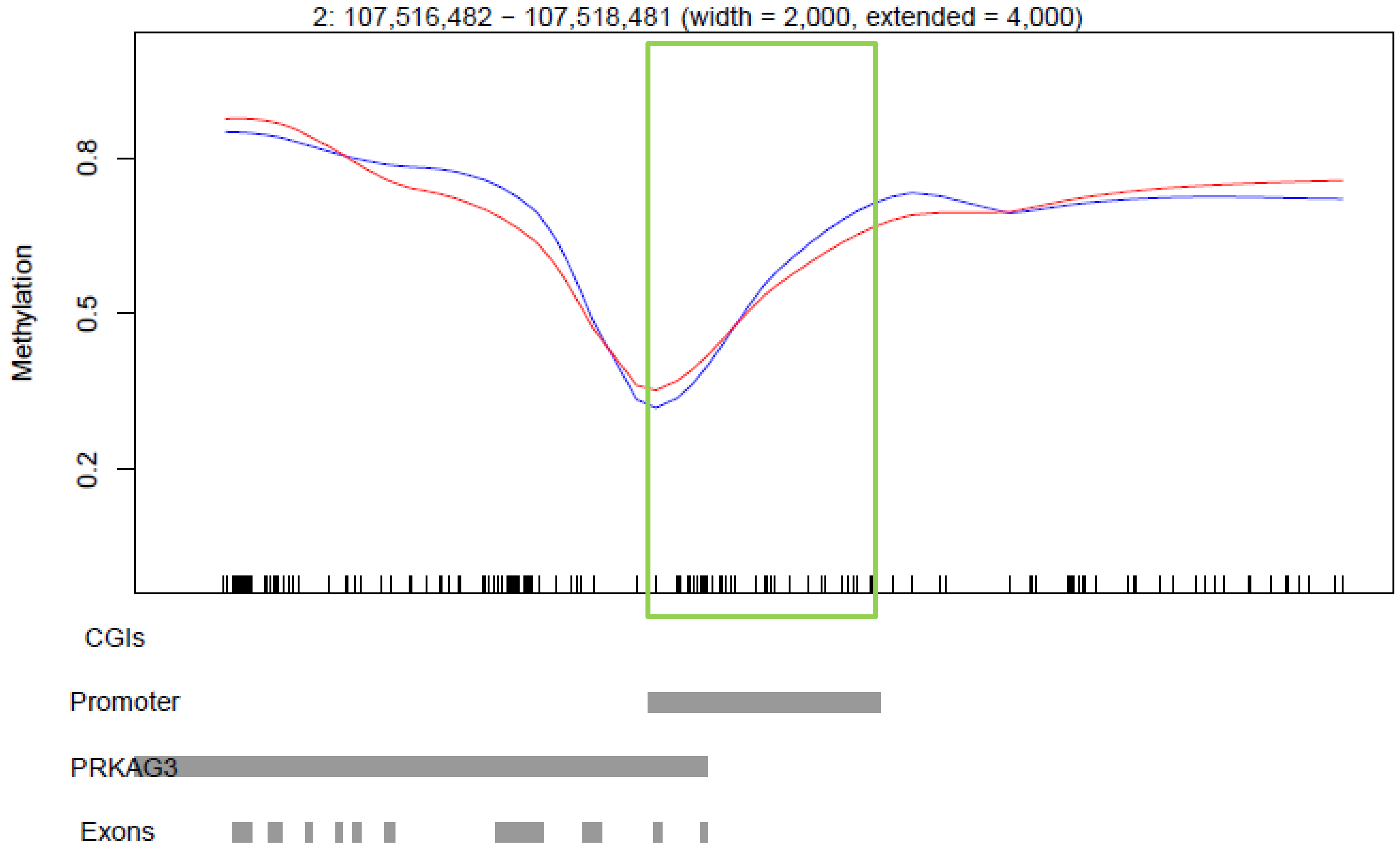

2: 107,516,482 − 107,518,481 (width = 2,000, extended = 4,000)
Methylation
0.8
0.5
0.2
CGIs
Promoter
PRKAG3
Exons

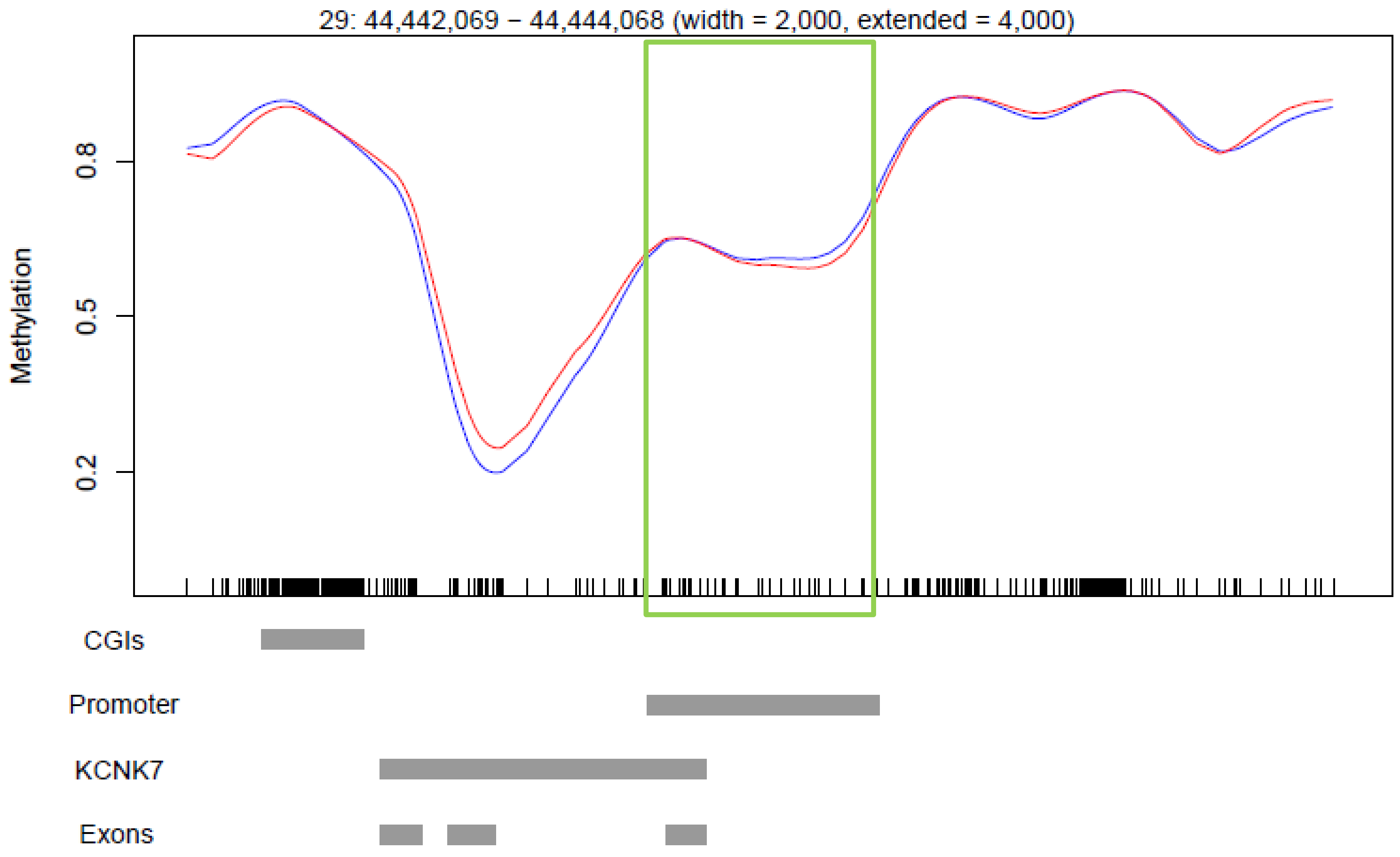

29: 44,442,069 – 44,444,068 (width = 2,000, extended = 4,000)
Methylation
0.2
0.5
0.8
CGIs
Promoter
KCNK7
Exons

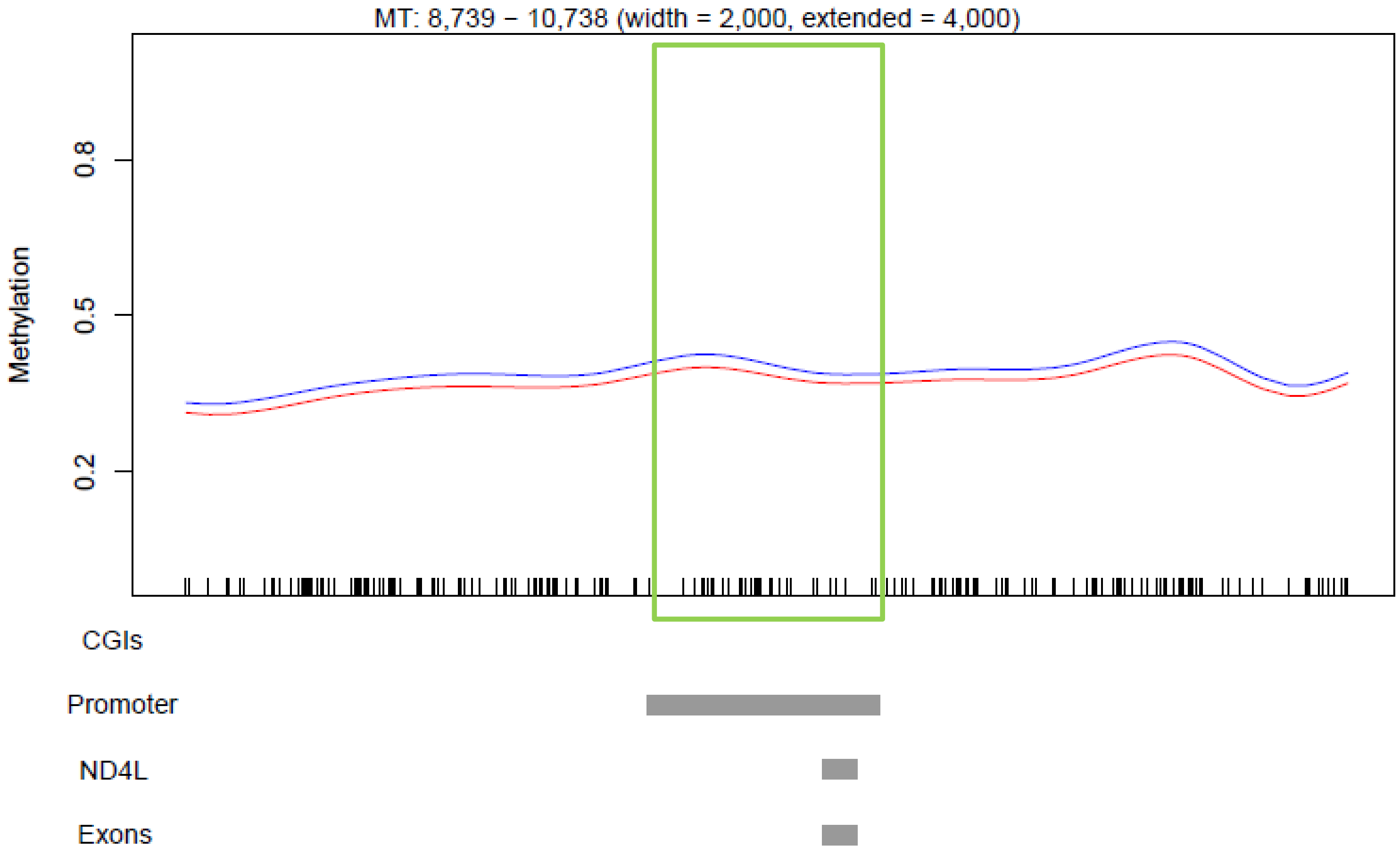
MT: 8,739 – 10,738 (width = 2,000, extended = 4,000)
Methylation
0.8
0.5
0.2
CGIs
Promoter
ND4L
Exons

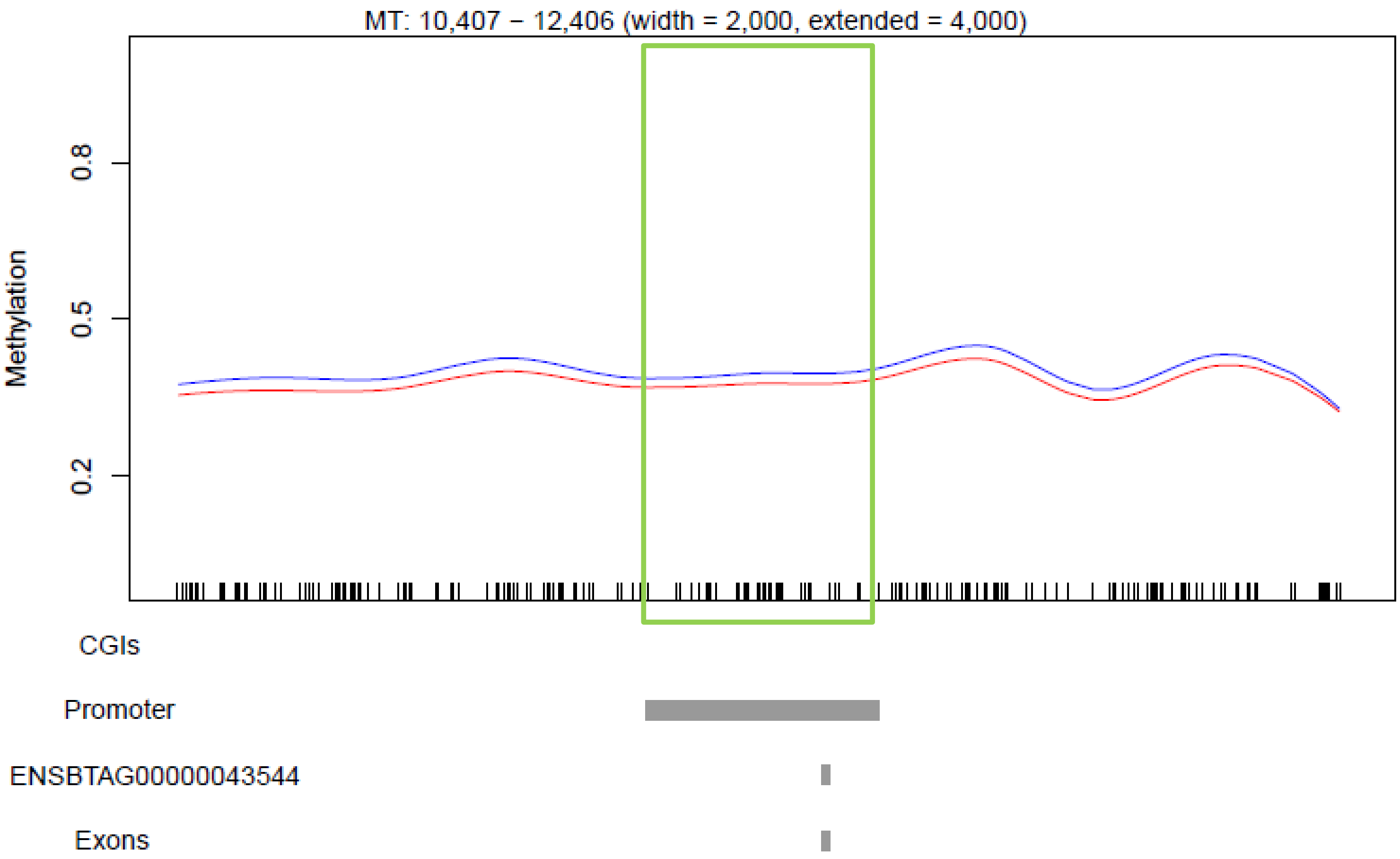
MT: 10,407 − 12,406 (width = 2,000, extended = 4,000)
Methylation
0.8
0.5
0.2
CGIs
Promoter
ENSBTAG00000043544
Exons

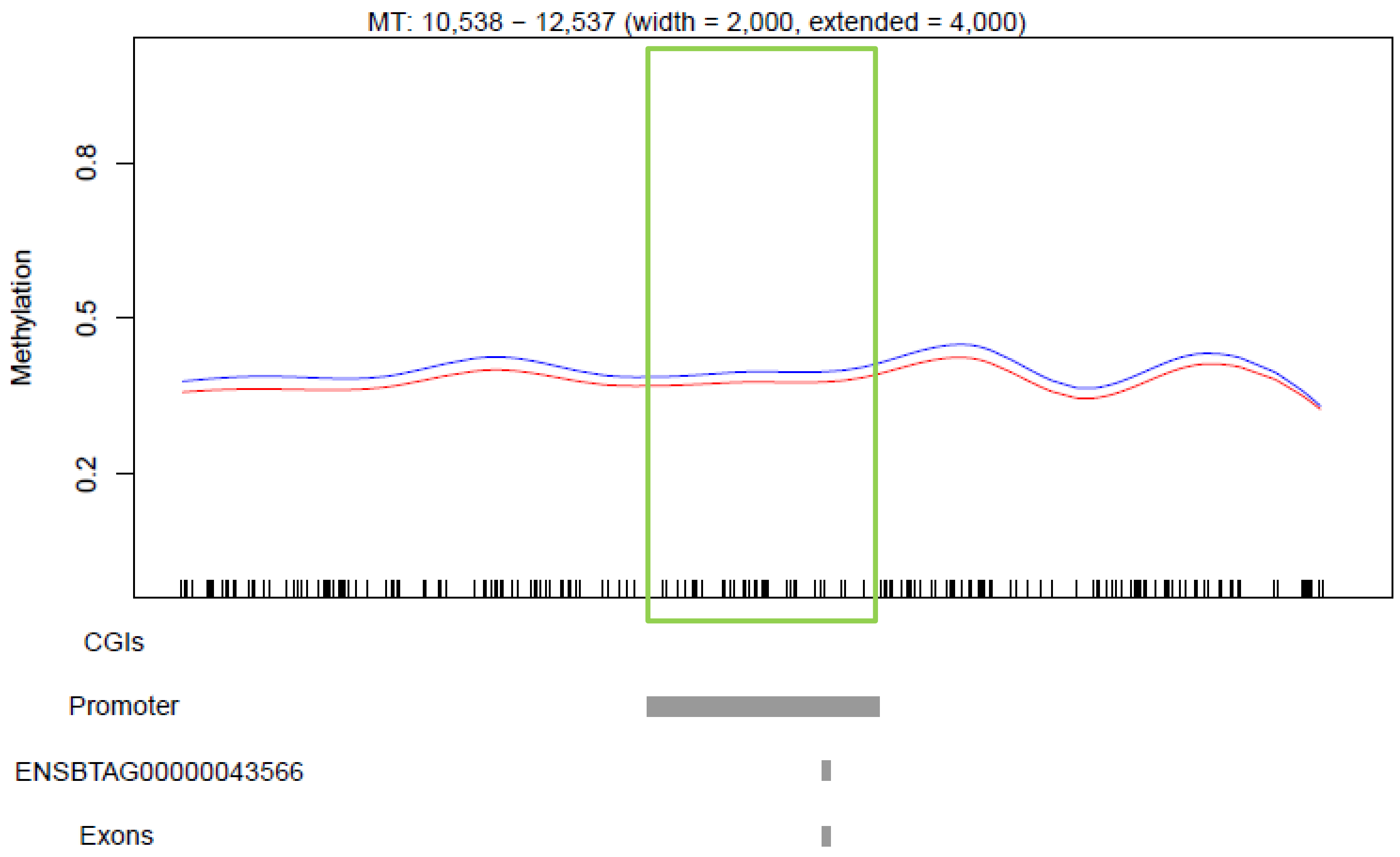

MT: 10,538 – 12,537 (width = 2,000, extended = 4,000)
Methylation
0.2
0.5
0.8
CGIs
Promoter
ENSBTAG00000043566
Exons

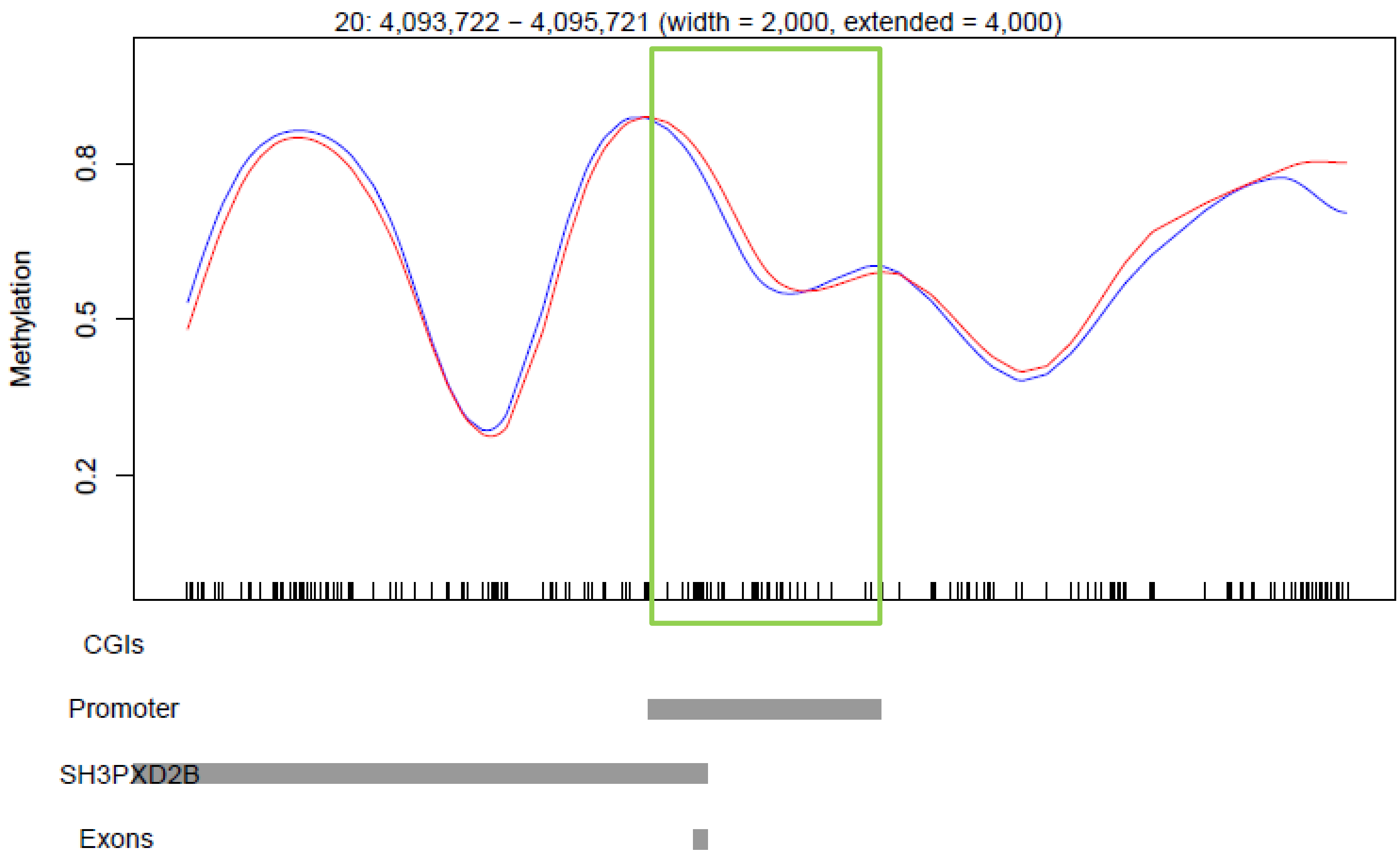
20: 4,093,722 − 4,095,721 (width = 2,000, extended = 4,000)
Methylation
0.2
0.5
0.8
CGIs
Promoter
SH3PXD2B
Exons

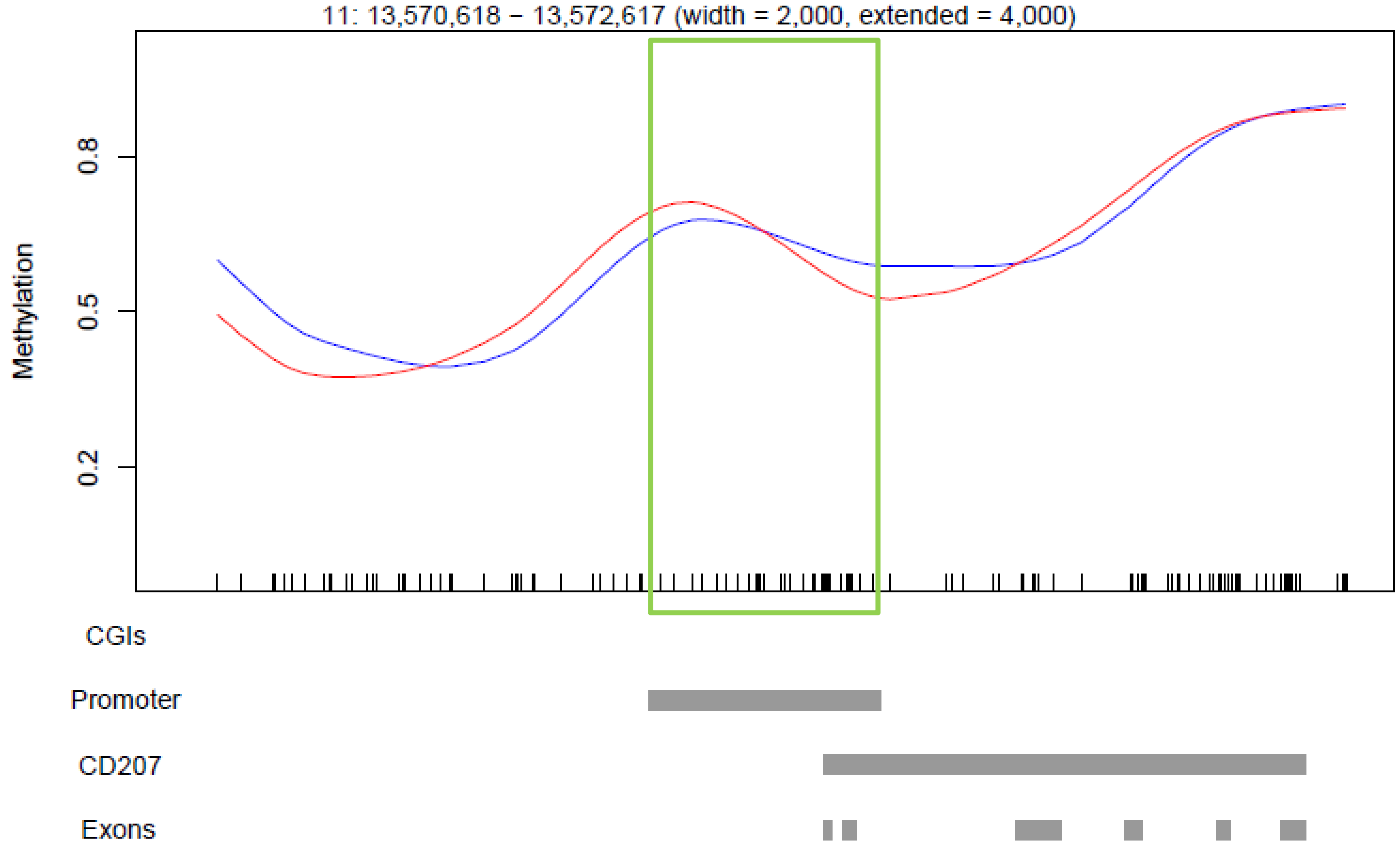

11: 13,570,618 − 13,572,617 (width = 2,000, extended = 4,000)
Methylation
0.2
0.5
0.8
CGIs
Promoter
CD207
Exons

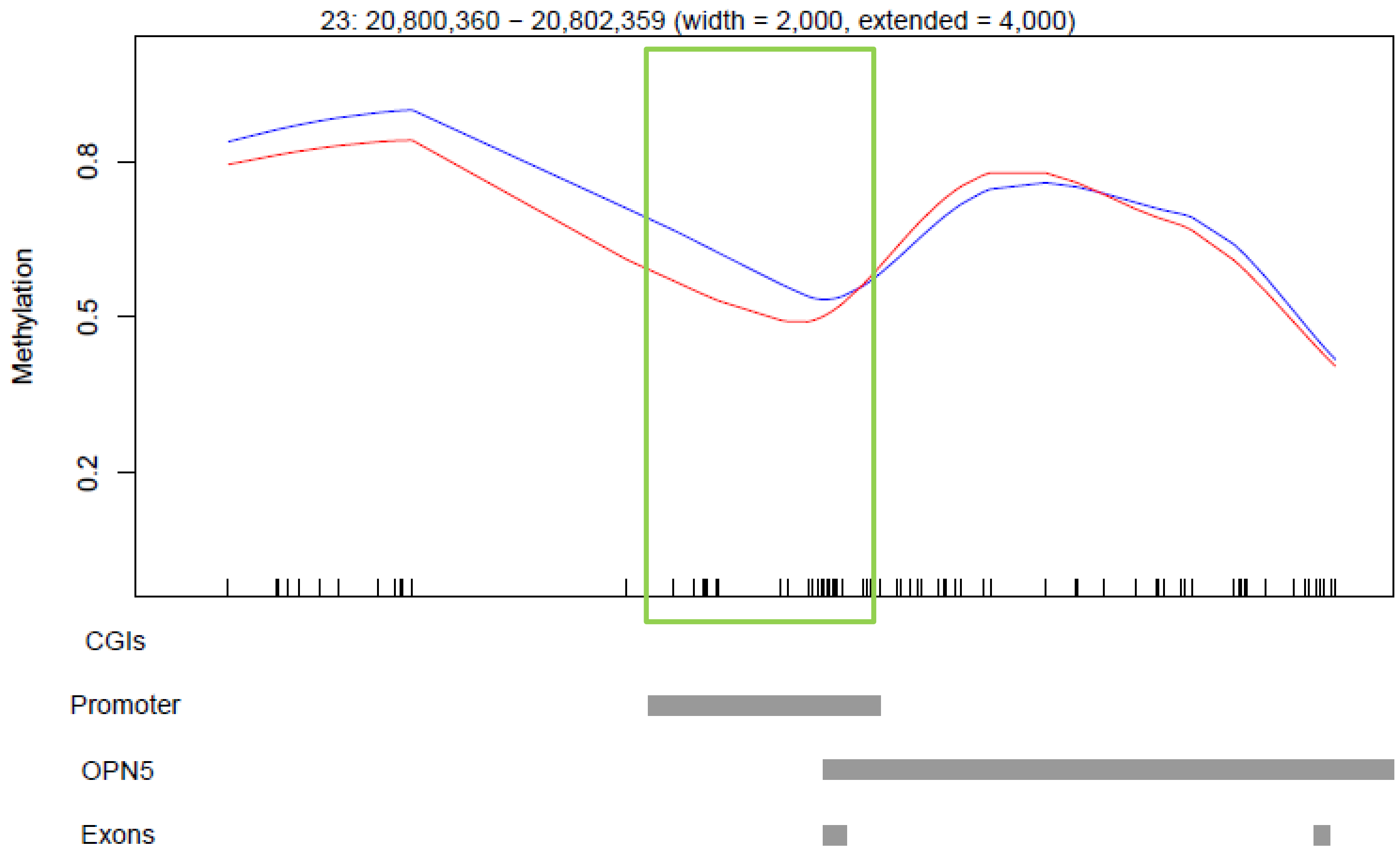

23: 20,800,360 – 20,802,359 (width = 2,000, extended = 4,000)
Methylation
0.2
0.5
0.8
CGIs
Promoter
OPN5
Exons

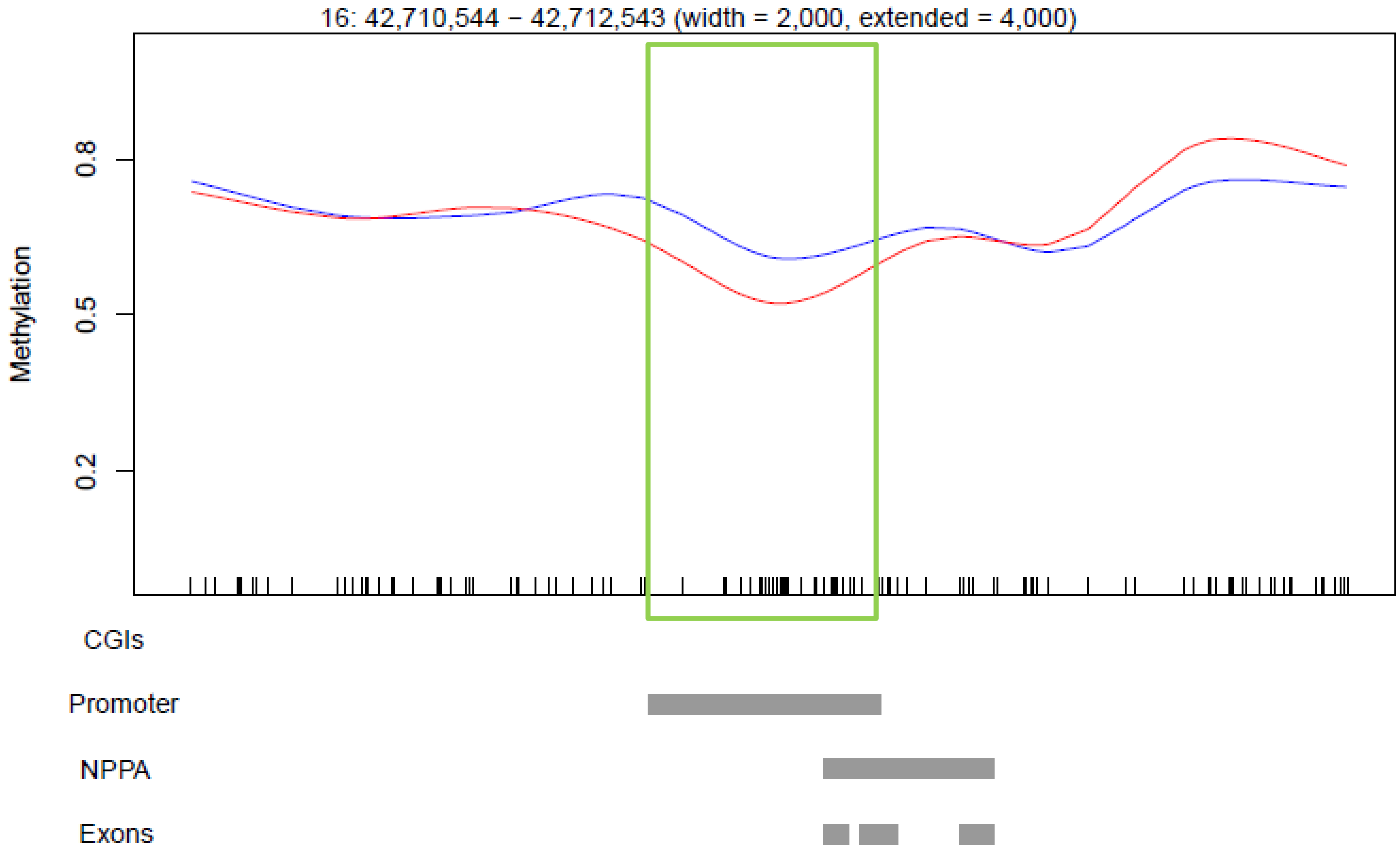

16: 42,710,544 − 42,712,543 (width = 2,000, extended = 4,000)
Methylation
0.2
0.5
0.8
CGIs
Promoter
NPPA
Exons

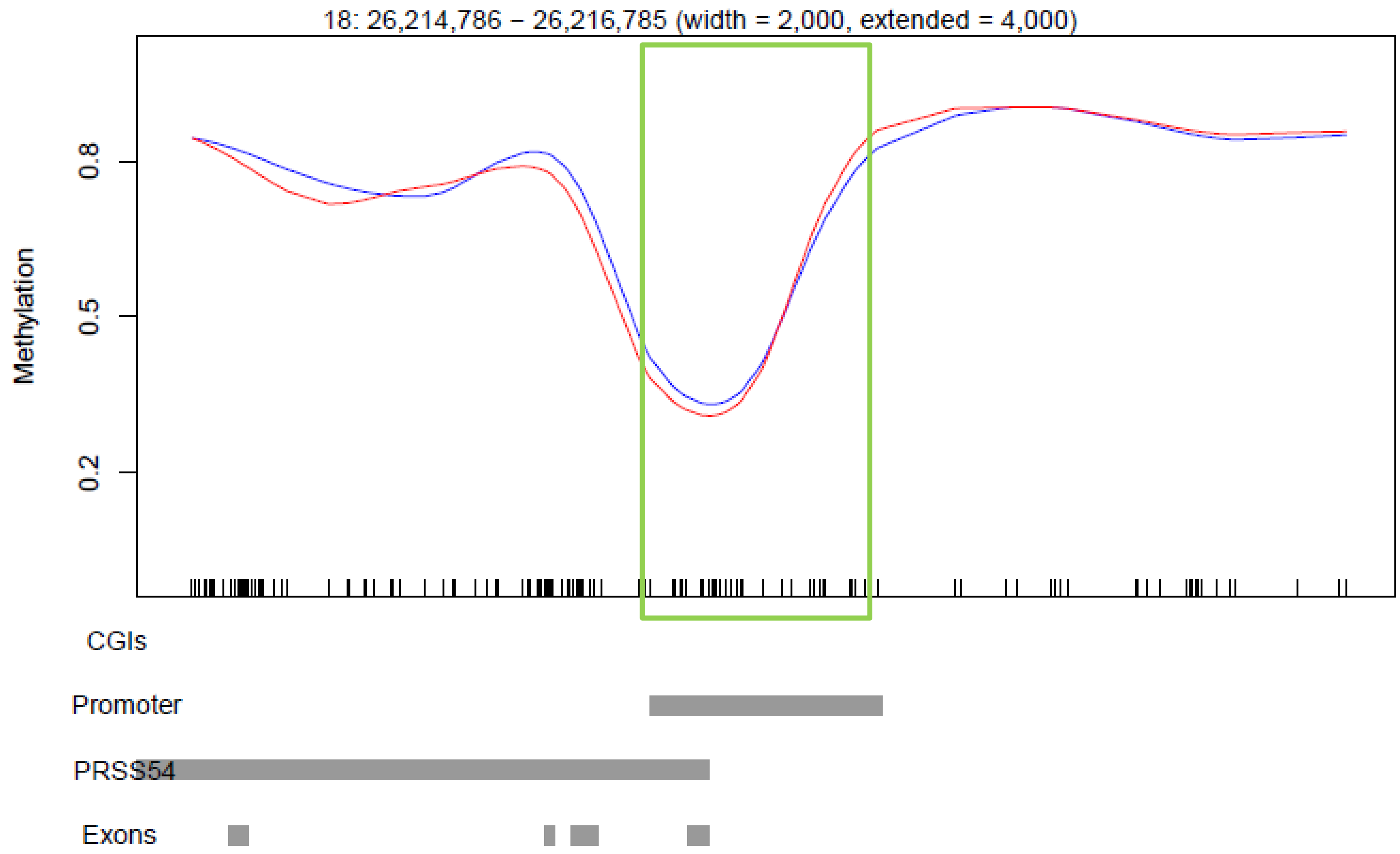
18: 26,214,786 – 26,216,785 (width = 2,000, extended = 4,000)
Methylation
0.8
0.5
0.2
CGIs
Promoter
PRSS54
Exons

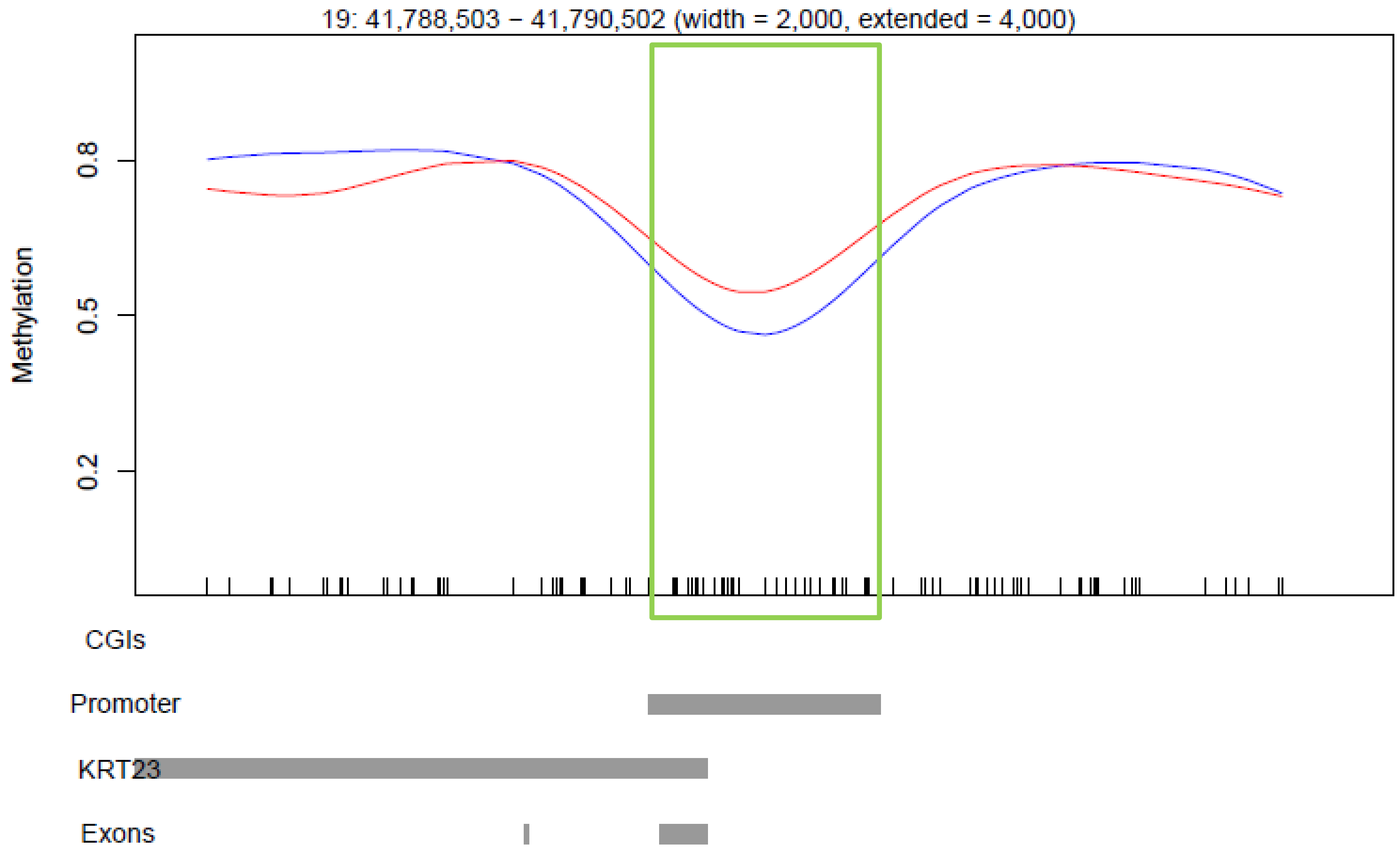
19: 41,788,503 – 41,790,502 (width = 2,000, extended = 4,000)
Methylation
0.8
0.5
0.2
CGIs
Promoter
KRT23
Exons

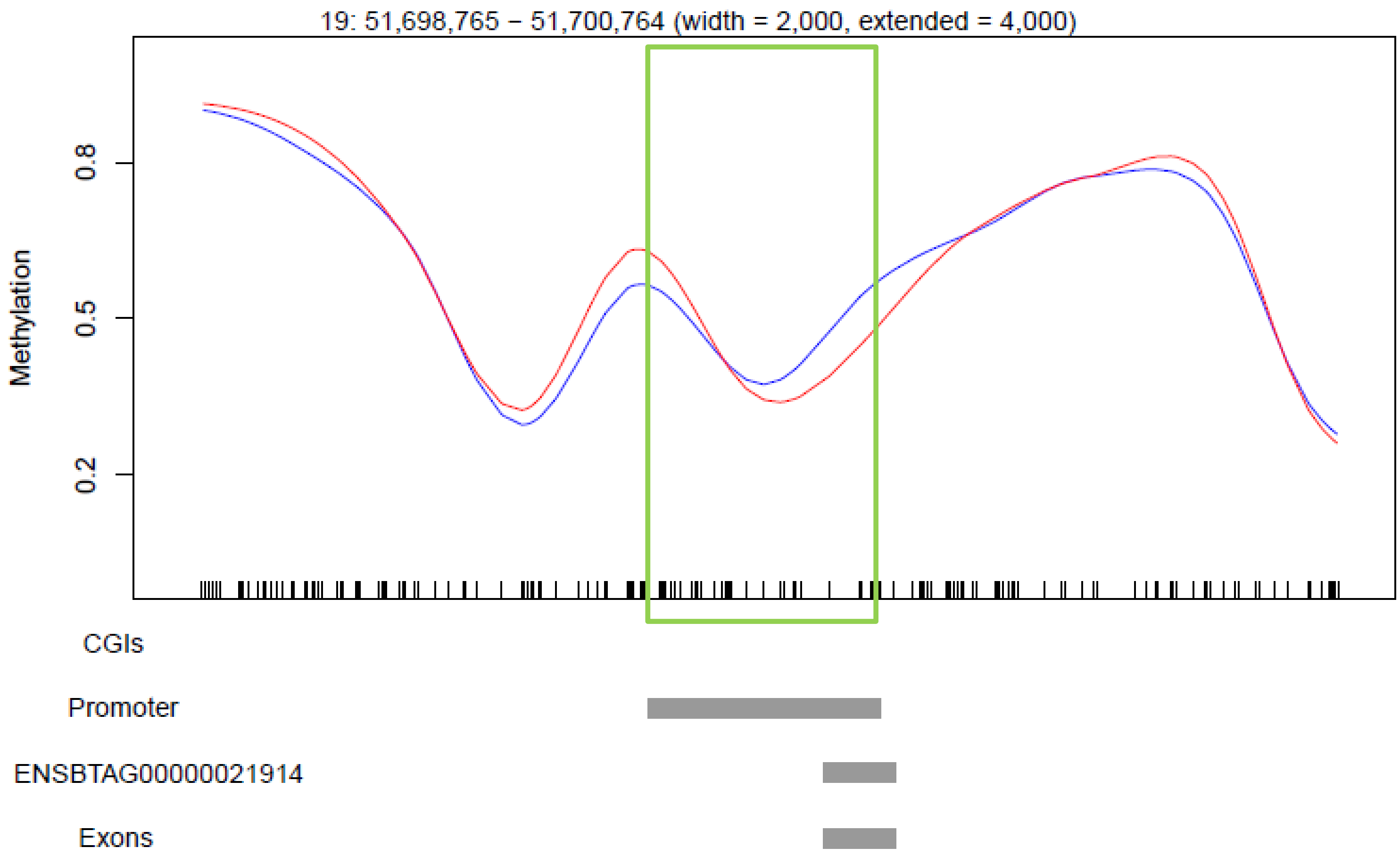

19: 51,698,765 – 51,700,764 (width = 2,000, extended = 4,000)
Methylation
0.8
0.5
0.2
CGIs
Promoter
ENSBTAG00000021914
Exons

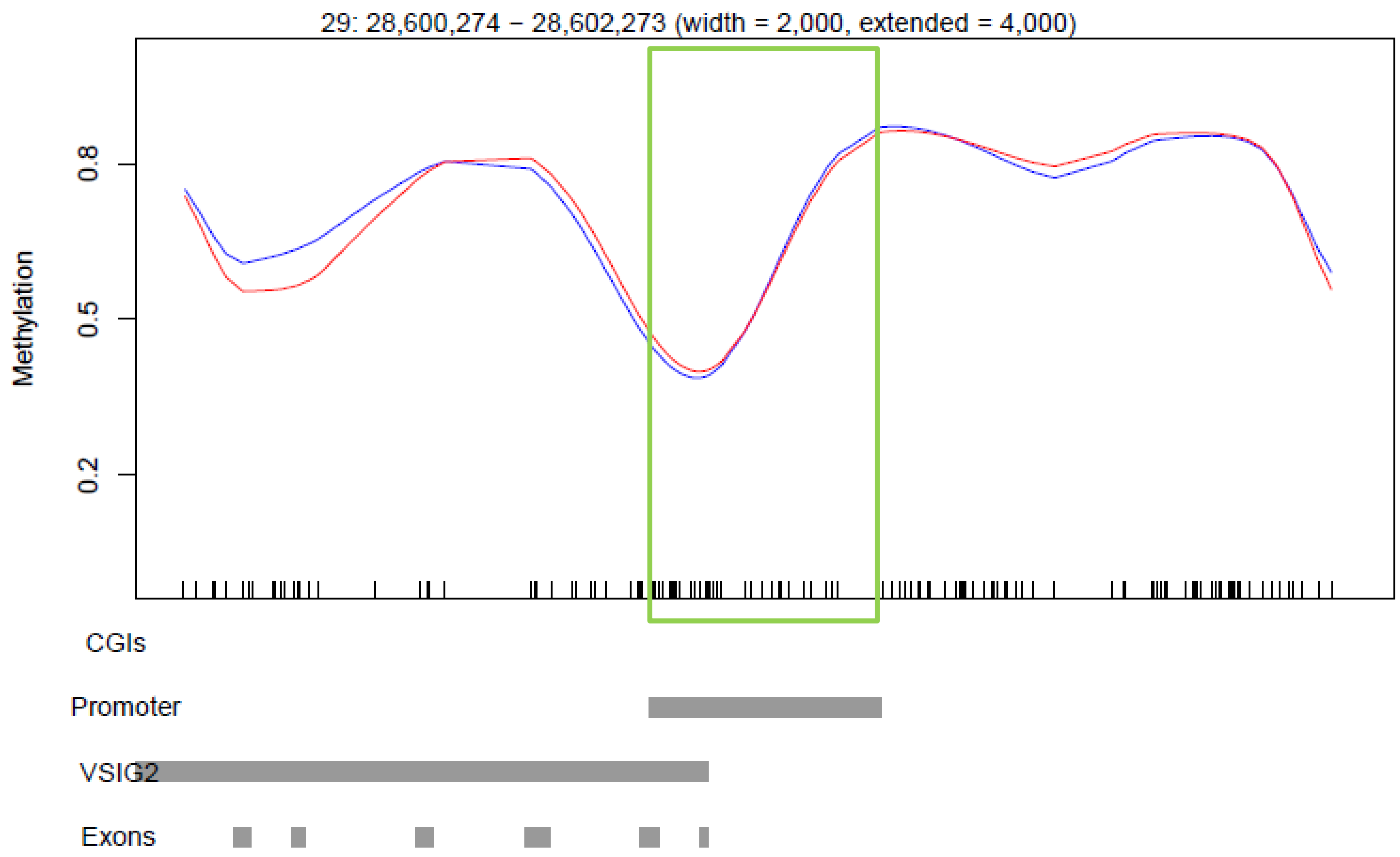
29: 28,600,274 − 28,602,273 (width = 2,000, extended = 4,000)
Methylation
0.8
0.5
0.2
CGIs
Promoter
VSIG2
Exons

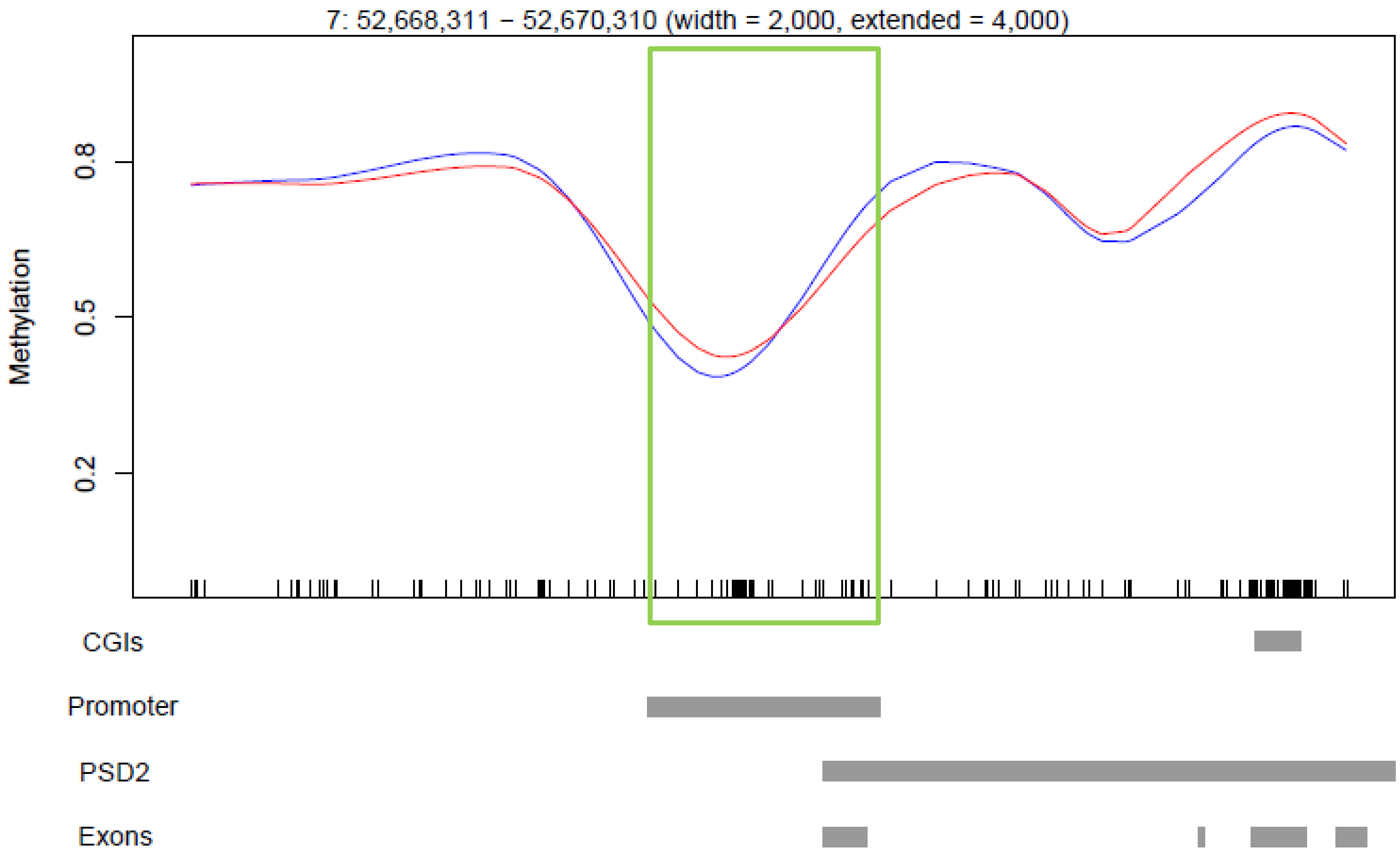

7: 52,668,311 – 52,670,310 (width = 2,000, extended = 4,000)
Methylation
0.8
0.5
0.2
CGIs
Promoter
PSD2
Exons

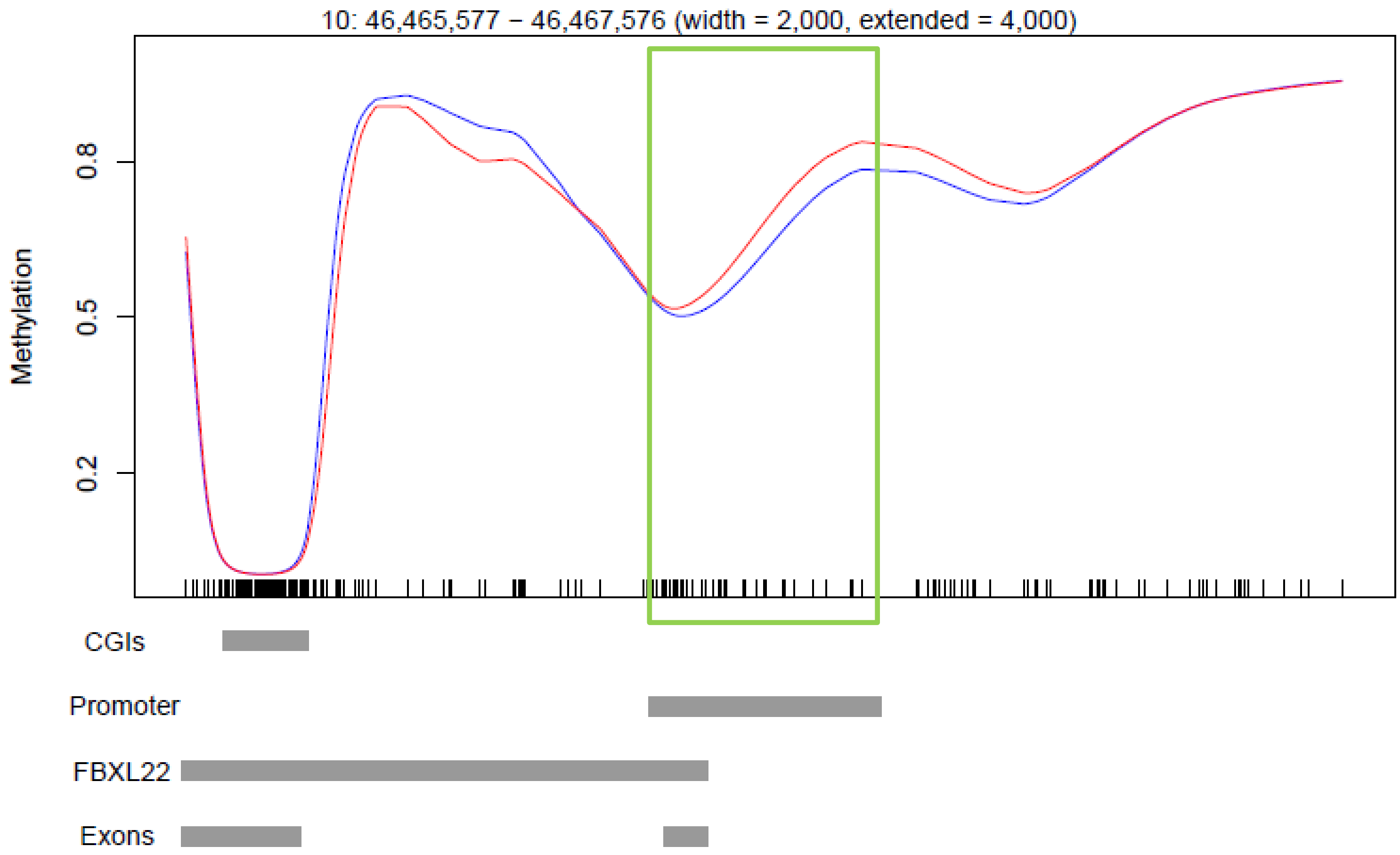

10: 46,465,577 − 46,467,576 (width = 2,000, extended = 4,000)
Methylation
0.2
0.5
0.8
CGIs
Promoter
FBXL22
Exons

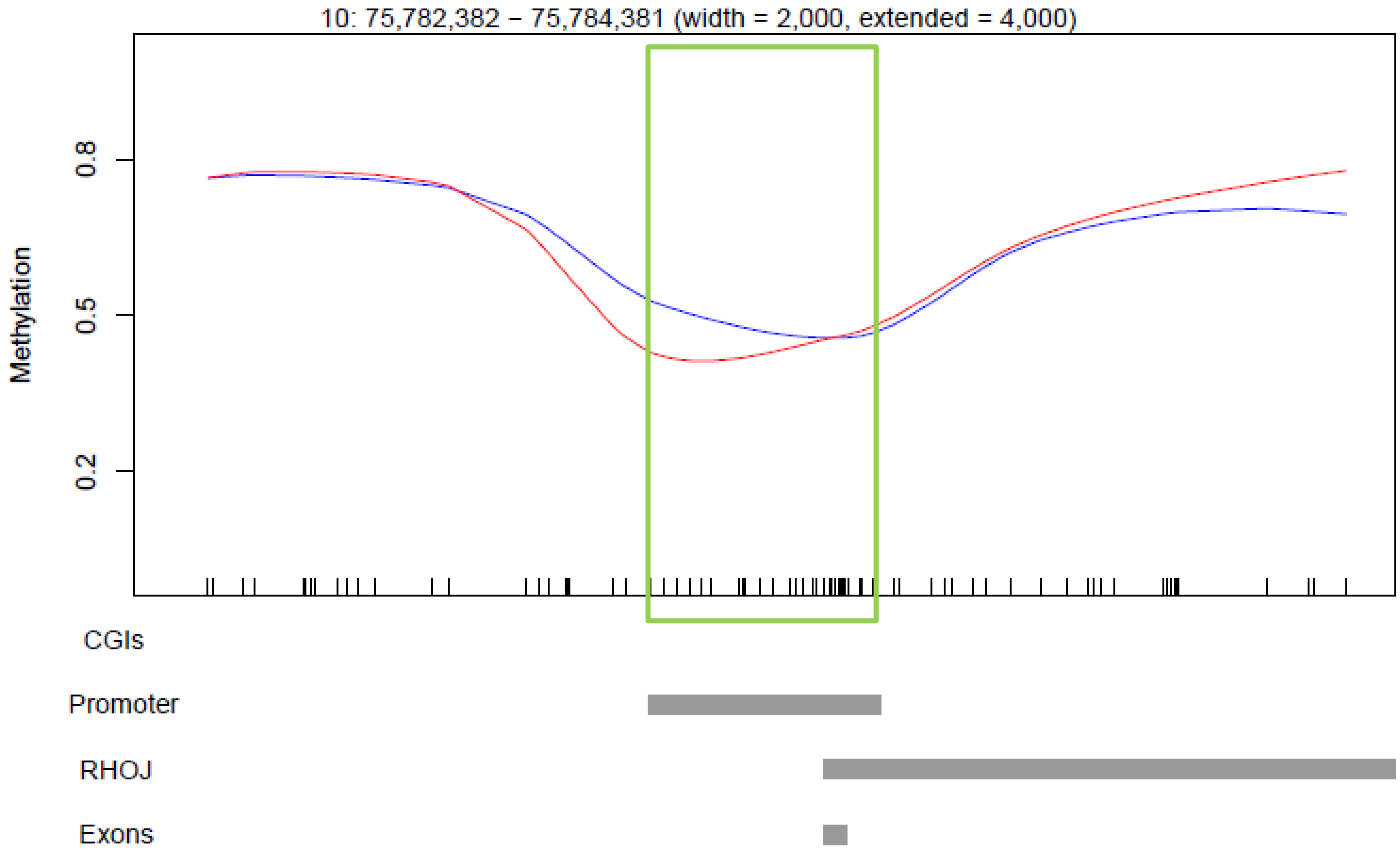
10: 75,782,382 − 75,784,381 (width = 2,000, extended = 4,000)
Methylation
0.8
0.5
0.2
CGIs
Promoter
RHOJ
Exons

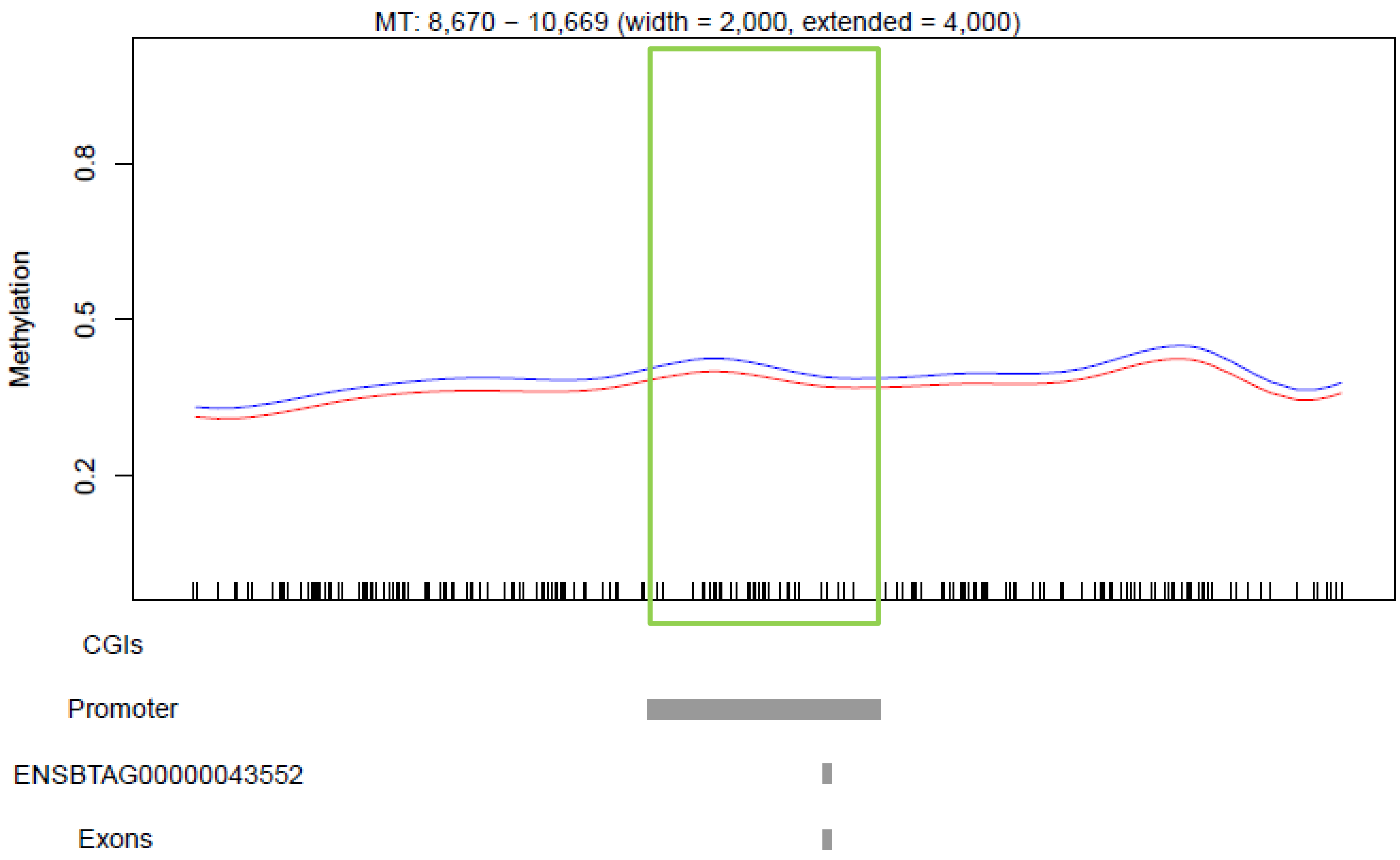
MT: 8,670 – 10,669 (width = 2,000, extended = 4,000)
Methylation
0.8
0.5
0.2
CGIs
Promoter
ENSBTAG00000043552
Exons

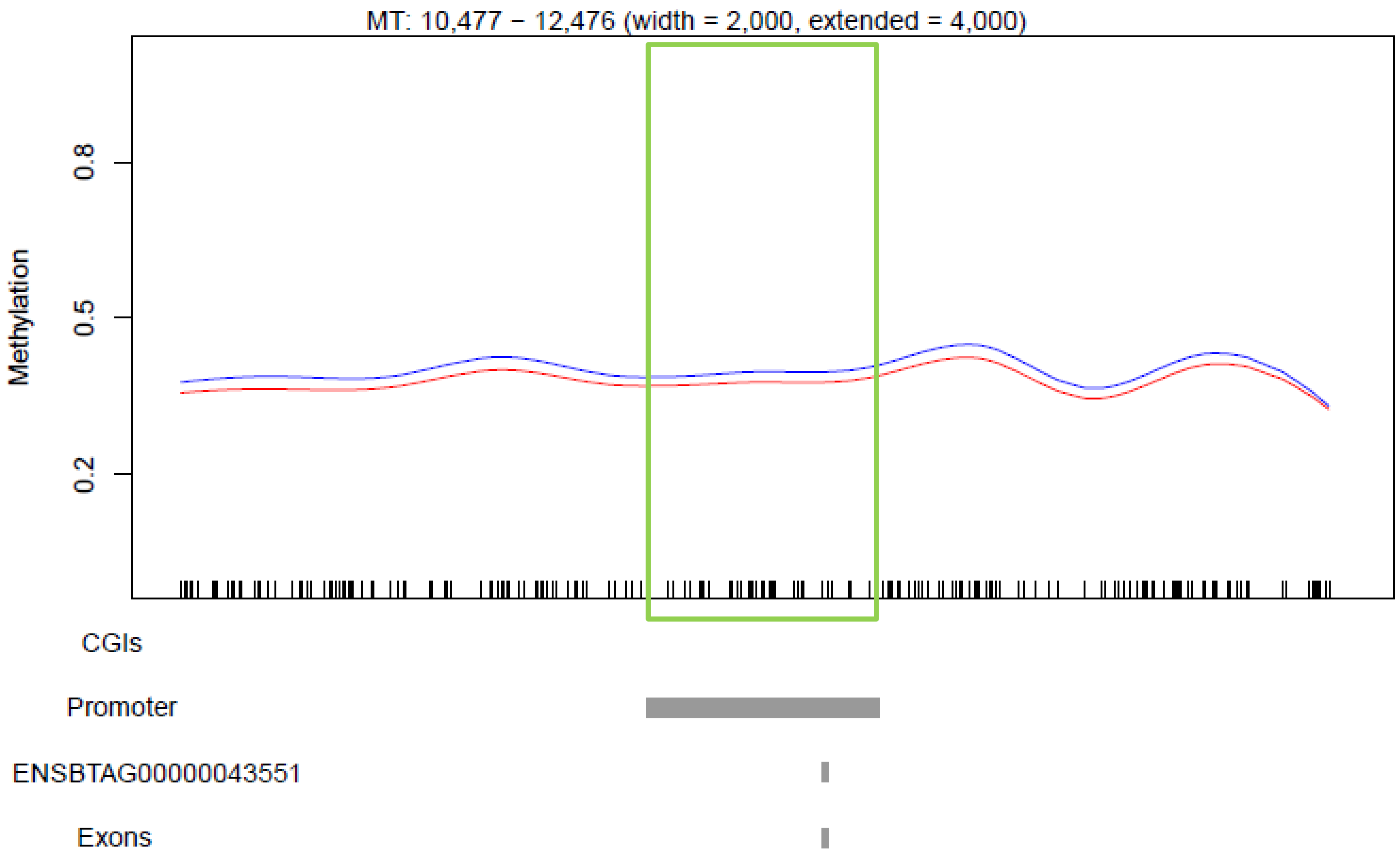

MT: 10,477 − 12,476 (width = 2,000, extended = 4,000)
Methylation
0.8
0.5
0.2
CGIs
Promoter
ENSBTAG00000043551
Exons

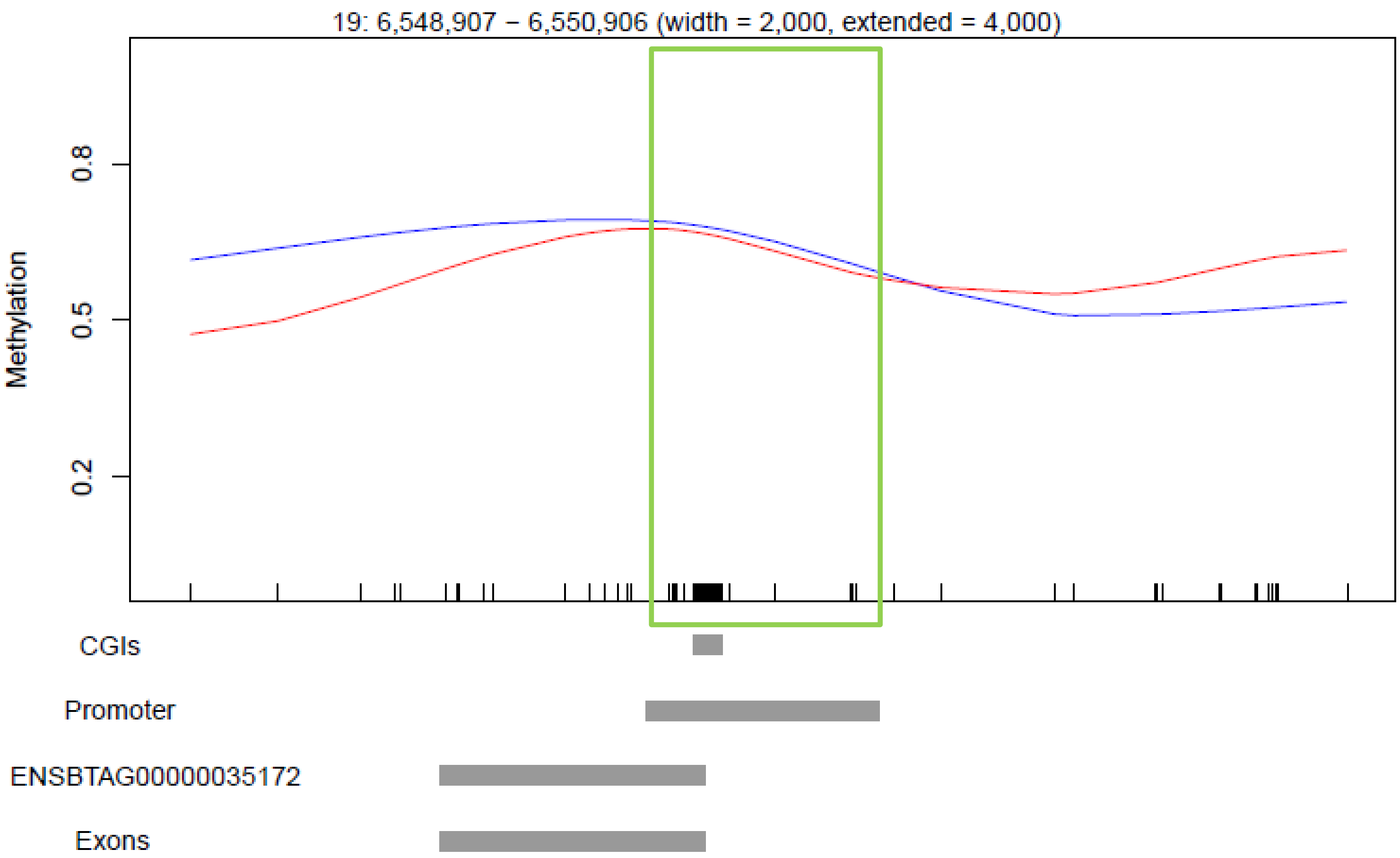

19: 6,548,907 − 6,550,906 (width = 2,000, extended = 4,000)
Methylation
0.8
0.5
0.2
CGIs
Promoter
ENSBTAG00000035172
Exons

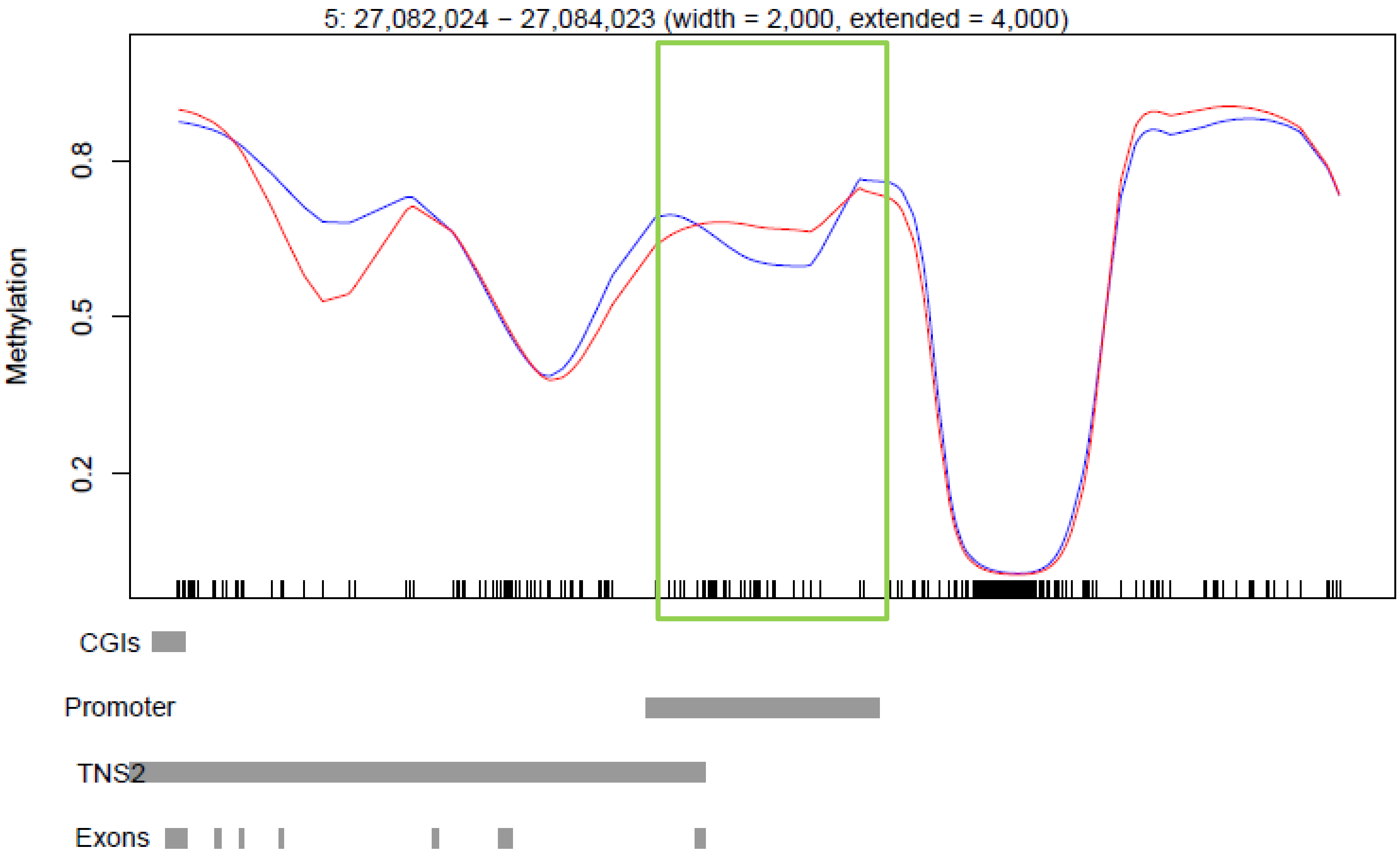

5: 27,082,024 − 27,084,023 (width = 2,000, extended = 4,000)
Methylation
0.8
0.5
0.2
CGIs
Promoter
TNS2
Exons

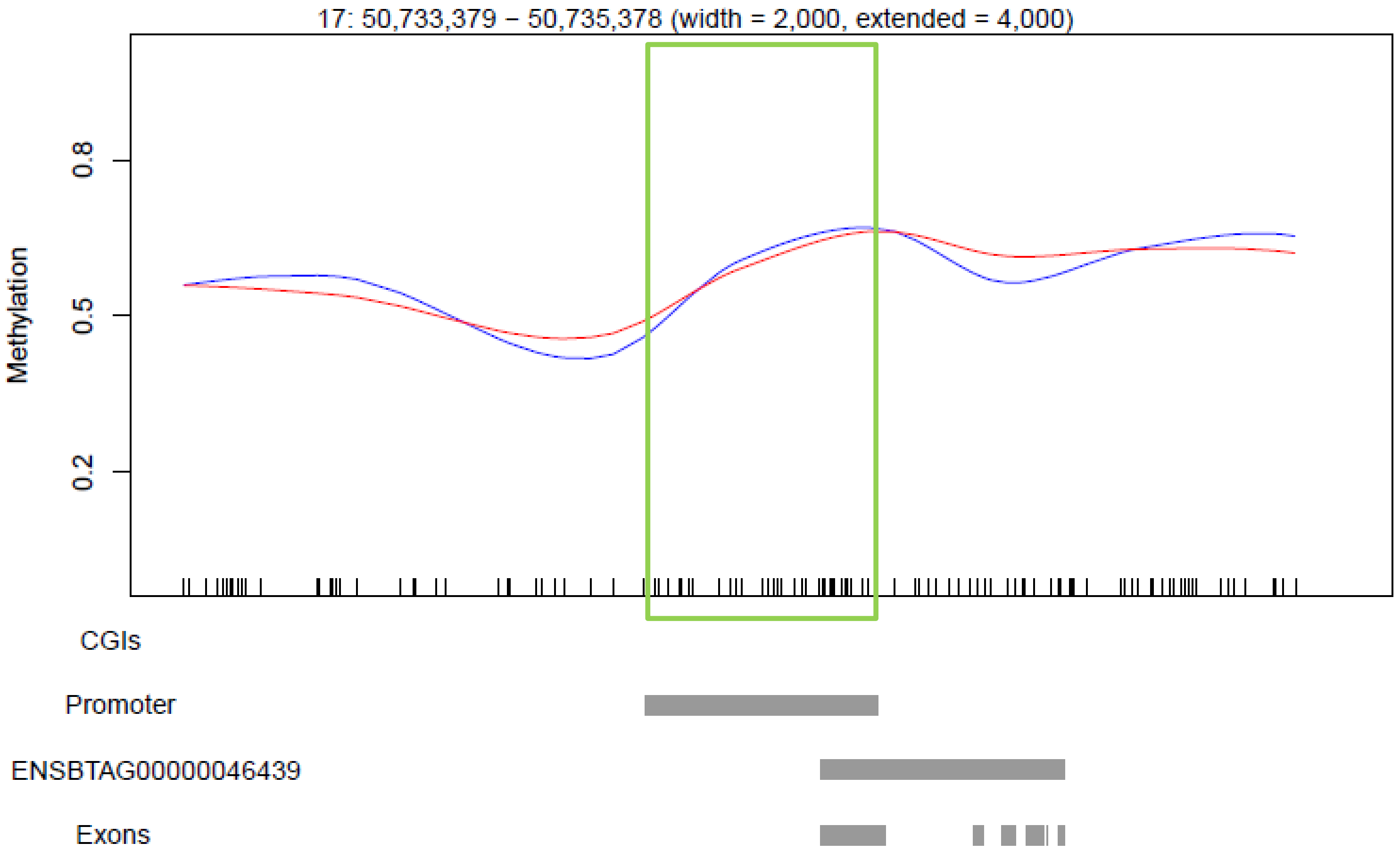

17: 50,733,379 – 50,735,378 (width = 2,000, extended = 4,000)
Methylation
0.8
0.5
0.2
CGIs
Promoter
ENSBTAG00000046439
Exons

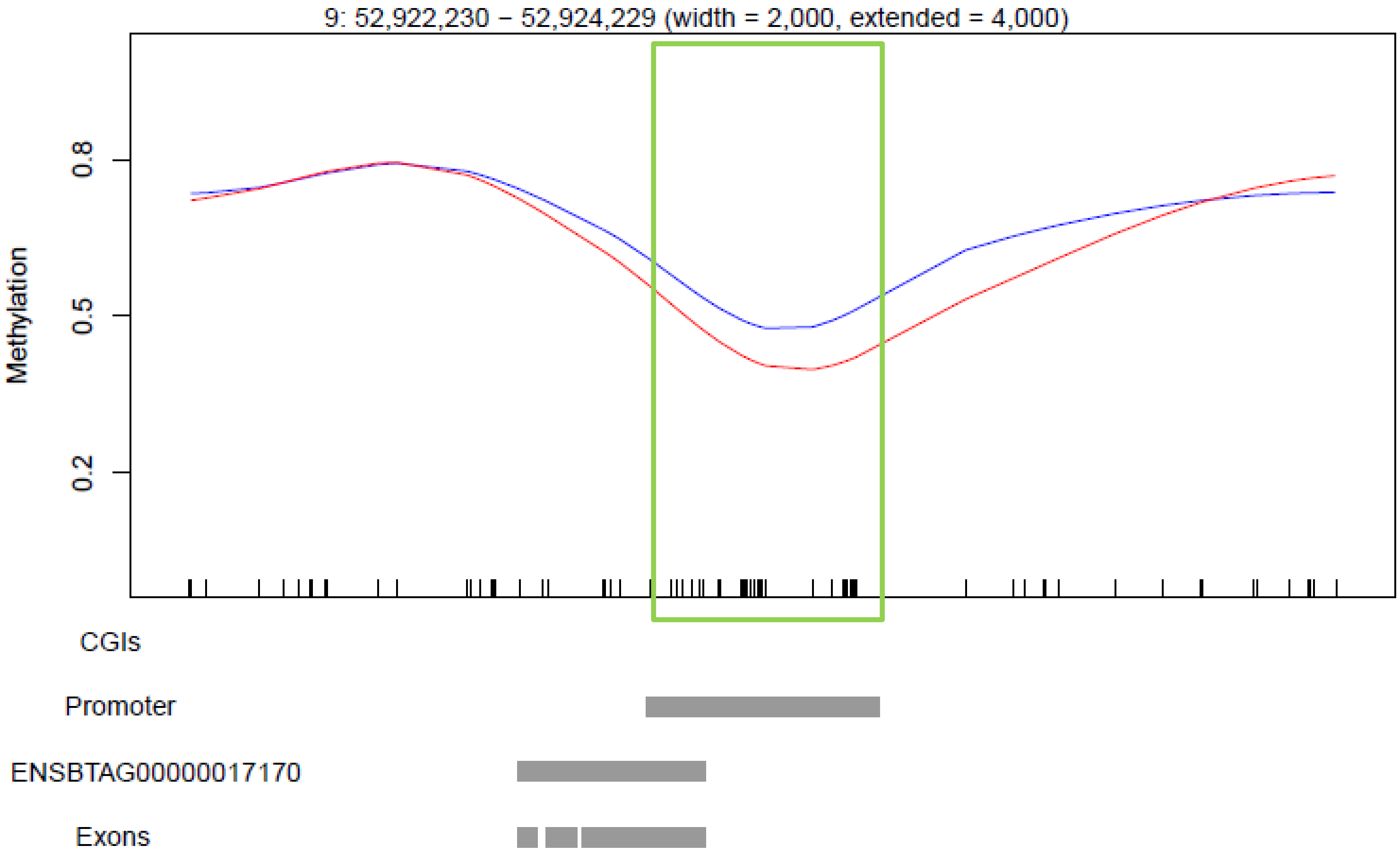
9: 52,922,230 – 52,924,229 (width = 2,000, extended = 4,000)
Methylation
0.8
0.5
0.2
CGIs
Promoter
ENSBTAG00000017170
Exons

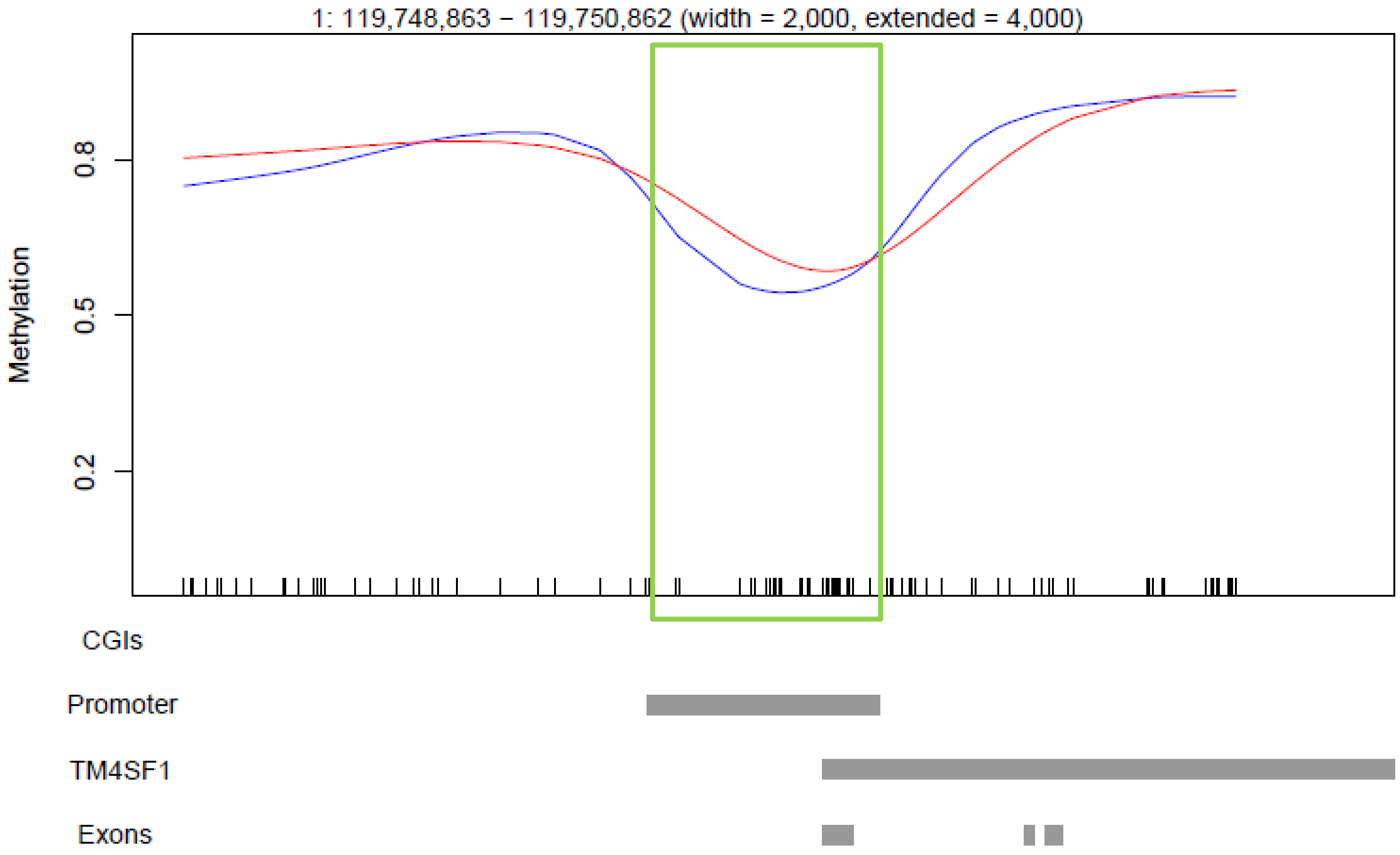
1: 119,748,863 – 119,750,862 (width = 2,000, extended = 4,000)
Methylation
0.8
0.5
0.2
CGIs
Promoter
TM4SF1
Exons

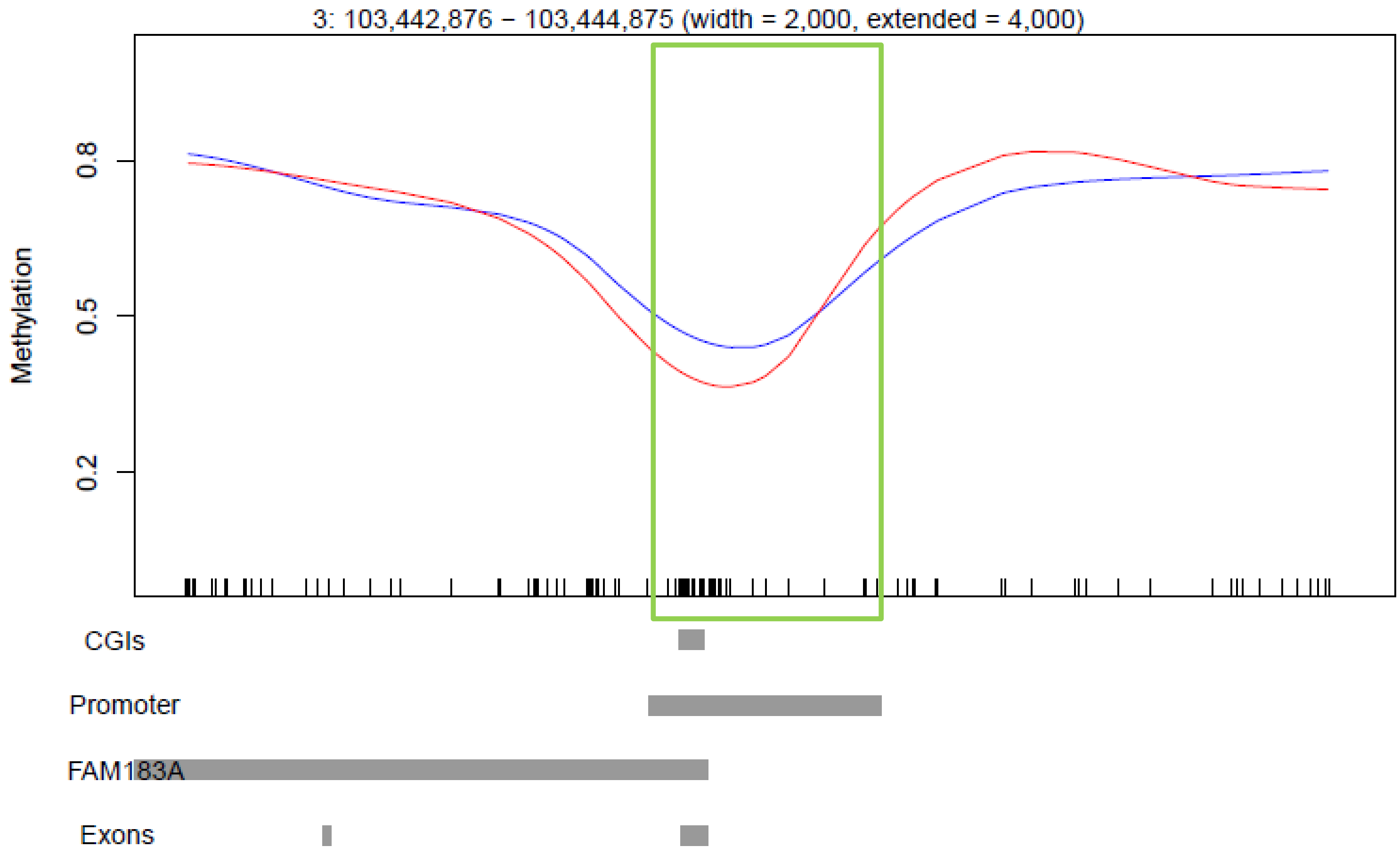
3: 103,442,876 − 103,444,875 (width = 2,000, extended = 4,000)
Methylation
0.8
0.5
0.2
CGIs
Promoter
FAM183A
Exons

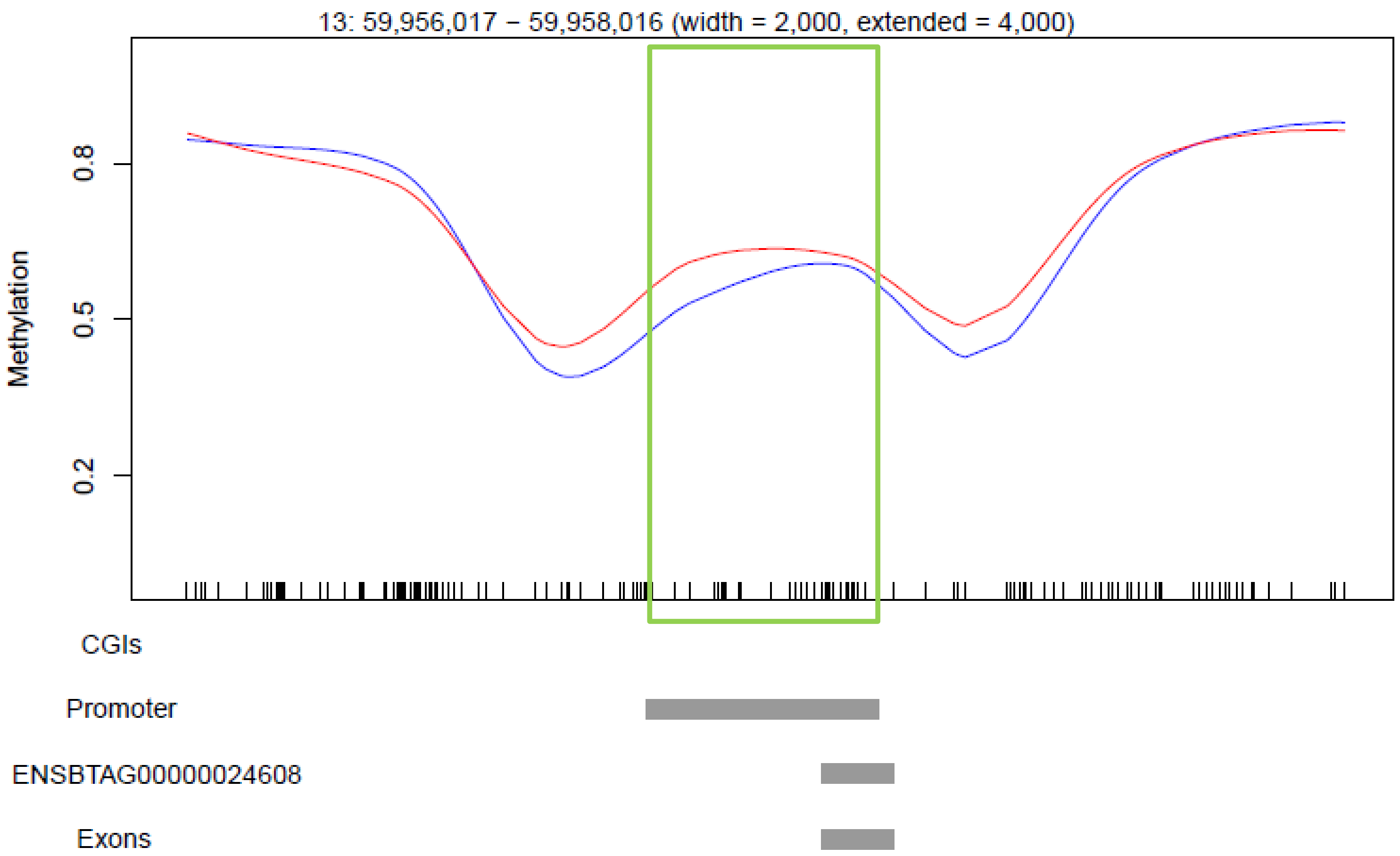
13: 59,956,017 − 59,958,016 (width = 2,000, extended = 4,000)
Methylation
0.8
0.5
0.2
CGIs
Promoter
ENSBTAG00000024608
Exons

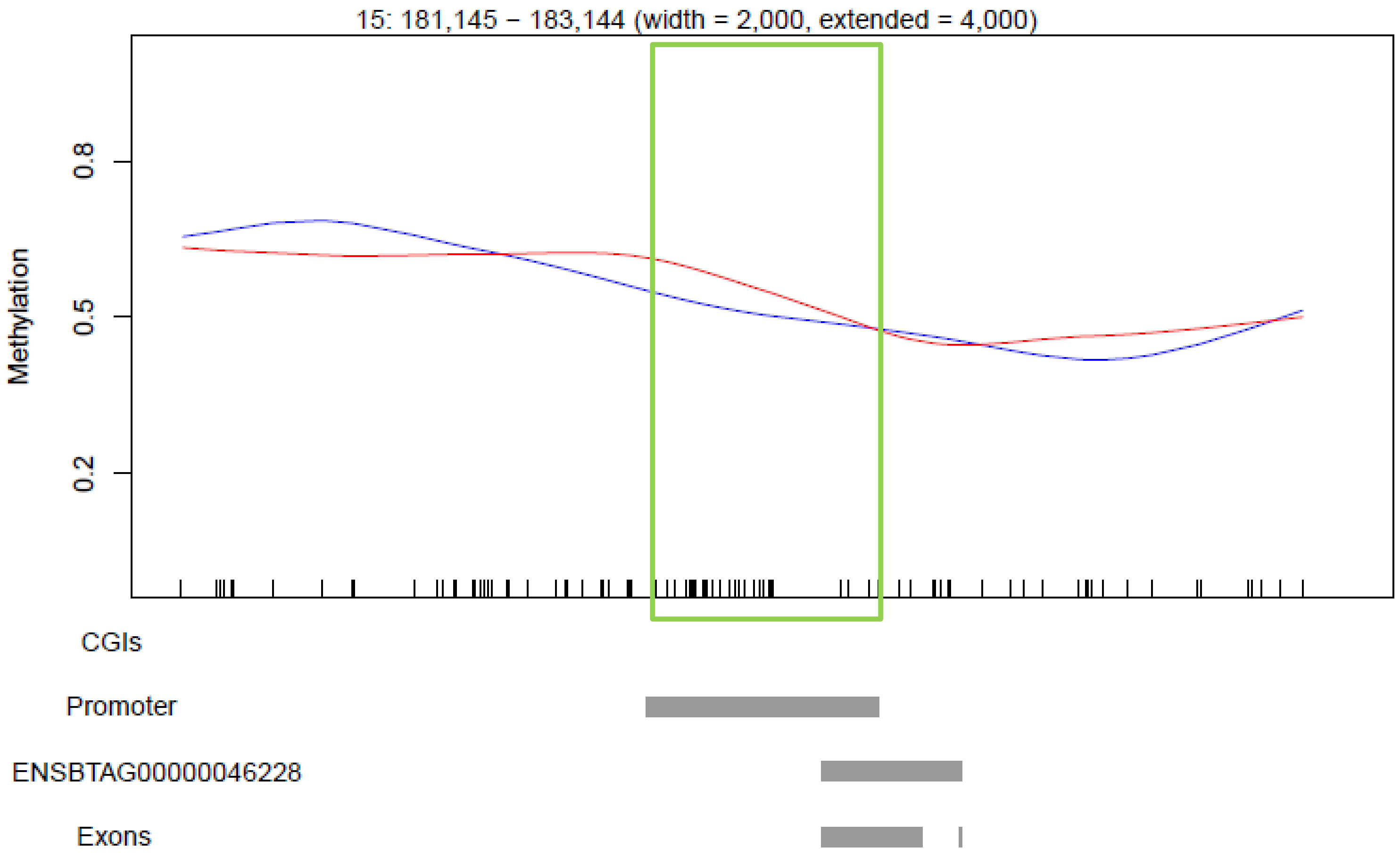

15: 181,145 − 183,144 (width = 2,000, extended = 4,000)
Methylation
0.2
0.5
0.8
CGIs
Promoter
ENSBTAG00000046228
Exons

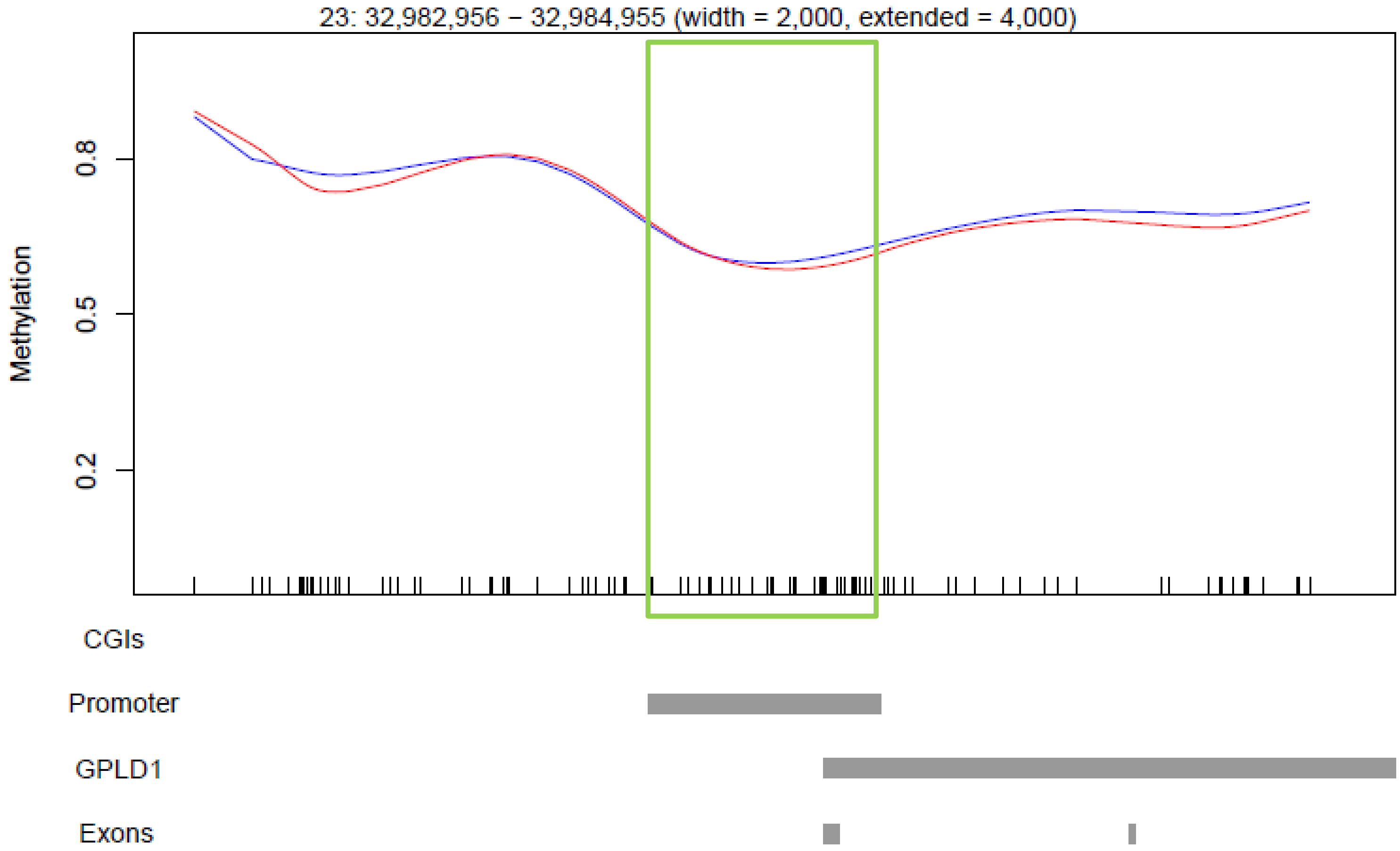
23: 32,982,956 − 32,984,955 (width = 2,000, extended = 4,000)
Methylation
0.8
0.5
0.2
CGIs
Promoter
GPLD1
Exons

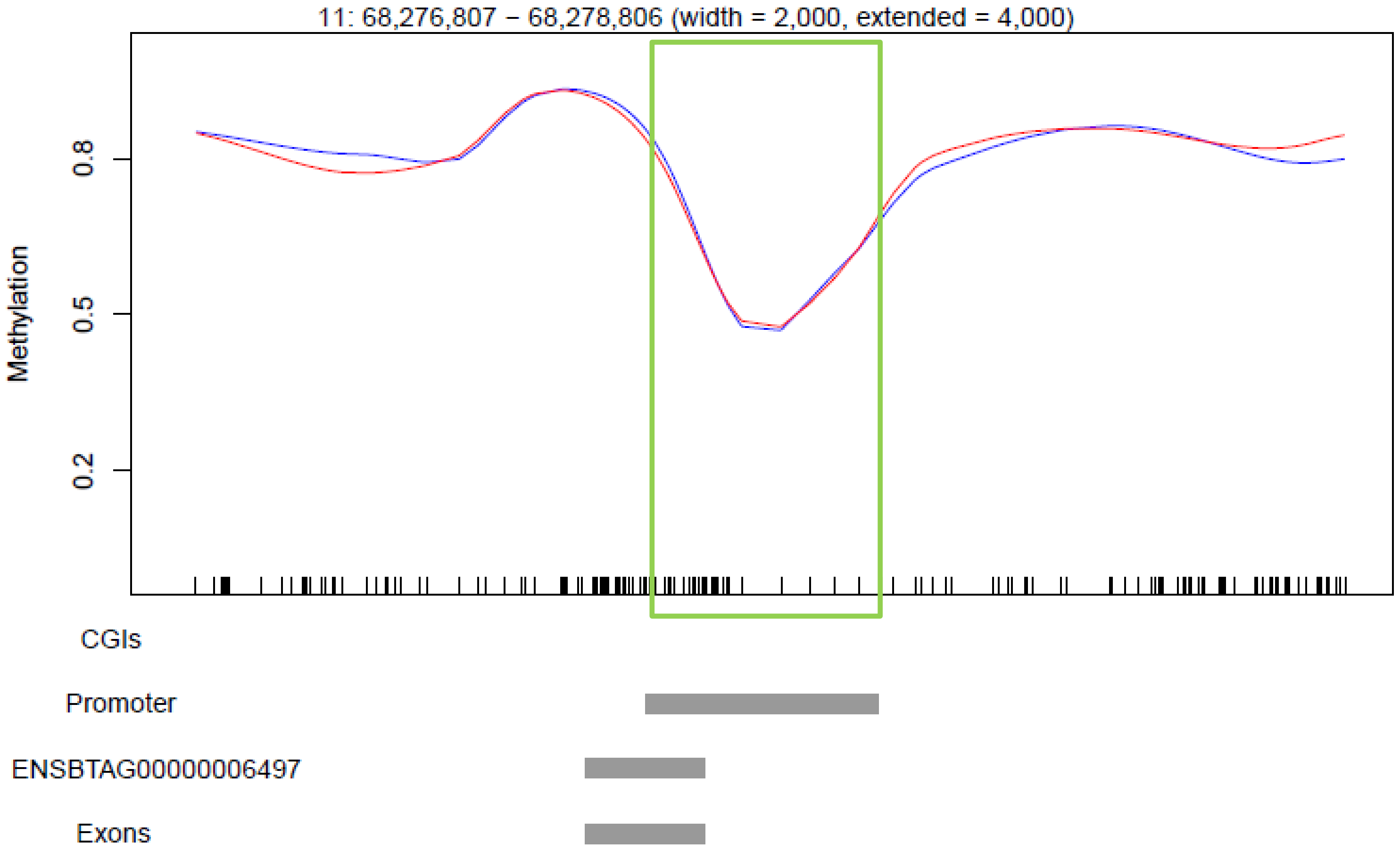
11: 68,276,807 – 68,278,806 (width = 2,000, extended = 4,000)
Methylation
0.2
0.5
0.8
CGIs
Promoter
ENSBTAG00000006497
Exons

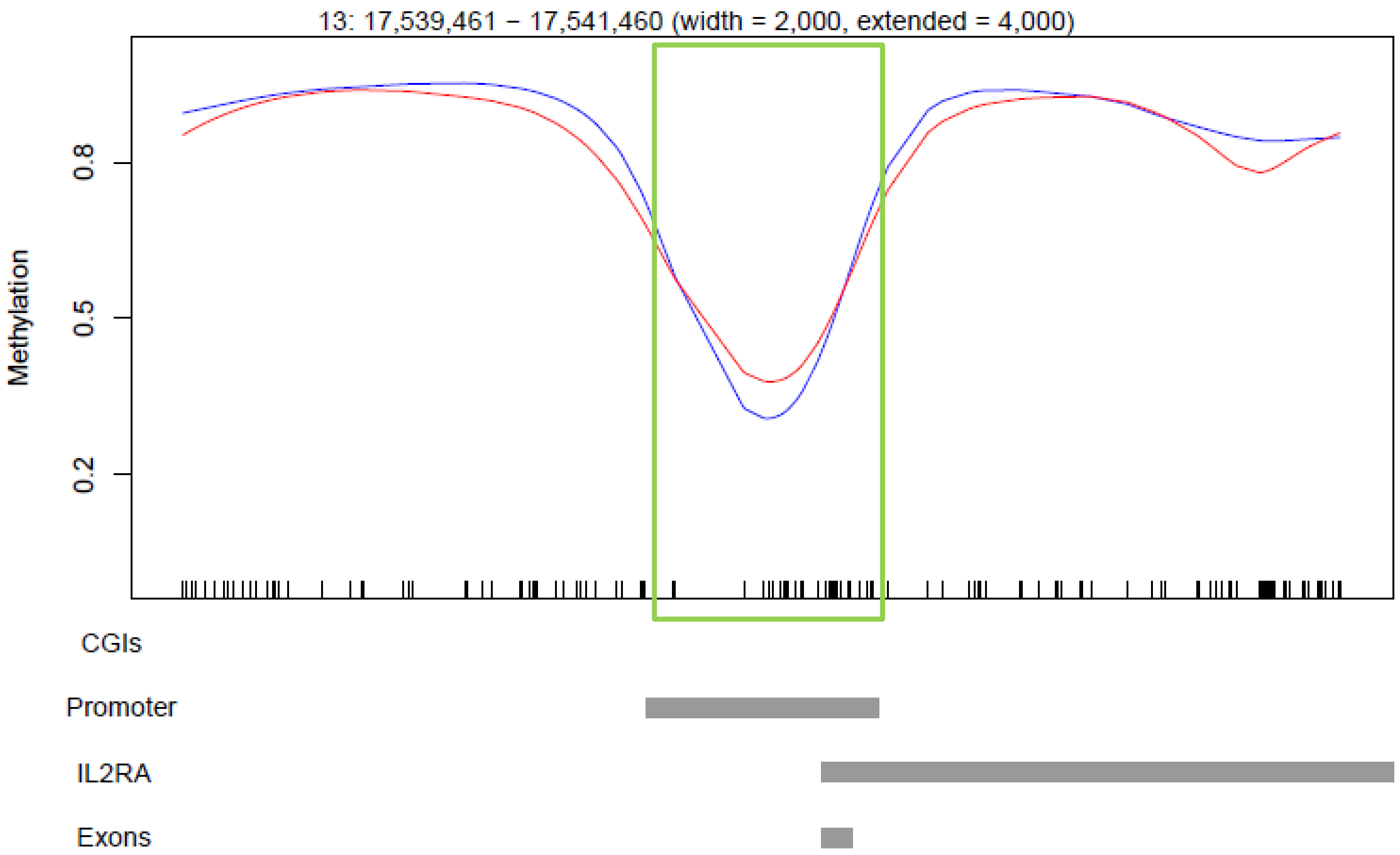
13: 17,539,461 – 17,541,460 (width = 2,000, extended = 4,000)
Methylation
0.8
0.5
0.2
CGIs
Promoter
IL2RA
Exons

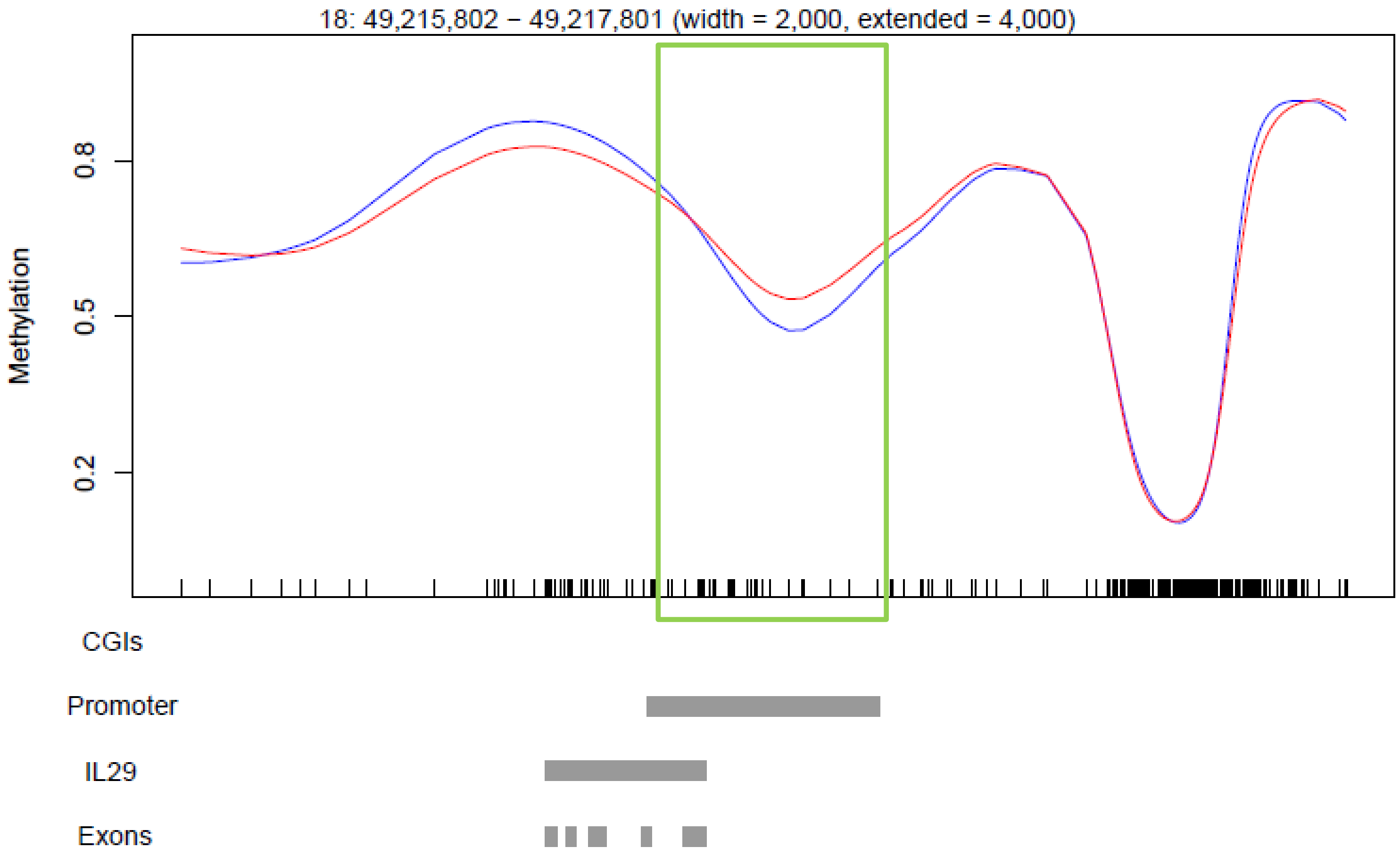

18: 49,215,802 − 49,217,801 (width = 2,000, extended = 4,000)
Methylation
0.2
0.5
0.8
CGIs
Promoter
IL29
Exons

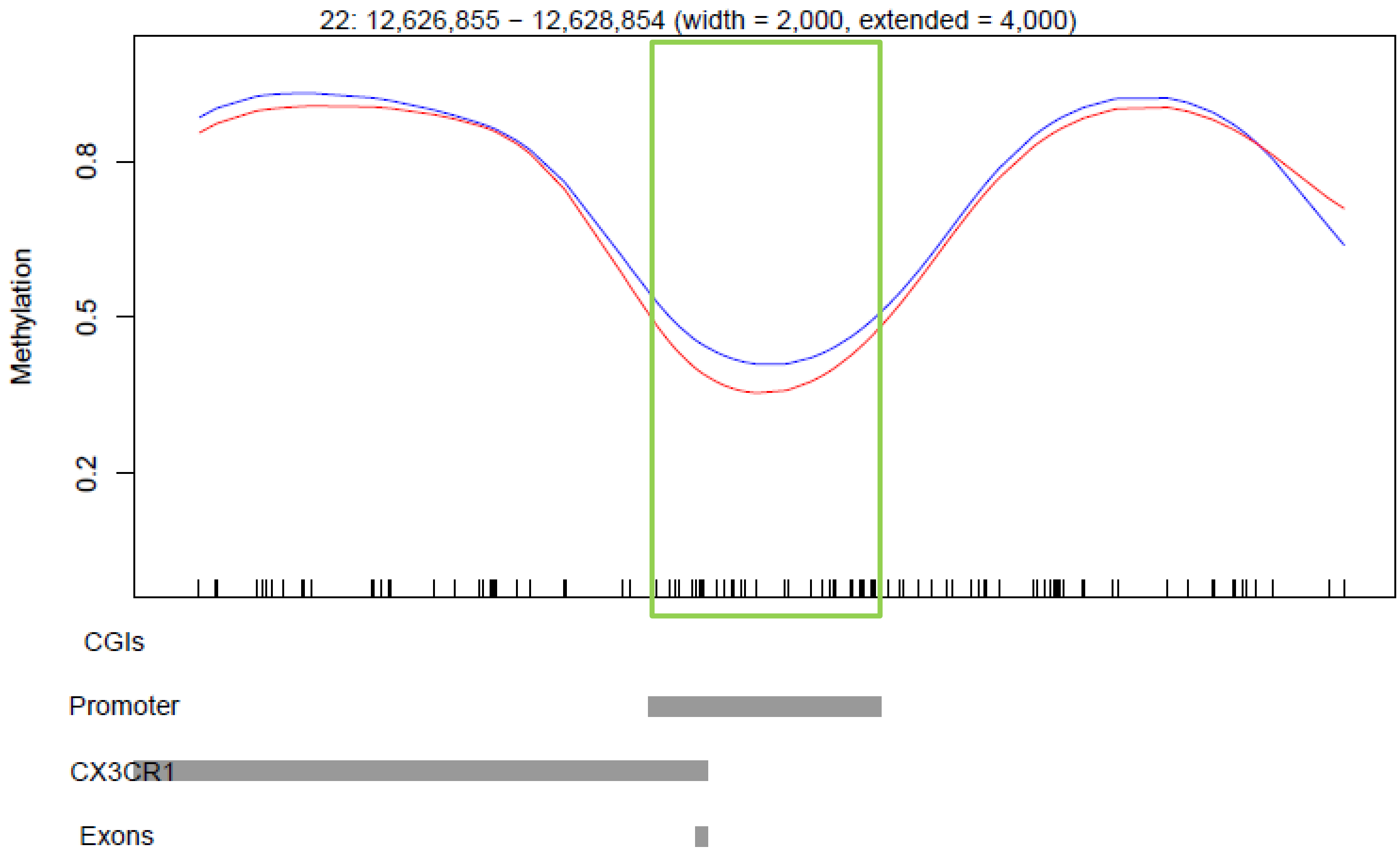
22: 12,626,855 – 12,628,854 (width = 2,000, extended = 4,000)
Methylation
0.2
0.5
0.8
CGIs
Promoter
CX3CR1
Exons

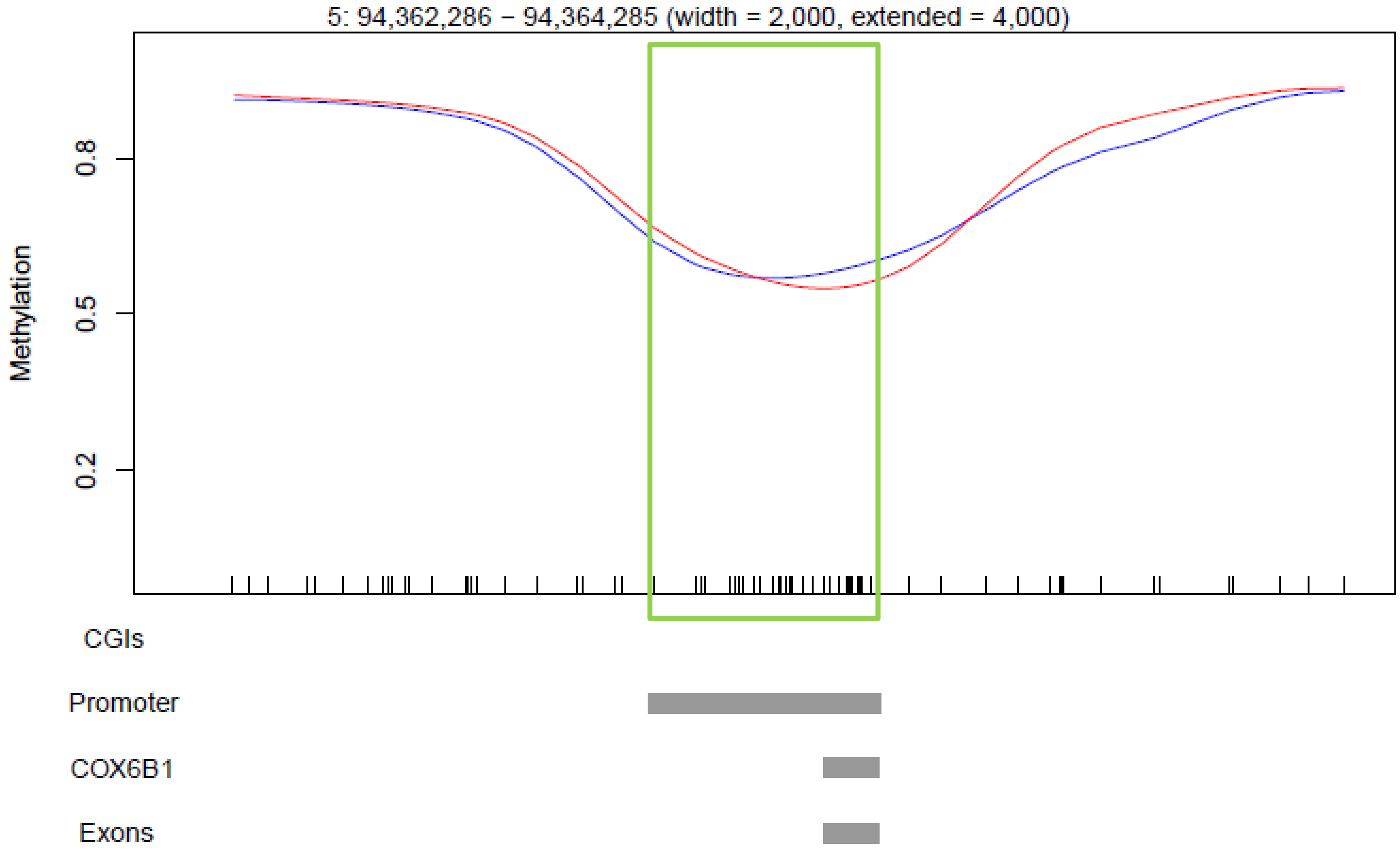
5: 94,362,286 − 94,364,285 (width = 2,000, extended = 4,000)
Methylation
0.8
0.5
0.2
CGIs
Promoter
COX6B1
Exons

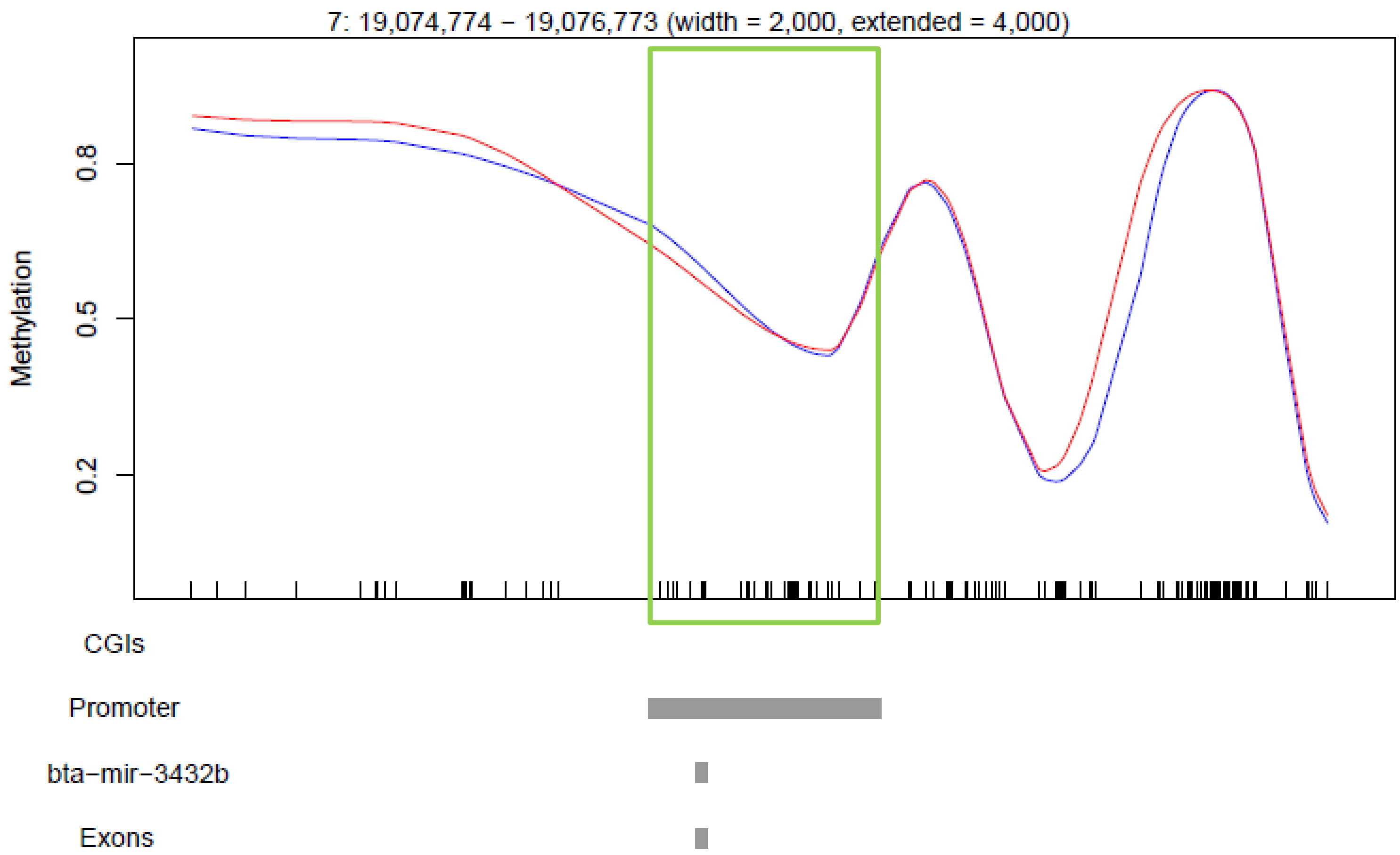

7: 19,074,774 − 19,076,773 (width = 2,000, extended = 4,000)
Methylation
0.8
0.5
0.2
CGIs
Promoter
bta−mir−3432b
Exons

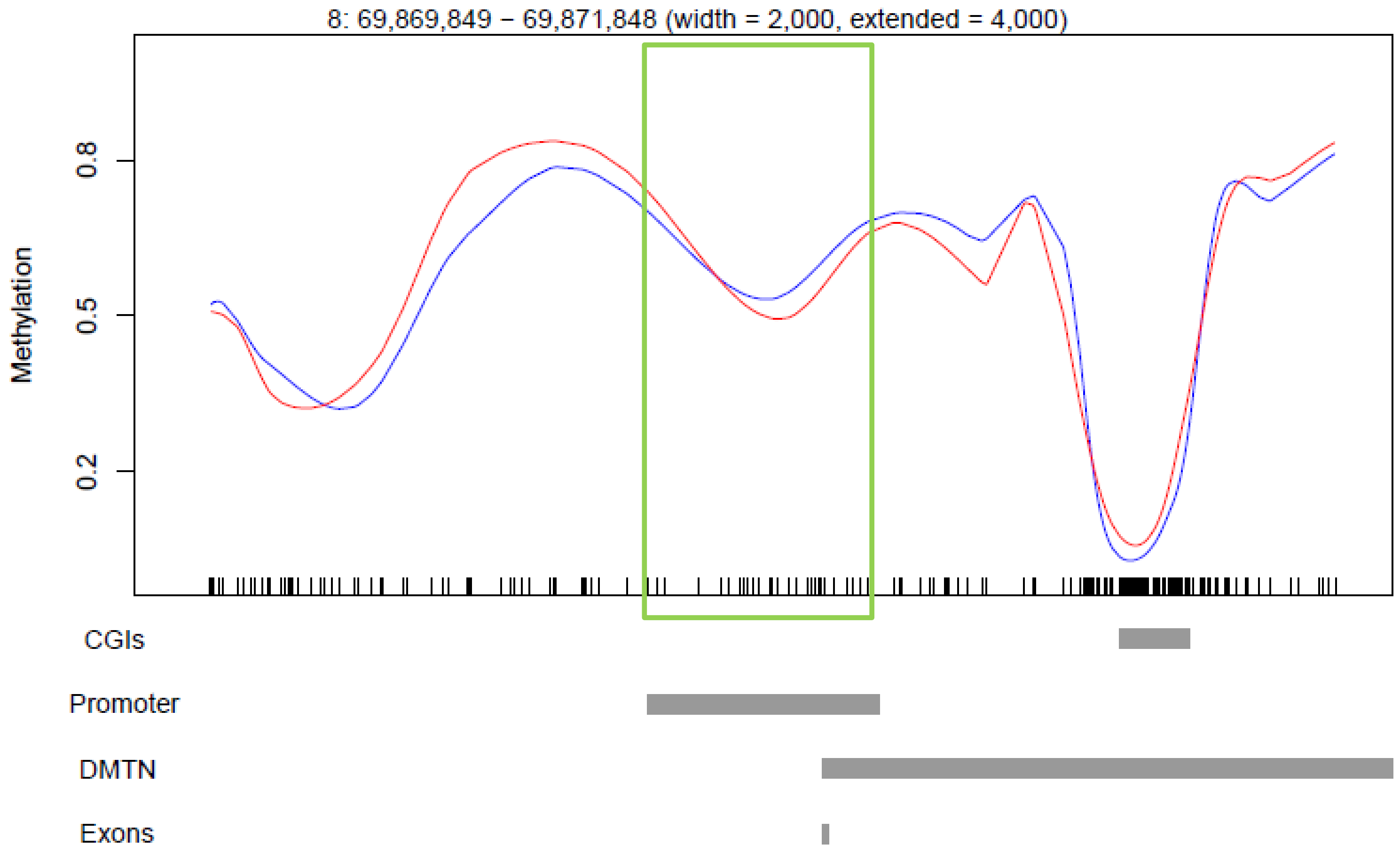
8: 69,869,849 − 69,871,848 (width = 2,000, extended = 4,000)
Methylation
0.8
0.5
0.2
CGIs
Promoter
DMTN
Exons

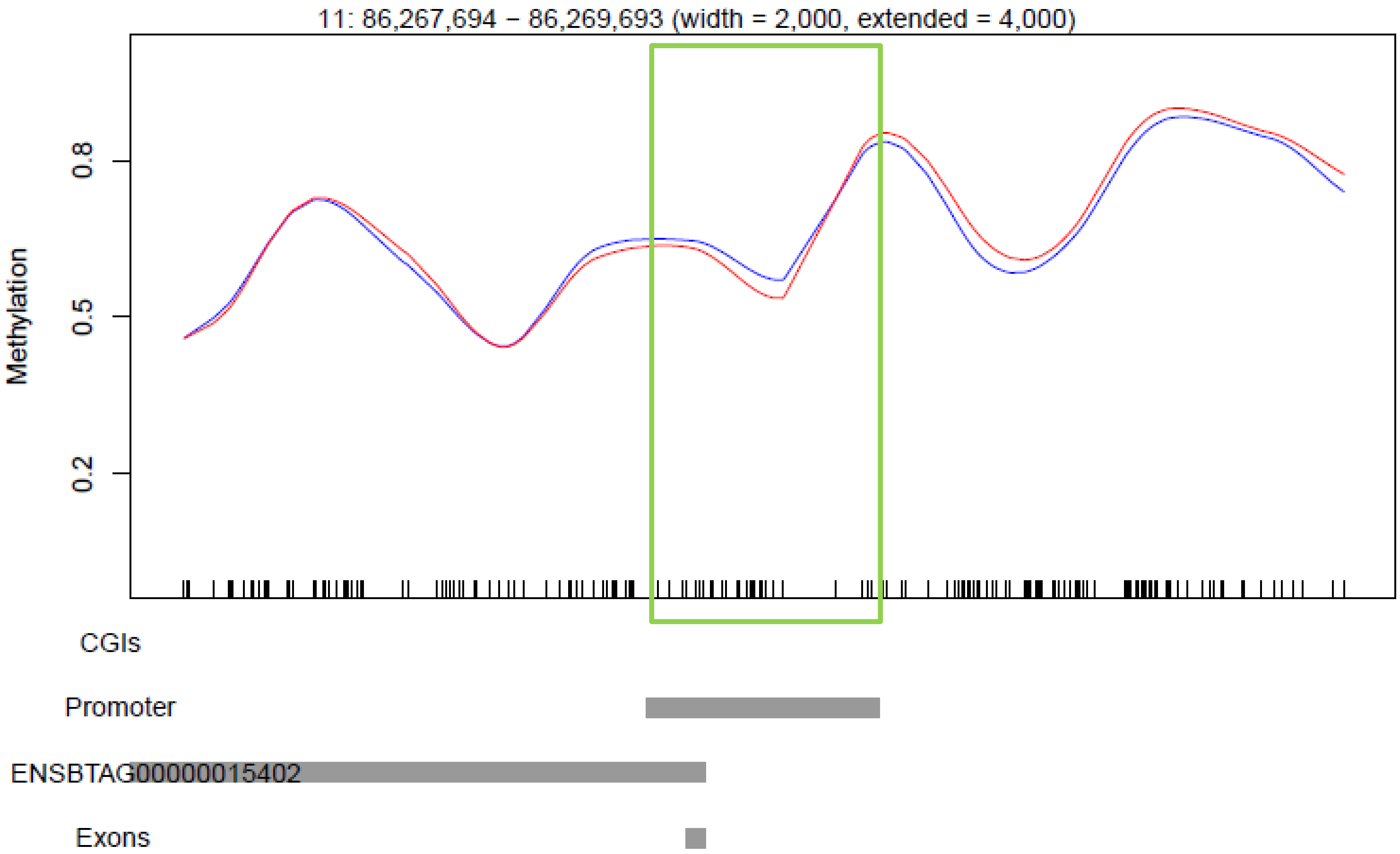

11: 86,267,694 − 86,269,693 (width = 2,000, extended = 4,000)
Methylation
0.8
0.5
0.2
CGIs
Promoter
ENSBTAG00000015402
Exons

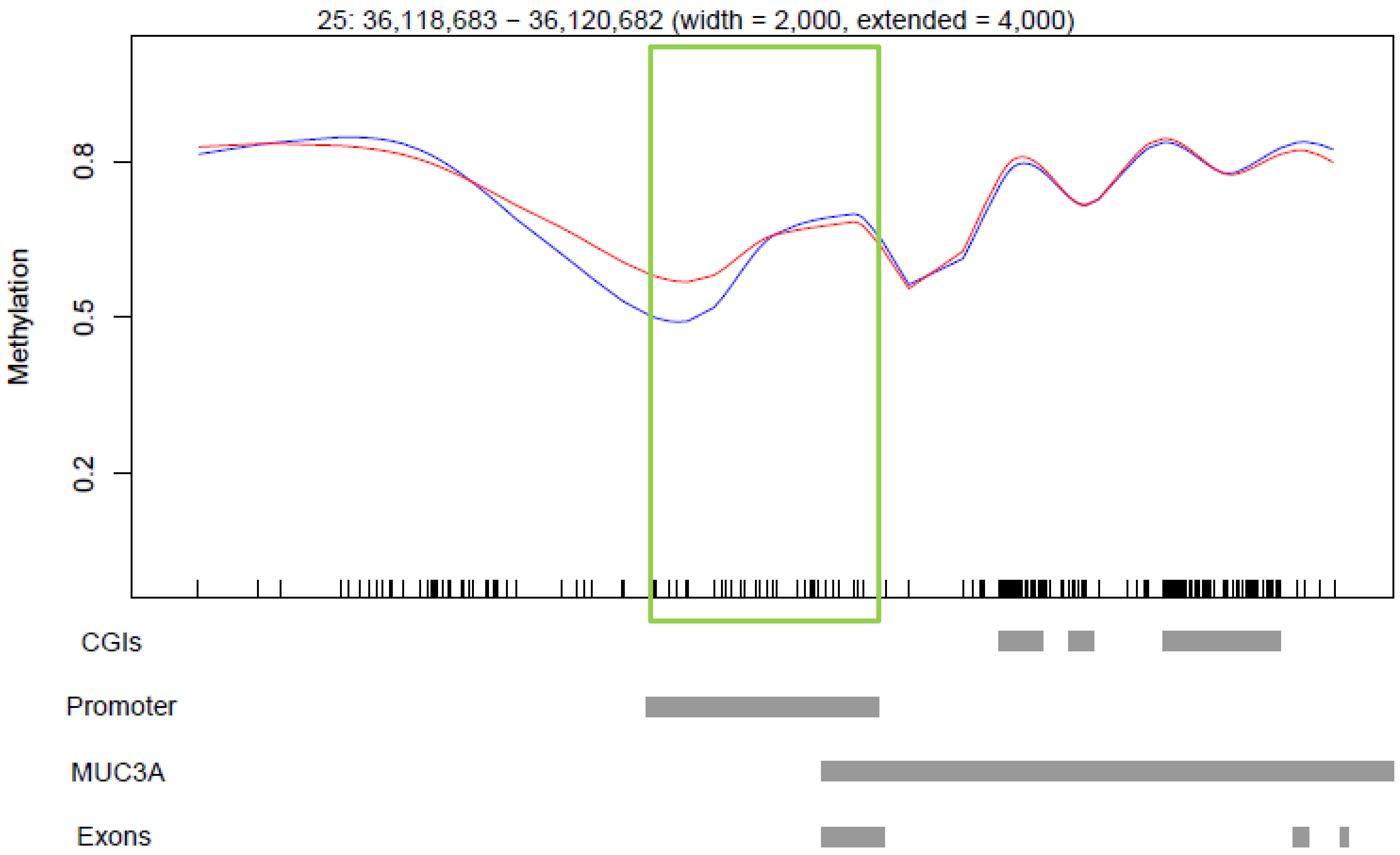

25: 36,118,683 − 36,120,682 (width = 2,000, extended = 4,000)
Methylation
0.8
0.5
0.2
CGIs
Promoter
MUC3A
Exons

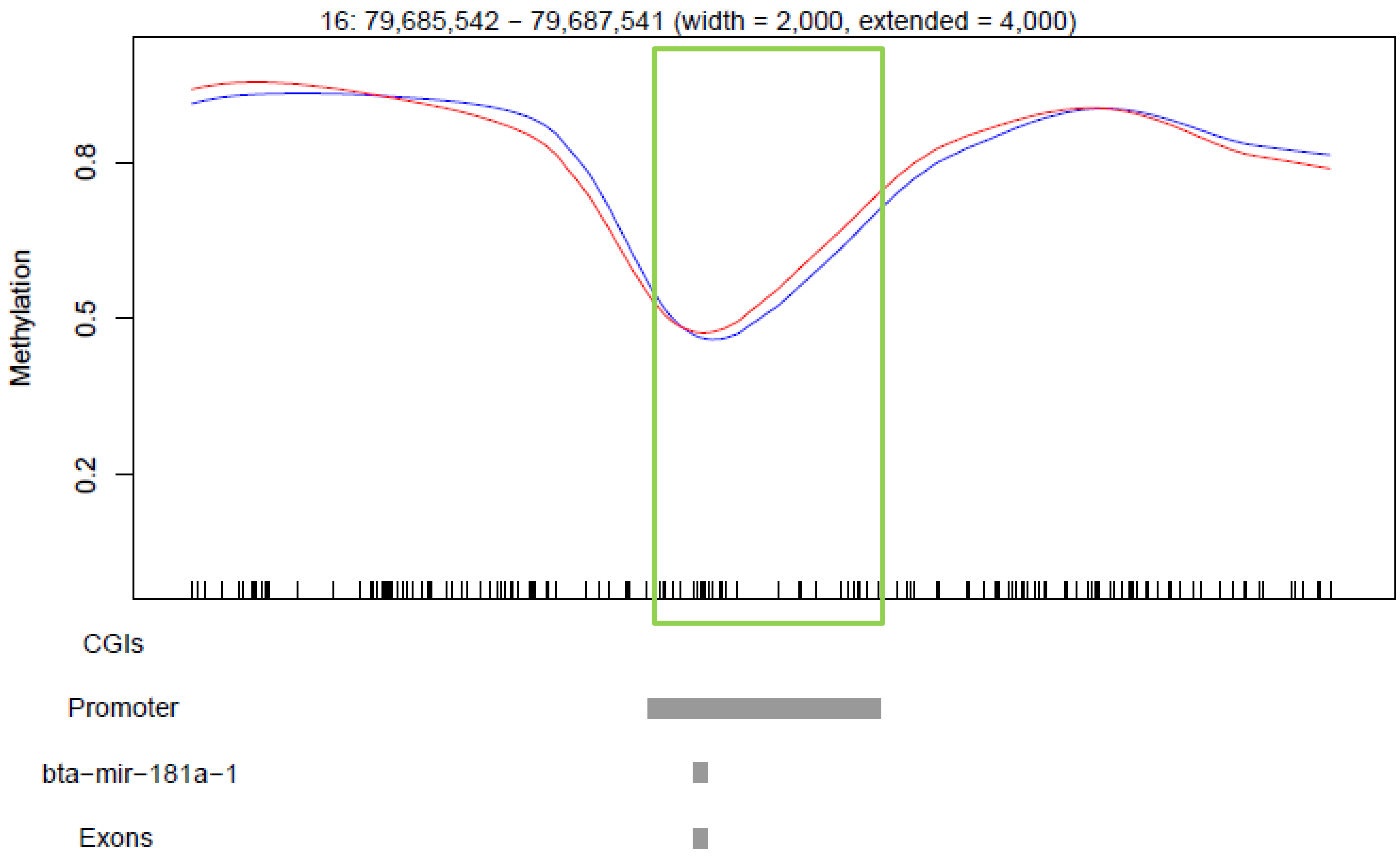

16: 79,685,542 – 79,687,541 (width = 2,000, extended = 4,000)
Methylation
0.8
0.5
0.2
CGIs
Promoter
bta–mir–181a–1
Exons

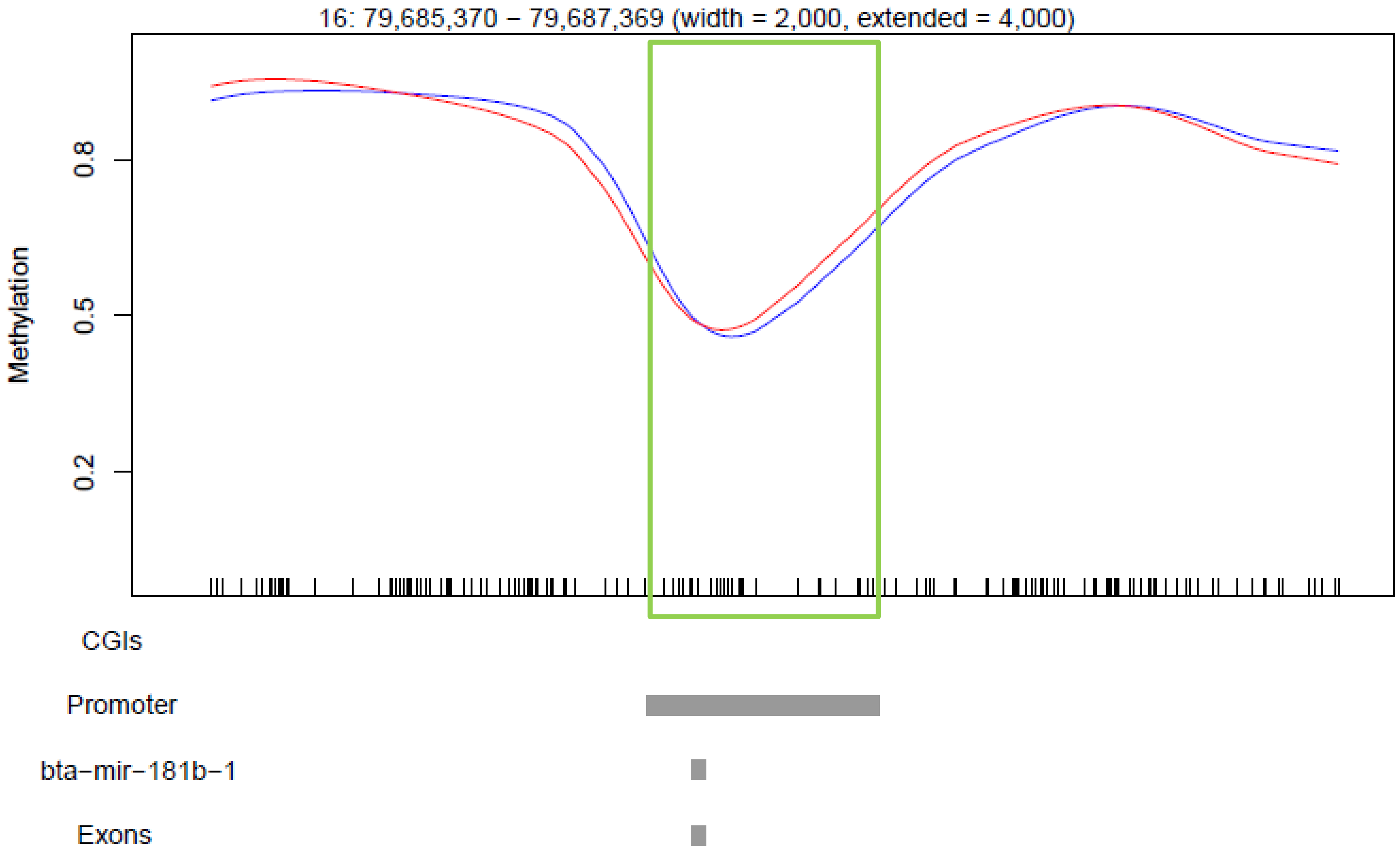
16: 79,685,370 − 79,687,369 (width = 2,000, extended = 4,000)
Methylation
0.8
0.5
0.2
CGIs
Promoter
bta−mir−181b−1
Exons

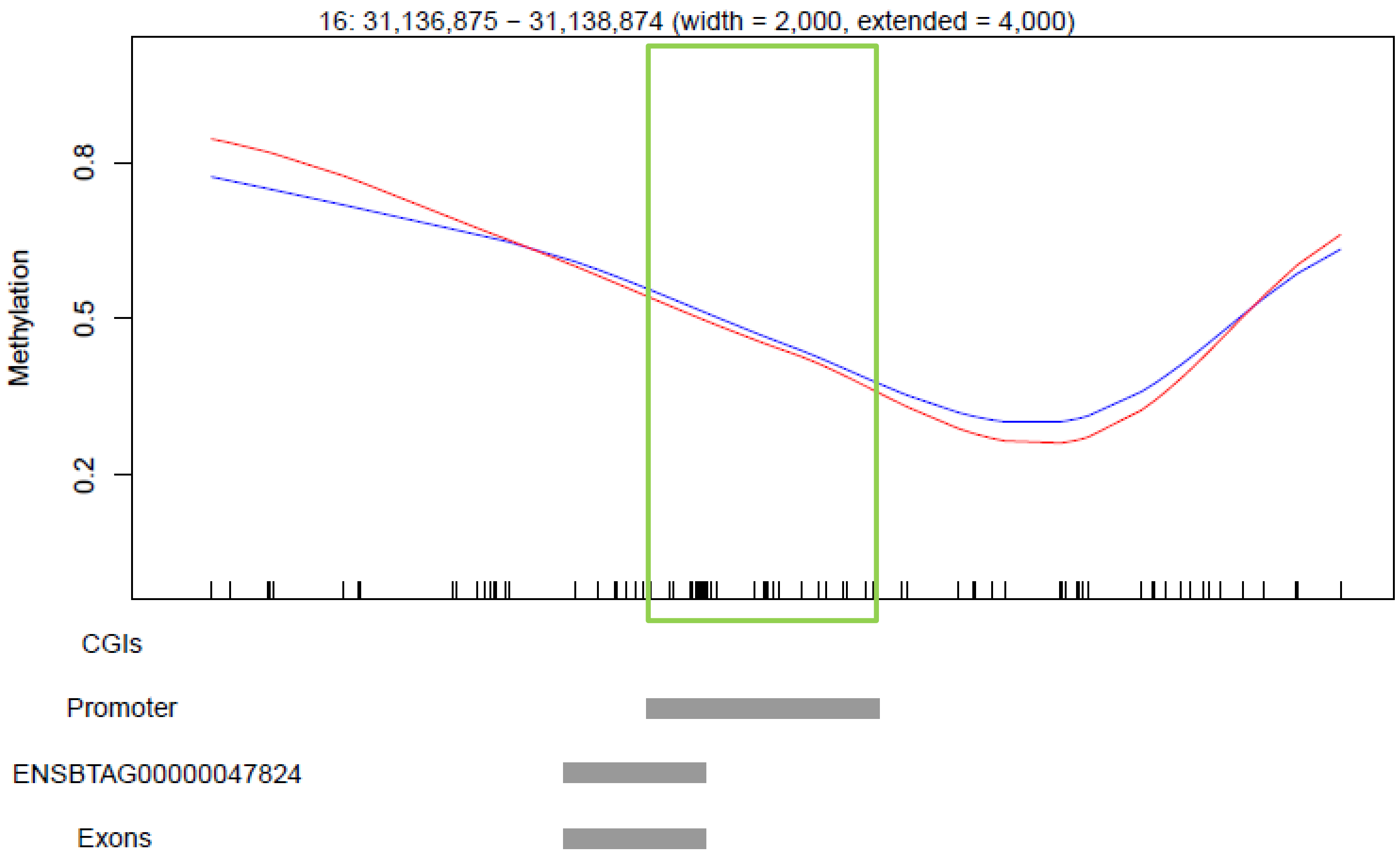

16: 31,136,875 – 31,138,874 (width = 2,000, extended = 4,000)
Methylation
0.8
0.5
0.2
CGIs
Promoter
ENSBTAG00000047824
Exons

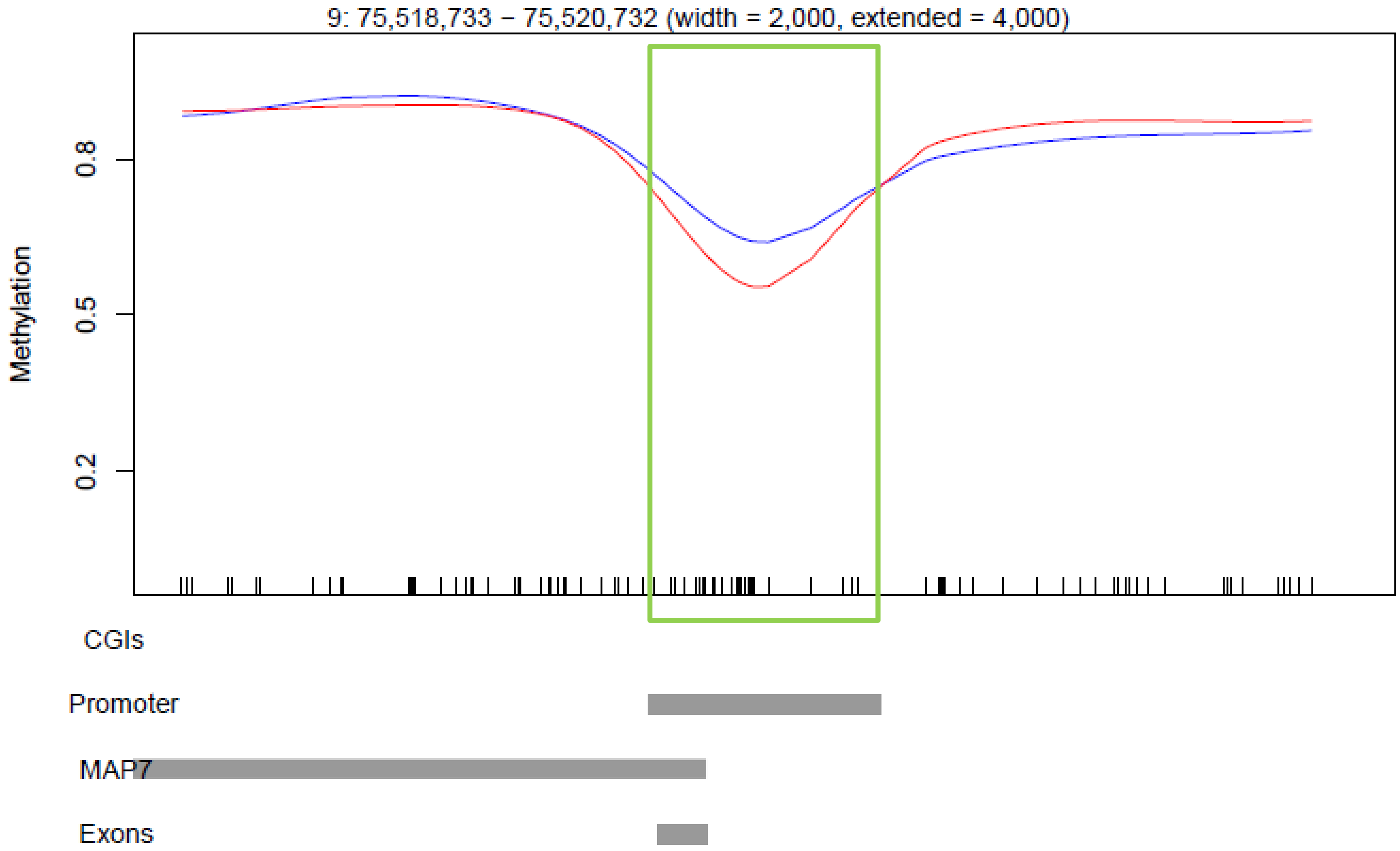
9: 75,518,733 − 75,520,732 (width = 2,000, extended = 4,000)
Methylation
0.2
0.5
0.8
CGIs
Promoter
MAP7
Exons

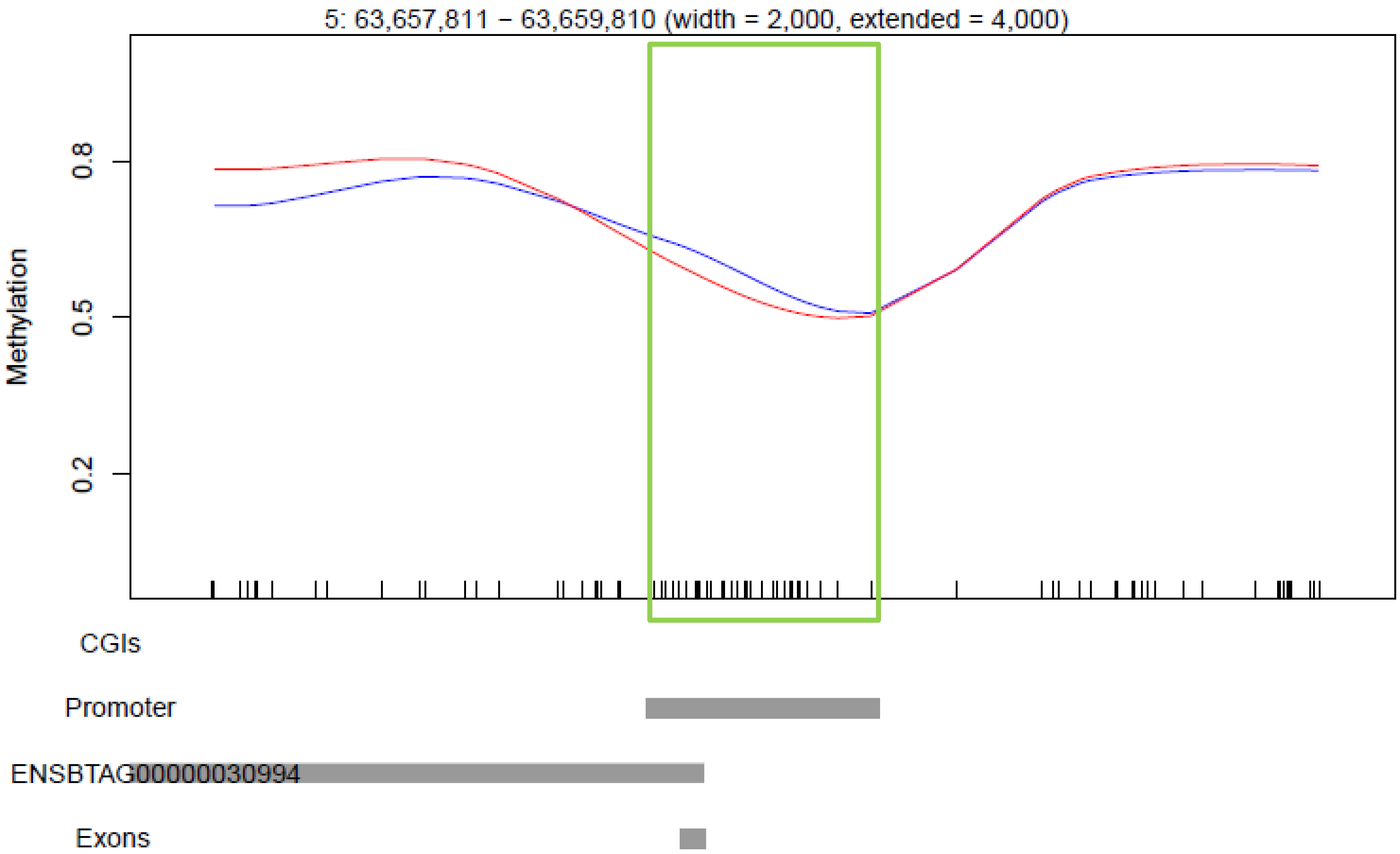
5: 63,657,811 − 63,659,810 (width = 2,000, extended = 4,000)
Methylation
0.8
0.5
0.2
CGIs
Promoter
ENSBTAG00000030994
Exons

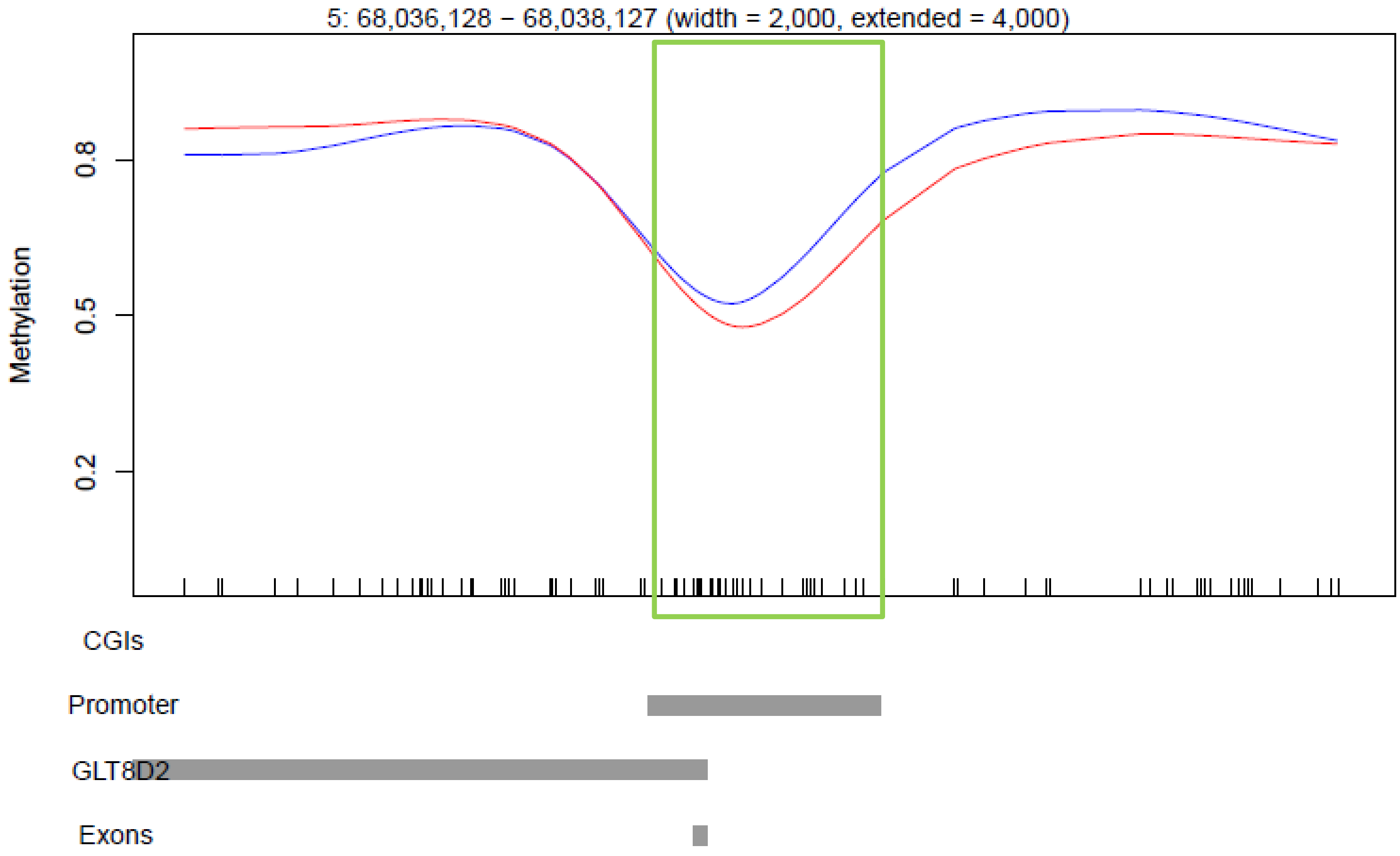

5: 68,036,128 – 68,038,127 (width = 2,000, extended = 4,000)
Methylation
0.8
0.5
0.2
CGIs
Promoter
GLT8D2
Exons

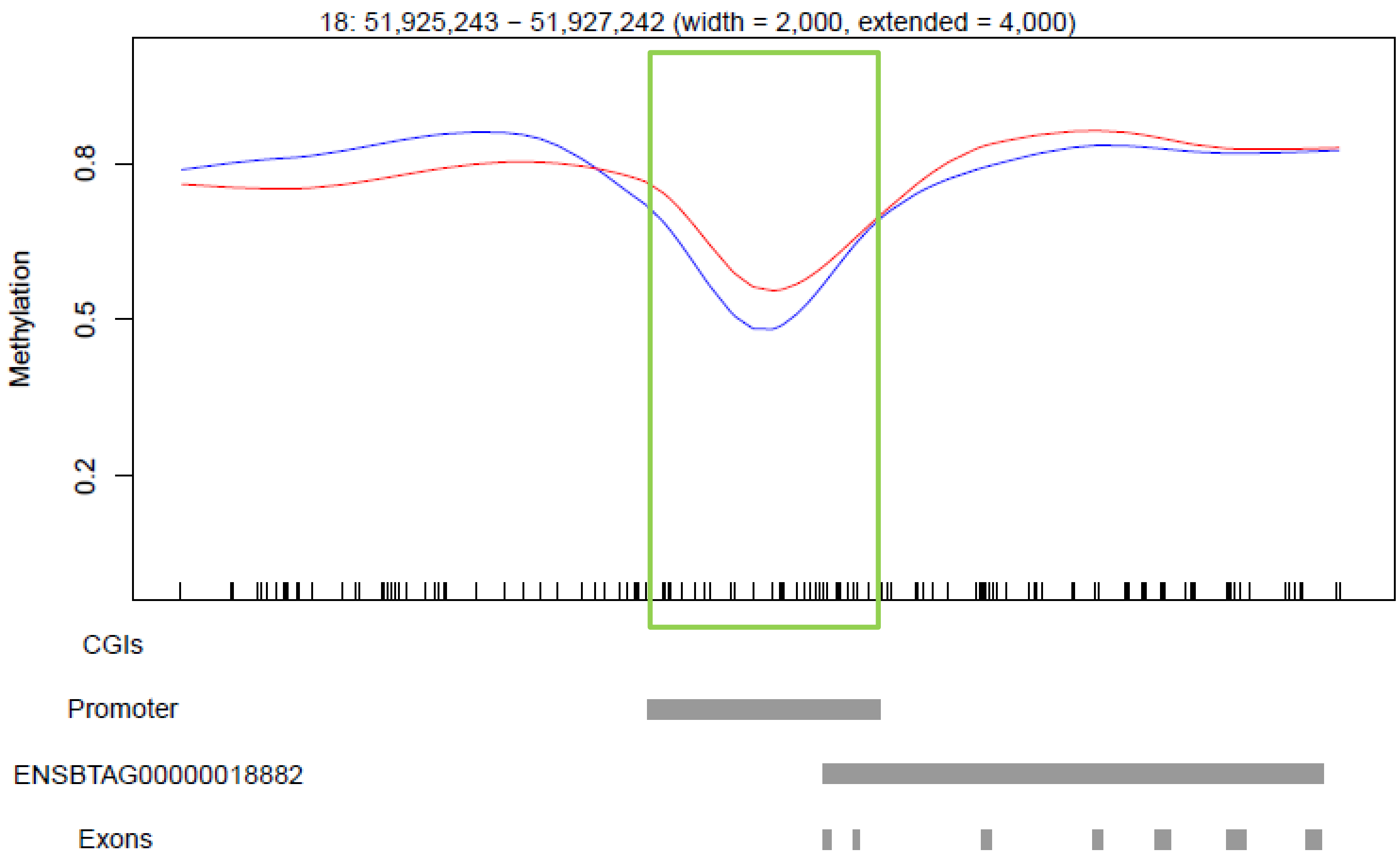
18: 51,925,243 − 51,927,242 (width = 2,000, extended = 4,000)
Methylation
0.8
0.5
0.2
CGIs
Promoter
ENSBTAG00000018882
Exons

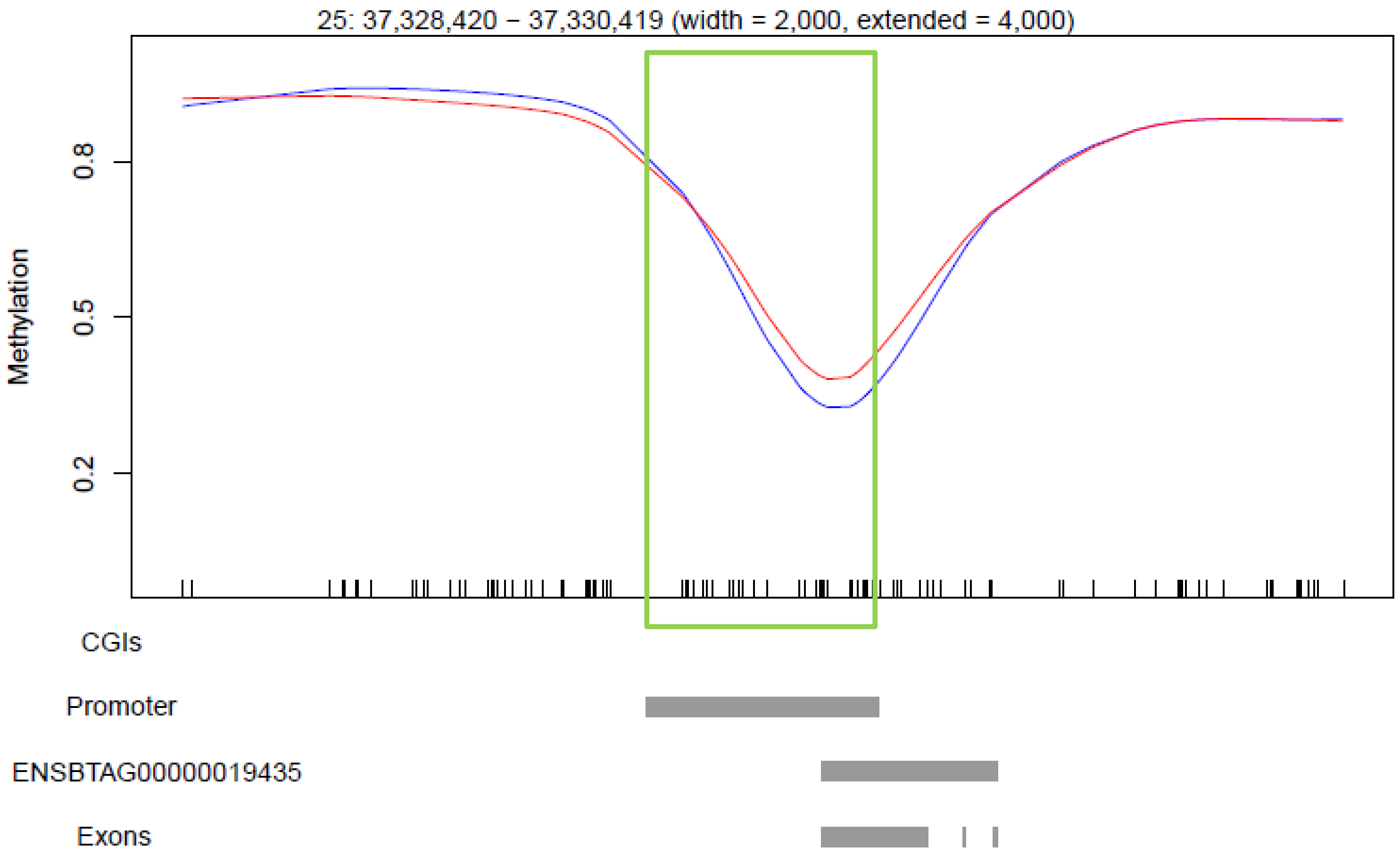
25: 37,328,420 − 37,330,419 (width = 2,000, extended = 4,000)
Methylation
0.8
0.5
0.2
CGIs
Promoter
ENSBTAG00000019435
Exons

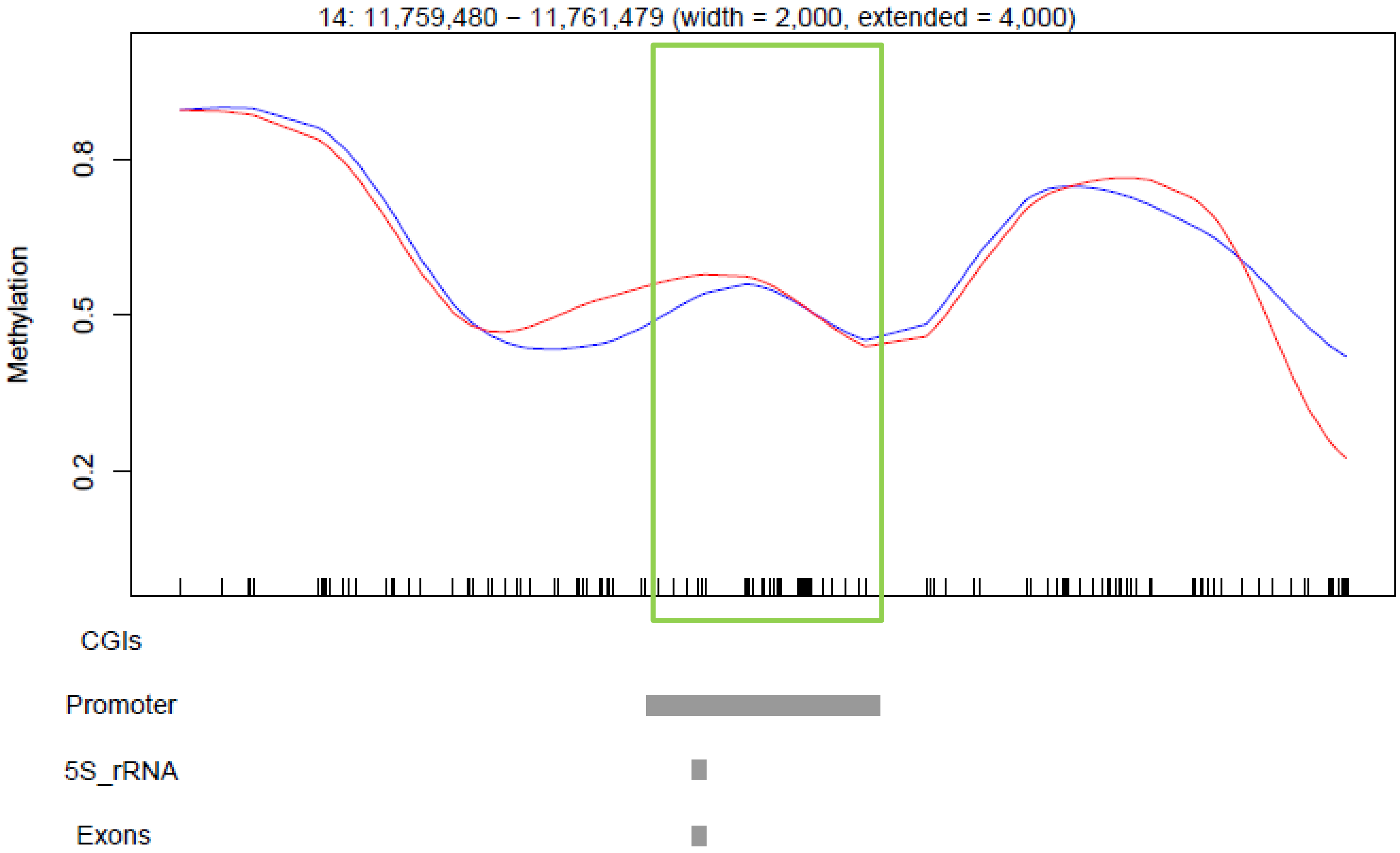

14: 11,759,480 − 11,761,479 (width = 2,000, extended = 4,000)
Methylation
0.8
0.5
0.2
CGIs
Promoter
5S_rRNA
Exons

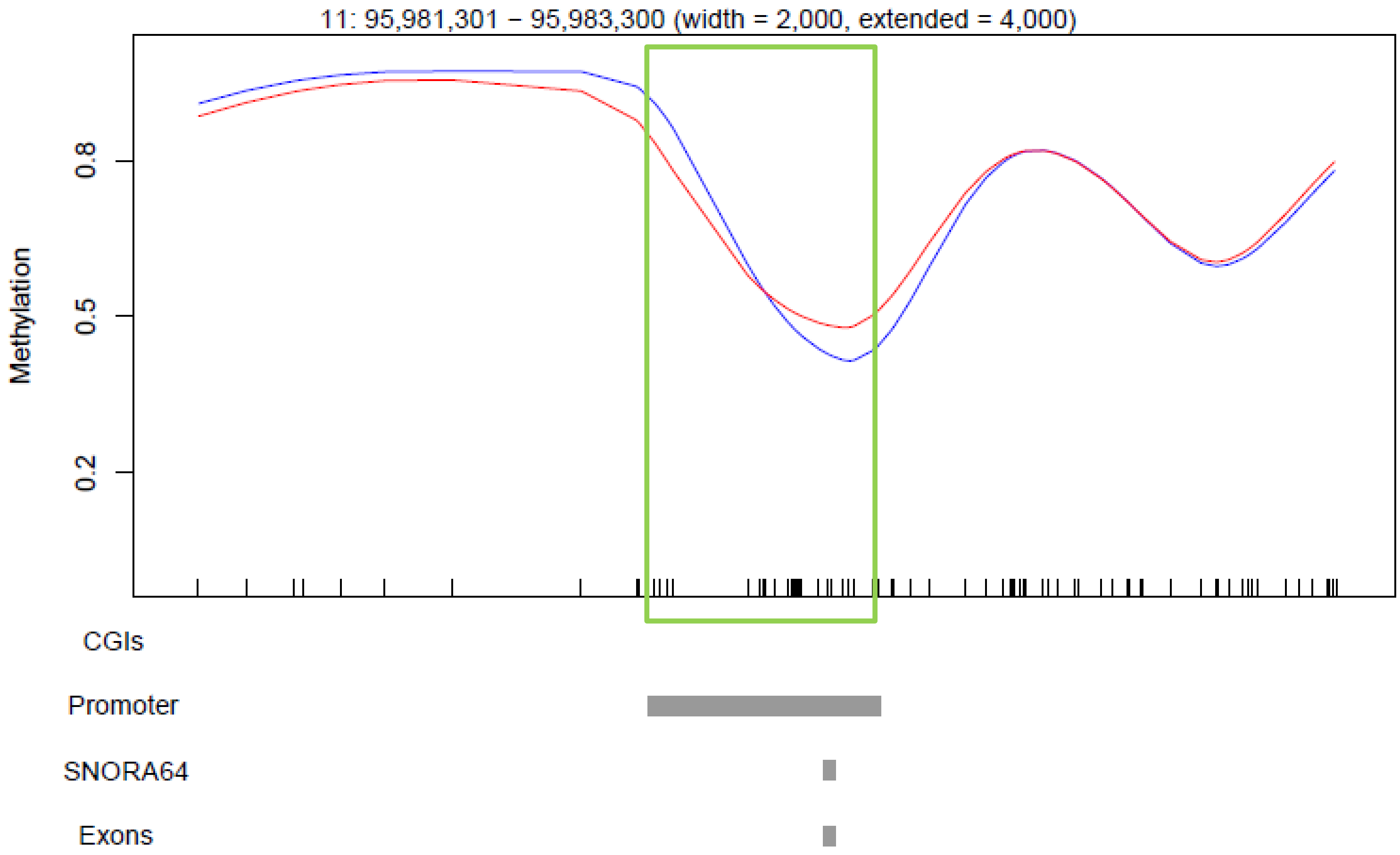
11: 95,981,301 – 95,983,300 (width = 2,000, extended = 4,000)
Methylation
0.2
0.5
0.8
CGIs
Promoter
SNORA64
Exons

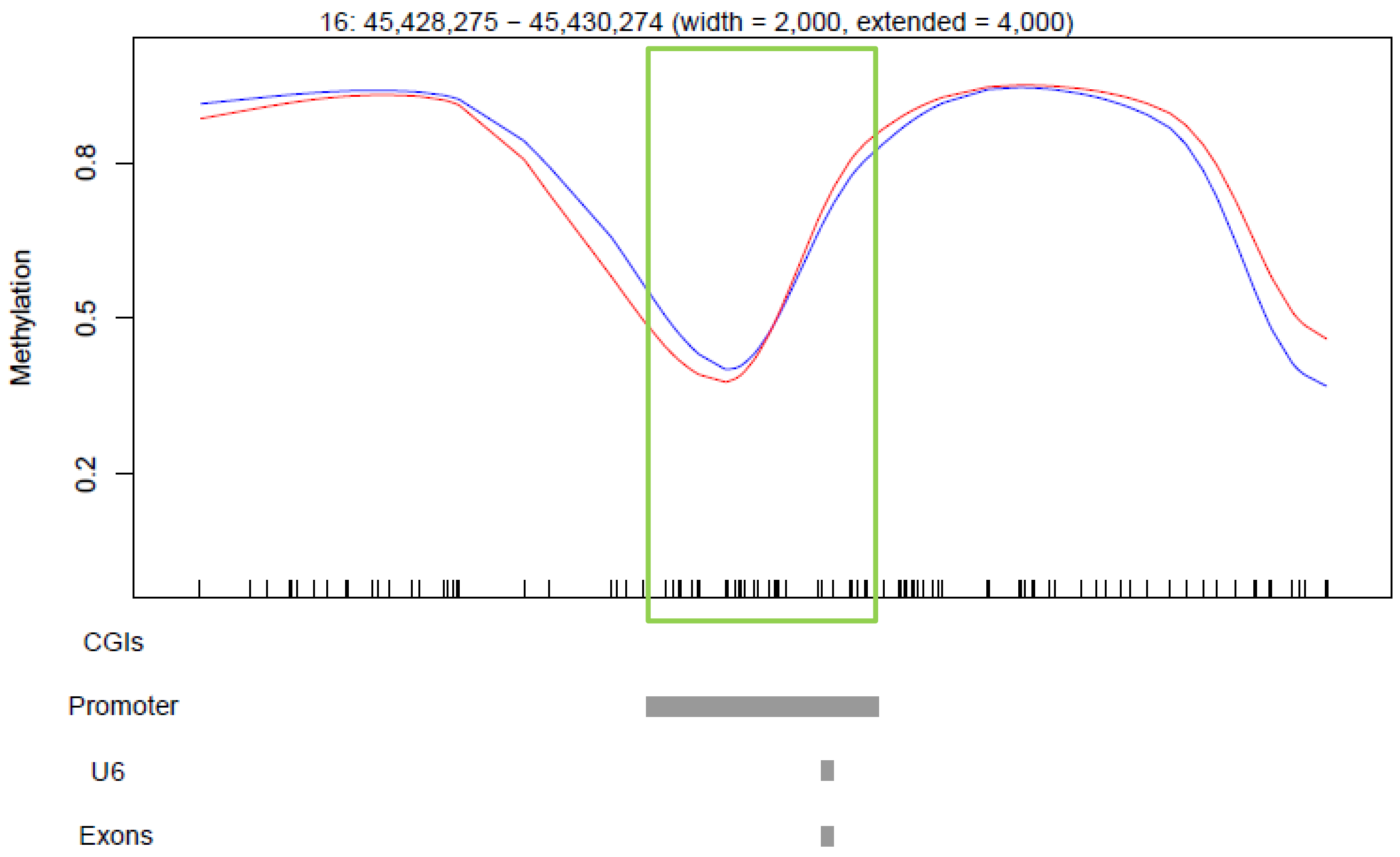

16: 45,428,275 – 45,430,274 (width = 2,000, extended = 4,000)
Methylation
0.2
0.5
0.8
CGIs
Promoter
U6
Exons

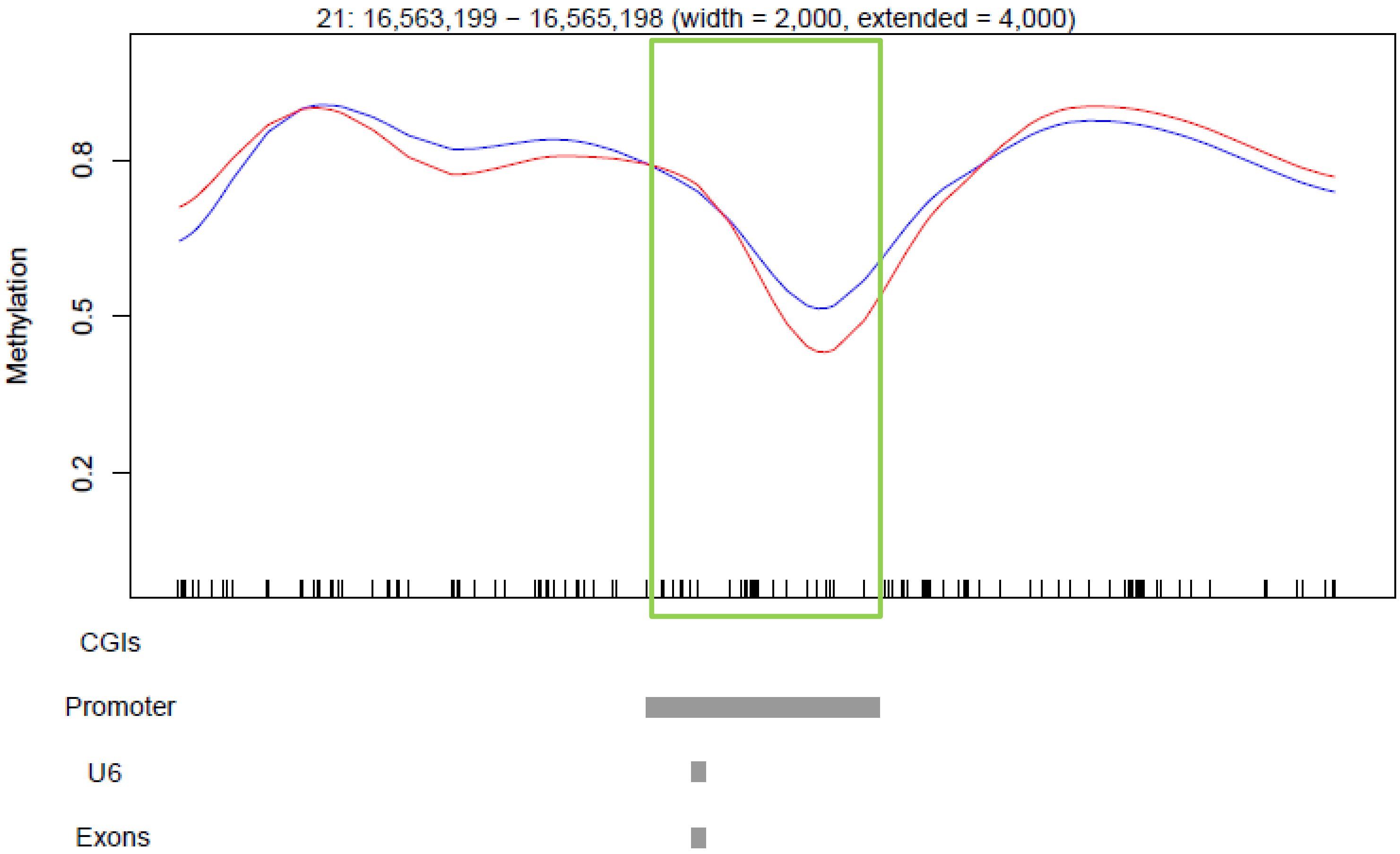

21: 16,563,199 – 16,565,198 (width = 2,000, extended = 4,000)
Methylation
0.2
0.5
0.8
CGIs
Promoter
U6
Exons

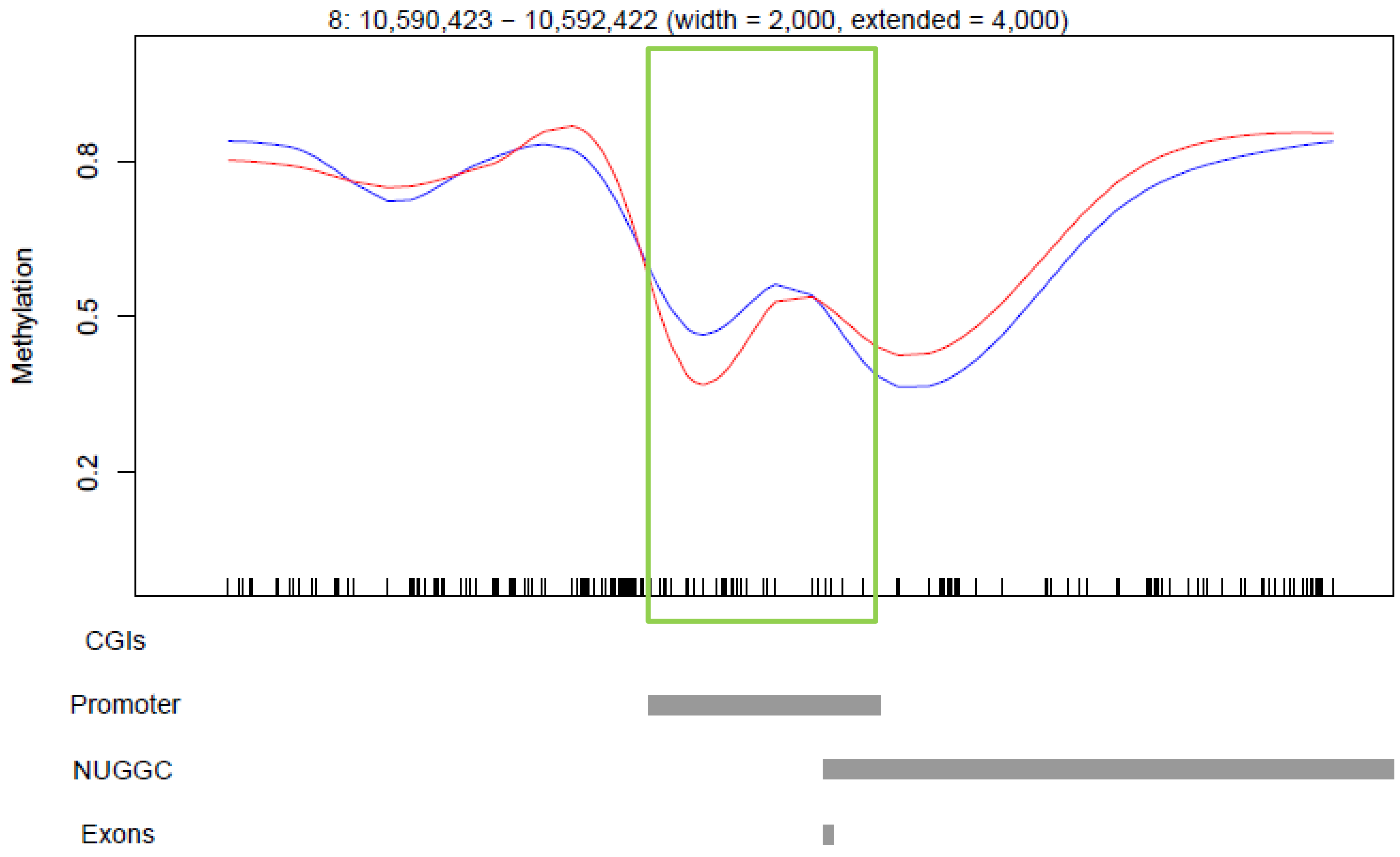
8: 10,590,423 – 10,592,422 (width = 2,000, extended = 4,000)
Methylation
0.8
0.5
0.2
CGIs
Promoter
NUGGC
Exons

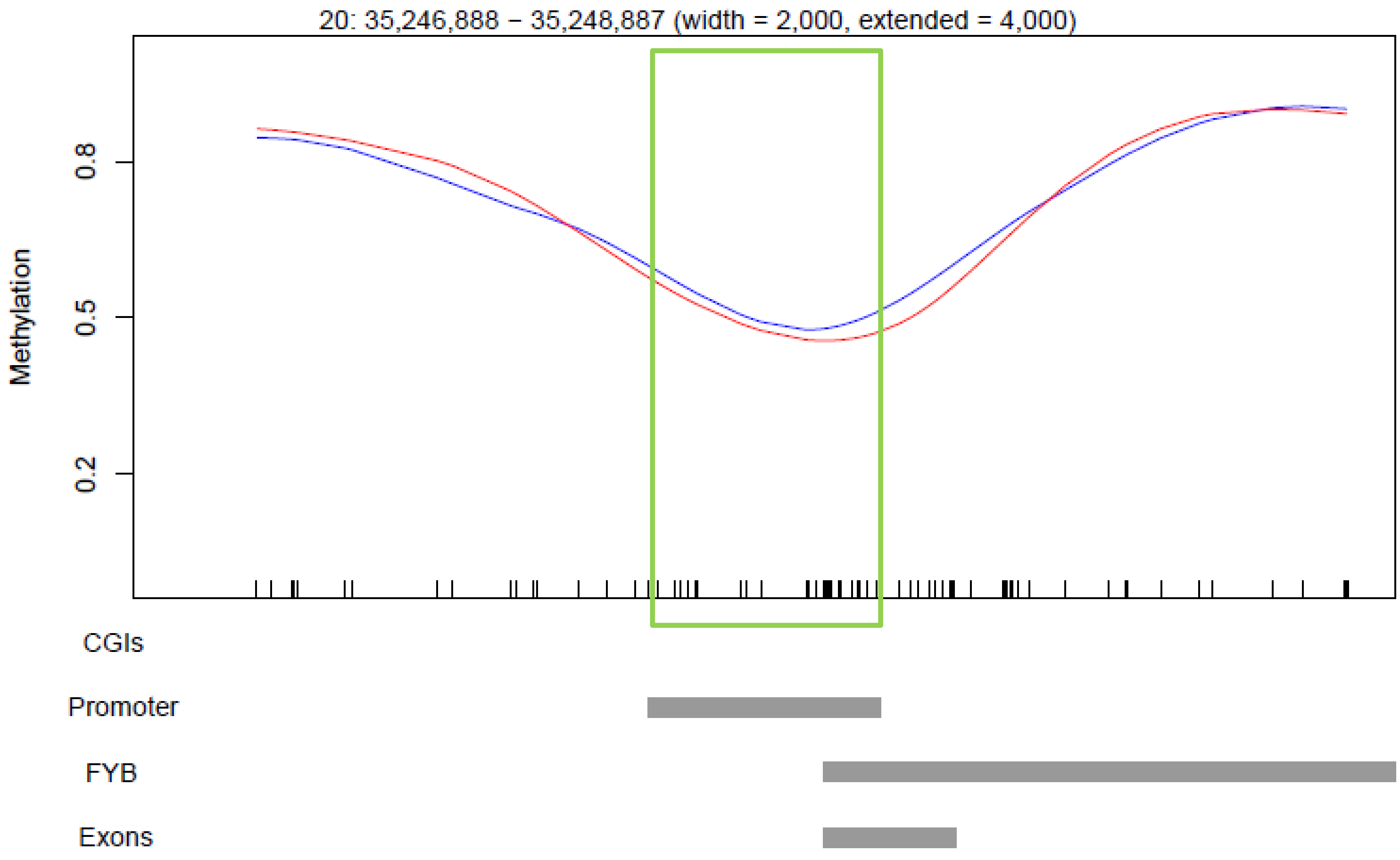

20: 35,246,888 – 35,248,887 (width = 2,000, extended = 4,000)
Methylation
0.8
0.5
0.2
CGIs
Promoter
FYB
Exons

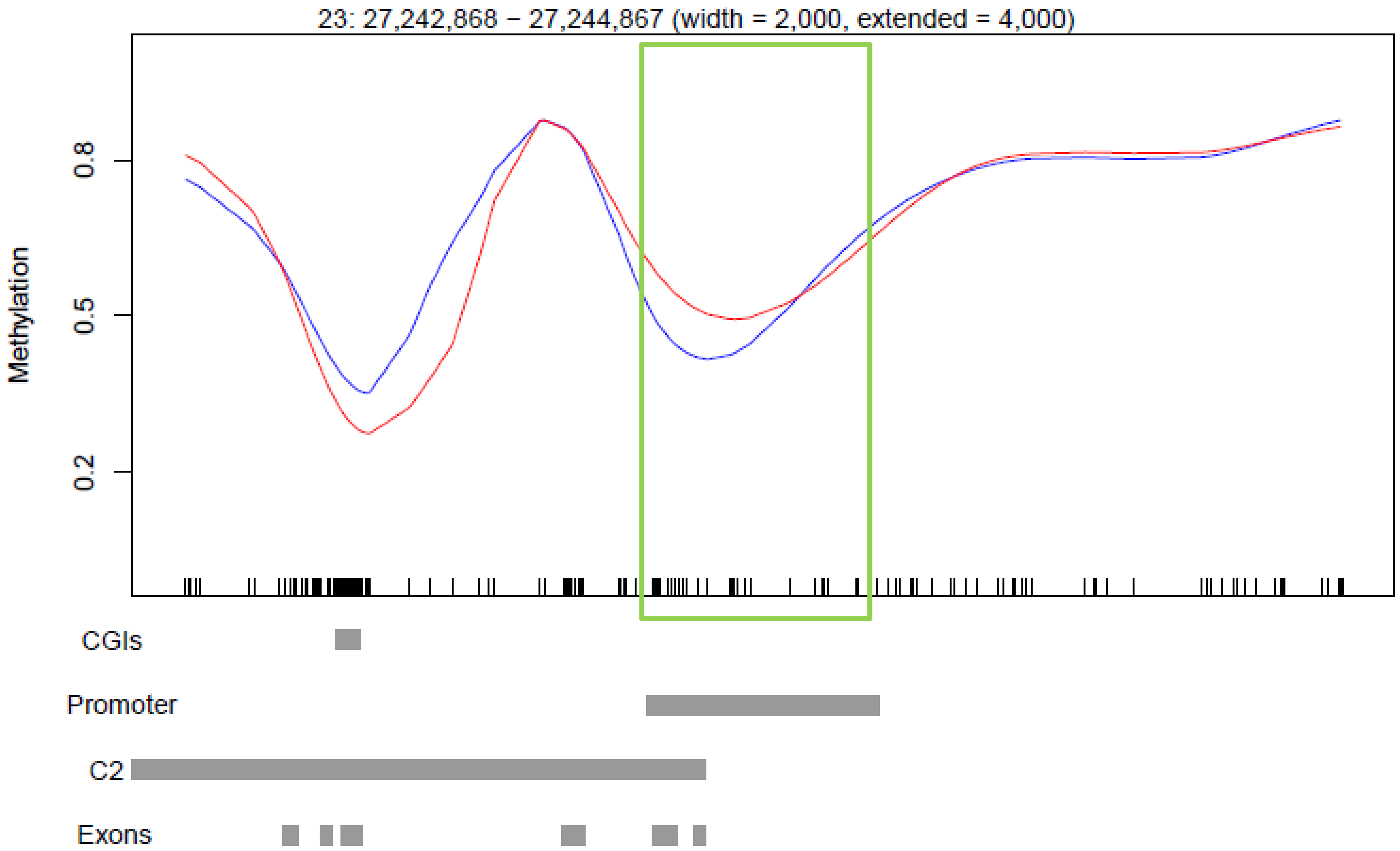
23: 27,242,868 – 27,244,867 (width = 2,000, extended = 4,000)
Methylation
0.2
0.5
0.8
CGIs
Promoter
C2
Exons

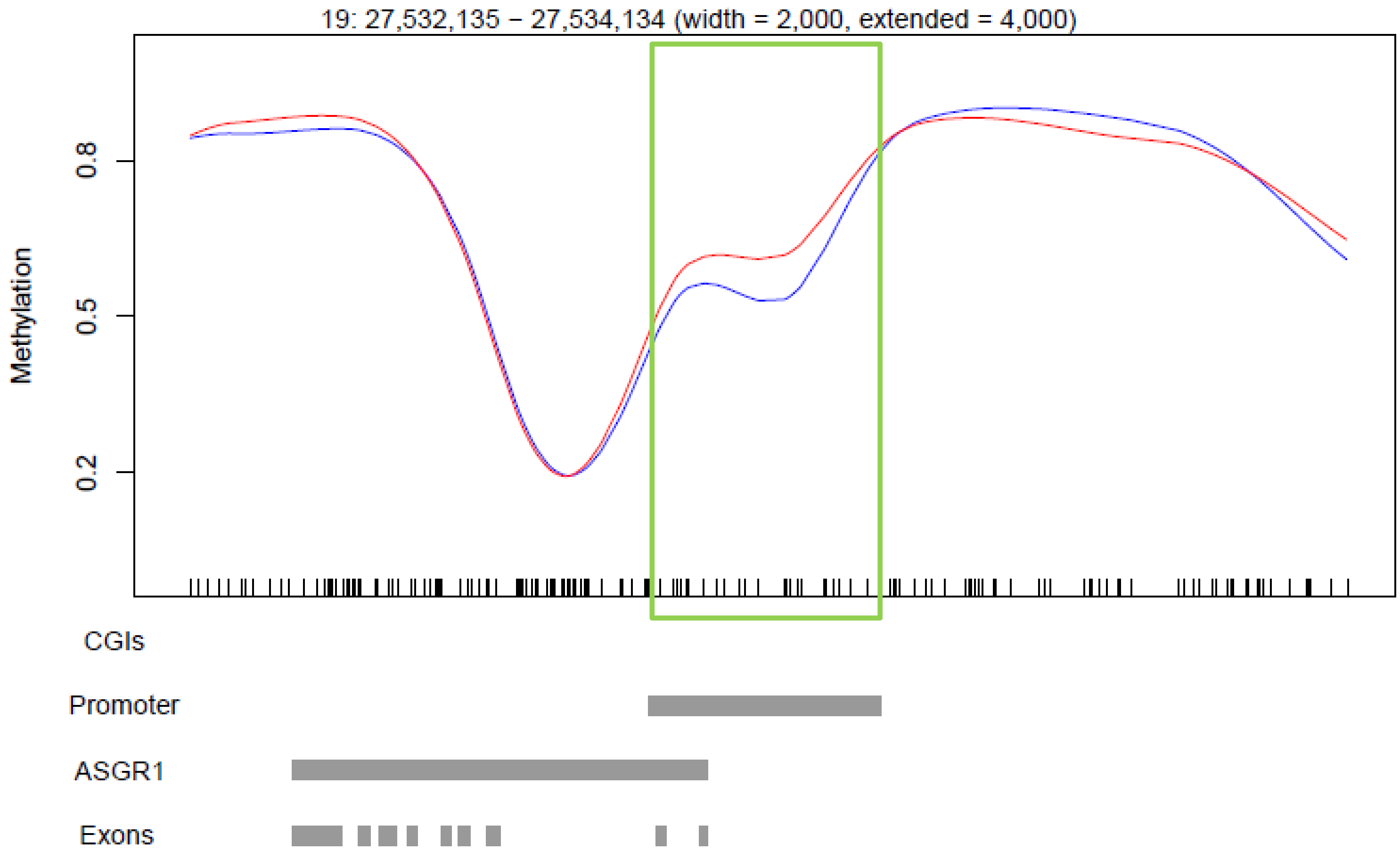

19: 27,532,135 – 27,534,134 (width = 2,000, extended = 4,000)
Methylation
0.8
0.5
0.2
CGIs
Promoter
ASGR1
Exons

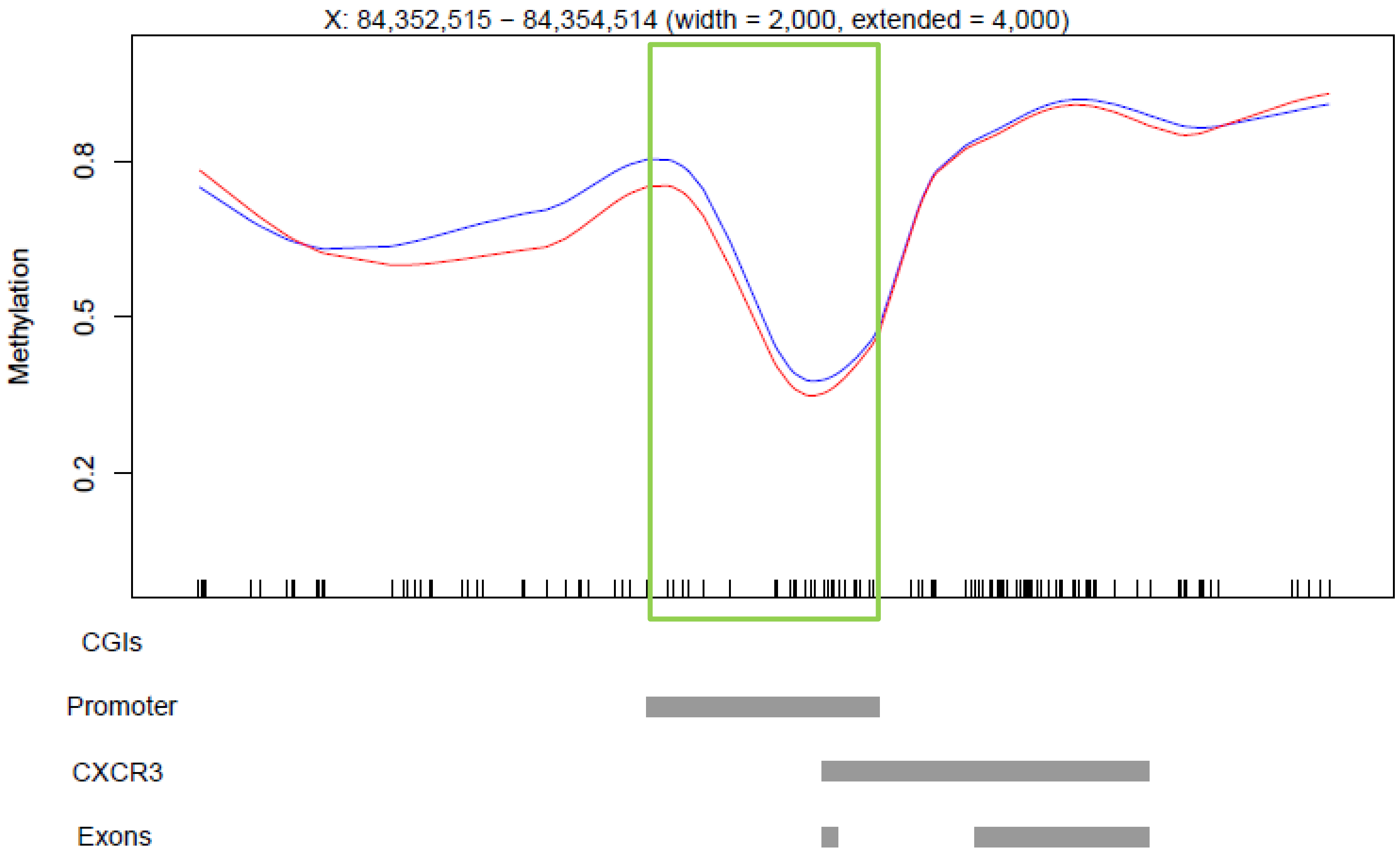
X: 84,352,515 – 84,354,514 (width = 2,000, extended = 4,000)
Methylation
0.8
0.5
0.2
CGIs
Promoter
CXCR3
Exons

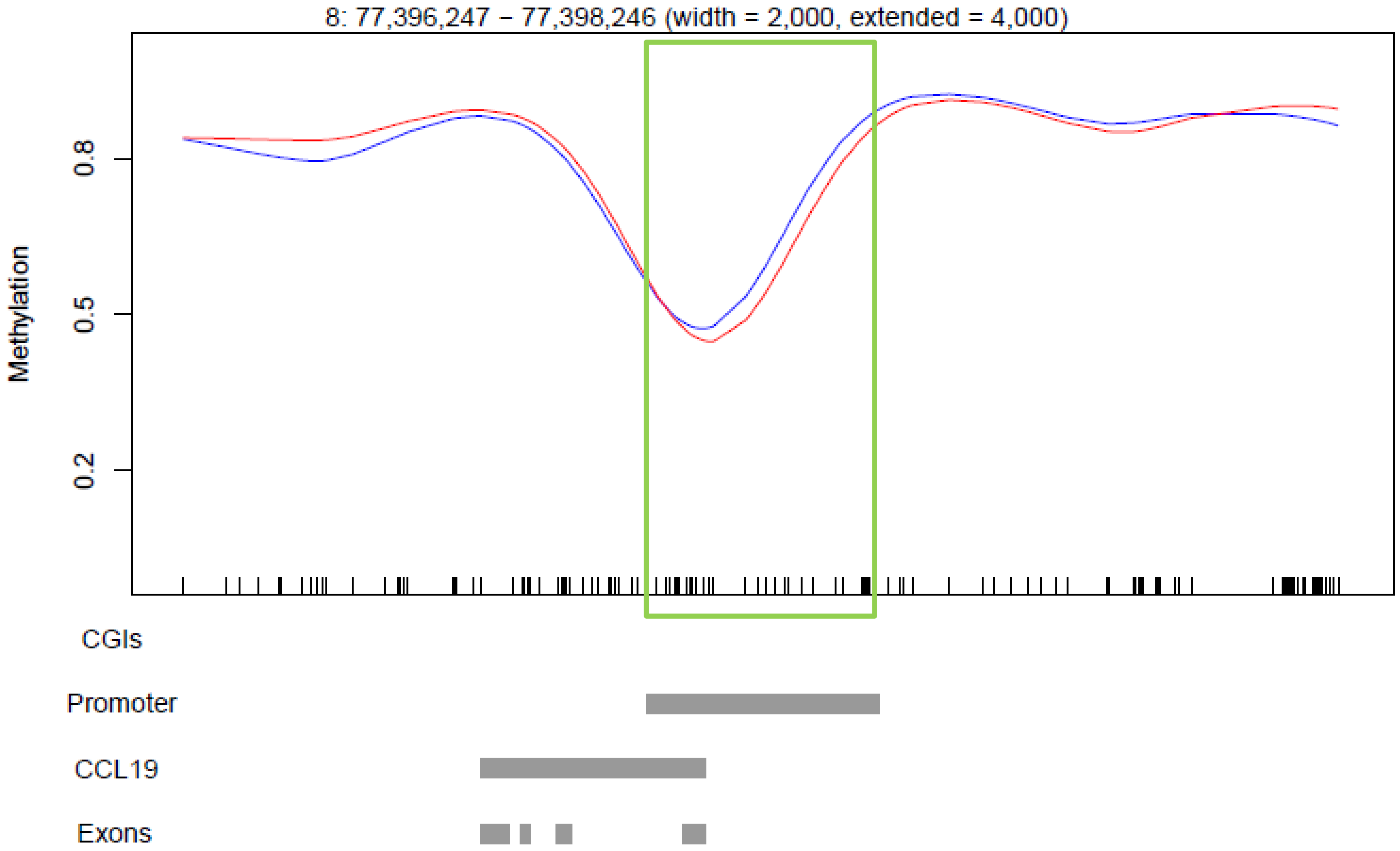
8: 77,396,247 − 77,398,246 (width = 2,000, extended = 4,000)
Methylation
0.8
0.5
0.2
CGIs
Promoter
CCL19
Exons

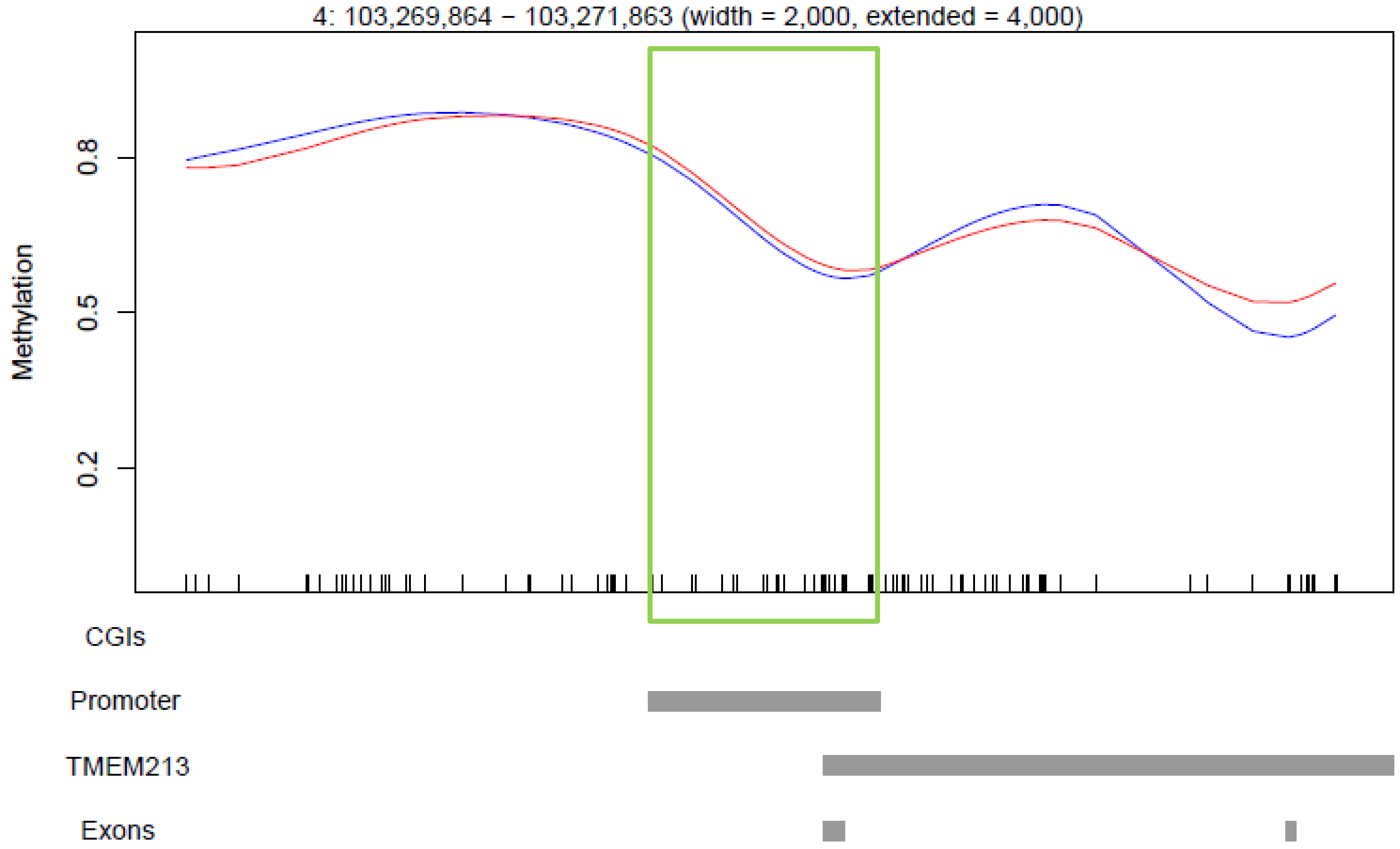

4: 103,269,864 − 103,271,863 (width = 2,000, extended = 4,000)
Methylation
0.2
0.5
0.8
CGIs
Promoter
TMEM213
Exons

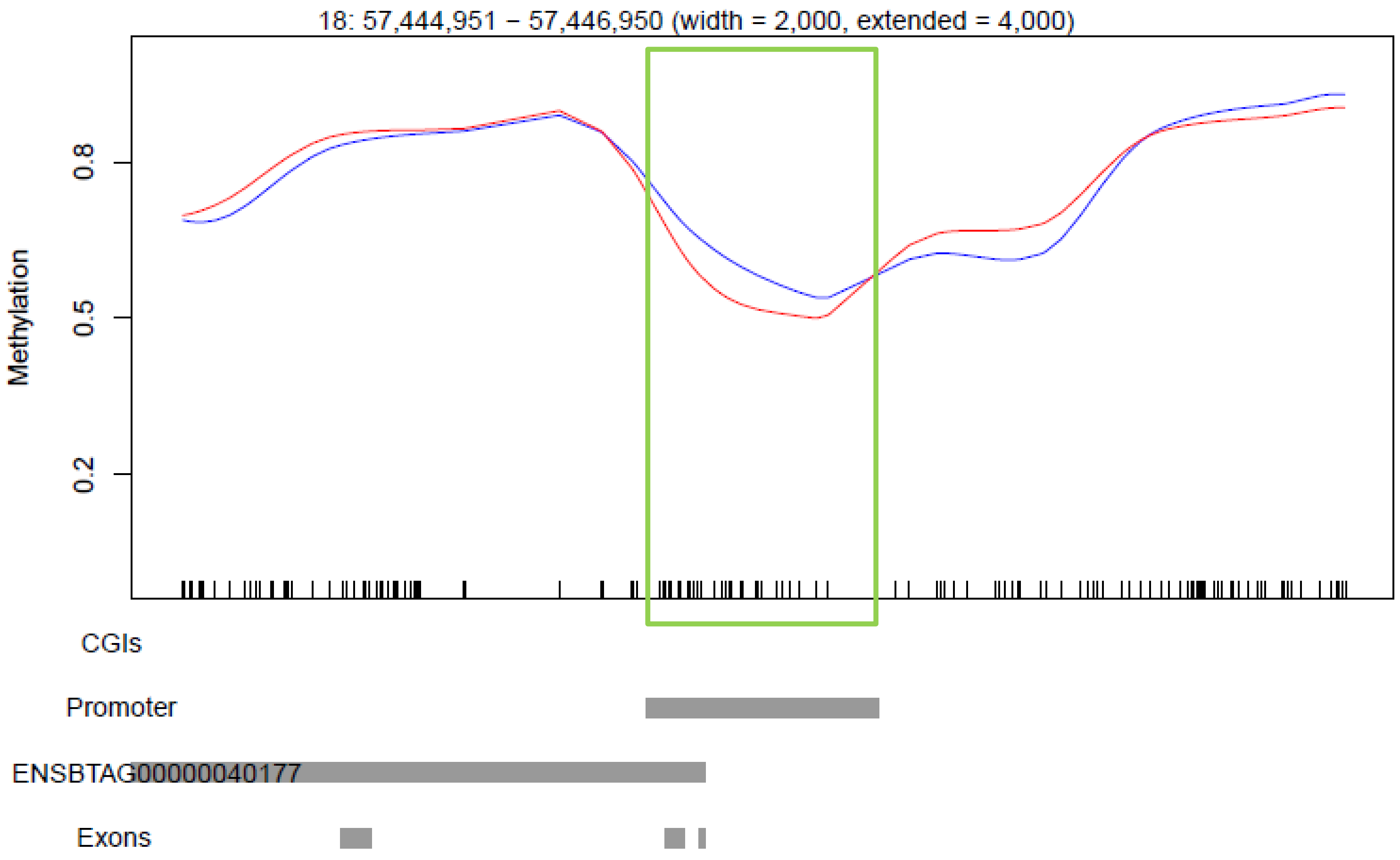
18: 57,444,951 − 57,446,950 (width = 2,000, extended = 4,000)
Methylation
0.2
0.5
0.8
CGIs
Promoter
ENSBTAG00000040177
Exons

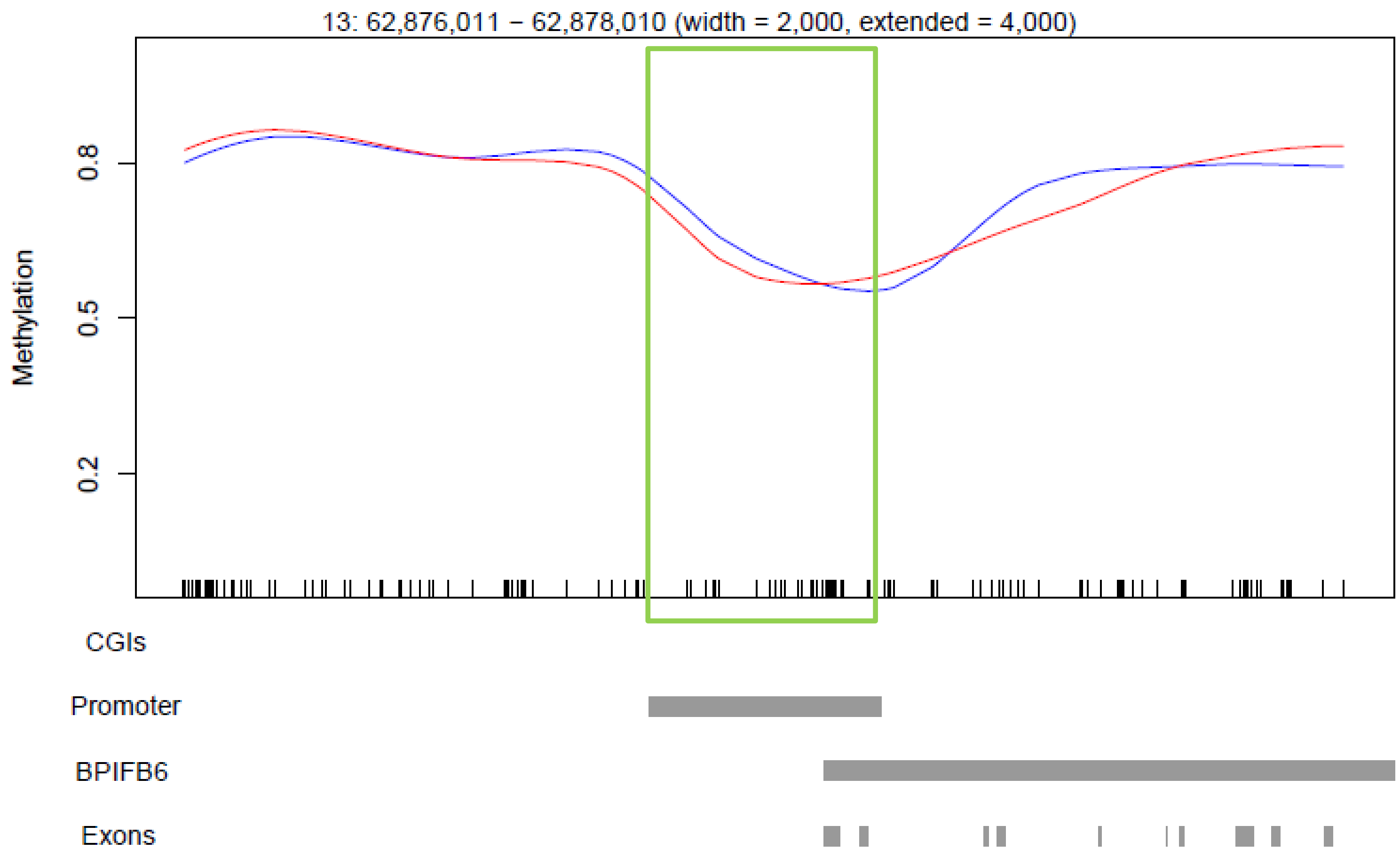
13: 62,876,011 − 62,878,010 (width = 2,000, extended = 4,000)
Methylation
0.2
0.5
0.8
CGIs
Promoter
BPIFB6
Exons

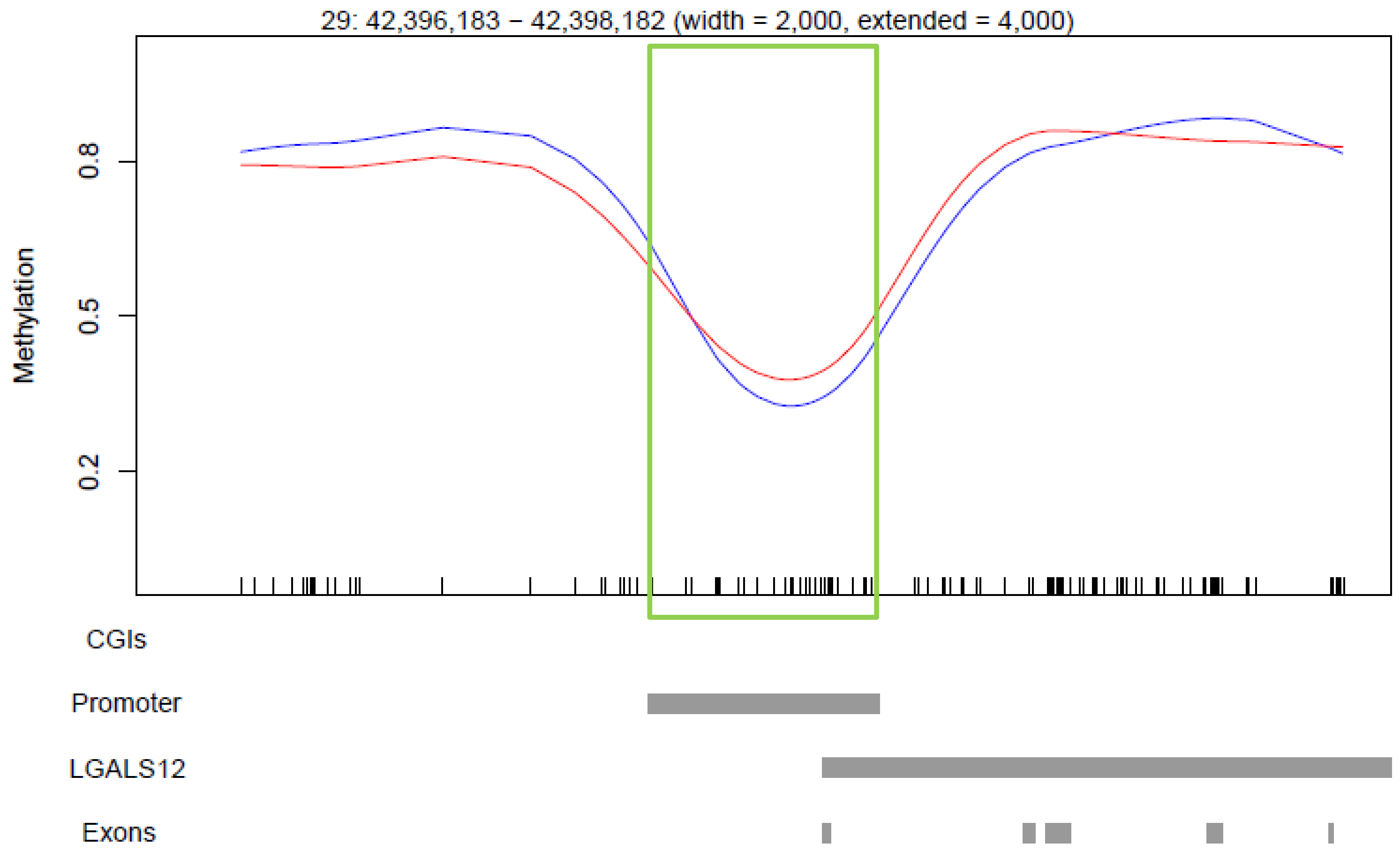
29: 42,396,183 − 42,398,182 (width = 2,000, extended = 4,000)
Methylation
0.8
0.5
0.2
CGIs
Promoter
LGALS12
Exons

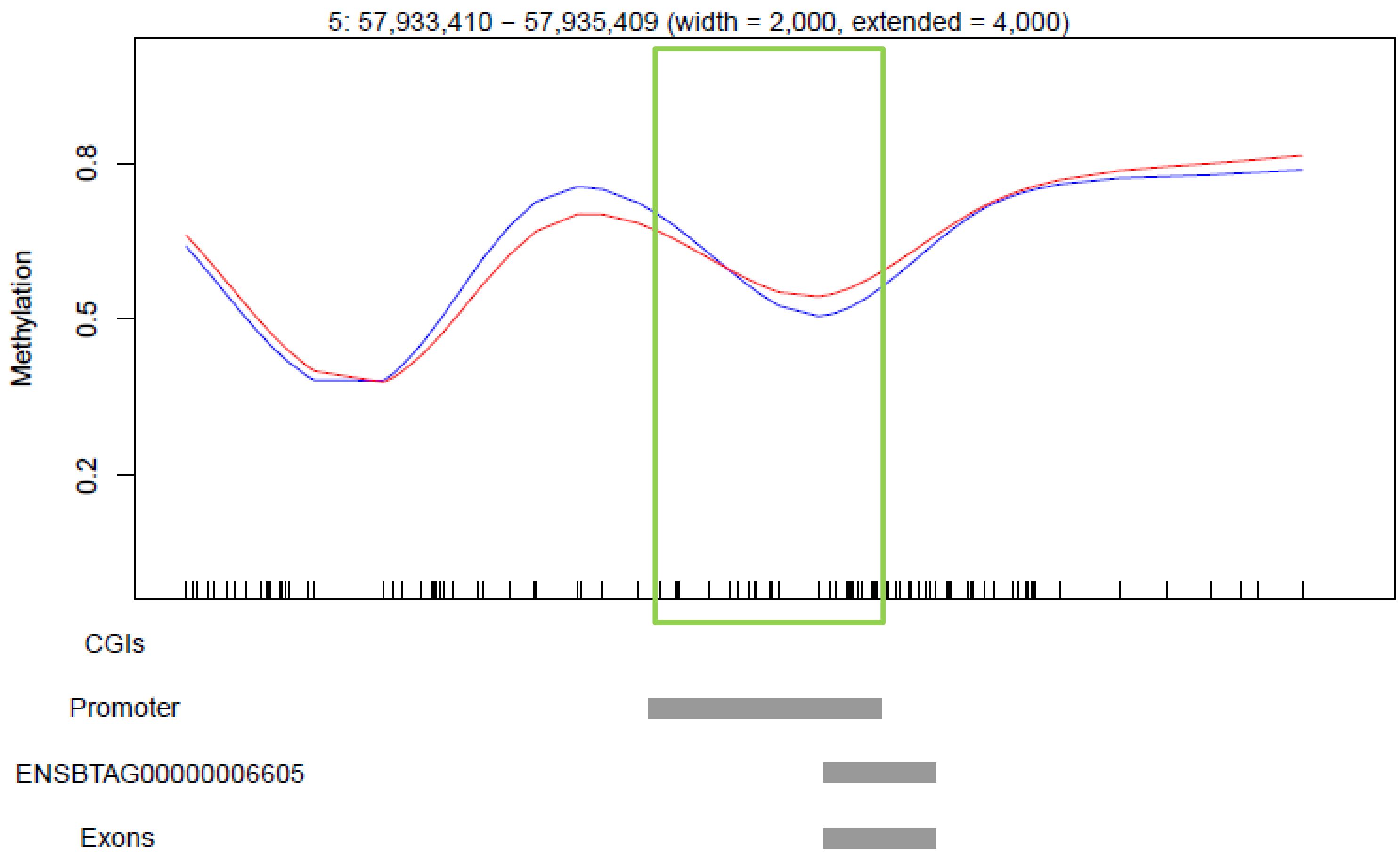
5: 57,933,410 − 57,935,409 (width = 2,000, extended = 4,000)
Methylation
0.8
0.5
0.2
CGIs
Promoter
ENSBTAG00000006605
Exons

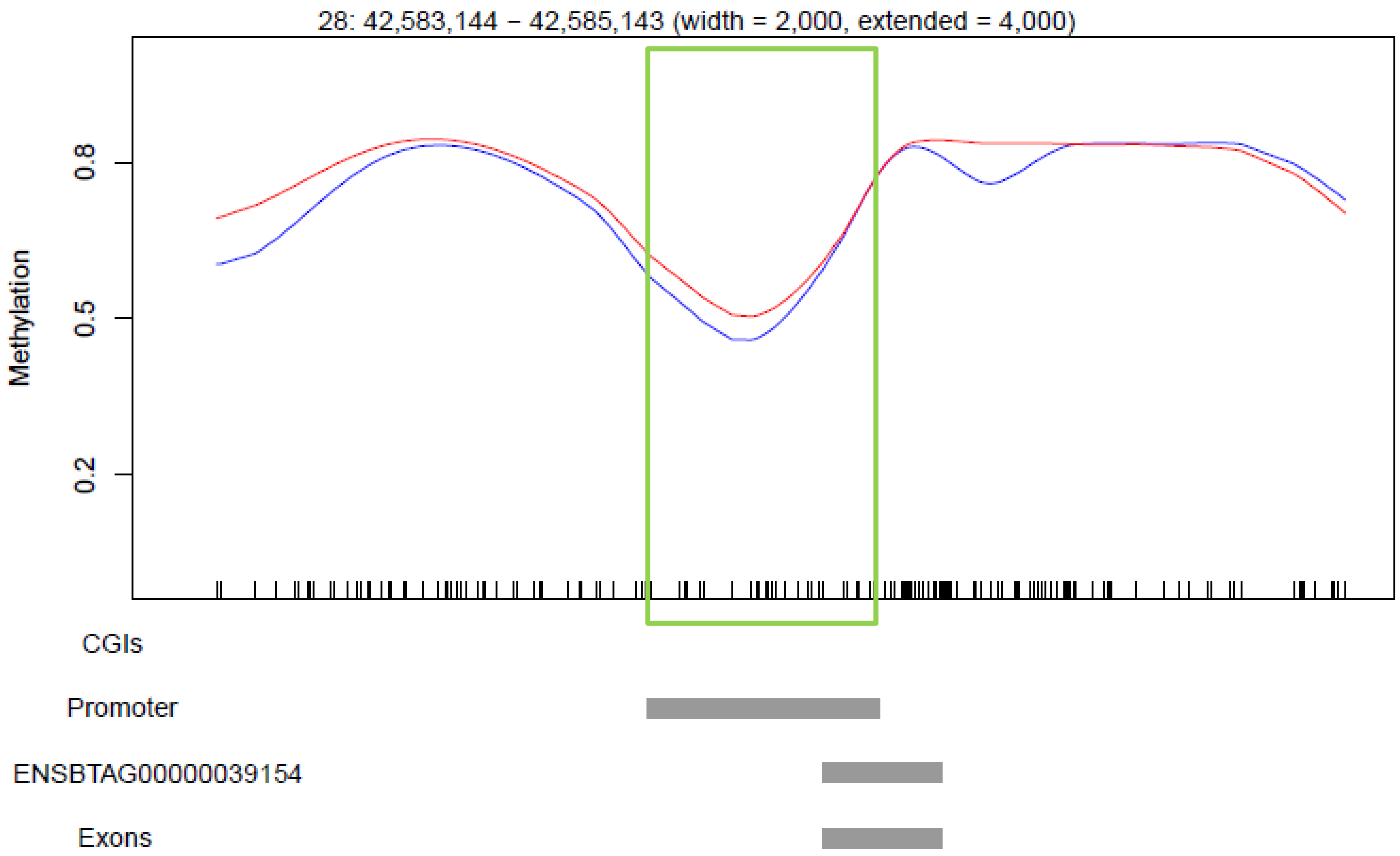
28: 42,583,144 − 42,585,143 (width = 2,000, extended = 4,000)
Methylation
0.8
0.5
0.2
CGIs
Promoter
ENSBTAG00000039154
Exons

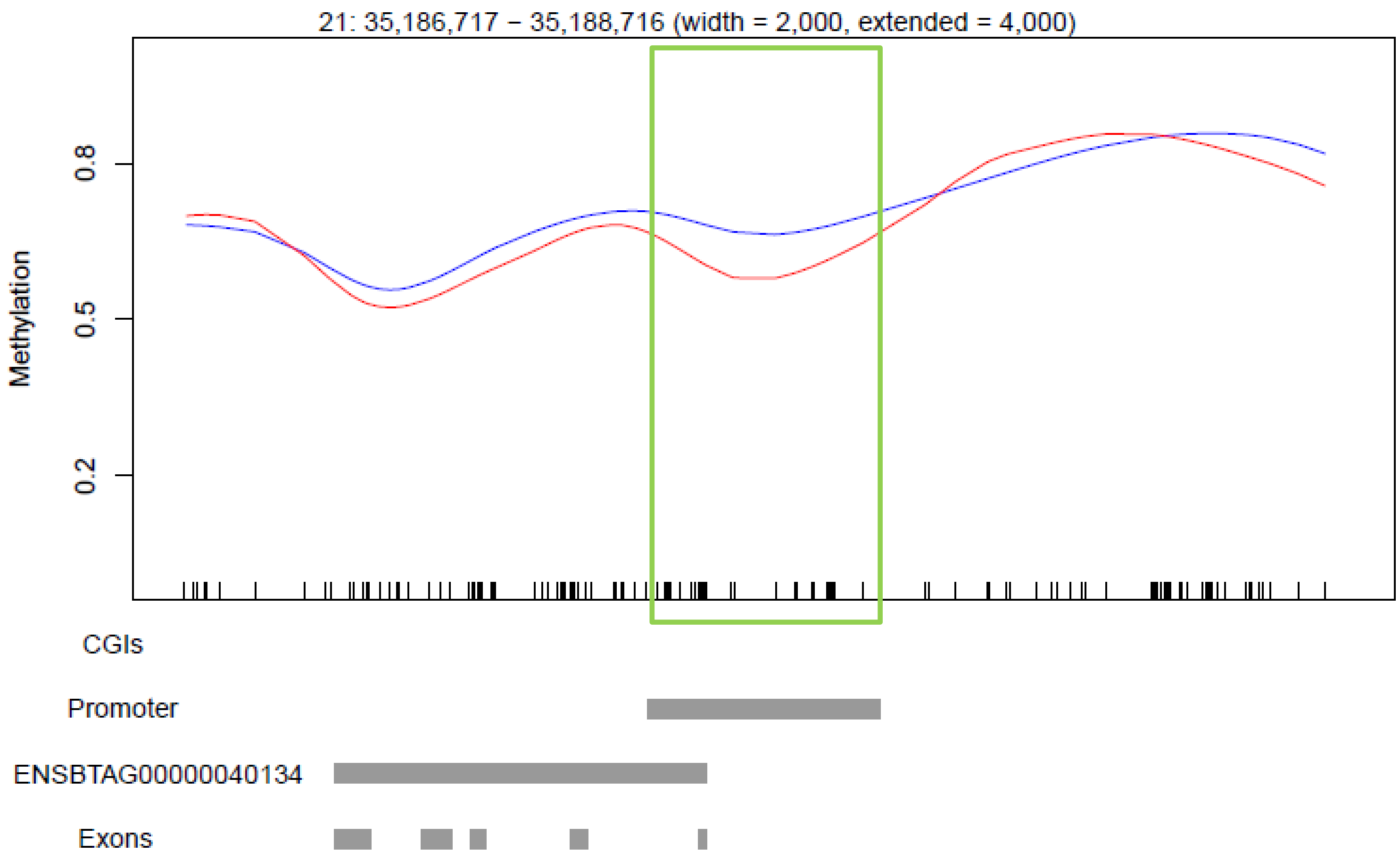
21: 35,186,717 − 35,188,716 (width = 2,000, extended = 4,000)
Methylation
0.8
0.5
0.2
CGIs
Promoter
ENSBTAG00000040134
Exons

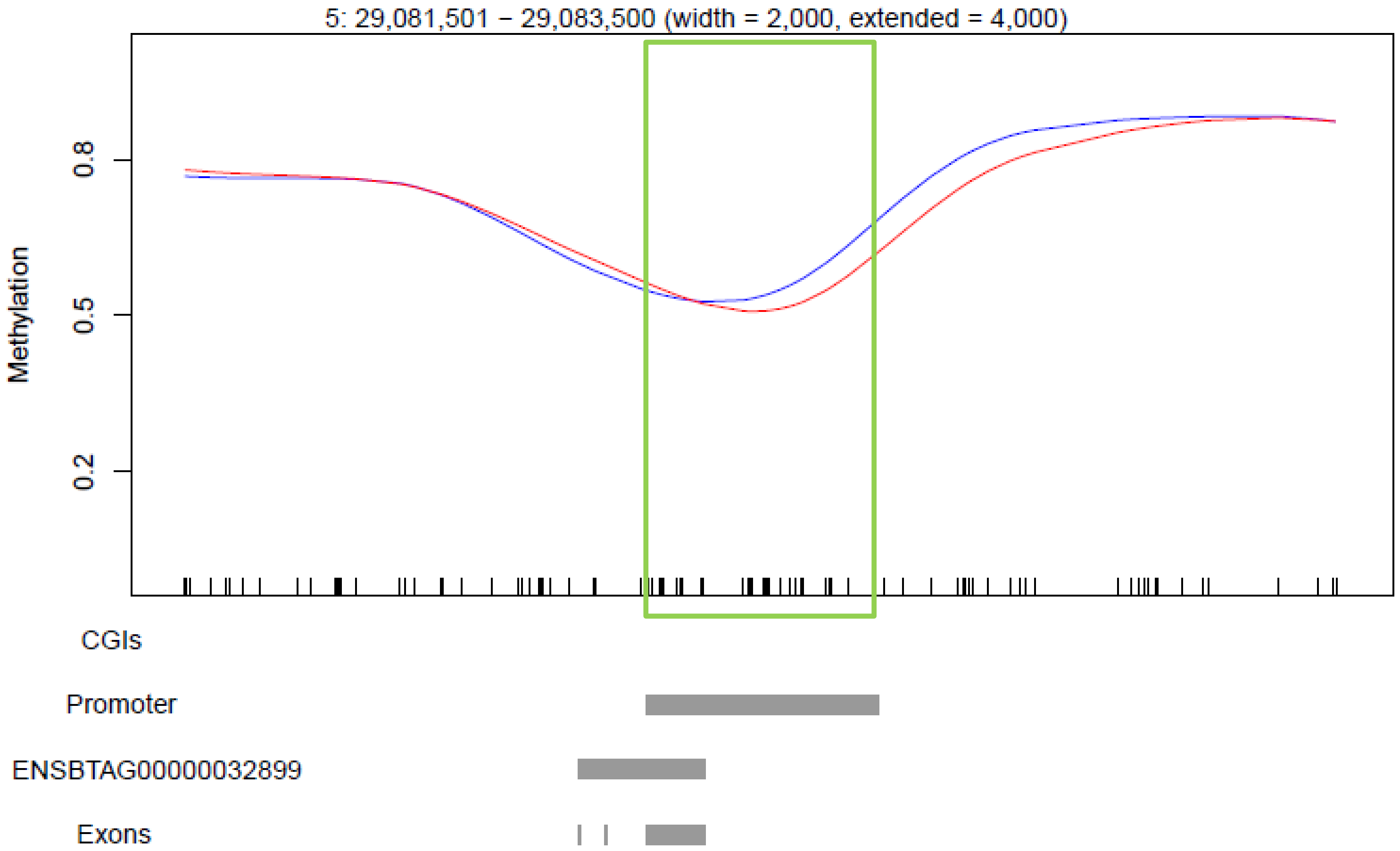
5: 29,081,501 – 29,083,500 (width = 2,000, extended = 4,000)
Methylation
0.2
0.5
0.8
CGIs
Promoter
ENSBTAG00000032899
Exons

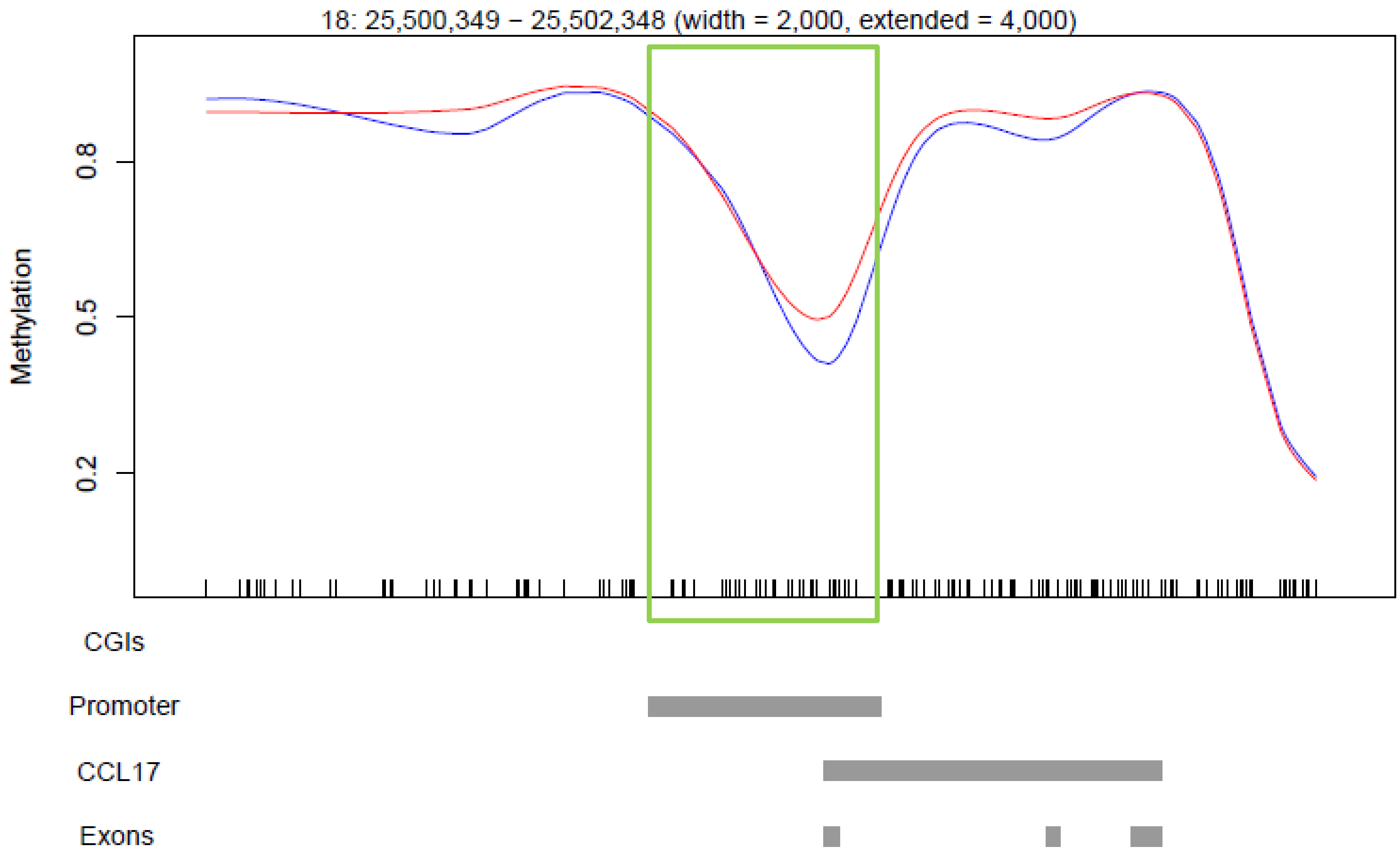
18: 25,500,349 − 25,502,348 (width = 2,000, extended = 4,000)
Methylation
0.2
0.5
0.8
CGIs
Promoter
CCL17
Exons

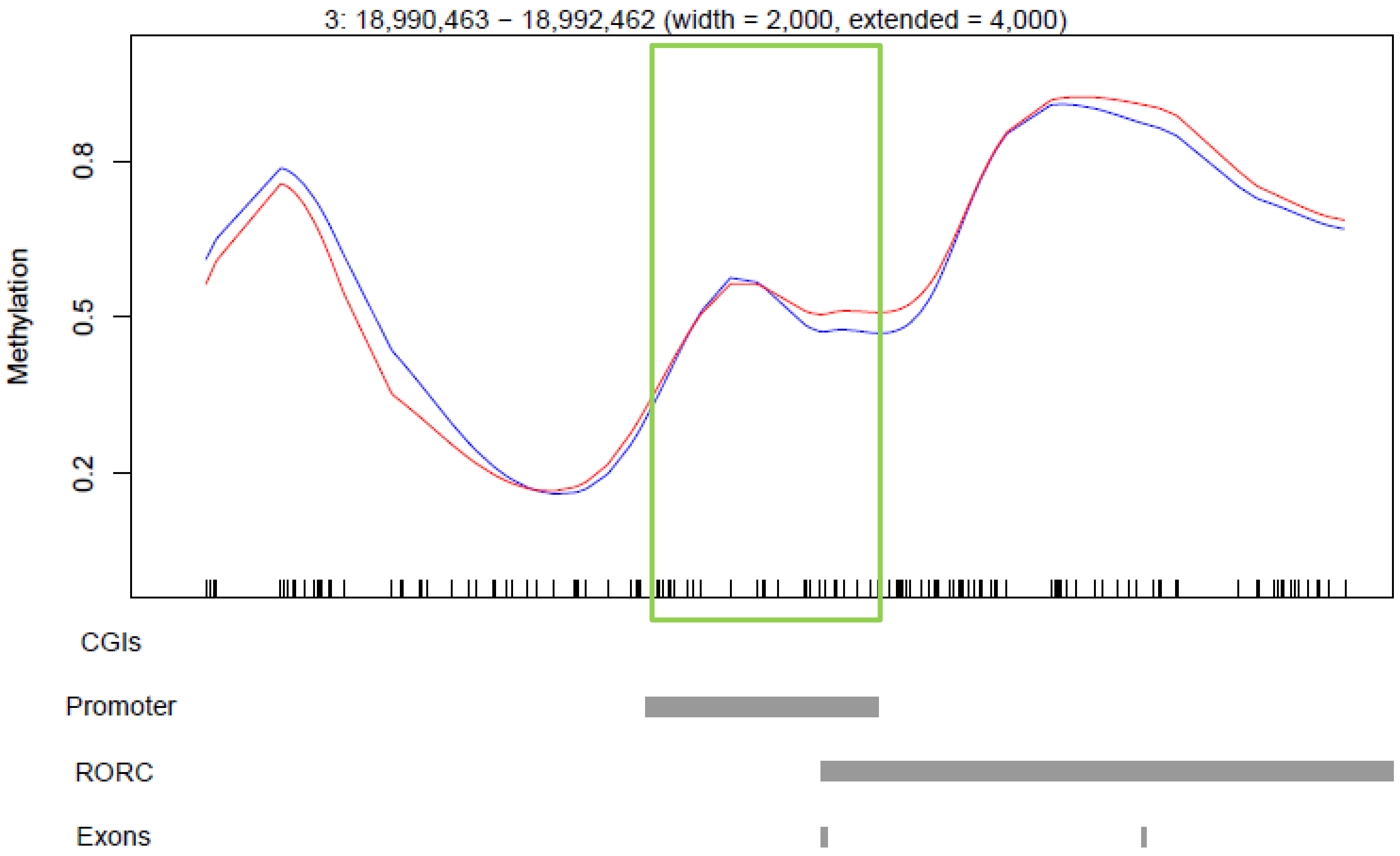
3: 18,990,463 – 18,992,462 (width = 2,000, extended = 4,000)
Methylation
0.8
0.5
0.2
CGIs
Promoter
RORC
Exons

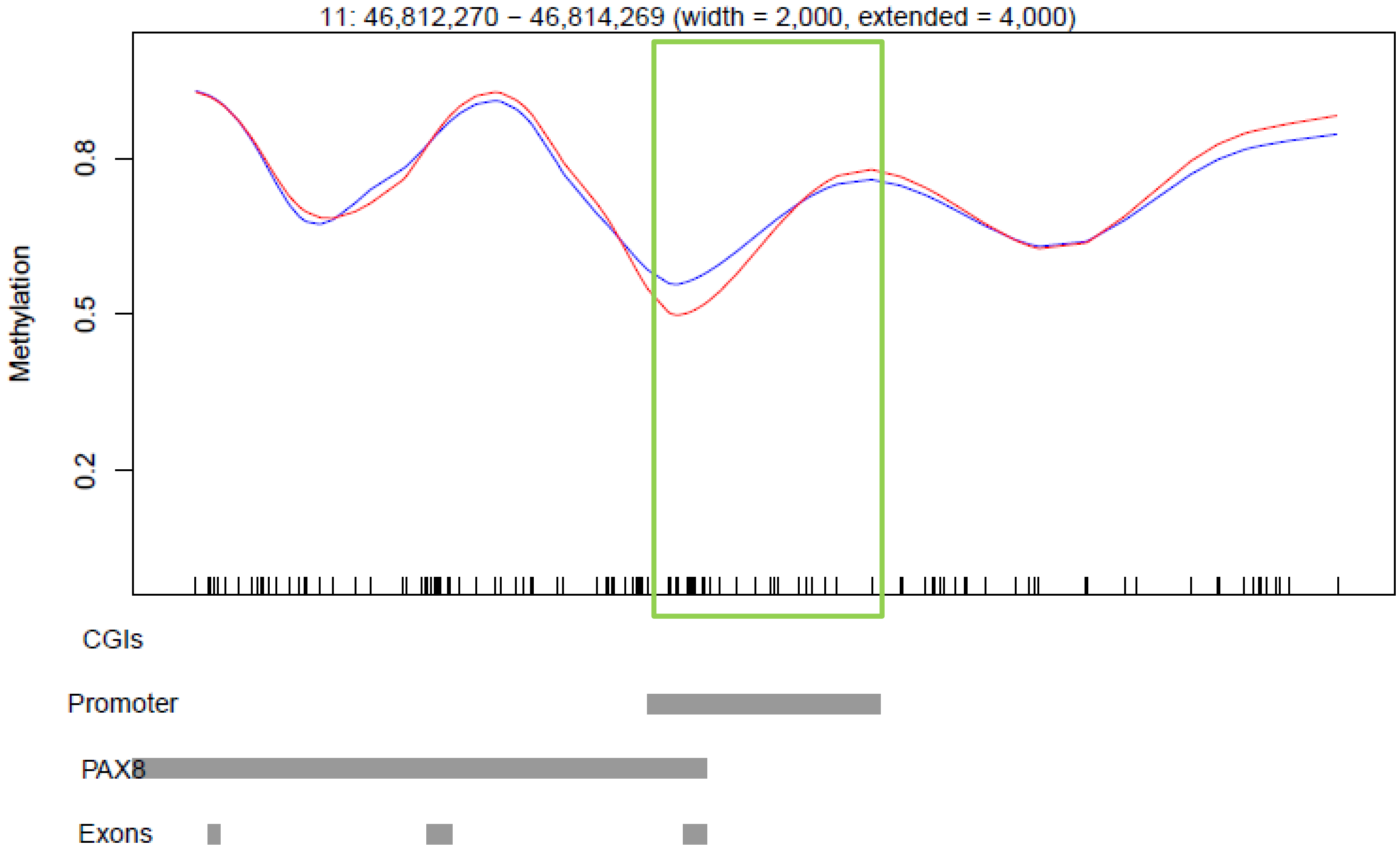
11: 46,812,270 − 46,814,269 (width = 2,000, extended = 4,000)
Methylation
0.2
0.5
0.8
CGIs
Promoter
PAX8
Exons

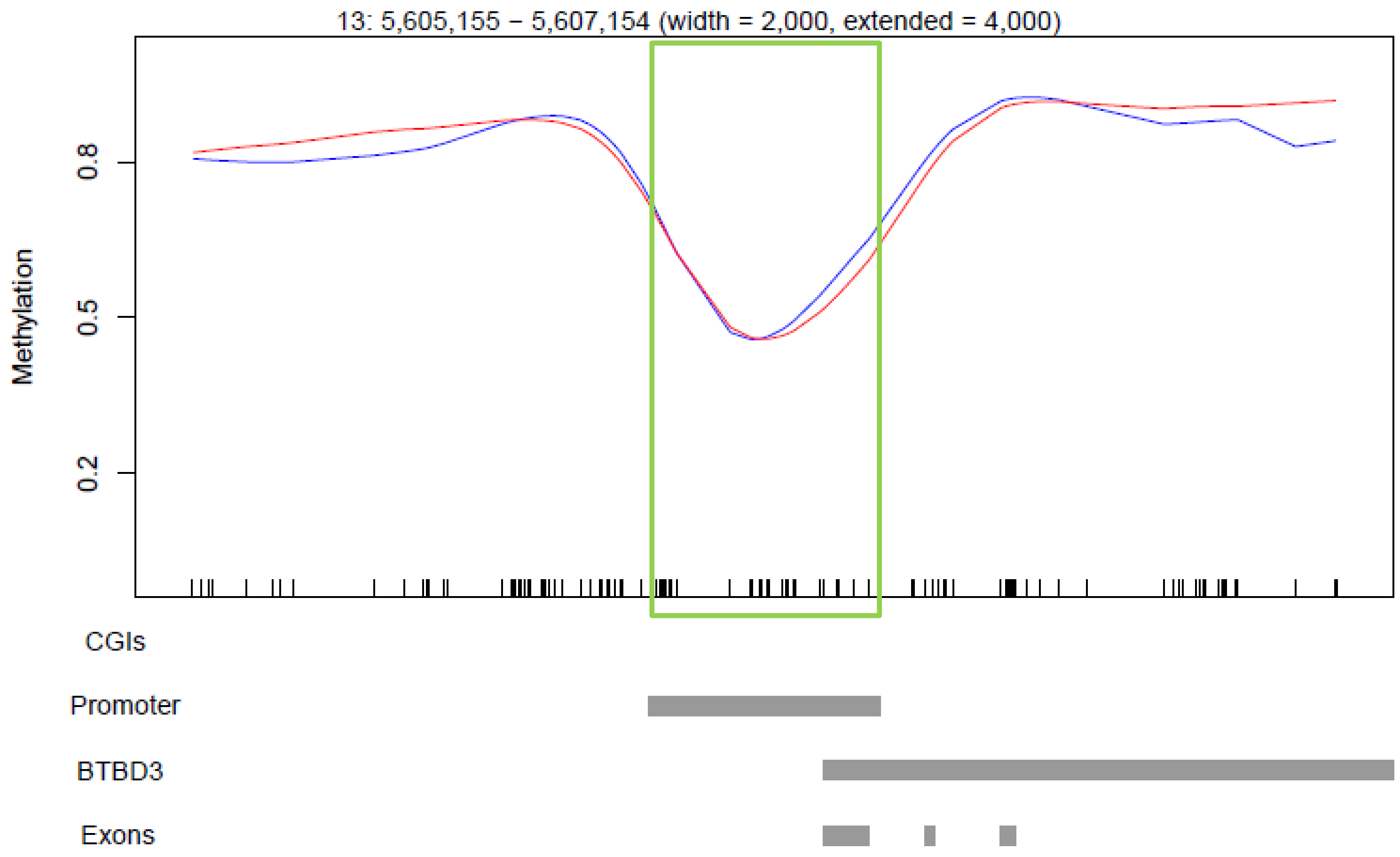
13: 5,605,155 – 5,607,154 (width = 2,000, extended = 4,000)
Methylation
0.8
0.5
0.2
CGIs
Promoter
BTBD3
Exons

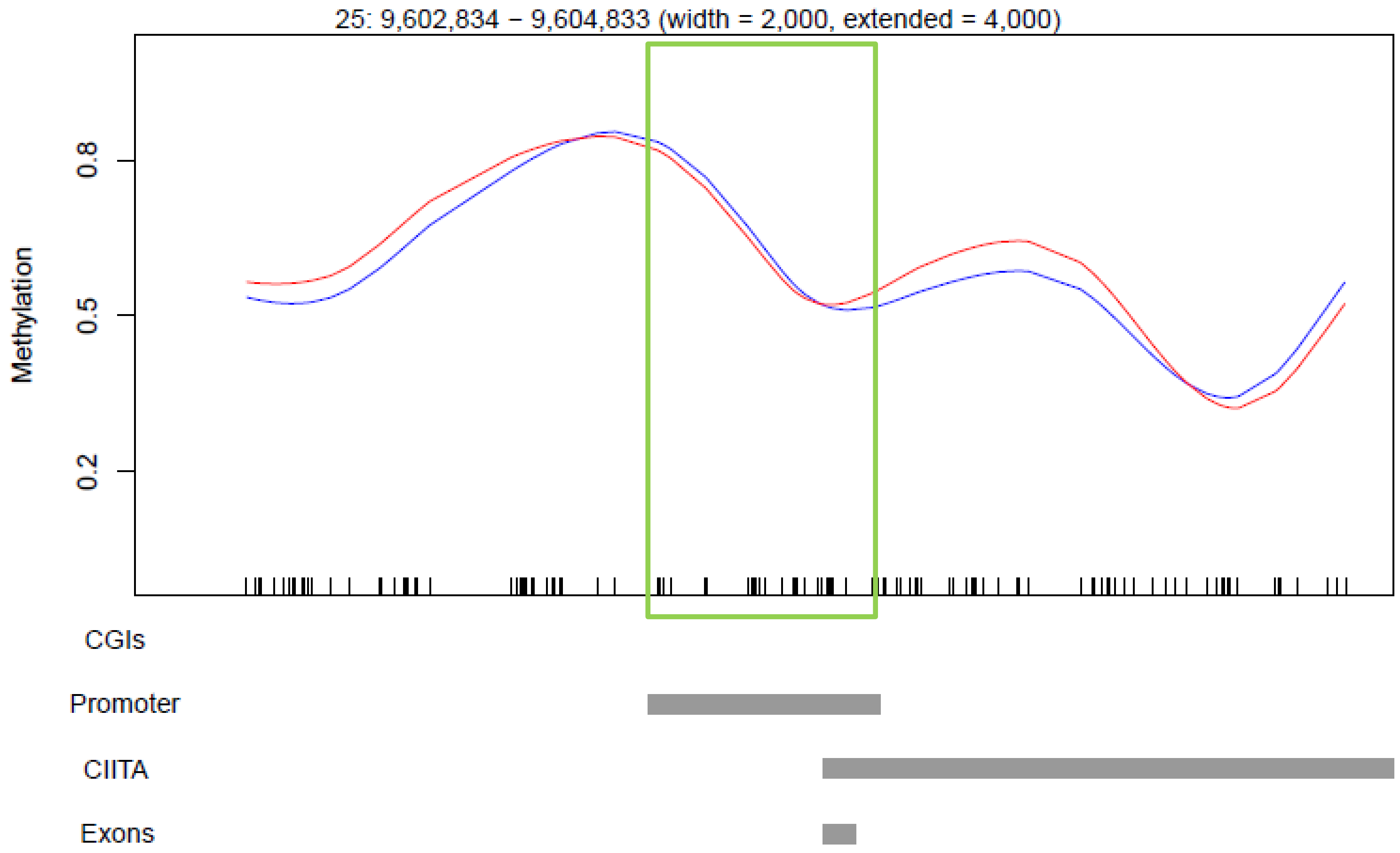
25: 9,602,834 − 9,604,833 (width = 2,000, extended = 4,000)
Methylation
0.8
0.5
0.2
CGIs
Promoter
CIITA
Exons

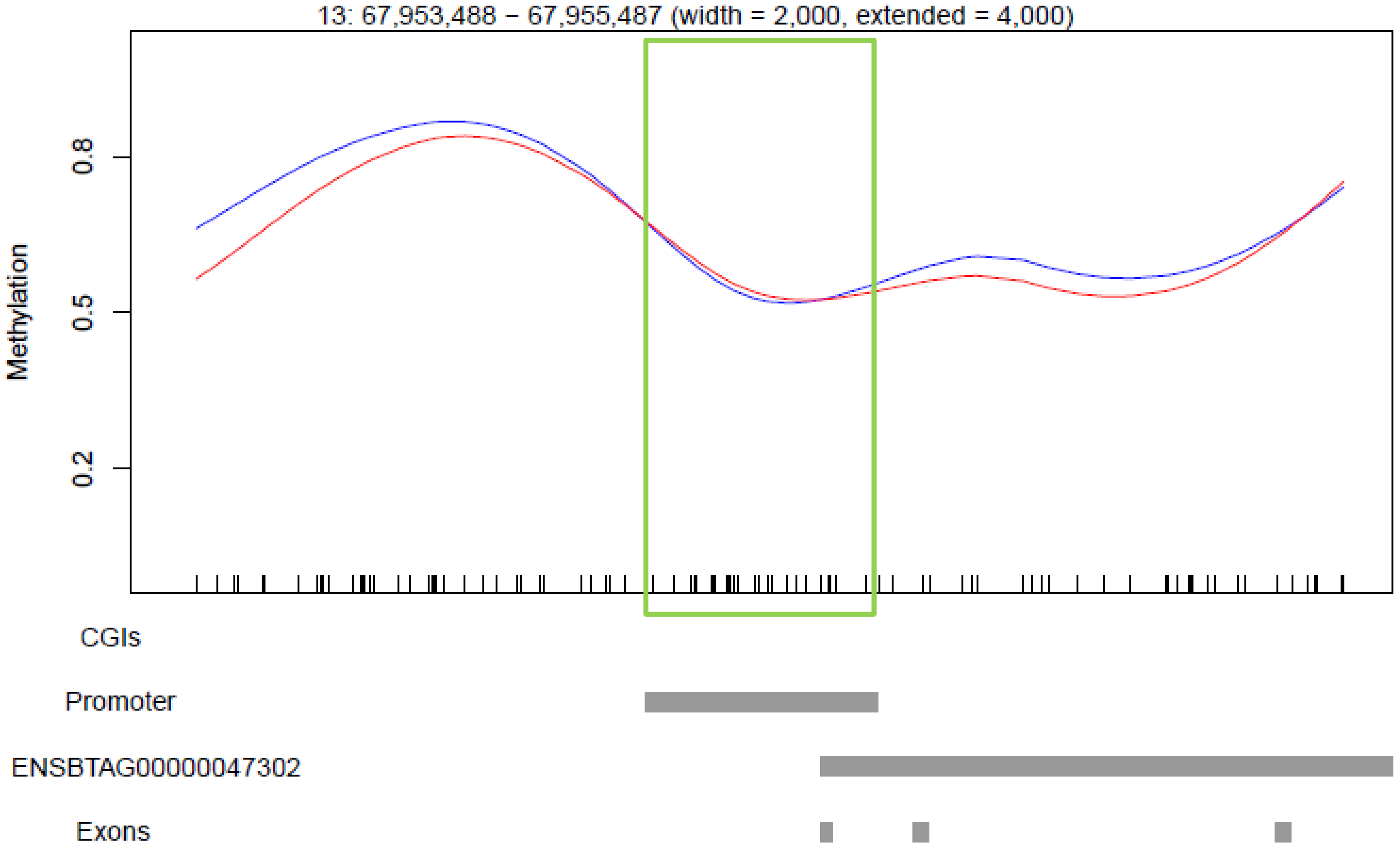
13: 67,953,488 − 67,955,487 (width = 2,000, extended = 4,000)
Methylation
0.8
0.5
0.2
CGIs
Promoter
ENSBTAG00000047302
Exons

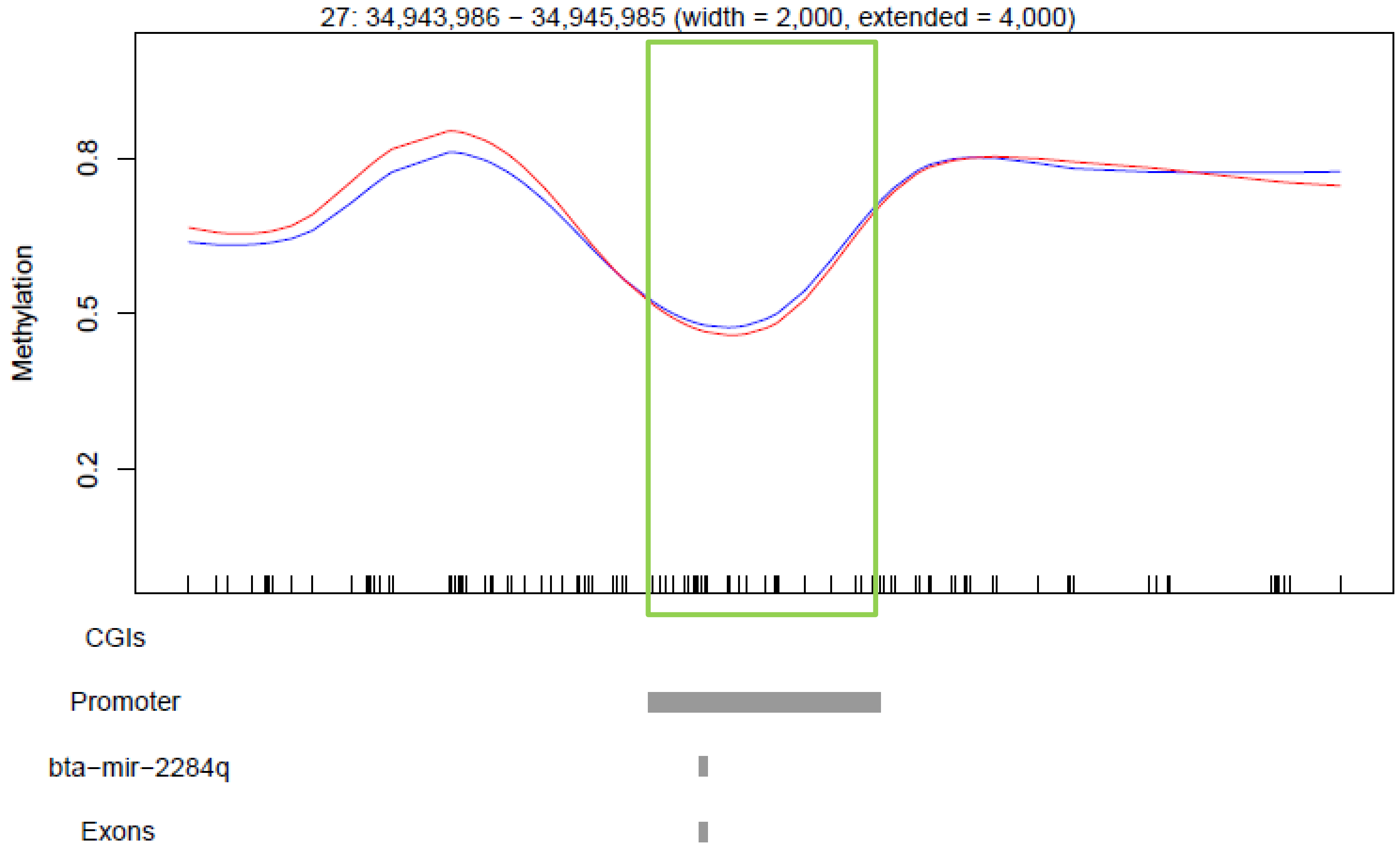

27: 34,943,986 − 34,945,985 (width = 2,000, extended = 4,000)
Methylation
0.8
0.5
0.2
CGIs
Promoter
bta−mir−2284q
Exons

Supplementary Table 1. Quality metrics for read quality trimming and adapter filtering. Quality trimming and adapter filtering were performed using Trim Galore!. Metrics reported include count of reads processed, count of reads containing adapter sequence, count of nucleotides quality-trimmed, count of reads passed quality trimming, proportion of A/T/C/G in the base preceding trimmed adapter sequences, and count of read mates passing trimming without their paired read mate. (See accompanying supplementary data file).

Supplementary Table 2. Quality metrics for raw sequencing reads. Quality metrics were computed using FASTQC. The resulting PASS/FAIL status is reported for all metrics. Pivot tables summarise the count of PASS/FAIL results by infection status, bisulfite treatment, individual sample, and read mate. (See accompanying supplementary data file).

Supplementary Table 3. Quality metrics for alignment of quality-filtered read pairs. Alignment of read pairs that passed quality filtering was performed using bismark. Metrics reported include alignment efficiency (*i.e.*, proportion of read pairs aligned to a unique locus), mapped to multiple loci, and unmapped, proportion of first read mapped to forward and reverse strand, count of methylated and unmethylated cytosines by context, and proportion of methylated cytosines by context. (See accompanying supplementary data file).

Supplementary Table 4. Quality metrics for deduplication of aligned read pairs. Deduplication of aligned read pairs was performed using the script *deduplicate_bismark*. Metrics reported include count of aligned read pairs, count of aligned read pairs removed by deduplication, proportion of aligned read pairs removed by deduplication and count of distinct genomic positions affected by deduplication. (See accompanying supplementary data file).

Supplementary Table 5. Table of differential methylation statistics for candidate differentially methylated regions (DMRs). Candidate DMRs were identified as genomic

regions including at least three loci with absolute *t*-statistics greater than 4.6 and a mean difference in methylation level (across samples and loci) greater than 10 % between the two groups (Sheet “DMRs”). The analysis was repeated after randomisation of the sample labels (Sheet “DMRs – Randomised”). The count and proportion of candidate DMRs in either direction (*i.e.*, hyper/hypo-methylated) were compared between the original and randomised analyses (Sheet “Direction”). (See accompanying supplementary data file).

Supplementary Table 6. Count and Gene Ontology analysis of gene promoters within discrete ranges of methylation levels. Gene promoters displaying a methylation level within 0-1%, 1-10%, 10-20%, 20-33%, 33-66%, 66-80%, 80-90%, 90-99%, and 99-100% were counted and analysed for Gene Ontology enrichment. Result for Biological Process (BP), Molecular Function (MF), and Cellular Component (CC) are reported together for each discrete range of methylation level. (See accompanying supplementary data file).
