# Supplementary material for: The bovine alveolar macrophage DNA methylome is resilient to infection with Mycobacterium bovis
Source: Sci Rep. 2019 Feb 6;9:1510. doi: 10.1038/s41598-018-37618-z (PMC6365515; doi:10.1038/s41598-018-37618-z)
Supplement: Supplementary file 3 — Supplementary Table Legends [file 41598_2018_37618_MOESM3_ESM.pdf]

Supplementary Table 1. Quality metrics for read quality trimming and adapter filtering. Quality trimming and adapter filtering were performed using Trim Galore!. Metrics reported include count of reads processed, count of reads containing adapter sequence, count of nucleotides quality-trimmed, count of reads passed quality trimming, proportion of A/T/C/G in the base preceding trimmed adapter sequences, and count of read mates passing trimming without their paired read mate. (See accompanying supplementary data file).

Supplementary Table 2. Quality metrics for raw sequencing reads. Quality metrics were computed using FASTQC. The resulting PASS/FAIL status is reported for all metrics. Pivot tables summarise the count of PASS/FAIL results by infection status, bisulfite treatment, individual sample, and read mate. (See accompanying supplementary data file).

Supplementary Table 3. Quality metrics for alignment of quality-filtered read pairs. Alignment of read pairs that passed quality filtering was performed using bismark. Metrics reported include alignment efficiency (*i.e.*, proportion of read pairs aligned to a unique locus), mapped to multiple loci, and unmapped, proportion of first read mapped to forward and reverse strand, count of methylated and unmethylated cytosines by context, and proportion of methylated cytosines by context. (See accompanying supplementary data file).

Supplementary Table 4. Quality metrics for deduplication of aligned read pairs. Deduplication of aligned read pairs was performed using the script *deduplicate_bismark*. Metrics reported include count of aligned read pairs, count of aligned read pairs removed by deduplication, proportion of aligned read pairs removed by deduplication and count of distinct genomic positions affected by deduplication. (See accompanying supplementary data file).

Supplementary Table 5. Table of differential methylation statistics for candidate differentially methylated regions (DMRs). Candidate DMRs were identified as genomic

regions including at least three loci with absolute *t*-statistics greater than 4.6 and a mean difference in methylation level (across samples and loci) greater than 10 % between the two groups (Sheet "DMRs"). The analysis was repeated after randomisation of the sample labels (Sheet "DMRs – Randomised"). The count and proportion of candidate DMRs in either direction (*i.e.*, hyper/hypo-methylated) were compared between the original and randomised analyses (Sheet "Direction"). (See accompanying supplementary data file).

Supplementary Table 6. Count and Gene Ontology analysis of gene promoters within discrete ranges of methylation levels. Gene promoters displaying a methylation level within 0-1%, 1-10%, 10-20%, 20-33%, 33-66%, 66-80%, 80-90%, 90-99%, and 99-100% were counted and analysed for Gene Ontology enrichment. Result for Biological Process (BP), Molecular Function (MF), and Cellular Component (CC) are reported together for each discrete range of methylation level. (See accompanying supplementary data file).
